# An exploratory study of CT radiomics using differential network feature selection for WHO/ISUP grading and progression-free survival prediction of clear cell renal cell carcinoma

**DOI:** 10.3389/fonc.2022.979613

**Published:** 2022-10-27

**Authors:** Fu Yin, Haijie Zhang, Anqi Qi, Zexuan Zhu, Liyang Yang, Ge Wen, Weixin Xie

**Affiliations:** ^1^ College of Electronics and Information Engineering, Shenzhen University, Shenzhen, China; ^2^ Medical Imaging Department, Nanfang Hospital, Southern Medical University, Guangzhou, China; ^3^ Center of Positron Emission Tomography-Computed Tomography (PET/CT), Shenzhen Second People’s Hospital, Shenzhen, China; ^4^ College of Computer Science and Software Engineering, Shenzhen University, Shenzhen, China

**Keywords:** clear cell renal carcinoma, radiomics features, differential network feature selection, WHO/ISUP grade, progression-free survival

## Abstract

**Objectives:**

To explore the feasibility of predicting the World Health Organization/International Society of Urological Pathology (WHO/ISUP) grade and progression-free survival (PFS) of clear cell renal cell cancer (ccRCC) using the radiomics features (RFs) based on the differential network feature selection (FS) method using the maximum-entropy probability model (MEPM).

**Methods:**

175 ccRCC patients were divided into a training set (125) and a test set (50). The non-contrast phase (NCP), cortico-medullary phase, nephrographic phase, excretory phase phases, and all-phase WHO/ISUP grade prediction models were constructed based on a new differential network FS method using the MEPM. The diagnostic performance of the best phase model was compared with the other state-of-the-art machine learning models and the clinical models. The RFs of the best phase model were used for survival analysis and visualized using risk scores and nomograms. The performance of the above models was tested in both cross-validated and independent validation and checked by the Hosmer-Lemeshow test.

**Results:**

The NCP RFs model was the best phase model, with an AUC of 0.89 in the test set, and performed superior to other machine learning models and the clinical models (all *p <*0.05). Kaplan-Meier survival analysis, univariate and multivariate cox regression results, and risk score analyses showed the NCP RFs could predict PFS well (almost all *p* < 0.05). The nomogram model incorporated the best two RFs and showed good discrimination, a C-index of 0.71 and 0.69 in the training and test set, and good calibration.

**Conclusion:**

The NCP CT-based RFs selected by differential network FS could predict the WHO/ISUP grade and PFS of RCC.

## Introduction

The nuclear grade of clear cell renal cell carcinoma (ccRCC) is strongly related to 5-year survival time, with higher grades associated with shorter survival (1, 2) and higher risk for recurrence after partial nephrectomy (3). The World Health Organization/International Society of Urological Pathology (WHO/ISUP) (4) grading system is a new four-level system commonly used in clinical which has improved the interobserver reproducibility, and is easier to apply and more clinically relevant, as well as a better independent prognostic factor (5), compared to the former Fuhrman grading system. However, earlier studies have shown no significant difference in the survival rate between grade 1 and grade 2 RCC (6, 7) and between grade 3 and grade 4 RCC (1, 2). Therefore, some scholars tend to simplify it into low-grade and high-grade lesions. In terms of clinical decision-making, patients with low-grade RCC may be treated relatively conservatively, such as through nephron-saving surgery, radiofrequency ablation, or active surveillance. In contrast, patients with high-grade RCC may receive more radical interventions and closer follow-up (8). Therefore, preoperative WHO/ISUP grading is very helpful in guiding clinical decision-making (8, 9).

Histopathological examination is the standard method to determine the WHO/ISUP grade of ccRCC. However, needle biopsy accuracy remains controversial (10, 11), and tumor grade is often underestimated (12–14). At the same time, the biopsy is invasive, associated with complications, and may be limited by tumor location and timely status. Therefore, a new noninvasive method to preoperatively predict the pathological grade of ccRCC would be of clinical merit. Studies have shown that radiomics can be used noninvasively to predict the presence of oncogenes, prognosis, and the effectiveness of different treatments (15, 16). Accumulating evidence has shown that radiomics features (RFs) are useful for predicting the pathological grade of RCC (17).

In radiomics, the number of features is usually larger than the experiment samples, which is easy to overfit and hinders the model’s prediction. Therefore, feature selection (FS) methods are necessary. Traditional FS methods pick up a subset of features based on specific criteria, removing redundant, irrelevant, and noisy data. Based on a reasonable assumption, the RFs used to predict grade very well could also perform well on progression free survival (PFS) prediction, as the grade is strongly related to the prognosis. However, unfortunately, we are unsure about that, as most studies only focus on a single experiment objective: predicting the grade or the PFS, which causes the support for the assumption not enough and the interpretability of RFs poor. Therefore, designing a suitable FS method should make the selected RFs that can not only make accurate grade predictions but may also decipher the survival mechanisms associated with prognosis remains a significant challenging problem (18).

Several machine learning FS methods have been used in earlier studies to analyze image data, including Lasso regression (LR), decision tree (DT), support vector machine (SVM), convolution neural network (CNN), and random forest. Although the above methods have been successfully used to select RFs and build prediction models, they have a few limitations. For example, some methods select at most *n* variables before it saturates (19). However, the most number ‘*n*’ is not easy to decide. For example, the sparsity ratio λ in LR and penalty coefficient C in SVM should be chosen based on the prior empirical knowledge of the researchers or complicated cross-validation, which is not easy and very time-consuming. Moreover, suppose there is a group of features among which the pairwise correlations are very high. In that case, they tend to arbitrarily select only one feature from the group, which means some important RFs will be lost. It could work to improve the model prediction performance. However, the interpretability of the RFs should be selected was not good enough, as choosing only one from the redundant features and removing the rest could lose much helpful information about the RFs. At last, most machine learning FS methods were wrapped-based; improving the model’s performance in the training sets was their priority. Thus, the generalization performance of the models was easy to overfit. In the meantime, the performance of the existing machine learning FS methods is not stable when dealing with small and unbalanced sample size problems. Therefore, a more reliable FS method is urgently needed. The ideal RFs should not only have an accurate WHO/ISUP grade classification but also have some interpretable biological characteristics, such as PFS.

Differential network analysis based on network theory and related methodologies has shown outstanding robustness in analyzing various forms of large-scale data, which is evident in its ability to identify biomarkers (20). Most of the existing machine learning FS methods are a feature-centric analytic approach that assesses changes in individual features to a target. In contrast, differential network FS is a network-centric analytical approach that focuses on detecting the changes in a feature’s associations with other features—comparing the difference between two different populations or groups’ networks to select features. It is especially effective in detecting essential features that have less dramatic changes for specific experiments and show outstanding performance in dealing with small and unbalanced sample problems.

The correlation networks are widely used in constructing the networks, such as Pearson correlation, Euclidean distance, Spearman rank correlation, and so on. It should be noted that this correlation is between features, unlike in some filter FS methods between features and target labels. However, the biggest problem of such network constructing methods is that they could be misleading in reflecting the correlation of two features as it ignores the influence of the rest ones. The maximum-entropy probability model (MEPM) (21) is proposed to solve such a problem. It finds that inverting the matrix of covariances of features (Pearson correlation) could describe the correlations that remain once the indirect effects are removed, thereby providing a more robust description of the interactions between features.

However, there was no literature report on its application in the search for imaging RFs. For these reasons, this study aimed to investigate the feasibility of predicting the WHO/ISUP grade and PFS of ccRCC from the RFs based on the differential network FS using the MEPM. Furthermore, this paper expected to find evidence that the selected RFs of the WHO/ISUP grade prediction model were related to PFS of ccRCC to make the radiomics prediction models with more interpretable biological information through our new FS method.

## Material and methods

### Patients

This retrospective study was approved by the Ethics Committee of Southern Medical University, and because of the retrospective nature of the analysis, the requirement of informed patient consent was waived.

Medical records and picture archiving and communication systems were searched for patients with RCC treated at our hospital from March 2011 to March 2016. The age, gender, maximum tumor size, clinical stage, symptom, growth pattern, histological subtype, WHO/ISUP nuclear grade, and PFS were collected.

**Figure 1:**
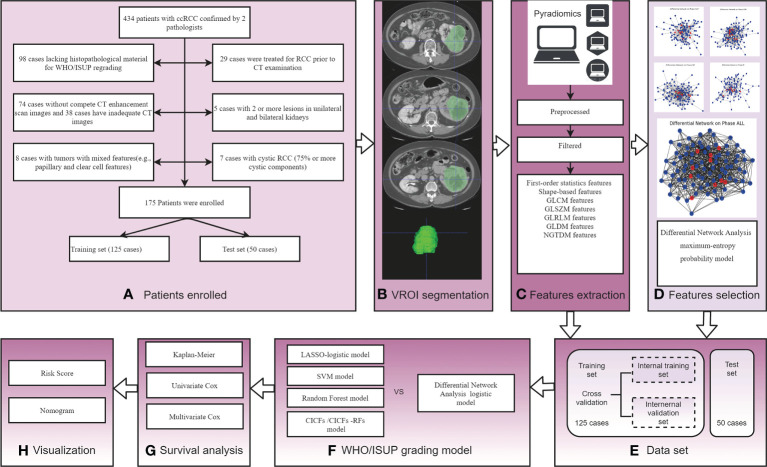
The experimental process of this study. **(A)** Patient enrollment flow charts; **(B)** Schematic diagram of image segmentation; **(C)** Schematic diagram of feature extraction; **(D)** Schematic diagram of differential networks features selection method; **(E)** Experimental Data set. **(F)** WHO/ISUP grading model construction and comparison; **(G)** Survival analysis by Kaplan-Meier survival, univariate, and multivariate Cox analysis; **(H)** Visualization risk score and nomogram.

There were 434 patients with ccRCC confirmed by two pathologists were preliminarily enrolled. Exclusion criteria: 1) Patients lacking histopathological material for WHO/ISUP re-grading (98 cases); 2) Patients who were treated for RCC before CT examination (29 cases); 3) Patients without compete non-contrast phase (NCP), cortico-medullary phase (CMP), nephrographic phase (NP), and excretory phase (EP) phases CT scan (74 cases) and patients with inadequate quality images (38 cases); 4) Patients with 2 or more lesions in unilateral (2 cases) or bilateral (3 cases) kidneys; 5) Patients with tumors with mixed features (8 cases) and cystic RCC (75% or more cystic components) (7 cases). Finally, 175 Patients were enrolled and randomly divided into a training set (125 cases) and a test set (50 cases). Patient enrollment and experimental flow charts are shown in **Figures 1A, E**.

In performing survival analysis, the follow-up information is selected based on the criteria as follows: 1) survival information was obtained by telephone follow-up visits for all patients for at least 5 years after surgery; 2) tumor recurrence included *in situ* recurrence and distant metastasis; 3) diagnosis was mainly based on imaging examination comparison, and the follow-up deadline was June 2021;4) PFS was selected as the clinical endpoint.

### CT parameters

A 64 multiprobe spiral CT scanner (Siemens, Somatom Definition CT scanner, 121 patients) and a 256 multiprobe spiral CT scanner (Philips, Brilliance ICT, 54 patients) were performed with patients. The range included both kidneys and masses in the supine position and a breath-holding scan. The scanning parameters were: tube voltage = 120 kV; tube current = 150-320 mA; slice thickness = 5 mm; layer spacing = 5 mm; field of view (FOV) = 360 mm; matrix = 512×512. Spiral scanning and thin-layer reconstructions were performed for all 4 stages. After the NCP scan, the contrast agent was injected into the anterior cubital vein with a high-pressure syringe at a dose of 2 ml/kg and an injection rate of 2.5 ml/s. CMP, NP, and EP scanning were started at 30-35 s, 60-70 s, and 190-200 s, respectively.

### Images segmentation and radiomics feature extraction

The tumor volume region of interest (VOI) was segmented by 2 radiologists with 10-year and 15-year experience using ITK-snap software (www.itk-snap.org). Four phases (NCP, CMP, NP, and EP) VOI segmented images were obtained for each patient, and its boundary was kept about 2 mm away from the tumor edge to reduce interference from adjacent tissues (22). When the boundary of the tumor was not clear, the boundary of the CMP image was compared for segmentation. Images segmentation examples are shown in **Figure 1B**.

The segmented images were first preprocessed, including resampling, normalization, and filtering to remove noise. Then the RFs were extracted from segmented images using the PyRadiomics computing platform. Features extraction is shown in **Figure 1C**. The initial setting of the Pyradiomics are as follows: binWidth = 25, label= 1, interpolato r= ‘sitkBSpline’, resampledPixelSpacing = ‘None’, weightingNorm = ‘None’.

To assess feature robustness, we conducted a test-retest study. Two physicians (Doctor A 10 years, and Doctor B 9 years experience) individually contoured the ROIs in the random 30 images. Intraclass correlation coefficient (ICC) was used to test the stability between Doctor A and Doctor B groups, and the results showed that ICC was> 0.75 between groups. One week later, Doctor A repeated the same procedure to assess the reproducibility, and the results showed that ICC > 0.75 within the group (Doctor A). The results between groups and within the group suggest the segmentation was consistent, and the remaining image segmentation was performed by Doctor A.

### Features selection

**Figure 2:**
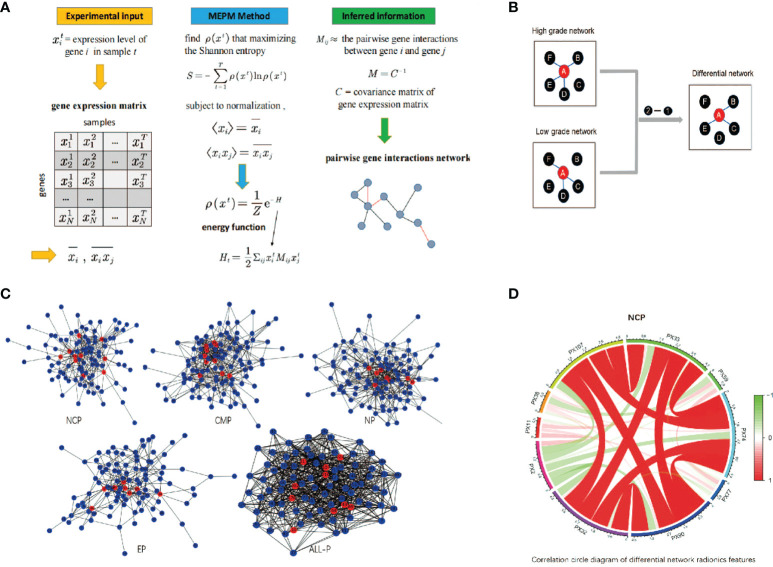
The schematic diagram of differential network feature selection. **(A)** An example of GRN inference using maximum-entropy probability model (MEPM); **(B)** An example of using differential networks analysis; **(C)** The differential networks using MEPM of the non-contrast phase (NCP), cortico-medullary phase (CMP), nephrographic phase (NP), excretory phase (EP), and all phase (ALL-P), where the red nodes were the selected RFs; **(D)** Correlation circle diagram of the RFs of the NCP model.

A differential network FS using MEPM was proposed in this study. The flow chart is shown in **Figure 2**. At first, five control groups were constructed based on different phases of RFs (NCP, CMP, EP, MP, ALL). i.e., the NCP group consists of all the samples with only NCP RFs, and the ALL group consists of all the samples with all 4 phase RFs. All samples in each group were marked based on their WHO/ISUP grade (high-grade or low-grade). Then, high-grade and low-grade networks of each group were constructed using MEPM based on the corresponding samples (**Figure 2A**). i.e., NCP high-grade network was constructed only using the samples in the NCP group marked high grade. After that, each phase’s differential network of the high-low grade was constructed after comparing the differential topology structure between their high-grade network and low-grade network, i.e., network2 - network1 (**Figure 2B**). To be more specific, For example, features A and F were linked in both high-grade and low-grade networks, which means the structure of feature A to feature in these two networks was no different. So there was no link between feature A and feature F in its high-low differential network. At last, the RFs for each control group were selected based on the node degree histogram in their high-low differential network (**Figure 2C**), i.e., the nodes (RFs) which had the highest degree in the network were considered the critical RFs (marked in red in **Figure 1D** and **Figure 2C**); in this case, the number of selected features was set to less than 15.

### MEPM networks construction

Let the state vector *x* = (*x*_1_, …, *x_N_*) denote the expression levels of the *N* features in an experiment, and a series of *T* measurements then has associated with its *T* distinct state vectors. Let *ρ*(*x*) denote the probability that the genome is in the arbitrary state *x*. We determine *ρ*(*x*) by maximizing the Shannon entropy


(1) $$S=-\rho (\vec{x)}\ln(\vec{x)}$$

subject to the *ρ*(*x*) is normalized


(2) $$\sum_{\vec{x}}\rho (\vec{x)}=1$$

first moment, <*x_i_* >, and second moment, <*x_i_*, *x_j_* >


(3) $$<x_{i}>=\sum_{\vec{x}}\rho \vec{\left(x_{i}\right)}x_{i}=\frac{1}{T}\sum_{k=1}^{T}x_{i}^{k}$$

(4) $$<x_{i},x_{j}>=\sum_{\vec{x}}\rho \vec{\left(x\right)}x_{i}x_{j}=\frac{1}{T}\sum_{k=1}^{T}x_{i}^{k}x_{j}^{k}$$

Eq. (2) provides the normalization condition that the probabilities of all observable states sum to 1. Eqs. (3) and (4) ensure that the distribution *ρ*(*x*) preserves the mean expression level of each gene and the correlations between genes. This procedure leads to a Boltzmann-like distribution:


$$\rho \left(x\right)\sim e^{-H}$$

where


$$\text{H = }\frac{1}{2}\sum_{ij}x_{i}M_{ij}x_{j}$$

The elements of the matrix M are the effective pairwise gene interactions that reproduce the gene profile covariances exactly while maximizing the entropy of the system. The matrix of M can be obtained by inverting the matrix of their covariances C. This makes a substantial difference. The covariance matrix C reflects the unconditional correlation between features and, therefore, contains indirect effects. On the other hand, its inverse, i.e., M, describes the correlations that remain once the indirect effects are removed and thereby provides a more robust description of the interactions between features.

However, in the high dimensional setting where the number of features *p* is larger than the number of observations *n*, the empirical covariance matrix C is singular and so can’t be inverted to yield an estimate of M. Many MEPM-based methods have been proposed for inferring networks, including partial-correlation-based, likelihood-based, and mutual-information-based approaches.

In our case, we chose a multi-objective memetic algorithm to infer the MEPM networks (23, 24) and some other method like Glasso could get the similar results (25).

### Prediction model construction and performance comparison

The performance of all experiment models was explored and verified by 5 times hierarchical 5-fold cross-validation in the training set and independently valid in the test set. To be more specific, the training set was divided into an internal training set and validation set (4:1) in each 5-fold cross-validation. The data split is using the python package sklearn: ‘train_test_split’ and set the ‘stratify’ = result, which makes the classification ratio of data in the training set and the test set will be the same.

In this study, the NCP, CMP, NP, EP, and ALL models were constructed using Logistic regression based on the corresponding RFs. At first, each experiment model was constructed based on the corresponding selected RFs using the proposed differential network FS in the internal training set. For example, the selected RFs of the NCP model was taken in the internal training set and then constructed using Logistic regression. Then, the average performance of the experiment model in 5 times cross-validation in the internal training and validation sets was considered the final performance in the training and validation sets. At last, the best selected RFs of each model in cross-validation were tested in the test set.

According to the performance results in all data sets, the best of the above five models was selected as our WHO/ISUP grade prediction model. After that, the LASSO, SVM, and Random Forest models were constructed based on all four phase RFs as control models and compared with our model on the same dataset. More specifically, model training and performance testing follow the same processing mentioned above (**Figure 1F**). Lasso is implemented using the python package sklearn: ‘lassocv’, which could select penalty parameter adaptively; SVM is using the python package sklearn: ‘RFE’ and ‘SVM’; Random Forest is using the python package sklearn: ‘RandomForestClassifier’. We keep the default parameter values for all these methods. Then, the conventional image and clinical features mode (CICFs) is constructed using Logistic regression based on the clinic features. The CICFs-RFs model combined clinic features, and RFs were constructed as control models and compared with our model following the performance mentioned above test processing. Finally, the receiver operating characteristic (ROC), the area under curve ROC curve (AUC), precision, sensitivity, and accuracy were determined to estimate the performance of the above models. At last, the best prediction model was refit on the complete training set as the final WHO/ISUP grade prediction model.

### Survival analysis and performance comparison

Survival analysis was performed to explore more biological information about the selected RFs in the final WHO/ISUP grade prediction model and find whether they were related to PFS. At first, Kaplan-Meier analysis by converting the RFs into a dichotomous variable (high and low group) was used to estimate the selected RFs. Then, the univariate and multivariate Cox proportional hazard regression models were used to investigate the factors of RFs associated with PFS (**Figure 1G**). Independent variables with *p* < 0.05 in univariate results and multivariate were selected. After that, risk score analyses of ccRCC patients were used to describe the selected RFs. Finally, the selected RFs were used to build the final multivariate Cox regression model and visualized using nomograms (**Figure 1H**). The C-index of the final model was determined. The Hosmer-Lemeshow test was used to check the calibration.

### Statistical analysis

Continuous data were presented as mean ± standard deviation, and categorical data were presented as numbers and percentages (%). For comparisons of means between groups, Student’s independent t-test or Mann-Whitney U test was used, depending on the normality assumption. Categorical data were tested using the chi-square test or Fisher’s exact test (if an expected value ≤ 5 was found). In all analyses, a 2-tailed value of *p* < 0.05 was considered to indicate statistical significance. The surv_cutpoint function in the surviminer package finds the best cutoff value for a continuous variable and is used to predict PFS. Statistical analyses and mapping were performed by R software (version 4.2) with the ‘rms’, ‘proc’, ‘survival’, ‘rmda’, and ‘ggplot’ package.

## Result

### Patient and tumor characteristics

175 patients were included in the analysis, including 125 cases in the training set and 50 cases in the test set. **Table 1** summarizes the characteristics of the patients. 122 cases were diagnosed with low-grade ccRCC (WHO/ISUP grades 1 and 2) and 53 cases with high-grade ccRCC (WHO/ISUP grades 3 and 4). All characteristics of patients in the training set and the test set were no statistical difference (*p* > 0.05).

**Table 1:** Patient’s clinical characteristics between the training set and test set.

	Levels	Training set (N=125)	Test set (N=50)	*p*
Age (year, mean ± SD)		52.31 ± 14.51	52.06 ± 13.19%	0.915
Gender	Male	84 (67.2%)	31 (62.0%)	0.632
	Female	41 (32.8%)	19 (38.0%)	
Diameter (mm, median)		43.98	43.9	0.589*
WHO/ISUP	low	91 (72.8%)	31 (62.0%)	0.221
	high	34 (27.2%)	19 (38.0%)	
T	T1	97 (77.6%)	38 (76.0%)	0.541
	T2	16 (12.8%)	9 (18.0%)	
	T3	12 (9.6%)	3 (6.0%)	
N	N0	115 (92.0%)	47 (94.0%)	0.891
	N1	10 (8.0%)	3 (6.0%)	
TNM	Stage I	90 (72.0%)	37 (74.0%)	0.439
	Stage II	17 (13.6%)	9 (18.0%)	
	Stage III	18 (14.4%)	4 (8.0%))	
Symptom	no	65 (52.0%)	25 (50.0%)	0.943
	yes	60 (48.0%)	25 (50.0%)	
Grow pattern	Exophytic	40 (32.0%)	20 (40.0%)	0.241
	Mixed	60 (48.0%)	17 (34.0%)	
	Endophytic	25 (20.0%)	13 (26.0%)	
PFS (month, median)		56	66.5	0.122*

*p values were calculated by the Kruskal-Wallis test.

### Features extraction and selection

A total of 107 RFs were extracted from the 3D multiphase CT images of each phase of each patient: 18 first-order statistics features, 14 shape-based features, 24 gray level co-occurrence matrix (GLCM) features, 16 gray level size zone matrix (GLSZM) features, 16 gray level run length matrix (GLRLM) features, 14 gray level dependence matrix (GLDM) features, and 5 neighboring gray-tone difference matrix (NGTDM) features. A total of 428 (4×107) RFs were extracted from the 4-phase CT images.

Differences in scanner models should be verified as the dataset collected comes from two scanners. Thus, principal component analysis (PCA) was performed on the extracted features to plot data in the space of reduced dimensions (26). Visual inspection of **Supplement Figure 1** suggests the absence of batch effects. Furthermore the Kruskal–Wallis test, carried out on both the first and second main component scores, also confirmed the absence of clusters (PC 1 scores: p-value = 0.526 > 0.05 and PC 2 scores: p-value = 0.174 > 0.05).

Through the differential network FS, there were 10, 10, 8, 8, and 10 RFs were selected from NCP, CMP, NP, EP, and ALL-P in the complete training set, respectively. The differential networks of different phases are shown in **Figure 2C** where the red nodes represent the selected RFs. The designation, phase, abbreviation, classification, and description of RFs are shown in **Table 2**.

**Table 2:** The name and the abbreviation of the RFs of different phases using the differential network feature selection.

NCP (P)	CMP (A)	NP (V)	EP (D)	ALL-P
X107-Strength	X7-Maximum2D_DiameterSlice	X1-Elongation	X11-Sphericity	VX11-Sphericity
X77-Long Run Low Gray Level Emphasis	X8-Maximum3D_Diameter	X107-Strength	X1-Elongation	AX11- Sphericity
X59-Dependence Non-Uniformity Normalized	X11-Sphericity	X11-Sphericity	X45-Idn	VX70- Small Dependence Low Gray Level Emphasis
X74-High Gray Level Run Emphasis	X34-Cluster Shade	X21-Maximum	X35-Cluster Shade	AX70- Small Dependence Low Gray Level Emphasis
X2-Flatness	X35-Cluster Shade	X2-Flatness	X54-Sum Average	PX11-Sphericity
X11-Sphericity	X2-Flatness	X94-Low Gray Level Zone Emphasis	X90-High Gray Level Zone Emphasis	AX44- Idmn
X90-High Gray Level Zone Emphasis	X21-Maximum	X99-Small Area Low Gray Level Emphasis	X95-Size Zone Non-Uniformity	AX45- Idn
PX38-Correlation	X45-Idn	X48-Inverse Variance	X103-Busyness	DX104- Coarseness
X33-Autocorrelation	X26-Range			VX1- Elongation
PX32-Variance	AX29-Skewness			VX96- Size Zone Non-Uniformity Normalized

### WHO/ISUP grade prediction model construction and performance comparison

**Figure 3:**
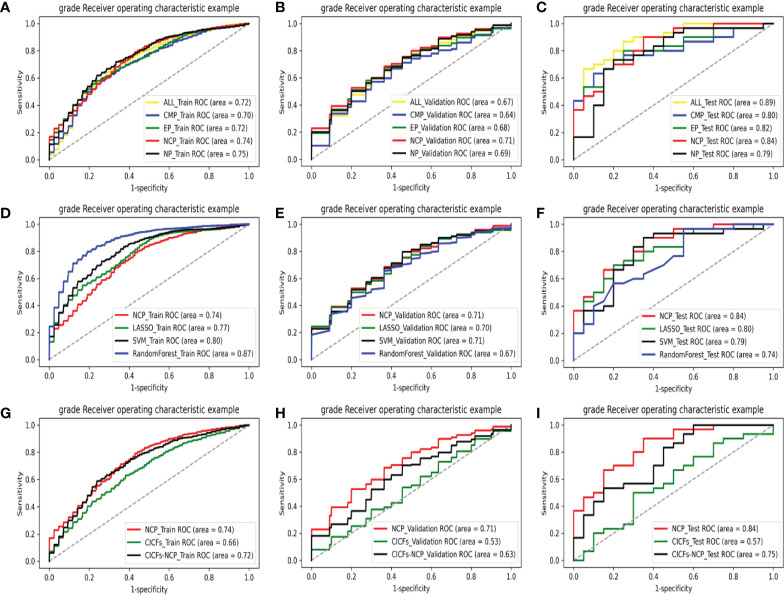
The ROC curves of various models on all data sets: comparing with different phases models in the training set **(A)**, validation set **(B)**, and test set **(C)**; comparing with the machine learning control models in the training set **(D)**, validation set **(E)**, and test set **(F)**; comparing with the clinic control models in the training set **(G)**, validation set **(H)**, and test set **(I)**.

The ROC analysis of different phase models in the training set, validation set, and test set are shown in **Figures 3A–C**, respectively. DeLong test was used to compare the AUCs of the different models. The best two models were the NP and the NCP models in the training set (AUC = 0.75 and 0.74, respectively). They were significantly better than the rest models (*p* < 0.05), and there was no subtle difference between them (*p* = 0.087). The NCP model was still the best in the validation set (AUC = 0.71), significantly better than the other models (all *p* < 0.05). Finally, the NCP model (AUC = 0.84) remained one of the best two models in the test set; the other one was the ALL-P model (AUC = 0.89). Their performance was significantly better than the rest models (all *p* < 0.05), and no significant difference (*p* = 0.062) was found between them. Taking all the results together, the NCP model performed robustly and showed a good capability of predicting WHO/ISUP grade.

This result indicated that the NCP features selected by our method contain more helpful information than the enhanced scan features in terms of ccRCC grading prediction. This could provide a new basis for reducing the use of contrast media and reducing patients’ radiation in the future. The model formula constructed in the training set (125 cases) is NCP_Y = 0.56×PX107+0.40×PX77-0.35×PX59+ 0.11×PX74 +0.25×PX2+0.84×PX11+0.11×PX90-0.33×PX32 +0.11×PX33-0.37×PX38. The performance of the NCP model is shown in **Table 3**.

**Table 3:** The performance of the NCP model of predicting WHO/ISUP grading in the training set, validation set, and test set.

NCP - Model	AUC	Precision	Sensitivity	Accuracy
Training set	0.74	0.71	0.66	0.67
Validation set	0.71	0.70	0.64	0.66
Test set	0.89	0.79	0.76	0.76

After that, the NCP model was compared with the machine learning control models (LASSO, SVM, and the Random Forest model). The ROC analysis of the above models in the training set, validation set, and test set are shown in **Figures 3D–F**, respectively. The control models performed significantly better (all *p* < 0.05) than the NCP model (AUC = 0.74) in the training set. However, their performance sharply deteriorated in the validation set, which made the NCP model become the best (AUC = 0.71), and no significant difference was found among them (all *p >*0.05). What’s more, the NCP model (AUC = 0.84) significantly outperformed other models (all *p <*0.05) in the test set. Compared with the traditional FS methods, experimental results show that our FS method was more effective. Moreover, unlike the other methods, i.e., the validation and test set performance sharply deteriorated from the training set, our approach performed stably in all data sets with good prediction capability and outstanding robustness.

Finally, the NCP model was compared with the clinic control models (CICFs and CICFs-NCP model), The ROC analysis of the above models in the training set, validation set, and test set are shown in **Figures 3G–I**, respectively. The performance of the NCP-model (AUC = 0.74, 0.71, and 0.84) was significantly better than the CICFs (*p* < 0.001) and the CICFs-NCP model (*p* < 0.001) in all data sets.

### Kaplan-Meier survival analysis

**Figure 4:**
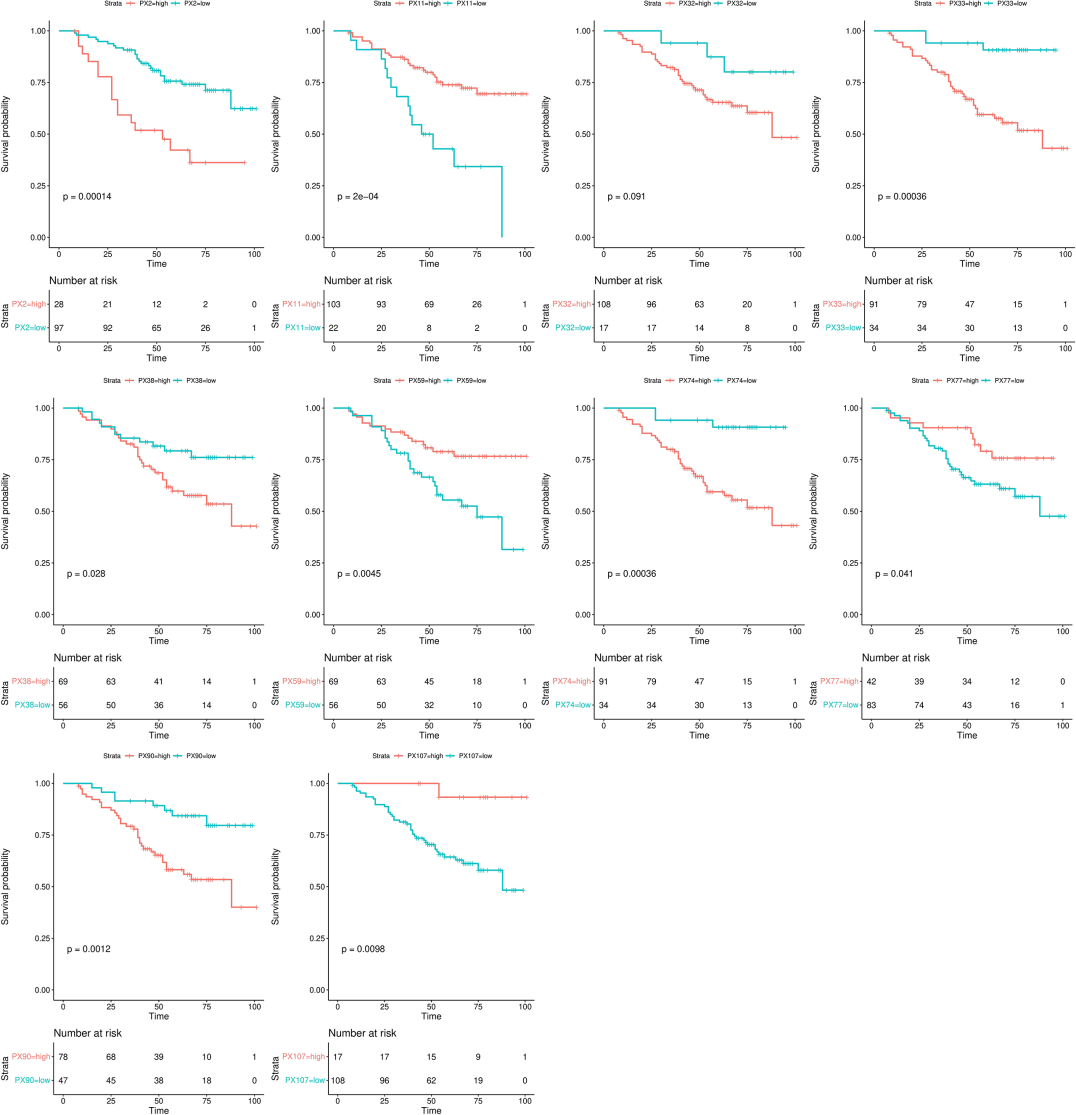
Kaplan-Meier analysis of the RFs of the NCP model.

The Kaplan-Meier survival analysis results of the RFs of the NCP model in the training set are shown in **Figure 4**. All RFs were significant differences between their high and low groups (all *p* < 0.05), except PX32 (*p* = 0.091). The correlation circle diagram of the RFs of the NCP model is shown in **Figure 2D**.

### Univariate and multivariate Cox regression analyses

The univariate and multivariate Cox regression analyses results of the relations of independent variables of the RFs to PFS in the training set are shown in **Table 4**. Univariate results showed that PX2, PX11, PX38, and PX107 were significant (*p* < 0.05), and were entered into the multivariate model. Similarly, RFs were substantial in the multivariate model: PX2 and PX11 (*p* < 0.05) were established as the final NCP model for PFS.

**Table 4:** The univariate and multivariate Cox regression analysis of the independent RFS of the NCP model to PFS in the training set.

RFS	Levels	HR (univariable)	HR (multivariable)
PX2	0.7 ± 0.1	299.26 (6.08-14724.37, *p* = .004)	3828.42 (39.13.00-37458.02, *p* <.001)
PX11	0.7 ± 0.0	0.00 (0.00-0.06, *p* = .006)	0.00 (0.00-0.00, *p* <.001)
PX32	41.0 ± 160.1	1.00 (1.00-1.00, *p* = .687)	
PX33	287.0 ± 618.4	1.00 (1.00-1.00, *p* = .598)	
PX38	0.3 ± 0.1	19.94.00 (1.02-389.01, *p* =. 046)	13.32 (0.65-272.58, *p* = .093)
PX59	0.1 ± 0.0	0.00 (0.00-5764.93, *p* = .097)	
PX74	40.2 ± 158.8	1.00 (1.00-1.00, *p* = .687)	
PX77	0.7 ± 0.7	0.68 (0.36-1.28, *p* =. 228)	
PX90	38.6 ± 156.5	1.00 (1.00-1.00, *p* = .695)	
PX107	0.0 ± 0.2	0.00 (1.00-0.18, *p* = .027)	0.00 (0.00-1202.86, *p* = .402)

### Risk score analyses

**Figure 5:**
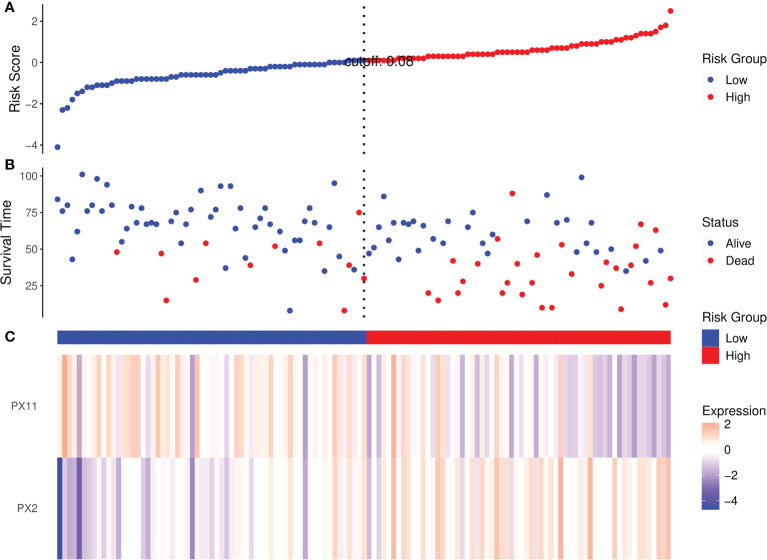
Risk score analysis of RCC patients in the training set based on the RFs of the NCP model. **(A)** Distribution of risk scores per patient; **(B)** Relationships between survival status and survival times of ccRCC patients ranked by risk score; **(C)** Heatmap of the RFS. Colors from blue to red indicate decreasing levels from high to low.

Risk score analyses of ccRCC patients in the training set based on the NCP model are shown in **Figure 5**. The risk scores of the NCP model where the rank of patients was set into the high-low risk group are shown in **Figure 5**.

Risk scores ranked the relationships between survival status and survival times of RCC patients are shown in **Figure 5B**. In addition, the heatmap of the two RFs of the final NCP model is shown in **Figure 5C**. The PX2 was a risk factor as its expression distribution was like to the risk scores; conversely, PX11 was a protective factor. Thus, these two NCP RFs could accurately predict patient prognosis and potentially impact the occurrence and development of tumors.

### Nomogram

**Figure 6:**
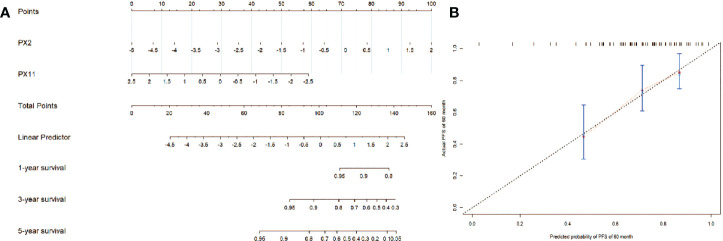
**(A)** The nomogram of the final NCP model; **(B)** The calibration curve of the nomogram of the final NCP model.

The nomogram of the final NCP model for clinical visualization was established in **Figure 6A**. The final NCP model for PFS was established using the PX2 and PX11, including risk estimations of PFS and 1-, 3-, and 5-year survival. It was found that the C-index of the final NCP model was 0.71 (*p* = 0.038) and 0.69 (*p* = 0.066) in 3the training set and test set. The calibration curve of the nomogram of 60 months is shown in **Figure 6B**, indicating that the final model fits the real predicted value.

## Discussion

In this retrospective analysis, we developed a new RFs FS method based on differential network analysis using MEPM. According to the Radiomics Quality Score (RQS) (27), this paper received 22 points for self-evaluation, with a total score of 36 points, indicating the high quality of this paper. Five WHO/ISUP grade prediction models with different phase RFs were constructed based on this method. According to their performance in all data sets, the NCP model was set as the final WHO/ISUP grade prediction model. This model was very competitive with three classical machine learning models and the clinical models in terms of good prediction capability and outstanding robustness. Survival analysis was performed to further explore the biology of the selected RFs. The results showed that almost all selected RFs could effectively distinguish PFS. In the meantime, PX2 (NCP-Flatness) was a risk factor, and PX11 (NCP-Sphericity) was a protective factor for PFS. The results showed that the newly selected NCP RFs were significant for WHO/ISUP classification and survival prediction. Thus, the competitiveness and the interpretability of our new FS method were verified.

The one main difference between this study and prior similar studies is that we first used a new FS method based on differential network analysis using MEPM to select RFs. It focused on the inherent topology of the network of RFs that could reflect the high-low WHO/ISUP grades of RCC very well. It should be noticed that different network inference methods could lead to vastly different results in differential network analysis. The most common association measure was the well-known Pearson correlation coefficient. However, the Pearson correlation could be misleading in reflecting the correlation of two features as it ignores the influence of the rest ones (28). The MEPMs were proposed to solve this problem which relied on Boltzmann’s concept of entropy maximization to support statistical inference with minimal reliance on the form of missing information (21). An example of applications of MEPM to infer gene networks is shown in **Figure 2A**. The elements of the matrix *M_i j_* reflect the pairwise gene interactions between gene *i* and gene *j*. The matrix M can be obtained by inverting the matrix of their covariances C by using Pearson correlation (21). The covariance matrix C reflects the unconditional correlation between features and contains indirect effects. On the other hand, its inverse, i.e., M, describes the correlations that remain once the indirect effects are removed, thereby providing a more robust description of the interactions between genes. For the above reasons, MEPM was used to construct the high-low WHO/ISUP grade networks.

In our study, the NCP model performed better than the other phase model based on our FS method. It is a promising noninvasive rediomics model even without a tri-phase enhancement scan for predicting the grade of ccRCC. It can preoperatively predict the tumor’s aggressiveness and provide a reference for predicting the prognosis. What’s more, it can also provide a reference for selecting surgical plans and follow-up plans and can help guide to make more accurate treatment decisions for ccRCC. Our conclusion is consistent with the study of Kocak et al. (29) who reported that using an artificial neural network is a promising noninvasive method for predicting the grade of ccRCC. Using the traditional machine learning methods, most other research groups suggested that RFs from the CMP or NP, or combined phases, produced more accurate results. For example, Shu et al. (30) found that a combined CMP and NP model provided a diagnostic accuracy. Our method is different from the traditional machine learning method, and it could have advantages in exploring more potential significant information of NCP RFs in predicting WHO/ISUP grade.

The existing machine learning FS methods can generally be classified into two categories: 1) filter-based and 2) wrapper-based methods. Filter-based FS methods use feature relevance criteria, such as mutual information or the Pearson correlation coefficient, to select the feature subset. Wrapper-based methods utilize a classification algorithm to estimate the importance of the selected features. Although the filter-based methods are computationally less expensive than the wrapper ones, they ignore the performance of the selected features on the prediction performancethus, the selected features by the filter methods are often worse than those achieved by the wrapper-based FS methods (31). Therefore, this study mainly focuses on comparing wrapper-based FS methods. However, it should be noted that it does not mean the filter-based FS method does not work for the radiomics study. Parmar C et al. (32) mRMR showed the highest prognostic performance in head and neck cancer. Stefano Barone et al. (33) showed promising results on prostate cancer radiomics.

In the meantime, unlike the other FS methods, i.e., the validation and test set performance sharply deteriorated from the training set, our approach performed stably in all data sets with good prediction capability and outstanding robustness. One reason for that could be our FS method paid more attention to the differences topological of high-low grade network of the RFs, rather than the prediction performance in the training set, avoiding the overfitting of the model. On the other hand, our method used MEPM to construct the GRN, which could remove the variational effect due to the influence of the remaining RFs to ensure the selected RFs were more robust with good generalization performance. Finally, the differential network analyses have proved to have strong and stable performance in finding biomarkers in bioinformatics studies nowadays when the dataset is small and unbalanced (34), and the results in our study confirm these advantages.

Another contribution of this paper is that we first explore the relationship between the RFs of the WHO/ISUP grade prediction model and the PFS of the ccRCC. According to survival analysis, the biological association of the selected RFs with the PFS of ccRCC was proved. The results showed that almost all the selected NCP RFs of the WHO/ISUP grade prediction model could effectively distinguish PFS. This could suggest that the RFs may be related to some intrinsic biologic behavior. Most previous studies had either explored the grading or survival prediction abilities of RFs alone rather than combining them. Feng et al. (35) reported that entropy was the most critical imaging marker for predicting the Fuhrman grade of ccRCC. Bektas et al. (36) reported that the SVM method provided the best model for predicting Fuhrman low-grade or high-grade ccRCCs using ML-based portal-phase contrast-enhanced CT texture data. In another study, Shu et al. (37) reported that a model combined k-nearest neighbor, logistic regression, multilayer perceptron, random forest, and SVM methods exhibited better performance than a CMP or NP model. Beyond an accurate classification, learning an interpretable model with features biologically relevant to the target could be more meaningful in understanding the mechanism of a radiomics model. This study further explored the underlying molecular basis of the identified RFs of the WHO/ISUP grade prediction model by assessing the possible biological association with the PFS. The results showed that our differential network FS method is applicable.

In this experiment, the image segmentation method is manual, unlike the other semi-automatic or automatic segmentation methods, which are most widely used in the lung and brain. However, the existing general semi-automatic segmentation method seems to have low accuracy in renal tumors. For example, separating tumors from normal tissues during the NCP is impossible because most tumors are of equal or slightly low density. In the EP, due to the highly enhanced tumor, which may infiltrate the renal pelvis and encroach renal veins, it is difficult to exclude these non-tumor normal tissues using semi-automatic segmentation of regional growth. Though it could cause problems with repetition and consistency (38, 39), manual segmentation is still used in most renal radiomics studies (40, 41). In response to the lack of automated segmentation, the Medical Imaging Computing and Computer Assisted Intervention (MICCAI) society developed the KiTS19 (Kidney Tumor Segmentation) Grand Challenge, where scientists compete using algorithms to automate the segmentation of kidney tumors. Although the effect is good in the arterial phase, there is still a lack of studies on the effectiveness of the NCP. To make the experiment more rigorous, we selected senior doctors for image segmentation and trained two doctors to standardize the segmentation process. In addition, our experiments aim to propose a new FS method, and we prefer to let the feature selection part mainly decide which RFs should be retained to test the performance of FS methods. Thereby we are relatively relaxed in RFs estimation before the feature selection, choosing ICC >0.75 instead of ICC >0.8 or 0.9 to ensure more features could be involved in the feature selection while avoiding eliminating potentially valuable features to improve the repeatability (30). Fortunately, all features were retained.

There are still some limitations to this study. First, as our work was a single-center and retrospective study, the dataset was relatively small. In the meantime, comparing with the other network construction methods and filter-based FS methods are necessary to verify the effectiveness of the model developed in this study. Furthermore, we know that only using the protective or risk factor for PFS to prove the biological meaning of the RFs is not enough. Therefore, future research will study the relationship between the RFs and the genomic or pathology information in the RCC pattern (42).

## Data availability statement

The original contributions presented in the study are included in the article/**Supplementary Material**. Further inquiries can be directed to the corresponding authors.

## Ethics statement

The studies involving human participants were reviewed and approved by Ethics Committee of Southern Medical University. Written informed consent for participation was not required for this study in accordance with the national legislation and the institutional requirements.

## Author contributions

FY: Conceptualization, Writing - Original Draft, Software, Validation. HZ: Conceptualization, Writing - Original Draft, Methodology, Statistics. AQ: Investigation, Formal analysis. ZZ: Investigation, Formal analysis. LY:Investigation. WX: Conceptualization. GW: Conceptualization, Writing - Review and Editing. All authors contributed to the article and approved the submitted version.

## Conflict of interest

The authors declare that the research was conducted in the absence of any commercial or financial relationships that could be construed as a potential conflict of interest.

## Publisher’s note

All claims expressed in this article are solely those of the authors and do not necessarily represent those of their affiliated organizations, or those of the publisher, the editors and the reviewers. Any product that may be evaluated in this article, or claim that may be made by its manufacturer, is not guaranteed or endorsed by the publisher.

## Supplementary material

Supplementary Figure 1 PCA results, PC 1: First Principal Component, PC 2: Second Principal Component.

## References

[1] Delahunt B Mckenney JK Lohse CM Leibovich BC Thompson RH Boorjian SA. A novel grading system for clear cell renal cell carcinoma incorporating tumor necrosis. Am J Surg Pathol (2013) 37(3):311–22. doi: 10.1097/PAS.0b013e318270f71c 23348209

[2] Kuthi L Jenei A Hajdu A Németh I Varga Z Bajory Z. Prognostic factors for renal cell carcinoma subtypes diagnosed according to the 2016 WHO renal tumor classification: a study involving 928 patients. Pathol Oncol Res (2017) 23(3):689–98. doi: 10.1007/s12253-016-0179-x 28032311

[3] Mouracade P Kara O Maurice MJ Dagenais J Malkoc E Nelson RJ. Patterns and predictors of recurrence after partial nephrectomy for kidney tumors. J Urol (2017) 197(6):1403–9. doi: 10.1016/j.juro.2016.12.046 27993666

[4] Perrino CM Cramer HM Chen S Idrees MT Wu HH. World health organization (WHO)/International society of urological pathology (ISUP) grading in fine-needle aspiration biopsies of renal masses. Diagn Cytopathol (2018) 46(11):895–900. doi: 10.1002/dc.23979 30488673

[5] Dagher J Delahunt B Rioux-Leclercq N Egevad L Srigley JR Coughlin G. Clear cell renal cell carcinoma: validation of world health Organization/International society of urological pathology grading. Histopathology (2017) 71(6):918–25. doi: 10.1111/his.13311 28718911

[6] Robila V Kraft AO Smith SC. New entities, new technologies, new findings: A review of the cytologic features of recently established subtypes of renal cell carcinoma. Cancer Cytopathol (2019) 127(2):79–97. doi: 10.1002/cncy.22093 30690877

[7] Siegel RL Miller KD Jemal A. Cancer statistics 2018. CA Cancer J Clin (2018) 68(1):7–30. doi: 10.3322/caac.21442 29313949

[8] Chen W Zheng R Baade PD Zhang S Zeng H Bray F. Cancer statistics in China 2015. CA Cancer J Clin (2016) 66(2):115–32. doi: 10.3322/caac.21338 26808342

[9] Yan Y Liu L Zhou J Li L Li Y Chen M. Clinicopathologic characteristics and prognostic factors of sarcomatoid renal cell carcinoma. J Cancer Res Clin Oncol (2015) 141(2):345–52. doi: 10.1007/s00432-014-1740-1 PMC11824019 25178995

[10] Kutikov A Smaldone MC Uzzo RG Haifler M Bratslavsky G Leibovich BC. Renal mass biopsy: Always, sometimes, or never? Eur Urol (2016) 70(3):403–6. doi: 10.1016/j.eururo.2016.04.001 27085625

[11] Millet I Curros F Serre I Taourel P Thuret R. Can renal biopsy accurately predict histological subtype and fuhrman grade of renal cell carcinoma? J Urol (2012) 188(5):1690–4. doi: 10.1016/j.juro.2012.07.038 22998910

[12] Blumenfeld AJ Guru K Fuchs GJ Kim HL. Percutaneous biopsy of renal cell carcinoma underestimates nuclear grade. Urology (2010) 76(3):610–3. doi: 10.1016/j.urology.2009.09.095 20163843

[13] Ficarra V Brunelli M Novara G D'elia C Segala D Gardiman M. Accuracy of on-bench biopsies in the evaluation of the histological subtype, grade, and necrosis of renal tumours. Pathology (2011) 43(2):149–55. doi: 10.1097/PAT.0b013e32834317a4 21233677

[14] Jeldres C Sun M Liberman D Lughezzani G De La Taille A Tostain J. Can renal mass biopsy assessment of tumor grade be safely substituted for by a predictive model? J Urol (2009) 182(6):2585–9. doi: 10.1016/j.juro.2009.08.053 19836799

[15] Lambin P Rios-Velazquez E Leijenaar R. Radiomics: extracting more information from medical images using advanced feature analysis. Eur J Cancer (2012) 48:441. doi: 10.1016/j.ejca.2011.11.036 22257792 PMC4533986

[16] Pinker K Shitano F Sala E Do RK Young RJ Wibmer AG. Background, current role, and potential applications of radiogenomics. J Magn Reson Imaging (2017) 7:604–20. doi: 10.1002/jmri.25870 PMC5916793 29095543

[17] Zhou H Mao H Dong D Fang M Gu D Liu X. Development and external validation of radiomics approach for nuclear grading in clear cell renal cell carcinoma. Ann Surg Oncol (2020) 27(10):4057–65. doi: 10.1245/s10434-020-08255-6 32424585

[18] Li ZC Zhai G Zhang J Wang Z Liu G Wu GY. Differentiation of clear cell and non-clear cell renal cell carcinomas by all-relevant radiomics features from multiphase CT: a VHL mutation perspective. Eur Radiol (2019) 29(8):3996–4007. doi: 10.1007/s00330-018-5872-6 30523454

[19] Al-Kasassbeh M Mohammed S Alauthman M Almomani A. Feature selection using a machine learning to classify a malware. In: Gupta BB Perez GM Agrawal DP Gupta D, editors. Handbook of computer networks and cyber security: Principles and paradigms. Cham: Springer International Publishing (2020). p. 889–904.

[20] Grimes T Potter SS Datta S. Integrating gene regulatory pathways into differential network analysis of gene expression data. Sci Rep (2019) 9(1):5479. doi: 10.1038/s41598-019-41918-3 30940863 PMC6445151

[21] De Martino A De Martino D. An introduction to the maximum entropy approach and its application to inference problems in biology. Heliyon (2018) 4(4):e00596. doi: 10.1016/j.heliyon.2018.e00596 29862358 PMC5968179

[22] Kocak B Ates E Durmaz ES Ulusan MB Kilickesmez O. Influence of segmentation margin on machine learning-based high-dimensional quantitative CT texture analysis: a reproducibility study on renal clear cell carcinomas. Eur Radiol (2019) 29(9):4765–75. doi: 10.1007/s00330-019-6003-8 30747300

[23] Tian Y Lu C Zhang X Cheng F Jin Y. A pattern mining-based evolutionary algorithm for Large-scale sparse multiobjective optimization problems. IEEE Trans Cybern (2021) 52(7):6784–97. doi: 10.1109/TCYB.2020.3041325 33378271

[24] Yin F Zhou J Zhu Z Ma X Xie W. Inferring small-scale maximum-entropy genetic regulatory networks by using DE algorithm. Springer International Publishing Cham (2021) 347–57.

[25] Friedman J Hastie T Tibshirani R. Sparse inverse covariance estimation with the graphical lasso. Biostatistics (2008) 9(3):432–41. doi: 10.1093/biostatistics/kxm045 PMC3019769 18079126

[26] Pasini G Bini F Russo G Comelli A Marinozzi F Stefano A. matRadiomics: A novel and complete radiomics framework, from image visualization to predictive model. J Imaging (2022) 8(8):221. doi: 10.3390/jimaging8080221 36005464 PMC9410206

[27] Lambin P Leijenaar RTH Deist TM Peerlings J De Jong EEC Van Timmeren J. Radiomics: the bridge between medical imaging and personalized medicine. Nat Rev Clin Oncol (2017) 14(12):749–62. doi: 10.1038/nrclinonc.2017.141 28975929

[28] Chanda P Costa E Hu J Sukumar S Van Hemert J Walia R. Information theory in computational biology: Where we stand today. Entropy (Basel) (2020) 22(6):627. doi: 10.3390/e22060627 PMC7517167 33286399

[29] Kocak B Durmaz ES Ates E Kaya OK Kilickesmez O. Unenhanced CT texture analysis of clear cell renal cell carcinomas: A machine learning-based study for predicting histopathologic nuclear grade. AJR Am J Roentgenol (2019) 11:W1–8. doi: 10.2214/AJR.18.20742 30973779

[30] Shu J Tang Y Cui J Yang R Meng X Cai Z. Clear cell renal cell carcinoma: CT-based radiomics features for the prediction of fuhrman grade. Eur J Radiol (2018) 109:8–12. doi: 10.1016/j.ejrad.2018.10.005 30527316

[31] Chandrashekar G Sahin F. A survey on feature selection methods. Comput Electrical Eng (2014) 40(1):16–28. doi: 10.1016/j.compeleceng.2013.11.024

[32] Parmar C Grossmann P Rietveld D Rietbergen MM Aerts H. Radiomic machine learning classifiers for prognostic biomarkers of head & neck cancer. Front Oncol (2015) 5(4). doi: 10.3389/fonc.2015.00272 PMC4668290 26697407

[33] Barone S Cannella R Comelli A Pellegrino A Salvaggio G Stefano A. Hybrid descriptive-inferential method for key feature selection in prostate cancer radiomics. Appl Stochastic Models Business Industry (2021) 37(5):961–72. doi: 10.1002/asmb.2642

[34] Wu N Huang J Zhang XF Ou-Yang L He S Zhu Z. Weighted fused pathway graphical lasso for joint estimation of multiple gene networks. Front Genet (2019) 10:623. doi: 10.3389/fgene.2019.00623 31396259 PMC6662592

[35] Feng Z Shen Q Li Y Hu Z. CT texture analysis: a potential tool for predicting the fuhrman grade of clear-cell renal carcinoma. Cancer Imaging (2019) 19(1):6. doi: 10.1186/s40644-019-0195-7 30728073 PMC6364463

[36] Bektas CT Kocak B Yardimci AH Turkcanoglu MH Yucetas U Koca SB. Clear cell renal cell carcinoma: Machine learning-based quantitative computed tomography texture analysis for prediction of fuhrman nuclear grade. Eur Radiol (2019) 29(3):1153–63. doi: 10.1007/s00330-018-5698-2 30167812

[37] Shu J Wen D Xi Y Xia Y Cai Z Xu W. Clear cell renal cell carcinoma: Machine learning-based computed tomography radiomics analysis for the prediction of WHO/ISUP grade. Eur J Radiol (2019) 121:108738. doi: 10.1016/j.ejrad.2019.108738 31756634

[38] Timmeren J Cester D Tanadini-Lang S Alkadhi H Baessler B. Radiomics in medical imaging—"how-to" guide and critical reflection. Insights into Imaging (2020) 11(1):91–107. doi: 10.1186/s13244-020-00887-2 PMC7423816 32785796

[39] Sharma N Aggarwal LM Automated medical image segmentation techniques. Journal of Medical Physics (2010) 35(1):3–14. doi: 10.4103/0971-6203.58777 PMC2825001 20177565

[40] Suarez-Ibarrola R Basulto-Martinez M Heinze A Gratzke C Miernik A Radiomics Applications in Renal Tumor Assessment: A Comprehensive Review of the Literature. Cancers (Basel) (2020) 12(6):1387. doi: 10.3390/cancers12061387 PMC7352711 32481542

[41] Bhandari A Ibrahim M Sharma C Liong R Gustafson S Prior M.CT-based radiomics for differentiating renal tumours: a systematic review. Abdom Radiol (2020) 46(5):2052–63. doi: 10.1007/s00261-020-02832-9 33136182

[42] Algohary A Shiradkar R Pahwa S Purysko A Verma S Moses D. Combination of peri-tumoral and intra-tumoral radiomic features on bi-parametric MRI accurately stratifies prostate cancer risk: A multi-site study. Cancers (Basel) (2020) 12(8):2200. doi: 10.3390/cancers12082200 PMC7465024 32781640

