# Supplementary figures and images for: An exploratory study of CT radiomics using differential network feature selection for WHO/ISUP grading and progression-free survival prediction of clear cell renal cell carcinoma
Source: Front Oncol. 2022 Oct 27;12:979613. doi: 10.3389/fonc.2022.979613 (PMC9648858; doi:10.3389/fonc.2022.979613)

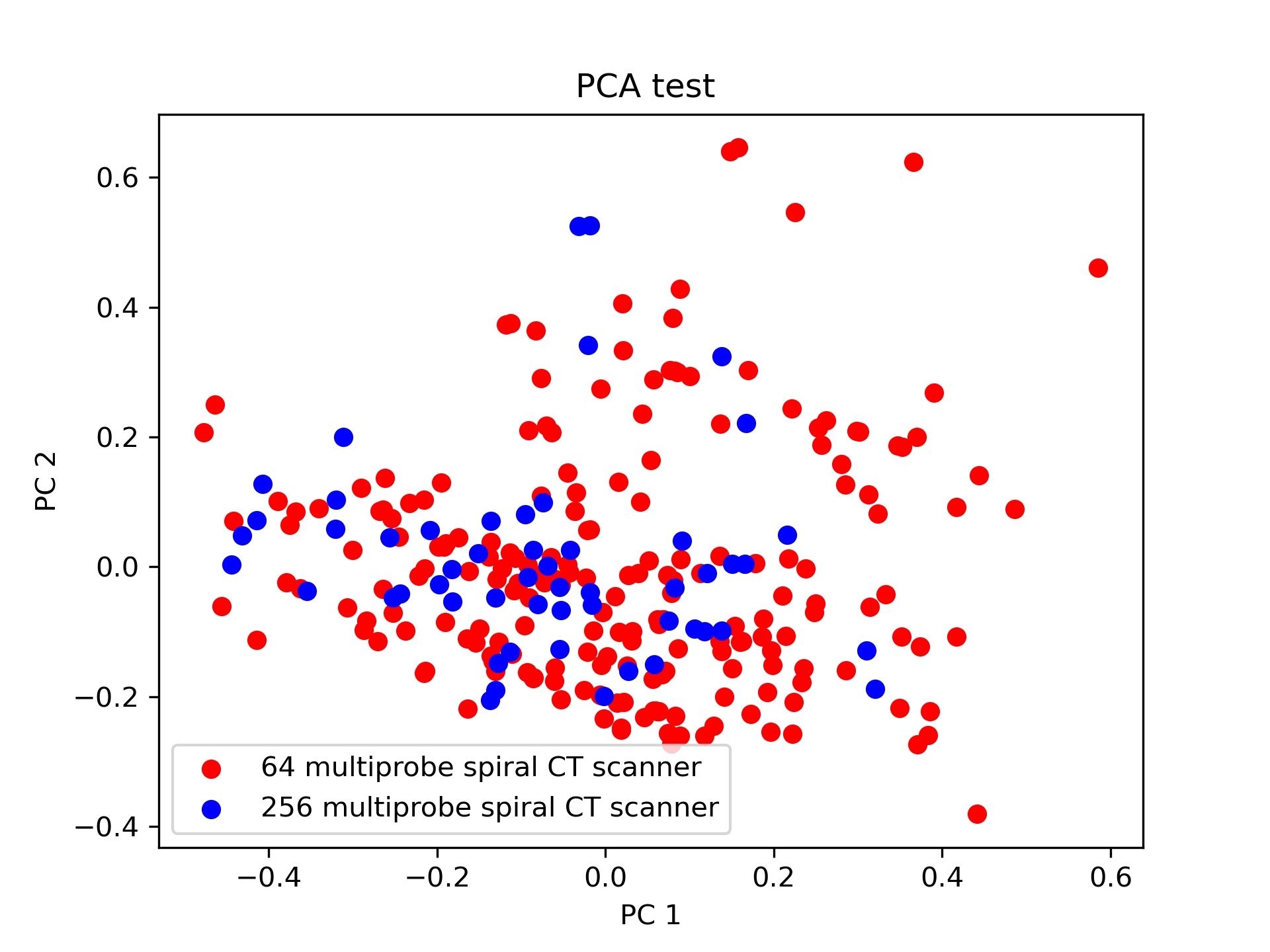

Supplement: Supplementary Figure 1 — PCA results, PC 1: First Principal Component, PC 2: Second Principal Component. [file Image_1.jpeg]
