# Supplementary material for: An exploratory study of CT radiomics using differential network feature selection for WHO/ISUP grading and progression-free survival prediction of clear cell renal cell carcinoma
Source: Front Oncol. 2022 Oct 27;12:979613. doi: 10.3389/fonc.2022.979613 (PMC9648858; doi:10.3389/fonc.2022.979613)
Supplement: Supplementary file 2 [file Table_1.docx]

Supplement table 1 Abbreviation list

| **Abbreviation** | **Full name** |
| --- | --- |
| WHO/ISUP | World Health Organization/International Society of Urological Pathology |
| PFS | Progression-free survival |
| ccRCC | Clear cell renal cell cancer |
| RFs | Radiomics features |
| MEPM | Maximum-entropy probability model |
| FS | feature selection |
| DT | Decision tree |
| SVM | Support vector machine |
| CNN | Convolution neural network |
| NCP | No-contrast phase |
| CMP | Cortico-medullary phase |
| NP | Nephrographic phase |
| EP | Excretory phase |
| FOV | Field of view |
| VROI | Volume region of interest |
| ICC | Intraclass correlation coefficient correlation coefficients |
| GRN | Gene regulatory network |
| CICFs | Conventional image and clinical features |
| ROC | Receiver operating characteristic |
| AUC | Area under curve ROC curve |
| GLCM | Gray level co-occurrence matrix |
| GLSZM | Gray level size zone matrix |
| GLRLM | Gray level run length matrix |
| GLDM | Gray level dependence matrix |
| NGTDM | Neighboring gray-tone difference matrix |
