# Supplementary material for: An exploratory study of CT radiomics using differential network feature selection for WHO/ISUP grading and progression-free survival prediction of clear cell renal cell carcinoma
Source: Front Oncol. 2022 Oct 27;12:979613. doi: 10.3389/fonc.2022.979613 (PMC9648858; doi:10.3389/fonc.2022.979613)
Supplement: Supplementary file 3 [file DataSheet_1.pdf]

id	WHO	grade	age	sex	symptom	side	grow	diameter	cystic	calcificatic	T	N	M	TNM	NCP
1	2	1	47	1	1	1	1	36.09	1	0	1	1	0	1	26.32
2	2	1	48	1	1	1	1	39.93	1	0	1	1	0	2	14.52
3	3	2	25	2	1	2	1	46.31	1	0	1	1	0	1	48.595
4	3	2	44	1	1	2	1	11.91	0	0	1	0	0	1	33.535
5	3	2	69	1	0	2	0	26.01	1	0	1	1	0	3	36.16
6	2	1	80	1	1	1	0	50.01	1	0	1	1	0	3	24.85
7	3	2	43	1	0	2	1	31.29	1	0	1	0	0	1	17.85
8	3	2	56	1	0	1	2	44.65	1	0	1	0	0	1	27.52
9	3	2	53	1	0	2	0	22.61	1	0	1	0	0	1	28.99
10	2	1	49	1	0	1	0	55.44	1	0	1	0	0	1	37.175
11	2	1	73	1	1	2	2	107.46	1	0	3	1	0	3	39.63
12	4	2	16	1	1	2	2	130.54	1	1	3	0	0	3	51.865
13	3	2	36	1	1	1	0	53.66	1	0	1	0	0	1	25.535
14	1	1	53	2	1	2	1	27.63	1	0	4	0	0	4	23.305
15	2	1	28	1	0	1	1	18.02	1	0	1	0	0	1	33.085
16	3	2	45	1	0	1	2	87.41	1	1	2	0	0	2	37.67
17	2	1	50	2	0	2	1	22.05	1	0	1	0	0	1	17.745
18	2	1	73	2	0	1	0	55.84	1	0	3	0	0	3	32.465
19			57	1	1	2	2	50.77	1	0	4	1	0	4	38.21
20	4	2	60	2	1	1	2	91.82	1	0	2	0	0	2	37.505
21	1	1	60	1	1	2	1	95.55	1	0	4	0	0	4	37.645
22	1	1	51	1	1	1	0	70.27	1	0	4	0	0	4	33.85
23	2	1	70	1	0	2	0	28.69	1	0	1	0	0	2	33.265
24	2	1	59	2	1	1	2	90.5	1	0	2	0	0	2	37.025
25	2	1	68	2	0	2	0	57.81	1	0	1	0	0	1	45.17
26	1	1	67	1	1	1	0	27.78	1	1	1	0	0	1	34.215
27	2	1	13	2	1	1	1	40.96	1	1	1	0	0	1	40.655
28	1	1	45	2	1	1	1	45.07	1	0	3	0	0	3	36.935
29	2	1	62	1	0	2	0	44.02	1	0	2	0	0	2	38.315
30	2	1	82	1	0	1	1	44.38	1	0	1	0	0	1	39.24
31	2	1	41	1	0	1	0	35.56	0	0	1	0	0	1	30.04
32			57	1	1	1	2	65.17	1	0	4	1	1	4	36.46
33	1	1	56	1	1	2	1	36.03	1	0	1	0	0	1	27.87
34	4	2	62	2	1	1	2	57.47	1	1	4	1	0	4	30.89
35			50	1	1	1	0	138.47	1	1	4	1	1	4	39.315
36	2	1	60	1	1	2	1	36.88	0	0	1	0	0	1	30.885
37			38	2	1	1	2	108.02	1	1	2	1	1	4	37.29
38	1	1	63	1	0	1	1	29.2	1	0	1	0	0	1	14.61
39	3	2	53	1	1	1	1	38.79	1	0	1	0	0	1	38.61
40	2	1	58	2	1	1	0	28.31	1	0	1	0	0	1	23.385
41	1	1	74	1	0	1	0	49.3	1	0	4	0	0	4	34.765
42	2	1	54	2	1	1	0	105.35	1	0	4	0	0	4	46.95
43	2	1	50	1	1	2	0	34.76	1	0	1	0	0	1	31.54
44	3	2	58	2	1	2	2	70.07	1	1	2	0	0	2	31.01
45	3	2	66	2	1	2	2	98.1	1	0	4	0	0	4	43.585
46	2	1	52	1	1	2	2	28.28	0	0	1	0	0	1	26.56
47			48	2	0	1	0	32.51	1	0	4	0	0	4	33.42
48			55	2	1	1	1	61.82	1	0	1	0	1	4	37.435
49	2	1	36	1	1	2	1	51.83	1	0	1	0	0	1	33.79
50	3	2	75	2	1	2	0	68.54	1	0	2	0	0	2	36.465
51	3	2	60	1	1	1	0	77.04	1	0	2	0	0	2	46.13
52	2	1	60	1	0	2	2	26.09	1	0	4	0	0	4	29.775
53	3	2	57	1	1	1	0	35.46	1	0	1	0	0	1	27.85
54	2	1	70	2	1	1	0	37.98	1	0	3	0	0	3	29.905
55	3	2	51	2	1	1	2	62.53	1	1	1	0	0	1	42.88
56	2	1	52	2	1	1	1	54.12	1	0	4	0	0	4	33.405
57	2	1	64	2	0	1	2	42.91	1	0	1	0	0	1	29.115

58	2	1	56	1	0	1	2	34.83	0	0	1	0	0	1	24.355
59	2	1	39	1	0	1	0	29.67	1	0	1	0	0	1	34.78
60	2	1	46	2	0	2	2	35.64	1	1	1	0	0	1	46.18
61	4	2	17	2	1	2	1	143.14	1	1	2	0	0	2	83.29
62	2	1	50	1	0	2	0	28.69	1	0	1	0	0	1	27.465
63	4	2	54	1	1	1	2	66.35	1	0	1	0	0	1	36.575
64	4	2	51	1	1	1	2	76.63	1	1	3	1	0	3	37.3
65	2	1	49	1	0	2	2	97.77	1	1	3	0	0	3	46.595
66	3	2	57	1	1	1	1	27.55	1	0	1	0	0	1	34.835
67	2	1	49	1	0	2	0	28.48	1	0	1	0	0	1	31.1
68	2	1	81	1	0	1	1	31.31	1	0	1	0	0	1	34.63
69	2	1	40	1	0	1	0	80.7	1	0	2	0	0	2	34.34
70	2	1	50	1	1	2	1	135.14	1	1	2	0	0	2	30.35
71	2	1	53	1	1	1	2	87.49	1	0	2	0	0	2	42.76
72	3	2	62	2	1	1	1	124.57	1	0	2	0	0	2	41.105
73	3	2	54	1	1	1	1	103.84	1	1	2	1	0	3	45.455
74	2	1	75	1	1	2	0	18.75	0	0	1	0	0	1	40.975
75	2	1	67	1	0	1	2	57.38	1	0	1	0	0	1	26.805
76	4	2	61	2	1	2	1	85.68	1	0	3	1	0	3	36.97
77	3	2	52	1	0	2	1	43.91	1	0	1	0	0	1	28.835
78	1	1	38	1	0	2	1	72.78	1	1	2	0	0	2	31.16
79	1	1	59	1	0	1	2	22.35	1	0	1	0	0	1	28.045
80	1	1	40	1	0	2	1	41.84	1	0	1	0	0	1	24.09
81	1	1	63	2	1	1	2	65.9	1	1	1	0	0	1	31.615
82	2	1	53	2	0	2	0	31.63	0	1	1	0	0	1	38.8
83	2	1	48	1	1	2	2	79.53	1	0	3	0	0	3	38.94
84	2	1	52	1	1	1	2	134.99	1	0	4	1	1	4	40.895
85	2	1	56	1	0	2	0	64.13	1	0	1	0	0	1	31.645
86	2	1	44	2	1	1	2	53.03	0	0	1	0	0	1	33.78
87	2	1	51	1	1	1	0	53.17	1	0	1	0	0	1	21.81
88	2	1	26	1	0	1	0	23.28	1	0	1	0	0	1	24.59
89	3	2	57	1	0	2	0	62.37	1	0	4	0	0	4	32.785
90	1	1	49	2	1	1	2	64.78	1	0	2	0	0	2	22.235
91	3	2	48	1	1	2	1	51.34	1	1	1	0	1	4	42.335
92	2	1	60	2	1	2	0	43.78	1	0	1	0	0	1	33.79
93	1	1	33	2	1	1	0	53.95	1	0	1	0	0	1	25.995
94	2	1	49	1	0	1	1	26.45	1	1	1	0	0	1	33.375
95	2	1	76	1	1	2	1	64.68	1	1	1	0	0	1	38.265
96	2	1	47	1	0	1	1	19.58	0	0	1	0	0	1	35.685
97	2	1	53	1	0	2	1	19.98	1	0	1	0	0	1	10.98
98			37	1	1	1	1	36.46	1	0	1	0	0	1	25.38
99	2	1	40	1	1	1	1	47.28	1	0	1	0	0	1	33.025
100	2	1	42	1	1	1	1	24.44	1	0	1	0	0	1	29.575
101	3	2	68	2	0	1	2	55.78	1	0	1	0	0	1	41.46
102	3	2	52	1	1	1	1	56.35	1	0	1	0	1	4	32.245
103	2	2	42	2	0	2	0	29.54	1	0	1	0	0	1	11.31
104	1	1	58	1	0	1	2	41.88	1	1	1	0	0	1	30.25
105	4	2	46	2	1	1	0	44.03	1	0	1	0	0	1	44.92
106	2	1	53	2	1	2	1	29.27	0	0	1	0	0	1	69.705
107	2	1	46	1	0	2	2	3.751	1	0	1	0	0	1	53.645
108	1	1	53	1	0	1	2	59.59	1	1	1	0	0	1	39.745
109	2	1	49	1	1	2	2	114.67	1	0	3	0	0	3	33.89
110	2	1	76	1	1	1	2	90.77	1	0	2	0	0	2	22.43
111	1	1	39	1	1	2	1	34.35	1	0	1	1	0	3	34.115
112	1	1	50	2	1	1	0	45.18	1	0	1	0	0	1	11.935
113	2	1	73	1	1	2	2	111.45	1	0	4	0	1	4	29.205
114	1	1	37	1	0	1	0	37.51	1	0	2	0	0	2	38.625
115	1	1	53	2	1	1	2	50.17	1	0	1	0	0	1	31.685

116	1	1	59	1	0	1	0	35.37	1	1	1	0	0	1	29.02
117	2	1	60	1	1	2	1	29.54	1	0	1	0	0	1	28.88
118	3	2	52	1	1	1	1	9.24	1	1	4	0	0	4	32.195
119	2	1	40	1	0	2	1	31.44	0	0	1	0	0	1	36.52
120	2	1	31	1	0	2	1	41.86	1	0	1	0	0	1	34.71
121	3	2	52	2	0	1	1	64.57	1	0	1	0	0	1	36.605
122	2	1	65	1	1	2	1	31.54	1	0	1	0	0	1	33.98
123	4	2	54	1	0	2	0	66.17	1	0	2	1	0	3	40.505
124	1	1	35	2	1	1	1	32.59	1	0	1	0	0	1	27.235
125			54	2	0	1	0	38.25	1	0	1	0	0	1	31.33
126	2	1	67	1	1	1	1	50.18	1	0	1	0	0	1	34.475
127	2	1	62	1	0	2	0	46.98	1	0	1	0	0	1	36.805
128	2	1	48	2	0	1	1	50.17	1	1	1	0	0	1	49.135
129	4	2	63	1	1	2	2	59.68	1	0	1	0	1	4	37.38
130	2	1	68	1	1	1	1	85.67	1	1	2	0	0	2	37.59
131	2	1	72	2	0	2	0	38.55	1	0	1	0	0	1	37.11
132	4	2	57	2	1	2	0	70.18	1	0	2	0	0	2	25.415
133			47	1	1	1	2	13.24	1	1	3	1	1	4	31.64
134	2	1	30	1	0	2	1	27.56	1	0	1	0	1	4	38.215
135	2	1	78	1	0	1	1	37.86	1	0	1	0	0	1	30.04
136	1	1	51	1	0	1	2	25.14	1	0	1	0	0	1	17.575
137	3	2	64	2	1	1	2	45.26	1	0	1	0	0	1	34.83
138	2	1	52	2	0	2	1	30.87	1	0	1	0	0	1	17.975
139	2	1	64	2	0	2	1	33.96	0	0	1	0	0	1	38.68
140	2	1	28	2	0	1	2	87.68	1	0	2	0	0	2	48.855
141	1	1	43	2	0	1	2	43.98	1	0	2	0	0	2	27.72
142	2	1	59	2	0	2	2	38.92	1	0	1	0	0	1	39.45
143	4	2	71	1	0	1	2	73.26	1	0	1	0	0	1	34.7
144	2	1	33	1	0	2	1	25.56	0	0	1	0	0	1	42.28
145	1	1	60	2	1	2	1	35.19	0	1	1	0	0	1	34.55
146	2	1	43	2	0	2	1	22.89	1	0	1	0	0	1	31.58
147	2	1	43	2	1	2	1	32.53	0	0	1	0	0	1	24.9
148	2	1	49	2	0	1	1	67.27	1	0	3	0	0	3	36.76
149	3	2	46	1	0	1	0	76.1	0	0	3	0	0	3	39.73
150	3	2	49	1	0	2	0	25.35	0	0	1	0	0	1	42.31
151	3	2	50	1	1	1	1	21.29	0	0	3	0	0	3	35.35
152	2	1	49	1	0	2	1	35.27	1	0	1	0	0	1	39.2
153	1	1	40	1	1	2	1	55.86	1	0	1	0	0	1	20.76
154	4	2	49	2	1	2	1	69.14	1	0	1	0	0	1	42.13
155	2	1	58	2	1	1	1	45.71	1	0	1	0	0	1	30.01
156	2	1	32	2	0	2	1	48.7	1	0	1	0	0	1	28.22
157	2	1	30	1	0	2	1	26.74	0	0	1	0	0	1	28.52
158	1	1	77	1	1	1	1	53.01	1	0	1	1	0	3	22.22
159	2	1	53	1	0	2	1	17.8	0	0	4	0	0	4	22
160	4	2	53	2	1	2	0	41.31	0	0	1	0	0	1	41.22
161	4	2	69	1	0	1	1	41.44	0	0	1	0	0	1	42.17
162	2	1	50	2	0	1	0	35.61	0	0	1	0	0	1	31.59
163			51	1	1	3	1	69.62	1	1	3	1	1	3	51.73
164	3	2	68	2	0	2	1	40.17	0	0	1	0	0	1	36.01
165	2	1	54	1	0	1	0	69.01	0	1	1	0	0	1	29.3
166	1	1	83	2	0	2	0	43.39	0	0	1	0	0	1	26.8
167	2	1	51	2	0	1	0	31.67	1	1	4	0	0	4	32.1
168	2	1	43	1	0	1	1	12.28	0	0	1	0	0	1	28.56
169	1	1	37	1	0	2	1	47.97	0	0	1	0	0	1	30.91
170	3	2	44	1	1	2	0	57.36	1	0	1	0	0	1	36.77
171	2	1	43	1	0	1	1	30.44	0	0	1	0	0	1	30.19
172	4	2	63	1	1	3	1	94.92	1	0	2	0	0	2	28.14
173	2	1	50	1	1	2	0	34.68	1	0	1	0	0	1	43.73

174	4	2	42	1	1	1	0	56.11	1	1	2	0	0	2	42.31
175	3	2	73	1	1	2	2	41.49	1	0	1	0	0	1	56.11
176	3	2	51	1	0	1	0	37.91	0	0	1	0	0	1	45.02
177	1	1	41	1	1	1	1	29.2	0	0	1	0	0	1	32.33
178	3	2	62	1	0	2	0	60.84	1	0	1	0	0	1	51.04
179	2	1	67	1	0	1	0	41.33	1	0	1	0	0	1	35.37
180	2	1	75	1	1	2	1	58.69	0	0	1	0	1	1	40.36
181	1	1	72	2	0	1	1	42.89	1	0	1	0	0	1	30.88
182	1	1	73	1	1	1	0	36.95	0	0	1	0	0	1	9.61
183	2	1	44	1	1	1	0	178.04	1	1	4	1	0	4	39.42
184	4	2	53	2	0	1	0	51.21	0	0	1	0	0	1	42
185	3	2	52	1	0	2	1	39.87	0	1	1	0	0	1	52.73
186	1	1	50	2	0	2	1	50.28	1	1	1	0	0	1	48.64
187	3	2	27	2	1	1	1	109.13	0	1	1	0	0	2	60.37
188	3	2	62	1	1	2	1	66.92	1	1	1	0	0	1	45.42
189	1	1	79	2	0	1	0	93.62	0	0	3	0	0	3	36.31
190	1	1	40	1	1	2	0	75.1	0	0	1	0	0	1	23.21
191	1	1	34	1	1	2	1	57.8	1	0	1	0	0	1	27.3
192	3	2	54	1	0	1	1	73.63	0	0	1	0	0	1	24.35
193	2	1	47	1	1	1	1	64.8	0	0	4	0	0	4	27.42
194	1	1	50	2	0	1	0	33.2	0	0	1	0	0	1	38.25
195	3	2	1	1	1	2	0	42.2	0	0	1	0	0	1	38.11
196	2	1	43	2	0	2	0	61.1	0	0	1	0	0	1	46.76
197	2	1	35	1	1	2	0	14.92	0	0	1	0	0	1	26.2
198	2	1	53	1	1	2	1	107.14	0	1	2	0	0	2	35.21
199	2	1	55	2	1	1	1	59.34	1	0	3	0	0	3	33.15
200	2	1	41	1	0	1	2	27.05	0	1	1	0	0	1	35.4
201	2	1	37	1	0	2	1	56.24	1	0	1	0	0	1	30.75
202	1	1	61	1	0	1	0	79.51	0	0	1	0	0	1	23.99
203	2	1	35	1	0	1	0	53.93	1	0	1	0	0	1	24.79
204	3	2	39	1	0	1	2	63.92	1	1	1	0	0	1	43.47
205			57	1	0	1	0	19.6	1	0	1	0	0	1	30.93
206	2	1	59	2	0	2	1	56.23	1	1	1	1	0	3	19.98
207	3	2	62	1	1	1	1	37.33	1	0	1	0	0	1	30.63
208	2	1	47	2	0	2	1	39.62	1	0	1	0	0	1	26.2
209	2	1	69	1	0	1	0	43.21	1	1	1	0	0	1	26.01
210	2	1	56	1	0	1	1	1.425	0	0	1	0	0	1	37.57
211	2	1	53	2	1	1	2	47.78	1	0	3	0	0	3	31.145
212	1	1	44	1	0	2	1	14.24	0	0	1	0	0	1	31.035
213	2	1	56	2	1	2	2	72.87	1	0	2	0	0	2	29.365
214	3	2	50	1	0	1	2	83.98	1	0	2	0	0	2	35.35
215	3	2	48	1	0	1	0	44.97	1	0	1	0	0	1	17.35
216	4	2	53	1	1	2	0	74.17	1	1	1	0	1	4	34.64
217	2	1	46	1	0	2	2	34.47	0	0	1	0	0	1	29.155
218	2	1	52	1	0	1	0	38.26	0	0	1	0	0	1	30.515
219	2	1	55	2	1	1	2	44.77	0	0	1	0	0	1	36.505
220	3	2	50	2	1	1	2	63.18	0	0	1	0	0	1	30.6
221	3	1	52	1	1	1	2	114.11	1	0	3	0	0	3	35.46
222	2	1	49	2	1	2	1	49.17	1	0	1	1	0	3	39.71
223	2	1	58	1	0	1	1	42.11	0	0	1	0	0	1	17.14
224	2	1	44	1	1	2	1	41.65	0	0	1	0	0	1	28.115
225	3	2	65	1	1	2	1	30.44	0	0	1	0	0	1	53.405
226			43	1	0	2	1	110.14	1	0	2	1	0	3	26.21
227	3	2	59	1	1	1	1	23.47	0	0	1	0	0	1	39.42
228	4	2	76	1	1	1	2	88.62	0	0	2	1	0	3	38.52
229	3	2	26	2	0	1	1	18.66	0	0	1	0	0	1	44.33
230			48	1	1	1	0	27.23	0	0	4	1	1	4	43.45
231	1	1	62	1	1	1	0	45.23	0	0	1	0	0	1	45.87

232	2	1	26	2	1	2	0	90.32	1	1	2	1	0	3	39.3
233	3	2	47	1	1	2	1	103.49	1	1	4	0	0	4	32.04
234	4	2	63	1	0	1	0	121.09	1	1	4	1	0	4	41.5
235			29	2	1	1	2	77.11	0	1	2	0	0	2	39.595
236			35	2	0	2	2	63.56	1	1	1	0	0	1	42.055
237			70	1	1	2	0	44.49	0	0	1	0	0	1	19.285
238			51	2	0	1	0	42.91	0	0	1	0	0	1	36.615
239			42	2	1	2	1	59.61	1	0	1	0	0	1	29.76
240			40	2	0	1	1	24.26	0	0	1	0	0	1	33.91
241			39	1	0	2	0	28.79	0	0	4	0	0	4	32.495
242			65	1	0	2	0	30.26	1	0	1	1	0	3	34.85
243			44	2	1	1	1	38.44	0	0	1	0	0	1	49.59
244			72	2	0	1	1	15.82	0	0	1	0	0	1	43.42
245			50	1	0	1	2	35.56	0	0	1	0	0	1	36.905
246			58	1	1	1	1	50.32	0	0	1	0	0	1	37.875
247			72	1	1	1	1	20.34	0	0	1	0	0	1	34.71
248			29	2	0	2	2	32.73	0	0	1	0	0	1	39.45
249			47	2	0	2	1	83.03	1	0	2	0	0	2	35.78
250			76	2	1	2	0	56.91	0	0	1	0	0	1	31.55
251			37	1	0	1	1	51.96	0	0	1	0	0	1	32.51
252			59	2	0	1	0	80.15	0	0	1	0	1	1	29.825
253			70	1	1	2	0	73.72	1	0	2	0	2	2	41.05
254			38	2	0	1	0	63.63	0	0	2	0	0	2	37.9
255			66	2	0	2	1	12.74	0	0	1	0	0	1	28.87
256			57	2	0	1	2	55.08	0	0	3	0	0	3	33.68
257			78	1	1	1	1	98.32	1	0	2	0	0	2	41.3
258			72	2	1	1	0	81.85	1	0	2	0	0	2	33.12
259			61	2	0	2	0	55.01	1	0	1	0	0	1	40.27
260			52	2	1	1	2	19.59	0	0	1	0	0	1	40.4
261			49	1	0	1	1	21.28	0	0	1	0	0	1	32.72
262			54	1	1	2	1	47.36	1	0	1	0	0	1	37.04
263			56	1	0	2	1	27.45	0	0	1	0	0	1	48.18

CMP	NP	DP	NCP1	CMP1	NP1	DP1	AX1	AX2	AX3
59.48	67.58	68.16	36.17	192.505	176.6	125.84	0.984394	0.899752	29.57354
17.19	19.02	17.775	32.44	133.265	135.68	93.275	0.804714	0.764563	34.76453
105.31	85.72	75.625	33.975	139.285	128.39	145.41	0.913655	0.812273	38.57083
156.73	113.8	91.4	37.395	165.975	143.89	115.465	0.951285	0.869126	13.31081
146.875	102.4	77.23	31.01	128.165	140.51	142.235	0.813321	0.769322	23.41182
41.64	48.14	49.715	30.42	110.785	125.97	108.91	0.873116	0.802494	35.48681
108.125	76.23	64.235	26.08	143.395	97.655	88.605	0.851263	0.793712	29.28619
86.5	58.8	52.99	31.735	109.285	114.8	109.16	0.823931	0.701304	31.93443
85.53	67.13	58.15	29.6	120.35	127.42	107.57	0.822772	0.701016	13.56092
180.3	110.8	75.735	37.75	181.42	155.62	127.965	0.823782	0.736775	46.3662
40.72	63.96	55.455	33.475	38.9	119.215	100.83	0.760371	0.70367	76.45108
81.945	74.36	77.865	34.72	184.335	162.49	162.01	0.752097	0.681648	89.59063
141.58	102.5	77.815	38.46	141.325	132.625	113.31	0.917636	0.844529	41.42841
200.165	125.7	76.06	33.47	188.345	142.61	100.53	0.192279	0.157414	21.53337
104.995	80.54	69.03	29.54	141.91	114.115	117.115	0.975184	0.666841	12.67124
106.955	77.36	64.59	34.09	140.985	115.93	121.32	0.723728	0.695375	62.88057
119.045	81.14	64.65	19.965	120.23	97.24	92.625	0.355186	0.289305	20.83539
149.03	103.4	75.46	28.51	100.44	141.99	121.645	0.881265	0.735689	43.92762
194.145	112.9	99.14	35.165	54.665	60.285	79.61	0.716958	0.509537	39.3866
115.905	93.53	70.195	28.065	103.295	127.295	113.065	0.8165	0.79953	69.86302
106.505	94.18	80.285	32.475	94.755	119.745	126.845	0.875734	0.762027	70.28665
171.59	115.8	73.88	33.115	157.72	129.63	101.585	0.895287	0.806086	47.68152
42.62	51.58	47.59	23.915	77.435	131.885	111.51	0.915246	0.721214	18.91237
78.25	79.15	68.43	39.895	141.035	121.77	125.66	0.847263	0.718163	56.37776
84.895	76.77	69.145	29.63	134.88	130.58	101.21	0.850873	0.795009	44.09204
130.3	86.99	67.455	40.015	139.255	121.45	94.28	0.746881	0.643454	21.1895
71.475	62.14	59.21	21.845	102.51	86.66	70.65	0.946809	0.835464	32.49643
211.485	112.4	86.42	38.965	154.02	142.35	135.37	0.712119	0.604768	33.0634
83.15	76.01	76.71	31.865	161.92	127.78	131.98	0.790048	0.67714	25.42137
120.565	87.84	72.955	33.64	91.025	125.615	123.4	0.899674	0.820674	35.50561
131.62	82.9	68.05	29.67	155.925	90.645	85.195	0.625698	0.591383	20.23813
123.715	84.3	70.705	32.565	174.62	132.31	121.85	0.927777	0.770297	48.73609
125.975	79.61	58.445	33.865	137.08	104.285	91.115	0.895586	0.799794	25.40015
133.15	102.7	83.085	25.05	170.6	126.715	122.22	0.711486	0.594912	39.13017
84.9	68.58	60.225	28.99	109	100.11	89.605	0.822039	0.760968	100.5626
120.87	69.9	73.545	35.365	102.975	97.82	85.385	0.830701	0.629886	20.83333
173.68	134.4	104.75	27.18	160.645	153.025	144.185	0.663435	0.609851	63.91656
77.365	64.72	41.165	31.515	169.315	126.375	114.18	0.863894	0.77558	23.33353
181.515	87.27	71.53	31.695	169.86	111.805	107.37	0.77014	0.706742	27.4581
105.99	118.8	71.72	24.325	72.315	131.13	111.955	0.855858	0.774376	16.12702
156.06	97.2	81.5	31.305	133.755	156.945	151.36	0.654626	0.582571	34.54983
234.02	119.4	94.33	35.475	216.395	164.775	151.76	0.929404	0.875798	82.88527
182.43	118	107.76	37.52	209.77	161.965	123.4	0.929628	0.858365	29.8108
143.14	85.77	68.78	37.56	167.27	154.395	146	0.799743	0.723889	56.58826
105.15	77.46	68.815	27.89	131.97	117.015	115.44	0.791655	0.607834	48.76254
226.08	129.7	82.86	35.78	207.74	171.735	146.435	0.883303	0.76262	21.02002
88.24	90.22	61.36	33.43	198.15	171.425	144.165	0.848139	0.702518	22.79115
162.83	106	80.96	29.49	168.7	145.985	135.895	0.786848	0.672229	38.95855
118.065	108	88.64	34.42	166.715	146.2	130.625	0.887515	0.728065	37.18455
106.32	96.41	76.72	33.54	157.635	160.39	123.34	0.637504	0.405785	30.7538
92.77	92.69	75.81	36.745	83.065	130.175	105.15	0.869191	0.741202	52.51968
153.81	99.6	78.245	34.535	129.885	114.44	123.4	0.890736	0.853543	21.95426
144.025	93.24	69.5	29.675	118.655	111.585	104.56	0.879129	0.829539	29.57886
170.26	102.6	77.635	41.725	135.395	181.025	149.955	0.600746	0.524836	28.99235
104.6	102.2	78.82	34.585	160.81	138.46	119.12	0.744438	0.674187	56.80893
85.985	112.7	85.015	29.88	87.16	137.76	138.9665	0.947071	0.828802	41.42902
61.045	122.1	91.345	32.825	79.24	151.135	135.815	0.751632	0.707166	32.13803

64.425	94.63	58.765	23.9	99.3	133.95	94.06	0.883995	0.818316	24.57444
156.555	94.51	89.345	34.845	166.89	133.28	139.745	0.8005	0.692471	20.72202
83.505	83.74	71.925	27.155	106.745	151.885	121.105	0.648891	0.636401	27.56702
85.515	89.14	89.31	35.745	179.355	150.205	150.59	0.861911	0.729678	92.31666
160.61	108.1	65.915	30.91	169.3	143.32	96.26	0.924517	0.75679	19.91279
80.555	69.33	61.875	29.425	76.615	76.385	90.51	0.929199	0.748851	47.51109
124.06	73.96	62.05	29.44	118.675	89.825	90.77	0.829735	0.747133	61.49623
159.67	99.95	80.855	31.1	114.595	122.35	100.855	0.705043	0.579039	58.28735
165.79	98.72	73.095	31.53	139.04	129.74	115.635	0.807687	0.69711	22.06972
125.355	84.67	63.765	36.12	157.97	124.78	112.565	0.728863	0.688856	19.68243
160.785	124.1	72.85	32.605	194.24	203.085	174.435	0.708873	0.666031	20.2398
77.71	74.07	73.88	23.27	108.03	106.76	97.785	0.879913	0.635059	48.47122
60.615	62.94	55.83	35.175	94.775	112.755	109.225	0.844522	0.68498	87.97347
153.725	123.4	77.515	37.975	166.09	208.03	132.075	0.965666	0.853745	66.04387
72.37	64.74	66.14	29.755	128.49	123.48	105.68	0.981275	0.781671	77.95158
62.935	74.44	65.575	25.77	70.61	104.225	97.73	0.712257	0.608163	58.68182
121.045	107.2	82.325	39.25	107.19	143.53	108.79	0.969929	0.860706	14.80377
97.51	80.19	63.475	32.5	154.77	153.565	118.985	0.71734	0.688069	33.3323
118.895	81.42	71.025	28.925	129.52	132.425	93.575	0.766402	0.700568	68.24086
84.8	91.87	60.88	31.51	83.145	108.625	110.63	0.6982	0.605488	29.32634
95.725	62.79	55.3	35.445	196.895	121.795	120.82	0.797107	0.630173	49.74949
110.08	91.62	65.845	29.11	88.005	121.85	121.895	0.786084	0.746459	34.94679
139.315	101.1	105.62	32.295	122.39	159.05	168.3	0.88974	0.798522	34.24692
157.065	117.6	90.13	31.84	133.09	150.37	140.74	0.933815	0.839185	49.66224
88.385	82.12	64.165	35.575	171.345	142.225	100.45	0.922909	0.817764	21.58481
141.6	94.53	67.92	30.21	146.505	142.075	116.97	0.853056	0.742306	58.14296
60.845	75.41	67.335	23.845	101.26	109.13	120.895	0.648793	0.603644	82.06648
133.25	73.76	55.55	28.49	145.365	145.365	145.365	0.841532	0.717185	43.29036
105.21	75.45	57.755	24.155	197.21	164.96	117.155	0.659823	0.575436	28.31946
145.065	92.7	60.33	23.49	129.62	129.41	118.46	0.898706	0.808986	37.6304
143.11	76.8	61.44	30.005	159.235	134.965	89.225	0.822673	0.710952	18.76675
175.175	113.2	93.585	37.4	166.52	150.175	128.635	0.799554	0.772606	46.65827
145.175	72.82	51.515	30.995	128.575	122.035	111.175	0.89699	0.811931	62.7092
98.53	99.56	89.07	31.395	138.36	175.665	131.265	0.960434	0.780297	39.92916
148.96	144.2	116.97	38.4	163.79	157.565	151.485	0.850845	0.721752	28.14227
208.765	151.7	125.55	31	169.915	162.9	153.51	0.822976	0.786579	41.03433
167.93	120.7	83.095	31.99	121.73	133.85	116.57	0.920269	0.812078	21.78833
111.825	69.68	61.695	34.31	137.865	137.84	122.88	0.885891	0.713148	42.02049
169.195	125.3	99.765	34.605	199.185	177.78	123.5475	0.700295	0.605747	12.03819
64.525	69.46	60.85	33.45	135.25	190.665	127.275	0.366274	0.326975	18.25451
88.865	72.8	52.87	31.215	161.55	146.76	114.915	0.891946	0.788629	26.13504
83.165	92.97	87.53	34.205	158.62	163.09	136.825	0.899293	0.850328	36.72785
131.85	93.82	67.99	31.91	226.71	173.53	131.465	0.795277	0.697914	20.25449
124.48	115.2	86.39	31.33	152.97	188.465	127.81	0.878915	0.79421	42.80084
90.755	71.39	60.32	27.57	143.245	102.225	89.675	0.813897	0.681446	41.22187
173.92	108.9	60.64	38.545	145.9	183.82	140.165	0.824612	0.728296	23.03633
79.95	65.95	63.65	28.945	149.85	118.01	111.56	0.923835	0.58451	25.71525
89.14	84.52	72.555	32.275	150.73	139.035	126.85	0.834282	0.81736	40.5382
105.635	104.1	91.905	35.025	176.355	177.955	130.395	0.829255	0.773546	19.51104
89.61	108.9	82.04	32.25	112.915	146.405	115.91	0.910145	0.804684	28.41382
118.69	87.54	73.65	38.115	142.08	129.435	99.81	0.756801	0.587658	41.30359
108.02	78.64	65.95	30.1	106.135	143.285	118.095	0.735073	0.694987	62.07698
78.855	85.52	60.19	34.023	121.89	136.905	117.345	0.878913	0.731663	58.75002
200.33	109.2	88.37	36.41	178.825	150.505	121.665	0.88478	0.823942	26.0525
176.155	78.12	50.735	31.825	145.46	143.325	123.815	0.93119	0.730221	27.48055
83.95	75.44	58.63	19.345	91.65	91.195	95.84	0.89733	0.767886	77.8299
65.25	63.65	56.695	34.255	141.715	111.075	92.145	0.701539	0.562143	26.34023
125.4	78.99	65.82	33.19	114.625	111.57	99.685	0.806947	0.644246	36.87361

65.35	54.09	45.355	29.54	148.495	98.175	95.21	0.855331	0.80094	25.42767
147.1	96.28	65.92	35.66	168.195	139.655	94.45	0.919085	0.843724	24.8393
192.73	88.63	66.955	29.19	160.55	114.745	109.28	0.670801	0.645335	66.06837
180.73	95.04	78.37	31.58	133.795	115.215	111.88	0.882966	0.780896	19.84008
107.22	86.74	65.965	37.67	121.74	130.585	93.235	0.711906	0.677028	28.52579
192.535	123.7	93.175	31.54	203.9	190.865	194.76	0.79095	0.646082	45.74156
126.305	100.6	84.64	32.085	137.45	116.23	109	0.945431	0.845094	24.36607
47.11	47.28	48.545	23.56	135.58	117.415	116.895	0.848311	0.838704	47.45189
177.685	72.1	65.435	31.08	224.755	114.15	96.285	0.977521	0.803096	21.53987
248.84	127.2	93.035	35.53	153.555	174.685	149.975	0.958112	0.942756	30.85808
147.38	107.5	83.6	31.185	161.36	176.185	120.395	0.866618	0.767253	35.41942
78.795	57.04	62.815	32.07	96.105	98.105	95.275	0.693454	0.646242	29.87851
97.315	95.45	75.09	34.64	180.925	156.44	129.9	0.715203	0.59849	41.28255
182.155	131.2	94.39	31.475	162.405	109.7	79.245	0.848693	0.779727	44.10318
111.01	86.21	69.46	29.865	80.4	101.14	104.385	0.69114	0.679263	59.20638
138.89	137.5	94.905	36.81	107.445	146.56	118.62	0.910379	0.820264	30.73154
41.675	53.3	38.96	27.85	98.875	101.79	111.71	0.849571	0.743392	52.90436
174.1	109.1	79.475	30.98	154.6	132.445	123.69	0.691628	0.568431	76.7866
167.58	111.4	94.215	33.355	226.86	177.175	119.645	0.934384	0.671816	15.87084
168.415	137.9	90.49	36.24	118.73	273.325	166.285	0.333849	0.311296	32.82808
66.405	64.14	55.295	34.005	143.885	167.99	114.715	0.903029	0.749181	19.6314
193.085	106.7	85.4	38.835	159.495	153.885	130.275	0.450293	0.389627	33.5339
249.795	138.4	112.26	40.895	185.815	183.685	168.11	0.953303	0.802504	25.63241
198.06	77.49	60.81	33.065	150.465	94.455	78.375	0.891151	0.813702	27.41905
76.25	102.4	85.905	30.835	205.73	180.995	144.825	0.740418	0.624241	48.13997
195.82	119.9	93.08	33.03	221.73	166.09	168.485	0.899301	0.792657	31.49534
198.215	117.2	84.76	32.97	167.12	121.21	115.675	0.849378	0.825009	34.85656
40.335	50.2	46.28	29.455	96.71	106.34	95.815	0.765927	0.741947	48.40386
64.94	70.49	58.56	34.88	155.5	165.29	126.33	0.773211	0.691039	23.58148
103.23	102	73.95	30.85	152.28	165.76	153.8	0.816381	0.760193	32.00744
92.9	74.61	62.53	39.32	120.17	124.08	135.5	0.628025	0.571317	13.36903
124.9	122.8	106.62	32.13	157.67	157.38	136.78	0.73933	0.689886	27.88609
65.56	82.61	58.24	31.95	120.8	137.3	156.05	0.858683	0.75118	49.91205
62.15	77.59	68.65	37.72	104.68	105.93	109.14	0.853311	0.795015	58.61826
63.78	74.61	78.48	33.67	98.05	125.25	121.54	0.828807	0.727109	23.45996
125.39	94.75	75.65	38.95	143.7	131.88	107.04	0.536303	0.513566	16.41019
206.55	145.8	105.51	29.6	176.76	187.22	145.43	0.883236	0.834237	29.30997
67.82	59.66	53.33	35.59	129.67	136.44	141.78	0.901389	0.782254	43.55009
84.95	69.99	75.13	27.31	62.44	70.37	86.15	0.83086	0.720505	48.68327
85.22	111.8	71.46	30.46	125.11	132.98	102.44	0.847907	0.774355	38.15036
151.32	117.4	81.85	33.83	157	127.45	131.35	0.94229	0.762914	35.3983
71.1	52.4	53.63	33.28	123.36	135.85	115.14	0.738907	0.690568	21.91235
63.65	76.36	70.66	31	130.73	141.63	150.88	0.857336	0.79138	45.61604
37.04	56.12	40.14	24.7	107.29	130.98	133.03	0.749586	0.602231	13.87013
79.77	93.52	77.97	30.04	131.71	139.71	151.83	0.75685	0.697638	31.7362
134.95	110.5	110.37	36.38	139.33	133.68	147.05	0.695135	0.548813	31.89667
108.06	71.24	57.94	32.45	106.95	121.23	132	0.898881	0.80044	27.95218
117.53	93.37	82.85	35.5	82.05	102	110.47	0.853446	0.759876	59.55211
137.93	120.2	89.87	30.63	133.82	152.15	157.4	0.81889	0.765638	32.04614
93.3	61.48	51.72	34.48	118.33	118.42	118.41	0.964745	0.782555	52.44421
137.86	106.8	78.7	38.5	139.65	123.5	157.83	0.917928	0.659976	30.88782
50.38	70.9	58	31.17	81.92	99.11	119.7	0.633723	0.560933	29.39908
74.73	58.64	44.34	29.95	131.5	127.71	102.07	0.901959	0.754265	12.10738
97.56	66.36	57.82	31.55	95.84	85.46	104.29	0.539378	0.493721	29.17982
104.36	78.82	55.18	32.1	111.79	107.71	102.95	0.990393	0.890712	43.99758
94.66	98.69	85.31	29.79	106.6	122.35	133.46	0.727974	0.695484	17.20791
77.99	114.7	92.45	35.25	137.38	155.38	136.83	0.410795	0.277945	65.07935
85.29	75.82	64.07	29.47	113.65	130.38	104.5	0.784439	0.692907	31.57714

53.44	67.71	69.8	35.58	104.8	131.48	129.01	0.896792	0.77539	54.84402
51.59	92.22	75.26	35.81	93.55	154.77	151.27	0.571806	0.477412	31.40493
49.09	78.21	66.75	31.52	48.31	111.33	101.86	0.807959	0.74173	34.1057
149.02	87.47	74.5	32.04	127.47	122.54	120.58	0.78603	0.708312	31.06298
77.21	75.19	64.84	29.32	109.88	70.57	82.5	0.696963	0.570178	45.22973
81.44	77.2	62.08	34.84	102.81	111.52	105	0.883341	0.753957	31.15396
122.65	109.2	96.39	32.64	136.58	176.17	160.04	0.721158	0.474981	30.58507
130.41	111.7	110.45	28.53	159.75	137.27	127.88			
88.33	58.33	44.28	35.08	132.4	117.45	112.54	0.714463	0.654643	34.44482
45.17	46.35	54.42	35.89	137.05	110.69	100.33	0.824587	0.712697	117.6781
70.05	68.8	58	32.66	76.11	118.47	93.93	0.713925	0.660601	40.71914
80.12	67.11	57.16	32.36	114.91	99.37	101.06	0.737075	0.653561	25.41658
91.03	97.48	88.44	32.84	101.81	121.85	110.65			
92.05	79.14	69.93	29.79	153.55	126.55	120.41	0.912054	0.812623	89.5438
151.82	107	82.39	25.22	126.07	143.5	123.54	0.723427	0.684079	50.32279
150.04	86.69	74.77	34.2	117.33	129.5	131.81	0.89101	0.810722	71.77697
65.89	61.12	69.88	36.87	115.66	124.43	130.52	0.922456	0.702474	13.74597
87.93	86.25	58	34	98.4	117.63	141.31	0.820057	0.790384	37.78956
51.89	45.44	38.89	28.57	102	122.38	113.88	0.799462	0.686476	61.30548
56.72	53.42	57.58	29.3	93.5	84.45	111.63	0.776314	0.590772	44.79227
95.35	102.4	87.08	36.14	125.31	133.83	141.25	0.959029	0.87874	28.03697
93.8	70.35	75	36.04	129	133.71	112.32	0.848149	0.794784	30.54502
102.97	100.3	86.39	31	138.69	149.56	153.58	0.812451	0.694032	44.55605
96.73	100.9	77.08	27.94	94.44	117.81	123.33	0.910563	0.683377	13.14888
69.88	68.65	54.2	31.07	64.66	88.59	102.32	0.948801	0.800544	67.45132
110.68	90.7	73.68	32.07	80.56	131.28	131	0.807671	0.73897	51.73516
84.9	67.31	54.38	28.28	91.18	110.28	113.77	0.59132	0.585962	12.81198
45.63	82.72	69.18	25.72	129.33	133.69	104.77	0.867996	0.759886	44.24571
66.44	59.34	60.94	29.56	114.5	129.42	138.38	0.844653	0.73149	56.80049
80.97	72.38	64.5	37.27	113.88	107.2	110.7	0.993722	0.800233	39.28167
129.73	90.75	67.38	31.2	125.55	106.83	118.9	0.925855	0.730513	39.91238
82.71	98.95	79.32	27.59	108.17	110	92.91	0.923176	0.842148	15.08564
86.53	53.68	35.28	32.29	105.25	84.81	83	0.874394	0.812543	38.93526
71.45	82.65	67.41	31	106.3	114.96	119.9	0.722738	0.62896	30.99216
96.73	100.9	77.08	27.94	94.44	117.81	123.33	0.874671	0.855542	33.42353
91.31	108.7	69.75	30.25	94	131.4	111.46	0.899811	0.83871	34.94858
69.69	74.69	51.735	36.67	143.625	97.64	95.865	0.840766	0.719507	8.129642
42.585	47.62	48.025	38.885	153.67	131.93	127.955	0.814846	0.49153	22.02618
148.78	117.3	88.745	42.895	166.25	155.675	135.215	0.916747	0.45705	9.517249
48.905	54.56	49.205	34.545	171.48	129.895	140.185	0.584677	0.550004	38.85405
112.545	89.15	69.995	38.625	168.765	157.835	137.785	0.802911	0.729662	62.92168
20.11	29.47	31.22	41.625	161.025	114.305	103.465	0.759072	0.684348	34.53164
142.07	99.43	79.08	31.76	149.34	118.3	106.495	0.706072	0.605678	41.85623
37.2	49.88	41.375	33.87	119.93	120.15	111.375	0.922125	0.84711	27.72267
34.695	48.79	57.18	30.29	77.53	96.865	80.575	0.985439	0.776793	25.77031
53.76	62.32	66.175	32.135	177.76	135.635	130.225	0.929415	0.918479	28.1304
57.125	67.93	73.055	34.81	181.62	145.795	154.79	0.868355	0.762747	39.84534
50.645	61.08	55.375	44.67	68.255	83.31	91.685	0.827128	0.77982	81.92941
53.605	70.62	83.81	32.725	204.285	169.475	143.785	0.877612	0.787996	36.7806
28.875	49.7	42.355	29.355	203.225	125.135	95.375	0.722738	0.574677	20.79627
40.82	41.23	45.055	32.49	121.265	82.485	90.635	0.910092	0.815538	30.26053
104.175	77.24	81.98	31.9	210.495	151.37	149.115	0.582906	0.531186	18.49451
33.74	42.48	39.86	32.67	136.53	149.25	149.86	0.807624	0.752336	75.84019
44.73	50.56	46.13	24.06	110.09	126.44	104.67	0.731835	0.514395	16.70309
61.81	61.9	65.46	29.81	146.05	137.29	136.05	0.905072	0.723694	51.88482
61.16	80.49	87.96	33.11	161.64	138.2	127.57	0.751316	0.559743	11.93908
60.8	73.68	67.25	34.5	146.82	158.76	151.89	0.538448	0.466383	21.80864
47.87	60.39	60.71	35	108.1	152.19	125.6	0.907579	0.678956	30.94649

66.51	76.51	66.43	40.94	149	126.72	119.8	0.629785	0.552776	56.0139
111.02	74.26	69.93	29.82	100.38	169.45	132.52	0.882838	0.654305	69.46235
42.36	38.36	38.77	33.04	132.83	124.05	126.44	0.625375	0.530439	60.91434
102.71	72.23	69.375	32.15	211.845	143.25	142.935	0.89994	0.78333	59.50058
137.14	86.56	70.695	32.11	186.165	161.065	161.745	0.888972	0.825541	46.1253
25.69	37.28	30.855	22.525	79.245	110.835	91.04	0.682756	0.659432	31.0057
75.555	61.82	52.065	35.385	154.9	107.79	116.63	0.895527	0.729269	28.31645
83.43	52.84	46.135	33.295	180.425	151.24	121.49	0.832754	0.738912	42.25244
106.65	76.52	65.945	40.1	214.36	163.28	130.815	0.939136	0.843418	21.92698
53.25	53.71	49.4	27.875	95.98	123.12	99.15	0.703011	0.597474	23.86685
108.74	103.9	70.42	29.07	124.54	151.69	125.77	0.882156	0.775255	23.644
97.21	79.84	69.365	27.525	205.115	189.87	122.26	0.873161	0.849922	29.89729
111.625	90.81	80.2	32.895	178.505	210.495	171.335	0.880649	0.840676	13.82537
65.44	87.74	67.155	43.67	60.985	97.17	86.335	0.857694	0.829015	23.49386
109.62	95.17	67.5	22.285	132.8	116.925	118.33	0.891754	0.847022	40.8408
71.295	57.47	51.245	33.91	167.15	121.89	100.8	0.853817	0.614235	14.40435
67.615	58.37	63.945	40.73	165.32	147.45	131.44	0.778304	0.717314	25.77686
153.575	129.7	99.35	30.925	152.42	147.915	135.765	0.920403	0.861494	70.05193
67.05	64.58	57.63	30.395	170.435	159.145	112.3	0.870327	0.711296	38.27719
91.635	98.91	91.215	34.5	219.285	151.21	150.375	0.87752	0.796881	41.09002
73.37	61.33	60.51	28.72	175.24	157.32	158.39	0.593432	0.555358	40.35736
112.34	119.6	110.64	35.21	157.15	153.33	152.98	0.723895	0.6181	59.7991
70.76	64.71	53.9	34.9	99.54	102.71	120.21	0.667599	0.538674	43.80566
52.97	83	54.84	39.89	133.5	129	100.38	0.832931	0	0
58.48	79.31	61.1	30.34	70.5	134.92	136.96	0.841259	0.667969	33.71628
85.21	76.89	66.88	40.12	75.59	105.88	104	0.741905	0.590946	53.03871
98.04	77.81	62.03	27.1	128.17	134.06	94.45	0.914376	0.837395	63.41445
146.12	95.65	68.67	32.78	136.56	116.84	98.05	0.828994	0.700265	40.06349
78.16	64.78	63.21	33.45	168.77	148.34	134.83	0.853232	0.782902	17.87611
43.2	31.59	37.56	25.66	110.4	98.75	87.32	0.775317	0.675908	14.01876
111.15	80.8	67.13	33.8	109.64	106.11	120.58	0.638003	0.607127	32.60103
98.53	88.52	69.59	36.43	170.37	166.16	145.11	0.850894	0.764086	23.35281

AX4	AX5	AX6	AX7	AX8	AX9	AX10	AX11	AX12
32.86855	39.46285	39.68352	36.50957	41.19324	22694.27	32.3556	0.791066	4900.221
45.46978	53.82467	52.8841	42.89247	54.3791	40770.8	36.59015	0.735588	7787.813
47.48505	55.62359	54.96638	50.61733	59.41264	57111.97	43.38496	0.778704	9210.068
15.31516	19.15581	19.53188	18.18603	19.97204	2143.368	14.56908	0.802361	1001.949
30.43177	33.87037	37.12837	28.97018	37.67507	12555.19	24.75081	0.793172	3293.563
44.22064	48.61749	51.25293	51.63534	52.29248	41885.15	38.60975	0.735407	7931.034
36.89775	48.39983	41.6358	41.64874	48.61404	23207.25	31.4097	0.725366	5424.285
45.53577	47.37355	52.43892	51.07803	53.24798	38557.6	37.51831	0.749274	7366.31
19.34468	22.96229	23.31678	19.08927	25.28086	2932.372	15.91627	0.77201	1283.337
62.93129	73.66658	70.41119	63.62221	76.61139	102628.9	51.84166	0.702534	15089.09
108.6462	129.1573	116.4029	106.0551	136.4217	476244.4	82.6114	0.694592	42459.42
131.4325	151.4641	147.3659	113.0732	152.1482	811250.6	98.85001	0.696959	60355.07
49.05506	57.90159	56.07748	55.64618	59.13411	65081.84	45.01469	0.743233	10527.67
136.7945	31.53	30.57394	285.6852	285.6852	9876.872	26.3027	0.769788	2891.961
19.00189	21.59891	21.81798	21.50327	22.81563	3143.616	18.53034	0.765504	1355.677
90.42682	97.86718	104.5573	89.80282	107.8556	256204.3	65.44443	0.698599	27924.16
72.01871	30.77962	29.7344	24.17821	320.0226	7759.98	25.58001	0.786989	2408.551
59.70946	66.73631	69.10315	65.4868	70.6066	97356.68	52.61984	0.723968	14136.51
77.29881	90.33104	76.12085	79.55538	92.04342	89739.72	55.41996	0.571134	16972.12
87.38016	101.779	101.2639	84.68713	102.9699	309647.4	71.34589	0.738879	29956.35
92.23647	103.2911	101.8385	101.4663	109.1489	366654.4	80.77465	0.723762	34228.91
59.15188	68.06714	67.90831	69.80464	72.81966	104911.8	52.95793	0.726879	14799.19
26.22298	32.06292	29.61959	29.48166	32.52957	8338.908	24.00047	0.777959	2556.232
78.50272	93.66752	88.70541	82.7456	95.63064	205019	66.51245	0.731111	22998.37
55.46104	67.72229	58.4257	62.46718	69.71732	81374.92	47.19031	0.756583	12002.95
32.93085	30.15073	31.22	320.5346	321.9716	9706.357	24.59542	0.81627	2695.8
38.89625	47.34333	48.19437	42.08779	49.49839	32494.24	36.82732	0.737263	6679.328
54.67117	64.23918	59.5069	51.18858	68.02662	49235	38.93237	0.714406	9093.297
37.54227	42.09497	38.76142	39.63291	43.00497	19584.25	29.66019	0.755087	4653.268
43.26395	53.18449	53.18449	44.68004	55.21966	41480.06	38.92344	0.774588	7481.224
34.22171	32.23888	38.99919	39.49922	40.945	9518.499	21.41245	0.714609	3039.449
63.26923	75.07443	74.36121	74.44408	78.85242	126159.4	58.69975	0.719557	16905.65
31.75837	36.28683	40.68386	34.0888	40.87283	15758.01	28.44236	0.758853	4005.539
65.77474	57.76615	73.08096	70.66496	74.81696	80016.36	46.79782	0.664437	13515.01
132.1509	146.327	158.8989	184.264	190.4847	998355.8	108.6333	0.661207	73058.46
33.07474	36.2866	36.76731	37.006	43.42322	12436	27.47521	0.702155	3696.902
104.8069	103.9352	102.871	111.051	116.3174	323302.2	69.53255	0.699018	32588.79
30.08527	33.69971	37.35635	33.01478	38.31404	12870.92	25.99049	0.767561	3460.276
38.85165	44.57798	41.37869	41.70378	48.29563	21382.66	29.92121	0.745989	4994.123
20.82581	25.7448	25.3415	20.13291	25.94815	4241.854	17.82393	0.791667	1600.707
59.30576	52.85605	71.77357	57.49427	76.47987	51567.05	38.82308	0.653996	10244.49
94.63968	114.607	111.6486	103.718	119.9198	495641.4	87.95852	0.740072	40924.97
34.72973	43.00255	41.63366	37.06683	43.27799	24154.99	32.28573	0.794809	5084.241
78.17253	92.42462	87.22403	76.0117	96.38441	177458.3	62.51796	0.668066	22859.28
80.22344	93.25867	92.49991	79.27107	96.12831	154030.2	63.50932	0.573275	24239.58
27.5629	32.90226	34.05533	27.4655	35.83432	9750.56	24.34639	0.762654	2894.076
32.44211	40.76783	42.2167	32.77015	42.58979	14199.79	27.51543	0.741492	3824.418
57.95432	68.45925	55.48912	64.84258	72.59385	71969.83	45.60121	0.720702	11609.91
51.07311	62.0483	62.36031	53.17803	62.85206	58998.21	45.32815	0.736124	9956.174
75.78839	63.12639	77.42274	79.06505	80.80031	75850.47	48.31544	0.659653	13136.33
70.85742	86.29655	85.5392	78.033	87.30753	143435.3	61.58864	0.648553	20431.92
25.72134	32.04737	31.61938	27.35209	32.95923	9241.759	22.91092	0.811978	2622.866
35.65698	43.38365	42.12264	37.54689	43.60565	23621.88	31.34709	0.793553	5017.079
55.24075	57.06827	55.13306	42.8148	58.31527	32152.79	33.18568	0.640027	7640.09
84.26293	95.19183	98.43471	85.41094	101.3235	202506.1	62.72849	0.692563	24079.64
49.98663	67.00784	58.8591	55.05708	67.24059	67775.9	47.34087	0.728153	11040.24
45.44622	51.7286	50.8546	39.66888	53.13598	35195.84	34.15884	0.77549	6697.34

30.03051	36.82353	35.5881	32.69828	37.64623	13756.44	26.54684	0.746018	3721.676
29.92475	34.45382	31.27702	32.07641	35.76282	10474.22	23.95475	0.806171	2871.692
43.31704	38.06989	49.60808	42.76942	50.1039	22843.48	28.10803	0.735378	5294.381
126.5171	141.6372	141.6372	136.1714	145.0499	914845.8	109.0465	0.727088	62680
26.31219	30.03107	29.13891	29.39488	31.96257	9168.865	24.32608	0.790314	2680.578
63.44535	75.78435	77.44596	70.05892	79.52776	116119.7	58.95338	0.678408	16966.68
82.30958	85.93665	99.06006	100.2399	103.2336	227331.5	68.29516	0.675198	26678.36
100.6623	115.2146	99.30385	97.19386	124.4064	278723.5	70.97122	0.630877	32708.09
31.65887	34.50212	36.49258	30.86259	39.28172	12579.87	25.57046	0.772129	3387.752
28.57265	25.99877	34.7982	32.27693	35.86956	7992.608	20.82554	0.767662	2518.294
30.38867	35.00381	35.00381	26.89352	35.97812	9040.582	21.54172	0.770029	2725.468
76.32549	84.16471	79.11222	80.41457	89.70314	171288.5	67.15981	0.710137	21003.62
128.4322	144.7699	151.7444	133.91	153.6851	851066	108.4637	0.676102	64236.34
77.35782	88.64112	96.84626	89.30563	97.45022	268976.4	74.70183	0.711748	28311.77
99.72424	122.9258	123.8801	115.7052	126.465	511881.3	97.85688	0.683525	45273.33
96.49022	83.47951	103.0328	355.6831	355.856	261110.1	68.72584	0.702871	28107.63
17.19957	22.19381	21.71608	20.31484	22.53835	2933.942	16.68235	0.793809	1248.54
48.44324	56.98843	52.30453	41.93947	57.88146	40135.17	34.75028	0.776496	7300.648
97.40794	99.39484	101.4444	112.4195	113.7603	313839.2	74.65362	0.534127	41812.93
48.43425	52.68408	53.11755	41.81485	53.54339	31460.64	33.81678	0.674511	7145.074
78.94582	84.00092	92.36688	76.61131	92.73043	167944.3	62.92827	0.702213	20963.26
46.81673	55.29255	51.28958	47.20263	58.48384	42029.19	36.80189	0.728455	8025.074
42.88789	44.81891	53.10652	52.53008	54.02744	38155.18	38.15907	0.609389	8994.118
59.1791	68.28515	69.20287	102.1241	129.7001	92179.31	55.26237	0.691971	14261.05
26.39492	30.90766	30.2643	32.37603	32.45765	9797.506	24.36011	0.708818	3123.869
78.32751	92.35626	93.42208	81.46124	100.314	206491	66.81772	0.692952	24380.83
135.9519	144.6809	153.7865	110.1642	158.2329	670691.8	88.20457	0.683359	54223.06
60.36149	67.33603	64.0402	66.09037	68.15011	93556.46	50.79611	0.756107	13181.04
49.2139	58.05902	58.93721	40.55585	59.57658	29812.87	32.47248	0.627617	7408.418
46.51553	54.06234	55.50742	49.24763	55.57982	52573.19	41.80379	0.780177	8698.96
26.39664	31.74536	30.0459	25.30952	33.11348	7604.163	21.7158	0.796736	2347.13
60.3908	65.86081	67.20433	67.17678	69.78833	93246	48.28573	0.695464	14298.7
77.23468	89.24273	90.68935	81.29393	92.34392	237500.9	69.27871	0.754408	24584.13
51.17175	60.50907	57.54038	58.49476	63.08799	71306.17	49.14708	0.729453	11400.01
38.99159	46.48117	41.41924	41.36876	47.37304	25668.77	33.17582	0.763427	5512.137
52.1681	61.17508	64.68543	53.40725	65.13558	65079.4	42.93307	0.747854	10462.35
26.83035	33.74284	29.77548	28.93806	33.81683	9931.278	24.69113	0.791393	2823.333
58.92256	66.81139	66.30041	64.46043	73.34501	90345.82	52.19896	0.736768	13215.75
19.87329	24.62041	18.97498	20.42141	25.20157	2383.539	13.91716	0.755355	1142.39
55.82842	25.58162	25.29997	257.4785	257.9143	4606.544	20.4485	0.752289	1779.711
33.13982	39.98332	38.80234	41.23027	41.61506	17725.1	29.55894	0.716044	4591.312
43.19255	50.03514	52.65625	49.72463	53.2407	43991.54	38.84275	0.749344	8042.339
29.02146	34.59315	28.31191	35.94088	36.88205	7973.525	23.08011	0.658214	2932.358
53.89109	62.58779	58.86191	59.83516	63.22207	78290.12	47.3657	0.737505	12000.25
60.49179	68.21255	71.6187	59.54112	72.10038	83899.96	49.2341	0.718544	12898.49
31.63047	37.99306	35.7409	32.03997	38.69741	13370.69	26.08288	0.77138	3531.708
43.99457	50.41417	54.30164	50.61082	55.49275	30079.84	40.64374	0.685564	6822.651
49.59652	53.15952	57.27539	57.52851	58.38847	59210.13	41.37747	0.77596	9467.651
25.22285	29.22534	30.56126	25.5824	31.31646	7218.371	20.91616	0.759786	2377.309
35.31052	40.36208	43.00209	38.2492	43.32518	22938.57	32.13769	0.794254	4915.511
70.28506	69.79996	72.9539	83.49675	86.47809	100984.7	53.19181	0.631864	16597.06
89.32101	104.9214	91.75873	85.35651	106.8951	236153.4	65.65746	0.583575	31660.44
80.29661	95.2566	90.25114	86.06021	96.12734	236295.1	70.57373	0.723296	25554.73
31.61933	36.6451	36.17367	34.93597	42.2899	16215.63	27.97615	0.751752	4121.282
37.6332	44.98174	46.74777	44.53339	46.96939	24477.8	35.04367	0.73384	5555.603
101.3561	114.1435	121.513	107.805	125.5046	514407	90.9498	0.746169	41608.76
46.85681	44.57859	49.90422	49.52052	55.47077	28455.53	32.87187	0.734443	6137.2
57.23525	68.43819	74.08495	51.93114	75.15098	59307.26	46.18579	0.664121	11074.11

31.74727	32.2423	38.8348	38.21979	38.96861	15515.64	27.15442	0.75205	4000.218
29.44007	36.73352	34.89626	31.07328	36.84708	14213.37	27.05793	0.77849	3644.986
102.3784	111.075	111.9967	83.94576	113.7438	296294.9	68.67556	0.61618	34881.3
25.40683	29.95987	30.65968	28.74247	31.22134	8009.254	22.43338	0.759506	2548.87
42.13386	45.40461	49.96877	38.85579	51.1798	23456.42	29.99536	0.644966	6144.054
70.7984	82.48285	79.96101	67.72029	83.98375	127320.5	55.99799	0.679264	18018.19
28.83239	36.33117	37.42799	31.89932	37.80289	13183.83	27.25904	0.764112	3532.005
56.57761	62.97585	62.28736	62.18825	70.46629	91841.08	47.99541	0.760289	12947.82
26.82104	30.97712	32.82853	31.83669	33.59955	10437.65	26.21812	0.598742	3857.56
32.73177	39.98332	40.56563	38.41394	42.03651	22615.7	31.36071	0.76277	5070.261
46.16392	54.74031	54.89816	45.33532	55.71679	46229.73	40.00649	0.750521	8299.818
46.23427	51.48482	41.35514	50.1386	51.76953	31278.23	32.06134	0.753125	6374.485
68.97783	57.61834	53.04774	345.1603	353.4612	58062.12	49.33313	0.740736	9789.244
56.56233	60.39442	68.86311	61.6162	73.73122	79387.94	48.00407	0.671354	13305.64
87.16268	122.4618	89.44885	114.6802	122.4716	210412.3	60.24166	0.655631	26093.87
37.4654	45.25392	46.96404	40.11253	47.33397	28020.23	34.10771	0.765386	5828.879
71.16618	74.96486	83.90529	74.11018	84.74183	161665.8	60.46072	0.758519	18920.37
135.0852	121.8282	152.9359	133.3091	155.3235	665060.2	93.42874	0.665883	55334.22
23.62379	28.04624	28.04624	25.21381	28.28955	5870.462	22.07368	0.802041	1962.165
105.4562	41.43319	42.0186	356.5582	356.9566	20939.22	35.20642	0.699847	5249.542
26.20379	31.58815	31.71153	29.45365	32.17963	8635.366	23.66278	0.76705	2653.676
86.06677	51.06919	48.41443	47.16806	347.7925	35765.79	38.75524	0.712626	7366.608
31.94055	39.81651	37.67257	34.76112	41.14051	17328.06	30.44902	0.748589	4325.878
33.69669	39.21176	37.25197	39.62634	41.02991	19686.19	30.02885	0.794966	4435.169
77.11761	89.45855	80.14585	68.21491	91.67834	149530.6	57.09929	0.732572	18597.47
39.73387	47.84758	45.48943	45.39491	49.76696	32006.16	35.73271	0.778636	6260.93
42.24992	53.31606	48.41876	43.83922	53.43573	37871.71	35.88617	0.771871	7065.61
65.23899	73.04158	69.99013	69.81152	75.94302	101698.8	49.96834	0.691621	15234.44
34.12466	39.7091	39.25204	33.34473	40.46754	14030.64	26.38555	0.75673	3717.591
42.10434	50.9876	47.92111	39.0768	53.65628	32057.98	34.37318	0.804887	6063.266
23.40037	22.82575	22.26733	24.08897	28.37743	3390.321	14.69602	0.785576	1389.279
40.4213	45.64616	47.52731	34.63403	48.351	22923.32	29.88466	0.757329	5152.891
66.44484	83.40671	82.82429	147.8401	170.3022	126649.3	57.05503	0.727829	16756.75
73.73231	85.03679	101.8734	75.70603	103.844	191401.7	62.91662	0.731539	21955.52
32.26472	37.92605	35.88311	33.05435	40.17942	14100.88	26.74123	0.775642	3639.041
31.95346	35.23736	32.26765	19.95812	37.16942	5972.721	17.13674	0.735024	2165.863
35.13385	42.75079	40.9062	38.12736	43.42736	22758.93	31.03147	0.794196	4890.173
55.67253	65.02933	68.57722	58.32058	69.46894	84883.54	50.18261	0.748976	12470.92
67.56828	69.60622	78.80021	73.01675	80.85971	131170	56.13975	0.74887	16671.23
49.2673	62.18754	58.67104	48.97153	66.18854	54672.68	41.77409	0.717157	9713.675
46.39881	51.63064	56.67467	53.18712	58.35514	51104.09	43.72112	0.769904	8650.04
31.73089	37.96193	35.3031	29.14686	40.00543	11320.24	23.44617	0.78166	3119.155
57.64113	70.95745	66.84356	57.22362	71.27631	93872.07	49.41783	0.782727	12761.4
23.03123	27.70884	26.91233	20.97624	27.84233	3896.679	17.2639	0.756941	1582.044
45.4909	50.54264	56.82697	40.4375	57.85232	33270.8	34.42981	0.767863	6514.922
58.11935	60.33558	72.50538	112.4663	127.659	46665.27	40.4008	0.691503	9064.678
34.92102	43.09443	40.7565	37.01206	43.653	21689.71	31.38983	0.772208	4870.639
78.37083	91.68377	99.50344	77.79626	99.98917	211964.1	66.88529	0.719308	23900.71
41.8555	51.82132	50.39038	40.76737	51.87091	31945.52	34.27506	0.749258	6498.193
67.01663	78.54499	83.90527	79.47619	89.14957	155851.1	64.65395	0.70942	19741.82
46.80142	53.82467	59.79457	47.63121	59.94321	44236	42.96033	0.762888	7928.791
52.411	60.92362	57.16456	121.4163	129.6692	33960.01	33.21405	0.740654	6847.218
16.05188	21.54721	25.31359	21.51149	26.22488	2005.966	14.47814	0.778657	987.8408
59.1018	55.22735	64.21951	48.63312	66.75126	35859.44	31.87823	0.659486	7974.081
49.39597	66.43634	61.39546	64.05912	66.50229	68542.96	48.92143	0.717371	11290.57
24.74234	25.76448	29.29837	25.2531	30.74181	4511.498	18.01179	0.695142	1899.434
234.1445	209.5321	119.5345	183.1965	231.83	417976.5	96.18547	0.602107	44899.89
45.57195	51.22249	51.75379	44.14965	56.44373	35639.48	35.74841	0.755618	6931.109

70.73085	86.82195	81.9731	80.90832	87.83308	165763.3	63.43087	0.717867	20328.2
65.78168	77.83575	77.83575	46.208	78.13175	50322.72	37.61436	0.666222	9894.064
45.98127	54.16466	55.14202	42.2841	56.12879	41863.46	37.151	0.796907	7316.441
43.85494	48.17019	51.88588	44.71897	53.82671	32430.4	34.47131	0.765357	6425.719
79.32558	85.33085	90.23894	72.13459	90.51246	126557.7	55.28702	0.634567	19210.22
41.32061	46.25218	46.25218	44.53938	51.26627	33192.62	36.5002	0.755357	6612.406
64.39219	57.91637	67.80878	69.38907	73.85352	60918.76	46.43696	0.662574	11300.14
52.61617	62.31996	63.89677	43.96973	65.24677	46124.79	37.59232	0.707442	8791.901
165.1165	199.5341	200.7831	165.2422	202.0707	1826838	136.153	0.679136	106412.8
61.63955	73.22447	57.98117	70.98429	80.31953	65915.39	44.00602	0.658973	11974.97
38.88937	39.43401	45.9733	37.24799	47.19392	18732.13	28.66437	0.746679	4568.163
110.1911	136.7972	139.3284	120.1587	149.5179	690071.9	100.5002	0.707703	53361.63
73.56284	88.7764	83.30104	70.23676	89.67171	130079.8	53.21737	0.66883	18562.73
88.53466	99.37857	101.2103	97.88483	114.3125	353181.6	78.88524	0.762186	31702.16
19.56796	24.75071	24.19518	21.46547	25.73006	3610.09	18.05058	0.790101	1440.39
47.81166	51.14669	57.68769	51.72714	59.3078	48304.7	39.2083	0.731465	8768.999
89.30465	101.7497	104.5345	84.3959	106.081	277190.9	71.39566	0.734597	27986.89
75.81987	76.40268	87.89784	78.99898	90.8389	132124.8	58.86006	0.661991	18950.53
31.90588	40.85048	40.85048	40.53379	42.54366	16722.15	30.59867	0.710492	4450.95
38.43184	44.85389	48.17019	41.07117	48.78717	26749.15	32.59592	0.763762	5663.257
64.19886	84.04696	73.11188	64.99983	84.5037	97538.33	52.15846	0.680221	15064.37
19.24104	24.19518	22.57799	20.43906	25.10768	3314.85	17.52019	0.792351	1356.883
84.25687	93.52258	103.1469	94.69836	103.3947	290148.9	79.94304	0.65554	32331.96
70.00985	84.70768	89.67838	65.59821	90.88584	139780	56.54493	0.733308	17762.11
21.86485	25.51472	24.40515	16.53041	25.874	2546.297	12.92913	0.774616	1164.136
58.2268	73.60631	67.56927	64.91446	74.11538	84900.35	50.54065	0.734673	12715.4
77.65044	92.573	85.9002	79.5262	101.2226	207075.2	65.58771	0.760613	22253.88
49.08781	58.32508	58.03842	56.1983	65.31976	66683.9	48.77966	0.751511	10581.88
54.63606	60.23574	64.79102	65.41708	70.44246	74432.25	50.58507	0.699536	12232.48
17.91327	21.37539	22.33814	19.56305	22.95756	3241.425	16.53711	0.830538	1275.308
47.9178	50.93763	60.19338	55.08577	60.88932	54829.33	41.89903	0.768808	9078.373
49.27527	54.06456	56.37946	44.56055	56.58332	34464.9	35.61312	0.677678	7557.506
39.0671	44.31765	46.15707	44.06635	48.50494	32168.91	34.17086	0.815824	5995.771
41.66944	46.88643	49.299	46.41405	51.64487	38724.48	37.49463	0.778094	7113.921
11.2989	13.98421	13.98421	10.9486	14.30403	630.2228	9.499733	0.806355	440.8463
44.81146	51.58825	43.28787	52.42042	54.35036	23725.94	36.51442	0.703967	5672.15
20.82323	21.39844	23.03394	24.81915	24.81915	2762.846	19.08962	0.728246	1307.506
70.64318	72.6732	75.10708	49.5769	75.14437	76447.67	41.30341	0.704527	12364.11
86.23405	101.2529	96.00294	87.01499	103.4528	258856.2	69.23824	0.70348	27921.47
50.45915	47.51992	48.28267	375.11	375.6997	36672.3	38.30214	0.7971	6696.736
69.10645	71.91201	68.16623	75.414	83.4897	98459.26	48.79414	0.695173	14833
32.72616	37.35054	38.19528	39.90028	40.89113	19477.98	30.17762	0.771654	4536.88
33.17525	41.70939	38.60145	38.45319	44.15989	19326.03	32.69217	0.774268	4498.018
30.62716	37.11356	36.92216	36.35903	37.8328	17195.81	28.46533	0.748218	4305.971
52.2393	63.84621	56.20412	53.68187	64.55088	67417.13	45.36223	0.765227	10468.25
105.062	116.6055	112.292	115.6386	126.1845	518408.5	86.89976	0.758119	41164.99
46.67614	58.86735	56.42284	48.60054	59.40268	49040.2	40.96354	0.709308	9134.489
36.18773	39.9901	33.36754	42.44544	42.44544	13329.58	26.15427	0.722165	3764.657
37.10501	43.77232	42.38524	43.56574	49.91926	26573.03	33.76898	0.753333	5716.426
34.81739	26.76922	35.45796	38.97084	39.53037	8324.081	20.29527	0.69039	2877.049
100.8063	116.0116	118.2108	101.9119	120.2035	417411.1	81.41355	0.687887	39265.36
32.47133	38.36025	33.43686	30.71296	41.03772	8920.246	23.76365	0.705766	2947.189
71.69442	81.73903	84.13182	95.9767	97.47392	152096.6	64.88864	0.662996	20783.54
21.32958	26.85462	24.98352	17.75268	28.0625	2651.403	16.02527	0.712457	1300.297
46.76117	34.05034	40.62099	49.44683	55.17901	18323.01	25.17847	0.712896	4714.72
45.57953	53.06614	55.01026	46.06408	56.57426	41377.84	41.36701	0.753837	7674.528

101.3319	113.99	106.8826	77.82	115.6986	253918	63.8173	0.738034	26274.63
106.1621	122.3012	133.7158	110.3448	134.4034	464462.1	93.72389	0.600076	48333.1
114.8375	122.3015	125.7835	89.33126	131.3307	341390.1	71.81653	0.665648	35487.35
75.95851	87.17422	88.6874	96.15129	100.8073	207352.2	68.35814	0.667485	25381.37
55.87278	66.43841	59.97498	62.47314	71.15091	88059.06	49.66934	0.720909	13277.6
47.0188	56.38262	46.43495	48.41257	56.70782	32011.36	32.10237	0.712764	6840.289
38.82855	45.28798	44.48832	43.49069	51.1672	26226.47	34.77202	0.743627	5740.577
57.182	63.47461	59.30592	64.34207	66.60076	81863.67	47.61852	0.746968	12206.08
25.99774	32.60448	32.60448	28.83591	33.37373	9458.322	24.41543	0.756758	2858.049
39.94625	43.38509	45.26306	35.57615	45.63873	18506.83	28.08265	0.754962	4481.744
30.49833	33.21921	37.04971	34.17969	37.27605	13844.12	26.90427	0.754956	3693.23
35.17653	39.81651	43.13183	38.83337	44.3146	22733.6	30.71479	0.770688	5035.599
16.44553	20.54318	20.27128	17.80914	21.55186	2377.971	14.48274	0.791616	1088.364
28.33949	32.17918	34.5097	32.94206	35.92095	11256.02	24.30662	0.744253	3263.522
48.21691	54.43153	55.13186	55.4359	57.7951	60435.99	42.9976	0.764218	9745.345
23.45088	27.57547	28.4616	24.2211	28.90833	4391.119	20.02277	0.722509	1794.833
35.93524	42.12264	40.86498	31.68942	42.38265	18474.13	27.96854	0.791266	4271.076
81.3145	92.85414	94.8904	89.13949	95.82172	299000.9	74.84208	0.743525	29082.86
53.81328	65.07285	60.74824	55.60547	65.30154	67961.92	46.83517	0.723035	11138.73
51.56358	60.82583	58.94129	53.23699	62.40532	67869.64	45.24809	0.746891	10773.19
72.66913	82.31321	67.30796	61.95235	85.63898	80757.01	43.1242	0.67991	13288.82
96.74669	111.2784	112.8578	90.3645	113.6292	282345.7	70.03445	0.656139	31720.67
81.32125	88.30424	94.27466	68.04352	95.06193	135464.2	54.28998	0.695629	18336.76
9.585451	10.38798	10.89765	10.36596	12.32158	403.595	7.984017	0.840838	314.1008
50.47583	57.78426	59.96198	57.08182	61.66401	48164.97	42.46322	0.67276	9515.793
89.75226	97.36831	101.425	85.91543	105.3489	210385.9	66.58764	0.683236	25037.51
75.72828	86.47562	93.2212	84.63867	93.79849	236924.3	69.24414	0.731137	25325.52
57.21189	60.66657	63.14441	66.55595	71.39793	70797.98	47.42832	0.641795	12895.42
22.83314	26.44971	28.13002	24.79515	29.31265	4999.351	19.48197	0.727966	1942.297
20.74063	22.33092	20.63225	23.16455	24.56086	3129.085	16.08057	0.754764	1370.728
53.69718	48.88228	57.68398	59.15192	61.50008	42126.58	34.25899	0.699904	8365.332
30.56306	37.12837	33.55851	34.24116	40.19264	12016.34	26.00591	0.684536	3706.26

AX13	AX14	AX15	AX16	AX17	AX18	AX19	AX20	AX21
0.215923	22754.42	17	70	48981435	1.898018	28	4.156151	180
0.191014	40840.73	12	66	84365140	1.884816	30	4.057949	145
0.161263	57223.24	64	101	2.22E+08	1.442772	18	4.032406	149
0.467465	2178.854	114	165	25296784	1.782126	25	4.510286	183
0.262327	12628.79	55	141	61625809	2.40798	53	2.251095	186
0.189352	42028.2	8	53	23632986	1.742033	20	5.639411	125
0.233732	23326.42	35	105	45151878	2.274123	35	5.002402	170
0.191047	38682.09	17	78	43327506	2.066163	32	4.038157	152
0.437645	2977.84	14	98	6214245	2.265658	38.75	3.516383	116
0.147026	102841.2	50	177	5.98E+08	3.032047	70	2.851862	330
0.089155	476750.1	15	51	2.52E+08	1.42756	19	6.526662	108
0.074398	811595	43	95	2.45E+09	1.863859	27	5.630261	279
0.161761	65209.77	38	116	2.14E+08	2.33184	41	2.922692	178
0.292801	9960.938	50	197	1.76E+08	3.180609	82	47.11451	260
0.431248	3194.46	77	128	16479844	1.808335	25	4.866255	153
0.108992	256458.5	34	97	5.43E+08	2.060653	34	3.127783	196
0.310381	7821.953	13	123	38716768	2.730774	68	145.1518	160
0.145203	97531.33	14	102	1.72E+08	2.447292	43	5.801227	248
0.189126	89941.93	51	190	6.58E+08	3.185992	79	3.297658	386
0.096743	309972.6	17	134	9.73E+08	2.738512	73	2.023626	258
0.093355	366956.3	18	84	4.79E+08	2.121026	39	3.065516	198
0.141063	105064.2	19	144	3.68E+08	2.90642	76	2.157249	219
0.306543	8402.65	22	59	7916627	1.489429	19	4.689909	86
0.112177	205279.2	25	107	6.74E+08	2.377272	47	3.095727	276
0.147502	81515.06	40	104	2.63E+08	2.093014	32	4.242604	217
0.277735	9783.62	54	136	42485811	2.359134	45	222.3233	193
0.205554	32591.6	54	86	1.03E+08	1.301424	16	5.13141	172
0.184692	49344.62	35	163	3.53E+08	2.990236	70	2.780943	267
0.237603	19696.33	41	102	50456079	2.026798	32	2.894286	162
0.180357	41592.1	18	109	87466131	2.545568	51	2.588392	184
0.31932	9608.373	63	147	47606145	2.315436	34	4.932415	183
0.134002	126345.8	27	114	3.68E+08	2.456858	44	3.443198	255
0.254191	15855.62	44	116	39204133	2.248099	34.25	5.391785	162
0.168903	80159.22	24	142	3.68E+08	2.800044	69	3.194786	282
0.073179	998588.4	20	90	1.74E+09	2.272979	39	7.696219	266
0.297274	12526.88	63	140	69461573	2.25678	44	2.803434	166
0.1008	323536.7	23	135	1.29E+09	2.740405	75	2.151512	304
0.268844	12958.93	14	94	15679478	2.367785	45	3.238174	176
0.233559	21505.01	112	185	2.04E+08	2.252141	37	5.302442	223
0.37736	4290.909	23	98	10433277	2.298821	36	5.98302	128
0.198663	51771.61	39	128	2.03E+08	2.598184	49	3.955599	250
0.08257	495897.1	25	157	2.79E+09	3.08642	68	4.901052	362
0.210484	24233.51	17	160	1.32E+08	3.119073	88	2.336226	257
0.128815	177702.1	43	143	1.09E+09	2.709143	55	3.624403	328
0.157369	154340.4	37	111	3.54E+08	2.277559	41	12.85673	570
0.296811	9809.607	161	250	2.67E+08	2.506642	45	3.634789	296
0.269329	14265.18	57	107	68249616	1.757162	24	3.945161	167
0.161316	72103.78	27	161	5.15E+08	2.957742	78	2.148208	260
0.168754	59152.61	32	129	2.09E+08	2.594757	53	2.503947	197
0.173187	76022.91	40	141	3.37E+08	2.730698	53	3.453648	246
0.142447	143676	35	95	3.29E+08	2.043606	29	4.297944	212
0.283806	9307.489	83	157	63136331	2.208495	40	2.938912	185
0.212391	23724.14	24	128	58937852	2.676509	61	2.347444	187
0.237618	32235.4	89	184	3.55E+08	2.637028	49	3.954346	262
0.118908	202744.7	32	104	5.48E+08	2.265495	42	6.817244	440
0.162893	67908.74	32	100	2E+08	2.211206	34	7.798102	155
0.190288	35291.15	25	80	58026952	1.868829	29	3.041272	136

0.270541	13852.68	33	80	19120268	1.745881	23	4.902122	126
0.274168	10547.75	54	134	44384572	2.357083	40	2.824517	190
0.231768	22927.09	36	112	91095774	2.288832	38	3.743131	219
0.068514	915223.5	33	103	2.77E+09	2.220573	35	4.914526	307
0.292357	9232.637	40	153	59969799	2.724871	67	2.104359	203
0.146114	116390.5	38	99	1.67E+08	2.151694	31	5.26267	214
0.117354	227575.6	24	129	6.17E+08	2.775251	60	3.401571	251
0.11735	278946.7	23	153	8.5E+08	3.015305	78	3.229095	347
0.269299	12656.63	72	170	93813758	2.589486	51	2.816172	212
0.315078	8080.989	54	141	29841236	2.476485	46.5	3.261942	184
0.30147	9102.118	64	188	78765230	3.009643	69	3.070174	268
0.122621	171509.2	10	84	1.98E+08	2.246384	43	2.610414	196
0.075478	851572.5	19	72	7.85E+08	1.89537	28	6.03121	222
0.105257	269288.1	14	159	1.34E+09	3.048017	80	5.497752	453
0.088445	512149.6	27	116	1.4E+09	2.564472	46	7.135529	299
0.107647	261236.3	30	74	4.24E+08	1.652024	23	819.7896	365
0.42555	2977.466	86	137	18565451	1.827215	26	3.48302	169
0.181902	40243.91	43	112	1.22E+08	2.197252	37	3.130681	198
0.13323	314322.6	50	102	8.37E+08	1.879167	24	5.560469	178
0.227112	31591.74	18	84	46698274	2.122962	36	2.794013	148
0.124823	168153.4	22	116	3.99E+08	2.582622	51	4.08823	437
0.19094	42152.83	29	112	1.14E+08	2.354604	49	2.420954	165
0.235725	38200.87	11	112	4.01E+08	2.687178	58	2.81583	226
0.15471	92400.98	25	162	5.71E+08	3.052449	86	2.81568	244
0.318843	9828.687	51	114	1.39E+08	2.137352	32	4.934563	233
0.118072	206758.7	46	142	8.86E+08	2.648539	53	17.25632	743
0.080846	671026.9	31	67	8.35E+08	1.487913	19	12.63706	219
0.140889	93760.47	33	133	2.1E+08	2.625612	60	2.194404	186
0.248497	29850.01	50	141	7.59E+08	2.583556	48	3.255265	295
0.165464	52696.24	19	121	1.25E+08	2.646544	62	2.246739	185
0.308664	7673.734	46	164	42158198	2.853795	66	2.597386	215
0.153344	93399.33	41	170	5.43E+08	3.066013	73	2.798957	319
0.103512	237752.5	13	102	2.72E+08	2.372226	47	3.761478	206
0.159874	71393.41	49	118	6.79E+08	2.290245	35	29.6503	599
0.214741	25742.92	35	150	1.81E+08	2.783881	69	2.168064	212
0.160763	65153.66	29	140	6.64E+08	2.806822	55	4.540268	333
0.284287	9979.192	55	150	76416550	2.587001	53.5	2.603366	259
0.14628	90515.21	62	194	9.15E+08	3.086609	68	4.65344	436
0.479283	2413.61	140	197	64154991	2.037545	30	5.139049	253
0.386344	4646.005	35	97	35112886	2.071036	34	248.3681	141
0.259029	17789.61	4	98	66151539	2.598745	54	2.605824	156
0.182816	44070.59	50	122	3.49E+08	2.26227	37	3.230095	209
0.367762	8035.247	93	171	1.54E+08	2.410022	39	3.410453	239
0.153279	78390.67	47	155	9.16E+08	2.747742	54	2.73777	265
0.153737	84108.07	26	102	1.3E+08	2.324261	43	3.192412	169
0.264138	13421.7	81	198	2.62E+08	2.826227	64	2.546321	259
0.226818	30211.25	25	103	64651386	2.497328	39	10.17105	379
0.159899	59341.33	56	105	1.82E+08	1.864999	24.5	5.927476	202
0.329342	7262.23	76	123	63229053	1.767194	24	4.864059	154
0.21429	23028	63	125	1.02E+08	2.032226	31	3.136968	169
0.164352	101124.9	39	142	1.04E+09	2.647928	52	2.857045	216
0.134067	236318	25	102	1.09E+09	2.364732	41	3.865386	299
0.108148	236513	23	88	3.57E+08	2.130824	35	3.906641	227
0.254155	16275.1	136	228	5.36E+08	2.542696	44	4.819784	273
0.226965	24550.93	105	183	5.24E+08	2.3847	39	4.953666	250
0.080887	514782.4	12	80	3.19E+08	2.061426	40	3.289689	224
0.215677	28538.82	15	81	62675134	2.202672	33	4.19625	157
0.186724	59410.5	59	142	6.65E+08	2.463249	43	3.181094	250

0.257818	15545.14	22	103	1.17E+08	2.419038	44	2.98775	255
0.256448	14266.22	31	121	79053111	2.516217	47	3.299169	208
0.117725	296728.8	48	172	1.87E+09	2.996947	71	4.805214	655
0.318241	8047.481	116	199	2.53E+08	2.436663	44	5.378093	240
0.261935	23574.83	65	140	1.27E+08	2.310965	39	2.930679	204
0.141518	127450.1	55	182	2.04E+09	2.95544	68	2.602942	362
0.267904	13242.22	32	124	77922306	2.526245	56	2.258365	180
0.140981	91988.09	26	67	96727508	1.617913	20	5.032434	121
0.369581	10451.59	91	196	1.15E+09	2.794228	52	3.500614	337
0.224192	22675.5	126	264	9.5E+08	3.084566	80.25	3.077103	341
0.179534	46320.9	63	194	9.24E+08	3.027806	66	3.283338	422
0.203799	31417.75	19	77	39651127	2.006678	31	3.923452	140
0.168599	58160.21	30	110	4.65E+08	2.384702	42	253.7616	352
0.167603	79496.12	55	188	1.44E+09	3.002101	75	2.453953	282
0.124013	210729.9	43	118	5.73E+08	2.288573	36	4.428253	223
0.208024	28105.68	24	163	2.84E+08	3.062429	82	2.196839	234
0.117034	161877.2	12	71	1.21E+08	2.001518	30	3.253256	134
0.083202	665439	31	126	2.18E+09	2.559614	56	3.336448	302
0.334244	5910.832	98	204	1.45E+08	2.716436	56	2.684981	292
0.250704	21018.36	66	168	3.95E+08	2.656805	63	120.2007	214
0.307303	8681.404	17	96	32292247	2.378302	41	3.205306	207
0.205968	35875.31	70	194	6.81E+08	3.021634	67	126.1731	279
0.249646	17383.58	107	269	7.16E+08	3.282897	94	2.490831	410
0.225293	19793.08	98	182	1.27E+08	2.527971	43	4.417106	254
0.124372	149644.1	26	91	5.63E+08	2.088595	36	2.812506	168
0.195616	32083.74	28	197	4.86E+08	3.106971	95	2.090558	239
0.186567	37987.86	20	152	1.14E+08	2.836422	70	2.788395	221
0.1498	101894.4	26	60	77682960	1.409255	17	6.257819	132
0.264962	14153	28	91	17102859	2.052046	29	3.623628	133
0.189134	32225.97	48	141	72051449	2.647609	49	3.880183	318
0.409778	3470.76	68	139	10066964	2.176995	38	3.491613	162
0.224788	23045.46	58	175	1.24E+08	2.936336	66	3.477572	235
0.132308	126903.4	25	101	1.91E+08	2.329927	44	88.0736	208
0.114709	191753.3	35	86	2.15E+08	1.915749	26	6.813778	146
0.258072	14183.9	40	87	25147959	1.719418	25	3.084765	133
0.362626	6053.345	88	135	24693396	1.722779	24	3.515932	165
0.214868	22913.46	34	196	1.03E+08	3.234369	92	2.273379	265
0.146918	85089.66	23	79	77182740	2.005845	30	4.805512	138
0.127096	131440.8	51	108	2.33E+08	2.007171	29	4.190987	212
0.17767	54770.06	18	125	2.94E+08	2.8389	56	4.012577	192
0.169263	51328.26	82	160	2.26E+08	2.368228	43	4.127163	204
0.275538	11439.93	39	100	14065462	2.049731	30	3.468835	154
0.135945	94133.37	12	108	81020553	2.641503	51	2.803089	177
0.405998	3971.146	-8	95	3072740	2.59778	43	2.967949	134
0.195815	33429.56	49	104	62430589	1.963901	27	7.678139	140
0.194249	46865.64	51	150	1.6E+08	2.654843	58	2.67989	209
0.22456	21798.64	50	129	72221277	2.399244	38	5.29576	179
0.112758	212376.6	40	124	4.6E+08	2.367493	49	2.278772	184
0.203415	32115.49	72	162	1.21E+08	2.553265	45	3.912487	222
0.126671	156031.8	56	119	6.76E+08	2.087745	29	4.752227	189
0.179238	44405.64	46	138	1.18E+08	2.567863	54	2.505296	193
0.201626	34128.03	-6	74	19736554	2.368297	38	3.92362	156
0.492451	2072.708	26	88	2163278	2.006784	36	2.37618	121
0.22237	35985.63	53	111	1.38E+08	2.054589	30	8.330903	356
0.164723	68778.23	31	107	99970799	2.349161	39	4.345279	158
0.421021	4583.945	76	138	20250752	2.006217	34	2.508755	175
0.107422	418512.9	19	108	5.41E+08	2.459924	50	34.68347	231
0.194478	35834.88	35	125	64018525	2.576848	45	4.209123	180

0.122634	166076.7	24	88	1.51E+08	2.174569	31	4.254066	198
0.196612	50515.99	17	83	60100513	2.207353	31	4.172858	162
0.174769	42059.37	0	67	17050942	2.102583	29	5.91386	112
0.198139	32592.32	66	152	1.23E+08	2.411552	47	3.05537	184
0.15179	126865.3	27	88	1.35E+08	2.100308	33	4.993604	166
0.199213	33423.59	0	86	15542932	2.468702	46	2.90305	131
0.185495	61131.07	31	141	2.37E+08	2.917942	56	3.917079	292
0.190611	46320.42	33.2	118	87890070	2.473384	41	4.366077	177
0.05825	1827758	22	72	1.76E+09	1.934012	25	171.3155	1362
0.181672	66193.18	31	88	82374040	1.969745	24	7.630223	133
0.243868	18877.92	30	99	27125216	2.206378	33	5.300089	146
0.077328	690452	55	104	2.14E+09	1.825406	23	8.187166	227
0.142703	130327.1	34	138	3.86E+08	2.69644	60	2.748285	247
0.089762	353636.8	12	108	3.33E+08	2.571505	44	6.512952	292
0.39899	3689.346	40	101	5547190	2.025629	37.5	2.399639	130
0.181535	48507.5	48	129	1.16E+08	2.340911	41	3.553982	167
0.100966	277527.7	16	83	2.35E+08	2.252447	36	4.661294	156
0.143429	132576.2	20	83	91871877	2.113976	33	3.901036	169
0.266171	16855.89	25	127	42932309	2.567634	42	4.148707	166
0.211717	26900.71	38	100	40621222	2.072397	30	4.277845	144
0.154446	97713.37	24	132	4.06E+08	2.758798	64	2.52113	226
0.409335	3392.715	52	111	6681341	1.971291	30.5	3.29663	141
0.111432	290483.1	29	78	3.03E+08	1.778134	27	3.867021	192
0.127072	140035.7	25	129	2.67E+08	2.749694	60	3.472098	241
0.457188	2625.458	-51	97	1990457	2.97604	104.75	1.768602	123
0.149768	85149.88	20	90	80622599	2.272916	34	3.740513	160
0.107468	207317	35	113	4.82E+08	2.371863	44	3.242868	181
0.158687	66877.96	15	102	90248894	2.413183	51	2.402827	164
0.164344	74676.82	62	136	2.25E+08	2.334285	36	4.862245	200
0.393441	3285.721	5	103	6234026	2.665095	52	2.9023	169
0.165575	55017.7	20	118	88461721	2.694498	55	3.88858	198
0.219281	34601.87	7	92	65985664	2.380789	43	4.311079	168
0.186384	32303.81	32	139	73669664	2.844208	56	3.593065	262
0.183706	38857.69	-22	100	48411520	2.86213	68	2.784143	169
0.699509	658.73	2.6	99.8	1045369	2.707978	55	3.743039	143
0.23907	23830.61	36	65	34384150	1.307461	14	9.173135	178
0.473246	2815.055	103	171	27522770	2.1463	32	3.955849	211
0.161733	76574.63	35	65	1.19E+08	1.360228	15	9.332336	164
0.107865	259101.5	47	128	1.28E+09	2.369953	43	2.964927	231
0.18261	36784.37	8	35	17356416	1.227141	14	1234.503	86
0.150651	98629.5	28	116	2.39E+08	2.38752	45	3.190727	197
0.232924	19577.69	25	54	13919218	1.164537	15	11.07496	99
0.232744	19440.43	17	52	8897322	1.434221	17	10.04481	86
0.250408	17270.4	40	68	31030266	1.458055	13	8.502316	141
0.155276	67562.38	36	83	1.08E+08	1.881091	21	9.590803	164
0.079406	518721.6	18	63	3.41E+08	1.733273	23	6.042733	200
0.186265	49108.1	24	67	1.67E+08	1.686098	21	5.802909	161
0.282429	13429.53	9	60	8763989	1.895202	25	6.616372	135
0.215121	26677.02	24	62	24778744	1.482064	20	4.124473	131
0.34563	8406.734	53.7	121	31223859	2.136562	34	3.428029	177
0.094069	417852.8	17	57	1.99E+08	1.568788	21	325.4427	161
0.330393	9047.241	23	76	7315796	1.963729	24	7.488924	113
0.136647	152318.9	26	71	1.89E+08	1.778178	22	7.908885	195
0.490418	2706.405	51	82	5741515	1.302765	15	5.955032	136
0.257311	18480.18	36	81	22879242	1.779287	23	4.987575	128
0.185474	41557.98	2	62	25240144	2.027434	22	6.700483	118

0.103477	254225.4	12	46	87230093	1.546063	17	62.85033	205
0.104063	464893.8	28	115	1.25E+09	2.57321	46	7.24976	435
0.10395	341637	25	64	3.68E+08	1.454035	20	4.296273	145
0.122407	207581.9	79	118	1.21E+09	1.727134	18	284.6584	1604
0.150781	88235.66	86	137	4.95E+08	1.861958	25	6.299959	294
0.213683	32125.91	6	43	12833752	1.557207	18	7.296595	86
0.218885	26323.94	39	86	54565403	1.755865	24	5.026696	145
0.149102	82012.92	53	96	2.21E+08	1.690329	22	5.701937	197
0.302173	9517.92	80	133	74387803	1.881024	26	3.683272	234
0.242167	18600.76	-5	65	16169557	1.990421	26	4.799069	97
0.266772	13900.63	53	110	98778496	1.971182	31	3.946863	153
0.221505	22795.95	69	119	2.06E+08	1.799389	25	4.249127	235
0.457686	2402.062	81	131	35621251	1.811154	24	3.687748	168
0.289936	11334.56	40	80	18744860	1.52423	22	2.919504	114
0.161251	60572.11	90	128	4.26E+08	1.469337	19	4.782622	186
0.408742	4447.016	49	87	11308949	1.557519	19	5.936751	154
0.231192	18542.86	58	83	55385142	1.135019	12	11.18406	171
0.097267	299362.9	47	154	1.47E+09	2.674259	65	2.436051	322
0.163897	68100.54	35	91	2.86E+08	2.056878	27	6.862565	177
0.158733	68006.23	53	115	2.27E+08	2.069374	30	3.683466	195
0.164553	80952.67	55	93	1.63E+08	1.569838	19	6.557019	187
0.112347	282676.9	19.01132	114.9908	6.44E+08	2.495633	52.05042	3.28448	270.0346
0.135362	135714.1	56	88	2.39E+08	1.423774	15	8.424911	204
0.778258	441.6943	36	83	432978	1.659988	30	2.307993	102
0.197567	48423.37	42	78	38310281	1.468618	19	12.86141	136
0.119008	210661.6	45.03653	100.963	4.13E+08	1.984295	28.97842	6.69727	175.9932
0.106893	237178.9	42	110	6.1E+08	2.16652	34	5.336572	228
0.182144	71027.52	92	153	4.77E+08	2.11504	27	13.78494	205
0.38851	5074.644	34	95	8385068	2.069712	24.75	6.020119	189
0.43806	3189.045	23	78	3214456	1.964216	27	5.054997	137
0.198576	42248.28	67	114	2.16E+08	1.83821	22	13.96681	152
0.308435	12117.1	59	121	46705754	2.21132	31	9.146527	172

AX22	AX23	AX24	AX25	AX26	AX27	AX28	AX29	AX30
16.93274	41.95222	40	-44	224	11.51084	47.2797	0.530855	50864549
17.68895	36.56574	33	-53	198	12.50471	42.87494	0.890069	75075912
11.55661	82.92665	83	-28	177	7.906413	84.27688	-0.05223	4.06E+08
15.77022	142.0717	145	34	149	10.82422	143.527	-0.9272	44884364
27.73883	101.6057	105	-21	207	21.44048	106.7784	-0.28197	1.44E+08
14.66036	27.71727	25	-67	192	8.951253	34.38286	0.817393	49684949
22.05566	68.83865	69	-111	281	14.80564	74.74437	-0.43441	1.3E+08
19.24709	46.71585	47	-97	249	13.47783	52.76539	-0.28989	1.08E+08
25.68562	64.20451	73	-43	159	17.37685	72.0221	-1.06542	15446598
40.00713	112.651	113	-104	434	29.1471	122.937	-0.06299	1.55E+09
11.5369	32.91533	34	-106	214	8.008779	36.22288	-0.77511	6.26E+08
16.27969	69.20512	69	-62	341	11.12074	72.42443	0.605617	4.26E+09
23.7838	75.57319	74	-48	226	16.96218	81.23269	0.214005	4.3E+08
63.43385	108.6792	128	-1008	1268	34.01912	185.531	-6.33292	3.43E+08
16.14686	103.9045	107	-18	171	10.85277	106.0253	-0.94693	35910097
19.53067	64.69833	64	-93	289	14.03651	69.12641	0.112069	1.23E+09
38.58705	64.77432	71	-1004	1164	27.6085	97.40164	-9.88398	74207483
29.47419	48.77002	36	-76	324	18.82555	62.44073	1.519878	3.8E+08
44.87384	117.5171	114	-82	468	32.32988	130.0117	0.398836	1.52E+09
37.25362	80.48572	87	-76	334	29.70885	91.49619	-0.19576	2.59E+09
21.36454	47.99629	45	-86	284	15.93131	54.58809	0.489129	1.09E+09
39.62909	78.55392	76	-79	298	30.52042	91.50067	0.187688	8.8E+08
12.11983	41.07936	42	-41	127	7.983597	44.10348	-0.82835	16344140
26.126	62.93646	59	-105	381	19.33679	70.57357	0.599656	1.02E+09
19.56811	74.14589	76	-72	289	13.48456	78.34964	-0.43584	5E+08
26.1931	97.39253	102	-1007	1200	19.17816	103.8949	-7.56332	1.06E+08
9.917757	70.69705	72	-18	190	6.992306	71.85453	-0.38873	1.68E+08
40.06748	97.16126	93	-43	310	28.93887	108.9119	0.442167	5.85E+08
18.71258	71.39091	71	-3	165	13.24734	75.15504	0.112157	1.11E+08
28.75423	61.64824	60	-55	239	21.2082	70.89782	0.172202	2.09E+08
25.59746	113.5099	122	-43	226	15.59948	118.6613	-1.34657	1.35E+08
26.30555	68.42022	65	-39	294	18.42875	76.09148	0.559761	7.32E+08
22.23321	82.27508	86	-95	257	15.06601	87.30024	-0.92228	1.21E+08
39.16979	72.5782	60	-64	346	29.03845	86.73279	0.864166	6.03E+08
24.53868	52.18319	45	-81	347	16.32568	61.80927	1.685873	3.81E+09
24.27835	105.5589	110	-26	192	18.00902	109.5963	-0.56638	1.5E+08
37.39219	76.3865	74	-74	378	29.89017	87.84018	0.32776	2.5E+09
25.62687	50.57831	46	-39	215	18.52497	59.70199	0.6483	46189875
23.56434	153.0125	159	-44	267	15.95211	156.0162	-1.16992	5.23E+08
24.0449	63.83978	70	-103	231	15.65157	71.49717	-1.35315	21934462
28.59467	85.06873	87	-113	363	19.97763	92.53516	-0.2556	4.43E+08
41.89281	92.27005	89	-123	485	28.54451	107.5384	0.945641	5.73E+09
46.21712	84.28754	81.5	-50	307	35.27956	100.6197	0.325247	2.45E+08
31.59371	94.51534	95	-96	424	22.54421	102.4711	0.287666	1.87E+09
23.59312	71.94108	69	-115	685	17.17592	78.01308	0.853185	9.39E+08
28.07096	210.4068	217	63	233	19.02657	213.4251	-0.80846	4.47E+08
15.37808	84.6714	87	-16	183	10.33365	86.99996	-0.72274	1.08E+08
41.46706	100.0668	105	-17	277	31.75557	111.5155	-0.14076	8.97E+08
29.75856	80.9168	80	-31	228	21.96636	88.68937	0.150464	4.65E+08
31.5583	91.95225	93	-111	357	22.17524	100.248	-0.00327	7.64E+08
18.77801	62.87884	60	-105	317	12.55077	67.51551	0.418277	6.55E+08
23.2335	124.0912	129	12	173	16.97914	127.3332	-0.63958	1.51E+08
33.14761	71.37501	67	-70	257	25.2177	81.62971	0.259149	1.58E+08
29.75261	138.3612	142	-88	350	20.53531	143.4853	-0.61358	6.64E+08
23.41899	67.5386	66	-99	539	17.40741	73.54354	0.2874	1.1E+09
22.38223	68.85444	74	-126	281	14.46461	75.37787	-1.57872	3.86E+08
16.97048	50.83753	49	-45	181	12.21351	55.07537	0.468641	1.07E+08

14.91716	57.78708	60	-56	182	9.625245	61.16973	-0.93504	51833049
24.12729	92.01904	91	6	184	16.92884	96.88963	0.226781	99018041
23.33964	77.06903	81	-80	299	16.29746	82.58685	-0.53183	1.56E+08
21.67934	68.77879	68	-82	389	15.0044	74.30139	0.679962	5.05E+09
35.44851	100.805	106	-5	208	27.52151	109.114	-0.26999	1.1E+08
20.01138	67.72195	66	-84	298	13.09589	72.89068	0.450725	6.18E+08
34.34809	72.29121	66	-101	352	24.78227	83.88623	0.548444	1.6E+09
42.34296	84.77487	80	-115	462	32.00977	99.07954	0.51312	2.74E+09
30.07936	125.8469	130	-11	223	21.45786	131.2244	-0.49247	2.18E+08
27.75949	101.818	107	-51	235	19.72556	107.4846	-0.66326	93359160
39.97721	129.7613	137	-39	307	28.48877	138.9432	-0.40463	1.76E+08
23.39978	46.25109	45	-104	300	17.64158	54.19955	0.171758	5.04E+08
17.19846	43.54994	40	-121	343	11.59561	49.05296	1.040316	2.05E+09
48.82339	73.88329	55	-38	491	33.85874	96.91287	1.447984	2.53E+09
30.12483	68.28307	61	-104	403	19.36025	79.94733	1.624629	3.27E+09
14.17335	52.89684	53	-1006	1371	9.734972	57.71227	-17.6721	8.7E+08
16.00735	113.6844	117	34	135	10.80507	115.5285	-0.66737	39739756
21.60814	79.68805	81	-38	236	15.41203	84.1417	0.029831	2.85E+08
15.92539	75.9003	76	-105	283	10.40186	78.8789	-0.13461	1.96E+09
20.61233	51.16904	52	-49	197	15.14268	57.08968	-0.00636	1.03E+08
29.95197	64.26091	58	-95	532	21.35169	74.4368	0.769789	9.32E+08
26.36008	73.21379	76	-70	235	20.54837	79.59052	-0.24564	2.67E+08
32.34719	57.93273	53	-131	357	23.69241	70.07978	0.416218	1.88E+08
44.70943	96.53281	102	-124	368	34.55787	110.25	-0.35597	1.12E+09
20.11833	83.11123	85	-100	333	13.5484	87.21121	-0.63437	74754978
30.59735	94.39301	93	-27	770	22.02455	102.3384	1.443634	2.17E+09
11.88078	49.92465	49	-60	279	7.846753	52.70286	1.570343	1.86E+09
31.50248	83.18214	83	-45	231	23.86255	91.27289	0.014806	7.81E+08
28.27697	98.05909	100	-50	345	19.87526	104.3636	-0.03467	3.25E+08
32.77894	66.6618	64	-70	255	25.38003	77.08799	0.234993	3.13E+08
37.387	111.2385	117	-41	256	27.66861	120.0955	-0.49994	1.11E+08
40.99151	105.0832	105	-82	401	29.79871	116.5373	0.065821	1.27E+09
28.69235	47.99454	36	-91	297	20.10818	59.86438	1.104459	8.52E+08
22.81773	85.41184	84	-32	631	14.65341	91.96561	2.968616	6.04E+08
36.51459	88.82138	84	-46	258	27.99629	98.74474	0.25216	2.51E+08
34.96165	78.58136	71	-83	416	23.43245	90.82094	1.047004	5.37E+08
29.66941	102.8017	104	-30	289	22.17016	108.9393	-0.07365	1.18E+08
42.06242	124.026	117	-50	486	28.53225	135.5148	0.983059	1.66E+09
18.81683	167.6826	168	14	239	12.91256	169.4625	-0.41025	69312960
22.5412	63.18239	67	-1024	1165	13.7974	88.59012	-14.5044	36462817
29.87422	50.443	51	-86	242	22.13656	62.14105	-0.04926	68694765
22.34394	86.24122	86	-51	260	15.69983	90.74557	0.14832	3.63E+08
24.47457	134.1964	136	8	231	16.58744	137.8202	-0.35466	1.53E+08
32.476	105.4728	109	-42	307	22.93058	113.0084	-0.18708	1E+09
24.34622	66.70377	70	-70	239	17.5525	73.18939	-0.45097	4.51E+08
36.47247	145.1072	149	-3	262	26.81052	151.7072	-0.4318	3.09E+08
25.97212	62.53572	61	-105	484	16.39482	72.52922	0.509647	1.59E+08
16.00825	80.44152	82	-74	276	10.22574	83.35714	-0.6923	4.12E+08
15.30982	99.86031	102	-13	167	10.19477	101.8775	-0.87325	75374905
18.84552	95.16011	97	4	165	13.17428	98.10058	-0.32577	2.22E+08
30.90079	96.03382	101	-96	312	22.10752	103.4303	-0.43664	1.08E+09
24.29568	62.14317	60	-85	384	17.1298	69.3508	0.442212	1.14E+09
20.72981	53.88613	52	-69	296	14.70814	59.89894	0.61322	8.49E+08
28.92534	186.8279	194	-68	341	18.69643	190.7237	-1.16387	5.92E+08
24.40461	145.3414	148	-39	289	16.45965	148.8088	-0.76941	5.44E+08
22.42455	40.20106	32	-67	291	16.76369	48.3948	0.849506	1.21E+09
20.99895	47.59747	49	-94	251	14.0296	54.9868	-0.41143	86288515
26.06304	102.6894	105	-45	295	18.12975	107.828	-0.28433	6.91E+08

25.54643	62.37332	62	-92	347	18.17439	69.995	0.169692	76160276
28.03099	72.01798	67	-22	230	19.64202	80.19191	0.676764	91742367
39.78932	111.0233	111	-119	774	29.29601	121.4368	0.379693	4.38E+09
26.40855	160.0917	165	-89	329	18.40228	163.6794	-1.0231	2.16E+08
23.26556	103.0076	103	-2	206	16.37618	107.0569	-0.08567	2.7E+08
38.77182	115.0412	111	-75	437	28.35701	124.4312	0.282025	1.97E+09
29.51308	77.80813	78	-14	194	22.68786	85.31739	0.089621	96390904
13.22445	46.33543	47	-49	170	8.85529	49.56696	-0.61303	2.26E+08
32.54172	146.4951	149	-21	358	22.08363	152.3635	-0.1082	2.43E+08
45.18388	201.1291	212	-18	359	33.06871	208.5506	-0.71797	9.86E+08
39.77111	134.9103	140	-118	540	27.93784	143.8956	-0.24936	9.59E+08
18.47603	48.24876	49	-64	204	12.51474	53.83748	-0.55771	91063548
27.06473	72.16591	78	-1015	1367	17.86005	91.12197	-13.1247	4.83E+08
41.65989	127.8022	135	-49	331	30.96149	137.3308	-0.42151	1.5E+09
23.60734	84.72757	90	-98	321	15.47033	90.22064	-0.97364	1.72E+09
43.67815	97.63804	103	-70	304	33.23522	110.6676	-0.17573	3.44E+08
18.41779	40.40094	39	-79	213	12.9516	46.61386	0.168568	3.52E+08
31.05701	73.53847	67	-57	359	23.03638	82.71913	0.739406	4.55E+09
32.22222	154.3695	158	30	262	23.41537	159.3803	-0.24812	1.5E+08
39.92726	114.452	126	-1024	1238	25.64573	148.5417	-9.97514	4.64E+08
24.79174	53.79653	50	-57	264	17.47427	62.1507	0.442828	33533737
42.69967	127.7596	132	-1024	1303	27.60255	152.814	-9.16218	8.38E+08
51.57497	197.2498	208	-10	420	38.47723	206.7451	-0.37078	7.43E+08
26.92427	136.9871	136	-49	303	18.20257	141.4418	-0.32054	3.96E+08
20.4268	57.27681	55	-84	252	15.00117	62.50658	0.28418	5.85E+08
50.77522	126.7465	138	-9	248	39.41213	140.2649	-0.48073	6.31E+08
41.05028	71.76372	58	-38	259	29.86865	87.44636	0.838222	2.9E+08
10.95733	43.00019	43	-80	212	7.321171	45.46153	-0.53697	2.11E+08
19.12706	62.21037	65	-40	173	12.95994	66.93808	-0.52372	63415447
29.70752	94.38149	95	-61	379	20.7391	101.6728	-0.18825	3.33E+08
22.44336	106.5901	111	5	157	15.74751	110.3307	-0.79144	42249108
37.51937	118.3028	122	-163	398	26.4704	127.263	-0.50898	3.73E+08
24.55838	63.70165	65	-735	943	17.90633	71.88554	-4.27399	6.56E+08
17.10416	59.94205	62	-89	235	10.95817	64.45878	-1.22676	7.97E+08
14.82408	63.5251	64	-4	137	10.38912	66.202	-0.08236	62163839
14.70432	113.9503	117	33	132	10.31032	115.4702	-0.65668	80711469
50.11532	105.5401	99	-49	314	37.91273	121.4053	0.343584	3.38E+08
18.46866	50.3681	51	-65	203	12.62859	55.89998	-0.59579	2.66E+08
18.23633	77.44791	77	-81	293	12.36238	81.00703	-0.0964	8.63E+08
34.68836	73.2421	78	-119	311	23.45597	85.8187	-0.77204	4.03E+08
25.17197	123.698	128	-32	236	17.59077	127.772	-0.84123	8.38E+08
18.82651	70.37525	71	-24	178	12.7196	74.45893	-0.25503	63424457
30.18195	56.27433	53	-65	242	21.45206	67.6338	0.084158	4.31E+08
29.04635	38.0112	37	-67	201	19.22584	53.5634	-0.00818	11393369
18.19889	76.1487	79	-72	212	11.7265	80.20904	-1.47681	2.15E+08
31.72076	101.1601	103	-77	286	23.87054	108.1867	-0.30062	5.49E+08
25.46447	93.44196	99	-77	256	16.00932	99.54705	-1.21002	2.16E+08
26.63887	83.79604	85	-42	226	20.53562	89.58958	0.063269	1.7E+09
28.83017	121.3151	128	-17	239	19.19836	126.9063	-0.88444	5.17E+08
19.41771	91.24711	95	-63	252	12.40617	94.88601	-0.83788	1.4E+09
29.75516	92.73764	93	-38	231	22.26505	99.39312	-0.16563	4.39E+08
24.54465	37.81864	42	-94	250	16.19982	49.65099	-0.76006	84133122
19.98715	57.48479	59	0	121	15.27761	62.20858	0.002105	8021188
18.82519	82.02924	83	-90	446	12.73627	85.82724	-0.31845	2.65E+08
24.27378	70.62518	74	-108	266	16.55555	77.28776	-0.81653	4.11E+08
19.14771	107.2271	107	41	134	14.10021	109.7255	0.042739	55189258
28.47014	56.23723	48	-971	1202	20.92477	66.51317	-0.45996	1.85E+09
28.54223	81.72505	86	-93	273	19.25242	89.75347	-0.81826	2.89E+08

20.20455	54.5654	54	-70	268	13.03075	60.91677	-0.29003	6.16E+08
20.95015	52.69422	55	-79	241	13.58596	59.59195	-0.49789	1.79E+08
21.12114	37.61371	44	-115	227	12.99589	47.49436	-1.48619	94873905
27.30489	113.5816	120	-22	206	19.96614	118.3857	-0.6961	4.57E+08
19.79949	57.15695	59	-138	304	13.59535	62.71549	-0.74685	4.99E+08
26.75754	44.05124	45	-80	211	19.25734	55.13515	-0.38021	1.02E+08
35.06087	87.58052	89	-89	381	23.28308	98.85909	-0.01854	5.97E+08
26.77305	79.82142	85	-85	262	17.18788	87.37268	-0.97951	3.54E+08
17.75365	48.82122	47	-108	1470	10.76816	58.93729	8.494117	6.35E+09
18.75943	62.24277	68	-102	235	10.71889	67.92846	-1.7894	3.05E+08
22.12642	67.45823	73	-83	229	14.01441	73.80265	-1.22492	1.03E+08
15.72567	80.99881	83	-123	350	9.928631	83.94196	-1.22677	4.87E+09
32.6962	84.27816	82	-47	294	24.57342	93.17908	0.340139	1.13E+09
32.00054	47.9638	34	-102	394	19.73993	64.64709	1.537029	1.48E+09
19.74104	70.83819	72	-11	141	15.05456	74.66636	-0.11905	20568347
24.73026	91.86824	96	-67	234	17.25768	97.07976	-0.73552	4.57E+08
22.06709	48.47127	50	-104	260	14.81055	56.52952	-0.70653	8.87E+08
19.77963	51.18643	51.5	-94	263	13.69991	57.167	-0.22975	4.33E+08
29.31295	82.4043	88	-61	227	18.58475	91.24174	-1.02491	1.4E+08
19.31759	70.41048	72	-48	192	12.90889	74.82691	-0.66884	1.51E+08
34.54611	76.69388	75	-52	278	26.08223	87.23184	0.301378	7.44E+08
18.2379	82.26339	84	-6	147	12.61941	85.45181	-0.51182	24773649
15.53711	54.06443	54	-59	251	11.0354	57.52415	0.145103	9.61E+08
34.07189	72.39958	67	-79	320	24.27933	84.00437	0.725013	9.88E+08
50.54369	30.6519	45	-89	212	41.81946	64.80182	-0.36371	11025023
21.90711	53.72111	53	-71	231	14.47886	60.89738	-0.17255	3.16E+08
25.14807	71.3463	69	-85	266	18.21427	77.85076	0.051983	1.26E+09
27.79993	53.90809	49	-47	211	21.18653	63.26971	0.440696	2.68E+08
23.79987	100.9424	105	-64	264	15.52661	105.7988	-0.99955	8.36E+08
30.25576	52.16411	52	-59	228	21.31665	64.48868	0.190827	13664622
32.22734	71.44777	78	-110	308	22.57347	82.30749	-0.80589	3.73E+08
27.46337	56.88829	66	-106	274	18.47207	66.94176	-1.17603	1.55E+08
34.21978	82.86204	80	-81	343	23.73349	93.64376	0.241077	2.83E+08
38.93092	40.02884	37	-102	271	28.30412	62.27766	-0.36111	1.51E+08
33.26232	52.27897	61	-94	237	22.19547	66.98181	-0.74726	2955433
9.707869	49.9104	48	-69	247	6.052804	51.75844	1.353826	63840689
21.05443	140.6796	145	40	171	13.79876	143.3692	-0.88082	57862659
9.937469	50.38069	50	-78	242	6.453076	52.2582	0.382547	2.09E+08
24.988	90.99859	94	-62	293	18.0359	96.14056	-0.28534	2.39E+09
9.302071	21.30393	22	-1003	1089	5.938932	33.67765	-31.5215	41720244
27.59393	64.08705	56	-72	269	19.62846	72.67736	0.789189	5.21E+08
9.172145	39.53772	40	-81	180	6.298053	41.45641	-1.04678	33646889
11.58424	34.19568	36	-109	195	7.262063	38.02649	-1.51756	28111124
10.2908	53.48549	54	-41	182	5.812826	55.72154	-0.41446	53622687
16.33714	58.40722	60	-116	280	9.134489	63.4698	-1.45818	2.72E+08
14.5866	40.5048	41	-110	310	9.845876	44.87946	-0.25855	1.04E+09
13.78733	45.6631	46	-57	218	9.116779	49.33666	0.17608	1.2E+08
16.51688	33.87909	35	-108	243	10.66331	40.72194	-0.56404	22269868
12.04638	42.50136	42	-8	139	8.343275	45.25483	0.496258	54634529
20.65725	89.37493	92	-28	205	14.37876	93.1564	-0.47185	72954611
13.09597	35.70694	35	-715	876	8.728352	41.98577	-11.3823	7.37E+08
17.28066	49.02459	50	-102	215	10.67064	54.75653	-1.20752	27126136
14.95451	48.35703	50	-106	301	9.409074	52.78394	-1.1225	4.24E+08
9.756612	67.15114	68	16	120	6.258661	68.48927	0.200977	12695149
15.09373	59.34455	61	-40	168	9.866262	62.6719	-0.69986	72585842
19.32655	38.54943	46	-100	218	10.50327	47.45499	-1.67424	93587570

14.90078	27.7623	33	-656	861	7.10376	39.07382	-4.77652	3.88E+08
27.96485	70.03483	68	-121	556	19.17148	79.16937	0.632451	2.91E+09
12.05591	43.49197	42	-98	243	8.464809	46.1574	0.43867	7.28E+08
17.25979	101.9456	98	-67	1671	8.10285	116.8146	14.74106	2.83E+09
16.19448	112.4181	114	-60	354	10.74045	114.5134	-0.56205	1.16E+09
12.22474	24.7533	26	-89	175	7.552952	30.11503	-1.24397	29135462
14.77207	62.04982	62	-71	216	10.13173	65.01986	-0.35604	1.11E+08
13.90974	75.07269	74	-31	228	9.134028	77.37671	0.651378	4.91E+08
16.36382	106.8123	107	39	195	11.13531	108.879	0.156602	1.13E+08
20.97029	39.0294	48	-87	184	12.51558	48.15821	-1.45316	43139133
18.2838	82.71373	85	-43	196	12.87799	85.9025	-0.65203	1.03E+08
15.63214	94.79879	96	2	233	10.79027	96.91171	-0.2485	2.14E+08
15.58306	107.2959	109	31	137	10.36277	109.2036	-0.5649	28645619
12.58402	59.77746	60	7	107	8.989721	61.80635	0.000386	43298293
11.93983	109.4691	111	6	180	8.164653	110.5804	-0.61729	7.41E+08
12.19938	69.38811	70	-12	166	7.946459	71.35704	-0.25059	22643437
8.39892	70.5673	69	7	164	5.306132	71.6052	1.439259	95074897
34.21077	105.2971	110	-63	385	26.71128	112.7611	-0.20235	3.81E+09
18.88801	63.51716	66	-121	298	11.53889	68.86261	-1.21336	3.23E+08
19.22274	84.82904	87	-32	227	13.10541	88.38987	-0.4584	5.31E+08
12.39489	74.42805	75	-59	246	8.099311	76.33185	-0.51266	4.72E+08
30.83218	57.64729	45.95941	-88.8733	358.9079	22.17443	68.97685	0.889213	1.34E+09
10.36249	72.46287	74	-69	273	6.611427	73.91462	-0.79357	7.41E+08
15.78024	58.73684	57	7	95	12.2949	61.62834	-0.03191	1677578
11.87244	59.64828	61	-98	234	7.668881	61.98524	-1.66611	1.86E+08
18.18171	73.73482	76.04529	-99.9479	275.941	12.19373	77.65222	-1.12477	1.27E+09
21.37472	79.41557	84	-120	348	14.47742	84.13346	-0.9451	1.68E+09
21.45429	124.0918	130	-120	325	11.75216	128.5038	-2.62331	1.17E+09
18.93006	69.88755	74	-59	248	11.27217	74.91659	-0.75965	28481416
17.45921	47.90603	46	-61	198	11.42021	53.38253	-0.189	9087806
17.11336	91.05453	96	-108	260	9.511298	94.88089	-2.67455	3.8E+08
21.76286	89.49518	94	-102	274	12.92571	94.9006	-1.82501	1.09E+08

AX31	AX32	AX33	AX34	AX35	AX36	AX37	AX38	AX39
0.323648	475.3818	17.85477	19.40533	1.638916	2.195463	1.012466	0.365047	0.691834
0.313672	501.2075	24.16896	24.50029	3.325202	2.585545	0.554522	0.633412	0.449681
0.450101	225.7636	34.6133	5.028818	-0.04542	1.155799	0.517139	0.378508	0.443189
0.341439	415.63	28.84268	9.580169	-1.19499	1.593802	1.001567	0.218905	0.676666
0.207521	1077.904	32.7166	75.09874	-2.94015	5.667314	1.44827	0.59294	0.845762
0.368166	413.9341	21.3784	16.29947	2.040959	1.792936	0.674913	0.429139	0.521063
0.252687	847.9606	69.49553	56.37632	-0.34362	3.624722	1.588407	0.386641	0.920788
0.28139	601.8159	41.47029	25.5036	0.225152	2.771435	1.044105	0.448325	0.701164
0.259519	1064.964	27.64581	57.62118	-6.91462	3.955765	2.214696	0.274153	1.000237
0.139638	2423.279	104.5557	364.5501	0.728017	11.43628	3.689659	0.509256	1.389689
0.457367	228.6777	47.14856	5.694323	-0.42719	1.119531	0.510267	0.36908	0.442642
0.330346	455.9493	40.00001	30.26262	1.932665	2.543389	0.667819	0.583492	0.513484
0.229782	887.4437	31.9805	68.93013	3.200709	4.946169	1.115301	0.634028	0.720977
0.123038	22610.58	2168.436	268583.9	-2986.05	51.40715	3.091001	0.724921	1.277577
0.347349	445.2214	32.66279	13.68647	-1.67833	1.840401	0.930169	0.322392	0.661839
0.277032	592.5864	51.46759	30.44425	1.086066	3.255374	0.777273	0.614431	0.583701
0.163293	5291.366	1954.1	32373.65	-377.955	13.85053	1.551631	0.726141	0.891893
0.236327	1520.33	43.26514	475.7843	42.99326	8.774636	1.21457	0.755883	0.710917
0.12715	3092.776	89.9657	729.4899	21.51844	15.53975	3.680409	0.611639	1.359633
0.163196	1893.602	62.50773	254.0289	-9.15284	11.24443	1.490892	0.767326	0.82526
0.260144	676.2167	41.73644	43.81917	3.857462	3.921227	0.693435	0.698416	0.54569
0.143934	2201.654	60.88618	336.2777	8.470698	12.56053	2.066011	0.718252	1.001246
0.430287	257.6033	17.7011	5.173051	-0.45467	1.146144	0.518195	0.367172	0.436651
0.220914	1019.631	65.14645	99.21031	7.783975	5.732757	1.089292	0.680496	0.686976
0.282339	641.0536	42.61805	35.92357	-1.35988	3.215754	0.935112	0.545953	0.625931
0.222001	1308.854	2061.497	74.37538	-5.12769	5.314969	1.407707	0.585704	0.835984
0.455337	165.0013	18.93882	3.367253	-0.04618	0.971635	0.419647	0.392011	0.387369
0.141586	2421.5	43.21903	566.858	26.04134	14.04166	2.734738	0.679163	1.156548
0.285398	551.6173	19.36385	27.31354	0.369818	3.006256	0.883843	0.546031	0.627543
0.192811	1225.995	37.41241	101.3702	3.518715	6.424896	1.746615	0.573989	0.925315
0.258076	1195.993	52.4416	98.88633	-10.5422	4.206067	2.253476	0.272937	1.018533
0.213621	1108.587	28.27703	124.3076	7.846888	6.10252	1.137579	0.683661	0.717744
0.254539	852.1438	62.5133	65.46782	-4.99592	3.806465	1.392512	0.45638	0.832525
0.177799	2254.983	42.36216	465.95	36.98712	11.84709	2.477559	0.650874	1.025096
0.25889	1097.301	44.81448	307.3682	26.8947	6.399687	0.796018	0.777471	0.559885
0.237363	868.6619	49.87091	44.54717	-4.8057	3.714761	1.138622	0.500734	0.744005
0.162791	1881	44.54581	267.825	11.93536	11.17406	1.224292	0.802439	0.735404
0.222949	1006.162	20.84345	83.31057	7.724739	5.047539	1.202556	0.610281	0.766008
0.253558	928.21	77.74032	63.9077	-6.52589	3.763945	1.698813	0.369714	0.898123
0.251379	1036.328	69.10664	125.8961	-11.7739	4.347423	1.500115	0.474167	0.850348
0.197046	1326.067	81.60889	127.3352	-1.21112	5.91434	2.173163	0.453546	1.009858
0.140197	3050.756	88.96454	1411.455	61.4977	17.11533	3.009732	0.702276	1.132573
0.127128	3019.935	38.72928	548.9126	12.25415	15.61316	3.632132	0.621777	1.333134
0.176534	1567.168	69.86504	260.5329	7.76976	8.7101	1.700526	0.673967	0.869799
0.239485	910.5215	71.80084	129.5055	4.73875	4.778725	0.835518	0.696732	0.605607
0.211802	1279.262	51.86317	88.27798	-6.69746	4.877987	2.136747	0.368545	0.982324
0.362165	399.748	24.74066	12.5433	-1.44502	1.808119	0.734605	0.413457	0.545241
0.138847	2422.361	32.48702	405.9819	-6.22222	13.61715	2.616983	0.67955	1.149584
0.184509	1318.275	34.34515	130.2954	2.59824	7.165552	1.903455	0.584386	0.986438
0.178117	1594.45	87.19143	244.0087	1.022721	8.784965	1.444718	0.716539	0.823073
0.297103	604.5961	65.88848	39.58999	3.231178	3.207822	0.818473	0.591345	0.588559
0.245754	815.1296	32.02732	36.57658	-3.76105	3.507262	1.634633	0.361364	0.876719
0.174993	1569.016	43.18762	158.4561	7.412293	8.370553	2.285011	0.575385	1.062839
0.190589	1444.2	105.3529	162.2477	-8.84378	6.75717	1.951089	0.544651	0.973679
0.238533	847.1898	54.13288	91.5186	2.472648	4.305321	0.988812	0.620374	0.633476
0.272738	940.8899	88.46326	108.9849	-9.0399	3.845634	1.301195	0.468045	0.742178
0.31988	448.8412	20.98818	17.69659	1.630116	2.449035	0.692452	0.557066	0.545045

0.381723	402.3883	34.96248	13.55393	-1.46905	1.731565	0.83733	0.330846	0.597445
0.224825	920.0969	18.91367	72.88903	4.371654	5.097834	1.089448	0.649089	0.721761
0.245127	880.9523	59.34949	54.84804	-3.42104	4.077661	1.445064	0.473231	0.81123
0.254971	790.1737	53.49963	85.36666	4.632316	4.480949	0.872973	0.673714	0.588672
0.163653	1744.2	31.00111	202.3326	-6.21945	9.783734	2.472911	0.603018	1.162619
0.281972	726.7892	53.43332	60.43183	4.103356	3.505561	1.162049	0.495751	0.725878
0.172321	1810.88	73.61266	308.0437	16.54994	9.961755	1.662718	0.712579	0.87014
0.139285	2629.976	82.94035	633.0392	24.66315	14.86029	2.421034	0.720359	1.065861
0.19019	1382.406	46.02493	109.2398	-7.22348	6.090558	2.416369	0.427819	1.09177
0.208603	1186.023	59.9912	96.02433	-8.00543	5.584511	2.014412	0.471397	1.027339
0.147686	2467.215	62.13996	400.4938	-13.4453	11.75902	2.300055	0.663566	1.086923
0.236318	798.4282	54.48236	46.56883	1.954609	4.344499	1.014523	0.620813	0.696979
0.326755	509.5956	53.20992	43.12808	4.593294	2.802597	0.768604	0.569074	0.576016
0.152798	3933.365	34.36403	2680.919	149.2567	22.79922	2.388903	0.808999	0.993944
0.214083	1728.998	70.0567	645.3056	47.58756	9.782924	1.474336	0.737162	0.745899
0.379296	532.6304	1906.431	2125.563	-24.4467	2.076875	0.605823	0.516292	0.488214
0.333271	422.6948	17.33783	12.09434	-1.11107	1.926656	0.971966	0.330245	0.671812
0.252695	729.6408	33.07391	42.43598	0.443952	3.769635	1.175371	0.524614	0.731152
0.330755	461.0246	73.58993	29.42299	-0.16254	2.490844	0.592475	0.612019	0.488227
0.262331	640.9612	21.006	28.37204	0.86597	3.255649	1.008267	0.524402	0.701115
0.197132	1411.374	50.99838	190.0594	14.12931	7.217696	1.898134	0.581877	0.946818
0.215025	974.3911	44.04994	56.44778	-2.56998	4.80671	1.234837	0.580706	0.774962
0.177577	1554.975	79.83468	215.7774	13.08155	8.852832	1.302357	0.743324	0.826529
0.132347	2836.478	91.89338	546.5738	-12.5967	14.95481	2.402623	0.7175	1.080842
0.277235	698.3183	62.64614	42.93928	-2.9901	3.176922	1.183448	0.455653	0.783757
0.183755	1563.099	41.25948	615.2307	15.98803	8.23341	1.586141	0.671389	0.87453
0.42091	285.1205	30.80918	25.39701	2.576722	1.535083	0.590567	0.442495	0.473907
0.176137	1411.472	35.99833	137.0937	-0.08741	7.930742	1.633487	0.660064	0.92192
0.197	1276.184	43.58264	145.4017	-0.89437	6.873311	1.289916	0.68342	0.81687
0.174504	1498.762	39.9453	148.6384	6.085057	8.255478	1.678237	0.663127	0.884176
0.156321	2048.927	50.80955	251.3338	-8.65923	9.76555	2.68032	0.56169	1.165786
0.136241	2538.482	80.31323	475.7023	6.373777	12.99962	3.202996	0.604219	1.278384
0.232597	1280.268	41.96908	193.0468	21.20995	6.96984	1.485542	0.647126	0.819253
0.255524	1162.49	36.56204	752.3848	32.09895	5.914162	1.913248	0.5121	0.797287
0.158319	1861.286	38.61756	224.4068	7.620253	10.19446	1.847573	0.692023	0.95274
0.174643	2073.414	60.23032	508.8622	36.21896	10.45101	2.553383	0.601707	1.082458
0.186464	1299.582	47.52324	112.9767	-2.71878	6.604699	1.748463	0.579357	0.955242
0.14136	2981.805	57.14529	1098.521	58.91982	15.48163	3.342043	0.64153	1.228121
0.293368	600.0779	53.83115	31.52941	-1.46292	2.583665	1.140027	0.381416	0.75144
0.274875	3856.195	1952.744	29027.23	-343.9	6.736388	1.145401	0.446779	0.767529
0.185553	1317.015	45.61377	127.5264	-0.28685	7.469652	1.345696	0.696287	0.809827
0.24453	797.2119	49.7078	50.36563	1.442528	3.998332	1.315797	0.503814	0.780593
0.225516	985.7141	35.23718	75.3465	-1.75923	4.69916	2.015845	0.406066	1.005
0.172248	1646.389	47.4546	231.7092	-7.99257	8.907382	1.86962	0.652126	0.952531
0.231995	907.2927	39.34879	62.58936	-2.8766	4.707467	0.966505	0.656023	0.672395
0.158627	1958.98	56.20356	229.0908	-12.455	9.220481	3.328765	0.470254	1.310116
0.226798	1349.771	64.82761	247.5161	4.219885	5.170217	1.760901	0.463137	0.912415
0.345905	477.5733	46.15546	19.56375	-0.85763	2.010803	0.975787	0.333458	0.631148
0.355418	406.9491	31.17537	13.5523	-1.57563	1.707406	0.944563	0.280019	0.654146
0.285464	568.2785	19.10663	26.00909	-1.22807	2.868927	0.981708	0.487996	0.678903
0.184358	1475.339	72.6969	188.1883	-12.1051	7.946628	1.546506	0.67182	0.87245
0.228919	947.7599	50.23216	86.54108	4.075145	4.941088	1.282838	0.584869	0.774947
0.26832	684.1672	32.90019	50.44377	3.88369	3.767758	0.942134	0.600426	0.644497
0.213376	1470.839	122.5455	234.0004	-20.4071	7.270785	2.296801	0.524218	1.045496
0.231848	1019.944	71.38637	109.144	-7.58529	4.74591	1.527914	0.50408	0.836925
0.272794	725.931	26.40421	55.09679	7.231964	4.190093	0.734195	0.700578	0.548824
0.265064	758.029	42.44775	41.34822	-1.32382	3.276801	1.334358	0.408846	0.804778
0.213605	1081.754	45.23552	101.9888	-3.9587	5.710363	1.420763	0.600815	0.842626

0.215331	1008.869	50.31463	84.14913	2.306418	5.442488	1.368787	0.598358	0.857733
0.205294	1244.153	21.05893	149.7634	10.28169	6.964204	2.018649	0.559469	0.975338
0.143013	2420.741	104.0397	738.893	16.7288	13.19388	2.182459	0.715041	0.936276
0.220413	1161.613	124.8441	116.0709	-9.13222	4.816818	1.826663	0.430977	0.929899
0.234206	850.6328	32.73617	47.19133	-0.52763	4.001169	1.876336	0.364091	1.0145
0.14537	2248.665	68.09636	403.13	11.37215	12.61159	2.063297	0.718807	0.970503
0.190658	1224.952	20.59739	82.42534	2.14817	5.757322	2.347491	0.417358	1.108679
0.388941	309.9113	19.45452	8.495512	-0.67954	1.401122	0.705721	0.322989	0.563533
0.174387	1753.822	57.19666	370.2991	-3.88708	10.24998	1.391386	0.761142	0.818041
0.136811	3040.474	99.85804	622.8893	-37.673	13.38896	4.093225	0.515062	1.369366
0.143674	2505.148	122.5973	551.0443	-14.5502	13.1587	2.881503	0.638798	1.191817
0.296447	570.5316	30.85072	21.66878	-1.11119	2.490375	1.105542	0.379026	0.719836
0.226069	3095.295	1976.337	26677.4	-310.641	9.073727	1.163081	0.673338	0.726381
0.14003	2526.338	62.95471	409.0554	-17.1782	12.91112	2.178011	0.701794	1.020132
0.252395	961.0042	64.3349	119.7444	-11.9829	5.252069	1.005747	0.678135	0.667301
0.130859	2714.133	60.21185	475.1105	-15.4221	14.66135	3.168298	0.646924	1.233297
0.293734	540.6161	37.93361	22.94656	1.348658	2.716333	0.827934	0.529728	0.634274
0.198335	1434.548	43.22144	228.9915	17.5576	8.460647	1.030876	0.782834	0.645093
0.173665	1572.15	33.67588	179.8526	-3.28446	8.003419	2.074219	0.585866	1.031958
0.176656	8965.369	2154.513	103411.4	-1146.18	20.53371	1.658416	0.667571	0.919892
0.226698	968.6422	32.24186	77.17643	6.334842	4.716431	1.56569	0.497507	0.889025
0.143394	7029.62	2202.32	66644.69	-727.414	18.83273	3.422276	0.536435	1.30063
0.116407	3836.069	96.60602	1059.997	-43.68	20.31615	3.634263	0.694598	1.354292
0.211721	1240.311	65.78093	109.5054	0.531149	5.3059	2.237739	0.402469	1.049388
0.268686	626.4395	47.18511	31.71307	1.778655	3.413353	0.903408	0.581596	0.64295
0.126383	3609.583	45.92873	987.8547	-40.1964	22.16427	2.87002	0.774428	1.163358
0.165334	2496.835	31.15379	577.9772	45.67085	14.08059	2.746527	0.675094	1.132686
0.457659	217.7347	39.42842	4.9054	-0.05757	1.027279	0.552283	0.296464	0.470172
0.291281	610.5764	25.59265	20.87979	-1.30762	2.292047	1.406373	0.226641	0.795935
0.189296	1429.501	56.86102	152.6154	-2.50195	6.347032	2.414646	0.443581	1.123115
0.260482	811.4245	23.92128	35.21499	-3.7689	3.106642	1.973359	0.227248	0.993161
0.151695	2200.316	157.5533	282.7956	-9.25203	9.030411	4.07237	0.360415	1.399107
0.227667	1109.63	1094.576	1060.901	-18.4282	4.848834	1.317789	0.553006	0.769839
0.331801	561.8855	48.88784	34.47104	-3.04844	2.357847	1.10628	0.340804	0.677168
0.357071	347.2668	16.7193	7.335485	-0.17834	1.523671	0.916153	0.247418	0.682546
0.350616	348.6908	17.59103	7.745585	-0.858	1.511186	0.878847	0.259415	0.650121
0.117589	3600.533	49.13602	938.04	47.79748	19.78541	3.919798	0.670899	1.420403
0.303579	587.8621	30.79939	32.02989	-1.10594	2.781135	0.941399	0.48283	0.63053
0.302226	563.9603	58.79657	28.21156	0.479088	2.715717	0.954702	0.472864	0.63019
0.167409	2000.445	75.68459	342.7503	-17.3121	9.186491	2.231834	0.589316	1.040948
0.228936	1024.484	57.78586	86.32537	-6.75035	4.863765	1.691193	0.480174	0.898192
0.290554	591.4564	18.8514	20.27473	-0.06294	2.370999	1.370481	0.262856	0.852328
0.18634	1407.53	34.93993	120.0067	3.797538	6.592766	1.765081	0.567736	0.963606
0.199256	1424.187	26.66394	107.1549	1.403439	6.185794	1.921981	0.515025	0.980951
0.317878	634.8655	45.23866	42.13504	-3.70974	2.485082	0.839491	0.447795	0.579254
0.178152	1471.001	75.58942	136.7911	-3.46717	7.585212	1.773427	0.620327	0.915104
0.239013	1178.216	70.42326	105.11	-9.34469	4.591931	2.106971	0.355277	1.003753
0.211695	1004.516	35.64162	76.36258	0.494626	5.821178	0.97077	0.714652	0.638835
0.206858	1387.85	42.95129	129.1697	-8.6605	5.776927	2.553713	0.369912	1.057484
0.292589	677.3196	52.60312	52.14783	-4.91495	3.423574	0.920332	0.570978	0.629514
0.187754	1278.721	39.93597	93.08848	-0.35819	6.085562	2.198396	0.467808	1.06948
0.237427	1034.971	38.40376	72.45549	-5.18542	4.213229	1.635884	0.418049	0.883163
0.272596	565.4054	7.26817	10.90009	0.105652	1.831009	2.410623	-0.11218	1.242636
0.293933	637.5183	61.98735	49.17042	-1.41098	2.718138	1.353184	0.327803	0.831864
0.235932	985.4827	71.24624	66.72299	-4.18642	4.15012	1.804136	0.383982	0.937919
0.280258	542.0328	15.80571	18.77238	-0.10116	2.714287	1.169077	0.39985	0.796178
0.214797	1261.375	1747.551	361.4548	10.22373	7.005863	1.153471	0.716672	0.717646
0.201512	1376.7	62.98606	131.8122	-7.66449	5.72252	2.137645	0.437112	1.017414

0.273816	733.4692	34.14066	45.12336	0.188092	3.370997	1.181986	0.470935	0.733183
0.27175	774.5193	45.60495	46.95728	-1.69405	3.497594	1.241928	0.462963	0.732286
0.294297	840.9231	51.63013	65.87889	-8.27565	3.174367	1.688253	0.283448	0.891807
0.217614	1114.407	38.4805	89.06737	-7.4713	5.709964	1.547792	0.575044	0.868939
0.279463	666.3162	79.95891	37.01854	-2.57555	2.93046	1.128684	0.432704	0.749544
0.206593	1099.373	41.98542	64.50117	-2.07739	4.755848	1.942563	0.408076	0.986313
0.162996	2102.772	68.74138	396.6809	1.336025	10.05349	3.110015	0.521288	1.228048

0.223102	1262.526	62.23829	109.8492	-8.09638	5.090729	1.978575	0.421018	0.961824
0.334851	1090.093	57.10742	5264.529	112.3992	5.576186	1.735443	0.526215	0.699141
0.332086	740.1122	66.56658	60.65991	-7.15125	2.709495	1.14463	0.372976	0.664745
0.270781	896.2173	53.33462	75.28566	-7.91766	3.817657	1.461301	0.441674	0.845064

0.367777	485.4449	78.044	34.53524	-3.1649	2.255218	0.658991	0.533499	0.478934
0.173967	1579.533	35.59285	198.9215	8.813674	8.568506	1.825566	0.648523	0.919336
0.227109	1878.72	56.90291	615.5655	49.22424	9.691532	2.048407	0.646048	0.902598
0.273931	557.016	19.12413	18.14577	-0.10765	2.792656	1.185135	0.407162	0.78963
0.232883	984.7059	52.86298	59.32812	-4.42448	4.197899	1.845325	0.380223	0.941928
0.257202	846.123	57.09213	55.79125	-2.49014	3.626341	1.356487	0.438438	0.760464
0.277245	648.0153	44.09636	29.1798	0.037839	2.897137	1.254549	0.391018	0.788794
0.207721	1534.586	52.26894	128.8542	-9.67836	5.109758	1.916681	0.356289	0.977524
0.290236	641.4307	28.98297	28.52634	-1.53736	2.691332	1.240249	0.357616	0.735913
0.164263	1727.443	45.11732	245.9401	7.818961	10.02947	1.551693	0.733197	0.817813
0.296557	534.747	22.9347	12.42547	-1.1775	1.79475	0.969542	0.232622	0.730883
0.344607	386.065	32.82977	16.22165	0.614838	2.138778	0.625192	0.54736	0.517048
0.175614	1815.035	57.14742	383.0226	26.65325	10.62453	1.568921	0.744313	0.841642
0.141439	3259.737	36.03072	358.262	-6.2974	12.97286	7.646723	0.263271	2.144842
0.252525	822.5332	32.56921	54.05068	-0.31567	3.806399	1.129455	0.530758	0.737818
0.223176	970.4467	55.09165	71.07603	2.434434	4.905197	1.29888	0.575913	0.777386
0.208352	1096.973	22.70731	100.2375	8.661739	6.337794	1.309318	0.660643	0.805857
0.244072	1004.031	59.32065	77.25754	-6.0891	4.148134	1.633742	0.416394	0.892585
0.182915	1437.696	34.26835	141.3782	3.334557	7.283299	2.678463	0.475862	1.212797
0.184377	1669.739	72.56186	158.6243	-8.36664	6.831196	2.85342	0.401059	1.136657
0.23932	1244.923	64.21831	122.766	-13.4529	5.080584	1.691865	0.482049	0.861145
0.167877	1903.036	64.37337	274.5356	9.61115	8.979709	3.209412	0.47601	1.289572
0.163291	2276.198	55.29314	288.6553	-4.63141	10.66259	2.474998	0.615438	1.007286
0.183813	1753.472	40.07398	106.4138	-9.00347	4.141354	4.55181	-0.08599	1.676901
0.461274	187.8883	29.76404	4.941395	0.977891	0.953679	0.467312	0.328175	0.411495
0.27676	763.9668	26.95967	49.58419	-3.93495	4.033517	1.222661	0.53988	0.759698
0.446216	192.7064	42.85607	5.978682	0.274592	1.017787	0.500289	0.3372	0.442576
0.225157	962.2635	52.79679	77.60311	-3.71916	5.173175	1.095875	0.648437	0.706034
0.471637	680.3266	1799.06	4016.844	-48.8608	1.340787	0.453969	0.339768	0.420091
0.235531	1174.849	38.5693	150.7458	15.71194	6.844999	0.750664	0.801264	0.548258
0.568152	155.4028	37.20481	2.861558	-0.06928	0.631611	0.422141	0.186641	0.385005
0.492558	276.6693	47.98225	8.518148	-1.01965	0.984146	0.566882	0.247736	0.453819
0.457905	244.1918	21.94048	7.557713	-0.59817	1.084656	0.591968	0.273677	0.465498
0.378489	617.0116	63.05838	57.05491	-4.47092	2.338435	0.936473	0.404684	0.591718
0.367891	373.5271	51.58251	16.38699	-0.17428	1.789583	0.749836	0.406802	0.588138
0.38151	348.9864	28.65517	12.93974	0.477557	1.579128	0.640791	0.400968	0.49894
0.339358	510.4832	47.45377	24.72822	-1.1345	1.973067	0.974624	0.321371	0.658579
0.434713	241.6334	10.10715	5.008259	0.443705	1.129275	0.601921	0.300156	0.507315
0.269705	690.2371	38.25553	31.99269	-2.12185	3.263659	1.058598	0.506561	0.684612
0.411266	487.8192	957.3862	864.7495	-18.6211	1.897009	0.669301	0.439866	0.469378
0.3192	594.8666	56.29155	31.81223	-2.47173	2.168827	0.940144	0.363875	0.66237
0.362986	447.7421	56.65242	21.83749	-1.77402	1.747996	0.898333	0.303813	0.63823
0.503687	181.5041	10.02432	2.446311	0.192128	0.714233	0.423265	0.215328	0.385127
0.366717	405.9913	24.15981	12.86038	-0.97352	1.656004	0.86202	0.293064	0.623482
0.316091	765.9173	38.86856	58.51943	-7.16701	2.808205	1.10643	0.38663	0.666153

0.49469	756.0182	821.8634	583.6809	-26.752	3.527793	0.630205	0.686472	0.362836
0.203433	1362.912	70.83387	280.4101	11.29579	6.902077	1.347498	0.666028	0.771388
0.444489	238.9542	39.13107	6.088731	0.730418	1.250545	0.464569	0.454986	0.416976
0.390071	3252.744	61.64453	75482.68	1029.016	18.27101	4.651769	0.603529	0.689645
0.3419	475.471	65.25488	24.93279	-1.22523	2.145181	0.7875	0.453317	0.585363
0.408829	294.1888	30.95079	8.398315	-1.12302	1.215788	0.646342	0.285776	0.518681
0.362022	377.4023	36.23816	11.70106	-0.38661	1.696349	0.707045	0.403006	0.538588
0.374166	351.2455	30.34626	13.5864	1.00889	1.72541	0.594965	0.47783	0.492732
0.324793	445.7687	14.67932	13.5309	0.091693	1.96578	0.823399	0.395368	0.609158
0.317445	795.9192	39.43912	41.09515	-6.2827	2.787071	1.433172	0.295665	0.759582
0.300156	537.6779	35.46705	23.02011	-1.66759	2.631037	0.897787	0.487343	0.616336
0.340139	405.0685	19.09456	13.37562	-0.65901	1.898853	0.783858	0.408349	0.599155
0.348335	413.0065	15.2527	11.39317	-1.25572	1.684281	0.788211	0.33694	0.591566
0.405399	246.6796	9.01539	4.403499	-0.02142	1.225863	0.611384	0.330677	0.512891
0.45252	244.5551	24.52381	5.893431	-0.45292	1.135882	0.546499	0.340367	0.458654
0.403505	277.1173	19.17405	8.464865	0.101132	1.229277	0.61577	0.312477	0.509692
0.54965	147.5602	10.99628	5.697713	1.170878	0.835875	0.358864	0.394032	0.324273
0.172122	1627.576	62.59337	206.7376	-7.41199	9.495979	1.187443	0.778003	0.702219
0.309357	707.6289	66.17423	47.63455	-3.89914	2.721015	1.213959	0.352444	0.705831
0.287373	616.8032	35.98424	27.40809	-1.72889	2.741957	1.118992	0.409138	0.729002
0.402368	287.0169	42.62251	7.05724	-0.3452	1.25275	0.626374	0.325181	0.517877
0.216315	1434.596	47.23931	207.1241	20.95672	7.871656	1.059065	0.760586	0.696424
0.430354	212.5025	41.90928	6.628093	-0.33534	1.065296	0.57243	0.290114	0.490665
0.340566	348.036	8.455993	5.150574	0.262855	1.474835	0.936854	0.224547	0.700696
0.466064	284.2531	48.568	10.21845	-0.89485	0.981825	0.64245	0.181924	0.518837
0.309704	593.0432	57.69634	42.15215	-3.64209	2.670922	0.819997	0.51451	0.601107
0.271047	771.6059	76.3321	63.74198	-5.35096	3.984131	0.97887	0.602388	0.651114
0.311754	1114.454	114.3725	241.8324	-18.3779	3.903596	1.109486	0.506855	0.656468
0.310101	728.2256	41.47627	52.54689	-4.50105	2.886339	0.992716	0.472478	0.657363
0.323714	554.7075	29.01903	28.50654	-0.7555	2.322721	0.880276	0.443249	0.636773
0.356897	711.4558	86.61201	94.5571	-9.69259	2.723414	0.890891	0.467563	0.552818
0.27715	996.7359	85.69622	104.7562	-9.00235	3.620385	1.462673	0.387726	0.756362

AX40	AX41	AX42	AX43	AX44	AX45	AX46	AX47	AX48
1.409723	0.487553	0.702183	0.685907	0.990232	0.939457	-0.11174	0.420211	0.460026
1.128786	0.327325	0.791389	0.785621	0.993351	0.955962	-0.25224	0.706886	0.369536
1.10927	0.304336	0.790355	0.785793	0.992155	0.951569	-0.12416	0.440097	0.38198
1.415297	0.49902	0.710281	0.693899	0.980973	0.919738	-0.1039	0.403888	0.442847
1.577084	0.642829	0.662904	0.636562	0.983157	0.920622	-0.18919	0.684003	0.451104
1.232217	0.379862	0.762781	0.754772	0.991948	0.949249	-0.14352	0.497262	0.407871
1.657401	0.690131	0.633268	0.604775	0.989369	0.932669	-0.09228	0.472962	0.491777
1.444343	0.520557	0.698565	0.682859	0.991666	0.94367	-0.12579	0.544579	0.470786
1.810217	1.096754	0.644902	0.612934	0.96173	0.889692	-0.11408	0.469641	0.420921
2.089248	1.533125	0.553828	0.504097	0.990192	0.935518	-0.13417	0.593666	0.433832
1.121983	0.308112	0.789247	0.78541	0.994996	0.960262	-0.0983	0.438764	0.389887
1.225227	0.382681	0.765646	0.758239	0.997076	0.968455	-0.19972	0.670157	0.406785
1.463525	0.543133	0.69666	0.678388	0.989305	0.937317	-0.20406	0.717502	0.453001
1.973933	1.23498	0.573501	0.526483	0.998862	0.976511	-0.21733	0.794326	0.428542
1.382527	0.457775	0.710633	0.695913	0.986143	0.929367	-0.10048	0.425456	0.456985
1.296139	0.409329	0.737691	0.727366	0.994713	0.956138	-0.20375	0.699737	0.439031
1.577953	0.63883	0.648516	0.619246	0.999328	0.982064	-0.25772	0.807096	0.45548
1.514539	0.667811	0.708833	0.691828	0.994013	0.954566	-0.24977	0.797298	0.434395
2.084607	1.598357	0.56514	0.517658	0.991164	0.939833	-0.17013	0.696149	0.428993
1.619568	0.739148	0.67596	0.65146	0.993559	0.950745	-0.26707	0.838784	0.436623
1.245192	0.378105	0.749349	0.741713	0.995278	0.958812	-0.25554	0.78516	0.437854
1.78702	0.967152	0.629931	0.597937	0.988393	0.933149	-0.22361	0.802186	0.457516
1.123986	0.315506	0.794623	0.789829	0.986374	0.939047	-0.12039	0.446845	0.371363
1.439617	0.555347	0.712719	0.695572	0.996296	0.962968	-0.24155	0.757992	0.429212
1.375113	0.504869	0.729394	0.716581	0.99372	0.953456	-0.18591	0.653409	0.432468
1.56924	0.635193	0.661897	0.637906	0.999415	0.983501	-0.18154	0.690159	0.472235
1.014899	0.260234	0.811389	0.80951	0.993598	0.95731	-0.12771	0.460044	0.361785
1.888899	1.18155	0.603994	0.563765	0.984837	0.924189	-0.21363	0.753612	0.428767
1.356579	0.460175	0.723656	0.711484	0.986919	0.932985	-0.17027	0.640155	0.450596
1.683617	0.795288	0.644364	0.615692	0.98635	0.927784	-0.17259	0.668649	0.462307
1.804062	1.092726	0.634214	0.60326	0.979516	0.91573	-0.11296	0.441077	0.450336
1.472905	0.573707	0.69893	0.681576	0.993471	0.950632	-0.2302	0.768523	0.454638
1.584926	0.647525	0.661704	0.638465	0.988965	0.93403	-0.12467	0.547611	0.478724
1.848712	1.256341	0.649893	0.616441	0.989562	0.940778	-0.21706	0.729667	0.393406
1.30554	0.460115	0.751877	0.74257	0.996534	0.965832	-0.29228	0.831785	0.416771
1.445543	0.515666	0.687626	0.667347	0.986571	0.929074	-0.1703	0.578709	0.455928
1.512005	0.622261	0.699415	0.679682	0.995319	0.958272	-0.30815	0.871217	0.432777
1.507864	0.569562	0.679905	0.659933	0.988485	0.933523	-0.18568	0.697057	0.472007
1.68864	0.818776	0.653207	0.626274	0.986773	0.92989	-0.10693	0.44746	0.459012
1.598825	0.687824	0.664197	0.638266	0.988154	0.933079	-0.16012	0.591538	0.449718
1.795592	1.037509	0.631135	0.599369	0.991859	0.944091	-0.14018	0.566917	0.454588
1.95225	1.577472	0.620042	0.58562	0.992824	0.949707	-0.23168	0.820866	0.437807
2.062408	1.597548	0.576592	0.530915	0.980254	0.914426	-0.19059	0.729084	0.419088
1.637489	0.835786	0.665404	0.639758	0.994913	0.956253	-0.23291	0.769316	0.44501
1.336725	0.44455	0.730777	0.719518	0.99894	0.979376	-0.24302	0.781523	0.444548
1.773421	1.041107	0.643453	0.613375	0.98058	0.918472	-0.13778	0.495947	0.442132
1.27275	0.409066	0.755803	0.746107	0.989056	0.941441	-0.13759	0.491748	0.407719
1.864719	1.080267	0.603258	0.561828	0.982951	0.91895	-0.21541	0.752441	0.423233
1.6945	0.788388	0.629594	0.595594	0.982135	0.916812	-0.18192	0.660075	0.447389
1.569499	0.680089	0.671199	0.647501	0.993764	0.950721	-0.25253	0.804662	0.454531
1.324071	0.447055	0.738455	0.72807	0.995906	0.961683	-0.19101	0.678848	0.435649
1.644841	0.780943	0.658555	0.633201	0.976963	0.9102	-0.12009	0.468326	0.461759
1.824114	1.021702	0.618013	0.582576	0.982416	0.91857	-0.16621	0.660322	0.448597
1.740095	0.887175	0.636375	0.604665	0.991637	0.942506	-0.16318	0.625628	0.449851
1.398349	0.550272	0.72922	0.715523	0.997992	0.973066	-0.21884	0.727364	0.425259
1.514206	0.682125	0.69885	0.680392	0.992629	0.949445	-0.15922	0.557431	0.441715
1.23904	0.371299	0.750547	0.742177	0.989591	0.94104	-0.18179	0.636219	0.429931

1.347237	0.460917	0.736051	0.724768	0.990112	0.942333	-0.10233	0.431144	0.433243
1.449611	0.516537	0.694052	0.675644	0.98393	0.923731	-0.2139	0.738372	0.458283
1.595311	0.707759	0.679193	0.655247	0.991757	0.944904	-0.15044	0.564245	0.435758
1.338027	0.490394	0.743795	0.732573	0.997033	0.968088	-0.25674	0.780225	0.416712
1.785244	0.890767	0.589084	0.5448	0.976999	0.903497	-0.20028	0.715056	0.434863
1.491903	0.588721	0.697268	0.679421	0.993326	0.95013	-0.14583	0.573427	0.453375
1.671317	0.828708	0.664318	0.638839	0.993705	0.951242	-0.23403	0.799392	0.448339
1.875739	1.170797	0.622123	0.587161	0.993517	0.949688	-0.22917	0.818826	0.441986
1.846328	1.074881	0.611079	0.574393	0.978055	0.909699	-0.1296	0.515954	0.450737
1.776728	0.8711	0.61478	0.580589	0.984287	0.920269	-0.12316	0.575312	0.473132
1.827622	0.971267	0.609852	0.570967	0.987035	0.927733	-0.21226	0.768809	0.439061
1.436718	0.502734	0.698342	0.682758	0.994131	0.95168	-0.186	0.711154	0.473131
1.301776	0.421618	0.739706	0.730671	0.996149	0.962372	-0.16485	0.641829	0.444864
1.830517	1.28797	0.649283	0.620166	0.994773	0.957333	-0.28361	0.872796	0.43326
1.582148	0.86627	0.708703	0.690477	0.995094	0.960503	-0.25967	0.820812	0.418764
1.179878	0.351483	0.772876	0.767039	0.999807	0.99147	-0.15754	0.579518	0.405793
1.386214	0.477855	0.7084	0.69389	0.975405	0.909107	-0.11078	0.443005	0.461661
1.485794	0.577251	0.69699	0.677718	0.988792	0.936721	-0.16846	0.613845	0.442911
1.17556	0.341151	0.77144	0.766052	0.996548	0.965608	-0.2113	0.700086	0.412406
1.420127	0.483269	0.695905	0.67995	0.985049	0.925392	-0.1498	0.610547	0.475559
1.745682	0.929673	0.641476	0.612808	0.996164	0.960454	-0.16228	0.676954	0.465011
1.477711	0.548736	0.680427	0.658137	0.988155	0.932906	-0.19782	0.661376	0.4525
1.588384	0.608905	0.655757	0.633449	0.995002	0.952889	-0.22039	0.82434	0.501225
1.835444	1.060129	0.619029	0.581732	0.989768	0.936947	-0.23574	0.794007	0.429157
1.53479	0.561222	0.666742	0.647305	0.99409	0.949362	-0.08977	0.536701	0.504056
1.636466	0.744662	0.655961	0.630047	0.998462	0.974114	-0.21165	0.758587	0.46478
1.16549	0.353781	0.778988	0.774197	0.99599	0.964151	-0.12215	0.489114	0.401305
1.67426	0.724651	0.637073	0.608235	0.984546	0.921265	-0.18476	0.740986	0.479772
1.5813	0.611471	0.659103	0.637709	0.993575	0.947439	-0.19045	0.777107	0.501599
1.669744	0.815916	0.658064	0.63196	0.986971	0.930988	-0.21246	0.761246	0.456163
1.905325	1.171342	0.592992	0.553749	0.979664	0.911533	-0.17003	0.692788	0.46049
2.002667	1.357447	0.576819	0.531694	0.989389	0.934134	-0.1717	0.690994	0.433973
1.628445	0.753969	0.677931	0.654221	0.991541	0.944465	-0.18939	0.700636	0.438686
1.559761	1.180178	0.693945	0.674147	0.997368	0.971714	-0.1921	0.649966	0.435731
1.697984	0.821855	0.639672	0.609048	0.985584	0.925931	-0.22398	0.770153	0.453928
1.837606	1.178009	0.624405	0.588443	0.992419	0.946583	-0.19448	0.667296	0.425679
1.709816	0.774348	0.62889	0.598435	0.990041	0.935334	-0.15254	0.668829	0.481078
2.001387	1.616546	0.598432	0.558246	0.992038	0.945623	-0.19524	0.725862	0.429104
1.486243	0.537922	0.681922	0.662806	0.990877	0.939789	-0.1087	0.496183	0.476365
1.454148	0.503137	0.67402	0.653868	0.999482	0.984169	-0.1169	0.469364	0.484854
1.565892	0.634328	0.67016	0.647404	0.989332	0.935779	-0.22459	0.784652	0.466534
1.525717	0.629234	0.682846	0.661513	0.991203	0.942738	-0.16336	0.585443	0.451985
1.726476	0.85533	0.627779	0.593854	0.981239	0.915693	-0.14133	0.523265	0.448218
1.70701	0.847968	0.640556	0.610285	0.989423	0.93603	-0.20748	0.738713	0.454355
1.413941	0.490798	0.706767	0.692692	0.990689	0.941009	-0.20815	0.748304	0.46858
1.981574	1.322067	0.572737	0.524492	0.978735	0.909117	-0.14922	0.54966	0.415062
1.705706	0.899887	0.642343	0.616543	0.996133	0.960069	-0.11434	0.584326	0.491318
1.402779	0.546243	0.729705	0.716908	0.99348	0.953216	-0.11276	0.445159	0.430657
1.395903	0.486487	0.715392	0.701558	0.986052	0.930391	-0.08748	0.353235	0.453228
1.402834	0.481509	0.705954	0.690626	0.981295	0.919169	-0.14436	0.567063	0.460332
1.616622	0.702165	0.654994	0.628829	0.991167	0.940722	-0.21471	0.755076	0.46572
1.522344	0.615608	0.682333	0.661516	0.995096	0.956004	-0.18464	0.667409	0.458174
1.393367	0.495459	0.72001	0.706683	0.994563	0.955323	-0.18976	0.69132	0.447657
1.815953	1.060853	0.625149	0.591088	0.988865	0.935075	-0.15146	0.592989	0.444886
1.600663	0.733932	0.671484	0.647092	0.991316	0.943301	-0.16522	0.588381	0.445765
1.291963	0.419512	0.752587	0.743713	0.99502	0.958763	-0.24512	0.772403	0.42054
1.564366	0.644401	0.669659	0.648562	0.989456	0.936129	-0.11288	0.503585	0.48052
1.578296	0.642272	0.659596	0.634998	0.99185	0.942443	-0.17709	0.678134	0.472138

1.613187	0.623927	0.645524	0.621402	0.994038	0.948213	-0.1396	0.688889	0.506582
1.729791	0.928962	0.640223	0.609276	0.98141	0.918488	-0.17722	0.64752	0.445312
1.762403	1.178186	0.663251	0.634642	0.997912	0.972661	-0.25741	0.824863	0.415766
1.681225	0.836176	0.64856	0.619017	0.991049	0.941491	-0.14725	0.500347	0.445028
1.754583	0.795021	0.610361	0.576395	0.982312	0.913903	-0.07491	0.43109	0.488452
1.723863	0.954037	0.646111	0.61433	0.993827	0.951602	-0.25703	0.802405	0.426516
1.780419	0.914316	0.605327	0.564667	0.973489	0.899462	-0.14536	0.492056	0.432591
1.262873	0.378804	0.739888	0.732356	0.986307	0.931463	-0.07503	0.384253	0.457746
1.627508	0.702963	0.667906	0.645779	0.99398	0.950874	-0.24007	0.847635	0.474669
2.078329	1.872777	0.579703	0.534662	0.983308	0.923106	-0.17152	0.594726	0.410112
1.913964	1.224108	0.598585	0.556582	0.99419	0.95105	-0.20193	0.717247	0.421636
1.476193	0.5553	0.694497	0.677815	0.987064	0.931329	-0.10392	0.470359	0.469685
1.466721	0.576211	0.696184	0.678182	0.99963	0.987386	-0.22199	0.741428	0.454992
1.739658	0.936939	0.633608	0.598529	0.989362	0.936477	-0.25335	0.785275	0.42248
1.433747	0.532536	0.713507	0.698931	0.994215	0.95387	-0.21183	0.747929	0.44975
1.968161	1.397019	0.595931	0.553613	0.982618	0.920126	-0.20678	0.739557	0.417015
1.342728	0.415581	0.713238	0.702176	0.991938	0.943779	-0.13831	0.617372	0.482407
1.428856	0.568112	0.729856	0.714394	0.996059	0.963256	-0.3068	0.85743	0.408473
1.744688	0.865859	0.61743	0.582562	0.98389	0.920174	-0.18671	0.694674	0.460973
1.610553	0.680898	0.643834	0.612744	0.999338	0.982238	-0.21943	0.729031	0.44789
1.612322	0.683891	0.6483	0.621231	0.989546	0.935138	-0.1505	0.583258	0.472038
2.012557	1.480542	0.575917	0.530824	0.998789	0.976602	-0.15852	0.609978	0.432483
2.02895	1.476425	0.569506	0.520977	0.989272	0.934189	-0.21857	0.768584	0.411558
1.846793	1.070511	0.615042	0.581887	0.987541	0.930173	-0.10209	0.528622	0.475446
1.355162	0.448954	0.717998	0.704386	0.992736	0.948045	-0.18676	0.659968	0.450935
1.903014	1.256598	0.614495	0.572945	0.978302	0.912828	-0.27138	0.839464	0.392063
1.922818	1.281886	0.617876	0.579524	0.979228	0.914836	-0.20786	0.74924	0.413002
1.157829	0.324672	0.777669	0.773069	0.994592	0.957865	-0.07148	0.351551	0.406715
1.574175	0.697556	0.684702	0.661529	0.979813	0.917851	-0.12377	0.463217	0.431061
1.865273	1.04005	0.596054	0.557046	0.990925	0.937855	-0.11695	0.531852	0.464239
1.779732	0.931809	0.625572	0.594584	0.965202	0.887896	-0.07829	0.470824	0.476978
2.068503	1.756292	0.567552	0.52023	0.986769	0.929206	-0.15117	0.5179	0.416432
1.52817	0.657878	0.686683	0.6669	0.999137	0.98108	-0.18623	0.667406	0.455905
1.443806	0.619101	0.713956	0.700725	0.989642	0.941372	-0.10962	0.468762	0.455856
1.392055	0.442282	0.695526	0.682012	0.982308	0.917853	-0.04203	0.305495	0.497779
1.342467	0.426885	0.710065	0.697782	0.977405	0.911066	-0.08387	0.361249	0.478069
2.071147	1.537484	0.56246	0.510825	0.978532	0.909165	-0.22135	0.781723	0.391501
1.360549	0.503973	0.726399	0.714443	0.989028	0.939565	-0.17231	0.608602	0.444702
1.376532	0.509843	0.7298	0.716292	0.994505	0.956458	-0.16549	0.568461	0.425591
1.793269	1.005416	0.623378	0.589408	0.987488	0.930851	-0.18867	0.687064	0.450711
1.655559	0.785213	0.653097	0.625575	0.986821	0.929849	-0.14805	0.563301	0.455188
1.578509	0.604422	0.649701	0.62517	0.979939	0.91079	-0.07471	0.396297	0.494667
1.695425	0.753839	0.6277	0.596197	0.986103	0.924549	-0.16405	0.666347	0.474663
1.718554	0.833923	0.635885	0.601672	0.978002	0.909875	-0.22644	0.77321	0.431641
1.315197	0.460145	0.747756	0.736014	0.990111	0.944325	-0.17495	0.549506	0.405433
1.681918	0.824736	0.652731	0.623622	0.989957	0.93845	-0.20284	0.715015	0.441117
1.813748	1.054084	0.626334	0.596385	0.986368	0.92823	-0.08582	0.472581	0.480466
1.405996	0.519295	0.728536	0.713308	0.990682	0.94433	-0.26272	0.799553	0.414698
1.822939	1.242582	0.633034	0.60049	0.977392	0.91365	-0.15848	0.544882	0.434247
1.382416	0.49453	0.726198	0.713418	0.992657	0.949312	-0.17755	0.653218	0.43969
1.788804	0.91579	0.608804	0.57164	0.979643	0.910607	-0.13768	0.556942	0.457636
1.654093	0.779005	0.655783	0.63021	0.987206	0.930881	-0.13384	0.533123	0.465473
1.52491	0.550772	0.551038	0.495277	0.919617	0.819101	-0.25345	0.789386	0.42991
1.588059	0.642202	0.65574	0.633437	0.996318	0.959596	-0.06382	0.395316	0.500271
1.720235	0.869764	0.638084	0.610513	0.988169	0.932209	-0.10381	0.510872	0.482845
1.48855	0.511125	0.659429	0.639151	0.977675	0.905501	-0.09161	0.499324	0.513287
1.475204	0.577445	0.702347	0.683739	0.999521	0.985816	-0.25147	0.787975	0.437468
1.804411	1.023578	0.623143	0.591835	0.986145	0.927285	-0.12295	0.563763	0.475658

1.50175	0.5987	0.695446	0.677193	0.990621	0.941626	-0.15159	0.594438	0.453336
1.501727	0.635641	0.702252	0.683247	0.990197	0.942044	-0.16618	0.573703	0.431576
1.690532	0.852644	0.652639	0.62824	0.984338	0.924402	-0.07824	0.378675	0.479555
1.608956	0.697306	0.657558	0.63074	0.98221	0.918846	-0.18192	0.66991	0.455686
1.497811	0.54705	0.680308	0.662433	0.993484	0.948203	-0.10105	0.509849	0.489433
1.747814	0.879211	0.628763	0.597335	0.981936	0.916983	-0.13074	0.56227	0.467526
1.934429	1.322682	0.594127	0.550662	0.988471	0.933339	-0.17411	0.597674	0.414919
1.761902	0.988231	0.638378	0.610369	0.987149	0.931051	-0.12012	0.555804	0.473784
1.474812	1.232918	0.716875	0.705019	0.999535	0.988777	-0.10844	0.542283	0.45778
1.476732	0.677589	0.725883	0.711847	0.991083	0.947397	-0.10836	0.451701	0.423769
1.615922	0.704684	0.659235	0.636325	0.986228	0.927512	-0.10741	0.515269	0.484304
1.213458	0.411416	0.7845	0.777658	0.997128	0.970694	-0.18942	0.625813	0.372319
1.701469	0.867814	0.654403	0.625111	0.988	0.933877	-0.21549	0.745972	0.434849
1.756864	1.169032	0.670691	0.646705	0.993236	0.952879	-0.19633	0.734934	0.436668
1.453263	0.500996	0.666027	0.644529	0.977437	0.906615	-0.13192	0.552691	0.490045
1.712592	0.864545	0.642899	0.613969	0.982868	0.920624	-0.12322	0.495841	0.460387
1.55167	0.728126	0.693434	0.675125	0.991044	0.944511	-0.15109	0.59336	0.451783
1.55689	0.610979	0.670999	0.650937	0.990033	0.937105	-0.08845	0.484573	0.488074
1.671214	0.806293	0.631528	0.599934	0.98217	0.917668	-0.15729	0.474427	0.461562
1.502834	0.639268	0.698061	0.67985	0.982266	0.923341	-0.13771	0.518511	0.446638
1.617599	0.790056	0.684277	0.660115	0.991214	0.944835	-0.27201	0.830075	0.420361
1.315445	0.388326	0.673009	0.658424	0.981209	0.91191	-0.11985	0.489633	0.534581
1.204967	0.346033	0.758475	0.752238	0.994928	0.957594	-0.16649	0.631917	0.431737
1.657081	0.802859	0.671809	0.647886	0.992285	0.946726	-0.24898	0.829577	0.447159
2.227703	2.059656	0.466877	0.393868	0.922192	0.827118	-0.25355	0.855114	0.314085
1.484595	0.550776	0.687303	0.669428	0.989177	0.935755	-0.1514	0.617546	0.474924
1.512535	0.611733	0.683355	0.661653	0.991313	0.942918	-0.19337	0.658616	0.448937
1.539075	0.599499	0.669179	0.64663	0.984713	0.923764	-0.20551	0.736683	0.471272
1.667463	0.787634	0.648077	0.623054	0.989206	0.935108	-0.11133	0.519603	0.486215
1.845533	0.981601	0.579197	0.533396	0.975241	0.899937	-0.1724	0.675961	0.437149
1.959625	1.467365	0.609513	0.574471	0.984569	0.925925	-0.11994	0.593358	0.457605
1.654453	0.849263	0.671133	0.645675	0.988921	0.937929	-0.15621	0.549371	0.433897
2.036767	1.3909	0.569829	0.523916	0.986538	0.925782	-0.11795	0.573297	0.443432
1.876042	1.346332	0.655233	0.624181	0.984257	0.929622	-0.22261	0.78489	0.401414
1.752322	1.070322	0.481732	0.414338	0.959746	0.866574	-0.42492	0.936992	0.421842
1.061452	0.286237	0.80263	0.799793	0.996201	0.966056	-0.10707	0.372672	0.371099
1.52023	0.600493	0.685206	0.665752	0.982196	0.920426	-0.18341	0.704048	0.46405
1.090522	0.293634	0.787441	0.784407	0.995931	0.963478	-0.09995	0.404348	0.399949
1.447496	0.541725	0.702534	0.685111	0.993687	0.951351	-0.21733	0.728306	0.447924
1.055708	0.272617	0.79531	0.793339	0.999776	0.990883	-0.06074	0.30489	0.393164
1.283075	0.421288	0.75599	0.745901	0.993962	0.955567	-0.32219	0.85725	0.403823
1.046453	0.27188	0.813563	0.811207	0.99356	0.957631	-0.0404	0.223921	0.353649
1.181299	0.356443	0.789659	0.784177	0.993234	0.955601	-0.06361	0.311735	0.374837
1.175732	0.359919	0.785802	0.779799	0.991141	0.94962	-0.10681	0.354107	0.378016
1.391821	0.563224	0.748575	0.736649	0.993754	0.956243	-0.12377	0.487545	0.399373
1.299884	0.396597	0.730789	0.721946	0.996227	0.961451	-0.09003	0.474126	0.465327
1.188031	0.362927	0.771444	0.764502	0.993776	0.955675	-0.14985	0.468279	0.39909
1.408668	0.524405	0.712743	0.700115	0.992288	0.946998	-0.08913	0.435449	0.467307
1.18877	0.335211	0.761366	0.755793	0.98821	0.937875	-0.07337	0.353938	0.43149
1.454336	0.559959	0.709002	0.693812	0.989907	0.940424	-0.15788	0.631218	0.452525
1.154997	0.441562	0.780744	0.77633	0.999508	0.987429	-0.12491	0.516376	0.401414
1.395434	0.472552	0.710722	0.696411	0.99091	0.941825	-0.1126	0.453783	0.459492
1.378444	0.479823	0.71705	0.705951	0.994814	0.955592	-0.06951	0.364462	0.471912
1.008682	0.260122	0.813579	0.81125	0.988731	0.945653	-0.11898	0.349109	0.35378
1.354082	0.450936	0.723644	0.711795	0.987202	0.933262	-0.09147	0.375713	0.453813
1.452479	0.62099	0.724461	0.709356	0.987238	0.937073	-0.14912	0.51386	0.419341

1.09582	0.494775	0.842692	0.837504	0.999527	0.990363	-0.24644	0.704836	0.268159
1.5577	0.711392	0.685013	0.66591	0.997499	0.968763	-0.21311	0.765052	0.464824
1.081735	0.286532	0.799158	0.796245	0.995432	0.962449	-0.13221	0.53253	0.377846
1.367175	4.138254	0.754401	0.746885	0.999132	0.990613	-0.1308	0.537077	0.414298
1.323	0.430073	0.737124	0.727139	0.996552	0.964137	-0.12096	0.527261	0.442436
1.208824	0.362376	0.759237	0.753186	0.990319	0.943717	-0.08751	0.340753	0.430789
1.250804	0.392577	0.755528	0.747303	0.991592	0.947609	-0.13186	0.489063	0.420182
1.170022	0.334879	0.769323	0.763785	0.994186	0.955961	-0.15275	0.553241	0.415524
1.315261	0.418124	0.728518	0.71676	0.990194	0.940983	-0.12692	0.462263	0.445754
1.597237	0.81002	0.703425	0.683762	0.979898	0.922301	-0.09637	0.364754	0.4179
1.350324	0.478747	0.73148	0.719209	0.989457	0.94078	-0.16758	0.593176	0.433259
1.281073	0.391338	0.729282	0.71881	0.992371	0.946903	-0.12707	0.474317	0.455135
1.280704	0.397269	0.735436	0.723862	0.979614	0.918927	-0.13659	0.455912	0.434051
1.191717	0.336458	0.759534	0.753404	0.977328	0.916832	-0.08761	0.394736	0.430824
1.138739	0.320212	0.784506	0.779439	0.991735	0.949995	-0.10977	0.397141	0.389286
1.177074	0.339158	0.761105	0.755737	0.99072	0.944504	-0.10368	0.401156	0.433023
0.962803	0.248237	0.843199	0.841312	0.992921	0.959932	-0.12597	0.435228	0.297991
1.489506	0.64529	0.71029	0.693491	0.995494	0.960175	-0.29543	0.861305	0.434364
1.490207	0.67995	0.70813	0.693153	0.993139	0.951793	-0.11741	0.477131	0.451541
1.477395	0.55231	0.690719	0.673417	0.989299	0.936529	-0.11241	0.486831	0.472567
1.211344	0.351449	0.757497	0.751798	0.994925	0.95752	-0.07608	0.389253	0.437705
1.462347	0.547705	0.702661	0.686981	0.99539	0.957753	-0.25691	0.82153	0.462003
1.154763	0.322418	0.76711	0.762778	0.996084	0.962695	-0.08155	0.352739	0.429854
1.294588	0.389936	0.68836	0.673268	0.965934	0.888794	-0.11506	0.445481	0.500207
1.240567	0.368762	0.759692	0.752796	0.993732	0.953747	-0.04274	0.245769	0.423737
1.323369	0.433748	0.73153	0.720907	0.994441	0.954921	-0.15427	0.585687	0.448127
1.396257	0.522529	0.718153	0.705073	0.995756	0.960443	-0.19499	0.69895	0.452542
1.454321	0.656187	0.724946	0.712057	0.994576	0.957952	-0.14517	0.582523	0.436191
1.429412	0.542806	0.716254	0.703255	0.992129	0.94723	-0.14269	0.599062	0.456273
1.382643	0.456669	0.719595	0.705888	0.989525	0.938488	-0.13129	0.571851	0.44775
1.306433	0.557347	0.762004	0.753423	0.994139	0.959134	-0.15739	0.543945	0.398766
1.561255	0.839255	0.699929	0.682463	0.990573	0.945237	-0.13813	0.535201	0.443291

AX49	AX50	AX51	AX52	AX53	AX54	AX55	AX56	AX57
4.190344	0.12962	3.51383	0.38489	0.26565	8.380689	2.550862	0.801982	5.984064
4.86391	0.165067	3.108316	0.648487	0.268775	9.727819	2.520662	0.785017	6.187583
5.869711	0.244439	2.651928	0.388414	0.436728	11.73942	2.0679	0.418234	5.75693
5.356404	0.155983	3.164043	0.311836	0.244783	10.71281	2.282449	0.648842	5.573491
5.626874	0.064689	4.359438	0.606357	0.119451	11.25375	3.218657	1.778896	6.482303
4.593309	0.188579	2.972048	0.459907	0.305762	9.186618	2.289512	0.616962	5.956246
8.305807	0.077835	4.23622	0.496132	0.16062	16.61161	2.939406	1.303282	6.054514
6.406121	0.103758	3.755753	0.475384	0.19161	12.81224	2.748936	0.953885	6.179272
5.21622	0.097465	4.074268	0.37814	0.225516	10.43244	2.863402	1.542615	5.886588
10.13004	0.028763	5.591567	0.523398	0.050783	20.26007	3.784839	3.781486	6.76803
6.855377	0.243222	2.633745	0.403975	0.436459	13.71075	2.062549	0.407449	5.682048
6.287377	0.148674	3.343963	0.593283	0.282658	12.57475	2.640532	0.802802	6.294715
5.569804	0.07783	4.189386	0.642147	0.142152	11.13961	3.154509	1.515367	6.593144
46.43558	0.026595	5.641876	0.745382	0.054381	92.87115	4.013969	13.62454	6.821455
5.695176	0.148406	3.305158	0.363154	0.292741	11.39035	2.39529	0.692643	5.599117
7.13078	0.109626	3.673158	0.62039	0.208936	14.26156	2.854152	1.008162	6.455111
44.17041	0.050114	4.682776	0.747	0.094504	88.34082	3.535619	3.85054	6.541239
6.432293	0.099199	4.17052	0.788834	0.198985	12.86459	3.186675	2.497302	6.684368
9.327325	0.026951	5.763084	0.62102	0.051834	18.65465	3.982631	4.805039	7.010619
7.750442	0.051857	4.736257	0.78978	0.107048	15.50088	3.602667	3.183832	6.938461
6.397574	0.107111	3.654428	0.719089	0.201748	12.79515	2.9228	1.153665	6.472982
7.632936	0.037548	5.144891	0.730725	0.073258	15.26587	3.76926	3.656636	6.942522
4.188563	0.246866	2.574912	0.392044	0.426605	8.377127	2.038866	0.416085	5.64134
7.999071	0.082866	4.158499	0.685885	0.176346	15.99814	3.191853	1.705512	6.706677
6.484424	0.113826	3.727335	0.581053	0.221114	12.96885	2.828178	1.037716	6.406374
45.39294	0.069655	4.313941	0.611622	0.122788	90.78588	3.159856	1.680669	6.296568
4.33598	0.249988	2.373846	0.445228	0.386623	8.67196	1.915915	0.34782	5.721999
6.355385	0.034731	5.390907	0.683986	0.064331	12.71077	3.876481	4.1941	6.927301
4.339659	0.109397	3.704268	0.551304	0.202746	8.679317	2.81183	0.972525	6.265389
6.020195	0.055239	4.626257	0.595048	0.106256	12.04039	3.330361	2.042878	6.630276
7.207658	0.090742	4.144178	0.367344	0.176598	14.41532	2.892417	1.614886	5.99372
5.199452	0.073927	4.311471	0.690309	0.162691	10.3989	3.285305	1.810025	6.73458
7.868265	0.083294	4.134876	0.52181	0.177947	15.73653	2.946864	1.299744	6.206901
6.325779	0.067437	4.873078	0.667756	0.201857	12.65156	3.557408	3.581163	6.834618
6.588882	0.114324	3.838049	0.802143	0.251023	13.17776	3.086476	1.798926	6.656988
7.014572	0.100459	3.8268	0.517966	0.189747	14.02914	2.841678	1.213346	6.023729
6.485055	0.058311	4.604982	0.817272	0.142656	12.97011	3.597413	3.099587	6.99814
4.458818	0.076563	4.167311	0.648704	0.141306	8.917635	3.099381	1.562524	6.390945
8.787529	0.089026	4.086112	0.395514	0.181245	17.57506	2.887445	1.36569	6.101687
8.269737	0.094888	4.009876	0.729463	0.1942	16.53947	2.917939	1.461884	5.97759
8.981837	0.056945	4.720866	0.493015	0.105852	17.96367	3.291361	2.021876	6.645514
9.24327	0.036742	5.449559	0.758252	0.076389	18.48654	3.943705	5.031264	7.171735
5.97768	0.029531	5.613895	0.664863	0.067178	11.95536	3.922783	4.811324	6.915465
8.252982	0.053919	4.7836	0.688255	0.095725	16.50596	3.553093	2.602657	6.912884
8.415161	0.090037	3.944891	0.720591	0.171383	16.83032	3.095296	1.403561	6.617449
7.153269	0.074053	4.416786	0.419048	0.155789	14.30654	3.093301	1.753684	6.432266
4.946935	0.181795	3.117025	0.43137	0.360108	9.893871	2.362898	0.635681	6.034294
5.453002	0.03423	5.288101	0.682123	0.063881	10.906	3.82835	4.058533	6.869947
5.747121	0.050085	4.750705	0.588072	0.085116	11.49424	3.424521	2.267252	6.616056
9.238841	0.056662	4.730913	0.730159	0.113193	18.47768	3.567068	2.557421	6.870926
8.080268	0.124927	3.632734	0.611641	0.257652	16.16054	2.804991	1.006574	6.366503
5.617475	0.085063	4.026005	0.408992	0.175438	11.23495	2.848405	1.285474	6.171153
6.454891	0.0465	4.896778	0.589987	0.115871	12.90978	3.48123	2.663891	6.593469
10.20525	0.056541	4.733729	0.55635	0.111003	20.4105	3.370466	2.177065	6.671104
7.300851	0.092353	3.935331	0.650414	0.16456	14.6017	3.020552	1.323533	6.657256
9.371496	0.108397	3.888919	0.594438	0.222843	18.74299	2.862928	1.286707	6.398265
4.533055	0.139857	3.356549	0.567308	0.264337	9.066109	2.618191	0.785372	6.187982

5.893881	0.185465	3.136536	0.388286	0.372531	11.78776	2.348738	0.642224	5.772094
4.232183	0.079324	4.173996	0.669977	0.169692	8.464366	3.15177	1.546821	6.450848
7.661015	0.086768	4.150415	0.49195	0.19787	15.32203	2.991599	1.380681	6.462523
7.252413	0.104038	3.859881	0.70982	0.197747	14.50483	3.045023	1.33848	6.620844
5.400339	0.040907	4.967385	0.610168	0.076315	10.80068	3.582819	3.064161	6.383245
7.269615	0.106781	3.886797	0.516107	0.220083	14.53923	2.864053	1.166902	6.316975
8.457989	0.053073	4.854171	0.724975	0.126901	16.91598	3.619672	2.906118	6.985852
8.934793	0.036237	5.313222	0.750924	0.081358	17.86959	3.886659	4.320331	7.062493
6.715802	0.053627	4.739091	0.450677	0.107054	13.4316	3.277845	2.126732	6.405447
7.68751	0.058561	4.588179	0.491432	0.111839	15.37502	3.196867	1.899731	6.127055
7.731408	0.040022	5.217589	0.689845	0.084921	15.46282	3.760851	3.514769	6.779668
7.324542	0.080825	4.044076	0.635528	0.134808	14.64908	3.052452	1.339756	6.414463
7.259574	0.136736	3.461918	0.597721	0.282741	14.51915	2.679036	0.8928	6.178437
5.409227	0.05331	5.186256	0.824572	0.131141	10.81845	3.907024	6.29703	7.074581
8.244961	0.081866	4.438085	0.752875	0.182933	16.48992	3.416277	2.814315	6.906317
43.65849	0.179147	3.042978	0.587643	0.317491	87.31698	2.39342	0.670675	6.006701
4.135063	0.139651	3.369601	0.354651	0.260746	8.270126	2.453298	0.724655	5.65694
5.694288	0.090092	4.004541	0.53931	0.180691	11.38858	2.949935	1.236252	6.494957
8.55075	0.153052	3.283372	0.634886	0.255794	17.1015	2.634313	0.77083	6.299522
4.52136	0.092166	3.854922	0.53687	0.163032	9.042721	2.863999	1.065979	6.280577
7.04753	0.058799	4.685218	0.604581	0.137874	14.09506	3.374211	2.278957	6.606939
6.569054	0.076187	4.123379	0.592851	0.126773	13.13811	3.080044	1.510387	6.46822
8.82876	0.050418	4.757898	0.761906	0.105754	17.65752	3.545566	2.538797	6.725249
9.421002	0.034429	5.292999	0.733824	0.065488	18.842	3.878023	4.339359	7.105557
7.883381	0.092713	3.98122	0.503499	0.194574	15.76676	2.842732	1.090093	6.037527
6.292661	0.052396	4.745187	0.688415	0.092837	12.58532	3.514343	2.454888	6.801963
5.529289	0.206751	2.771384	0.5364	0.282157	11.05858	2.170903	0.531413	5.834136
5.867197	0.04599	4.779092	0.665933	0.074597	11.73439	3.485582	2.391057	6.662186
6.495125	0.058151	4.620865	0.697411	0.122832	12.99025	3.423227	2.040807	6.615349
6.188762	0.051982	4.697966	0.690038	0.107299	12.37752	3.459941	2.483429	6.797962
7.002708	0.038076	5.150186	0.627329	0.073455	14.00542	3.63824	3.111468	6.489898
8.823984	0.030026	5.564694	0.616436	0.054055	17.64797	3.874157	4.050654	6.93053
6.371573	0.089658	4.207267	0.660296	0.189817	12.74315	3.131292	2.113845	6.432427
5.96336	0.093114	4.174843	0.584909	0.194419	11.92672	3.094741	1.956853	6.515872
6.044009	0.044812	4.914191	0.702914	0.088181	12.08802	3.623656	3.010509	6.817973
7.632518	0.052129	4.998384	0.617119	0.105366	15.26504	3.572474	3.251098	6.813627
6.804702	0.050051	4.722445	0.590697	0.092162	13.6094	3.378023	2.088291	6.463734
7.355625	0.036629	5.497864	0.651153	0.076256	14.71125	3.901658	4.705918	7.032356
7.312296	0.109029	3.73351	0.477107	0.196473	14.62459	2.676452	0.930923	5.770392
44.17402	0.097544	3.78639	0.467272	0.175364	88.34804	2.722865	1.970447	5.936001
6.639279	0.055685	4.60095	0.71463	0.096461	13.27856	3.439958	2.203837	6.683102
7.002641	0.084352	4.124272	0.517221	0.16909	14.00528	3.00822	1.328532	6.437792
5.879256	0.069203	4.475158	0.42833	0.148767	11.75851	3.121497	1.678751	6.170525
6.759818	0.049119	4.905511	0.688304	0.109161	13.51964	3.569741	2.694251	6.855664
6.197865	0.082817	4.072592	0.668071	0.172465	12.39573	3.12302	1.418493	6.573709
7.398003	0.039088	5.196832	0.483761	0.083784	14.79601	3.564952	3.137312	6.591237
7.998414	0.071806	4.445551	0.524354	0.150985	15.99683	3.134349	1.732779	6.332565
6.774702	0.153982	3.373278	0.393028	0.306981	13.5494	2.477885	0.746647	6.068758
5.56635	0.152415	3.258233	0.323075	0.26928	11.1327	2.349174	0.662992	5.768506
4.316777	0.107162	3.726694	0.493341	0.182603	8.633555	2.771886	0.962659	6.184976
8.431799	0.055694	4.684664	0.782034	0.117463	16.8636	3.453347	2.373284	6.729089
7.022643	0.076369	4.258436	0.593192	0.145037	14.04529	3.155991	1.555981	6.598488
5.673953	0.100704	3.846697	0.612205	0.189934	11.34791	2.938082	1.177473	6.468342
11.01371	0.063702	4.691502	0.612878	0.140382	22.02743	3.326657	2.391897	6.431691
8.401269	0.080578	4.271307	0.536303	0.15386	16.80254	3.08143	1.568456	6.50108
5.053639	0.127118	3.54759	0.749726	0.25739	10.10728	2.81183	1.231072	6.342544
6.47777	0.091335	4.008494	0.447227	0.161443	12.95554	2.869158	1.15279	6.140961
6.645532	0.065731	4.468284	0.604693	0.137701	13.29106	3.275597	1.782781	6.548503

7.021125	0.058689	4.508205	0.601907	0.11233	14.04225	3.257665	1.702819	6.354781
4.452129	0.059004	4.678396	0.577813	0.119975	8.904258	3.373509	2.245713	6.494538
10.06403	0.042993	5.178791	0.740457	0.085126	20.12806	3.827352	3.844083	7.335069
11.13938	0.076528	4.317708	0.450259	0.1568	22.27875	3.065125	1.66087	6.33601
5.674913	0.061703	4.472525	0.368131	0.121828	11.34983	3.037333	1.469376	6.057729
8.090668	0.042135	5.140114	0.728726	0.084952	16.18134	3.807641	3.66872	7.128314
4.441807	0.0531	4.653837	0.42881	0.094413	8.883613	3.243323	2.026203	6.277569
4.390971	0.174383	2.995162	0.376415	0.263108	8.781942	2.241673	0.526711	5.765862
7.41498	0.050966	4.92527	0.763884	0.115896	14.82996	3.699221	2.910342	6.994393
9.874681	0.037112	5.42541	0.522939	0.085637	19.74936	3.77619	4.370546	6.840898
10.95572	0.035925	5.419239	0.66207	0.075327	21.91145	3.845223	4.01005	7.019028
5.522998	0.109695	3.696282	0.411304	0.183043	11.046	2.669614	0.898979	6.095064
44.43376	0.079179	4.204432	0.724667	0.168445	88.86752	3.18256	2.559202	6.606046
7.76314	0.041624	5.143565	0.719472	0.088208	15.52628	3.786523	3.772284	7.031217
7.95444	0.096515	4.043699	0.719243	0.186026	15.90888	3.092212	1.564454	6.59653
7.571935	0.03185	5.471007	0.6858	0.060697	15.14387	3.868031	4.457412	7.019129
6.120568	0.11176	3.632049	0.550842	0.224524	12.24114	2.740353	0.886067	6.167675
6.431454	0.082556	4.3104	0.792006	0.201166	12.86291	3.402625	2.372881	6.922224
5.673837	0.046972	4.886326	0.606094	0.085154	11.34767	3.510013	2.51941	6.455727
46.36567	0.054687	4.635075	0.680093	0.103149	92.73134	3.414987	5.548031	6.611635
5.608241	0.073377	4.320877	0.53472	0.152013	11.21648	3.084416	1.57053	6.235735
46.88766	0.033336	5.451348	0.561693	0.066143	93.77532	3.746145	5.563753	6.847532
9.613913	0.028653	5.759133	0.704575	0.064668	19.22783	4.086389	5.987603	7.038962
8.063112	0.05888	4.687685	0.448802	0.113963	16.12622	3.208682	1.88591	6.375773
6.823309	0.100918	3.780462	0.588431	0.175104	13.64662	2.875658	1.07919	6.393216
6.410399	0.034397	5.418384	0.779396	0.093668	12.8208	4.029083	6.258572	7.01415
5.321458	0.051437	5.018716	0.682243	0.137907	10.64292	3.674866	4.206778	6.752344
6.26973	0.240298	2.608	0.316602	0.410607	12.53946	2.000074	0.394891	5.619544
5.03693	0.118292	3.660804	0.381887	0.245983	10.07386	2.561318	0.924605	5.992712
7.474665	0.048726	4.878028	0.460973	0.090231	14.94933	3.349864	2.190419	6.355956
4.861848	0.081867	4.10681	0.360069	0.155997	9.723697	2.770721	1.27	5.563578
12.50178	0.03762	5.295179	0.427285	0.069425	25.00356	3.574986	3.275695	6.61406
33.07104	0.076946	4.181008	0.632001	0.127115	66.14208	3.080673	1.541656	6.539723
6.969519	0.135917	3.483718	0.464938	0.277206	13.93904	2.566991	0.866032	6.080668
4.070308	0.140437	3.308114	0.255394	0.276337	8.140616	2.339615	0.609956	5.601103
4.175126	0.150853	3.157092	0.298789	0.253668	8.350253	2.281153	0.597508	5.539611
6.720766	0.027389	5.714677	0.731601	0.060558	13.44153	4.014825	5.926301	6.925461
5.50811	0.125354	3.561125	0.577472	0.212507	11.01622	2.713346	0.930634	6.203985
7.639125	0.128553	3.599353	0.493607	0.217836	15.27825	2.693546	0.917605	6.28533
8.598777	0.046991	4.975556	0.611018	0.090409	17.19755	3.572406	2.854581	6.783326
7.549265	0.071714	4.333819	0.503425	0.143394	15.09853	3.110931	1.63874	6.337005
4.312873	0.100318	3.830177	0.327598	0.188597	8.625746	2.636233	0.93537	5.804179
5.808005	0.055208	4.686408	0.59389	0.12819	11.61601	3.354696	2.089462	6.519975
5.059357	0.07115	4.379058	0.64942	0.183269	10.11871	3.250111	2.026944	6.0385
6.694935	0.148856	3.342541	0.515664	0.283916	13.38987	2.543329	0.831143	6.202026
8.610246	0.052974	4.703711	0.64406	0.098123	17.22049	3.434817	2.33966	6.755883
8.354675	0.073547	4.411369	0.434756	0.152849	16.70935	3.040839	1.674725	6.130588
5.867615	0.079505	4.102637	0.725896	0.139777	11.73523	3.19134	1.697987	6.760773
6.491687	0.066214	4.568158	0.474905	0.144741	12.98337	3.211107	2.08266	6.527504
7.209507	0.121728	3.713551	0.621209	0.216657	14.41901	2.815659	1.085977	6.414269
6.242118	0.049419	4.750024	0.485907	0.079787	12.48424	3.305488	2.07099	6.4008
6.144634	0.084348	4.182278	0.467143	0.166253	12.28927	3.003628	1.462278	6.282194
2.722	0.111635	3.532086	0.715037	0.179509	5.444001	2.300512	1.060408	5.012858
7.851472	0.097058	3.899772	0.41498	0.195599	15.70294	2.712434	1.017831	5.875008
8.405901	0.070764	4.337473	0.461648	0.138911	16.8118	3.027162	1.488564	6.268793
3.92618	0.088066	3.846118	0.430968	0.156556	7.85236	2.741836	0.970841	5.686817
41.78622	0.079679	4.274067	0.745352	0.188598	83.57244	3.280767	2.039833	6.719882
7.879621	0.057202	4.657281	0.492771	0.107901	15.75924	3.244059	1.965041	6.422148

5.795828	0.103609	3.883796	0.551901	0.195228	11.59166	2.861022	1.138246	6.332608
6.711134	0.108388	3.901635	0.506788	0.233809	13.42227	2.876742	1.18488	6.438032
7.159208	0.112019	3.808791	0.353117	0.175219	14.31842	2.656854	1.215655	5.781825
6.118811	0.071209	4.349027	0.593242	0.162545	12.23762	3.186688	1.814439	6.420687
8.91656	0.097061	3.847469	0.47483	0.184899	17.83312	2.777534	1.014786	6.115243
6.424736	0.06115	4.463872	0.487743	0.111044	12.84947	3.124821	1.674603	6.269132
8.185229	0.043984	5.263289	0.529269	0.091799	16.37046	3.66348	3.290877	6.721151
7.839509	0.070722	4.434973	0.506818	0.14915	15.67902	3.14215	1.767326	6.369776
7.493142	0.135761	3.577365	0.598814	0.231546	14.98628	2.661615	1.827907	6.1098
8.134622	0.156311	3.393295	0.419475	0.289842	16.26924	2.522267	0.963531	6.106322
7.262604	0.09421	4.038206	0.474573	0.164562	14.52521	2.884653	1.31974	5.977321
8.81161	0.199245	3.12229	0.580754	0.391079	17.62322	2.467995	0.728552	6.178625
5.822759	0.053029	4.777865	0.672371	0.114002	11.64552	3.514123	2.598518	6.808526
7.415623	0.088605	4.474661	0.692408	0.16771	14.83125	3.276961	2.934985	6.63933
4.326831	0.09114	3.755103	0.459769	0.143874	8.653661	2.714722	0.994448	5.509112
7.230128	0.071863	4.296834	0.438718	0.138487	14.46026	3.015671	1.510806	6.265811
7.518232	0.095115	3.99201	0.537349	0.171858	15.03646	2.91943	1.245707	6.457673
6.609503	0.092723	3.950504	0.42169	0.162039	13.21901	2.795085	1.037921	6.156653
7.170955	0.069603	4.32136	0.400898	0.1293	14.34191	3.041585	1.75661	6.272715
5.349783	0.114027	3.706176	0.457831	0.192371	10.69957	2.697363	0.982895	6.147935
6.55724	0.053695	4.756836	0.765258	0.115479	13.11448	3.601098	2.895291	7.024753
4.767242	0.134841	3.277929	0.383511	0.231479	9.534484	2.325061	0.691073	5.285071
5.696608	0.149624	3.24846	0.556284	0.267704	11.39322	2.554931	0.690992	6.139365
7.408329	0.056529	4.802497	0.75586	0.140854	14.81666	3.610155	3.048361	6.951879
5.890437	0.040081	5.110325	0.674991	0.100638	11.78087	3.617013	5.154895	5.897828
5.647951	0.093187	4.020634	0.542373	0.166345	11.2959	2.971287	1.233964	6.341971
7.361383	0.077914	4.201574	0.593542	0.143205	14.72277	3.121338	1.551019	6.532718
4.631319	0.068158	4.321866	0.674091	0.125786	9.262638	3.23438	1.911778	6.395527
7.660927	0.079201	4.225987	0.472165	0.163839	15.32185	3.00028	1.445469	6.233315
5.754385	0.047255	4.894992	0.528087	0.09261	11.50877	3.433496	2.49044	6.114497
8.459741	0.048368	4.928384	0.517702	0.101688	16.91948	3.36802	2.421154	6.600975
7.960345	0.096496	4.138874	0.509802	0.197208	15.92069	2.986264	1.693112	6.389577
7.932855	0.038077	5.312056	0.492604	0.081602	15.86571	3.595128	3.04728	6.52339
7.296688	0.06037	4.883706	0.71845	0.170681	14.59338	3.565608	3.284396	6.838412
6.333817	0.092445	3.95686	0.926702	0.166809	12.66763	2.570575	2.173291	5.109665
5.444443	0.268332	2.287953	0.376233	0.410635	10.88889	1.811242	0.355248	5.510857
5.124083	0.096527	3.948979	0.602335	0.175702	10.24817	2.96156	1.314045	5.741393
6.536566	0.231062	2.48791	0.434774	0.316604	13.07313	1.947663	0.379519	5.68196
7.19564	0.081185	4.175985	0.658879	0.166669	14.39128	3.169158	1.567263	6.654317
42.41271	0.250636	2.249539	0.44031	0.351683	84.82542	1.757979	0.448689	5.583584
6.086515	0.111486	3.930284	0.813352	0.253504	12.17303	3.182918	1.898916	6.727555
6.09528	0.362985	2.113146	0.260081	0.571056	12.19056	1.636462	0.263438	5.171745
6.919386	0.307458	2.46634	0.329083	0.521091	13.83877	1.896198	0.387757	5.383395
4.670899	0.260645	2.511181	0.361039	0.429649	9.341798	1.940424	0.419156	5.586458
7.918799	0.200786	3.232486	0.529535	0.399691	15.8376	2.431875	0.818727	5.989089
7.163974	0.156204	3.237545	0.454596	0.303081	14.32795	2.425277	0.634855	5.857706
5.331083	0.197776	2.928732	0.435306	0.340032	10.66217	2.25586	0.55498	5.957591
6.870524	0.150228	3.367164	0.426796	0.302758	13.74105	2.464435	0.736923	5.809649
3.158225	0.222173	2.752048	0.318873	0.406445	6.31645	2.085695	0.432799	5.569689
6.140325	0.103748	3.817791	0.548268	0.195067	12.28065	2.851144	1.080564	6.224946
30.9367	0.211156	2.827227	0.536311	0.394839	61.87339	2.226	0.641578	5.905561
7.482265	0.139006	3.400286	0.460653	0.2216	14.96453	2.490168	0.777243	5.928146
7.512617	0.157749	3.224738	0.378777	0.239172	15.02523	2.348677	0.661582	5.763529
3.154581	0.341041	2.109332	0.298169	0.53748	6.309163	1.65319	0.284374	5.141288
4.895026	0.167442	3.190285	0.344218	0.334199	9.790052	2.33712	0.629506	5.713274
6.199862	0.15448	3.294798	0.446746	0.243211	12.39972	2.526387	0.978659	6.058089

28.65552	0.33858	2.568791	0.776443	0.553119	57.31104	2.124007	1.0395	5.718675
8.33338	0.067029	4.490891	0.689406	0.134284	16.66676	3.376574	2.062394	6.826801
6.239748	0.244621	2.645338	0.516875	0.437487	12.4795	2.121492	0.428779	5.726459
7.631474	0.187411	3.111089	0.691783	0.286599	15.26295	2.383874	5.730694	5.987058
8.056999	0.153006	3.335482	0.504812	0.305994	16.114	2.518805	0.73317	6.125264
5.550513	0.2092	2.730903	0.322527	0.329337	11.10103	2.074683	0.465532	5.679909
5.999234	0.175328	3.105926	0.501873	0.339727	11.99847	2.35871	0.600849	6.008701
5.482968	0.180263	2.995377	0.501837	0.274139	10.96594	2.357291	0.580094	5.957466
3.793901	0.146966	3.32624	0.401874	0.286201	7.587801	2.478704	0.697295	5.958716
6.25274	0.152739	3.410343	0.332572	0.27658	12.50548	2.504828	1.055061	5.924009
5.918762	0.123717	3.523083	0.523943	0.231453	11.83752	2.677247	0.882206	6.179874
4.337717	0.147012	3.269326	0.433691	0.223916	8.675434	2.446797	0.670678	5.999001
3.87655	0.183339	3.094964	0.391547	0.360274	7.753101	2.289979	0.618123	5.578189
2.976705	0.194166	2.836701	0.335221	0.366366	5.95341	2.15473	0.459312	5.757706
4.937217	0.253874	2.645772	0.385861	0.45487	9.874433	2.044875	0.420595	5.64751
4.361219	0.210556	2.700197	0.359269	0.31592	8.722438	2.075975	0.461262	5.569688
3.298034	0.36663	2.005626	0.478125	0.558462	6.596067	1.635676	0.298685	5.433877
7.779194	0.060121	4.529139	0.799706	0.131349	15.55839	3.524858	2.670856	7.063591
8.111534	0.129784	3.618818	0.448271	0.260579	16.22307	2.655702	0.983744	6.144699
5.964725	0.108137	3.77716	0.429805	0.225052	11.92945	2.730995	0.965237	6.17869
6.516586	0.193051	2.832292	0.342372	0.280308	13.03317	2.155526	0.469781	5.790715
6.747929	0.085597	4.24863	0.781606	0.211529	13.49586	3.281184	2.23268	6.660799
6.464172	0.21306	2.608618	0.36825	0.272718	12.92834	1.998604	0.409432	5.640956
2.884677	0.139166	3.101639	0.349288	0.202853	5.769353	2.22716	0.602922	4.070864
6.96294	0.255689	2.643426	0.289048	0.458317	13.92588	1.928387	0.406069	5.45096
7.56513	0.132862	3.490415	0.538574	0.249784	15.13026	2.661749	0.87273	6.190619
8.69372	0.10344	3.866864	0.653467	0.224384	17.38744	2.952826	1.24075	6.465244
10.66146	0.14328	3.571599	0.600122	0.301159	21.32291	2.723458	1.253271	6.24125
6.403199	0.149753	3.459534	0.559454	0.271486	12.8064	2.618342	0.969764	5.895888
5.353265	0.147384	3.418757	0.642324	0.295018	10.70653	2.547458	0.800749	5.671007
9.281756	0.177891	3.130079	0.517966	0.262152	18.56351	2.443928	0.903576	6.101192
9.227831	0.111382	3.806719	0.480379	0.19985	18.45566	2.80749	1.270764	6.298696

AX58	AX59	AX60	AX61	AX62	AX63	AX64	AX65	AX66
1200.67	0.054795	25.71404	7091.766	0.843001	18.4702	147.9239	2548.677	9.517969
2092.348	0.045591	37.54753	14395.65	0.903677	25.6106	253.5851	5661.262	12.51717
1368.359	0.043831	38.37619	14051.69	0.437602	34.58064	254.6694	8913.692	7.464322
70.21987	0.057182	26.02577	419.2866	0.760517	27.82085	118.5489	3692.485	3.992653
342.3565	0.063341	19.98411	1121.652	1.802551	33.14598	115.8067	4000.908	4.230124
928.6248	0.046452	34.67506	7360.001	0.756704	22.1023	209.5545	4168.136	11.24702
562.4316	0.069591	16.21355	2042.217	1.441036	69.92168	89.08117	6185.514	1.340208
875.0626	0.056231	23.75611	4378.988	1.0505	41.89641	142.6533	5938.057	3.656337
76.50584	0.063861	21.31526	310.9032	1.80254	27.70367	95.26377	3051.508	3.434328
3231.119	0.081596	12.6197	5529.54	3.953828	104.5264	66.16008	6942.785	0.722632
8555.937	0.044609	40.07827	87722.55	0.445027	47.13723	274.5456	13002.13	5.903332
21428.58	0.04593	34.59808	154121.8	0.812272	40.33455	248.8906	9688.4	6.756491
1785.274	0.055156	25.26231	7437.582	1.504699	32.22173	152.8068	4819.938	5.72798
438.9871	0.086076	12.58243	627.4925	36.3029	2140.36	65.9698	142365.5	0.410345
96.52797	0.065844	19.42632	509.2142	0.786413	32.87176	103.1501	3508.158	3.180416
5494.893	0.04836	31.47834	31477.44	1.029212	51.56296	204.1298	10357.28	4.28167
287.8743	0.07054	18.02763	666.3992	8.461107	1953.939	103.4259	199294	0.080714
2143.458	0.048457	31.83366	10453.68	2.529154	44.3614	175.1151	6600.004	5.269952
3037.71	0.078014	14.94791	4950.985	5.028909	90.08308	71.27228	6082.343	1.020154
5934.118	0.051075	30.98223	18960.96	3.119577	63.00321	158.9783	9375.79	3.602474
8114.983	0.050496	28.88569	41806.67	1.179429	42.6174	213.8695	8559.185	5.863546
2711.403	0.061684	21.39442	6326.771	3.616608	62.33336	109.5431	6162.752	2.558095
189.4491	0.046548	37.58525	1751.266	0.493922	17.90147	206.6747	3682.038	12.13057
6888.744	0.050901	29.88111	29897.8	1.717537	66.32427	185.0399	11378.28	3.269856
2106.995	0.04923	30.82649	12083.81	1.106384	43.20753	187.8096	7964.085	4.787484
262.78	0.066763	18.40524	873.7967	2.154321	2064.389	104.9009	219232	0.050577
903.2033	0.045378	36.19977	9063.021	0.367078	19.2811	254.593	4801.696	14.24196
2121.973	0.07138	17.3758	4209.08	3.956324	45.01231	94.44894	3717.328	3.710406
493.7908	0.055277	25.25372	2549.461	0.974863	20.11855	154.7466	2957.578	9.58126
1073.451	0.061689	21.26045	3355.105	2.047561	37.90702	113.8528	4017.827	3.946296
226.2748	0.066925	18.40055	872.5534	1.974732	51.82727	89.60101	5095.944	1.716046
3410.791	0.053719	26.20508	13563.43	1.857046	29.50176	158.8232	4269.076	7.46792
333.2251	0.064779	19.04507	1309.349	1.444051	62.44732	106.9102	6789.884	1.791891
2713.497	0.05543	32.86961	8703.964	3.672958	44.9981	140.3256	4729.142	5.073796
20968.9	0.046028	34.11357	117943.2	1.838891	45.53932	230.003	9580.486	6.041096
372.8613	0.064475	22.07422	1372.67	1.48837	46.90334	116.0901	6254.23	2.353058
8463.88	0.050776	30.51904	27135.74	3.108051	46.31901	174.7211	6764.269	6.004937
265.3714	0.060325	21.73232	980.7527	1.702738	22.32371	118.8841	2256.756	8.19695
498.7025	0.059575	22.98528	2122.535	1.569838	76.23092	111.9112	9382.644	1.387667
131.5056	0.064432	21.23616	513.0637	1.750872	66.9559	105.6761	7837.523	1.503637
1455.719	0.06127	21.60526	4681.622	2.206638	81.81864	105.6301	8642.973	1.391098
14016.72	0.058001	24.09274	33880.83	4.971411	89.7947	118.0704	9075.229	1.862668
966.8806	0.074079	17.29531	1659.271	4.928721	39.59079	80.9623	2559.938	4.492154
6014.137	0.058097	23.91905	18274.64	2.587057	71.47566	139.1888	9296.087	2.397215
2955.273	0.050812	28.96574	13928.66	1.536688	72.04083	176.3823	12297.71	2.70401
348.4923	0.059368	25.08727	1243.276	2.123709	50.23578	110.261	6144.551	2.161925
402.5065	0.044639	37.98743	3265.64	0.71578	24.78962	202.07	5172.049	8.370531
2996.555	0.072295	17.33384	5755.06	3.973574	34.43719	95.14447	3088.135	7.209778
1803.022	0.068	17.93799	4892.266	2.196278	35.32506	106.1421	3553.014	4.16315
1976.467	0.059022	23.70938	5964.588	2.627631	87.20533	128.2676	10899.76	1.675022
3487.009	0.048295	31.45274	21451.4	1.051978	65.59271	195.7832	12604.76	3.160691
237.115	0.060892	21.61837	956.9656	1.38372	31.43349	110.396	3828.777	3.623867
602.9941	0.068173	19.41673	1547.817	2.604374	43.29565	96.46523	3916.259	2.963959
1064.86	0.061727	21.79712	3287.846	2.388523	103.4417	106.4369	11729.35	1.034728
4810.34	0.047506	32.84732	24153.37	1.43546	53.62752	199.6499	10791.43	3.993802
1823.135	0.051782	28.69381	9602.571	1.589024	87.61086	160.5143	14808.36	1.813453
938.6664	0.049068	30.84728	6119.314	0.805325	21.56519	201.5286	4076.301	11.05052

248.6102	0.048652	33.29041	1950.604	0.729105	34.68082	167.8595	6047.538	4.803545
283.489	0.05996	22.37122	1062.972	1.563365	19.15969	127.0871	2364.821	9.304041
730.9784	0.05473	27.00202	3273.917	1.480419	59.27957	137.6171	8496.138	2.391903
23539.24	0.04684	33.58215	128135.9	1.34688	54.21385	226.7763	11446.04	4.844498
439.4789	0.08725	11.73444	824.3186	2.88458	33.75025	71.18384	2484.398	3.431664
1676.2	0.053399	26.78493	8851.106	1.251972	53.51994	147.1396	7601.314	2.996499
4838.384	0.055215	26.23074	15100.18	2.980149	73.72467	137.864	9333.763	2.310503
5231.45	0.060425	24.94353	12059.05	4.304357	83.69349	112.6483	7944.855	1.958971
385.7746	0.07081	16.82797	1036.156	2.27097	45.22302	85.96072	4242.121	2.062289
198.369	0.076798	14.16097	538.8211	1.988478	59.54975	73.0813	4744.091	1.242917
290.9838	0.07132	16.28118	602.5603	4.023139	63.46422	85.49706	5467.382	1.644433
3631.264	0.0538	26.16346	15950.28	1.359381	55.69175	156.2667	8069.61	3.287145
16134.93	0.049474	29.86272	106563.8	0.901584	53.64711	209.2798	10745.43	4.240297
7655.144	0.05346	28.72875	21879.87	6.386393	36.33896	138.9628	3132.144	10.05867
9792.026	0.044673	37.49144	46925.85	2.840059	70.97268	195.5089	12069.8	3.424925
5853.372	0.045978	34.62465	48287.37	0.930915	1905.407	243.6064	464231.3	0.131402
90.08124	0.06476	20.27837	463.5794	0.776789	17.23436	110.1531	1993.104	6.790209
893.9275	0.0519	29.26982	4352.414	1.249273	33.87059	159.2012	5131.474	5.626476
6374.145	0.047382	32.81295	44495.49	0.82626	74.0819	219.7133	16147	3.082078
800.4403	0.055865	24.22061	3758.68	1.118598	21.97802	137.4422	2826.995	7.945739
4260.798	0.059204	22.44205	14187.22	2.339131	52.61647	117.7491	5357.29	2.911953
1058.528	0.058673	23.1697	3879.258	1.649754	43.22477	136.4297	6410.276	3.336519
5180.508	0.063372	18.68586	14516.6	2.573187	80.67028	119.3794	9371.052	1.693755
2975.534	0.063289	21.6637	6222.274	4.634502	92.60625	108.5821	9743.225	1.704117
1119.593	0.061106	19.74014	5079.495	1.197791	62.7366	122.5121	7740.681	2.027523
5105.483	0.060363	21.53776	15542.01	2.583879	42.21669	126.7964	4984.383	4.08127
14309.86	0.047575	33.14031	126603.8	0.544729	31.00314	260.0223	7997.13	8.805055
1635.176	0.064986	18.63672	4431.969	2.348679	36.549	105.557	3779.862	4.008986
4329.577	0.062094	19.27113	13736.01	2.125587	43.64068	122.5474	5244.485	3.384355
1180.587	0.056307	24.89836	3658.815	2.491243	40.74789	128.3589	4652.357	4.553253
240.7797	0.082374	11.82267	456.9275	3.379986	51.98803	69.86486	4004.592	1.588131
2991.093	0.074826	15.85525	5446.111	4.147298	80.25321	79.26822	6115.875	1.260905
3896.863	0.051278	30.84583	17676.23	2.158518	43.5158	156.9413	5439.722	5.116886
4370.362	0.054475	26.37104	20499.95	1.942298	37.17995	158.5232	5571.99	5.079731
1197.512	0.064681	19.79096	2931.112	3.055549	39.96738	108.5784	3963.778	4.104865
5197.678	0.064571	21.30944	14058.06	3.395011	62.1227	113.6891	6120.977	2.432578
448.463	0.069648	16.34061	1200.641	2.163759	46.29057	92.79376	4536.217	2.26638
3236.018	0.064942	19.96507	7043.824	4.853194	60.80722	97.79685	4790.122	2.511775
162.6705	0.072816	16.40537	655.3841	1.035451	53.30304	89.73232	4867.178	1.7254
308.7868	0.069018	16.75097	1229.789	6.173868	1946.563	99.4658	194341.1	0.07524
1036.343	0.060495	21.76234	3178.708	2.199596	44.88897	116.5425	5344.27	3.055806
2430.147	0.057262	23.82209	10377.63	1.355459	49.91769	143.1185	7012.895	3.157212
624.58	0.077071	15.66302	1827.583	1.691703	36.3423	83.50987	3099.794	2.563571
4518.538	0.062975	20.33391	12358.95	2.725204	48.15154	115.0898	5468.953	3.171497
1292.723	0.053201	26.40889	5637.246	1.530371	39.83077	158.4001	6530.469	4.39534
886.784	0.077925	14.62518	1805.171	3.20265	56.87179	74.929	4614.904	1.551549
806.5349	0.065625	17.85973	2787.345	2.243901	66.58153	90.01432	5796.181	1.494089
1243.61	0.047577	32.54022	9041.62	0.848305	46.2471	187.2632	8767.632	4.13695
344.5341	0.056555	24.68898	2165.206	0.734356	31.14527	137.9025	4434.918	4.508752
600.0034	0.056775	23.70723	3016.78	0.993583	19.75606	145.8424	2912.734	8.585716
6176.182	0.063423	19.71719	17953	2.442938	72.35294	115.931	8499.28	1.836568
12757.9	0.056062	24.94556	52094.87	1.598844	50.67442	144.6609	7062.429	3.257536
4957.839	0.049822	29.80006	26701.08	1.180149	33.38821	185.2802	5750.067	6.658537
996.1898	0.067621	19.56616	3143.459	2.43245	123.2384	98.03747	12897.5	0.78131
1352.045	0.057188	24.38222	5481.343	1.716765	71.19952	131.3728	9516.047	1.938785
6031.521	0.044254	37.23706	37179.87	1.267287	27.43414	230.5577	5342.219	11.48041
1288.905	0.062179	20.13812	5494.509	1.286817	42.57065	114.9686	4963.139	2.854732
3571.164	0.062421	20.01014	12220.56	1.812308	45.72219	119.6884	5537.554	2.985315

1645.968	0.068895	16.06385	5144.479	1.689159	50.91679	107.604	5307.284	2.452253
836.9263	0.068082	18.02115	2523.676	2.084868	21.4483	105.5432	1988.933	7.913912
6614.343	0.052083	29.89009	18162.26	3.953049	103.1422	151.1272	15166.26	1.73511
619.2293	0.065548	23.11187	2082.238	1.943817	121.3053	110.0196	14405.77	0.871822
851.8323	0.076576	13.88152	2605.311	1.444163	33.27571	74.10716	2517.701	2.47278
8008.049	0.060851	23.12318	19130.85	3.674994	69.62987	130.1989	8423.614	2.53254
834.3295	0.077938	14.9354	2040.99	2.047104	23.53321	84.78627	1695.783	6.192602
2018.173	0.051262	28.05759	15312.59	0.581089	19.70244	187.9924	3729.616	10.1803
2893.755	0.058222	22.4287	8667.368	2.885965	57.37836	136.3075	7930.702	2.902865
1613.077	0.073872	16.96696	2987.395	4.946769	96.46987	84.07694	9299.399	0.923935
3110.64	0.069736	18.11056	6408.72	4.095183	123.285	94.9501	11794.61	0.881881
763.2238	0.055791	23.87733	4055.396	0.986533	30.6731	136.1692	4308.394	4.65609
3068.338	0.054785	25.48254	12661.43	5.035187	1976.952	153.4398	302747.5	0.123732
5137.631	0.067112	20.18554	10719.69	4.134009	62.40367	114.8911	7699.891	2.26692
3437.324	0.048788	30.79585	17782.23	1.616427	64.18629	175.8716	11811.28	2.881884
1607.147	0.069262	18.23455	3036.46	4.440273	59.62166	92.51483	5543.312	2.253776
3052.254	0.054701	24.83292	16390.06	0.947458	38.59209	163.8957	6202.549	4.622706
14126.72	0.044363	39.58629	63156.96	2.371775	44.13959	218.1149	8205.622	6.906803
466.7944	0.082009	12.9292	988.4986	2.60644	35.07361	76.71574	2764.411	3.017027
1214.386	0.067778	18.41141	3165.152	14.19646	2139.377	107.6204	232515	0.415089
594.673	0.071133	16.42319	1895.193	1.641977	33.82045	94.25526	2976.481	3.435943
2160.306	0.0741	15.35155	4180.503	11.18993	2185.7	77.17514	169957.9	0.259584
1327.155	0.07928	15.36414	1948.656	6.218463	94.70317	75.79665	7982.073	0.96769
421.4983	0.066294	18.13487	1346.124	2.057709	66.1156	85.78924	5583.107	1.420424
7600.206	0.052741	26.88304	38718.71	1.084582	47.46236	180.4277	8387.89	4.172353
1637.475	0.066313	23.1251	3120.767	5.894166	49.29405	110.8534	5389.944	8.223847
895.5336	0.060232	26.15086	2458.186	4.088325	33.12772	111.7967	2652.975	7.266949
1680.628	0.044713	36.80341	17202.05	0.434749	39.29606	236.8716	9149.175	6.248378
223.9798	0.05868	24.61087	1111.819	1.05066	26.1792	119.4857	3180.502	4.878765
539.5839	0.077415	14.12303	1319.393	2.37095	55.58766	74.12884	4277.397	1.443085
70.29383	0.084999	10.70469	215.4184	1.35293	24.4208	54.53325	1456.002	2.420481
641.8694	0.084169	13.06239	1156.827	3.596393	153.7119	69.1689	11091.48	0.465209
2070.522	0.055883	24.41445	8435.296	1.863879	1095.436	144.2724	157457.7	0.13302
2634.435	0.050941	28.29189	17159.11	0.984619	48.79751	168.4488	8356.695	3.553306
332.5967	0.057964	22.38901	2048.873	0.635806	17.12043	124.8508	2099.731	8.008775
115.5616	0.062398	19.11268	649.3413	0.66153	17.21922	114.0594	2108.677	6.761172
575.8337	0.082628	14.98429	819.4778	5.841161	51.38671	71.08265	3416.485	2.551782
1308.245	0.052965	27.25814	7498.407	1.024367	31.61198	171.6296	5309.013	6.082288
1769.057	0.049904	31.25721	10713.61	0.977323	58.95862	182.148	10513.66	3.278965
2672.69	0.066851	18.62967	6693.032	3.287677	74.68299	102.4634	7894.704	1.577381
904.7463	0.065358	19.0347	3169.158	1.73192	57.47649	111.256	6886.46	1.985194
171.8727	0.067746	18.34465	737.1364	1.020182	19.82302	87.03784	1665.493	5.12355
1191.418	0.067266	18.75703	3300.45	2.339633	35.65323	98.22572	3348.106	3.464094
79.68347	0.074401	16.32479	213.4034	2.337768	27.89169	78.85434	2025.922	3.92592
486.0206	0.050085	33.24228	3084.692	1.085192	44.22805	183.7646	8620.395	4.229727
832.1363	0.060815	23.9971	2437.65	2.431196	75.89074	115.4487	8880.557	1.682771
471.9212	0.064753	18.02137	1741.928	1.973442	70.08123	91.07602	6692.861	1.305641
2678.058	0.046756	36.76958	12125.28	1.686888	36.17475	195.9579	6659.637	7.230933
482.8199	0.064419	21.20368	1550.401	2.309292	42.90634	104.9891	4903.795	2.799906
3571.913	0.047549	32.65299	21979.3	1.172079	52.55132	190.795	10250.5	3.785267
879.3238	0.073424	14.68931	2248.536	2.131885	40.9651	83.66416	3523.829	2.387417
484.5876	0.060528	23.3871	1900.837	1.733261	38.12553	113.7724	4598.176	3.132511
68.34884	0.12227	5.332331	152.381	0.991689	8.849732	42.61002	346.8104	8.605999
1227.193	0.065363	17.67863	5518.588	1.105796	61.9134	100.4096	6300.652	1.663672
1079.901	0.064526	18.56999	3948.563	1.653779	71.25998	96.66252	7174.065	1.380592
130.7883	0.077758	13.71324	471.3948	0.956495	15.35315	72.07848	1190.992	5.3024
6492.593	0.053135	28.41766	26246.03	2.115227	1746.76	165.9237	285384.5	0.096951
537.9037	0.067686	17.184	1601.413	2.295212	62.8987	83.94526	5540.284	1.406061

2164.429	0.053277	27.09938	11124.05	1.254918	33.79708	149.7774	5138.059	4.798379
875.9874	0.05176	30.51609	4599.09	1.329355	45.17732	156.761	7240.486	3.637287
458.9897	0.060721	20.5032	2224.591	1.430119	50.74176	105.8853	5878.069	2.016942
553.1495	0.062929	21.71154	1912.825	1.871866	38.63629	111.0796	4725.958	3.10866
1993.914	0.058276	21.91708	9561.841	1.151328	78.67108	123.4138	10032.97	1.570546
355.6157	0.069551	17.14215	1056.31	1.857155	41.26697	85.78271	3838.722	2.16791
1879.533	0.077593	15.6021	3948.254	3.448876	67.85906	81.46964	5553.047	1.396124
717.4887	0.06232	19.98418	2568.574	2.101905	61.63815	99.03857	6482.978	1.643482
24824.19	0.04897	30.92462	169743.1	1.826819	57.66485	188.4296	10423.13	3.511712
849.0307	0.047559	34.94462	5928.391	1.268487	65.38685	159.9929	11029.86	2.386625
336.7964	0.06763	17.49969	1348.488	1.507723	53.69819	91.08715	5079.443	1.735851
12644.74	0.041667	42.94447	111608.9	0.859304	77.63216	271.4604	21492.85	3.500887
2618.836	0.05887	26.67669	7738.935	2.604921	37.30691	127.5187	3980.385	5.423039
3949.937	0.049607	30.977	18083.35	3.107561	58.38222	150.6731	7237.221	3.414211
88.86332	0.08931	9.776757	272.5618	0.997749	19.87437	65.22312	1254.829	4.159365
823.6007	0.066862	18.06811	2868.654	1.642987	53.42385	98.00779	5370.315	1.975718
3739.908	0.050803	28.65738	18934.16	1.435557	57.03319	159.8622	9186.341	2.949222
1598.629	0.056866	23.12068	7793.921	1.117737	44.25701	120.1648	5321.228	2.87424
404.485	0.078434	14.22191	1071.215	2.529293	49.0318	81.61974	4295.578	1.893798
404.2675	0.055723	25.5874	2105.659	1.102702	29.53784	135.177	4109.753	4.9063
2784.542	0.052234	32.06447	8756.723	2.857117	46.2367	159.8551	6208.733	5.396815
79.18361	0.086539	10.57299	271.3497	0.935679	24.04918	66.04699	1567.789	3.078908
4421.242	0.048287	31.44234	31552.56	0.697333	33.0092	202.6331	6606.765	6.57711
2017.6	0.053422	28.4054	6632.407	2.980825	58.02931	141.2019	7091.625	3.272688
50.72152	0.107007	8.473642	67.04219	5.325892	38.59494	36.20253	1844.549	1.520204
1232.247	0.056681	23.68082	5489.902	1.406795	33.57249	131.2828	4180.52	4.593173
4547.076	0.057231	24.41897	17731.58	1.635382	56.01373	149.1517	7855.658	3.102516
1439.458	0.063848	20.21729	4697.304	1.844753	23.69545	116.7988	2406.293	7.85933
1274.598	0.063287	19.16375	4915.618	1.691873	58.83391	101.2434	6285.889	1.745873
134.6785	0.089846	12.08357	274.1901	2.398504	33.91261	61.68312	1891.287	2.513575
823.8729	0.063093	19.72412	2407.591	2.750282	72.93613	93.12039	7048.692	1.361986
785.6424	0.053354	28.99108	3523.994	2.073403	62.86574	137.9308	9717.225	2.098344
648.2541	0.077164	13.87587	1410.335	3.121408	64.59291	69.5835	4194.853	1.310763
616.0298	0.049353	38.45136	2038.201	3.72311	54.33672	145.586	7688.234	3.338545
30.6824	0.131684	4.623957	42.82833	2.826245	46.7382	29.11159	1398.266	0.722318
633.1742	0.049332	32.22664	5920.451	0.415251	30.56806	220.5737	6207.585	8.069771
108.8865	0.081319	12.3915	370.5818	1.302282	27.74907	70.77296	2104.181	2.814906
2184.308	0.049951	30.40939	19512.58	0.416694	43.19079	244.3712	10590.45	5.790764
7275.712	0.052472	27.43844	31220.26	1.620304	52.87944	170.8364	9194.03	3.555081
720.1091	0.047057	33.44044	7217.468	1.207001	1796.805	244.08	438469.7	0.140985
2014.316	0.044502	37.98745	10660.84	1.952838	38.98427	219.325	7597.23	7.23771
344.5962	0.042548	46.30379	4601.467	0.319467	37.4408	257.484	9378.408	7.108294
260.8196	0.042389	46.12287	3030.708	0.512299	47.95043	225.1902	11013.74	4.63497
477.1813	0.047747	33.08601	4576.304	0.505493	22.39954	218.2682	5048.28	9.888007
1118.271	0.041599	43.70648	10174.54	1.064493	62.8204	220.313	14205.03	3.482211
8634.87	0.051055	28.30109	62220.59	0.68158	51.65688	192.2594	9908.756	3.82899
3164.971	0.046265	35.18085	26099.08	0.643796	29.23166	228.4603	6450.519	8.462049
287.5718	0.054413	25.63045	1793.506	0.887335	48.13377	132.1868	6345.843	2.84143
558.1021	0.046128	34.0121	5259.592	0.468935	10.82734	197.8644	1929.393	21.82026
201.358	0.055964	26.73629	970.3974	1.166847	38.18788	126.0039	5025.25	3.404313
4905.673	0.043531	38.6189	46346.76	0.870041	958.7086	248.1373	236974.6	0.260378
140.3844	0.057535	24.37658	778.8492	1.03294	56.89631	119.759	6720.873	2.203291
3674.878	0.054139	25.62847	24639.13	0.803427	56.3842	166.9963	9614.263	2.975425
71.34967	0.058292	28.87242	616.5131	0.373799	10.52614	158.8399	1520.216	17.36435
333.8913	0.05732	23.95423	2136.126	0.738411	24.64841	126.0215	3082.771	5.474376
534.3953	0.04768	33.82649	3542.745	1.347117	38.05398	172.3423	7156.572	4.444264

2794.013	0.048903	56.71424	28263.63	1.268281	820.6541	376.5151	312926.9	0.453744
10619.06	0.05337	26.18765	40476.98	2.265584	71.52383	150.7897	10321.22	2.405008
7601.338	0.044001	42.2866	76786.42	0.453829	39.64603	285.8595	11009.73	7.577865
4097.333	0.046119	34.85568	34655.12	5.29475	62.97833	222.4211	12376.83	4.098343
1722.182	0.045604	35.02069	12911.5	0.842982	65.11146	193.4829	12833.71	2.986566
694.5137	0.049079	30.31574	5785.337	0.56892	30.90955	193.0184	6243.161	6.191783
603.7515	0.046777	33.74657	4672.623	0.69226	36.69505	197.6528	7157.659	5.668515
1813.493	0.049042	31.33139	13835.93	0.649416	31.19403	221.9099	6636.642	7.784192
331.9112	0.052894	27.59493	2038.074	0.805796	15.22884	155.7445	2331.244	11.82543
325.9948	0.046758	35.612	2213.227	1.370255	38.37894	163.7705	7225.7	3.902212
710.6472	0.053089	27.14596	4017.89	0.944701	34.94547	161.9598	6009.528	4.715808
1169.023	0.053254	26.73296	7466.731	0.729277	19.33833	167.3097	3298.418	9.419782
172.5119	0.057754	26.10947	1040.476	0.757832	15.22431	138.9156	2204.977	9.630481
241.4271	0.049201	31.44364	1989.292	0.484958	8.9301	174.2089	1623.277	21.40116
1498.092	0.043024	39.73853	15756.75	0.466948	24.44966	248.5385	6181.354	10.29395
122.2679	0.055051	25.83123	896.1851	0.553914	19.00405	150.1824	2877.118	8.316176
462.0257	0.042772	46.66779	5937.314	0.327353	11.23838	314.1759	3149.125	32.77427
5531.646	0.047729	32.64516	19948.46	2.686817	62.44935	185.7088	11525.58	3.727305
3013.423	0.049974	29.36929	18654.22	1.215457	66.17211	167.5187	11307.69	2.561213
1545.022	0.053083	26.27774	8364.279	1.065244	36.03419	147.252	5404.786	4.286061
1349.101	0.0481	31.96641	11285.61	0.550196	42.7761	203.7812	8827.373	4.833473
6964.468	0.051485	27.74276	29261.21	2.35537	48.91989	166.4423	7146.605	4.398912
2309.06	0.052689	26.78754	18859.84	0.453549	41.69747	209.7072	8929.849	5.065794
20.08772	0.176208	2.866113	38.82456	0.620499	9	18.03509	149.8947	2.726472
437.0112	0.043828	41.42283	4647.123	0.528701	48.26487	185.7576	9107.463	3.82165
3364.364	0.049157	30.27286	21196.46	1.032242	56.8154	185.5813	10996.31	3.264411
4431.406	0.051459	27.88497	23341.25	1.320416	76.92891	180.4008	14086.93	2.440757
1329.49	0.046013	34.99975	9007.825	1.863052	111.7986	175.9445	20865.19	1.530617
81.62918	0.054638	27.23629	463.2905	1.248091	41.08835	123.5756	5454.185	2.935023
69.8227	0.0619	20.27731	365.1489	0.973932	30.22784	105.3812	2949.238	3.994515
1093.619	0.045628	35.82283	8554.111	1.229935	85.27403	213.3144	19120.72	2.437145
276.9931	0.053412	27.18874	1437.302	1.683891	84.48477	132.6845	11703.86	1.558663

AX67	AX68	AX69	AX70	AX71	AX72	AX73	AX74	AX75
0.06682	0.025973	0.518414	0.002701	3806.938	0.282112	1.033801	18.98247	5.77321
0.044813	0.012766	0.377105	0.000598	6268.762	0.287258	1.060533	27.8349	15.70675
0.030566	0.016935	0.553727	0.000659	5268.968	0.355395	0.618938	34.22284	12.50617
0.042714	0.045249	0.831228	0.005072	256.2497	0.308133	0.909278	26.49877	4.867421
0.040264	0.032973	0.979586	0.001841	722.3423	0.201808	1.869792	32.71321	4.7268
0.0533	0.019578	0.464771	0.001628	3315.731	0.31156	1.010143	23.31452	9.21648
0.016088	0.045815	2.927292	0.001255	1333.837	0.231981	1.67475	69.86438	3.024519
0.027297	0.032878	1.207963	0.001589	2476.935	0.256295	1.259431	41.83989	4.707863
0.064058	0.055523	1.050841	0.011383	187.3024	0.218868	2.104764	26.3378	3.465007
0.011521	0.056091	5.350107	0.000887	4106.509	0.137093	4.132244	104.2492	2.577168
0.022207	0.019506	0.866765	0.00055	30184.6	0.349335	0.676945	46.90263	10.49528
0.026942	0.014518	0.63739	0.00042	64475.74	0.289629	1.049713	41.41155	10.47868
0.038786	0.02467	0.734175	0.001608	4309.752	0.220228	1.63635	32.39675	5.574185
0.010456	0.051538	108.0844	0.00049	479.0223	0.124501	37.844	2137.319	2.896132
0.035126	0.037244	1.029791	0.002601	313.8173	0.307677	0.946271	32.26983	4.418505
0.02118	0.019337	0.943309	0.000523	15594.06	0.256679	1.201514	51.83512	7.530601
0.001492	0.030736	59.29057	0.000286	454.34	0.1627	9.569196	1959.002	4.594506
0.027668	0.025042	1.240871	0.000795	5135.169	0.200644	2.946427	47.53921	5.748327
0.01437	0.055181	5.325028	0.000872	3628.904	0.124242	5.288649	91.23121	2.808027
0.02011	0.027969	1.642525	0.000703	11420.63	0.162603	2.892251	64.02408	5.376846
0.026367	0.016582	0.759254	0.000483	20329.82	0.245463	1.317116	44.04333	7.425913
0.020887	0.038827	2.411182	0.000978	4291.258	0.144096	3.555252	63.80411	3.56803
0.065363	0.024154	0.340996	0.003484	776.4514	0.348615	0.704208	17.69515	8.922653
0.016718	0.017419	1.252439	0.000298	15638.01	0.205973	1.878297	68.75106	7.962442
0.026751	0.022789	0.895981	0.001102	6108.324	0.254944	1.359814	43.47093	7.330672
0.00074	0.03478	70.32722	0.000271	575.5021	0.213745	2.560942	2056.679	4.244183
0.056687	0.013694	0.258538	0.000915	3881.023	0.413363	0.463842	19.46085	12.03808
0.033494	0.034796	1.653512	0.001194	2958.182	0.142228	3.88072	46.45341	4.059981
0.062702	0.024571	0.498177	0.001966	1435.347	0.266659	1.098671	20.53365	5.538913
0.034121	0.036122	1.270273	0.001773	2177.495	0.187233	2.162864	38.54153	4.061438
0.026308	0.047184	1.960594	0.002401	548.3635	0.226196	2.292911	50.24618	3.384363
0.044836	0.023906	0.760855	0.001365	7645.407	0.201889	2.033058	30.60367	5.626889
0.018916	0.039154	2.150802	0.001761	821.3663	0.23538	1.665966	61.99352	3.628826
0.030787	0.031429	1.706907	0.000928	4645.828	0.147359	3.990639	49.1259	5.790424
0.025406	0.016419	0.851473	0.000425	50223.27	0.220458	2.313951	47.99888	8.500381
0.025347	0.029399	1.16365	0.001079	855.8933	0.220874	1.603336	44.69674	5.402598
0.028923	0.020579	1.10715	0.000548	15104.29	0.156791	3.060876	49.60804	6.607644
0.062831	0.035027	0.894862	0.003056	621.5056	0.213704	1.817115	23.48168	4.126272
0.015162	0.04464	2.510542	0.001369	1288.418	0.2265	1.84337	73.55627	3.892851
0.018315	0.035663	1.991375	0.001371	313.9399	0.221731	2.068737	64.26007	4.246009
0.014068	0.043534	3.258723	0.000834	3009.486	0.184345	2.511599	81.63472	3.790155
0.013985	0.037847	3.984478	0.000553	21761.18	0.135022	5.320009	93.98452	3.837449
0.04996	0.046413	2.138562	0.003411	1175.466	0.123077	5.03788	41.43671	3.14175
0.016508	0.023857	1.796437	0.000443	11211.18	0.172962	2.750986	73.19738	5.772059
0.015388	0.022784	1.804941	0.000397	7601.761	0.227818	1.795498	73.13391	6.083338
0.026418	0.04499	1.590588	0.002781	758.7667	0.187753	2.482273	48.11169	4.479736
0.047475	0.02588	0.522008	0.002307	1446.971	0.292319	0.953875	24.19959	8.22173
0.060291	0.033195	1.119556	0.002366	4078.081	0.141721	3.752779	34.9595	4.274906
0.038099	0.030514	1.082446	0.00139	3270.272	0.182479	2.250904	35.82928	4.284363
0.013384	0.027644	2.32555	0.000494	3694.717	0.170242	2.895061	87.78739	5.264437
0.016325	0.021918	1.349885	0.000459	10247.25	0.25927	1.313442	66.07859	7.085586
0.041194	0.04025	0.955148	0.002927	603.5584	0.228972	1.525872	30.28352	4.035386
0.030864	0.039682	1.654527	0.001768	1042.281	0.168099	2.687745	43.79397	3.443812
0.011168	0.041227	3.603126	0.000701	2130.359	0.179534	2.646519	101.0855	4.06833
0.021438	0.020973	1.134167	0.000619	12097.89	0.220019	1.752223	53.35545	7.544198
0.013046	0.025764	1.893062	0.000553	5002.682	0.236521	2.026779	85.3154	6.547057
0.054413	0.018689	0.400894	0.001349	2927.462	0.285052	0.961414	22.18394	7.783645

0.032351	0.03042	0.972705	0.00149	914.015	0.301619	0.960635	34.0458	5.448252
0.080763	0.030129	0.526668	0.005179	651.7165	0.213749	1.689521	19.25829	5.031512
0.019325	0.035598	1.721387	0.001117	1884.589	0.221851	1.717728	58.19315	5.508783
0.020408	0.016113	1.015481	0.000332	59737.33	0.234146	1.642036	56.78623	9.675936
0.049153	0.041935	1.365526	0.00229	611.2016	0.164461	2.810472	33.598	3.731791
0.020761	0.029078	1.536936	0.000762	4846.152	0.24951	1.532413	54.23961	5.440209
0.016125	0.030897	2.358386	0.000572	8962.575	0.161506	3.261143	75.75355	4.813121
0.01501	0.040837	3.74312	0.00068	8067.801	0.137147	4.390697	87.26913	3.755039
0.030008	0.043904	1.632367	0.0022	707.0624	0.180386	2.440342	43.9719	3.356002
0.020555	0.058036	2.840867	0.00227	380.975	0.198075	2.146502	58.17483	2.810084
0.025634	0.044217	2.398363	0.003156	407.3988	0.13895	4.44651	63.0336	3.365275
0.019921	0.028265	1.575619	0.000655	9199.968	0.226891	1.479886	57.12072	4.845724
0.019928	0.019619	1.128436	0.000415	48079.63	0.281437	1.141319	55.03075	6.754926
0.054587	0.03315	1.846865	0.001697	12042.29	0.13211	7.086897	41.68941	4.642467
0.016291	0.02588	2.126923	0.000438	22445.06	0.183671	3.509717	75.81931	6.936904
0.000649	0.016029	30.57521	2.09E-05	19941.16	0.322921	1.372506	1905.492	9.84222
0.075356	0.032076	0.439919	0.005469	283.7754	0.299868	0.911741	16.86357	4.607649
0.035191	0.02768	0.892403	0.001265	2466.031	0.238017	1.405039	34.54574	6.728391
0.014235	0.019287	1.418734	0.000313	20414.69	0.296035	1.032549	74.40822	8.692973
0.05922	0.030584	0.650908	0.003014	2232.284	0.24696	1.261311	22.39964	4.53699
0.022868	0.037683	2.288927	0.001002	8684.227	0.181795	2.600758	54.92615	3.753934
0.028242	0.025909	1.010335	0.000931	2358.676	0.207415	1.752884	42.08202	6.228844
0.014161	0.03564	2.907436	0.000602	9077.579	0.17019	2.738122	81.3302	3.340729
0.016579	0.0383	3.174578	0.000787	4250.554	0.133154	4.51166	92.75572	4.276956
0.017737	0.040004	2.365516	0.001053	2944.851	0.247803	1.443239	62.50256	3.407304
0.030885	0.029171	1.573661	0.001033	9831.1	0.180297	2.867713	43.30004	4.30843
0.034558	0.015257	0.494408	0.000613	51471.3	0.371691	0.764303	31.28993	10.12445
0.03757	0.03978	1.390211	0.002171	3008.179	0.175713	2.369241	36.70057	3.355749
0.029579	0.034609	1.438172	0.00179	8336.776	0.184634	2.388561	43.88817	3.41332
0.032462	0.035931	1.33224	0.001757	2315.781	0.170613	2.550082	41.9282	4.336662
0.029922	0.053603	2.208779	0.00419	332.3621	0.152583	3.51033	50.8132	2.728146
0.016153	0.048199	3.590928	0.001066	3929.497	0.134254	4.306501	80.65042	3.000691
0.027616	0.028385	1.466387	0.000781	9477.104	0.205331	2.308892	47.15064	5.172662
0.032769	0.021993	1.240609	0.000747	11192.35	0.232125	2.589475	38.68467	7.401232
0.035847	0.033763	1.38348	0.001466	1936.379	0.154618	3.117279	40.85268	4.260035
0.019929	0.028574	2.044686	0.000589	8700.183	0.160774	3.766475	64.96573	4.916539
0.02725	0.040788	1.734703	0.001643	825.4031	0.182518	2.270986	45.58119	3.051606
0.023082	0.044164	3.778854	0.000935	4638.305	0.132506	5.398974	64.7308	3.681665
0.021396	0.042976	2.092162	0.001472	431.3485	0.270602	1.214642	52.92473	3.573954
0.001406	0.033255	63.54382	0.000264	797.3786	0.258562	7.218701	1944.024	4.031891
0.028664	0.033985	1.349205	0.001541	2063.393	0.180805	2.336084	44.39893	4.11372
0.022593	0.023733	1.183087	0.000645	5951.232	0.226689	1.554052	50.26558	5.740708
0.036416	0.037569	1.201717	0.0023	1234.211	0.209625	1.896958	35.96906	4.037175
0.028061	0.030349	1.414066	0.001005	7897.841	0.166058	2.846426	48.29125	4.292784
0.031496	0.028938	1.032745	0.001447	3140.58	0.216324	1.726768	39.21854	4.833791
0.024383	0.047814	2.26636	0.001862	1288.89	0.152874	3.327257	55.59124	3.365007
0.018585	0.057276	4.177892	0.00173	1806.26	0.206167	2.683807	67.20822	2.884759
0.023972	0.025951	1.083767	0.001028	4186.08	0.285318	1.147912	45.86729	6.532127
0.037009	0.028907	0.811116	0.001855	1207.432	0.313234	0.914328	30.77069	4.983272
0.068261	0.026012	0.463342	0.003387	1697.673	0.262055	1.15057	19.60589	5.209621
0.016542	0.029013	2.000689	0.00058	11448.68	0.177578	2.560757	71.95643	4.326607
0.022597	0.024078	1.294832	0.000591	30226.99	0.214572	1.81425	51.43779	5.501968
0.034795	0.022885	0.799804	0.000994	13787.78	0.247042	1.376688	34.52319	6.51828
0.009106	0.037815	3.991423	0.000539	2002.574	0.193712	2.763925	120.5361	3.839171
0.016659	0.030103	1.907874	0.000836	3199.355	0.210465	2.041393	70.41055	5.366417
0.043553	0.019812	0.636903	0.000907	17029.89	0.248327	1.345755	29.96636	8.359248
0.027861	0.034426	1.39888	0.001361	3296.686	0.239619	1.518011	42.30166	3.807336
0.026945	0.029152	1.291694	0.00106	7546.429	0.20164	1.991551	45.56869	4.240361

0.022822	0.037436	1.924673	0.001112	3328.168	0.207774	1.819957	51.34695	3.058767
0.073415	0.029677	0.698166	0.00261	1635.028	0.195474	2.236189	22.24517	4.358411
0.011569	0.03047	3.098574	0.000467	10855.54	0.138613	4.355058	104.1451	6.198445
0.009229	0.03208	3.348369	0.000438	1307.439	0.202088	2.220171	118.0561	4.700669
0.03735	0.054317	1.829282	0.00278	1833.683	0.222716	1.578216	33.16434	2.591638
0.018264	0.02435	1.829134	0.000468	12125.7	0.143609	3.75008	71.34941	6.009528
0.067561	0.035394	0.938842	0.002822	1438.581	0.187501	2.097225	24.24128	3.975907
0.06077	0.025684	0.489772	0.002564	7417.153	0.341095	0.752476	19.6133	5.804445
0.023026	0.03019	1.601076	0.001051	5159.449	0.165309	3.119361	57.03248	3.725474
0.014774	0.042397	3.296661	0.001028	2028.93	0.128082	5.22499	92.87402	3.625177
0.009721	0.036383	4.420315	0.000399	4363.877	0.139624	4.256378	123.0628	3.980727
0.039298	0.0372	0.955965	0.002708	2320.527	0.268676	1.173191	30.19443	4.387015
0.001581	0.023304	46.02813	5.27E-05	7263.829	0.214203	6.120764	1978.414	6.126959
0.023932	0.026104	1.465287	0.000867	6852.297	0.135355	4.327398	61.13112	5.909236
0.01828	0.028811	1.669982	0.000779	9088.563	0.224087	1.856294	62.82583	5.746862
0.025421	0.040263	2.164955	0.001458	2116.95	0.129075	4.481423	59.20976	3.728485
0.028856	0.029734	1.152053	0.001085	8645.226	0.26497	1.128729	38.89358	4.764782
0.028081	0.01905	0.94885	0.000542	30144.46	0.179647	2.535203	47.56952	9.680402
0.045976	0.04082	1.314589	0.003106	707.7525	0.169438	2.723267	34.73547	3.598479
0.006213	0.029861	62.69784	0.000126	2064.022	0.170873	13.9687	2134.615	5.0535
0.036742	0.035193	1.273958	0.001739	1248.676	0.212597	1.805615	34.45199	4.054617
0.00399	0.051195	107.7612	9.30E-05	2979.972	0.13878	10.82784	2180.349	3.189343
0.015483	0.046248	3.757901	0.001	1394.363	0.112371	6.359815	92.11478	3.616919
0.018483	0.0637	3.747634	0.002105	905.9245	0.197158	2.336947	66.06679	2.794108
0.023185	0.018734	0.902744	0.000494	20354.94	0.250863	1.227741	47.95724	7.126388
0.050413	0.032265	1.514442	0.001641	2120.345	0.127103	5.145158	49.29277	5.126461
0.05069	0.042784	1.69074	0.001926	1526.836	0.150099	4.065758	35.84834	4.13943
0.026798	0.023064	0.815967	0.000857	6876.153	0.368982	0.632425	39.52272	8.133214
0.0492	0.037883	0.965157	0.003667	649.6931	0.254854	1.286114	26.1154	4.926587
0.023268	0.053374	2.855564	0.002188	930.0135	0.179915	2.628979	55.07524	2.766114
0.064115	0.083088	1.569839	0.00931	159.8967	0.245483	1.475708	23.86804	2.408328
0.007542	0.049161	6.822144	0.000509	840.7299	0.147703	3.783293	152.2074	3.299801
0.000987	0.026452	28.33547	4.64E-05	4987.273	0.216727	2.306692	1096.199	5.643172
0.023274	0.028195	1.310787	0.000867	8572.251	0.284707	1.24873	48.38119	5.426077
0.070039	0.037546	0.678106	0.003863	1170.048	0.313818	0.775332	17.20873	3.596517
0.071831	0.03535	0.523951	0.004897	396.6004	0.320714	0.767871	16.72968	4.267458
0.035514	0.050231	2.637931	0.002229	605.4016	0.115869	5.721999	52.1041	3.360426
0.039066	0.021482	0.70848	0.001077	3855.299	0.270181	1.2552	31.96416	7.058551
0.018298	0.022148	1.289159	0.000496	5358.231	0.265967	1.223905	59.33139	8.755855
0.018859	0.033161	2.206679	0.000872	4357.219	0.159339	3.609732	73.8521	3.852876
0.020973	0.031288	1.587596	0.00099	2013.569	0.217066	1.899398	56.09407	4.349219
0.068448	0.056918	1.226625	0.005113	482.8868	0.263805	1.182448	20.01356	3.069985
0.040244	0.040697	1.465469	0.002604	2117.958	0.172037	2.609207	36.05569	3.464032
0.063803	0.051357	1.461591	0.004732	139.5966	0.177349	2.615031	28.19826	3.478416
0.028821	0.02143	0.846705	0.000834	1548.7	0.280272	1.366685	42.956	9.958899
0.015486	0.036526	2.460936	0.000859	1588.639	0.172751	2.603971	75.20971	4.484192
0.017469	0.059828	3.576901	0.001945	1096.962	0.212057	2.342386	68.95227	2.856286
0.034128	0.022301	0.797343	0.00091	6736.248	0.212542	1.661114	37.07648	8.639355
0.037681	0.041025	1.484119	0.00231	976.8724	0.189824	2.568111	41.66734	4.572525
0.021663	0.02288	1.101213	0.000759	10575.12	0.253802	1.446621	51.88206	6.673946
0.032415	0.043383	1.648369	0.002066	1569.758	0.182191	2.265577	40.61595	3.39854
0.036099	0.04091	1.405936	0.002646	1127.883	0.209576	2.047151	37.41548	4.033207
0.219774	0.059529	0.582493	0.015011	120.478	0.267133	1.026985	8.95026	3.673693
0.017908	0.04396	2.734045	0.000994	3459.458	0.267434	1.306847	61.68619	3.084108
0.015993	0.049033	3.20815	0.001119	2564.418	0.219159	1.883786	70.38289	3.162947
0.089261	0.050142	0.73362	0.005835	340.3615	0.271078	1.022827	15.08717	2.717965
0.000605	0.02165	37.88183	2.25E-05	14076.93	0.195293	2.368486	1757.626	6.878249
0.020526	0.059125	3.344011	0.00196	1091.312	0.189409	2.579434	61.98886	2.922436

0.036556	0.029785	0.916793	0.001729	6095.223	0.245189	1.501377	33.56022	5.363466
0.025918	0.027681	1.143595	0.001076	2424.991	0.235417	1.630221	44.79122	6.729926
0.024432	0.05101	2.204447	0.002266	1328.507	0.257653	1.695856	49.24011	3.207517
0.035811	0.035733	1.136193	0.002309	1204.195	0.201768	2.041018	37.27546	4.73942
0.013693	0.038543	2.852703	0.000674	5729.698	0.257177	1.356182	77.76285	3.780885
0.031446	0.05309	1.951196	0.002626	720.2585	0.195441	2.040957	40.38031	3.102077
0.019994	0.040617	2.714075	0.001114	2704.789	0.152573	3.857448	67.93275	4.244168
0.02019	0.052741	2.651629	0.001787	1612.49	0.20128	2.420443	60.37879	3.259802
0.01915	0.031775	2.911184	0.000667	79935.28	0.283628	2.864215	59.43755	5.545968
0.01744	0.038072	2.051593	0.001134	2989.433	0.273936	1.687381	63.56137	5.419879
0.022493	0.045139	2.222023	0.001808	866.31	0.24411	1.774805	52.97846	3.24844
0.013689	0.0166	1.158722	0.00032	38424.33	0.276518	1.295994	76.18974	13.53422
0.037528	0.031523	1.248836	0.001474	4963.007	0.170735	2.663709	39.07964	5.082938
0.020913	0.036614	2.511961	0.000907	9243.523	0.188165	3.777833	62.61826	4.535952
0.065324	0.04562	0.931569	0.003829	201.7066	0.267947	1.051803	19.9181	3.457698
0.022272	0.039438	1.976646	0.00123	1871.49	0.218977	1.817803	52.99536	3.649578
0.020246	0.028875	1.520055	0.000837	9975.391	0.226918	1.775551	56.6607	5.356977
0.0257	0.043706	1.828279	0.001595	4672.241	0.251801	1.321914	44.12707	3.591972
0.031634	0.038846	1.68136	0.00286	733.1408	0.196517	2.777833	48.03274	4.409486
0.044246	0.031677	0.824241	0.002403	1189.589	0.256313	1.345568	29.13962	5.557352
0.028571	0.024118	1.209173	0.000716	5275.802	0.163879	2.835549	48.94949	6.476378
0.051697	0.046107	1.176944	0.003484	191.3385	0.276427	1.050865	24.07943	3.955623
0.033259	0.020657	0.688406	0.000808	15127.42	0.306972	0.866904	33.20974	7.347266
0.02138	0.031408	1.95984	0.00081	3875.6	0.162302	3.243539	60.78844	4.640944
0.076736	0.129412	4.027753	0.015951	52.71829	0.132153	5.311642	37.00663	2.258921
0.038166	0.033832	1.123842	0.002093	3166.685	0.227906	1.668878	34.10368	4.314572
0.020553	0.021766	1.220367	0.000586	10208.76	0.21106	1.820576	57.1589	6.400313
0.060092	0.030492	0.760617	0.002406	3035.935	0.20339	1.860179	24.57255	4.411986
0.020525	0.04457	2.334739	0.001478	3093.446	0.222587	1.961394	57.78699	3.298778
0.043001	0.055636	1.83003	0.004742	201.9135	0.174816	2.578554	34.36241	2.905215
0.017592	0.064255	3.850275	0.002177	1592.951	0.171915	3.123739	71.82845	2.89312
0.019819	0.035044	1.718628	0.001258	1923.734	0.20278	2.496235	59.65447	5.572588
0.019993	0.060551	3.781827	0.0021	1005.474	0.159044	3.43184	65.38439	2.567147
0.02886	0.04821	1.884921	0.00285	1115.072	0.13918	4.302506	53.75853	4.925239
0.039019	0.099857	3.901983	0.006711	35.01199	0.17259	3.189003	46.31787	2.664623
0.034582	0.015826	0.550754	0.000629	2739.287	0.415141	0.52553	32.17834	11.50595
0.050141	0.052922	1.192806	0.00411	262.6192	0.259263	1.436821	27.15841	3.518443
0.024151	0.015755	0.672425	0.000494	8518.233	0.407555	0.553451	43.16275	9.685149
0.021901	0.02043	1.0351	0.000576	16792.54	0.209532	1.808912	52.48804	6.49668
0.000932	0.017808	31.20746	5.99E-05	3202.065	0.434326	2.129724	1795.538	8.651627
0.031475	0.019034	0.758994	0.000724	4597.557	0.194185	2.241859	41.21974	9.461109
0.028143	0.029706	1.059412	0.001149	1558.98	0.397588	0.560197	38.172	9.224135
0.022284	0.03683	1.602696	0.001276	1099.41	0.337803	0.839879	47.15673	7.726535
0.051099	0.018124	0.445635	0.001195	1932.29	0.374185	0.711619	22.07719	9.241942
0.0181	0.03117	1.722846	0.001041	3761.83	0.264001	1.654531	61.53641	7.94369
0.02062	0.025468	1.294718	0.000662	28646.66	0.310116	0.90811	51.63456	5.649983
0.037926	0.01501	0.438237	0.000733	11047.16	0.319901	0.880988	29.71681	12.51983
0.023569	0.041594	2.055479	0.001235	973.2208	0.286068	1.145566	48.22435	4.172282
0.112285	0.025603	0.334466	0.003746	2332.017	0.35145	0.63939	11.43606	6.867839
0.030834	0.041911	1.505885	0.002052	578.0036	0.244184	1.369362	37.61271	4.318293
0.001091	0.020502	19.413	3.82E-05	17974.38	0.330977	1.438712	961.1085	9.376544
0.020072	0.042255	2.267218	0.001633	464.3237	0.285243	1.31544	56.99389	4.537471
0.019214	0.030397	1.604411	0.000832	12343.39	0.312917	1.070713	55.76384	4.873946
0.111831	0.028447	0.392011	0.004065	309.7514	0.410366	0.525407	11.12264	11.06991
0.048858	0.036103	0.886695	0.002623	1190.275	0.312458	0.9189	24.7516	4.849852
0.0376	0.027692	0.840668	0.002344	1707.2	0.260631	1.788384	36.26202	6.566767

0.001252	0.020581	16.20744	5.13E-05	5929.149	0.294337	2.642162	806.0124	22.15562
0.016338	0.030134	2.32937	0.000642	23002.52	0.189594	2.682511	72.81153	4.830802
0.026468	0.019679	0.811829	0.000564	25745.32	0.339824	0.67764	40.52398	11.52388
0.018705	0.027228	5.777817	0.000534	14978.35	0.328519	10.02561	70.19894	7.82827
0.016423	0.029153	1.773104	0.000701	5953.406	0.284012	1.134144	64.36808	6.341002
0.03626	0.023487	0.653638	0.001578	2745.175	0.353248	0.761118	30.10965	7.035386
0.030151	0.023959	0.85826	0.00128	2139.233	0.300726	0.947993	36.93704	8.318087
0.034996	0.018029	0.617033	0.000757	6126.3	0.325312	0.869538	32.00146	9.276284
0.086149	0.026256	0.534411	0.002632	1066.48	0.279703	1.022574	15.51545	6.143339
0.033802	0.041899	1.050692	0.003484	1076.876	0.254958	1.715484	35.52434	5.11366
0.033358	0.022912	0.715974	0.001132	2218.605	0.277455	1.109387	33.97024	7.028858
0.063507	0.021633	0.3952	0.002251	3936.292	0.305877	0.894785	19.14456	7.20412
0.091155	0.025723	0.335372	0.005432	547.137	0.287045	0.970862	14.83846	7.290526
0.148938	0.027436	0.229383	0.007266	1011.198	0.354491	0.594981	8.741811	6.043449
0.045173	0.017184	0.382015	0.001187	5684.32	0.337008	0.701129	24.14308	11.24033
0.061351	0.025762	0.45683	0.002391	492.4885	0.359413	0.71327	18.85342	6.01641
0.100059	0.014362	0.194981	0.001855	1913.735	0.428081	0.503265	12.52012	17.70301
0.019718	0.02634	1.484519	0.000719	11115.46	0.17008	2.667729	62.31122	6.929035
0.016989	0.027857	1.637306	0.000749	9076.013	0.256863	1.61454	65.3424	5.448597
0.032348	0.032768	1.132647	0.001527	4507.072	0.25179	1.305812	35.74689	4.749731
0.024887	0.027722	1.130259	0.000985	5272.507	0.351166	0.747243	42.439	6.477509
0.024723	0.025372	1.401268	0.000664	14652.05	0.185142	2.635247	51.54538	5.235463
0.025303	0.017262	0.69556	0.000545	9000.783	0.396762	0.571885	41.22318	7.340583
0.153494	0.136846	1.35215	0.0256	23.59223	0.323432	0.695138	9.323424	4.470614
0.022479	0.039534	1.794461	0.001394	2015.02	0.348411	0.788724	47.79058	5.859788
0.019964	0.022842	1.1458	0.000728	10248.74	0.2669	1.339181	55.46343	6.787221
0.014428	0.02357	1.696936	0.000482	11874.64	0.243976	1.576359	76.29313	6.14323
0.011241	0.03772	3.35067	0.000792	4186.942	0.248218	2.593307	107.9957	5.565395
0.03014	0.050399	1.835004	0.003515	261.4092	0.262414	1.579246	40.03833	4.006977
0.041028	0.046998	1.552983	0.004735	220.3211	0.28173	1.207888	30.89777	3.962002
0.013473	0.022066	1.509414	0.000585	3806.616	0.298965	1.814041	82.24257	8.747728
0.014053	0.043971	3.036023	0.001188	816.2834	0.243183	2.161213	82.60775	4.790891

AX76	AX77	AX78	AX79	AX80	AX81	AX82	AX83	AX84
103.1788	0.374586	0.06859	3.594352	6836.123	0.468607	0.613275	2.083841	0.684924
361.9064	0.761829	0.042103	4.126369	8357.717	0.338681	0.473145	7.325616	0.558747
437.8071	0.369174	0.031648	3.734411	5545.939	0.343795	0.472811	5.800741	0.585078
143.1959	0.184254	0.047196	3.170925	477.1379	0.530689	0.668504	1.649617	0.732068
157.5925	0.18496	0.041734	3.845049	2011.11	0.518413	0.661965	1.613252	0.727204
190.0199	0.494756	0.054236	3.807116	4473.5	0.387297	0.529838	4.050424	0.621517
211.7744	0.046963	0.016536	3.570895	3404.71	0.570994	0.710144	0.811841	0.773324
196.8205	0.123742	0.028383	3.71828	4706.427	0.463634	0.619077	1.579395	0.692871
102.32	0.176669	0.074465	3.532902	508.8045	0.566749	0.707718	1.023056	0.766019
269.1875	0.029202	0.011707	4.108921	19591.5	0.628865	0.757033	0.629223	0.810249
496.7291	0.227174	0.02292	3.79567	30029.47	0.33561	0.449526	4.862408	0.586704
409.3355	0.285087	0.026852	4.141003	80697.56	0.338569	0.475433	4.58708	0.576602
177.4272	0.212108	0.039842	4.014864	9548.216	0.453316	0.604094	1.964084	0.677572
6197.863	0.03134	0.010807	4.197126	2624.019	0.638958	0.757436	0.779908	0.809145
149.6997	0.142823	0.037242	3.18209	599.3443	0.544608	0.686536	1.428256	0.741296
382.1113	0.159215	0.021477	4.049031	25250.01	0.389327	0.533089	2.984856	0.62565
8946.199	0.005086	0.001762	4.041273	1662.909	0.543244	0.683034	1.523997	0.736801
232.0216	0.167118	0.026657	4.287279	11905.17	0.440523	0.578076	2.152663	0.672559
245.8811	0.040638	0.014401	4.294835	19171.69	0.62655	0.749809	0.754359	0.806791
317.8185	0.119729	0.019447	4.324632	34716.59	0.468589	0.603326	2.063034	0.696147
305.701	0.199675	0.025914	4.14717	32208.31	0.367852	0.515351	2.84723	0.606063
213.1386	0.078155	0.020385	4.24287	16689.88	0.536464	0.677356	1.078449	0.748022
158.449	0.544468	0.071682	3.450949	924.8038	0.385329	0.538329	3.745436	0.620674
500.9153	0.138399	0.016272	4.255025	34719.38	0.415653	0.559374	3.170502	0.638243
311.9984	0.190632	0.027928	4.013455	10507.51	0.407092	0.556566	2.872913	0.639185
8818.738	0.002455	0.000895	3.728498	1550.269	0.536807	0.680758	1.390513	0.741984
227.8588	0.677544	0.057861	3.569568	3378.306	0.327956	0.470072	5.117531	0.558412
172.7614	0.145735	0.032109	4.246459	12836.17	0.569119	0.700803	1.33478	0.760827
109.1197	0.340019	0.063329	3.727013	2563.291	0.445262	0.600789	1.94297	0.670345
148.1726	0.140438	0.03433	3.959925	6517.307	0.526793	0.668366	1.303113	0.73705
182.4999	0.078642	0.028742	3.598907	1496.376	0.582867	0.713922	0.992879	0.773127
158.3304	0.257168	0.044251	4.171682	18048.46	0.445722	0.595539	2.014504	0.672341
228.3664	0.064622	0.019905	3.658294	1954.39	0.532307	0.677937	1.067595	0.74309
221.7322	0.195199	0.02893	4.348579	17827.59	0.52382	0.639765	2.432368	0.729382
365.5936	0.220028	0.024944	4.437972	88522.74	0.366162	0.500152	3.563794	0.605969
271.6369	0.122791	0.027245	3.65985	2259.484	0.528102	0.665532	1.947703	0.721357
273.7525	0.213238	0.026694	4.469654	45413.82	0.437763	0.576599	2.606821	0.664395
83.74727	0.27642	0.061311	3.809784	1560.048	0.508942	0.659363	1.346265	0.726275
309.4286	0.05373	0.016317	3.669063	3217.323	0.538516	0.675767	1.265586	0.748067
300.2282	0.068232	0.020091	3.669255	827.8779	0.544878	0.685034	1.349769	0.744051
309.8708	0.051753	0.014451	3.985385	9518.443	0.550911	0.685904	1.205595	0.755382
321.3199	0.056806	0.013506	4.511953	89243.29	0.530286	0.665607	1.239095	0.744192
111.4071	0.169176	0.049304	4.274821	6062.982	0.603359	0.731571	0.938075	0.791839
396.2713	0.097987	0.01633	4.30635	34105.35	0.481588	0.625524	2.135415	0.69568
428.4826	0.093619	0.015521	4.105306	15015.84	0.423586	0.57148	2.225366	0.656546
236.3751	0.102495	0.029394	3.888772	2418.963	0.559322	0.683502	1.624074	0.75834
207.2216	0.359445	0.051796	3.724901	2215.233	0.410349	0.54579	3.335899	0.639665
135.6225	0.319186	0.05659	4.204058	17880.91	0.569052	0.699191	1.506536	0.760649
147.3661	0.166829	0.037907	3.970429	10424.95	0.535109	0.677242	1.362231	0.735253
450.8169	0.06969	0.013635	4.266826	11957.03	0.505127	0.645178	1.853585	0.711722
456.9874	0.115311	0.016534	4.019674	16734.97	0.398671	0.545417	2.804817	0.635389
132.7972	0.150043	0.044462	3.594596	1503.897	0.533979	0.67607	1.26486	0.740016
145.0257	0.106091	0.031259	3.953175	3683.761	0.563592	0.700857	1.031804	0.764622
434.7305	0.042312	0.011748	4.013575	6963.124	0.550552	0.684616	1.350192	0.752642
402.0632	0.156441	0.022418	4.227776	23459.37	0.398885	0.541723	3.084169	0.634759
587.3225	0.079069	0.014059	4.003583	10568.51	0.459128	0.596873	2.529178	0.678564
161.8729	0.422602	0.05474	3.847313	4266.11	0.385591	0.535981	3.069993	0.619631

191.4665	0.166621	0.034271	3.494324	1455.31	0.456317	0.592293	2.032131	0.68716
94.60558	0.390744	0.08533	3.856301	1641.022	0.495724	0.644882	1.728322	0.709809
331.944	0.10114	0.020298	3.884322	4516.176	0.491654	0.633947	2.075613	0.708431
495.5217	0.204961	0.019841	4.345171	99461.64	0.362353	0.503868	4.185334	0.599082
125.4925	0.191582	0.048971	3.818632	2558.135	0.624667	0.74191	1.171187	0.78915
284.6838	0.112165	0.021033	3.846237	9718.853	0.470067	0.616514	2.006276	0.695329
336.8201	0.079727	0.016005	4.35469	28819.22	0.491474	0.63209	1.739787	0.713883
287.4134	0.061234	0.014488	4.361161	33576.34	0.547228	0.678266	1.256818	0.75666
157.2342	0.092245	0.031995	3.813774	2440.18	0.585677	0.718669	0.978395	0.776243
173.1289	0.053534	0.021637	3.60212	1224.278	0.608976	0.743232	0.725579	0.796252
215.0314	0.076127	0.028127	4.233471	1813.091	0.580256	0.718477	0.972049	0.77213
258.2544	0.099775	0.019628	3.912555	19001.2	0.451073	0.599758	1.684507	0.684943
351.707	0.13615	0.019772	3.944311	67012.96	0.377918	0.523282	2.612186	0.62217
133.8386	0.297686	0.048761	4.634138	48160.66	0.503934	0.634645	1.700391	0.724172
446.2047	0.119446	0.015735	4.489159	55204.09	0.431419	0.554981	3.042631	0.669833
18756.06	0.005681	0.000738	3.89782	22549.45	0.343263	0.482454	4.229548	0.583189
80.77737	0.316387	0.081328	3.234564	539.3186	0.523993	0.673837	1.464593	0.722849
215.3179	0.244869	0.035399	3.904363	5150.587	0.459077	0.600048	2.797593	0.686326
639.9333	0.12268	0.014419	3.98796	26862.58	0.364904	0.512648	3.65409	0.606163
97.20803	0.262211	0.060784	3.708005	4592.477	0.481308	0.629209	1.485473	0.703522
185.0811	0.088849	0.022392	4.041335	25811.6	0.519972	0.662979	1.17705	0.737883
275.58	0.166839	0.029681	3.932538	6112.804	0.486105	0.62914	2.341089	0.696003
265.6036	0.047373	0.014202	4.157559	26718.21	0.496868	0.652515	0.942776	0.728856
389.8311	0.074375	0.016782	4.387148	18836.06	0.547331	0.679884	1.452419	0.748738
214.5325	0.058337	0.018289	3.673053	5926.931	0.494834	0.648552	0.982648	0.727475
175.965	0.135122	0.030613	4.163985	28635.4	0.496122	0.643863	1.38257	0.714641
312.9155	0.343842	0.035159	3.84776	46579.58	0.320452	0.459131	4.325958	0.563474
121.6211	0.12583	0.037756	3.969658	9399.489	0.530053	0.679881	0.950387	0.746259
147.4238	0.09752	0.030536	4.095969	22451	0.493146	0.647596	0.979936	0.726084
167.7796	0.146626	0.031992	4.122297	7241.106	0.504013	0.647248	1.437195	0.722778
145.6921	0.075057	0.03198	3.949583	1386.066	0.607734	0.745441	0.666135	0.793896
236.2528	0.0484	0.016363	4.231276	18542.13	0.602783	0.732184	0.827264	0.790544
198.3134	0.157892	0.025705	4.030048	22788	0.47159	0.606568	1.979751	0.701737
262.0604	0.242813	0.032995	4.097962	24057.1	0.451466	0.596318	2.885455	0.666144
164.538	0.155419	0.03559	4.164751	7270.718	0.538134	0.67605	1.363006	0.738275
284.4655	0.101903	0.019467	4.277946	31792.97	0.537309	0.668791	1.701073	0.732512
143.2883	0.080044	0.028165	3.858692	2591.835	0.554115	0.702163	0.809235	0.763464
202.5634	0.089772	0.022302	4.414942	21015.92	0.566615	0.699145	1.153151	0.764354
192.6102	0.072338	0.022289	3.327357	961.1052	0.566751	0.709111	1.05336	0.761016
7860.678	0.004392	0.001636	3.431129	1806.473	0.542028	0.688663	1.198223	0.738797
186.1325	0.112652	0.029865	4.028157	6299.504	0.519585	0.664019	1.277771	0.72831
282.884	0.128798	0.022878	3.925391	13576.52	0.47317	0.618374	2.02196	0.686535
148.5555	0.135922	0.038494	3.657864	3896.125	0.604565	0.721657	1.268106	0.774316
205.8253	0.119472	0.028299	4.185697	26727.68	0.522065	0.664067	1.359108	0.727816
195.2324	0.142792	0.033464	4.032233	6691.04	0.443858	0.596587	1.643948	0.680289
198.1626	0.076649	0.025475	3.965928	5613.238	0.617487	0.739786	0.98314	0.791082
189.8292	0.050991	0.01931	3.808774	5086.408	0.566014	0.711754	0.751653	0.773333
304.0156	0.149308	0.025245	3.796914	6461.634	0.415001	0.560047	2.526027	0.651691
157.6918	0.172878	0.039124	3.387708	1979.68	0.481376	0.629224	1.681024	0.698476
104.1124	0.329611	0.072907	3.697148	3183.436	0.457367	0.613405	1.763771	0.680851
314.8834	0.069875	0.016856	4.103829	35837.23	0.517472	0.661198	1.365433	0.723955
273.1514	0.124091	0.022677	4.027356	72532.96	0.475839	0.617637	1.953677	0.691056
207.8337	0.231447	0.034518	4.011098	24282.72	0.412588	0.558878	2.548924	0.651132
488.2646	0.032941	0.009509	3.89496	6260.16	0.567777	0.697754	1.205713	0.761385
386.1379	0.083723	0.017631	3.977052	8304.984	0.500465	0.64063	1.870101	0.707197
198.3011	0.411263	0.040112	4.080696	27151.49	0.381133	0.503693	3.855477	0.628571
163.363	0.100908	0.029061	3.678397	7411.038	0.513522	0.663919	1.149012	0.72948
194.0062	0.111122	0.027695	3.961262	20197.03	0.506192	0.654465	1.326342	0.718764

153.3822	0.069715	0.022958	3.824898	8270.819	0.513489	0.670475	0.806426	0.741638
87.98686	0.32242	0.073128	3.912861	4840.76	0.534703	0.678641	1.407705	0.73323
619.952	0.072528	0.011718	4.650357	40437.55	0.475139	0.61497	2.452222	0.696572
591.3281	0.040229	0.009757	3.837224	3904.926	0.553134	0.679907	1.592656	0.742313
86.83773	0.093001	0.038365	3.470796	5098.792	0.602019	0.73998	0.618169	0.795143
400.3764	0.114193	0.017936	4.487343	47299.88	0.504223	0.640472	2.206636	0.704638
90.69923	0.269595	0.067168	3.73347	4975.298	0.591508	0.717012	1.236721	0.766987
114.4955	0.33299	0.065009	3.546888	9097.266	0.404991	0.552056	2.117987	0.647875
215.3987	0.082112	0.023797	4.363566	14779.85	0.4678	0.628023	1.096487	0.705504
367.3001	0.048148	0.015877	4.297575	10247.01	0.600094	0.723382	1.110128	0.779038
490.9448	0.038121	0.009859	4.326774	19376.63	0.57093	0.701315	1.25664	0.759801
136.4371	0.161466	0.041834	3.604372	4328.057	0.477529	0.630893	1.415736	0.703954
12096.95	0.008127	0.001873	4.083376	16828	0.457018	0.603392	2.249586	0.675814
371.2051	0.137743	0.025225	4.456987	30668.47	0.535568	0.664166	2.128725	0.722141
378.3224	0.098716	0.019287	4.107409	18385.47	0.433686	0.575202	2.196094	0.67232
224.4328	0.09196	0.026031	4.314032	10110.66	0.573643	0.706977	1.148389	0.764792
182.224	0.135387	0.029307	3.770382	14401.26	0.431069	0.584904	1.615312	0.673084
370.1785	0.303884	0.026375	4.525884	73760.84	0.408185	0.524559	4.710861	0.6432
128.1163	0.154981	0.048129	3.883472	2726.757	0.603289	0.731283	1.093482	0.780145
10796.18	0.047108	0.006086	4.050197	7209.596	0.539409	0.67519	1.771875	0.733259
133.1188	0.14715	0.037043	3.703009	3581.085	0.563648	0.699144	1.288206	0.752202
6970.983	0.023037	0.003686	4.187389	13956.26	0.611194	0.736046	0.914911	0.791815
356.9838	0.051892	0.016207	4.41804	8392.019	0.62565	0.739468	1.163134	0.796007
184.0563	0.049071	0.019259	3.770176	2754.997	0.584169	0.722107	0.725368	0.785097
331.9454	0.165866	0.023271	3.963742	36126.64	0.411187	0.561873	2.694553	0.638233
242.4169	0.343596	0.044401	4.391212	10151.95	0.550305	0.678218	1.927733	0.743191
117.656	0.239428	0.046644	4.167356	6012.785	0.557774	0.682519	1.480429	0.760462
313.9155	0.216693	0.027388	3.582665	6956.881	0.360625	0.494441	3.447937	0.609711
126.6804	0.226014	0.052832	3.514906	1463.613	0.531365	0.663832	1.772562	0.734425
154.5985	0.060534	0.024411	3.806737	3250.849	0.604679	0.740525	0.696741	0.794061
61.59597	0.135567	0.068983	3.128599	441.4758	0.652591	0.783834	0.558457	0.8233
510.0817	0.024117	0.007768	4.019746	3938.634	0.636104	0.750368	0.970924	0.800984
6167.674	0.005326	0.001033	3.974446	11604.03	0.469615	0.617386	2.066106	0.690659
265.6388	0.121579	0.024389	3.727961	13880.48	0.440164	0.583123	1.978426	0.675882
60.95818	0.2414	0.07268	3.233374	1886.945	0.4967	0.649141	1.058714	0.726027
75.46084	0.282056	0.077301	3.161376	672.3803	0.50893	0.66369	1.281815	0.719741
165.9003	0.121403	0.035473	4.285828	3601.902	0.637188	0.750389	1.029701	0.803783
218.0938	0.269719	0.040868	3.84886	6600.876	0.426212	0.576328	2.704003	0.654323
510.6073	0.158331	0.018608	3.917236	9486.585	0.428334	0.566009	3.836628	0.656577
288.5247	0.06912	0.0199	4.201759	16108.44	0.549154	0.686305	1.176968	0.747777
257.4459	0.085713	0.02208	3.802186	5245.065	0.524709	0.669108	1.405293	0.730817
59.48671	0.200827	0.071275	3.282788	1116.971	0.586058	0.719687	0.849124	0.780957
119.434	0.134989	0.041954	3.963671	7192.569	0.557182	0.695595	1.050193	0.762442
96.56515	0.201341	0.067863	3.706886	508.3703	0.6022	0.734324	1.075125	0.785918
444.2609	0.258288	0.032017	3.842367	2692.856	0.436212	0.566586	4.507197	0.658337
341.8582	0.067528	0.015959	4.064025	5388.953	0.541001	0.670653	1.494682	0.740115
203.684	0.046218	0.018556	3.702694	2984.801	0.566332	0.709723	0.751907	0.775929
292.2134	0.322154	0.033204	4.172826	14505.06	0.422868	0.553193	4.055539	0.656465
202.916	0.15276	0.041139	3.928784	3176.187	0.561517	0.686406	1.533862	0.751574
354.785	0.137294	0.022806	4.021042	18366.13	0.415379	0.55479	2.635102	0.651439
141.0527	0.104694	0.033672	3.791128	5316.179	0.581056	0.719034	1.002082	0.774354
155.6946	0.13227	0.039235	3.793714	3105.919	0.543986	0.672803	1.327585	0.749776
30.91738	0.772301	0.225052	2.81149	371.2735	0.736971	0.803633	1.21319	0.841752
191.5463	0.053615	0.018368	3.435686	7127.728	0.539108	0.688628	0.831366	0.75533
228.6178	0.048442	0.016567	3.682255	6720.34	0.555387	0.698577	0.878857	0.763966
42.64741	0.226817	0.093358	3.113294	804.9499	0.615378	0.744626	0.660554	0.797289
11873.95	0.004065	0.000624	4.232023	34760.26	0.444758	0.58745	2.782404	0.669879
186.9996	0.055753	0.021723	3.801331	3521.896	0.587652	0.724202	0.773231	0.78382

179.751	0.18834	0.038669	3.850378	12529.47	0.473518	0.612461	1.989468	0.69835
302.7729	0.167797	0.027217	3.943759	5331.759	0.473659	0.606312	2.722804	0.691001
168.4455	0.071308	0.026364	3.495106	2866.798	0.541334	0.683068	0.901621	0.756635
192.3912	0.150365	0.038854	3.82393	3534.24	0.545791	0.674867	1.672718	0.74268
300.6467	0.050263	0.01408	3.61676	11567.01	0.50408	0.650979	1.173097	0.728378
131.3416	0.090264	0.033316	3.684231	2265.053	0.585783	0.719809	0.860047	0.780076
284.0977	0.080108	0.020864	4.15926	11857.42	0.609415	0.726312	1.444009	0.778496
204.9517	0.06188	0.02138	3.81334	4624.305	0.556767	0.69649	0.953004	0.765251
311.2074	0.104514	0.019472	3.898365	120322.1	0.419912	0.555765	2.124959	0.666408
363.9723	0.086957	0.01883	3.707256	5464.137	0.477451	0.608365	2.082891	0.705353
177.6535	0.067893	0.023737	3.518491	2089.108	0.564203	0.708526	0.904312	0.765771
1063.256	0.177816	0.014465	4.223247	51923.47	0.347861	0.457048	6.839813	0.591475
172.2422	0.205033	0.036473	4.148872	16352.41	0.521031	0.65223	1.92049	0.728376
239.6212	0.099169	0.020359	4.26817	24672.8	0.485895	0.61767	1.643983	0.715913
68.98503	0.21696	0.066779	3.086816	516.0886	0.628116	0.752068	0.964721	0.79069
195.0569	0.079005	0.023013	3.677689	5061.174	0.555852	0.694581	1.091444	0.755456
304.1651	0.104705	0.021206	3.99568	21303.05	0.459062	0.597905	1.968323	0.688986
158.8675	0.089219	0.026521	3.600862	9800.672	0.515063	0.659573	1.096325	0.738379
222.3398	0.122543	0.034466	3.791366	2508.494	0.605472	0.722707	1.486858	0.774617
165.1433	0.22432	0.047897	3.675753	2456.657	0.490198	0.635827	2.043938	0.706309
269.8331	0.206211	0.026826	4.374279	16725.53	0.47585	0.603675	2.616532	0.692114
95.04378	0.194106	0.053423	3.023169	487.7746	0.641703	0.751997	1.299614	0.799327
239.9738	0.240643	0.033886	3.779286	20209.19	0.390391	0.535231	2.967307	0.631177
247.7863	0.103902	0.020889	4.320296	12289.76	0.492966	0.629978	1.681771	0.717404
94.5483	0.148764	0.082356	3.647623	318.0941	0.759379	0.83593	0.53867	0.875089
141.4029	0.157817	0.03986	3.841595	7148.26	0.489534	0.639495	1.405547	0.713383
347.1405	0.132586	0.02065	4.060293	24739.42	0.464202	0.608908	2.386683	0.679147
98.80188	0.274911	0.058686	3.83399	8254.852	0.518026	0.660477	1.470161	0.727026
198.2493	0.063171	0.021683	3.708063	7898.362	0.547568	0.690008	0.941841	0.757553
96.60925	0.119939	0.044318	3.675464	809.8776	0.653253	0.767589	0.747048	0.811028
214.2852	0.047303	0.018691	3.977529	5343.284	0.564046	0.708955	0.767733	0.774109
371.0797	0.096627	0.022071	3.982697	5168.693	0.507549	0.637022	2.165588	0.721289
161.9367	0.049964	0.020484	3.964519	4031.623	0.617747	0.751394	0.620614	0.805343
255.0603	0.13079	0.032015	4.341303	4419.952	0.528562	0.640362	2.055164	0.74631
126.6506	0.08379	0.042779	3.259832	174.7408	0.806677	0.848135	0.739141	0.884009
331.3564	0.414618	0.033461	3.380422	2552.658	0.352163	0.510617	5.23783	0.58206
100.8375	0.159615	0.053324	3.267539	669.9558	0.614595	0.744586	1.007588	0.783287
420.3242	0.23028	0.024553	3.629705	7149.188	0.321582	0.475868	3.881488	0.55726
343.441	0.140156	0.022484	4.175782	37479.24	0.431217	0.577889	2.4202	0.656612
15531.27	0.005903	0.00127	3.422088	2573.165	0.334408	0.480253	3.5051	0.577782
334.0431	0.311393	0.03105	4.447763	10014.96	0.390236	0.520532	4.219872	0.624883
338.2054	0.256107	0.028862	3.357333	1449.842	0.359453	0.479537	4.137565	0.612244
375.8732	0.162857	0.023705	3.451353	1346.125	0.402477	0.523785	3.432925	0.652051
212.4717	0.437657	0.054938	3.552964	2017.518	0.361253	0.515071	3.862306	0.59525
507.0888	0.131269	0.01994	3.982878	6119.71	0.409932	0.527333	3.560392	0.653423
290.8221	0.114155	0.021131	3.692687	37667.95	0.399821	0.545976	2.089733	0.647064
357.9297	0.4673	0.038965	3.886506	14292.12	0.367747	0.50375	5.431805	0.587748
200.3102	0.093951	0.024511	3.468101	1727.855	0.489158	0.641438	1.350614	0.714775
69.84386	0.757602	0.114205	3.407265	2818.758	0.406592	0.546135	2.812127	0.647668
167.7613	0.125416	0.03241	3.629379	1287.251	0.514126	0.655257	1.436831	0.72856
8966.874	0.009942	0.001136	3.79138	20295.84	0.359009	0.48174	4.27014	0.608494
257.045	0.086263	0.021056	3.450208	891.7134	0.515187	0.662831	1.575632	0.727728
278.0415	0.08989	0.020027	3.610933	17406.38	0.430387	0.581161	1.655129	0.6708
107.8659	1.233082	0.113637	3.033247	382.368	0.449288	0.599987	4.899378	0.653926
118.0254	0.225752	0.051194	3.299126	2022.458	0.498262	0.649891	1.702548	0.714508
259.7707	0.202077	0.044729	3.819715	3223.904	0.457849	0.585797	2.673798	0.683633

18417.98	0.026781	0.001333	4.56744	6359.024	0.311189	0.350655	13.11968	0.574653
336.4706	0.078066	0.016624	4.264961	58464.38	0.46169	0.609134	1.691653	0.693444
444.3302	0.306593	0.026571	3.849708	26112.43	0.333919	0.438199	5.624251	0.58672
450.299	0.145455	0.0189	3.930092	17711.92	0.372939	0.511713	3.291261	0.620462
417.3787	0.100195	0.01708	3.800632	9136.765	0.418517	0.554301	2.519443	0.660013
223.6252	0.236848	0.039124	3.51553	3310.932	0.399755	0.547866	2.757571	0.635374
302.5432	0.241932	0.031458	3.749815	3101.274	0.403839	0.546054	3.496106	0.637212
281.4917	0.324868	0.035161	3.833631	7132.725	0.353748	0.507499	3.966508	0.592281
92.02196	0.499366	0.090672	3.611803	1883.752	0.45391	0.603065	2.177817	0.671373
212.5257	0.143878	0.0394	3.697531	2064.579	0.469383	0.60305	1.890529	0.7021
252.2384	0.217561	0.035722	3.732717	3805.567	0.43667	0.591887	2.693698	0.655109
141.5032	0.423225	0.067869	3.630952	6087.031	0.433895	0.582442	2.787134	0.655209
114.5339	0.563884	0.104103	3.391508	1028.084	0.483069	0.629961	2.841763	0.688144
54.74744	0.811324	0.163791	3.301914	1299.709	0.429026	0.578906	2.246919	0.662198
275.8829	0.482117	0.048329	3.705892	6524.079	0.356148	0.482696	5.088517	0.593298
115.5776	0.345185	0.065167	3.264605	675.0796	0.45524	0.609566	2.128145	0.674158
177.5763	1.859711	0.09676	3.676237	1410.158	0.296085	0.409397	9.096793	0.541442
426.859	0.140136	0.019835	4.467481	29467.75	0.422101	0.56262	2.801076	0.658209
363.7034	0.08768	0.017982	3.880835	16573.94	0.445602	0.586396	1.980875	0.676614
172.0559	0.146781	0.03387	3.738392	8834.278	0.470599	0.61543	1.633102	0.699528
279.7748	0.156071	0.025753	3.587057	6051.86	0.390742	0.534315	2.523612	0.637368
238.2535	0.134005	0.024053	4.264227	36473.46	0.442818	0.585497	1.918277	0.678259
308.5181	0.181654	0.026064	3.481721	8777.629	0.367585	0.518278	2.801272	0.606814
38.17508	0.683805	0.155412	3.069939	38.69923	0.492747	0.638158	1.212268	0.692008
285.3746	0.124561	0.023736	3.285019	2721.785	0.458587	0.576387	2.408511	0.696723
393.7774	0.12606	0.021456	3.904485	16915.25	0.415379	0.559143	2.666585	0.651029
476.6346	0.085173	0.015006	4.049467	21220.24	0.412471	0.563472	2.255014	0.648304
642.855	0.054328	0.012781	3.977649	7887.968	0.4495	0.581846	2.138644	0.685972
170.9617	0.106856	0.033036	3.567493	532.9924	0.513553	0.660951	1.277665	0.732835
115.0301	0.154495	0.043061	3.311979	426.7525	0.523009	0.683647	1.309754	0.735351
769.9845	0.105765	0.015137	3.960361	5235.707	0.382758	0.526054	3.66872	0.618216
414.793	0.061309	0.01526	3.801169	1795.244	0.50296	0.643419	1.665313	0.719767

AX85	AX86	AX87	AX88	AX89	AX90	AX91	AX92	AX93
13.3093	0.048055	69.9443	0.185529	3.67666	20.77719	373386	6475982	22783.65
16.59812	0.022442	53.33003	0.176007	3.327778	29.83498	2110251	48376246	98996.93
19.87635	0.019071	90.03636	0.272837	1.987842	31.80606	1297745	45330386	37813.88
18.57341	0.03742	11.73469	0.239484	2.13661	16.85714	9725.796	288778.1	340.4355
23.45021	0.03143	17.23214	0.153858	3.630022	28.00893	48804.12	1727961	1593.628
15.14718	0.034649	52.83333	0.209656	3.779415	22.83333	564060.9	11191755	29938.82
54.00423	0.013087	40.10105	0.139725	6.112688	58.94077	51552.63	3542361	767.2346
28.88433	0.020439	66.51958	0.17368	4.280089	34.1436	164625.8	6795726	4148.363
19.27622	0.063969	10.31148	0.169041	4.318732	17.80328	4769.18	159424.4	153.1172
84.1718	0.009627	148.7007	0.099332	9.175833	87.12692	110049.7	11615586	1102.553
27.33339	0.013889	731.2245	0.258932	2.413941	43.14943	5728747	2.74E+08	121089.8
24.38918	0.01549	753.3553	0.180617	4.043637	46.46679	16635003	6.52E+08	438536
21.9926	0.027793	73.29724	0.15562	4.691207	27.85775	469903.9	14454569	16725.14
1725.577	0.009397	21.43333	0.119074	68.2	2033.644	10965.38	23580819	13.25414
23.64105	0.028836	9.93617	0.211408	3.148031	26.68085	14682.94	498497.1	444.1843
32.39416	0.013708	243.6516	0.177589	3.975161	46.84329	2454162	1.24E+08	50556.6
1446.842	0.001461	8.361111	0.116127	54.81771	1871.708	34276.63	66948996	18.13492
33.63407	0.017456	91.78797	0.138027	6.364534	46.29173	642509.3	22477142	19520.71
74.09436	0.011681	127.1725	0.083721	11.49244	100.9375	78651.77	6914185	986.6255
44.47831	0.013493	246.7071	0.126322	6.240809	54.97593	1003927	65312969	18585.41
27.43811	0.015524	225.7841	0.147283	3.995745	47.57469	4170206	1.67E+08	110805.8
48.41153	0.015057	128.0053	0.112089	6.848538	58.14273	181212.5	10039582	3898.421
10.70354	0.049015	16.73134	0.249722	2.442415	12.83582	103117.3	1827925	6043.011
44.97643	0.010194	137.8288	0.130396	5.7636	77.82876	3572000	2.18E+08	61632.75
27.82612	0.018702	100.3235	0.147535	4.811711	37.78971	692008.5	30186114	16441.13
1521.908	0.00077	14.33333	0.154122	25.396	2016.667	32959.46	68593322	15.87142
10.87249	0.033284	43.17333	0.287822	1.787956	18.38	1172379	22291589	64498.08
36.02783	0.023767	58.74131	0.1134	5.994443	47.89382	190845.6	7134660	6231.646
14.02865	0.042638	27.97059	0.205666	2.944637	21.13235	152247.6	2915816	8722.644
28.62502	0.025628	60.40247	0.149142	4.466917	31.79012	123359.2	4466325	3881.117
37.80007	0.023818	22	0.161765	5.286981	37.67647	19912.77	1148077	354.3168
21.21386	0.029446	125.7829	0.131297	4.97501	32.92797	819409.3	21446628	35324.75
45.75402	0.015472	18.37063	0.128466	5.968018	51.41259	43899.65	2838152	701.7449
37.71725	0.020231	90.28404	0.105967	7.722292	57.33216	396929	13162555	13607.89
30.4099	0.014867	568.3803	0.142953	4.575658	52.53546	12762945	5.07E+08	337907.7
30.96739	0.020756	16.11579	0.16964	4.273019	38.52632	75724.71	3922470	1537.91
34.45445	0.016726	195.5795	0.117819	5.81795	59.09036	2424909	1.01E+08	71660.38
17.62246	0.04415	13.4086	0.144179	5.151809	28.15054	43948.96	820419.2	2802.07
53.32923	0.012961	46.89865	0.158441	4.580704	47.93581	53952.11	4393771	676.1658
46.2449	0.016008	7.836364	0.142479	5.304463	47.43636	17838.6	1288591	254.0517
61.447	0.011182	95.97051	0.128647	6.196402	70.60456	127087.9	10565158	1584.515
72.24146	0.009833	488.2925	0.082218	12.63321	112.3304	1174033	94416649	16063.62
33.65406	0.039218	34.47849	0.092684	11.07408	51.76613	47708.28	1560835	2212.091
51.78429	0.011304	128.7577	0.104005	7.583972	78.60339	1328215	90351331	20997.26
48.53849	0.010316	101.0608	0.128087	9.614966	85.2763	918294.7	63962327	13697.03
35.13808	0.024143	29.51	0.14755	4.960775	30.615	31683.91	1747899	599.6958
15.0783	0.035816	42.35912	0.234028	2.361344	17.61878	151693.7	3902011	6077.017
26.58665	0.042125	75.9441	0.117926	6.470745	34.55745	287362.6	10950510	10772.62
26.59814	0.027854	55.61425	0.136644	5.317919	36.20639	278250.4	9354947	9520.845
62.62676	0.009879	59.91993	0.111583	6.873159	77.78399	296230.4	25580140	3617.898
42.04813	0.010691	182.0878	0.164785	4.170296	58.86335	1257815	78482497	20588.74
21.64705	0.035024	20.9646	0.185527	2.953246	20.54867	29828.23	1023834	942.8799
33.65895	0.024279	24.70854	0.124164	6.325497	40.59799	57728.75	2224097	1727.159
74.66874	0.009191	65.09747	0.126896	6.549981	80.95322	93302.26	10040008	891.5814
33.53823	0.014906	196.3055	0.141125	8.592583	56.26312	1636101	86750892	32543.72
56.38906	0.010175	81.20355	0.131185	6.290322	65.59774	508134.7	47172433	5565.858
14.04052	0.034199	51.2723	0.240715	2.959113	21.61502	530818.5	10631476	28419.43

22.89547	0.024871	25.04959	0.207021	2.823851	30.97521	74623.22	2651760	2131.674
13.73933	0.063773	15.27273	0.173554	3.976111	15.98864	53382.75	939372	3590.746
40.45595	0.01503	61.25485	0.169681	4.296514	43.45429	109985.2	6878286	1818.872
35.34934	0.011623	593.0685	0.130287	6.02411	69.07162	13358304	6.94E+08	268084.8
26.29043	0.039068	19.20168	0.161359	3.80298	32.27731	27518.13	942626.1	1120.122
38.02203	0.014857	95.74372	0.160375	4.74561	51.63484	432050.6	22302775	8605.628
54.95269	0.011433	178.4783	0.109295	7.447241	74.09002	711545.1	46827123	11628.72
67.59502	0.010795	198.9296	0.088689	11.06078	94.59206	367418.5	27719648	5551.314
33.35161	0.026103	27.58427	0.154968	4.641112	31.64607	25759.78	1293719	548.1009
45.46812	0.017938	16.568	0.132544	4.777344	43.416	9288.824	610023.5	148.1849
48.37313	0.0234	10.86777	0.089816	10.31009	49.1405	17371.22	1127144	304.7458
39.71858	0.013384	219.2489	0.165346	4.256734	54.73529	757786.3	40938591	14884.25
34.97659	0.01224	719.8356	0.173747	3.878744	60.05093	7968647	4.04E+08	160664.2
33.05813	0.032614	254.577	0.086975	15.44108	64.80526	949752	18281349	66066.75
53.05592	0.010326	423.0221	0.116599	8.209268	83.81009	2529605	1.56E+08	42987.82
1111.278	0.000485	297.1252	0.232492	7.485489	1910.916	4651844	8.87E+09	2439.757
11.87813	0.062926	6.586207	0.227111	2.437574	12.06897	21150.03	378238	1267.827
23.87675	0.024653	57.72555	0.1821	3.975201	30.67508	216107.9	7248526	6942.618
45.28471	0.008864	288.5861	0.173117	3.403369	72.4817	2272899	1.69E+08	31020.94
15.97416	0.043947	54.73913	0.169997	3.674521	20.5559	153593.4	3311996	7954.121
41.84626	0.016289	197.81	0.107389	7.968718	64.34093	492232.2	22533122	11581.63
28.50476	0.021581	49.09231	0.188817	3.884083	37.79231	238428.9	10847111	5810.92
59.58052	0.010404	251.8142	0.122478	6.979903	81.20282	511973.7	37922792	7396.329
69.20614	0.01304	121.0217	0.109522	7.160759	75.99819	207709.2	18935901	2652.443
45.28126	0.013696	98.49568	0.170113	5.933409	57.97755	153466.9	9759755	2474.854
31.58101	0.021756	153.5772	0.107849	17.11974	65.66854	824431.4	32998600	23192.92
17.84007	0.020169	1013.526	0.322676	3.071857	32.90481	11984360	3.65E+08	406296.4
27.42209	0.028384	85.28923	0.131214	5.218578	34.22923	149165	5407576	4975.22
31.95672	0.022871	201.1836	0.116899	7.201516	41.55433	508037.9	22204856	12673.46
30.81527	0.023084	72.87575	0.146044	4.814575	32.99599	132450.9	4956650	4204.203
39.44971	0.026942	11.31481	0.104767	7.239626	38.62037	10160.4	550515.4	221.1798
63.73221	0.013141	132.0124	0.102494	8.540232	68.13432	122788.4	9351698	1764.691
34.90705	0.017141	147.5192	0.128501	6.138899	53.68641	1100523	37807218	34621.63
26.60695	0.022199	110.4639	0.105004	18.10929	77.24715	1363219	47955029	40697.2
30.66112	0.026161	42.68508	0.117915	5.8965	41.46685	130303.6	4637751	4599.416
49.05914	0.014052	113.5968	0.10528	9.495737	76.05653	916832.8	47730066	18778.55
34.26335	0.022149	23.66272	0.140016	6.620987	42.83432	40975.4	1894388	976.5593
51.70118	0.016632	123.192	0.080465	12.25914	102.5153	191699.8	9261950	4465.194
40.04634	0.017534	15.43038	0.195321	4.477808	46.81013	16676.94	908617.6	312.9958
1434.46	0.001359	20.58	0.2058	38.5075	1883.21	48046.12	94243953	24.88125
31.94526	0.022563	55.87711	0.134644	5.427783	36.27711	109473.5	4915300	2736.93
34.60922	0.015941	85.9375	0.149197	4.83579	49.81424	692321.2	33704612	14865.49
27.64602	0.031015	25.4385	0.136035	4.930024	30.14439	57197.96	2092816	1667.007
35.21517	0.020746	164.0128	0.131001	5.514198	45.61182	585031.4	28827511	13212.5
26.25164	0.024099	69.6096	0.145323	4.615478	34.01044	262797.5	10882172	6851.917
43.2408	0.020797	44.0791	0.124517	6.004253	41.22881	44486.15	2759596	805.8493
52.4101	0.015443	65.76175	0.106583	9.891586	75.16856	48805.29	3097731	792.9567
29.56847	0.017338	88.80507	0.173109	4.239124	40.54386	428915.1	19974570	9380.94
21.26586	0.028721	29.73529	0.218642	3.341479	26.75	92026	2940830	2973.74
13.26918	0.052773	33.09143	0.189094	3.501584	18.02857	172540.4	3424830	9546.332
51.8017	0.0124	222.6645	0.131056	5.738712	66.84344	865632.9	66787521	11868.54
35.94529	0.015737	430.7037	0.132402	5.44938	55.93667	3175108	1.53E+08	69247.24
23.04284	0.022457	257.3511	0.177851	4.423285	35.73531	1717557	53373221	58714.07
90.23975	0.007489	41.19512	0.11164	7.517321	97.17615	109053.5	14243727	849.2991
49.25907	0.013083	65.03279	0.133264	5.868848	58.46107	228250.8	16848105	3183.095
19.82523	0.024084	280.9388	0.172038	4.217796	33.58849	2982388	68870221	144681.8
30.65714	0.022033	88.08922	0.163735	4.780448	40.60223	191587.6	8218333	4622.598
32.6142	0.020411	138.4214	0.13439	5.483579	44.20097	609076	28518779	13941.6

38.27775	0.01717	93.0189	0.146486	5.959561	51.19843	174108.3	8568198	3749.104
16.76561	0.053394	32.19249	0.151138	4.42853	23.8216	123532.7	2251060	8199.465
72.75846	0.008301	227.1943	0.090841	14.37978	101.583	690073.9	71778387	7069.625
85.79841	0.007551	27.52326	0.160019	5.662351	89.08721	99366.25	12908414	777.9907
26.30538	0.031184	62.25624	0.141171	4.567305	34.68027	55192.7	1792773	1828.811
51.05331	0.012508	150.7387	0.098458	8.057815	79.6597	1401642	89021071	24790.41
19.14085	0.050924	28.9801	0.14418	4.688498	28.801	90288.78	1856986	5595.744
12.62618	0.045193	249.5043	0.308411	2.76364	19.27194	727478.1	14351294	38801.64
40.01869	0.017259	105.5896	0.111972	7.456856	50.90774	414346.8	24008750	7676.564
70.11228	0.013046	62.44816	0.104428	8.731412	67.90301	89800.03	10066855	861.5062
93.32438	0.007575	89.83676	0.097121	9.505267	120.4032	246294	31561296	2028.126
20.88255	0.031278	80.89367	0.204794	3.216728	23.63797	128226.6	3989102	4291.088
1337.649	0.001506	113.8298	0.134551	16.99603	1964.872	768495.2	1.54E+09	387.6919
43.01705	0.019274	95.66737	0.100702	8.092392	52.82737	760012.7	52239396	12876.55
41.48076	0.013574	208.0444	0.144475	4.833316	56.13472	813454.7	55539855	12326.3
44.99362	0.020299	61.67972	0.10975	7.419311	48.74911	91072.55	5900517	1823.303
26.31993	0.020064	245.1281	0.198806	3.733781	38.28873	706294.1	26241893	19842.34
31.76306	0.016466	415.6536	0.133565	6.151819	51.07455	5870034	2.18E+08	177415.9
26.894	0.038845	16.21168	0.118333	6.140657	30.41606	34653.15	1274798	1099.997
1559.854	0.005349	42.2953	0.141931	11.79002	2066.191	165255.8	3.6E+08	111.6493
26.27667	0.028018	26.25532	0.139656	5.448025	38.63298	71456.11	2164129	2518.981
1721.571	0.003301	115.3845	0.10664	9.871431	2033.957	86460.59	1.92E+08	48.92382
71.83762	0.013311	46.18341	0.100837	9.794855	76.40611	53772.05	5723014	599.3338
51.71505	0.015682	43.0112	0.12048	6.46552	56.13445	20276.34	1291150	331.0807
30.80372	0.01495	282.8177	0.177761	3.832478	48.60717	3258427	1.49E+08	74331.16
36.44245	0.030413	49.05277	0.129427	5.933083	47.96042	166644.8	9046006	6642.71
28.60606	0.033403	54.37379	0.131975	4.564727	41.4733	69725.66	1366309	4280.204
23.98455	0.01725	241.1362	0.349473	2.18698	31.92899	918676.3	35366557	24221.7
18.95386	0.041297	18.80531	0.166419	3.766466	24.69912	28741.44	778008.5	1129.701
43.24912	0.02021	28.21705	0.109368	8.927649	53.37209	28988.33	1601331	554.8906
19.3421	0.060093	14.73134	0.219871	2.360882	16.50746	2062.642	56754.27	79.49625
120.5851	0.006384	31.28112	0.125627	7.290076	126.4418	26417.08	4363571	164.59
757.0152	0.000743	77.55611	0.12991	22.00201	1056.104	465782.8	5.12E+08	424.4444
32.307	0.017324	208.0725	0.191068	3.552815	46.70891	764834.2	37911615	15775.59
12.53187	0.054551	41.14379	0.268914	3.237558	18.71895	73301.8	1244409	4598.752
11.6643	0.059416	10.37778	0.230617	2.362469	13.62222	24743.51	449704.5	1439.229
42.05284	0.028684	23.96651	0.114672	7.054784	53.60766	12613.88	551554.8	427.2235
20.95848	0.028324	55.99674	0.1824	3.489056	35.2443	565783.8	17909543	18728.83
39.11005	0.012424	73.41986	0.165733	4.192021	58.24605	813509.7	47138049	14390.04
54.40385	0.015688	100.222	0.122222	6.835412	62.04756	271398.1	21432806	3632.45
40.07015	0.016887	36.34127	0.144211	5.192539	48.74206	158036.3	9768611	2659.642
15.69761	0.057725	28.38462	0.242604	3.330703	21.66667	14709.85	292593.1	790.0859
27.56432	0.033358	54.99097	0.124133	6.743892	35.60722	120279.9	4019694	4050.102
22.50934	0.055859	6.583333	0.137153	5.354167	27.91667	4154.313	106830.6	184.8889
27.25704	0.022928	24.05983	0.20564	3.162393	39.16239	242926.9	10981453	5536.12
55.08403	0.012137	36.21384	0.11388	6.364711	63.65723	88638.7	6951535	1217.528
52.6395	0.015183	47.98947	0.126288	6.380741	55.78684	29465.01	2195475	405.0508
24.5675	0.021721	111.2748	0.146994	4.080593	34.52708	837342.3	29904836	27061.51
30.52139	0.033065	29.8972	0.139707	4.148768	32.97664	43816.91	2009578	1027.445
33.35778	0.015575	194.2578	0.169214	4.607671	47.31185	1358159	74324163	25470.73
31.19527	0.026923	48.27841	0.137155	5.450639	36.39205	66300.97	2780200	1776.675
27.43607	0.031592	34.92593	0.143728	5.761622	34.86831	56710.21	2277963	1488.23
7.564632	0.190945	5.461538	0.210059	1.72929	10.5	2045.192	15603.88	390.9923
46.40865	0.014203	140.0351	0.20473	5.104722	62.92251	139444.9	8624156	2306.02
53.13471	0.013065	92.09291	0.145028	5.336063	63.09606	89279.55	6581342	1243.177
11.83991	0.077039	15.34375	0.239746	2.308594	14.125	10866.47	165228.5	808.3591
1181.368	0.000433	161.6697	0.123318	11.02742	1735.336	1566704	2.7E+09	909.2474
47.96009	0.017857	45.78261	0.117091	6.283986	52.49616	25156.74	1679953	392.4234

23.06997	0.028694	153.2038	0.183698	4.395298	29.92566	500882.5	16519122	15885.32
30.5537	0.019684	42.93506	0.1394	4.692739	40.14935	232837.1	10755398	5206.542
36.21474	0.021318	72.51032	0.213895	4.401302	41.86726	46833.11	2584484	869.1506
26.92144	0.030611	29.53589	0.14132	4.453424	28.22488	69395.79	2940823	1788.191
55.99912	0.010526	189.214	0.17918	4.357004	72.38826	283551.3	22752172	3607.829
30.87972	0.027264	30.21827	0.153392	4.947615	33.95431	23609.3	1026939	583.9125
52.67423	0.016781	58.49342	0.096206	9.272612	66.43914	107028.5	7385767	1624.864
45.227	0.017268	73.58527	0.142607	4.982318	47.03101	50315.99	3309914	791.1842
40.80893	0.013197	1930.175	0.14415	25.7372	107.2286	6157764	3.41E+08	113574.6
43.58391	0.014192	88.56364	0.161025	4.454096	49.56364	175527.7	12100964	2577.947
40.0999	0.019008	27.36364	0.155475	5.675491	46.89773	33670.22	1917504	605.6204
44.20688	0.009018	475.251	0.152961	4.640825	67.00354	10229084	8.09E+08	130490.4
29.25451	0.026249	101.7266	0.126999	5.719782	39.82772	345826.6	11154313	12672.49
47.14213	0.01437	219.5626	0.108159	10.10773	69.35222	645555.6	30086747	14456.97
15.81811	0.053469	5.6875	0.177734	3	21.0625	6932.969	140997.7	380.1886
39.61974	0.017942	45.66463	0.139221	5.048	49.62805	86919.92	4938197	1580.459
38.54608	0.015342	192.2525	0.137127	5.153899	51.27175	911440.4	52742130	16255.65
32.40283	0.020182	164.4761	0.174418	4.238462	41.2895	209671.5	9236135	4934.38
36.55659	0.028379	13.94309	0.113358	6.897085	42.02439	40955.6	2150388	835.5028
20.27274	0.036232	32.96386	0.198577	3.791261	24.31325	85296.86	2561569	2981.289
34.94163	0.017971	74.95238	0.118972	6.061691	51.67778	635502.2	26529351	17906.17
19.30154	0.043562	5.275862	0.181926	2.960761	27.27586	7572.931	188049	327.9243
21.01365	0.021807	264.0906	0.208109	2.631234	33.97006	2122208	69248432	67449.87
44.84785	0.014824	74.82461	0.105834	7.140388	61.75813	304215.1	15085929	6729.662
31.42696	0.075303	7.1	0.118333	5.6475	30.15	325.8667	16905.1	12.59935
24.61199	0.029662	80.98859	0.153971	4.6703	32.48289	203711.4	6572196	6654.808
39.29567	0.014184	117.1657	0.131205	5.330412	54.78611	1477165	78238766	29598.19
18.35164	0.041889	59.30208	0.154433	4.588535	24.86198	238539	4801751	14608.07
43.03744	0.01723	102.2108	0.145599	5.339163	49.53704	123172.9	7597313	2053.233
28.19801	0.036728	8.016393	0.131416	6.606289	31.44262	5358.311	169014.9	197.4221
54.69492	0.015291	82.50209	0.114746	7.218343	54.84562	38178.22	3022917	507.9916
41.11008	0.017266	49.31429	0.140898	6.492767	44.01714	136324.3	9731317	1970.389
53.07215	0.016874	37.97396	0.098891	9.401198	59.09635	26385.5	1592062	469.5953
38.95569	0.026208	66.43967	0.135868	5.173456	31.56237	44325.32	2425475	927.6843
40.75313	0.039234	3.230769	0.12426	5.390533	34.38462	300.8077	14789.46	6.934539
19.64355	0.018914	21.41176	0.209919	3.67474	37.58824	731655.8	20894278	26377.9
20.92653	0.043633	11.06667	0.184444	3.0275	21.95	7246.117	217175.8	257.9738
24.10528	0.013933	129.4701	0.276645	3.50794	42.46368	1807809	78408206	42665.64
34.11048	0.015188	211.6701	0.135339	5.502703	51.01343	2577287	1.41E+08	49696.87
1035.272	0.000963	81.59487	0.418435	26.06112	1705.379	564188.5	1.01E+09	314.5301
26.49046	0.019503	79.73984	0.162073	4.448642	37.5813	910688	29604961	30484.54
23.67795	0.018242	75.93103	0.327289	1.944411	34.12069	153587.8	5581871	4237.157
30.27065	0.016337	50	0.227273	2.651632	42.51364	79902.68	3897843	1644.468
13.02107	0.03523	27.28099	0.225463	2.983403	27.90083	368940.8	8605111	16326.75
39.35509	0.014169	90.95808	0.136165	5.682348	52.52695	389373.2	24878386	6142.616
33.3127	0.014036	672.7186	0.211281	3.72206	51.32946	3192517	1.64E+08	63245.83
17.65732	0.023646	138.6496	0.229173	3.241653	28.79008	2836220	80798059	102595.5
34.47866	0.01818	31.31868	0.172081	4.150344	49.82418	47962.07	2311662	1015.633
7.761569	0.074877	61.32407	0.283908	2.256173	14.12037	285275.8	2857797	30139.69
27.02171	0.024649	17.03478	0.148129	3.980794	35.24348	26588.49	1045020	709.169
586.2158	0.000723	319.8838	0.207582	17.91945	936.2336	3196835	3.06E+09	3345.472
41.55602	0.015959	12.19101	0.136978	5.562934	52.53933	19297.44	1103176	344.8125
36.99277	0.014008	453.4774	0.259575	4.183052	51.88552	933002.7	53297153	16625.93
7.543511	0.076018	8.428571	0.30102	1.96301	14.46429	25585.29	243043.9	2767.819
17.67172	0.038307	37.68263	0.225645	2.657535	24.64072	63123.98	1560414	2599.953
23.58085	0.035023	27.54822	0.139839	5.272488	26.56345	192089.6	7921949	4786.219

456.8523	0.000812	216.6203	0.239095	13.56661	786.9713	1747175	1.46E+09	2090.225
51.18311	0.011752	412.3719	0.09908	9.714657	81.12975	1661431	1.13E+08	25458.75
24.08032	0.015742	742.2635	0.283848	1.83652	41.47113	4848969	1.85E+08	129160.2
48.63867	0.011861	257.8811	0.126661	122.8187	268.9602	1480919	81580051	27351.42
42.00197	0.011692	135.3976	0.16313	4.568386	60.13373	560019	36886431	8636.258
18.68591	0.026723	60.91837	0.248646	3.493978	27.40408	329498.7	10564798	10599.36
23.65832	0.020893	38.57576	0.166995	4.262814	35.25541	236266.9	8412070	6779.747
19.40506	0.021017	92.92793	0.209297	3.48813	36.47297	1120543	33974014	38273.11
10.59753	0.063683	22.1068	0.214629	3.743237	24.38835	117217.4	1766881	8474.565
23.19139	0.031299	44.67078	0.18383	3.698149	22.54733	60440.34	2652017	1415.005
21.6069	0.024898	36.3301	0.17636	3.341526	30.1699	238471.8	8540920	6934.162
12.44011	0.047088	74.59873	0.237576	3.346262	18.84076	499234.5	9843920	26908.96
10.01685	0.079343	10.13725	0.19877	3.230296	13.19608	54180.33	828631.8	3736.89
5.636795	0.118152	31.23853	0.286592	1.745308	8.486239	83191.77	734509.5	10337.49
14.05177	0.030697	95.26598	0.243647	2.334469	21.10486	1335664	32838818	55273.37
12.62344	0.046354	9.697674	0.225527	2.601406	17.32558	44595.7	854539.2	2439.609
7.315007	0.052161	24.14607	0.271304	2.207802	13.49438	712823.1	7040382	74876.69
40.67194	0.013287	249.9086	0.122085	5.921877	52.71764	989702.4	65993731	16871.34
43.53236	0.012861	213.214	0.161771	5.420304	56.32929	803299.6	54422956	12046.69
24.76802	0.024753	121.6696	0.176333	4.120187	35.36667	332469.9	12196741	9449.537
26.86726	0.01699	150.4153	0.24028	3.578162	40.27157	496577.7	21396657	11800.39
36.37167	0.015958	270.361	0.125925	5.508406	56.27154	1682108	67464149	45171.1
24.67349	0.016367	119.1502	0.255687	3.268088	41.22532	1751732	73819954	42575.67
6.578127	0.110059	5	0.294118	1.00346	11.47059	155.7647	1124.824	26.05954
32.98287	0.017314	81.66258	0.250499	2.936881	44.98773	131387	6406838	2706.494
35.21155	0.014934	169.5211	0.162377	4.640694	46.75383	1326447	77465523	23227.46
49.04621	0.010109	192.9957	0.13965	5.53735	69.27135	1378561	1.11E+08	17604.81
71.62407	0.009766	116.7215	0.123124	6.989273	80.5	258493.1	30330761	2221.786
28.78428	0.026039	10.69444	0.148534	5.248264	38.79167	9056.417	394753.6	214.2177
23.15962	0.033098	8.041667	0.167535	4.714844	32.9375	7729.333	218279.6	282.9896
48.97537	0.01043	58.30198	0.144312	6.582265	61.39356	487817.8	43436524	5541.147
58.21499	0.011785	26.14286	0.128783	5.667985	62.32512	31763.07	2789920	366.982

AX94	AX95	AX96	AX97	AX98	AX99	AX100	AX101	AX102
0.14096	66.7931	0.17717	0.413963	7.992828	0.073071	5.670704	0.017205	370007.9
0.059658	38.76898	0.12795	0.342278	9.045425	0.024638	6.001242	0.006602	2087309
0.044597	75.30909	0.228209	0.481812	14.94001	0.024459	4.803961	0.01057	1288796
0.125288	9.326531	0.190337	0.418658	4.163569	0.088694	4.374016	0.039902	9097.731
0.073425	24.96429	0.222895	0.475187	12.43845	0.032839	5.14714	0.020722	46475.19
0.113336	42.02381	0.166761	0.400422	8.964539	0.04831	5.583656	0.012606	557767.7
0.033846	62.47038	0.217667	0.464409	25.90138	0.020389	5.650834	0.035511	50759.63
0.061286	97.67885	0.255036	0.514028	16.22997	0.036894	5.283524	0.024611	162974.8
0.243698	11.2623	0.184628	0.437315	6.307741	0.158547	4.933807	0.050918	4383.476
0.019924	346.5458	0.231493	0.488859	40.08775	0.011004	6.379234	0.037804	109349.9
0.031867	650.4093	0.230315	0.482844	20.59363	0.015889	5.164508	0.014724	5724134
0.03219	711.6483	0.170618	0.407445	18.33444	0.013637	6.205206	0.00894	16622491
0.093592	103.2208	0.219152	0.475691	11.96742	0.055269	5.703515	0.014551	465181.2
0.017131	32.82222	0.182346	0.422105	862.5598	0.000846	5.734448	0.035294	10162.6
0.081849	6.234043	0.132639	0.348149	7.762252	0.045587	5.096934	0.03206	13710.03
0.033021	288.0087	0.209919	0.458799	20.87827	0.015776	5.795026	0.012075	2447303
0.017873	11.80556	0.163966	0.392172	723.2351	0.014114	5.400702	0.017643	31063.94
0.040935	144.3383	0.21705	0.466399	18.95579	0.021538	5.891971	0.015034	638084.7
0.0183	296.4549	0.195165	0.446594	45.47184	0.007951	6.850386	0.039011	77994.67
0.031859	459.8162	0.235441	0.493735	25.54912	0.017624	6.082838	0.016809	1000388
0.033348	336.2237	0.219324	0.473505	21.00157	0.01707	5.854983	0.009539	4159216
0.031764	255.3818	0.223627	0.473497	26.76941	0.016467	6.093271	0.025981	179731
0.194971	15.77612	0.235464	0.493826	5.362324	0.120034	4.441065	0.016462	99427.17
0.019011	166.9849	0.15798	0.392673	27.37511	0.009022	6.550543	0.00781	3555606
0.063424	124.6412	0.183296	0.418707	13.87677	0.034233	6.003229	0.015888	688047.1
0.011243	17.8172	0.191583	0.412527	812.8373	0.010953	5.083972	0.023628	31168.26
0.085782	25.42667	0.169511	0.37841	7.32572	0.027778	4.98405	0.007536	1154772
0.043835	97.74903	0.188705	0.435759	18.83777	0.020674	6.246709	0.017425	187552
0.096475	28.16176	0.207072	0.459736	8.049173	0.054481	5.101907	0.015224	147933.3
0.063247	98.69383	0.243688	0.504551	14.88625	0.036808	5.515721	0.023275	121513.2
0.067848	19.32353	0.142085	0.348993	13.29671	0.027361	5.636608	0.040225	19294.74
0.073193	188.572	0.196839	0.436636	12.61256	0.039114	6.013532	0.015088	815016.7
0.057441	38.37063	0.268326	0.533504	25.26722	0.043606	5.341451	0.027799	42605.66
0.035891	132.507	0.155525	0.387113	19.67927	0.01538	6.698422	0.017404	393627.6
0.028744	891.7228	0.224276	0.480048	24.14052	0.014295	6.053569	0.008727	12749816
0.052062	14.85263	0.156343	0.392916	15.26432	0.018691	5.382382	0.016427	72019.1
0.029375	314.0169	0.189167	0.43506	23.08063	0.015186	6.394383	0.009959	2414825
0.117192	25.90323	0.278529	0.542799	12.91695	0.078197	4.971797	0.021141	41711.57
0.038825	69.9527	0.236327	0.494992	21.01803	0.023025	5.488101	0.03536	53152.33
0.053111	6.854545	0.124628	0.307409	18.18545	0.025426	5.229277	0.026948	16461.52
0.022875	157.1635	0.210675	0.4663	30.79858	0.011465	6.098213	0.031399	126073.6
0.01684	1202.201	0.202425	0.452367	48.18158	0.008282	6.94932	0.024575	1172377
0.112643	63.16667	0.169803	0.399068	20.45707	0.054142	6.42066	0.028501	46477.26
0.022748	190.8498	0.15416	0.377506	27.23908	0.009565	6.777933	0.011959	1321223
0.019697	194.5184	0.246538	0.50928	47.3719	0.008735	6.008296	0.013566	912860.8
0.079583	34.82	0.1741	0.413667	10.26835	0.045572	5.739332	0.034072	30822.49
0.112218	36.61326	0.202283	0.455817	7.632337	0.060438	5.150595	0.020073	149211.9
0.117338	116.1522	0.180361	0.428733	12.96924	0.05597	6.355992	0.015537	283220.2
0.059249	87.82064	0.215776	0.476746	14.83874	0.031759	5.899035	0.01535	274006.2
0.024366	73.61825	0.137092	0.352591	27.71129	0.009755	6.664785	0.016036	292341.7
0.024601	203.0072	0.183717	0.418302	22.98936	0.011732	6.029458	0.015304	1253545
0.096973	24.23894	0.214504	0.467984	9.063742	0.047629	4.987759	0.029019	28640.73
0.065353	44.22613	0.222242	0.480192	18.74675	0.032494	5.660422	0.022499	55753.19
0.021	96.29045	0.187701	0.424077	31.32532	0.009651	6.120971	0.029737	92171.43
0.037568	278.2106	0.200008	0.444669	27.20245	0.016262	6.295356	0.013737	1630802
0.028595	99.95638	0.16148	0.385248	24.71138	0.011552	6.300975	0.017581	504899.5
0.089729	40.57746	0.190505	0.437049	7.962506	0.048424	5.250983	0.011134	522752.3

0.056346	25.18182	0.208114	0.462469	14.95978	0.029604	5.105459	0.023679	72839.73
0.268952	19.34091	0.219783	0.477001	6.519155	0.159091	4.985706	0.018613	50496.13
0.039611	80.77839	0.223763	0.481775	18.26995	0.021239	5.5829	0.027029	108616.4
0.022497	758.6511	0.166663	0.401611	25.66836	0.010232	6.655605	0.009058	13346115
0.067959	24.86555	0.208954	0.455278	14.00074	0.033172	5.219862	0.023625	25726.49
0.031413	113.5561	0.190211	0.435401	21.18498	0.015057	5.926523	0.019019	429286
0.022521	377.6761	0.231277	0.490731	35.83593	0.010934	6.310972	0.018636	708665.6
0.0207	501.3977	0.223539	0.480591	43.00461	0.010093	6.628464	0.025907	365928.6
0.071146	26.49438	0.148845	0.358978	12.20943	0.02624	5.809023	0.032673	24823
0.047883	28.056	0.224448	0.479287	20.90333	0.029311	5.386455	0.048393	8861.823
0.111899	21.38017	0.176696	0.421323	16.33721	0.06686	5.818784	0.029657	16234.25
0.026222	351.3333	0.264957	0.52518	27.53351	0.014658	5.427192	0.019646	755195.4
0.022507	1029.012	0.248374	0.508877	28.65345	0.011979	5.652507	0.012704	7962450
0.067432	625.6525	0.213752	0.468548	26.40886	0.03567	6.784771	0.020441	947358.6
0.019106	800.548	0.220658	0.476033	37.3127	0.009591	6.437942	0.016551	2525955
0.002285	248.9531	0.194799	0.439824	840.9368	0.00102	5.606434	0.010039	4641920
0.232644	4.37931	0.151011	0.36577	3.131994	0.147651	4.211261	0.020848	18849.34
0.058402	61.70032	0.194638	0.436617	11.85962	0.026791	5.484877	0.018405	213155.7
0.018376	404.5885	0.242705	0.502876	36.14775	0.008839	5.679489	0.012392	2266387
0.133274	57.62112	0.178948	0.406022	7.715178	0.063746	5.556566	0.022473	151613.4
0.031989	445.6363	0.241931	0.499247	29.98066	0.017922	6.191828	0.025595	490705.7
0.044309	53.03077	0.203964	0.45728	16.53303	0.018752	5.351326	0.014412	233614.1
0.019152	630.0798	0.306459	0.569212	45.89253	0.011513	5.713929	0.02515	510392.8
0.025235	266.7249	0.24138	0.500169	35.37278	0.012827	6.091972	0.023503	205898.9
0.031483	186.8169	0.322654	0.588064	33.96104	0.018693	5.250749	0.031601	152465.6
0.043028	334.382	0.234819	0.495362	36.83449	0.022887	6.410675	0.016836	820903.5
0.045448	678.9733	0.216165	0.467779	15.02154	0.021714	5.167851	0.010443	11975190
0.068179	199.4308	0.306817	0.571041	18.28433	0.046564	5.410913	0.025833	147666.5
0.065745	479.6124	0.278682	0.540668	20.81103	0.042565	5.866888	0.024682	506396.4
0.06301	119.3687	0.239216	0.497455	14.34632	0.038357	5.602123	0.023799	130685.3
0.104171	34.16667	0.316358	0.582178	19.76493	0.081395	5.080211	0.036948	9427.894
0.028843	262.486	0.203793	0.454188	29.40933	0.01428	6.534816	0.032221	121825.2
0.033203	340.723	0.296797	0.563679	26.53726	0.021104	5.659607	0.015106	1096141
0.036816	152.0532	0.144537	0.36915	35.23776	0.012729	7.07027	0.013113	1357403
0.056587	79.51381	0.219651	0.473987	17.86818	0.028767	5.876656	0.019553	127687.9
0.025524	147.5579	0.136754	0.362344	24.28099	0.010868	7.029177	0.013404	911267.3
0.052663	40.71598	0.240923	0.49744	21.189	0.028727	5.420185	0.026246	39523.75
0.022511	323.1372	0.211063	0.467526	47.21005	0.010844	6.793732	0.030725	190640.5
0.045359	16.29114	0.206217	0.441321	19.16573	0.021122	5.058165	0.035363	15877.26
0.013014	17.3	0.173	0.380622	695.938	0.010216	5.145819	0.022351	46044.45
0.062333	72.61446	0.174975	0.393598	14.03934	0.027021	6.054465	0.024225	107769.5
0.032842	92.27083	0.160192	0.393758	18.89716	0.014347	6.154874	0.013572	686892.6
0.08939	22.3369	0.119449	0.316711	7.670641	0.035379	6.057058	0.023075	55319.87
0.041868	222.5431	0.17775	0.416833	17.16026	0.018648	6.365955	0.017449	581747.1
0.067922	125.1503	0.261274	0.523253	17.23353	0.033455	5.487607	0.019713	260224.1
0.054402	66.78531	0.188659	0.429221	16.12385	0.028013	6.030288	0.031107	43452.73
0.034929	162.8898	0.264003	0.520673	40.63129	0.02084	5.890529	0.050203	48408.53
0.047604	102.4542	0.199716	0.436022	16.57865	0.025388	5.649554	0.019626	426318.9
0.081769	19.76471	0.145329	0.375854	9.061805	0.038507	5.47345	0.022324	90019.49
0.175645	36.63429	0.209339	0.450506	6.855396	0.106367	5.13661	0.016559	168893.6
0.025141	280.5491	0.165126	0.394764	25.62863	0.009994	6.504057	0.017447	862347.7
0.028601	486.3342	0.149503	0.37013	20.33825	0.010715	6.66544	0.014295	3170214
0.052818	340.5964	0.235381	0.493674	15.75927	0.029351	5.691429	0.014541	1712827
0.01777	57.86721	0.156822	0.378321	33.33072	0.009334	6.30601	0.025048	107459.6
0.037198	76.31967	0.156393	0.384209	21.43304	0.01711	6.335989	0.020641	225903.7
0.054679	474.8285	0.290771	0.554435	16.79002	0.033259	5.276185	0.011982	2975422
0.048449	100.7732	0.187311	0.427486	17.25889	0.02127	5.880557	0.025954	190103.1
0.046762	185.4913	0.180089	0.420425	18.28856	0.021857	6.28916	0.018004	605990.8

0.03545	201.0063	0.316545	0.58088	29.24638	0.021782	5.330027	0.026579	172692.8
0.124259	32.53052	0.152725	0.36424	7.771441	0.050691	5.789428	0.017327	120201.8
0.019321	515.9036	0.206279	0.459016	45.63672	0.009586	6.869654	0.019693	687495.5
0.021606	19.01163	0.110533	0.311594	28.20728	0.006087	6.110038	0.018207	96349.56
0.060263	115.3583	0.261583	0.527787	18.26729	0.035329	5.532152	0.039644	54556.42
0.022981	204.1156	0.133322	0.349335	27.74718	0.00888	7.107343	0.011634	1394253
0.121674	32.37313	0.16106	0.383087	11.42014	0.042404	5.814978	0.018776	87452.29
0.121025	205.44	0.253943	0.508462	9.588086	0.062442	4.707916	0.020549	725109.9
0.047163	277.7572	0.294546	0.561953	26.79624	0.028278	5.884722	0.018973	411568.9
0.033489	91.82943	0.153561	0.375478	24.85589	0.014035	6.67446	0.027386	88466.68
0.014162	151.3935	0.163669	0.397308	47.31477	0.005024	6.840061	0.020737	243968.6
0.090921	108.1443	0.273783	0.538432	12.23608	0.054791	5.159971	0.028874	127027.2
0.004646	126.1158	0.149073	0.366898	718.5507	0.0004	6.413583	0.015105	764112.5
0.049205	128.8084	0.135588	0.34788	17.68398	0.016648	6.886796	0.01241	753519.2
0.031793	384.4014	0.266945	0.529087	29.04235	0.018234	5.665204	0.020439	811060.9
0.052263	114.548	0.203822	0.44903	21.03866	0.024021	6.177999	0.02422	89367.83
0.042001	386.8508	0.313748	0.575735	21.90483	0.024786	4.930705	0.022097	704246.2
0.033916	683.4839	0.219629	0.47474	23.16568	0.016568	6.219867	0.009773	5859563
0.120318	27.0292	0.197293	0.446109	13.44698	0.05729	5.640171	0.024069	32926.96
0.003841	49.04027	0.164565	0.39952	811.2213	0.000199	5.862083	0.016632	161640.9
0.06318	31.7766	0.169024	0.408683	14.68714	0.034026	5.793026	0.022488	69478.7
0.001423	204.6285	0.189121	0.432173	869.4942	0.000218	6.523856	0.037113	85734.58
0.028073	80.8821	0.176598	0.415633	29.42901	0.012798	6.491279	0.02736	52436.13
0.039643	99.26331	0.278048	0.540536	29.02622	0.024331	5.58447	0.05615	19959.16
0.030336	269.4865	0.169382	0.398406	18.67687	0.013399	6.07223	0.011041	3250223
0.079171	72.06596	0.190148	0.432225	18.44142	0.040741	6.002914	0.015348	162399.9
0.049964	82.74272	0.200832	0.451101	16.82085	0.025488	5.967965	0.027711	68423.36
0.043337	168.8551	0.244717	0.500487	16.63959	0.022551	4.739458	0.018357	915708.8
0.126654	21.0531	0.186311	0.427249	10.86932	0.070678	5.339399	0.029604	27600.44
0.055322	68.72868	0.26639	0.531142	29.84659	0.032877	5.820182	0.037016	28258.5
0.138154	16.43284	0.245266	0.48991	7.618499	0.062629	4.517474	0.081016	1910.285
0.014508	45.2008	0.181529	0.431405	53.57525	0.006142	6.098037	0.032651	25479.09
0.003279	112.2362	0.188	0.431572	439.0762	0.001268	6.273021	0.016113	461931.1
0.034635	267.2204	0.245381	0.503219	23.56786	0.01727	5.391344	0.021058	762579
0.131023	41.31373	0.270024	0.524084	10.45953	0.069792	4.355792	0.026664	71895.31
0.183531	10.37778	0.230617	0.491593	7.332114	0.108951	4.4606	0.024298	23049.73
0.058596	38.46411	0.184039	0.436927	22.88123	0.030313	6.005334	0.02999	11502.02
0.062819	63.77524	0.207737	0.459054	16.01876	0.024467	5.494457	0.012429	559310.7
0.026513	101.8262	0.229856	0.491111	28.57394	0.013759	5.68782	0.012497	807106.5
0.035635	117.7902	0.143647	0.362101	21.87515	0.012528	6.6244	0.02051	269020.9
0.040936	51.38095	0.203893	0.461668	21.99607	0.021518	5.784741	0.018204	155018.7
0.108454	31.88889	0.272555	0.533148	12.54786	0.05	4.578718	0.046117	14239.67
0.090406	113.14	0.255395	0.518905	18.77939	0.048222	5.764182	0.025011	118681.4
0.120646	7.125	0.148438	0.359042	10.82351	0.038284	5.001629	0.044818	3656.465
0.047783	24.69231	0.211045	0.471108	17.79468	0.016194	4.997741	0.012057	236047.8
0.029783	63.62264	0.200071	0.449357	26.17265	0.017182	6.003424	0.023241	86787.27
0.038463	107.9684	0.284127	0.545681	30.85058	0.023567	5.479054	0.052141	29097.18
0.052247	164.7992	0.2177	0.46521	15.31348	0.025477	5.765841	0.013216	831617.4
0.075448	48.04673	0.224517	0.486445	16.13249	0.031033	5.6754	0.028552	42590.28
0.041365	230.6533	0.200918	0.44541	20.29001	0.020989	5.964876	0.015282	1353878
0.064032	77.44318	0.220009	0.457191	16.24829	0.029504	5.657342	0.029392	65143.42
0.081855	57.82305	0.237955	0.496969	17.49015	0.041123	5.528354	0.030352	55624.73
0.263098	4	0.153846	0.374171	4.560507	0.087109	4.257756	0.046512	1582.942
0.025798	163.3216	0.238774	0.492429	31.85203	0.0126	5.400182	0.036431	138691.5
0.026343	168.8646	0.265928	0.525738	33.51301	0.015015	5.570107	0.037942	88584.91
0.150423	9.90625	0.154785	0.365746	5.881558	0.0448	4.863609	0.03805	10175.76
0.00181	292.5667	0.223163	0.480766	818.6731	0.000835	6.23246	0.010729	1558017
0.042155	98.46292	0.251823	0.508576	28.05621	0.021112	5.731885	0.049201	24743.64

0.073041	203.3285	0.243799	0.500745	14.42945	0.039518	5.59833	0.020529	498509.6
0.048549	59.94156	0.194615	0.436675	16.02371	0.025609	5.862322	0.018199	229817.8
0.051593	87.56047	0.25829	0.521234	22.24791	0.029597	5.091044	0.044847	46335.91
0.093448	38.45455	0.183993	0.418967	11.50113	0.044833	5.662897	0.023777	67626.96
0.01951	254.1231	0.240647	0.492301	36.31536	0.009999	5.505708	0.030864	282501.5
0.067112	57.43655	0.291556	0.558882	20.75087	0.03368	5.143583	0.038529	22935.68
0.037764	89.59211	0.147355	0.377527	24.57552	0.013755	6.833593	0.0251	105441.3
0.038444	133.7054	0.259119	0.517404	23.14574	0.022983	5.580677	0.044819	49818.17
0.021581	3857.804	0.288111	0.550873	67.87162	0.011665	6.149836	0.026414	6156331
0.035151	119.5891	0.217435	0.469336	22.99497	0.018886	5.770787	0.030809	174474.1
0.050651	31.40909	0.178461	0.416853	19.79524	0.027271	5.631614	0.035341	32869.58
0.023138	567.1835	0.18255	0.417136	27.5532	0.010654	6.244308	0.010238	10219544
0.062012	146.9326	0.183436	0.425711	15.24987	0.030907	6.276116	0.018006	342742.3
0.029676	496.5478	0.244605	0.50725	31.72235	0.016702	6.357073	0.025495	644017.1
0.116991	6.1875	0.193359	0.393015	8.883247	0.039169	4.51532	0.032161	5966.147
0.037837	67.23171	0.204975	0.458233	23.07425	0.018449	5.816035	0.026628	85509.55
0.035021	321.2454	0.229134	0.484034	24.98529	0.017735	6.03788	0.019045	908683.3
0.042944	261.6575	0.277473	0.539763	21.94832	0.024625	5.32835	0.033544	208782.8
0.113324	22.20325	0.180514	0.41258	16.16874	0.068629	5.546054	0.023851	39197.74
0.104457	28.72289	0.173029	0.415419	9.400598	0.044475	5.37893	0.022881	83386.75
0.037053	101.8444	0.161658	0.402059	19.52229	0.016551	6.463201	0.011818	628342.1
0.093073	4.586207	0.158145	0.351714	11.03626	0.045788	4.254196	0.031694	6577.42
0.043974	272.9842	0.215118	0.455539	15.249	0.020262	5.445088	0.01386	2117003
0.03306	147.3706	0.208445	0.457506	26.63332	0.017117	6.193173	0.01872	301361.5
0.14472	18.3	0.305	0.565506	17.25944	0.081368	4.727376	0.126582	263.4567
0.079839	116.5779	0.221631	0.476646	14.71906	0.041345	5.787728	0.024195	202003.1
0.03563	148.7805	0.166608	0.39688	20.32904	0.01709	6.361896	0.01124	1469249
0.114261	91.57292	0.238471	0.497987	11.19857	0.061813	5.46975	0.017033	235092
0.0406	154.0684	0.219471	0.471794	24.33424	0.020912	5.894248	0.034856	122349.8
0.113655	10.93443	0.179253	0.422768	11.03482	0.074566	5.100751	0.040694	4754.441
0.039667	228.7524	0.318154	0.582928	31.35739	0.026445	5.5607	0.055062	37848.39
0.049249	60.69143	0.173404	0.415981	19.20159	0.02101	5.996951	0.023769	134554.3
0.044402	96.77083	0.252007	0.512445	28.5588	0.027842	5.94155	0.045709	25906.87
0.073227	101.2699	0.207096	0.453915	14.29657	0.038754	5.880756	0.039176	43673.77
0.095981	4.769231	0.183432	0.349429	9.661289	0.029882	4.10391	0.111588	220.4985
0.053442	21.92157	0.214917	0.466165	14.45767	0.030133	4.847787	0.007947	715821.8
0.097422	10.83333	0.180556	0.42153	7.851391	0.042463	4.70291	0.04481	6748.083
0.036674	111.6709	0.238613	0.487994	19.58438	0.020674	4.887764	0.010702	1799078
0.034805	277.4783	0.177416	0.418135	20.4976	0.0167	6.37381	0.011279	2569427
0.008263	51.19487	0.262538	0.509961	874.3055	0.002099	4.0702	0.012743	558029.8
0.047052	98.21138	0.199617	0.448803	16.50052	0.020361	5.85018	0.01087	902224.4
0.04361	54.76724	0.236066	0.477491	15.82634	0.021144	4.598259	0.028646	152369.1
0.036901	45.96364	0.208926	0.449083	19.10152	0.019711	5.073442	0.035755	79120.46
0.081481	17.04959	0.140906	0.345483	9.897339	0.023019	5.254697	0.012107	362118.9
0.041213	161.8533	0.242295	0.500992	26.18924	0.022781	5.797375	0.024849	387753.7
0.029297	947.1049	0.297458	0.561167	28.91667	0.016181	5.091818	0.018826	3189695
0.062584	71.86942	0.118792	0.322584	8.802413	0.021974	6.166161	0.008844	2823434
0.035315	46.18681	0.253774	0.513586	26.23229	0.015073	5.102312	0.034437	47118.84
0.170331	56.18519	0.260117	0.522757	6.95207	0.100795	4.585122	0.017853	282138.2
0.058046	29.6087	0.257467	0.5192	18.22059	0.03553	5.204757	0.031962	25609.61
0.00258	431.1259	0.27977	0.542967	497.5295	0.001385	5.401622	0.013674	3191487
0.047273	18.03371	0.202626	0.448181	21.83102	0.028352	5.326512	0.036475	18545.82
0.030727	414.2238	0.237106	0.495559	25.99177	0.016034	5.31783	0.025737	931493
0.172748	6.071429	0.216837	0.467113	7.64707	0.083069	4.110577	0.022876	23674.35
0.088854	33.0479	0.197892	0.438219	10.82296	0.040809	5.10702	0.02867	61907.35
0.139214	34.08629	0.173027	0.419872	13.35906	0.045526	5.823265	0.017577	188852.8

0.002892	269.1898	0.297119	0.559761	436.9304	0.002269	5.172679	0.015857	1743198
0.026129	987.5545	0.237279	0.495257	39.51789	0.013106	6.509641	0.020918	1659146
0.030243	620.2864	0.237203	0.490683	20.60207	0.01484	4.977058	0.015137	4844605
0.020188	605.5138	0.297404	0.561728	197.4381	0.011025	6.48261	0.022917	1479015
0.027538	213.0072	0.256635	0.511623	30.2395	0.016412	5.502254	0.021979	557948.9
0.087128	51.12245	0.208663	0.459375	12.8761	0.045693	5.171851	0.017313	326162.6
0.067531	44.37662	0.192107	0.432476	14.31384	0.039355	5.561663	0.017897	233144.9
0.0467	92.24775	0.207765	0.461287	16.31615	0.024642	5.485332	0.012007	1113607
0.131006	19.66019	0.190876	0.441408	12.28028	0.043251	5.059065	0.016414	113505.9
0.10358	56.33333	0.231824	0.491856	10.17921	0.060935	5.17036	0.034854	59617.14
0.065302	26.37864	0.128052	0.341356	10.94764	0.020308	5.901914	0.015389	234249.4
0.140854	45.59873	0.145219	0.361419	6.326952	0.057675	5.605187	0.014304	494347
0.336481	4.803922	0.094195	0.236647	2.328129	0.120235	5.030173	0.017074	50750.05
0.343884	19.86239	0.182224	0.4089	3.86372	0.140572	4.658459	0.022213	81165.12
0.089947	64.82609	0.165796	0.394858	7.673669	0.039969	5.467518	0.011229	1327733
0.122815	6.534884	0.151974	0.371074	6.567194	0.038913	4.643672	0.019361	41927.86
0.182077	20.32584	0.22838	0.486141	6.100015	0.091673	4.571725	0.008239	698092.3
0.033779	522.1353	0.255073	0.516587	26.129	0.019228	6.021202	0.017662	986496.8
0.032998	235.9697	0.179036	0.414174	22.53363	0.015063	6.141035	0.021857	801206.4
0.053707	170.9275	0.247721	0.505722	17.15431	0.030571	5.359758	0.023706	330690.5
0.040099	179.6837	0.287035	0.549829	22.29943	0.023816	4.906902	0.022319	494570.2
0.030829	503.9688	0.234732	0.490139	25.7635	0.015593	6.053646	0.015872	1678138
0.037598	126.721	0.271934	0.534401	21.84282	0.018897	4.917966	0.010633	1742888
0.163709	2.882353	0.16955	0.363511	3.777412	0.094766	3.734522	0.149123	110.7958
0.041158	70.96319	0.217678	0.471489	20.66266	0.022953	5.143543	0.032695	130451.5
0.043505	202.772	0.194226	0.435697	21.28391	0.018514	5.984662	0.015254	1322149
0.026046	328.1259	0.237428	0.493414	34.30153	0.012336	5.886274	0.016048	1374678
0.027179	221.3207	0.233461	0.491615	39.53443	0.011484	6.043981	0.03281	257564.1
0.074164	14.69444	0.20409	0.449828	15.17289	0.050607	5.053529	0.048193	8625.854
0.11222	9.416667	0.196181	0.443309	15.3472	0.083873	4.928509	0.042553	7177.083
0.036983	62.12376	0.153772	0.373776	22.7892	0.013086	6.225536	0.016856	484298.1
0.031972	43.65025	0.215026	0.464865	29.08467	0.016755	5.568376	0.039144	31110.44

AX103	AX104	AX105	AX106	AX107	DX1	DX2	DX3	DX4
50.47808	0.000334	26.10118	0.010021	0.018406	0.900022	0.842287	30.5115	36.2246
83.29374	0.000205	13.74294	0.009334	0.007986	0.780375	0.738688	29.29693	39.66074
57.43006	0.000253	12.37313	0.005878	0.010209	0.886196	0.797146	39.18518	49.15685
4.046915	0.005377	12.18758	0.019314	0.132647	0.877353	0.634293	9.816471	15.47625
11.71461	0.001504	22.29688	0.032144	0.045793	0.882701	0.845169	23.70064	28.0425
47.47821	0.000367	15.94425	0.009153	0.015648	0.861369	0.773092	34.45639	44.56961
7.839803	0.000924	46.62243	0.01445	0.074079	0.688757	0.566113	31.073	54.8883
19.37613	0.000522	29.73998	0.01024	0.029541	0.864047	0.704454	31.78549	45.12073
4.295289	0.005844	13.86704	0.06444	0.108794	0.937061	0.84557	14.79145	17.49289
19.8028	0.000216	144.4333	0.025274	0.02983	0.869474	0.689624	45.31088	65.70372
218.5073	4.23E-05	18.87431	0.003689	0.002927	0.800016	0.764364	77.12592	100.9021
351.9023	1.86E-05	46.55765	0.003173	0.003514	0.762553	0.682127	87.92689	128.901
52.76532	0.000283	23.3721	0.017922	0.011247	0.84525	0.799928	41.77297	52.2209
0.824943	0.001909	194.9286	0.350355	2.051319	0.980329	0.942911	24.28028	25.75035
2.99552	0.00541	14.49073	0.013367	0.16952	0.941572	0.737129	15.1979	20.6177
101.5511	7.95E-05	31.04306	0.007115	0.006165	0.774426	0.760488	62.65455	82.38724
0.769729	0.002716	117.5878	0.090336	3.158451	0.964311	0.866921	19.63961	22.65445
40.59546	0.000204	49.82873	0.015355	0.022737	0.832889	0.717083	42.89314	59.81614
17.77183	0.000241	171.4526	0.02489	0.043598	0.911536	0.853884	42.92691	50.27256
68.40801	9.58E-05	60.83417	0.017496	0.009583	0.930283	0.886743	70.01739	78.9602
156.15	5.93E-05	29.59827	0.007846	0.004456	0.808173	0.764206	71.90998	94.0976
41.38861	0.000237	52.79406	0.03299	0.015011	0.892179	0.792774	49.01677	61.82943
16.82018	0.001847	6.204899	0.012727	0.029464	0.890086	0.809425	20.76862	25.6585
66.18997	7.41E-05	70.91987	0.006425	0.013156	0.828062	0.747839	52.57025	70.29619
42.85306	0.000203	32.35626	0.008186	0.014955	0.875905	0.804927	44.22091	54.93777
0.877814	0.002104	74.70074	0.029232	1.710523	0.949919	0.780806	20.73578	26.5569
54.84392	0.000356	10.41759	0.00451	0.013179	0.922423	0.690638	29.94251	43.35482
37.0191	0.000326	56.7174	0.04035	0.020127	0.781738	0.642857	32.35205	50.32543
26.06957	0.000912	13.62549	0.017128	0.02272	0.80412	0.762403	29.31506	38.45089
26.80136	0.000494	36.13691	0.025676	0.022085	0.934434	0.87405	35.72348	40.87119
5.172455	0.002047	33.29855	0.032589	0.100846	0.689671	0.641044	19.65245	30.65694
68.2255	0.000162	42.82526	0.012395	0.01486	0.938059	0.811192	51.03643	62.91534
5.420793	0.001566	35.86081	0.015662	0.10555	0.905772	0.779696	23.84138	30.5778
46.32083	0.000189	76.85509	0.026161	0.02033	0.692503	0.649144	46.93614	72.30468
301.4152	2.29E-05	47.49736	0.007653	0.003381	0.805358	0.764101	100.2281	131.1714
8.659483	0.001561	20.94053	0.02139	0.067257	0.885094	0.78309	26.33949	33.63534
102.1626	7.24E-05	67.53545	0.014057	0.009929	0.706578	0.643719	66.07592	102.6471
9.534491	0.002036	25.0491	0.020972	0.089184	0.864783	0.836468	20.74491	24.80061
8.57601	0.000885	43.65717	0.019574	0.071062	0.801148	0.756375	27.06967	35.7887
2.1527	0.00379	37.7346	0.020105	0.274036	0.842731	0.731658	18.57144	25.38268
13.99865	0.000349	88.5479	0.013934	0.042011	0.683187	0.591109	33.1674	56.11043
96.53032	4.29E-05	146.9107	0.021132	0.008174	0.918042	0.848394	82.42689	97.15637
21.04174	0.000698	66.75365	0.060707	0.041815	0.928793	0.905958	31.58348	34.86196
46.1289	0.000101	93.08393	0.01029	0.017683	0.787014	0.741078	58.43869	78.85629
14.56834	0.000177	189.2229	0.001956	0.202912	0.858139	0.625142	48.82255	78.09837
8.535586	0.001389	32.24703	0.035351	0.056568	0.96294	0.86924	22.42667	25.80034
19.79316	0.00093	12.76724	0.011413	0.024367	0.841399	0.708779	23.18437	32.71029
62.53618	0.000228	50.12525	0.048375	0.012144	0.856223	0.752115	42.77572	56.87388
46.53817	0.000358	27.49674	0.031945	0.012114	0.791031	0.672703	40.30794	59.91937
17.74656	0.000307	62.8659	0.015104	0.033734	0.664546	0.404375	29.25416	72.34411
48.29944	0.00012	43.19293	0.00536	0.014709	0.858292	0.751438	51.76367	68.88617
10.19508	0.001917	18.91315	0.033624	0.045882	0.968027	0.932048	23.27346	24.97023
14.27226	0.000984	38.42418	0.035241	0.042605	0.831866	0.711958	29.47645	41.40197
9.695771	0.000492	79.11186	0.016522	0.0605	0.62474	0.528366	28.84141	54.58605
38.62375	9.86E-05	131.4027	0.00301	0.053796	0.731633	0.686309	58.17802	84.76937
24.24625	0.000229	49.17949	0.011836	0.026391	0.932545	0.870004	41.45557	47.64986
48.45646	0.000453	12.08364	0.012229	0.011918	0.853103	0.779065	31.50093	40.43428

8.596329	0.001529	17.57364	0.009547	0.060373	0.901428	0.775256	24.2887	31.32989
14.96803	0.002002	13.75646	0.029814	0.043771	0.856184	0.809959	23.10098	28.52118
11.56828	0.000604	48.56707	0.011844	0.049426	0.843945	0.68412	28.91384	42.26426
260.9227	1.98E-05	64.73256	0.004494	0.004224	0.854831	0.720384	93.278	129.4837
11.2314	0.001528	34.24262	0.053026	0.053461	0.903752	0.875143	23.0883	26.38231
28.29373	0.000255	42.57316	0.008811	0.023094	0.908693	0.674546	43.9454	65.14813
46.54596	0.000123	70.94771	0.015214	0.014449	0.868154	0.748375	60.25745	80.51777
37.74832	0.000123	122.9713	0.018673	0.021	0.781195	0.635888	57.97927	91.17844
9.383291	0.001432	34.36739	0.040919	0.052663	0.821671	0.758709	24.5134	32.30937
2.98848	0.003149	39.52709	0.025431	0.175658	0.746	0.732237	19.55556	26.70659
3.175476	0.002768	48.21478	0.036731	0.16579	0.926962	0.773532	20.67274	26.72514
55.75163	0.000133	41.65289	0.009153	0.011367	0.850859	0.63932	48.75762	76.26486
246.9603	2.52E-05	42.27071	0.00446	0.003282	0.922997	0.74418	89.25412	119.9362
89.34647	8.03E-05	169.8248	0.021106	0.031106	0.94271	0.879968	68.55867	77.91043
105.6576	4.76E-05	73.62568	0.01147	0.007737	0.972109	0.77547	77.68477	100.1777
11.553	6.39E-05	129.0388	0.002407	0.166481	0.710168	0.613425	59.97562	97.77167
6.841324	0.005322	7.417199	0.025907	0.069038	0.903709	0.742821	13.77195	18.54008
28.93974	0.000469	25.40535	0.015873	0.019383	0.689715	0.670821	33.56615	50.03738
91.33232	6.30E-05	34.17171	0.004258	0.008298	0.750198	0.720194	227.098	315.3288
41.45092	0.000568	14.61585	0.021476	0.013239	0.847891	0.809757	31.86466	39.35092
36.31131	0.000121	167.5306	0.007691	0.049123	0.7808	0.618809	49.79192	80.46412
26.01539	0.000492	25.9428	0.020773	0.021127	0.891694	0.845752	36.67041	43.35837
40.3254	0.000131	68.58105	0.012428	0.016167	0.880768	0.835992	34.64982	41.44753
27.3778	0.000224	76.7418	0.033246	0.021351	0.880302	0.829725	50.36096	60.69598
13.43909	0.000443	52.15186	0.007477	0.047262	0.94029	0.800562	20.62952	25.76879
24.91651	0.000118	428.2614	0.003171	0.238662	0.87818	0.73636	59.33677	80.58123
423.2286	2.22E-05	28.5405	0.00333	0.002655	0.648315	0.609183	82.59492	135.5831
44.98479	0.000374	27.49943	0.034089	0.013046	0.837772	0.738879	44.1051	59.69191
55.04946	0.000148	51.50581	0.013273	0.013775	0.672931	0.592594	27.7721	46.86529
31.06895	0.000456	34.55985	0.030156	0.019741	0.902886	0.802047	37.65684	46.95092
3.953027	0.003039	40.22141	0.049743	0.133198	0.942461	0.845245	18.55248	21.94923
23.46473	0.000233	114.2509	0.027244	0.02687	0.806439	0.696672	42.27864	60.6866
85.9263	0.000107	47.56955	0.016827	0.009274	0.751328	0.684344	65.18936	95.25819
37.27626	0.000103	256.2871	0.003566	0.125356	0.963811	0.763962	39.50882	51.7157
31.13438	0.000519	35.79471	0.039187	0.022004	0.845397	0.718051	28.05892	39.07651
50.77895	0.000105	123.3598	0.01789	0.018686	0.818638	0.758529	40.78024	53.76226
5.986523	0.001507	48.4781	0.017633	0.103493	0.776171	0.659205	19.84755	30.10831
27.3515	0.000189	170.703	0.022604	0.042836	0.897639	0.713031	42.33299	59.37049
2.572491	0.003415	31.27154	0.010131	0.229627	0.694161	0.627102	13.97645	22.28736
1.140871	0.001738	103.2483	0.094065	3.019643	0.91849	0.704092	14.54167	20.65308
20.86977	0.000585	30.8211	0.023578	0.027195	0.748614	0.741157	25.01095	33.74583
44.38598	0.000195	38.90514	0.012082	0.012969	0.860417	0.842821	36.53151	43.3443
14.22365	0.000971	28.77208	0.025683	0.036453	0.900469	0.777468	19.29427	24.81679
69.80711	0.000132	51.4997	0.024285	0.008755	0.865943	0.79903	44.40862	55.57819
31.63149	0.000404	22.01437	0.016919	0.016966	0.846254	0.718096	37.06244	51.61211
14.47469	0.00068	57.97956	0.045902	0.035229	0.838529	0.729599	23.11022	31.67526
5.471423	0.000681	149.0742	0.007093	0.24599	0.880557	0.803522	31.63035	39.36464
27.4457	0.00029	34.4357	0.006642	0.022782	0.838317	0.762441	38.44806	50.42762
14.34518	0.001148	15.07626	0.013287	0.03636	0.80189	0.76957	20.44948	26.57261
37.36022	0.000783	10.17541	0.023939	0.013362	0.937381	0.822188	29.0181	35.29373
73.23154	9.73E-05	50.21602	0.019921	0.00768	0.720616	0.561994	41.22758	73.35948
151.1717	3.96E-05	69.73709	0.007459	0.0063	0.764609	0.739497	63.83205	86.31824
105.2736	8.83E-05	40.52602	0.007465	0.008968	0.90617	0.738118	58.52294	79.28668
9.864426	0.000518	67.8435	0.023527	0.062787	0.93085	0.879818	26.91191	30.58804
18.75992	0.000347	50.64759	0.014125	0.029989	0.817797	0.700167	26.05762	37.21629
177.0795	6.70E-05	31.0265	0.008441	0.006049	0.860041	0.742261	80.57847	108.5581
26.32852	0.000389	31.87957	0.013855	0.020553	0.764193	0.704053	30.54284	43.38144
55.04845	0.000153	47.11317	0.014203	0.011859	0.836579	0.727983	39.06391	53.66048

17.39058	0.000379	63.50208	0.009537	0.045165	0.897507	0.840398	25.59276	30.45314
32.42169	0.000668	29.55476	0.031622	0.027517	0.911261	0.807994	23.79962	29.4552
21.58516	9.41E-05	360.0392	0.005642	0.093231	0.663852	0.642101	66.78105	104.0039
5.416558	0.00084	76.11003	0.014779	0.128201	0.905611	0.806953	21.32932	26.43193
21.22181	0.000671	30.40527	0.02243	0.02499	0.873886	0.396002	12.35706	31.20457
65.10809	8.07E-05	106.0575	0.016807	0.012703	0.730897	0.624914	46.90251	75.0543
33.39501	0.000724	25.45653	0.04353	0.019432	0.874408	0.825394	21.47128	26.01338
153.3883	0.000164	10.17641	0.0131	0.003545	0.886324	0.836662	45.93771	54.90594
27.63025	0.000246	53.86421	0.014716	0.024729	0.873822	0.763077	22.27552	29.19172
14.98189	0.000377	102.0153	0.049143	0.034334	0.910698	0.853836	28.91377	33.86336
13.85596	0.000206	192.1653	0.015041	0.05195	0.90229	0.75489	33.92426	44.93932
27.764	0.000549	20.25168	0.015444	0.018338	0.775756	0.730932	32.10772	43.9271
4.255403	0.000165	185.8285	0.01443	0.291184	0.866875	0.802661	39.43652	49.13221
51.34026	0.000153	58.4271	0.031588	0.010732	0.865622	0.78863	44.98553	57.04264
50.3879	0.000132	39.76037	0.010486	0.011994	0.682035	0.669617	59.74081	89.21638
23.55332	0.000421	60.88074	0.049438	0.024029	0.838549	0.78747	31.37336	39.84072
81.08576	0.000146	22.72079	0.010327	0.007016	0.932881	0.82256	53.66765	65.24464
189.9144	3.74E-05	60.42695	0.009515	0.005797	0.70031	0.569792	76.65096	134.5245
9.410807	0.00149	37.44191	0.034709	0.067007	0.882541	0.660433	15.73202	23.82075
2.918793	0.00053	90.88685	0.138461	0.403636	0.721352	0.699087	26.2579	37.56028
13.20854	0.000978	35.04848	0.018822	0.053465	0.90358	0.836629	21.24194	25.38991
2.996133	0.000305	203.8486	0.067934	0.202136	0.809564	0.725646	32.18969	44.36006
8.23293	0.000576	139.0907	0.039799	0.072789	0.909271	0.804926	25.80654	32.06075
6.057422	0.001192	59.18446	0.020781	0.088588	0.869096	0.845161	29.19638	34.54534
161.1919	6.05E-05	28.00898	0.009628	0.003671	0.800959	0.649588	49.43958	76.10911
33.68917	0.00042	37.38963	0.085784	0.018075	0.927729	0.801373	31.27563	39.02756
37.60654	0.000573	40.51605	0.060484	0.026299	0.923506	0.921198	37.60719	40.82424
54.38804	0.000186	19.54576	0.003902	0.01215	0.794605	0.713229	48.90702	68.57124
9.98806	0.002	16.14225	0.022426	0.046036	0.727282	0.623791	20.5123	32.88328
5.277317	0.001144	95.68326	0.015733	0.144562	0.789047	0.67374	26.59938	39.48017
3.38288	0.007952	14.16332	0.046161	0.141703	0.864489	0.577575	13.961	24.17177
3.721682	0.001064	126.0234	0.029996	0.14156	0.790227	0.73115	27.58405	37.72694
2.629309	0.00024	463.9245	0.002877	0.839701	0.769011	0.677243	50.89099	75.14437
69.31735	0.000144	25.81692	0.011895	0.007993	0.821953	0.76912	58.8955	76.57514
21.8594	0.001255	11.06787	0.015729	0.021789	0.62649	0.582243	22.42913	38.52194
9.085378	0.003851	7.666603	0.021877	0.05097	0.702204	0.570356	14.73123	25.82813
12.22592	0.001242	68.54076	0.077337	0.071906	0.791235	0.668988	30.49202	45.57935
45.04968	0.000332	17.54821	0.014057	0.011761	0.854185	0.742885	42.00819	56.5474
28.40659	0.000233	39.42899	0.006187	0.022806	0.789044	0.617713	44.83531	72.58277
30.97732	0.00024	53.99707	0.030877	0.017213	0.836672	0.760567	37.67687	49.53789
15.8205	0.000582	36.40519	0.020431	0.034002	0.915382	0.725742	35.58936	49.0386
8.912945	0.002647	17.52268	0.023612	0.061281	0.542011	0.490176	20.66621	42.16081
27.19882	0.000518	35.25894	0.02945	0.022233	0.703425	0.627603	47.46274	75.62547
2.858106	0.007747	22.31572	0.046327	0.212203	0.756464	0.624513	14.95671	23.94939
13.96619	0.000875	18.07419	0.013784	0.041423	0.63988	0.55357	33.11132	59.81418
10.46656	0.000681	53.20926	0.021041	0.053197	0.926291	0.656649	28.14505	42.86165
7.228348	0.001002	51.08114	0.023117	0.072249	0.902074	0.81781	28.75044	35.15541
78.8486	0.000184	21.28324	0.017885	0.007374	0.870737	0.761385	60.16715	79.02326
13.4407	0.000971	34.14064	0.043105	0.035958	0.761708	0.67041	31.67873	47.25279
79.02661	0.000113	27.79061	0.010423	0.007506	0.970597	0.786795	52.73875	67.02981
21.87373	0.000679	32.03759	0.035721	0.02418	0.88773	0.6438	30.73538	47.74055
11.02211	0.001022	34.95698	0.020864	0.048705	0.678679	0.629514	32.95155	52.34447
10.26628	0.009486	7.005408	0.077774	0.069124	0.659277	0.591769	13.22325	22.34529
11.28536	0.000383	101.691	0.00443	0.103818	0.577509	0.482646	28.49716	59.04356
16.53503	0.000436	49.48977	0.017952	0.033963	0.980651	0.911403	45.13877	49.52669
7.71857	0.004446	11.39073	0.025101	0.075498	0.954949	0.74827	18.3624	24.53982
8.245804	8.23E-05	229.2468	0.004815	0.175165	0.386928	0.25193	62.82078	249.358
7.918286	0.001044	47.35921	0.025854	0.064437	0.832777	0.750503	30.98206	41.28174

57.83739	0.000198	30.76694	0.012936	0.010834	0.909098	0.79266	53.71615	67.76694
19.97146	0.000493	30.5861	0.013738	0.026795	0.621298	0.555685	33.2618	59.85735
12.20763	0.000837	32.68254	0.022402	0.045033	0.816777	0.777393	34.64375	44.56402
16.62027	0.00094	22.59864	0.033577	0.030662	0.811295	0.727399	31.477	43.27338
26.17196	0.000223	47.85349	0.008345	0.024743	0.692302	0.559636	44.09442	78.79129
8.778125	0.001542	31.24683	0.030177	0.057305	0.949975	0.92574	35.57884	38.43286
17.60869	0.000338	98.74028	0.025388	0.037274	0.829546	0.710272	39.95267	56.24981
11.85864	0.000671	46.77489	0.023326	0.044106	0.666906	0.595456	34.2526	57.52327
90.49988	1.41E-05	1302.261	0.000643	0.29384	0.824594	0.705361	117.3465	166.3637
18.20146	0.000417	32.37141	0.012451	0.034923	0.772495	0.652628	38.20612	58.542
6.861872	0.001492	29.54246	0.02081	0.082319	0.750888	0.572418	23.42469	40.92236
157.65	2.92E-05	43.92226	0.003326	0.00489	0.897939	0.824488	92.83216	112.5937
58.58735	0.000217	41.36723	0.026231	0.01209	0.715968	0.708408	55.1649	77.87164
54.45661	0.000103	91.0931	0.01572	0.018346	0.851604	0.799465	70.83896	88.60792
3.94564	0.007634	11.16465	0.025539	0.133929	0.814081	0.632622	13.89646	21.96645
18.10073	0.000603	33.33187	0.025775	0.029367	0.859377	0.849849	41.25074	48.53893
70.8807	0.000111	40.18221	0.013358	0.007574	0.783852	0.669459	60.81682	90.8447
36.78899	0.000267	33.14396	0.012216	0.014894	0.746774	0.581216	45.67315	78.5821
7.635784	0.001603	29.93137	0.037753	0.072287	0.879265	0.81352	27.30157	33.55979
18.16419	0.001046	15.49698	0.022596	0.027082	0.735862	0.727473	30.71731	42.22467
48.53728	0.000209	44.24741	0.021803	0.013953	0.807377	0.696373	45.69902	65.62433
3.106428	0.008135	11.10148	0.022589	0.168644	0.934971	0.771718	12.43197	16.10948
114.7372	9.19E-05	23.25224	0.005157	0.006379	0.94015	0.824642	65.95202	79.97654
28.62012	0.000281	53.20097	0.020131	0.023207	0.887759	0.816912	53.61961	65.63696
1.957992	0.011291	41.592	0.21905	0.322701	0.600736	0.591516	13.65792	23.08969
35.29852	0.000391	24.01187	0.017403	0.016007	0.872235	0.761923	44.08606	57.86156
77.3723	0.000112	38.53191	0.014368	0.007458	0.866629	0.770245	58.07568	75.39893
62.3701	0.00039	20.41658	0.030409	0.011594	0.933257	0.85069	42.34635	49.77884
21.08617	0.000373	44.10013	0.017287	0.026029	0.787244	0.762284	43.0525	56.47831
3.749048	0.004678	35.81166	0.047427	0.159861	0.913377	0.84497	15.76407	18.65637
11.35559	0.00062	63.65538	0.030471	0.042967	0.911959	0.814478	38.78259	47.61652
14.01743	0.000538	43.36712	0.02076	0.038654	0.720419	0.636428	31.48188	49.46654
7.052481	0.000958	87.83049	0.026947	0.085933	0.919988	0.871178	34.52891	39.63475
13.13819	0.000799	46.23731	0.044951	0.038649	0.939108	0.83501	33.30728	39.88849
0.497306	0.027528	40.15825	0.064111	1.038124	0.828035	0.720515	8.818098	12.2386
23.09934	0.000504	18.68259	0.003444	0.040366	0.795492	0.570675	23.59132	41.3393
3.090455	0.006421	13.17567	0.023006	0.168258	0.763988	0.73158	12.70033	17.36015
61.39558	0.000141	23.41912	0.003025	0.013493	0.607896	0.555499	38.6232	69.52883
111.6137	6.84E-05	41.40104	0.011067	0.005507	0.814786	0.745896	63.46176	85.08123
5.167651	0.000382	71.26797	0.01714	1.107427	0.879433	0.81699	33.00447	40.39765
46.72961	0.000265	22.24	0.01478	0.013965	0.666371	0.61281	43.27665	70.62002
11.79905	0.001113	11.4283	0.003706	0.0493	0.966078	0.803788	27.06257	33.6688
7.484414	0.0014	16.27029	0.005466	0.081806	0.905361	0.853921	28.60227	33.49522
27.0878	0.000662	11.95873	0.007552	0.022151	0.967481	0.853066	26.30284	30.83332
22.08826	0.00031	32.88387	0.007934	0.026909	0.826274	0.738479	40.75235	55.18419
138.4964	4.41E-05	43.77783	0.003477	0.006084	0.872737	0.833656	79.84157	95.77284
112.0219	0.000112	19.29989	0.00598	0.006237	0.838104	0.769673	33.13254	43.04755
6.414537	0.001389	29.40885	0.008461	0.092473	0.6936	0.577491	21.17712	36.67087
50.63563	0.000644	9.304177	0.009097	0.014428	0.92844	0.843179	29.46408	34.94404
5.185648	0.002357	23.58608	0.013708	0.102609	0.59199	0.54225	19.90851	36.71464
6.755929	7.11E-05	373.9597	0.000545	0.426922	0.807624	0.752336	75.84019	100.8063
3.238224	0.00291	27.58297	0.012134	0.204381	0.596975	0.482251	18.50067	38.36318
67.312	9.59E-05	41.71663	0.005377	0.011013	0.905072	0.723694	51.88482	71.69442
5.18208	0.007517	4.719195	0.007463	0.110943	0.751316	0.559743	11.93908	21.32958
14.8548	0.001262	13.84135	0.012913	0.034447	0.593032	0.399983	20.94256	52.35859
20.97732	0.000642	19.91573	0.020971	0.026176	0.894896	0.625062	26.39331	42.22512

3.286665	0.000173	208.6648	0.000733	0.68451	0.58227	0.466363	53.82283	115.4097
65.29681	5.02E-05	157.2647	0.00509	0.02432	0.882838	0.654305	69.46235	106.1621
205.4729	4.97E-05	16.94966	0.003509	0.003088	0.597555	0.526239	62.80892	119.3543
14.33338	7.31E-05	1801.036	0.001277	3.118173	0.920496	0.801899	59.87992	74.67263
24.17051	0.00021	50.45974	0.003764	0.033054	0.896336	0.866179	45.98582	53.09046
34.24554	0.000457	13.58224	0.009097	0.017262	0.774382	0.740012	32.61584	44.07472
20.45905	0.000634	16.89077	0.008457	0.026508	0.850589	0.751064	28.50093	37.94738
58.58491	0.000209	18.82498	0.005865	0.011846	0.89624	0.838251	48.10691	57.38965
19.99179	0.00128	14.65327	0.013627	0.041595	0.813822	0.689072	20.49328	29.74043
16.78946	0.000919	18.71449	0.031459	0.032585	0.759624	0.6598	22.71528	34.42755
23.47028	0.000602	17.92395	0.012746	0.022984	0.926499	0.819578	24.13054	29.44264
48.33077	0.000328	23.75776	0.007772	0.019731	0.889856	0.835621	29.75069	35.60309
12.97746	0.002923	6.369621	0.022325	0.034244	0.802188	0.718773	12.60358	17.53486
39.65122	0.001575	4.209185	0.020021	0.01231	0.726852	0.643957	22.88051	35.5311
72.10164	0.000242	11.21459	0.006355	0.007084	0.884754	0.801269	42.81239	53.43073
6.921899	0.00299	12.3577	0.008394	0.099253	0.667019	0.602805	18.31035	30.37523
41.78117	0.000711	6.89744	0.004719	0.020605	0.780309	0.64462	23.29838	36.14282
57.48284	0.000101	64.82827	0.012084	0.012549	0.937189	0.883127	70.38565	79.70052
48.82606	0.000127	45.03576	0.008919	0.012307	0.852648	0.680048	37.32047	54.87917
46.01081	0.000266	25.50241	0.013462	0.011462	0.902565	0.809117	40.90819	50.55903
38.23782	0.000233	25.59996	0.004455	0.019019	0.563797	0.529591	40.26168	76.02413
95.0009	7.59E-05	55.89667	0.011738	0.009496	0.723895	0.6181	59.7991	96.74669
59.61575	0.000134	30.34392	0.003	0.015528	0.635343	0.617397	49.0587	79.4606
1.480448	0.050002	5.857337	0.035657	0.401702	0.764654	0.537862	10.07326	18.72833
10.49954	0.000877	20.48899	0.004797	0.052885	0.878664	0.718369	30.53876	42.51126
59.50817	0.000123	31.97786	0.007325	0.010398	0.717868	0.553698	50.99278	92.09494
48.60128	0.000101	54.20948	0.006374	0.014069	0.910984	0.815278	64.38641	78.9748
15.34998	0.000282	53.92321	0.010786	0.049707	0.828994	0.700265	40.06349	57.21189
1.977863	0.005012	28.28718	0.012097	0.313395	0.853232	0.782902	17.87611	22.83314
2.163623	0.007072	17.12011	0.012928	0.260723	0.775317	0.675908	14.01876	20.74063
18.57266	0.000315	35.32536	0.008594	0.043997	0.638003	0.607127	32.60103	53.69718
4.087263	0.001547	42.78201	0.015009	0.160153	0.904827	0.821091	23.86155	29.06078

DX5	DX6	DX7	DX8	DX9	DX10	DX11	DX12	DX13
40.29192	43.53771	40.50881	43.90966	25947.99	32.60293	0.756627	5601.933	0.215891
46.45579	45.02584	35.44252	47.04398	25753.68	30.95024	0.779239	5412.19	0.210152
57.83366	55.26987	51.2507	60.79015	60550.45	43.56261	0.788193	9460.846	0.156247
16.96237	17.93847	16.35745	18.9486	1567.16	13.57814	0.829855	786.2443	0.5017
33.21921	33.21921	30.48706	37.67507	11406.22	24.75314	0.790041	3101.671	0.271928
47.67341	50.89352	51.12797	52.81926	41095.8	38.39086	0.759712	7580.536	0.18446
48.39983	42.6157	288.3508	291.6411	23163.83	37.80469	0.744585	5277.688	0.227842
46.30554	52.25953	51.07803	53.61471	39823.22	38.98642	0.756857	7451.231	0.187108
21.57808	20.58772	20.06688	22.40322	2994.514	16.39189	0.798611	1258.055	0.42012
72.7157	68.68506	373.3103	373.8981	98416.1	57.12767	0.666046	15477.14	0.157262
121.1829	109.419	108.5958	134.3183	438540.4	80.72332	0.712595	39172.49	0.089325
148.9066	144.4243	112.3184	149.9868	777744.1	98.29385	0.703992	58095.31	0.074697
60.04208	54.7031	59.6173	60.89363	69059.74	44.13974	0.769144	10583.42	0.15325
32.81101	32.81101	28.24281	34.12408	11101.48	25.2438	0.801938	3000.987	0.270323
24.32104	23.29598	23.25587	25.38199	4438.596	19.41305	0.806836	1618.81	0.364712
85.52261	94.26813	88.94417	97.55336	226258.6	63.80281	0.726866	24703.96	0.109185
29.48874	28.152	25.21051	29.90182	6785.83	21.84594	0.805541	2151.783	0.317099
63.56898	67.37275	69.4255	72.29769	90107.44	49.82024	0.721443	13472.74	0.149519
60.71956	60.71956	57.68141	64.18599	67417.08	45.82526	0.714312	11214.4	0.166344
96.79807	90.62184	86.47576	97.94542	290460.5	73.45532	0.758647	27957.77	0.096253
101.9668	106.0515	102.9962	110.2239	369520.5	76.04714	0.748521	33268.97	0.090033
72.35654	68.90414	70.88853	77.83153	117577.6	55.16292	0.722955	16054.21	0.136541
30.09669	30.09669	28.46266	31.60245	8570.394	22.83828	0.791821	2557.746	0.29844
81.99991	71.55121	80.95735	84.15829	151396.2	58.20958	0.750918	18293.7	0.120833
68.55798	59.51907	61.11403	70.6216	81828.37	48.12026	0.742409	12277.51	0.15004
31.25614	32.31543	30.28555	34.22797	9629.819	25.22689	0.772975	2831.809	0.294067
49.13557	48.7201	101.5993	104.8956	35490.48	39.99149	0.743471	7024.701	0.197932
54.38304	55.50027	49.19104	57.31404	45271.64	39.34132	0.737994	8323.685	0.183861
42.57375	41.65059	42.17712	44.38755	24015.53	30.91913	0.746183	5394.698	0.224634
49.83875	51.09405	42.98412	51.21087	39849.03	38.19142	0.786933	7169.543	0.179918
26.97005	35.36889	35.5057	36.51399	8606.968	21.14319	0.745251	2725.305	0.316639
76.02209	77.56663	74.44408	80.85491	129660.5	59.01829	0.685967	18060.06	0.139287
36.2278	36.57376	36.19391	37.97135	14183.83	27.69651	0.778891	3638.058	0.256493
73.11076	80.20251	72.86	86.85189	112929.8	50.07119	0.666503	16952.01	0.150111
135.4533	154.6157	144.2584	154.7974	955252.8	105.6399	0.663273	70719.24	0.074032
38.75943	42.91791	39.25562	44.56209	17712.57	29.77042	0.731834	4490.136	0.2535
104.9383	102.0189	110.1402	114.8794	342067.1	72.52819	0.71368	33142.7	0.096889
30.76915	28.80121	28.01397	31.37592	7632.777	21.44716	0.793766	2361.819	0.309431
37.7058	40.13822	39.21049	44.14141	19627.53	28.67204	0.765757	4595.19	0.23412
30.73972	26.15795	28.70755	31.50174	7106.489	21.39076	0.764572	2337.953	0.328989
52.85605	69.90333	55.51596	71.66135	46094.78	38.33393	0.653614	9511.832	0.206354
123.0775	110.5519	103.2599	125.1556	508140.1	89.19367	0.73472	41913.25	0.082484
41.63366	44.0898	37.19185	44.24114	25688.15	32.37955	0.800849	5257.208	0.204655
91.01479	87.8281	80.27985	93.4277	192151.8	62.06097	0.678323	23739.81	0.123547
91.0471	87.1772	80.24261	99.34202	165619.3	67.01928	0.654125	22296.18	0.134623
31.80202	33.08063	30.28956	35.23708	10004.78	24.84419	0.742509	3024.04	0.302259
38.3805	40.15716	32.23473	40.68553	14843.21	27.52241	0.771095	3787.87	0.255192
62.28287	63.22018	67.07641	71.82261	84134.99	48.69671	0.743727	12485	0.148392
69.07658	71.33589	56.57265	71.86246	81508.67	47.39806	0.743865	12221.54	0.149942
62.66602	74.28955	79.06505	79.06505	66695.33	48.07599	0.650343	12229.4	0.183362
83.46681	77.57536	73.8402	84.32307	140265.6	59.12447	0.692232	18859.64	0.134457
30.79215	30.39369	29.08019	31.51588	10098.85	24.17186	0.790803	2857.132	0.282917
40.92215	41.32551	35.77639	374.965	22820.79	34.44089	0.784073	4962.281	0.217446
57.97461	55.13306	43.16254	58.69534	33700.93	34.10207	0.664616	7591.735	0.225268
91.72761	100.0327	82.08288	102.0324	209840.9	62.0201	0.71832	23773.47	0.113293
56.29923	56.98093	51.02428	57.61714	62513.6	44.43566	0.761563	10002.18	0.16
47.77248	47.11925	38.99346	48.80777	31575.02	34.49461	0.806604	5989.442	0.189689

35.98956	37.69661	35.90685	40.84443	14548.59	28.24163	0.707376	4074.252	0.280045
33.77553	30.76643	31.50802	34.19565	11400.79	24.41936	0.794662	3082.658	0.27039
49.45271	49.21261	44.36518	49.92256	28605.72	35.66872	0.692502	6531.783	0.228338
144.9141	151.5208	136.9276	151.5977	960118.2	110.6867	0.728246	64628.31	0.067313
32.00343	29.22534	30.26134	32.64421	10417.65	23.84306	0.781724	2950.828	0.283253
82.83092	79.61716	70.1224	84.17196	112811.3	59.19967	0.695412	16235.93	0.143921
91.61077	93.4924	100.9087	103.5446	216844.9	69.90181	0.659627	26461.76	0.122031
107.7169	102.4456	94.90337	107.7824	258378.7	71.22811	0.681548	28784.52	0.111404
38.75778	37.49648	34.49533	42.14066	14079.28	26.54768	0.741592	3802.24	0.270059
26.42672	32.73127	29.89786	33.18951	7304.66	19.92312	0.799277	2277.825	0.311832
32.79498	34.17339	31.73184	35.46143	8937.105	24.77318	0.745226	2794.648	0.312702
82.8955	75.18358	80.73624	88.45749	168379.2	64.89066	0.724719	20347.31	0.120842
134.9281	139.4564	135.617	145.0472	834818.5	110.7008	0.706067	60724.83	0.07274
88.76485	97.27146	93.34173	101.4124	277332.7	73.44693	0.73072	28144.95	0.101484
123.3869	122.9258	114.3646	126.1247	514957.4	97.38363	0.697489	44544.55	0.086501
82.44811	109.5093	355.6831	355.856	273440.1	69.4343	0.716566	28431.68	0.103978
20.87184	21.24776	22.25571	22.88705	2816.57	16.75483	0.746314	1292.339	0.458834
56.6636	57.31956	42.18942	57.46612	41843.93	34.51155	0.774462	7526.14	0.179862
379.0685	294.0032	371.1266	420.5402	4092.383	236.559	0.027584	44855.66	10.96077
48.38683	47.29454	43.65169	49.80597	28225.69	33.36529	0.715415	6266.462	0.222013
84.06356	95.82583	77.00373	95.86484	169373.4	62.82638	0.682393	21694.33	0.128086
52.61417	48.84191	49.63945	54.34859	44051.14	38.66239	0.776366	7769.43	0.176373
46.24743	51.20014	46.78998	51.67178	35715.85	36.50566	0.61626	8510.612	0.238287
74.17604	71.11073	67.04002	74.99553	111418.6	53.43077	0.728291	15375.09	0.137994
28.84316	28.1998	30.32262	30.93957	9153.485	24.23014	0.752476	2812.22	0.307229
95.41676	94.83185	81.54222	100.5025	232459.8	70.76485	0.722546	25303.68	0.108852
143.3852	152.0406	109.229	156.5631	668465.2	87.90055	0.678755	54469.96	0.081485
68.34667	64.94157	64.9931	68.91675	93329.62	50.00819	0.76442	13016.63	0.139469
52.80065	54.51207	39.60523	54.90333	27373.39	31.53708	0.613863	7155.362	0.261398
56.46115	57.36642	49.97711	58.10212	53863.04	42.39131	0.766294	9000.826	0.167106
26.50347	27.97799	26.96676	28.43406	5823.12	20.68629	0.760253	2058.874	0.353569
65.56773	60.75326	66.36495	70.2987	87261.83	48.94001	0.710964	13381.96	0.153354
109.0027	109.2178	84.21995	109.9486	319465.1	71.57014	0.772103	29270.12	0.091622
59.70206	59.37184	58.71494	62.96797	72155.27	49.84418	0.734404	11412.87	0.158171
46.82987	40.86498	42.08851	47.05002	25585.69	33.03517	0.763684	5498.387	0.214901
65.19892	63.63246	58.45211	65.60651	67978.1	44.01183	0.736491	10936.94	0.160889
34.53125	28.59375	33.474	35.00671	9919.589	23.3692	0.789528	2827.782	0.28507
72.58674	68.17913	60.91866	72.78707	93164.55	53.29325	0.723469	13737.2	0.147451
25.99254	23.431	20.13235	27.06207	3258.536	15.47101	0.728159	1459.731	0.447971
25.77732	22.81601	22.08759	26.44938	4051.841	18.96965	0.777302	1581.234	0.390251
38.76215	32.93784	39.49435	39.49435	14876.49	25.26258	0.762199	3837.801	0.257978
49.64343	56.00061	46.31877	56.06733	41646.55	37.29417	0.760949	7635.682	0.183345
28.7205	29.22736	28.73905	30.76083	6900.787	22.34676	0.694698	2523.214	0.365642
63.02738	60.97266	61.22202	63.73433	85313.18	48.12756	0.7551	12411.48	0.145481
54.55235	55.00554	58.93343	65.16488	59375.94	43.67698	0.757266	9719.475	0.163694
38.77128	37.29027	32.35715	38.81811	13775.28	26.56061	0.754604	3682.693	0.267341
44.2086	49.77135	45.8397	50.26283	27044.82	34.6628	0.667806	6524.638	0.241253
53.26143	57.77344	58.55183	59.13628	57601.94	42.27433	0.750131	9615.5	0.16693
30.28304	32.33528	26.43041	34.07993	8149.991	21.3083	0.769731	2544.386	0.312195
39.8459	42.21975	39.23906	42.80401	24399.16	33.08366	0.797412	5101.738	0.209095
68.0387	72.24492	82.99903	84.93691	108677.9	52.86404	0.681688	16155.71	0.148657
102.405	88.88506	87.5949	102.8474	247151	65.99972	0.659083	28896.96	0.11692
94.8841	89.43748	86.06021	95.08678	238382.4	71.8472	0.758019	24527.51	0.102891
36.6451	37.30737	34.56721	37.95324	16289.65	28.47286	0.747466	4157.514	0.255224
43.54656	37.67257	44.69865	46.49409	19660.84	30.43537	0.73797	4773.61	0.242798
119.2277	111.6388	113.5518	366.8879	524985.4	93.36444	0.725272	43392.51	0.082655
46.10196	44.71227	46.11947	51.10007	29418.21	33.15179	0.706251	6525.328	0.221813
66.71353	70.92792	51.04574	72.56491	59258.46	44.89126	0.681395	10787.46	0.182041

34.79371	35.58189	34.8353	36.52437	15063.8	27.33191	0.689456	4278.262	0.284009
34.89626	35.39815	31.52563	37.95787	13640.7	26.84138	0.782328	3529.009	0.258712
113.6852	114.7896	87.23524	119.9496	309141.4	69.04321	0.632884	34935.34	0.113008
31.64411	32.68234	28.98154	32.99105	9457.835	23.93705	0.77291	2798.229	0.295864
28.64583	33.85417	36.04326	36.04326	6792.281	27.26925	0.645133	2688.511	0.395819
86.21011	84.3844	65.59177	86.57332	136055.5	54.85697	0.675881	18927.41	0.139115
32.30316	29.64745	28.78457	33.42854	8518.948	22.74631	0.781182	2582.197	0.303112
61.99818	63.57773	63.13535	69.91878	87414.97	48.66445	0.755638	12605.53	0.144203
35.586	35.22303	30.84516	36.42956	11504.74	25.50838	0.772549	3190.135	0.277289
39.42347	38.23076	38.35984	40.67839	21776.24	30.83929	0.777493	4850.388	0.222738
54.89816	55.05243	45.39484	55.53322	43684.72	40.54832	0.76062	7886.227	0.180526
50.99805	44.57798	49.71285	51.24429	33830.91	34.0767	0.766145	6602.607	0.195165
57.10132	54.74031	55.11309	60.18946	58252.77	42.59149	0.758076	9586.26	0.164563
62.04287	69.4238	61.69873	72.74579	85628.22	49.37738	0.684029	13734.76	0.1604
122.0795	93.87671	112.5823	122.7228	220842.9	60.84868	0.655169	26968.28	0.122115
50.77882	54.29473	39.57751	55.17986	30073.39	33.40838	0.78567	5952.49	0.197932
72.44653	75.81072	73.35474	76.89435	151168.1	60.86551	0.754891	18179.14	0.120258
121.8282	149.6779	133.3091	154.9396	666922.1	94.20886	0.662629	55709.66	0.083532
27.99784	25.69267	24.62722	28.61484	5722.901	21.02278	0.796246	1943.185	0.339546
36.88856	42.1264	39.95727	43.69291	18812.06	27.09417	0.775045	4413.482	0.234609
30.94031	32.04818	26.54989	32.09999	8756.648	22.94181	0.770884	2665.142	0.304356
54.22888	48.98297	47.62758	54.26518	35754.41	35.91231	0.732923	7161.086	0.200285
38.80234	34.86969	37.01239	39.99775	17155.9	29.15192	0.760245	4231.293	0.246638
39.57632	39.57632	41.22557	41.32167	21473.03	30.02321	0.797994	4681.807	0.218032
87.99464	83.05818	70.96802	91.85548	162051.8	60.96028	0.739325	19442.46	0.119977
45.81673	46.48117	45.18548	48.23522	31499.16	36.20701	0.757876	6364.319	0.202047
50.44643	48.35169	46.03949	51.17056	41260.82	37.70141	0.781867	7385.443	0.178994
79.43682	77.44619	379.754	386.1244	110434	54.48704	0.651194	17093.93	0.154789
37.08239	37.92605	27.34966	38.4182	11372.55	23.91542	0.791413	3090.196	0.271724
53.6384	52.87442	38.98993	53.90209	22665.67	31.15173	0.726198	5333.45	0.23531
27.87623	27.44125	23.65532	32.52676	4846.263	20.89623	0.763898	1812.953	0.374093
42.95391	46.75808	34.535	46.79684	21711.89	29.81285	0.763327	4930.663	0.227095
89.15128	90.89985	67.81084	92.83362	154440.5	57.78684	0.743947	18711.83	0.121159
86.74915	106.5066	73.33092	108.6391	201154.3	62.94116	0.745378	22273.78	0.11073
44.89741	43.41389	29.63987	45.41744	14036.3	24.13361	0.73496	3828.739	0.272774
30.86285	25.75997	23.34004	32.30478	4764.176	18.13661	0.743371	1841.917	0.386618
52.55408	53.60709	41.71812	57.31047	33760.97	36.06396	0.742899	6799.826	0.201411
67.93118	65.84009	59.73678	70.98634	79163.24	48.30194	0.722061	12347.89	0.15598
68.26109	83.47962	80.55002	84.14324	130851.9	57.27101	0.720377	17302.59	0.13223
63.24836	56.04622	50.8382	64.5115	54381.23	41.44695	0.737531	9411.737	0.17307
57.98117	60.72981	52.33442	61.60675	55098.56	44.88904	0.759264	9222.555	0.167383
48.69609	47.69096	38.75404	50.32932	13764.06	22.85162	0.732379	3792.387	0.275528
81.87072	86.00323	66.5499	87.63563	113664.3	53.19685	0.588414	19284.91	0.169665
26.53307	28.55242	19.44748	28.6906	4581.711	18.11684	0.796177	1675.558	0.365706
66.40969	64.27268	42.31362	67.08428	51855.89	38.27389	0.762257	8822.294	0.170131
53.60187	54.04731	123.5848	124.4918	28326.29	39.70238	0.683004	6579.418	0.232272
39.98598	41.46015	40.69195	42.39675	22436.97	31.71278	0.769232	5001.145	0.222897
92.49977	103.7718	107.9726	109.0096	220604.4	68.80847	0.710284	24857.72	0.11268
64.41305	55.79719	44.36448	64.90785	35804.27	35.99282	0.688077	7634.905	0.21324
80.6032	85.06487	76.70731	91.19717	156884.4	65.05892	0.686608	20487.79	0.130592
54.60887	63.63961	52.33442	63.70173	43463.22	42.3807	0.741505	8062.16	0.185494
61.7023	56.87049	86.89237	126.7649	37926.9	35.5251	0.752869	7250.969	0.191183
26.16721	25.81532	17.46526	26.83875	3109.371	14.73173	0.758011	1359.118	0.437104
63.34202	66.92046	50.36399	68.19713	35112.1	34.0982	0.598013	8671.183	0.246957
64.23485	60.5161	66.93697	67.35782	70668.3	48.56837	0.70977	11646.17	0.1648
29.29837	29.84213	90.38743	93.59256	6437.826	23.43429	0.733451	2281.772	0.354432
206.4294	124.8289	193.6617	231.481	427498	96.4835	0.588494	46633.48	0.109085
47.80415	50.69747	40.80944	51.69568	29395.88	34.37848	0.728329	6324.323	0.215143

78.47269	79.18831	82.69077	85.08124	152873.8	61.6068	0.711263	19439.09	0.127158
78.54832	77.50782	47.09522	78.55086	42655.58	37.18925	0.629217	9382.842	0.219968
52.77017	54.16466	43.0664	54.84523	40156.43	36.39888	0.781667	7254.915	0.180666
49.06505	51.40245	45.57313	52.87222	32836.88	35.10746	0.772616	6418.42	0.195464
87.39171	90.23894	66.82277	90.85592	127524.5	54.54734	0.681139	17987.78	0.141053
44.67046	47.36935	45.59343	48.89944	35722.16	36.51026	0.7685	6825.465	0.191071
61.39517	62.99658	64.86766	68.20214	70619.26	46.66178	0.716228	11535.82	0.163352
65.538	64.9625	44.89335	66.45245	51235.07	38.36262	0.721137	9250.766	0.180555
199.6822	202.1765	160.6634	202.4156	1840172	137.1825	0.680499	106715.8	0.057992
67.79274	59.89416	59.86894	71.46708	66614.18	45.2234	0.698754	11372.89	0.170728
41.21701	48.16389	39.41701	48.79465	18515.43	30.72812	0.718488	4710.718	0.254421
127.7768	133.9474	118.8973	150.3971	734112.6	101.1023	0.702511	56019.51	0.076309
93.38261	91.6856	70.18391	96.90524	157946.3	55.75362	0.674971	20934.95	0.132545
95.07074	100.0688	97.427	108.6016	335425.5	75.45883	0.754478	30943.43	0.092251
26.45947	23.10823	23.94757	28.2575	4037.27	17.88247	0.787128	1557.748	0.385842
54.32497	59.07797	55.7384	59.94262	58217.64	41.71324	0.742649	9781.459	0.168015
99.3198	103.8644	86.37102	105.7844	275120.6	71.20884	0.725354	28202.2	0.102509
76.72542	87.39204	80.86729	89.82259	139576.5	58.68307	0.661925	19658.5	0.140844
44.60398	39.53304	37.02763	45.08986	17143.65	29.50794	0.736621	4364.915	0.254608
47.36057	53.06614	41.20336	53.18247	27149.14	31.07151	0.705173	6194.782	0.228176
74.52228	76.75458	65.33734	81.59702	106445.8	52.98356	0.710816	15280.79	0.143555
20.08663	21.54721	17.10777	21.83213	2274.788	15.06189	0.825396	1013.404	0.445494
92.83762	94.64264	91.16848	98.55187	269835.8	75.18998	0.695002	29055.67	0.107679
85.8619	81.95789	74.60087	86.20955	138299.5	58.26978	0.69519	18603.49	0.134516
26.82692	24.75071	18.2301	27.49312	2931.023	13.8708	0.764369	1295.767	0.442087
74.45283	70.79629	59.41621	75.67382	82528.11	50.46887	0.728089	12590.24	0.152557
89.56446	83.70113	78.12928	96.79524	203706	65.34291	0.753621	22216.05	0.109059
58.32508	58.72718	56.75507	63.14298	70376.97	46.45643	0.764785	10778.66	0.153156
61.68294	65.14824	64.63398	75.43271	71923.87	44.46224	0.716581	11671.7	0.162278
22.8331	23.33635	20.7801	23.79214	3461.715	17.04031	0.813203	1360.857	0.393116
53.80753	55.37211	54.60894	56.5509	57103.96	43.42432	0.772208	9286.679	0.162628
57.20248	55.34141	43.17333	57.60257	35331.67	35.63666	0.667217	7804.166	0.220883
47.84415	46.50689	43.75	49.23223	35899.2	36.4635	0.807617	6516.311	0.181517
45.16474	47.10938	47.75919	48.90663	35247.08	37.45962	0.76373	6807.059	0.193124
14.71927	13.62744	12.46371	15.7733	802.636	10.13398	0.825712	505.8263	0.630206
50.44648	38.50742	46.13576	53.70176	20423.58	32.8851	0.693792	5208.064	0.255002
18.61494	22.56631	17.6989	23.28165	2000.129	13.26295	0.774832	990.7911	0.495364
70.24388	73.23166	49.24731	237.7243	75326.44	42.26628	0.714326	12074.97	0.160302
99.62501	93.01693	87.22305	102.6363	258339.8	69.32302	0.702047	27941.24	0.108157
43.80866	46.8883	44.5534	49.60363	34218.08	35.52701	0.799143	6378.179	0.186398
75.58878	64.50156	75.02873	82.94768	99590.09	47.05913	0.713718	14558	0.146179
40.74449	41.1048	38.01395	42.44723	21113.48	32.52668	0.787557	4690.749	0.222168
39.21052	36.81681	41.02906	43.81463	20080.89	30.32527	0.757094	4719.071	0.235003
38.27384	37.80742	34.69939	39.04944	17226.42	29.83063	0.768014	4199.957	0.243809
68.97917	58.47294	53.68187	69.85175	73257.08	45.59727	0.759212	11152.04	0.152232
108.6633	108.5292	112.3508	118.6109	442748.3	83.58454	0.668099	42048.23	0.094971
52.82735	52.58765	42.35792	56.90424	36208.16	36.07831	0.722197	7328.8	0.202407
40.87215	31.99123	38.91625	42.2298	13851.35	25.4349	0.756732	3685.841	0.2661
41.5072	40.15716	40.20837	42.38403	23896.56	32.44345	0.773953	5183.938	0.216932
28.16025	38.28125	41.1294	42.06738	10364.72	21.73471	0.694467	3310.33	0.319384
116.0116	118.2108	101.9119	120.2035	417411.1	81.41355	0.687887	39265.36	0.094069
40.05002	43.37458	28.63023	45.40165	10167.64	22.90184	0.627272	3618.332	0.355867
81.73903	84.13182	95.9767	97.47392	152096.6	64.88864	0.662996	20783.54	0.136647
26.85462	24.98352	17.75268	28.0625	2651.403	16.02527	0.712457	1300.297	0.490418
45.91198	59.48643	46.13652	59.83646	14865.84	31.05031	0.54216	5392.823	0.362766
50.00104	50.95595	43.2579	53.26143	29716.54	37.78709	0.7144	6494.436	0.218546

120.7418	129.4493	77.69983	130.5582	291612	67.19963	0.699256	30412.44	0.104291
122.3012	133.7158	110.3448	134.4034	464462.1	93.72389	0.600076	48333.1	0.104063
126.8092	126.5291	88.58585	137.11	361055	71.32071	0.659307	37191.64	0.103008
85.57152	86.48535	91.4816	93.95611	207601.7	68.73583	0.670466	25288.8	0.121814
62.09794	59.80519	59.25618	64.57121	82406.38	47.58691	0.774992	11816.65	0.143395
50.61329	48.04974	47.44152	51.63118	34124.27	34.13066	0.733796	6933.478	0.203183
43.35173	39.59855	42.3358	45.33857	24912.18	32.27763	0.755077	5463.034	0.219292
66.69668	62.89457	64.76511	251.3958	99995.72	51.43489	0.775141	13440.78	0.134414
35.36501	33.7318	27.38995	37.48863	10086.02	24.20342	0.737386	3061.511	0.30354
38.48472	38.03144	34.19967	40.19121	14029.05	26.15198	0.751808	3741.647	0.266707
35.72552	35.72552	33.5387	36.86989	13887.39	27.27859	0.769739	3629.84	0.261377
44.09912	44.09912	40.63251	45.14253	23626.8	31.68161	0.742695	5361.382	0.226919
20.23803	20.23803	18.25997	23.0066	2210.652	14.06624	0.767918	1068.684	0.483425
42.72576	35.70414	32.84725	45.60492	12780.07	25.82585	0.67636	3908.366	0.305817
61.59177	65.40257	54.85864	65.72362	75947.45	47.27306	0.74052	11711.78	0.154209
35.16788	31.66253	22.58706	35.97839	7588.225	20.26084	0.751011	2486.554	0.327686
43.692	42.80288	33.35215	43.79011	16539.07	28.20257	0.738498	4250.847	0.257018
92.56717	92.85414	90.47404	95.58253	297202.4	74.69446	0.746816	28838.47	0.097033
65.79944	59.91395	56.13105	66.08887	67461.89	46.79261	0.727584	11014.72	0.163273
59.08007	56.09802	54.73021	62.40354	67559.86	45.6328	0.781809	10260.68	0.151875
83.53489	67.5092	61.74703	85.52769	85985.03	42.8622	0.696518	13525.92	0.157306
111.2784	112.8578	90.3645	113.6292	282345.7	70.03445	0.656139	31720.67	0.112347
85.68183	92.47043	64.7507	95.18347	136301.1	50.48476	0.700185	18292.4	0.134206
20.80511	18.0625	20.74438	21.521	1775.979	14.32069	0.742093	955.6921	0.538121
46.89846	50.18772	47.50386	52.22877	32842.35	37.3531	0.699569	7089.402	0.215862
101.5998	103.1073	83.61734	109.2367	203717.3	66.11205	0.640683	26133.23	0.128282
87.54376	95.63534	85.781	97.56358	259702.6	71.94479	0.718192	27409.09	0.10554
60.66657	63.14441	66.55595	71.39793	70797.98	47.42832	0.641795	12895.42	0.182144
26.44971	28.13002	24.79515	29.31265	4999.351	19.48197	0.727966	1942.297	0.38851
22.33092	20.63225	23.16455	24.56086	3129.085	16.08057	0.754764	1370.728	0.43806
48.88228	57.68398	59.15192	61.50008	42126.58	34.25899	0.699904	8365.332	0.198576
36.05249	35.08021	33.84997	39.50058	11957.24	26.29498	0.690722	3661.014	0.306175

DX14	DX15	DX16	DX17	DX18	DX19	DX20	DX21	DX22
26014.86	26	70	58006312	1.661217	23	4.642377	129	14.0022
25807.77	10	53	31647420	1.552223	23	3.240964	92	13.37431
60661.88	62	89	1.95E+08	1.277674	13	25.1771	392	8.859194
1600.429	74	104	7244509	1.211298	16	4.488109	148	10.02026
11481.57	35	89	24108738	1.996637	25	7.662494	140	18.59901
41210.38	6	52	21851662	1.746791	22	4.399342	116	14.53602
23283.13	20	76	37390887	1.984313	29	345.2214	128	19.63125
39942.32	20	78	44182098	1.979447	29	3.466612	138	18.05372
3042.467	33	94	5478671	2.028129	32	3.095287	133	18.85762
98594.97	53	99	2.56E+08	1.832574	22	650.9084	365	15.79033
438908.2	19	71	4.45E+08	1.803612	29	3.151137	187	16.3032
778116.8	48	97	2.5E+09	1.776791	23	3.452418	206	14.90012
69190.69	42	95	1.87E+08	1.883278	26	4.255741	189	16.51118
11167.97	61	116	46809633	1.938437	28	3.704129	173	17.21482
4493.163	60	108.9	14664795	1.891632	23	4.882404	169	15.71095
226470.9	40	80	3.99E+08	1.542421	20	4.121401	139	12.55096
6840.615	43	83	15328045	1.46776	20	3.765443	120	12.11553
90259.58	17	81	1.16E+08	2.094167	40	9.220044	387	21.60284
67570.79	54	110	2.28E+08	1.922915	28	5.09705	143	17.91089
290694.2	21	88	4.91E+08	1.967512	31	3.159872	194	19.95769
369828.8	19	85	5.67E+08	2.07097	44	1.983018	163	22.10327
117823.2	18	87	2.04E+08	2.242651	35	10.1308	529	22.03235
8646.265	26	74	12166327	1.796231	23	5.067374	117	15.21157
151605.7	28	83	3.74E+08	1.883012	31	7.671157	368	17.37717
81964.55	38	82	1.83E+08	1.661484	20	7.742038	191	13.76592
9723.964	54	84	19537324	1.361877	15	12.39974	198	9.92524
35601.21	51	76	1.02E+08	0.962096	13	1162.24	245	8.56345
45377.53	34	102	1.6E+08	2.150867	36	2.932982	260	21.42258
24136.99	47	96	59612395	1.831953	25	4.678335	179	15.73645
39957.19	20	87	65073248	2.147151	37	3.832889	137	21.36101
8693.29	57	91	17178989	1.414504	17	3.691471	134	10.88063
129899.8	30	83	2.5E+08	1.818137	26	3.200173	148	15.91616
14283.62	39	80	17927095	1.631449	20	5.545759	157	13.26897
113127.4	23	86	2.41E+08	2.016505	42	2.764455	139	21.13304
955656.8	23	70	1.06E+09	1.665701	28	339.626	1320	15.18989
17805.81	65	89	49774135	1.139888	12	8.725141	159	8.072709
342299.8	23	96	8.04E+08	2.224221	44	3.114788	437	23.24454
7706.424	11	49	2886848	1.573135	19	4.619725	108	12.31698
19724.71	57	88	41291482	1.358066	16	12.05418	224	10.10956
7171.136	40	102	20113137	2.140428	24	5.864856	185	19.30576
46247.76	44	95	1.21E+08	1.929889	25	7.272933	181	17.02361
508445	26	107	1.64E+09	2.179456	38	4.17273	462	24.08609
25770.85	19	101	65080351	2.402988	45	2.783192	170	25.59551
192379.2	45	93	5.95E+08	1.797487	25	33.03679	622	15.37432
165849.4	40	79	2.55E+08	1.488313	19	16.38371	372	12.16205
10082	73	107	49375088	1.336935	18	3.070537	136	10.78332
14905.9	43	79	37645524	1.391362	18	3.294813	120	11.22933
84273.87	21	95	2.39E+08	2.143059	38	2.618237	244	22.65366
81678.12	41	97	2.1E+08	1.882752	26	3.687827	282	16.74051
66867.11	41	95	1.65E+08	1.894364	28	15.44695	396	17.29608
140466.3	49	84	3.3E+08	1.444482	17	6.715743	143	11.35422
10170.36	62	94	28008797	1.36188	15	66.16091	394	10.77894
22924.84	41	83	39920050	1.666842	21	800.0338	137	14.25044
33812.5	62	107	1.37E+08	1.727802	21	23.08234	484	14.35834
210074.9	38	91	5.41E+08	1.925367	29	25.67953	565	17.64811
62658.58	60	111	2.56E+08	2.062344	20	12.01	363	20.38522
31658.73	59	103	1.24E+08	1.609748	20	4.11407	147	13.53953

14649.68	41	78	20628800	1.514671	19	5.033097	136	12.05033
11473.58	40	104	28140245	2.097709	33	3.778901	176	19.99716
28701.78	33	84	71919774	1.832811	23	4.676986	200	15.96016
960442.8	35	96	2.75E+09	1.986813	29	3.310836	265	17.93802
10480.89	43	101	33870495	2.013842	29	3.553605	165	18.22959
113075.7	37	74	1.05E+08	1.45429	18	7.558806	121	12.13165
217114.6	29	72	2.65E+08	1.521657	20	5.601696	109	13.17826
258748.1	26	88	2.61E+08	1.913163	31	3.30799	253	18.61173
14166.69	57	93	35441785	1.521827	18	5.476062	155	11.76706
7379.15	39	83	10132532	1.685728	22	3.571185	127	13.90106
9018.459	51	95	16416383	1.634677	20	4.15277	157	13.11729
168587	11	72	1.59E+08	1.978838	34	2.505673	133	19.02863
835182.2	26	71	8.69E+08	1.630214	23	3.241188	167	13.69939
277715	18	83	5.05E+08	2.042442	44	1.994052	153	21.71003
515222.1	30	85	1.06E+09	1.918323	32	154.5515	788	18.7838
273607.8	37	75	5.19E+08	1.401819	18	965.4347	864	11.99884
2864.018	63.7	94	8766282	1.24101	15	4.296143	114	9.778091
41951.89	47	104	1.12E+08	1.913741	33	2.468758	136	18.1247
20808.89	0	0	10	######	0	888.6011	1	0.002243
28335.11	18	73	36340084	2.007217	27	7.306084	138	18.36998
169620.7	22	76	2.05E+08	1.900053	29	7.61975	408	17.0172
44169.24	56	90	1.03E+08	1.366278	17	3.527454	130	10.66553
35771.38	12	119	4.49E+08	2.74577	61	2.916698	385	33.72018
111573	38	104	3.76E+08	2.01105	26	3.624702	213	19.00675
9174.764	38	87	74053407	1.805033	26	4.269896	201	15.60787
232741.7	41	83	4.22E+08	1.600765	22	16.01007	383	13.21622
668713.6	39	78	1.07E+09	1.499725	21	3.152868	128	12.40213
93533.17	38	77	89886077	1.496384	20	3.686853	130	12.24811
27407.67	37	86	2.66E+08	1.786229	25	5.421464	376	15.39034
53990.59	28	78	76911156	1.783329	25	4.54103	127	15.88815
5893.784	37	77	7907710	1.595999	20	13.97205	115	13.32947
87415.55	46	101	2.46E+08	1.870347	24	4.976141	161	17.14482
319757.5	13	66	1.81E+08	1.842484	32	3.362701	152	17.58359
72244.15	60	105	6.47E+08	1.754952	22	55.24015	484	15.42314
25662.28	58	117	1.6E+08	1.951058	28	3.846442	164	18.07331
68060.23	31	121	5.28E+08	2.455181	50	2.413912	177	27.76657
9969.894	66.2	108	49873915	1.609667	21	11.3772	275	13.25126
93305.59	45	83	2.25E+08	1.460343	18	8.056602	261	11.75943
3294.945	75	118	27766728	1.66441	21	10.64106	166	14.02136
4087.321	50	96	22317597	1.657968	23.25	2.916639	134	14.05656
14934.87	12	81	40329733	2.159657	35	2.86447	130	21.05303
41720.58	57	103	2.73E+08	1.745566	24	4.745735	161	14.81005
6946.562	55	108	48548548	1.891034	24	4.087955	187	16.2749
85420.47	70	111	5.82E+08	1.614864	20	5.13904	154	13.40117
59546.12	23	74	51764103	1.828193	26	26.48386	474	16.35708
13828.84	65	110	1.02E+08	1.679046	21	4.831279	150	14.11237
27163.22	39	84	46690545	1.757223	22	13.73761	279	14.80354
57735.34	56	87	1.34E+08	1.459588	15	13.89673	128	11.32013
8206.367	79	110	62511630	1.36754	16	8.046341	153	10.28076
24492.31	65	99	77708091	1.389806	17	5.830952	136	11.01879
108816.6	34	82	4.22E+08	1.736416	24	8.360525	335	14.95341
247408.6	32	84	9.91E+08	1.859014	27	205.7341	1004	16.67316
238625.9	30	78	3.76E+08	1.751135	23	50.74787	588	15.24563
16349.12	74	107	1.24E+08	1.31759	17	3.53812	157	10.49842
19725.27	34	75	59195544	1.577008	21	4.012738	144	12.74702
525399.6	12	63	4.63E+08	1.751997	32	419.3372	280	18.30091
29509	29	76	62928258	1.836054	24	5.692449	183	15.5372
59377.27	48	82	2.52E+08	1.318285	17	4.893168	133	10.48784

15096.17	19	81	71941203	2.069617	33	3.520062	241	19.49125
13692.92	23	74	31144062	1.790247	28	2.75579	131	16.00621
309416	42	83	6.01E+08	1.648393	20	103.3964	789	13.65805
9499.896	63	97	73242968	1.508643	17	9.070076	145	11.91476
6879.171	47	87	15415440	1.51612	21	3.765435	134	12.58655
136170.1	65	118	1.32E+09	1.950731	25	368.499	1475	17.4006
8568.789	33	86	28351308	1.821968	30	2.625861	160	16.77184
87569.76	36	70	1.13E+08	1.415849	17	5.17589	116	10.93875
11575.03	50	87	23936411	1.459919	19	3.756639	129	11.73529
21836.43	80	116	2.04E+08	1.452049	19	3.836445	168	11.6874
43759.05	49	99	2.55E+08	1.785986	24	3.364449	152	15.3379
33946.62	19	76	39693253	1.945272	29	4.517236	108	18.17687
58339.87	30	93	3E+08	1.943345	23	7.654273	324	17.64261
85737.17	63	97	5.56E+08	1.379887	17	8.514817	219	11.00429
221192.5	44	88	3.71E+08	1.775584	20	12.53411	354	14.53273
30141.77	43	109	1.89E+08	2.007968	29	4.028749	146	20.06526
151375.3	17	79	1.4E+08	2.085295	33	3.54898	160	19.60988
667438.8	38	90	1.57E+09	1.808793	27	55.96447	699	16.15327
5752.563	76	112	87300251	1.432854	17	3.991117	171	11.18593
18876.28	48	90	81479837	1.614141	20	5.586289	135	13.01886
8800.825	22	77	24782532	1.861451	30	2.57441	117	16.93346
35834.7	64	104	2.19E+08	1.58966	20	6.422496	162	13.09992
17212.23	88	143	2.19E+08	1.961488	30	4.06364	202	17.71919
21585.95	50	78	28175583	1.101352	15	5.085656	106	8.84882
162167.8	30	88	6.48E+08	1.936212	33	3.002162	189	18.26708
31582.21	26	104	1.59E+08	2.095521	30	3.301756	183	22.19819
41378.35	35	103	99917818	2.049147	38	2.66621	145	21.30632
110826.8	34	68	1.18E+08	1.431117	17	1177.329	113	11.16788
11472.2	38.3	84	12380240	1.693323	23	4.356739	127	14.16923
22872.58	37	103	29578376	2.249187	32	7.390594	226	21.96816
4931.249	16.4	108	6609237	2.620412	43	4.304012	159	29.16617
21830.63	63	122	70311022	2.060323	27	4.447834	177	18.63434
154691.2	25	86	1.8E+08	1.998479	33	2.930754	177	18.93392
201505.1	40	85	2.36E+08	1.720818	22	4.639361	172	14.4322
14114.69	54	98	35060964	1.663557	23	3.9555	171	13.96848
4843.983	30	95	8092807	1.979216	26	5.183899	129	19.88157
33911.53	21	112	60740015	2.697644	54.75	3.639346	253	32.34506
79371.08	26	81	79845979	2.039389	30	13.82417	446	19.72041
131147.9	58	92	2.06E+08	1.373023	17	8.304331	123	10.81391
54478.27	25	87	1.77E+08	2.083029	25	7.337181	148	20.41689
55291.99	65	103	1.11E+08	1.489241	17	15.47376	313	12.18755
13892.95	11	85	10988271	2.31645	31	5.433423	141	23.79762
114132.5	15.4	132	1.57E+08	2.953639	66	4.330803	418	38.66693
4653.397	0	69	2649331	2.214627	31	5.002178	110	21.44886
52042.49	66	100	1.09E+08	1.384582	16	6.392625	159	11.21855
28527.66	47	119	67599431	2.377919	35	7.082653	378	23.85086
22543.4	34	96	38127862	2.17658	31	5.763212	186	20.67471
221008.5	47	88	3.2E+08	1.508223	20	14.80796	373	12.84948
35997.62	63	128	78254036	2.131852	26	5.023901	193	19.73108
157136.9	40	79	2.98E+08	1.559883	20	335.5094	256	12.74971
43656.65	33	84	48564624	1.888035	24	6.978678	241	16.68233
38113.76	10	85	30427687	2.356268	38	3.498456	167	23.70654
3177.658	8.6	73	2007288	2.052311	35	2.571243	110	19.71604
35276.45	36	84	74885889	1.805378	25	12.34012	291	15.50795
70915.22	29.5	82.5	64447248	1.960019	26	5.53573	205	17.32353
6524.354	69	133	24683132	2.171784	32	5.782737	185	20.75154
428130.6	21	78	4.14E+08	1.999087	36	130.8118	887	19.76081
29580.57	27	85	26184660	2.02884	26	5.112566	138	18.88647

153212	28	84	1.49E+08	2.000257	29	5.749687	125	18.82772
42928.45	55	106	3.02E+08	2.181044	23	48.46947	1145	39.14712
40362.3	26	92	33542313	2.144805	29	4.254885	147	20.27904
33000.18	52	99	54451862	1.819311	21	5.830348	171	14.90679
127821.9	38	85	1.49E+08	1.810046	23	11.69139	334	15.61908
35953.41	25	76	18368346	1.812504	23.25	4.819794	149	16.19018
70816.95	48	98	1.76E+08	1.844111	23	6.400201	236	16.29217
51458.2	1	72.1	31695622	2.29033	30	5.039708	232	22.122
1840976	25	74	1.91E+09	1.915582	24	159.0075	1274	17.44101
66845.76	15	72	54209798	1.870495	21	6.819427	123	17.76109
18669.42	15	82	17389985	2.118725	29	5.006019	128	20.88769
734552.2	58	86	1.74E+09	1.346867	13	16.66137	148	9.538508
158223.6	33	88	2.48E+08	1.942609	31	5.569609	290	18.04154
335822.7	14	76	1.84E+08	2.09186	38	3.265813	192	20.81491
4123.169	42	100	6440688	2.107136	31	5.557452	152	19.68671
58435.04	25	87.2	62814391	2.089749	28	4.953342	168	19.89653
275529.7	16	64	1.49E+08	1.830235	23	5.444745	185	15.60873
139909.5	24	78	95312318	1.933018	28	4.183628	158	17.37112
17270.99	64	103	38778582	1.56409	17	6.099087	149	12.8317
27308.58	35	83	29126812	1.854566	23	6.24923	163	16.35461
106636.2	43	103	3.86E+08	1.93508	28	3.56846	167	18.57498
2335.968	37	104	3952422	2.104124	29	4.639102	120	20.92017
270204	32	70	2.46E+08	1.423016	20	3.198733	103	11.67183
138589.6	27	90	1.62E+08	2.131165	36	4.484357	247	20.55869
3013.184	-45.7	92	1764036	3.010139	76	2.187368	145	42.84202
82776.34	10	80	65191608	2.24558	32	8.191096	369	21.59947
203948.3	34	93	3.33E+08	1.988923	28	7.350618	298	18.19507
70571.15	18	79	72013131	2.013195	37	3.061451	188	19.71771
72159.16	53	98	1.19E+08	1.7964	23	7.39043	268	14.93862
3511.491	-12	83	4944691	2.515476	51	3.082998	105	29.98696
57288.63	16	71	36443273	2.026011	27	7.485242	154	19.11607
35473.58	15	97	76263384	2.289464	27	4.426303	163	23.34505
36037.97	58	127	91352263	2.329396	32	7.71469	331	22.81836
35385.4	-2	75	28996184	2.387429	47	3.687305	127	26.71699
836.2393	45	95	1506499	1.764571	34	2.019054	113	16.90779
20521.99	38	69	33795323	1.387164	15	29.64806	311	10.11795
2037.183	80	134	11031050	1.864048	31	2.636975	173	17.59445
75452.16	37	65	1.25E+08	1.328721	14	1590.315	159	9.521401
258576.4	45	85	6.36E+08	1.535984	19	5.152144	248	12.42292
34327.76	14	46	15781049	1.26635	16	5.822497	105	10.13304
99797.46	38	87	1.92E+08	1.746719	28	2.866336	169	15.82423
21223.87	30	63	20390592	1.360524	16	5.633624	133	10.76289
20198.71	28	71	19473784	1.648864	19	8.28563	137	14.10773
17299.53	54	81	45450226	1.168525	13	6.061982	119	8.757914
73405.78	54	88	1.55E+08	1.598541	16	15.326	160	12.51419
443146.8	31	73	4.4E+08	1.607272	22	5.796478	124	13.35097
36267.92	36	94	2.45E+08	1.959089	33	3.17767	229	18.46041
13942.82	20	58	10008147	1.565222	17	5.972461	119	12.89186
23992.29	31	67	28557244	1.411151	19	3.361572	115	11.37521
10446.5	60	103	31348965	1.754657	22	5.618549	210	14.35205
417852.8	18	64	2.31E+08	1.735413	23	31.80341	153	14.65103
10326.46	30	108.6	15943802	2.452209	47	4.866666	248	26.46893
152318.9	42	87	3.51E+08	1.815407	22	204.9479	1078	16.08866
2706.405	72	121	11248594	1.890446	18	10.92796	158	18.82002
15117.26	39	86	21610389	1.792182	23	9.112228	152	15.31583
29881.85	33	72	27475509	1.518774	17	7.597163	126	12.98193

291976.1	19	47	89392745	1.178553	14	20.7622	226	9.766331
464893.8	31	91	8.42E+08	2.014078	28	5.497662	169	18.12766
361351.9	27	72	4.01E+08	1.559197	23	4.00214	147	13.98532
207803.8	47	79	7.07E+08	1.455106	15	287.744	1647	15.86501
82550.95	62	86	1.98E+08	1.186958	12	8.480332	191	7.92517
34234.94	12	48	16668989	1.489122	19	4.106164	87	11.61224
25004.38	35	71	37254410	1.618708	16	9.521227	244	12.30973
100158.9	36	64	1.15E+08	1.296472	14	1738.122	257	9.279806
10144.36	55	121	47620524	1.939533	32	2.936227	163	19.79435
14121.32	34	65	13782002	1.254456	15	3.731318	119	9.500384
13944.25	50	86	65813331	1.525523	18	9.48571	128	11.87872
23687.74	58	90	1.72E+08	1.342782	16	3.961573	133	10.2299
2232.729	70	128.9	32161463	1.947082	31	3.262972	180	18.41566
12890.42	52	79	23839793	1.142767	14	5.190009	110	9.007923
76076.98	58	86	2.34E+08	1.300352	13	17.29069	343	9.71939
7644.622	41	83	15622490	1.714437	23	6.745597	138	14.0947
16609.95	45	79	38836779	1.459186	17	17.37897	264	11.55878
297536.7	78	116	1.13E+09	1.502622	21	5.485437	225	12.55874
67579.91	30	69	1.72E+08	1.614695	18	9.125797	166	13.52626
67695.47	51	83	1.37E+08	1.436461	16	9.943784	189	11.23921
86168.07	43	77	1.14E+08	1.449834	17	8.70342	169	11.49031
282676.9	16.98099	94.96432	5.48E+08	2.4436	46.97458	4.645986	297.9979	26.97474
136553.3	39	67	1.32E+08	1.269011	14	8.806824	188	9.378266
1848.142	-5.4	97.4	2408202	2.600156	53	4.283633	126	34.94297
33052.8	43	80	27463640	1.506355	19	12.10633	257	12.17566
204041.7	45.03653	77.98334	2.84E+08	1.346045	15.96582	9.237239	213.0006	10.87346
259989.9	44	78	4.02E+08	1.445767	15	21.21641	130	13.25017
71027.52	-89	82	1.41E+08	2.766569	114	2.051346	122	58.84106
5074.644	-3	609.4	2.1E+08	4.610041	201.75	8.140682	1758	216.5288
3189.045	23	77	3285555	1.985354	27	6.809462	115	17.8762
42248.28	-21	76	1.04E+08	1.81008	16	7.225108	135	28.53411
12105.42	48	92	28201502	1.920357	18	11.6193	200	16.83538

DX23	DX24	DX25	DX26	DX27	DX28	DX29	DX30	DX31
46.79453	46	-80	209	9.600862	50.18149	0.166158	65510140	0.379794
28.46447	25	-44	136	9.503378	33.03411	0.602026	28162804	0.386167
75.73627	76	-1	393	5.761953	76.70461	1.104078	3.57E+08	0.458281
88.62639	88	41	107	6.586829	89.61923	0.591334	12854012	0.558345
64.79284	70	-83	223	10.88843	70.04379	-1.65451	56330116	0.319424
27.13284	26	-73	189	9.407174	33.38813	0.376294	45939972	0.358005
44.54804	45	-1024	1152	12.15487	68.08115	-16.6037	1.08E+08	0.308887
47.02471	45	-62	200	12.20229	52.4359	0.435469	1.1E+08	0.303943
62.47304	62	-24	157	13.06207	66.9032	-0.14805	13618200	0.287668
76.20203	78	-1024	1389	9.445935	82.1667	-18.1371	6.66E+08	0.35588
46.05149	48	-102	289	12.11706	50.21299	-0.26601	1.11E+09	0.327938
72.20035	72	-27	233	9.96517	74.75238	0.165463	4.35E+09	0.343716
70.48244	72	-43	232	11.22329	73.70038	-0.28996	3.76E+08	0.325604
87.69587	87	-16	189	11.70293	90.47853	0.082013	91425064	0.316877
81.55723	80	-10	179	9.826568	84.33225	0.003549	31955048	0.331745
60.86425	62	-56	195	8.39265	63.06208	-0.09049	9.01E+08	0.432546
63.63939	64	-22	142	8.360221	65.53452	-0.30281	29378889	0.438409
46.39642	42	-86	473	16.55203	53.28299	0.964238	2.56E+08	0.260133
85.05794	89	-44	187	11.99066	88.2255	-1.12358	5.26E+08	0.315307
62.13813	69	-91	285	14.05512	66.97814	-0.82194	1.3E+09	0.296887
53.44552	56	-57	220	17.80502	59.19176	-0.08723	1.3E+09	0.2533
57.26622	61	-95	624	15.00479	64.26917	0.24357	4.87E+08	0.261398
49.86414	51	-66	183	10.01552	53.89847	-0.57362	25117786	0.351072
57.22407	59	-58	426	12.95852	61.14059	0.39026	5.67E+08	0.309974
62.44155	65	-96	287	8.963654	65.21857	-1.13283	3.49E+08	0.398262
69.20501	70	-20	218	6.274184	70.66968	0.115491	48563454	0.471787
62.88566	63	-996	1241	5.447145	68.43622	-30.8962	1.67E+08	0.663684
71.78491	75	-37	297	15.35907	76.52854	-0.22043	2.66E+08	0.264474
70.79501	71	-66	245	10.4277	73.79394	-0.35227	1.31E+08	0.342593
56.37758	60	-75	212	15.56038	62.39102	-0.67385	1.56E+08	0.261832
73.6185	73	9	125	7.416746	74.9392	0.029218	48820508	0.427641
58.45685	60	-44	192	10.98046	61.89257	-0.29584	4.98E+08	0.341347
59.54877	60	-60	217	8.666682	62.19808	0.155219	55257611	0.408992
53.62027	53	-87	226	16.99398	59.01252	-0.155	3.94E+08	0.272493
46.91477	48	-49	1369	11.53425	50.82813	6.497972	2.47E+09	0.359183
77.00985	78	-14	173	5.243248	77.81547	-1.24611	1.08E+08	0.516719
61.46716	64	-37	474	17.88662	67.51056	0.183946	1.56E+09	0.236854
28.88456	28	-41	149	8.0783	33.2195	0.361373	8504310	0.396456
72.34918	73	-58	282	6.628651	73.73375	-0.85072	1.07E+08	0.446407
71.41278	72	-67	252	10.80583	76.78901	-0.47607	42284973	0.299707
71.65473	74	-88	269	10.48874	75.51845	-1.02104	2.64E+08	0.332386
75.69668	84	-78	540	17.35642	81.45973	-0.47783	3.37E+09	0.275741
60.86174	62	-82	252	18.84955	68.47471	-0.02899	1.21E+08	0.214568
69.74575	70	-73	695	10.31164	72.85105	1.818953	1.02E+09	0.346282
61.75366	64	-82	454	8.285222	63.82614	0.150838	6.76E+08	0.436746
89.45433	89	36	100	7.680939	90.46639	0.204355	82512812	0.481787
61.57185	62	-1	121	7.831307	63.2099	-0.21816	59556395	0.464266
64.48282	71	-41	285	16.47221	70.17928	-0.45083	4.15E+08	0.262456
72.47807	75	-16	298	11.15001	75.66722	-0.45124	4.68E+08	0.324147
71.31989	74	-25	421	11.89365	74.95397	0.839871	3.76E+08	0.322754
66.63688	68	-66	209	7.46764	68.38187	-1.05079	6.57E+08	0.443312
79.3215	80	9	385	6.795408	81.13298	4.054903	66946991	0.482332
62.35521	65	-1024	1161	8.797918	68.34216	-20.9653	1.07E+08	0.39862
84.65665	84	-35	519	9.32671	86.95396	1.153396	2.56E+08	0.38192
67.36927	69	-80	645	11.96155	71.7766	1.664061	1.08E+09	0.319004
81.20227	84	-119	482	9.06651	88.74351	-2.08565	4.93E+08	0.365334
82.94965	85	-14	161	8.850117	84.85391	-0.70348	2.28E+08	0.415924

59.64767	60	-24	160	8.001822	61.78447	-0.04393	55922522	0.443954
69.43321	67	-61	237	13.80465	73.97001	0.308586	62778389	0.280413
62.04892	65	-54	254	10.28635	65.58523	-0.77115	1.23E+08	0.349605
68.45967	70	-81	346	12.46065	72.20054	-0.10027	5.01E+09	0.295811
73.2674	74	-22	187	12.31032	76.96419	0.07923	62083380	0.296322
56.01961	58	-69	190	7.483702	58.61788	-1.5393	3.89E+08	0.483794
53.52895	57	-83	192	8.811535	56.27515	-1.08977	6.88E+08	0.440043
62.65546	68	-44	297	13.57272	66.74808	-0.50814	1.15E+09	0.301236
74.5328	74	-8	163	7.65786	76.2367	-0.24104	82337281	0.41108
62.52381	64	-7	134	9.440651	65.05743	-0.41973	31232027	0.377225
72.30943	71	-15	172	8.824014	74.3465	0.426753	49848644	0.38613
43.22755	45	-67	200	14.2575	49.0023	-0.13932	4.05E+08	0.285633
49.15448	50	-65	232	9.757624	52.11022	-0.21658	2.27E+09	0.374395
52.7829	57	-21	174	17.29029	58.45776	-0.14675	9.49E+08	0.260877
60.94989	63	-63	851	13.15704	69.21288	8.98711	2.47E+09	0.306261
58.08146	60	-1006	1870	7.840123	62.37501	-14.0601	1.06E+09	0.481365
79.8864	81	30	84	6.232088	80.94307	-0.78353	18764420	0.522277
75.84979	77	-5	141	13.66101	78.92803	-0.21167	2.61E+08	0.296229
0.001123	0	0	1	0	0.033509	29.79264	23.36502	1
46.67232	50	-97	235	11.76524	53.17708	-1.29593	80126070	0.31503
48.50408	48	-68	476	12.12363	53.14341	0.424387	4.79E+08	0.316803
72.38875	72	10	120	7.42077	73.66518	-0.13984	2.4E+08	0.43806
64.88964	64	-95	480	25.42564	76.60351	0.137883	2.1E+08	0.165883
77.26209	82	-33	246	11.94692	81.3311	-0.57458	7.38E+08	0.327892
62.65024	63	-83	284	10.71707	65.80159	-0.25916	39725343	0.343849
64.19606	66	-26	409	8.987946	66.55246	0.464463	1.03E+09	0.393396
58.80659	59	-31	159	8.700542	60.83977	-0.20047	2.48E+09	0.419663
57.74515	59	-35	165	8.45857	59.84126	-0.39367	3.35E+08	0.432508
61.33414	61	-71	447	10.68896	64.44498	0.131599	1.14E+08	0.346519
56.10744	60	-64	191	10.62491	59.83528	-0.87997	1.93E+08	0.368734
56.22004	57	-90	205	8.621265	59.34952	-1.85651	20760062	0.407289
77.81378	82	-71	232	10.97962	81.1149	-1.00878	5.75E+08	0.358012
36.34358	32	-111	263	13.08694	42.13084	0.398379	5.68E+08	0.310139
85.32453	86	5	479	9.348246	89.27587	4.645072	5.76E+08	0.387759
90.12457	94	-38	202	12.30506	93.08682	-0.78083	2.22E+08	0.308454
71.79871	67	-61	238	20.85696	79.23455	0.306634	4.27E+08	0.204543
86.127	84	-33	308	8.659629	88.05012	1.105875	77294827	0.409841
63.90827	64	-33	294	7.982218	65.82746	0.475908	4.04E+08	0.458143
98.24385	101	-47	213	8.75003	100.229	-1.79478	33100519	0.38825
73.203	73	17	117	10.02801	75.30021	-0.03509	23175607	0.364387
48.71525	51	-71	201	14.81928	55.37539	-0.27758	45796795	0.258546
80.14081	81	-46	207	9.897554	82.49525	-0.5986	2.84E+08	0.364018
80.37425	78	13	174	10.38144	83.24402	0.606143	48136668	0.326833
91.21006	93	-35	189	8.576052	92.98009	-0.98706	7.38E+08	0.396099
50.38348	53	-65	539	11.33168	54.8545	0.966299	1.79E+08	0.339654
89.51979	92	-12	162	8.979398	91.52012	-0.93081	1.16E+08	0.3862
61.45149	60	-94	373	9.409855	65.00299	0.858647	1.15E+08	0.379983
70.88562	74	-59	187	6.309655	73.17529	-2.67316	3.09E+08	0.430207
94.18129	95	3	150	6.507486	95.29278	-1.26586	74519670	0.461096
81.80614	83	-13	149	7.298189	83.14765	-0.78788	1.69E+08	0.478402
60.42882	62	-55	390	10.35076	63.48778	0.219577	4.39E+08	0.361738
59.10322	59	-49	1053	11.31278	64.48096	8.014358	1.03E+09	0.330972
57.01306	59	-93	681	10.10223	61.15886	2.958208	8.93E+08	0.374417
90.59423	91	36	121	7.178378	91.59236	-0.00173	1.37E+08	0.489129
53.30255	52	-9	153	8.630776	55.8245	0.582974	61471344	0.38939
33.65982	32	-1024	1304	13.02603	57.66592	-18.5124	1.75E+09	0.321313
52.15816	51	-58	241	9.815637	56.33566	0.593277	93652906	0.347917
65.02289	65	-76	209	7.438928	66.40941	-0.36074	2.62E+08	0.488022

50.04151	50	-66	307	13.81528	55.68465	0.109548	46809918	0.276378
47.43538	46	-9	140	11.57736	51.37656	0.239806	36143169	0.331778
63.66915	64	-82	871	8.567516	67.36899	4.641631	1.4E+09	0.415158
79.16141	80	-32	177	7.192453	81.04134	-1.44542	62392445	0.443665
67.02341	68	-38	172	8.72704	68.9134	-0.27219	32669578	0.409534
92.13767	91	-66	1541	11.01179	96.96354	12.02415	1.28E+09	0.327182
60.65136	62	-2	162	12.4876	63.97554	-0.14343	35070936	0.317016
53.03896	54	-62	178	7.24077	54.99063	-0.46672	2.65E+08	0.442611
67.82539	67	-6	135	7.942336	69.51068	0.156894	55927474	0.440907
97.43813	97	8	160	7.922357	98.59203	0.159866	2.12E+08	0.425655
75.28558	77	-6	158	10.47736	77.81397	-0.32814	2.65E+08	0.342634
49.38845	53	-65	173	12.20051	54.80765	-1.0233	1.02E+08	0.318649
68.89583	73	-37	361	10.76726	73.11773	-0.20986	3.12E+08	0.320486
80.6239	82	-66	285	7.120779	82.05672	-0.98799	5.77E+08	0.488809
67.51646	70	-80	434	8.608312	70.8197	0.033788	1.11E+09	0.372771
83.17006	90	-106	252	13.15822	87.12474	-1.11354	2.29E+08	0.30234
45.49761	43	-84	244	13.58391	51.88286	0.288148	4.07E+08	0.279925
66.60613	69	-30	729	11.41999	70.13061	2.635306	3.28E+09	0.329953
94.64618	95	44	127	7.609379	95.76616	0.164951	52757667	0.433677
68.95525	70	-43	178	8.523242	71.15961	-0.68747	95583647	0.395652
49.95068	51	-27	144	12.51922	54.0758	-0.10782	25735308	0.311667
84.74091	86	-60	222	8.199659	86.64733	-1.02265	2.69E+08	0.434026
112.5687	110	-12	214	12.49314	114.8409	-0.15314	2.27E+08	0.305949
64.14514	65	-23	129	6.14414	65.16524	-0.51456	91664906	0.584786
60.42717	62	-69	258	13.72156	64.40357	-0.22728	6.73E+08	0.295591
75.70037	83	-20	203	14.49531	80.98501	-0.86604	2.07E+08	0.30268
74.19265	80	-26	171	15.69033	78.54725	-0.64439	2.55E+08	0.284902
50.16961	51	-1024	1137	7.355454	53.75374	-21.6549	3.2E+08	0.430994
60.53329	60	-67	194	9.535166	63.25639	0.007562	45904515	0.3725
70.75723	74	-106	332	13.43773	77.32434	-1.26362	1.37E+08	0.271148
63.9566	67	-87	246	18.48561	74.99922	-0.87401	27737694	0.201414
95.35133	98	-12	189	11.6083	98.65585	-0.59757	2.12E+08	0.298597
58.62554	62	-79	256	13.87452	63.10666	-0.21405	6.16E+08	0.289123
63.15799	65	-44	216	9.691235	65.95488	-0.58099	8.77E+08	0.37171
76.28616	76	-18	189	9.69688	78.3599	-0.02621	86668032	0.371714
68.76046	76	-47	176	11.94828	73.89678	-1.42074	26451701	0.329825
64.85583	66	-77	330	22.55976	76.74037	-0.37105	2E+08	0.185367
51.0872	52	-89	535	12.46786	58.86881	-0.47457	2.75E+08	0.306788
74.84628	76	-64	187	7.375009	76.22779	-1.09335	7.62E+08	0.44195
59.76035	66	-112	260	11.28657	66.78629	-1.70962	2.43E+08	0.317962
84.44233	86	-29	342	7.653048	86.27353	-0.16397	4.12E+08	0.471558
49.15385	53	-96	237	13.61892	59.71987	-1.22549	49548683	0.260394
70.0204	69	-109	527	27.30015	85.43822	-0.08213	8.33E+08	0.150955
35.54821	39	-100	210	13.53856	45.94585	-0.93058	9823417	0.27183
83.46257	84	0	159	6.892402	84.98607	-0.57405	3.76E+08	0.516287
83.90335	87	-81	459	15.05603	90.08969	-0.39776	2.32E+08	0.244544
65.12034	68	-81	267	12.81563	71.12495	-1.04887	1.14E+08	0.282179
71.26021	75	-56	429	8.718632	73.24301	0.183777	1.19E+09	0.39944
92.43162	92	-39	232	11.78777	96.51346	-0.40586	3.35E+08	0.30763
59.64275	60	-820	1076	8.382695	62.74708	-8.85022	6.19E+08	0.428139
59.88126	62	-75	316	10.48717	64.2241	-0.91188	1.8E+08	0.346067
49.7214	52	-84	251	16.009	58.33664	-0.2775	1.3E+08	0.236839
41.91365	44	-28	138	14.39708	48.39655	-0.21419	7442795	0.270433
60.15284	60	-98	389	10.45344	63.78699	0.482944	1.44E+08	0.351636
56.39256	58	-70	275	11.03302	61.11279	-0.51128	2.65E+08	0.325825
97.59983	96	-52	237	13.74987	101.5402	-0.57172	67268798	0.276149
49.99989	49	-99	986	14.7892	57.53199	6.405484	1.42E+09	0.287524
57.61738	61	-65	203	11.57488	63.17883	-1.04642	1.18E+08	0.316668

57.82697	62	-91	216	12.2073	63.12177	-1.27911	6.1E+08	0.306687
96.55416	80	-51	1196	9.617262	144.929	6.403036	9.02E+08	0.337691
62.33209	66	-77	224	12.832	67.99981	-0.79568	1.87E+08	0.283401
75.35955	77	-41	212	9.094103	78.21884	-0.72499	2.02E+08	0.352176
62.03583	64	-105	439	9.983927	65.79805	-0.56436	5.53E+08	0.359246
53.54073	57	-56	205	10.22876	57.79013	-0.98498	1.2E+08	0.365122
75.99013	79	-53	289	10.19816	79.23605	-0.7539	4.45E+08	0.359359
38.98593	41.5	-94	326	13.06999	49.78109	-0.57966	1.28E+08	0.270271
51.38103	50	-129	1403	10.3515	61.12874	8.37184	6.88E+09	0.340743
48.37531	54	-97	220	9.710298	54.83592	-1.68922	2.01E+08	0.371935
52.29503	57	-82	210	12.96831	59.42189	-1.20511	65921017	0.29424
71.94425	74	-208	356	5.797225	73.39098	-2.53536	3.96E+09	0.446407
63.66541	68	-78	368	12.54721	67.75345	-0.55412	7.26E+08	0.305524
42.45667	40	-93	285	15.89409	49.3561	-0.14594	8.18E+08	0.264615
71.28058	74	-54	206	12.63244	76.1051	-0.95344	23881335	0.290039
59.18101	64	-79	247	12.34329	65.06198	-1.06618	2.47E+08	0.299845
39.64952	41	-83	268	9.901511	45.08374	-0.75059	5.6E+08	0.359102
52.05865	54	-68	226	11.91376	56.68103	-0.49178	4.49E+08	0.317246
83.66843	85	-21	170	7.455387	85.6672	-0.75684	1.27E+08	0.460032
58.46897	61	-56	219	9.916306	62.88689	-1.22279	1.08E+08	0.360867
77.82998	83	-49	216	12.3958	81.42435	-0.70385	7.07E+08	0.336112
74.04127	79	-28	148	13.28293	79.2066	-1.23975	14655129	0.28263
51.68703	52	-27	130	8.388858	53.7247	-0.29617	7.8E+08	0.429945
60.52428	62	-92	339	14.77743	65.89743	-0.00404	6.02E+08	0.269962
25.46324	34.5	-91	236	32.47124	56.94483	-0.09171	9770890	0.137138
47.13078	49	-86	455	13.86645	55.53989	0.008683	2.55E+08	0.262703
60.51945	58	-80	378	12.04411	65.26995	0.794235	8.69E+08	0.309655
49.59449	51	-41	229	15.05572	55.01848	0.170634	2.14E+08	0.280014
75.37614	76	-66	334	9.523748	78.19253	-0.00138	4.41E+08	0.3588
41.28027	47	-80	185	21.68141	55.55694	-0.78861	10838475	0.198061
44.08152	48	-105	259	11.24873	51.9914	-1.59901	1.55E+08	0.317772
62.34287	68	-75	238	13.58897	70.26677	-0.99726	1.75E+08	0.270144
92.82462	95	-78	409	13.67022	98.50824	-0.69498	3.5E+08	0.263011
37.71411	43	-102	229	19.01757	50.25734	-0.80628	89376437	0.227417
70.53737	72	18	95	13.62004	73.22026	-0.07118	4483252	0.325161
53.34561	52	3	308	6.489105	55.2953	2.557931	62747420	0.439455
104.5387	101	51	122	13.02942	106.6956	0.405417	23191194	0.314082
51.04672	51	-1004	1163	6.02258	53.90231	-26.0401	2.19E+08	0.455175
65.74752	67	-90	338	8.207047	67.78032	-0.42301	1.19E+09	0.421235
30.47819	31	-76	181	7.009547	33.24211	-0.39184	37933477	0.50094
61.84471	61	-35	204	11.8609	64.76167	0.338883	4.19E+08	0.330321
45.92984	44	-14	147	7.053282	48.19119	0.923283	49290124	0.475441
51.40028	55	-99	236	8.535389	55.19158	-1.57786	61527497	0.413049
67.184	68	-6	125	5.604343	68.27563	-0.63752	80642839	0.5526
70.14116	72	-104	264	7.003669	72.92043	-2.29627	3.9E+08	0.41146
52.38433	53	-109	233	9.092175	55.2133	-0.72949	1.35E+09	0.392059
65.91907	66	-18	247	13.62024	69.70553	0.237869	1.76E+08	0.291489
38.55859	40	-49	168	7.627223	42.70801	-0.80876	25431354	0.451077
48.52791	48	0	115	7.944103	50.62634	0.333885	61492894	0.429541
81.38425	81	-6	216	9.128169	83.73543	0.296527	73246922	0.371546
40.28317	39	-356	509	9.703179	45.25769	-1.58174	8.56E+08	0.376456
67.59928	67	-86	334	18.64292	75.66296	-0.33901	59117796	0.216381
64.62049	65	-101	1179	9.428971	71.90309	9.024729	7.87E+08	0.38011
90.23856	94	-91	249	8.50505	95.86463	-2.37851	24871934	0.387651
63.79119	66	-96	248	9.557125	67.34414	-1.43355	68560325	0.364431
55.28118	58	-84	210	7.450013	58.38917	-1.22799	1.02E+08	0.479399

33.67992	35	-112	338	6.103069	36.90963	-0.55049	3.98E+08	0.586389
60.3733	61	-104	273	12.04163	65.05311	-0.55715	1.97E+09	0.306703
46.49555	43	-85	232	9.86894	49.75682	0.680554	8.95E+08	0.421404
66.60638	62	-90	1737	6.608791	89.18888	14.96569	1.65E+09	0.531193
74.08678	74	14	177	5.258396	74.87414	0.902096	4.63E+08	0.476482
29.53229	29	-51	138	7.66802	33.24711	-0.07688	37842301	0.424092
51.97537	51	-71	315	7.168352	55.12437	0.45207	75980698	0.397659
49.63248	48	-1024	1281	5.921287	52.59831	-27.576	2.77E+08	0.458314
80.70425	74	15	148	13.98079	84.38183	0.767503	72230807	0.31619
49.55488	49	5	114	6.732857	51.02761	0.210972	36769320	0.459874
67.91875	69	-61	189	7.410268	70.00858	-1.36563	68343555	0.429732
73.30952	72	-2	135	6.826997	74.52028	0.35132	1.32E+08	0.449792
95.76178	92	15	165	12.78808	98.54231	0.464514	21681113	0.320961
65.41239	66	2	108	6.019628	66.48689	-0.63734	56982183	0.562909
71.69618	70	-5	348	5.733993	73.11221	1.793883	4.07E+08	0.501001
61.07805	61	-61	199	9.398483	63.96717	-0.60238	31280260	0.377925
60.95422	59	-16	280	7.097805	63.35395	2.271577	66667750	0.479167
97.45948	99	-65	290	8.522668	98.83787	-0.79179	2.91E+09	0.410719
49.66097	53	-102	268	7.755394	53.55916	-1.83919	1.94E+08	0.414865
66.74946	68	-46	235	6.867986	68.78079	-1.501	3.2E+08	0.466842
59.48046	59	-68	237	7.119297	61.75169	0.061579	3.29E+08	0.490398
53.60209	53.98846	-301.874	599.8718	19.17691	63.66269	-0.76625	1.15E+09	0.217939
53.05132	54	-48	236	5.811109	54.7144	-0.97912	4.09E+08	0.506494
55.08176	70	-96	222	22.31451	71.05379	-1.2915	9330607	0.2108
61.19924	61	-72	329	7.897288	63.5233	-0.08152	1.33E+08	0.455514
61.631	63.03268	-88.8733	301.874	6.977726	63.47559	-1.36713	8.22E+08	0.515282
60.74649	65	-122	252	6.562348	65.04877	-3.67499	1.1E+09	0.511215
21.26653	57	-142	264	47.22584	69.90063	-0.79443	3.47E+08	0.197607
212.7349	82	-94	1852	109.5862	374.9739	2.264299	7.14E+08	0.073303
48.09663	48	-88	203	11.75389	53.96968	-1.03185	9288814	0.313041
48.80149	63	-124	259	10.01448	65.7531	-2.27129	1.83E+08	0.466255
68.15055	69	-114	314	8.234789	73.77842	-1.43757	65892868	0.378432

DX32	DX33	DX34	DX35	DX36	DX37	DX38	DX39	DX40
328.4535	40.87782	8.859995	0.532301	1.512325	0.70508	0.355164	0.556762	1.247005
281.0268	12.79622	5.826255	0.972675	1.325555	0.588138	0.367864	0.475653	1.148861
147.6136	20.67257	4.10561	0.057586	0.864936	0.436725	0.32387	0.413093	1.023745
176.9702	9.12398	2.043063	0.087162	0.623366	0.35292	0.245071	0.327069	0.959985
708.0214	51.43777	50.18977	-5.70588	2.724783	1.245243	0.341576	0.689113	1.468699
378.5758	21.48119	11.46505	0.971707	1.736447	0.705902	0.412525	0.539712	1.250526
2650.515	1876.423	18905.49	-222.326	4.781043	0.954371	0.439047	0.680904	1.414967
538.2008	29.32096	25.6948	2.570241	2.640042	0.832348	0.514767	0.628311	1.321622
573.1578	16.21755	18.23777	0.057791	2.453553	0.746503	0.521669	0.578904	1.277669
944.616	1989.243	4472.708	-50.8297	2.466454	0.894569	0.377141	0.623025	1.374754
400.6048	54.64054	12.7582	-0.55256	2.173567	0.625647	0.551291	0.51392	1.202057
375.0274	29.38914	15.90384	0.558461	2.192447	0.514352	0.61868	0.447031	1.107111
463.9708	28.99168	19.02615	-0.96021	2.292366	0.647955	0.552785	0.517725	1.214001
495.7986	25.60226	19.84477	0.708229	2.366343	0.843907	0.469927	0.62994	1.330017
460.3466	22.57091	13.27095	0.245171	1.779171	0.746912	0.381062	0.57637	1.277527
272.3693	35.54293	7.589876	-0.07161	1.385447	0.481847	0.478606	0.421314	1.09276
244.8005	16.41409	4.364926	-0.30903	1.099435	0.498615	0.369327	0.445618	1.090781
686.4486	40.55139	76.43849	5.024971	3.624572	0.772888	0.642474	0.575449	1.281177
548.8844	36.06166	29.27444	-3.50606	2.658291	0.94022	0.471583	0.611218	1.371172
624.9239	49.51228	37.74869	-5.43357	3.651586	0.702084	0.67774	0.545918	1.258362
647.241	32.34959	28.84987	-0.57979	3.862117	0.652925	0.71067	0.532399	1.214827
851.1068	47.03211	90.71009	0.346267	4.061439	1.094062	0.567171	0.69713	1.4666
418.6126	30.72705	12.89272	-0.72715	1.75614	0.719412	0.406942	0.529839	1.264481
463.5773	33.57598	29.90231	0.599078	2.489075	0.678416	0.567314	0.522191	1.19859
354.5152	49.46954	15.18814	-1.25745	1.654463	0.603197	0.456274	0.470319	1.178606
204.87	18.16487	7.24904	0.408547	0.971941	0.575258	0.247918	0.463307	1.141737
728.9098	1853.055	4942.689	-59.9321	1.397076	0.307357	0.440097	0.269432	0.876306
703.5438	28.68215	37.40515	-1.73589	3.675559	0.949717	0.587336	0.654552	1.367671
433.6125	40.59214	13.83341	-0.2834	1.889406	0.727724	0.430492	0.55164	1.273877
714.2076	33.95814	37.29238	-2.43717	3.516234	1.153521	0.501895	0.69548	1.455792
196.2	11.79275	2.973869	0.149052	0.982468	0.542185	0.284054	0.491898	1.116564
413.4867	23.6307	16.80968	-1.19186	2.302984	0.636572	0.566157	0.52185	1.206009
322.5446	34.62304	9.048349	0.105221	1.350315	0.635303	0.350577	0.526509	1.220662
607.3443	44.89095	25.66402	0.117612	3.335087	0.708406	0.647225	0.537419	1.245154
382.5029	19.55302	542.549	7.407465	2.060316	0.501642	0.604727	0.415954	1.064936
124.73	21.61752	2.978233	-0.69158	0.738529	0.423885	0.26663	0.393493	1.029263
779.4635	25.01449	53.87142	0.846259	4.574699	0.623107	0.758748	0.501175	1.188534
269.2176	13.43363	5.452044	0.513757	1.165629	0.626203	0.291712	0.531311	1.185432
202.2631	41.39943	5.703349	-0.45145	0.936881	0.565683	0.241842	0.483083	1.138234
796.766	40.80427	53.89942	-1.97586	2.954854	0.83372	0.527825	0.561755	1.341928
568.6363	55.41181	32.81176	-2.1228	2.319894	0.882788	0.424994	0.603043	1.357194
905.7013	57.68548	94.16109	-7.47711	5.359983	0.724163	0.761916	0.540201	1.273152
984.6343	49.55917	66.74248	-0.18033	4.967685	1.234163	0.598154	0.768435	1.498442
442.8045	39.7201	100.0382	3.595219	2.25839	0.588935	0.576857	0.461908	1.146161
260.2625	48.82116	18.2607	-0.01272	1.433177	0.464443	0.507539	0.411802	1.062799
182.0904	9.70905	2.994645	0.191602	0.929704	0.446586	0.345219	0.406218	1.049168
204.3987	16.21269	3.935389	-0.15136	1.06829	0.474815	0.382445	0.425086	1.071953
767.0982	26.33857	46.05834	-4.23265	4.291951	0.967178	0.632965	0.651675	1.385226
472.4582	19.55187	22.70934	-2.42549	2.619648	0.599811	0.625843	0.503408	1.17881
531.5704	19.61637	76.4698	1.675098	2.847447	0.568002	0.660603	0.436118	1.123853
235.606	38.7192	5.817558	-0.55782	1.090665	0.542352	0.326782	0.462599	1.140957
290.66	13.86222	48.56791	2.941476	1.070624	0.738947	0.169172	0.500108	1.206976
782.478	1939.272	1552.355	-19.6041	1.723776	0.782816	0.326367	0.571973	1.309128
394.2441	35.12224	36.97159	1.320334	1.73783	0.678705	0.426749	0.501495	1.236532
613.2627	53.02047	148.7636	5.655403	2.904291	0.926683	0.506377	0.558888	1.322081
1281.602	78.71758	332.121	-21.48	4.9174	1.264205	0.542113	0.628783	1.449078
319.5407	23.57886	11.51649	-1.44444	1.712345	0.601428	0.482189	0.488694	1.19198

259.4762	15.25332	4.779852	-0.05501	1.026284	0.603236	0.237026	0.49365	1.199946
650.5916	39.35409	27.86637	1.77281	2.842818	0.913072	0.499814	0.630711	1.374276
451.353	36.80176	16.92277	-1.56499	1.975655	0.845507	0.388751	0.586911	1.335215
526.192	53.01801	30.05077	-0.85225	3.112436	0.589175	0.681705	0.488063	1.160669
555.3743	19.78273	20.94504	0.112651	2.472186	0.980462	0.421449	0.66894	1.396501
297.8597	33.9217	10.01892	-1.53046	1.18798	0.570352	0.329124	0.433657	1.165168
301.5444	44.69186	10.48214	-1.7392	1.521044	0.501942	0.498698	0.426085	1.115899
529.5988	25.55788	29.46365	-3.2689	3.170361	0.694237	0.641527	0.549705	1.255919
256.8963	20.26206	5.567467	-0.10275	1.060332	0.645179	0.222249	0.532014	1.205738
323.2427	16.08183	7.171778	-0.28197	1.479074	0.833574	0.28054	0.628834	1.320938
298.7484	19.34121	6.543423	0.577644	1.414683	0.535815	0.443095	0.464644	1.135683
532.604	27.61943	20.49266	-0.52796	2.92435	0.765525	0.584958	0.596411	1.302781
299.3123	30.20356	7.844948	-0.42007	1.59941	0.602409	0.451469	0.517206	1.182172
631.2743	13.55131	29.15309	-1.00729	3.850547	0.597996	0.730904	0.500387	1.172429
1075.534	36.41108	5075.731	119.3996	5.781451	0.834295	0.742398	0.488338	1.191534
517.1858	1921.564	2507.691	-14.868	1.819552	0.530675	0.499019	0.382201	1.041427
169.9437	7.393008	2.402054	-0.56079	0.743435	0.512184	0.172679	0.418069	1.111231
476.443	21.58242	16.08617	-0.448	2.57502	0.670165	0.583857	0.540627	1.221487
0.001122	1	0	0	0	0	1	0	######
649.4965	40.93701	43.75675	-3.8333	2.595707	1.040242	0.393344	0.648131	1.408231
471.5761	29.88375	30.9887	1.443246	2.332866	0.794915	0.487065	0.574718	1.295006
186.4264	11.68351	2.792096	0.019226	0.973101	0.463524	0.347004	0.424701	1.058414
1657.432	52.01215	196.1259	3.083252	8.943584	1.605942	0.694849	0.941509	1.698472
645.3167	32.01867	47.05355	-4.7568	3.547368	0.676145	0.677174	0.505426	1.241571
404.7968	49.78259	12.05779	-0.50254	1.791862	0.961327	0.300648	0.700527	1.424196
308.0952	26.08403	27.14018	0.155235	1.680016	0.551809	0.502843	0.463692	1.135186
243.2629	23.86803	5.582799	-0.15944	1.334074	0.514483	0.442635	0.453257	1.112901
246.4736	23.42232	5.113659	-0.51748	1.196179	0.651752	0.294217	0.540813	1.239034
391.2783	35.68637	12.96613	0.281985	1.781567	0.870927	0.341094	0.655765	1.368041
432.2158	33.76262	14.01505	-1.43356	1.869892	0.772033	0.406234	0.547136	1.301143
361.6718	46.01963	16.81048	-1.53882	1.266046	0.643107	0.291479	0.537242	1.217109
524.6416	44.90531	23.69912	-3.15542	2.252125	0.819622	0.45101	0.580512	1.339332
454.1517	48.45573	17.66689	1.885607	2.486677	0.670236	0.573317	0.532101	1.240506
689.9069	15.78177	490.7375	22.06923	3.364087	1.043733	0.513588	0.522764	1.2377
542.7191	38.11609	24.423	-2.73945	2.653192	0.807636	0.528526	0.596634	1.312112
1123.06	41.9653	91.99412	5.240467	6.126828	1.345719	0.640021	0.77717	1.510737
334.9634	35.5624	26.34872	2.644404	1.531113	0.716156	0.357475	0.534548	1.248809
248.9874	25.62474	9.196106	0.37656	1.250677	0.469776	0.44675	0.411036	1.0748
393.9955	42.48369	17.22266	-2.08519	1.50579	0.758949	0.292812	0.587614	1.263415
311.4423	12.12077	7.55193	-0.07066	1.61854	0.728344	0.382256	0.582645	1.239992
693.2577	30.34245	36.70229	-2.18147	3.657991	0.995679	0.570488	0.68417	1.41219
382.9174	33.25411	12.46232	-0.70713	1.711225	0.81005	0.346483	0.584876	1.29628
469.5471	13.50326	22.19546	2.052605	2.267758	0.660753	0.534116	0.54023	1.200638
326.0228	38.37462	15.37465	-2.18858	1.740562	0.614237	0.478189	0.509278	1.192559
470.521	30.82298	50.67374	0.702	2.215883	0.760588	0.484866	0.548281	1.27467
362.1389	26.66635	10.71318	-1.08472	1.538677	0.72639	0.342402	0.548377	1.253075
449.1027	48.52789	34.73067	2.225414	1.812569	0.887317	0.324028	0.597499	1.356993
329.852	41.04196	20.43424	-2.90622	1.342554	0.761363	0.249535	0.52827	1.241102
210.5979	18.56763	5.799117	-0.66087	0.985065	0.552745	0.2701	0.474367	1.130717
221.2869	22.95241	6.538072	-0.93806	1.093471	0.550892	0.328753	0.468274	1.153823
379.0566	35.49246	22.39652	-0.29285	2.008826	0.626076	0.521751	0.501124	1.182562
664.6031	23.96913	1220.406	26.87986	3.034457	0.921499	0.514466	0.523341	1.213006
489.9177	46.39655	154.2241	5.122517	2.301157	0.667843	0.538295	0.501131	1.208465
181.8462	9.70257	2.949218	0.013778	0.88436	0.477544	0.292495	0.428082	1.078859
275.2129	13.29316	6.266975	0.604326	1.353967	0.573046	0.394922	0.483681	1.14705
2192.375	1838.4	22267.18	-264.321	5.462443	0.488397	0.705835	0.436973	1.097489
453.2331	31.32567	18.17474	1.037919	1.808381	0.709092	0.395825	0.554299	1.261061
182.2329	50.75783	3.183406	-0.07675	0.894955	0.534311	0.250769	0.465011	1.134899

596.6279	30.52698	26.67177	-0.02258	2.942048	1.093461	0.456905	0.759215	1.489735
389.4361	11.74952	10.58683	0.360341	1.931481	0.830314	0.398094	0.624569	1.305
484.8198	50.62078	414.3718	14.11196	2.18575	0.905983	0.405935	0.540115	1.285376
301.1698	32.45691	11.9673	-1.60662	1.226482	0.69101	0.25014	0.495253	1.217731
256.9194	27.1576	3.856385	-0.07734	1.081711	0.756696	0.173306	0.609493	1.288574
912.5785	51.46694	2596.898	37.90689	3.318285	0.62924	0.630741	0.484282	1.168751
414.2817	15.31393	10.98376	-0.30199	2.022762	0.818703	0.418022	0.612728	1.293304
210.8386	32.15096	4.161129	-0.25289	1.063063	0.493377	0.362208	0.437891	1.09943
231.4501	17.57426	3.99233	0.292105	1.058751	0.57127	0.290444	0.490517	1.167376
226.1987	19.34846	4.331551	0.321717	1.099295	0.516441	0.349468	0.452096	1.094764
387.0949	20.89635	13.73753	-1.03926	2.007878	0.68586	0.486202	0.538672	1.223172
564.6595	31.31105	22.14453	-2.56402	2.242578	1.228293	0.278018	0.740532	1.522962
599.5658	28.25638	73.16569	-2.78485	3.482812	0.740697	0.649938	0.532782	1.244084
233.092	45.8806	7.191562	-0.75869	1.067113	0.467138	0.375986	0.388355	1.063357
456.9581	52.44478	62.84782	-0.05574	2.317562	0.741618	0.511279	0.549584	1.284934
673.4609	79.3991	43.5156	-6.31077	3.459748	0.923438	0.576184	0.616367	1.372113
621.7979	40.47923	30.89777	2.550846	3.094651	0.851448	0.56339	0.635403	1.354934
481.9256	27.05165	274.2517	8.199786	2.799587	0.594086	0.648835	0.436085	1.100916
213.2582	10.82155	4.141137	0.121144	1.089702	0.510659	0.355298	0.446074	1.076269
308.863	27.75522	8.572367	-0.56942	1.389221	0.634218	0.360386	0.502248	1.180899
429.1223	19.91637	11.58702	-0.09318	2.052343	0.952604	0.361987	0.675351	1.340891
326.7376	48.14309	9.820884	-1.04398	1.329658	0.708902	0.291136	0.519593	1.267371
516.7091	36.40591	18.79677	0.246218	2.302983	0.876849	0.432934	0.581952	1.334505
131.9095	16.64657	1.644058	-0.03067	0.595067	0.407934	0.183591	0.371664	1.032751
496.3772	36.02225	18.63588	-0.83711	2.626575	0.758137	0.548915	0.56412	1.269926
828.025	20.80624	73.03712	-9.47696	4.839535	0.864936	0.698715	0.585779	1.343465
665.1211	30.56523	32.84951	-3.79975	3.5241	1.056828	0.540953	0.707801	1.442426
372.4752	1895.962	817.6555	-10.1703	1.231025	0.560716	0.345906	0.473082	1.146706
337.0912	35.32914	7.928399	0.368995	1.466273	0.70632	0.340578	0.554449	1.259772
972.468	71.18427	85.1333	-5.93273	3.531842	1.78887	0.305892	0.912886	1.698283
1534.436	51.50292	159.7031	-10.7578	6.036288	2.584597	0.380047	1.152358	1.879685
641.1005	29.51478	30.59865	-1.85173	2.584213	0.984449	0.416449	0.645448	1.39116
545.4965	47.08398	24.00717	-1.18659	2.995817	0.709334	0.614991	0.555718	1.246892
361.1144	25.71106	10.36113	-0.73295	1.592765	0.783442	0.328984	0.589	1.314892
320.695	20.81692	5.99328	-0.11779	1.306731	0.872943	0.19602	0.661353	1.368439
732.7328	29.22919	45.92853	-6.6756	2.985813	1.218922	0.392303	0.707432	1.500793
1682.806	51.5846	181.3848	-3.96961	7.191619	1.881524	0.556148	0.877893	1.685371
855.6357	43.10403	124.6114	-3.12023	3.462695	1.325606	0.415327	0.676744	1.457461
208.7099	42.38459	5.233244	-0.51471	1.086112	0.455251	0.404754	0.408501	1.053598
889.1083	64.68482	106.8215	-11.4376	3.560915	1.180119	0.467252	0.699854	1.486359
312.6146	34.77981	13.23154	-0.74369	1.198704	0.565158	0.33474	0.4505	1.164233
1150.362	43.18729	101.866	-9.79053	4.075833	1.857425	0.337553	0.919443	1.717395
2396.833	71.23409	532.5637	4.388723	11.556	2.305697	0.655479	1.069385	1.822278
847.3457	36.31001	54.04959	-4.06311	3.373392	1.351005	0.419051	0.78859	1.557676
256.6317	15.23297	7.642463	-0.42177	1.089995	0.414301	0.428972	0.344272	1.024252
1076.38	63.25823	113.076	-3.33032	4.696107	1.105506	0.59821	0.685751	1.434182
818.0998	51.46572	51.54613	-4.20048	2.957306	1.51528	0.299516	0.846089	1.643237
286.5206	41.34959	28.38168	-0.08626	1.738593	0.485	0.563889	0.403192	1.072527
771.2443	39.05487	44.17312	-0.63132	2.903195	1.25715	0.361542	0.681147	1.450935
379.9384	1289.926	428.0709	-7.16115	1.549785	0.535129	0.474015	0.443224	1.131957
538.9687	35.65862	25.54706	-2.28914	2.053297	1.057161	0.294797	0.690584	1.455608
930.9465	43.88486	53.82261	-0.92665	3.866775	1.349249	0.463649	0.817448	1.568761
585.4721	17.98306	13.48794	-0.32766	2.241777	1.316473	0.241931	0.820894	1.498423
450.4156	47.99828	25.88635	1.062298	1.702453	1.037087	0.234838	0.713426	1.452294
554.6526	33.89408	25.67516	-0.93736	2.163428	1.149281	0.291518	0.741591	1.508696
784.687	53.45137	53.56324	-2.29604	2.844383	1.521616	0.258167	0.876319	1.625472
809.9416	42.65437	1151.854	27.15145	3.610737	0.766872	0.62206	0.522079	1.220803
671.8018	34.73091	34.70234	-2.99696	2.681906	1.261143	0.344531	0.74753	1.547182

640.3996	47.77541	35.04007	-3.61516	2.613067	1.223696	0.341674	0.707532	1.497472
11681.69	53.28193	83334.96	1285.429	24.24861	1.065551	0.374645	0.616079	1.415274
738.685	50.61056	34.16346	-2.86946	2.744494	1.496694	0.273248	0.859842	1.645439
439.1249	31.03566	18.44851	-1.40767	1.944256	0.690957	0.459752	0.528864	1.242952
480.9397	65.16505	40.3469	-0.6467	1.885192	1.007393	0.287369	0.679087	1.437157
473.0898	32.84914	14.0044	-1.86694	1.745254	1.017686	0.252017	0.665115	1.454929
503.8524	43.96639	28.49564	-1.2039	2.168018	0.751104	0.464098	0.531315	1.279762
958.2541	37.90425	72.37957	-3.426	3.677622	1.381329	0.425599	0.802457	1.602014
1096.714	74.72914	4911.446	111.1083	5.599757	1.783387	0.517798	0.714289	1.48596
666.8078	43.3389	47.79503	-6.61464	2.457902	1.099982	0.353579	0.657383	1.470061
796.1917	44.87372	46.58402	-5.15906	3.049635	1.490058	0.322937	0.819851	1.623192
210.2606	154.6597	10.85869	-1.58398	1.105227	0.504721	0.36392	0.433522	1.090698
537.2452	50.25052	27.55152	-1.83891	2.58075	0.775625	0.524259	0.561082	1.287742
633.4556	38.99646	25.81361	0.761429	3.128917	0.995078	0.512845	0.659005	1.417514
711.0652	39.73543	42.3601	-3.68637	2.868722	1.340926	0.349616	0.759084	1.532999
730.669	48.19471	38.26335	-3.91965	2.786922	1.171079	0.377997	0.712575	1.494206
460.4593	37.93639	18.51179	-1.03513	1.911103	0.827015	0.374596	0.573612	1.32779
502.6355	31.83272	16.40319	-0.83271	2.092807	1.063227	0.319345	0.721431	1.47551
338.462	24.40183	8.222033	-0.63787	1.051492	0.569703	0.213776	0.455116	1.175317
536.1396	34.74184	30.70575	-3.48526	2.267729	0.955481	0.387378	0.621676	1.384985
572.4188	31.92491	39.46714	-4.97966	3.268264	0.558163	0.707608	0.446586	1.149938
791.5761	31.8971	38.01952	-5.30053	2.897115	1.039051	0.422663	0.722651	1.425077
214.794	21.07379	4.083716	-0.299	1.173546	0.488944	0.410809	0.441451	1.092384
679.2823	48.06927	40.54777	0.61105	3.300611	0.787275	0.604625	0.580701	1.307877
2594.337	32.47445	263.7472	3.183224	10.07399	4.111712	0.398861	1.625585	1.933992
863.3682	41.81183	104.9331	-0.42408	3.900399	1.108752	0.541138	0.69634	1.459155
597.5633	48.27281	48.6434	3.89043	2.874049	0.760789	0.567056	0.545366	1.254
567.4199	20.23288	25.25389	0.57345	3.078095	0.778771	0.594463	0.603414	1.297726
432.5099	42.97172	18.80613	0.08362	1.731312	0.92801	0.289496	0.660324	1.396388
1382.513	41.28225	106.5573	-9.97545	5.58264	2.176824	0.412254	1.052002	1.738814
759.9258	54.22744	54.03965	-5.38819	2.667049	1.442362	0.275876	0.776559	1.598003
1050.786	38.0944	83.55321	-7.11783	4.181768	1.171233	0.538425	0.682935	1.489472
1087.463	68.28756	100.827	-3.94911	3.82477	1.870837	0.324203	0.907598	1.729435
1103.446	51.38712	64.98786	-4.34273	4.544924	1.634899	0.45976	0.79794	1.647752
385.6863	10.47763	4.671463	-0.10178	1.208249	1.570996	-0.1173	0.997456	1.328857
211.8163	6.977123	10.58152	0.830991	1.062253	0.477245	0.368631	0.421299	1.060439
455.6128	6.941012	13.15002	1.92696	1.961086	0.922921	0.348613	0.67084	1.335022
299.6914	1898.054	758.9457	-8.98437	1.029996	0.488263	0.325384	0.436517	1.079465
271.4361	51.1275	8.479767	-0.5611	1.408499	0.517178	0.458684	0.442752	1.1179
176.1182	33.2498	2.953333	-0.25717	0.88388	0.380184	0.395105	0.351999	0.995387
369.3059	24.9446	11.21604	0.90637	2.097764	0.49178	0.616855	0.43487	1.087404
212.8406	11.18757	4.510548	0.827282	0.939606	0.540608	0.259075	0.455023	1.151208
404.1215	44.32131	13.76402	-2.01373	1.42034	0.733105	0.293077	0.540585	1.30012
147.8718	17.46194	2.267361	0.029397	0.66039	0.401765	0.230962	0.358089	1.013589
397.6066	70.18248	26.48997	-2.62965	1.470509	0.704094	0.33304	0.513338	1.249506
304.3909	58.24923	9.706419	-0.96445	1.493974	0.666063	0.380415	0.55527	1.238391
513.537	17.04674	19.81383	0.457211	2.6534	0.766922	0.544983	0.558582	1.253043
337.2092	16.70874	9.866345	-0.97233	1.291638	0.697272	0.283007	0.515057	1.262515
208.0685	5.906741	3.843692	0.390839	1.083586	0.493723	0.370962	0.442606	1.097201
388.2259	22.66026	10.8495	0.85863	1.481935	0.803227	0.276784	0.574602	1.316789
425.5248	293.0442	79.81453	-1.43982	1.942565	0.616646	0.498254	0.492703	1.201024
1155.221	53.50096	86.54207	-1.46906	4.773322	1.325066	0.547279	0.811599	1.512786
994.2467	67.10087	4595.045	102.4692	4.741949	1.705998	0.469847	0.678138	1.441067
1047.03	63.62042	145.0208	-14.1599	3.786626	2.231637	0.247418	0.690619	1.488705
465.9184	50.09809	23.64062	-2.43036	1.654816	0.981826	0.225806	0.662351	1.399208
353.2866	46.334	11.77423	-1.29976	1.193241	0.678882	0.232672	0.472931	1.241224

227.9835	47.40976	19.61508	0.457331	1.064757	0.325937	0.522949	0.268364	0.901899
586.9718	63.75663	40.89401	-1.18503	3.01596	0.645806	0.640394	0.501641	1.223579
313.9049	40.62548	11.14338	1.780609	1.685646	0.387855	0.622081	0.353856	1.003822
3518.248	55.97317	90864.9	1201.394	20.03889	4.61852	0.634522	0.58748	1.274676
117.2864	12.09746	2.22132	0.309086	0.744053	0.455246	0.238392	0.432963	1.036249
233.2142	22.40408	4.275724	-0.08626	1.078348	0.606867	0.273	0.513371	1.185171
337.257	31.24829	15.62438	0.880632	1.420289	0.646884	0.3529	0.504994	1.211414
303.2	1889.061	791.0375	-8.58308	0.986827	0.459739	0.329486	0.424894	1.055859
607.1183	13.21771	19.7483	2.968912	2.328615	1.125606	0.300616	0.693434	1.446226
148.1302	6.355184	1.608938	0.059902	0.759554	0.49594	0.20724	0.465961	1.074102
288.2444	39.25226	11.26711	-1.0008	1.252567	0.590471	0.338127	0.475842	1.166458
178.9856	19.34559	2.769051	0.330726	0.896317	0.48593	0.279754	0.43763	1.052427
540.269	18.37782	14.69357	1.226389	1.943889	1.262284	0.189953	0.756079	1.45897
141.7265	9.975721	2.008615	-0.02125	0.665221	0.429459	0.211588	0.390372	1.047874
205.0532	18.83741	8.571168	1.239925	0.919296	0.451498	0.310271	0.394591	1.058178
361.271	35.43301	15.70981	-0.62443	1.605629	0.765973	0.342925	0.5676	1.29536
298.306	15.39383	17.19449	1.632607	1.169091	0.585414	0.312537	0.429363	1.165104
270.5746	55.27448	10.41796	-1.46062	1.559258	0.408026	0.584773	0.365656	1.019682
402.3715	57.68925	20.73078	-3.00538	1.582003	0.76427	0.317161	0.530127	1.277183
275.3068	27.37207	10.00384	-1.01971	1.113416	0.630457	0.260858	0.483602	1.20135
275.347	35.11569	10.52305	0.313023	1.106325	0.599777	0.285773	0.466139	1.20281
1179.754	246.9346	169.6206	-9.89802	6.207415	1.181501	0.676104	0.720696	1.502995
179.2227	22.02553	4.681861	-0.7605	0.852067	0.468912	0.274539	0.41812	1.079187
2014.641	49.15489	198.7279	-17.3315	5.840924	2.017243	0.367397	1.083302	1.683562
289.8636	35.75994	9.564121	-0.30308	1.055677	0.713732	0.181176	0.549414	1.281432
230.7704	49.20866	8.187663	-0.93022	0.977176	0.488733	0.317432	0.409944	1.105889
541.2061	64.2412	141.927	-13.4453	2.289174	0.643332	0.53975	0.444332	1.183739
4433.833	62.0947	1418.251	-110.934	24.8332	2.321478	0.825997	0.969406	1.799645
95349.26	260.5712	1312460	17138.32	453.1428	188.1101	0.417409	8.22621	4.198024
599.4401	41.14958	28.25487	-2.37229	2.317891	1.125047	0.331168	0.713787	1.47316
1941.885	59.40801	689.3864	-65.0158	8.661973	1.422563	0.696057	0.576945	1.396963
798.7571	68.57557	86.83289	-4.2895	2.23408	0.810799	0.319751	0.559914	1.324363

DX41	DX42	DX43	DX44	DX45	DX46	DX47	DX48	DX49
0.370899	0.74517	0.736421	0.993119	0.950492	-0.10801	0.41485	0.437811	6.37777
0.332748	0.779927	0.773368	0.984642	0.934009	-0.14455	0.424856	0.386235	3.550987
0.258921	0.797222	0.79577	0.998496	0.977119	-0.09212	0.386954	0.393698	4.534909
0.241873	0.840774	0.839051	0.986632	0.946104	-0.09135	0.334884	0.304449	3.009199
0.722782	0.719196	0.70516	0.98854	0.941074	-0.13743	0.497047	0.43476	7.146171
0.389406	0.755294	0.746697	0.989441	0.941815	-0.13602	0.492263	0.417898	4.606902
0.474397	0.700914	0.686618	0.999569	0.98593	-0.09101	0.449406	0.479465	43.30665
0.411778	0.717384	0.706112	0.990076	0.938977	-0.15262	0.601654	0.472117	5.372948
0.390953	0.736199	0.727214	0.985559	0.929886	-0.17012	0.633231	0.453145	3.973679
0.489868	0.725615	0.714316	0.999715	0.989151	-0.09087	0.416705	0.45302	44.59653
0.34868	0.760135	0.754094	0.996351	0.963812	-0.17453	0.642045	0.429746	7.3657
0.300626	0.787213	0.783197	0.995815	0.963173	-0.22583	0.704033	0.392627	5.382327
0.361804	0.760886	0.754025	0.993687	0.953892	-0.18325	0.639466	0.421305	5.34608
0.418061	0.718596	0.706393	0.987368	0.932333	-0.13638	0.547223	0.462267	5.022097
0.394479	0.737696	0.728851	0.988813	0.937792	-0.12645	0.486601	0.451033	4.723495
0.295755	0.799002	0.795385	0.994184	0.958411	-0.15338	0.556919	0.372323	5.942809
0.288219	0.785904	0.782491	0.986748	0.937281	-0.11509	0.449047	0.400379	4.032814
0.419499	0.739478	0.730584	0.998093	0.973005	-0.23014	0.743492	0.446661	6.311639
0.534329	0.736727	0.724992	0.986482	0.935385	-0.15746	0.583247	0.425404	5.969241
0.390191	0.750097	0.742363	0.995225	0.958835	-0.23799	0.756319	0.435281	6.983887
0.353199	0.752514	0.745781	0.993623	0.952493	-0.26804	0.796656	0.439411	5.61664
0.581548	0.703951	0.688898	0.998396	0.974687	-0.17891	0.687264	0.463109	6.803687
0.416706	0.762252	0.753591	0.989339	0.943119	-0.13934	0.509516	0.402619	5.519765
0.373981	0.760722	0.753155	0.99794	0.972906	-0.20782	0.655662	0.415901	5.755205
0.365593	0.783543	0.777756	0.995905	0.964518	-0.16257	0.565584	0.383975	7.01475
0.350044	0.78264	0.778727	0.993196	0.954593	-0.07994	0.329463	0.400892	4.250339
0.231301	0.86986	0.868623	0.999877	0.994731	-0.11074	0.380815	0.249596	43.04396
0.466836	0.717204	0.701968	0.994499	0.954616	-0.20054	0.665127	0.438899	5.2913
0.40323	0.750254	0.741439	0.994141	0.955119	-0.13547	0.523679	0.426049	6.34835
0.626435	0.708749	0.693971	0.986881	0.934178	-0.1796	0.661175	0.453744	5.776456
0.292931	0.762324	0.75908	0.985558	0.930622	-0.07009	0.353746	0.448915	3.417896
0.347717	0.75729	0.750509	0.99039	0.943269	-0.18055	0.646833	0.429751	4.81803
0.351507	0.753859	0.747573	0.993786	0.952945	-0.0857	0.427973	0.440566	5.868912
0.388422	0.757304	0.748238	0.993119	0.952402	-0.24312	0.733635	0.410203	6.650864
0.321828	0.799537	0.79695	0.999839	0.992594	-0.2273	0.709058	0.379647	4.377576
0.264572	0.808033	0.806293	0.993528	0.956609	-0.07048	0.325379	0.369605	4.640994
0.350671	0.76793	0.761327	0.998453	0.976393	-0.31453	0.841495	0.409601	4.901522
0.329359	0.749581	0.743834	0.987717	0.934885	-0.08832	0.386644	0.453748	3.646742
0.32476	0.769818	0.766384	0.996142	0.963269	-0.06121	0.30329	0.43121	6.427022
0.495293	0.756566	0.745687	0.993354	0.954837	-0.1957	0.654303	0.393805	6.346148
0.492424	0.736318	0.725162	0.994068	0.955059	-0.13836	0.53258	0.432249	7.419727
0.416686	0.755682	0.747196	0.998644	0.977787	-0.28999	0.829242	0.417716	7.518392
0.576301	0.68227	0.661448	0.990184	0.938817	-0.19451	0.684632	0.459734	6.973218
0.357603	0.785037	0.779938	0.999258	0.984209	-0.21057	0.666093	0.387202	6.269147
0.289898	0.801033	0.798675	0.998727	0.979526	-0.16632	0.598748	0.378377	6.96986
0.272619	0.803583	0.800928	0.98315	0.933256	-0.1044	0.404932	0.371237	3.096485
0.282734	0.795573	0.792429	0.987382	0.940153	-0.11698	0.446089	0.383194	4.00802
0.488452	0.721725	0.705499	0.993453	0.951558	-0.21905	0.703829	0.421547	5.0504
0.333718	0.763418	0.757748	0.9965	0.964492	-0.2135	0.709361	0.426685	4.364196
0.36417	0.797132	0.792756	0.998086	0.976117	-0.27041	0.76383	0.368523	4.364211
0.31898	0.780915	0.776597	0.993477	0.954444	-0.09038	0.382976	0.402437	6.211441
0.472394	0.773454	0.767586	0.997271	0.971241	-0.07788	0.312371	0.40209	3.712044
0.438632	0.743715	0.734541	0.999646	0.988172	-0.08544	0.365162	0.434965	44.0345
0.408365	0.773893	0.765911	0.998614	0.978504	-0.14236	0.518046	0.385332	5.904018
0.590779	0.758423	0.749127	0.998762	0.980449	-0.17685	0.632431	0.40179	7.247484
0.825252	0.752642	0.739488	0.996937	0.971313	-0.19452	0.632582	0.372947	8.820641
0.34688	0.773582	0.766914	0.988266	0.94045	-0.14457	0.550953	0.398088	4.827115

0.353062	0.769751	0.763995	0.98827	0.939756	-0.0712	0.333979	0.412734	3.891968
0.479634	0.725883	0.712546	0.992684	0.949183	-0.16806	0.604205	0.436277	6.23463
0.468325	0.742919	0.731647	0.994296	0.956208	-0.13011	0.470386	0.41886	6.043095
0.329861	0.77181	0.766004	0.997408	0.969862	-0.26438	0.771703	0.408604	7.237892
0.48685	0.712042	0.696477	0.985502	0.928999	-0.13783	0.498349	0.445992	4.405531
0.372517	0.802428	0.796577	0.991525	0.953244	-0.1077	0.41018	0.345503	5.810938
0.312239	0.798711	0.794493	0.993962	0.958064	-0.15602	0.567973	0.367753	6.666114
0.379032	0.747214	0.739405	0.995958	0.961407	-0.21091	0.722275	0.440983	4.993832
0.348839	0.751274	0.74524	0.990276	0.942105	-0.07907	0.327114	0.447433	4.489795
0.409392	0.718205	0.70605	0.98386	0.924189	-0.09039	0.398162	0.46382	3.990014
0.3103	0.779231	0.774795	0.991862	0.949156	-0.13801	0.537209	0.405283	4.3728
0.396327	0.728519	0.718668	0.990838	0.941869	-0.17262	0.672596	0.461976	5.203743
0.326448	0.755173	0.7499	0.99409	0.953621	-0.11917	0.52748	0.446539	5.473047
0.329741	0.765624	0.759566	0.990945	0.945475	-0.28762	0.807516	0.419226	3.568916
0.584884	0.779654	0.774534	0.999352	0.986649	-0.25606	0.771132	0.395679	5.930758
0.378148	0.818586	0.816073	0.99991	0.995058	-0.16703	0.57596	0.341491	43.83197
0.320738	0.806015	0.800377	0.971694	0.919451	-0.10177	0.329923	0.341732	2.708308
0.356595	0.750164	0.742621	0.986941	0.934183	-0.19585	0.671316	0.437648	4.594122
0	1	1	1	1	0	0	0	1
0.587302	0.724309	0.711847	0.990242	0.943763	-0.14156	0.538691	0.442406	6.367723
0.444195	0.741511	0.732524	0.998048	0.97308	-0.15347	0.595113	0.441248	5.431292
0.2735	0.794042	0.791532	0.987636	0.940018	-0.10336	0.408223	0.391466	3.399424
0.706763	0.623761	0.594337	0.996037	0.956564	-0.1829	0.784265	0.503893	7.083604
0.398748	0.772592	0.764022	0.99456	0.958937	-0.24943	0.748382	0.383429	5.594683
0.465237	0.69014	0.675646	0.994415	0.951181	-0.04814	0.363351	0.500068	7.040947
0.328826	0.77991	0.775966	0.99832	0.975812	-0.15484	0.589345	0.40739	5.079562
0.299186	0.78328	0.77949	0.992177	0.950311	-0.12949	0.515593	0.402453	4.864474
0.355715	0.747497	0.740669	0.990141	0.941128	-0.05412	0.353402	0.449149	4.825575
0.434387	0.705026	0.693166	0.997611	0.967714	-0.06387	0.409591	0.492142	5.954716
0.453233	0.757601	0.748322	0.990918	0.947206	-0.13561	0.527433	0.405784	5.786892
0.347246	0.748345	0.741906	0.992263	0.947225	-0.07154	0.35641	0.450604	6.772287
0.460432	0.744363	0.733232	0.992099	0.948959	-0.1339	0.518387	0.417744	6.674357
0.374425	0.754787	0.747481	0.995435	0.959806	-0.18184	0.660421	0.429908	6.928298
0.732644	0.777456	0.769797	0.997631	0.976004	-0.20231	0.617886	0.37627	3.898872
0.421423	0.733628	0.722587	0.990399	0.942193	-0.16594	0.605924	0.441058	6.136342
0.63391	0.689504	0.666506	0.989378	0.938677	-0.23171	0.715976	0.42481	6.385138
0.423728	0.754973	0.74862	0.996453	0.965053	-0.08752	0.437526	0.43574	5.946296
0.290713	0.803573	0.800302	0.997252	0.970915	-0.1432	0.515798	0.365858	5.042742
0.380533	0.733025	0.723326	0.990924	0.942765	-0.11373	0.404097	0.453975	6.503549
0.360583	0.732235	0.723247	0.980961	0.919332	-0.11494	0.45855	0.46201	3.449379
0.489706	0.704219	0.688707	0.988243	0.934307	-0.17815	0.664615	0.46211	5.447621
0.427529	0.740723	0.729771	0.990407	0.943475	-0.12998	0.425368	0.42694	5.747054
0.342324	0.749563	0.741937	0.99001	0.941304	-0.18797	0.630648	0.438636	3.618804
0.340696	0.761916	0.75583	0.992618	0.950009	-0.13904	0.547669	0.426109	6.171955
0.449412	0.751143	0.743342	0.99847	0.976509	-0.14674	0.599794	0.432073	5.518969
0.396188	0.752045	0.74333	0.989179	0.940958	-0.12201	0.418628	0.42308	5.144212
0.517223	0.737066	0.726947	0.996631	0.965727	-0.08717	0.413814	0.439173	6.949551
0.467728	0.763377	0.757363	0.991198	0.949055	-0.08077	0.312761	0.418723	6.395032
0.31421	0.774664	0.770621	0.98918	0.941751	-0.08479	0.325899	0.417028	4.296448
0.322616	0.779001	0.774088	0.989201	0.942591	-0.08631	0.392844	0.401687	4.776672
0.359093	0.766371	0.76069	0.99787	0.972502	-0.17303	0.619594	0.419988	5.92847
0.622946	0.764346	0.757497	0.999537	0.988264	-0.19355	0.646885	0.41323	4.841511
0.405564	0.769074	0.763089	0.99916	0.982897	-0.16729	0.622277	0.411424	6.781451
0.284511	0.79411	0.790905	0.987301	0.939724	-0.08514	0.342449	0.38568	3.098431
0.319915	0.772377	0.767088	0.988772	0.940758	-0.12945	0.462222	0.411821	3.619053
0.292107	0.789703	0.786585	0.999826	0.991925	-0.23549	0.730365	0.395082	42.86206
0.386887	0.746028	0.738085	0.994277	0.954769	-0.12189	0.480542	0.441909	5.572244
0.309065	0.778732	0.774405	0.994754	0.958247	-0.0636	0.295599	0.407184	7.118122

0.511912	0.67135	0.653478	0.993663	0.947325	-0.09029	0.540255	0.509258	5.483107
0.403292	0.720425	0.708277	0.983932	0.924733	-0.12234	0.46759	0.459382	3.387342
0.603647	0.761511	0.754283	0.999324	0.98563	-0.10048	0.460383	0.413306	7.092302
0.421936	0.778098	0.771108	0.989875	0.946962	-0.1134	0.345556	0.383251	5.685333
0.377591	0.718788	0.709974	0.988586	0.93389	-0.03583	0.248949	0.490123	5.203491
0.367639	0.777462	0.770945	0.999842	0.992467	-0.24261	0.708115	0.390751	7.126896
0.40081	0.726695	0.714229	0.98772	0.934177	-0.13978	0.489521	0.44446	3.8745
0.296113	0.789545	0.786541	0.99251	0.951942	-0.09777	0.439253	0.396059	5.657608
0.322711	0.767853	0.762808	0.988774	0.939795	-0.07276	0.351393	0.423075	4.177496
0.293568	0.784337	0.780377	0.989833	0.94437	-0.12078	0.409069	0.398924	4.382048
0.365332	0.753779	0.745362	0.989689	0.941748	-0.16224	0.555277	0.42312	4.534958
0.640321	0.694401	0.676468	0.98235	0.922691	-0.09224	0.384268	0.456488	5.572837
0.42899	0.7602	0.752187	0.996797	0.967408	-0.24332	0.735253	0.411735	5.250768
0.301655	0.817563	0.813602	0.996811	0.970546	-0.13948	0.436711	0.331857	6.76244
0.428395	0.751258	0.743305	0.997972	0.972958	-0.14179	0.57882	0.43014	7.21462
0.505054	0.734499	0.721755	0.992641	0.950503	-0.19167	0.655357	0.42254	8.874962
0.435807	0.714634	0.703551	0.993146	0.94839	-0.16192	0.664332	0.478582	6.318075
0.391391	0.795343	0.791897	0.99936	0.986068	-0.25704	0.750912	0.379753	5.147806
0.287251	0.787505	0.783421	0.986477	0.937418	-0.13715	0.419264	0.391659	3.267494
0.354238	0.7682	0.761687	0.99052	0.945588	-0.13509	0.445602	0.409314	5.250348
0.433345	0.705406	0.689951	0.981724	0.919325	-0.13296	0.428018	0.461597	4.431582
0.421274	0.767275	0.758704	0.993158	0.95413	-0.09447	0.355992	0.393248	6.927329
0.496158	0.749087	0.737491	0.991629	0.949188	-0.17769	0.581629	0.402576	6.004106
0.267958	0.820038	0.817791	0.989136	0.947544	-0.03725	0.224188	0.34155	4.074283
0.403543	0.747259	0.737103	0.993894	0.954198	-0.19139	0.632185	0.421188	5.962797
0.48132	0.747525	0.73457	0.989822	0.943841	-0.25142	0.756618	0.396037	4.450843
0.510838	0.698546	0.680735	0.98439	0.925089	-0.16	0.615997	0.454599	5.472475
0.329953	0.775754	0.771904	0.999735	0.989973	-0.08422	0.394963	0.416003	43.54072
0.383264	0.746087	0.73783	0.991559	0.945897	-0.10162	0.439183	0.439537	5.927822
0.896639	0.647808	0.622077	0.992378	0.945902	-0.09127	0.406167	0.477679	8.411193
1.117399	0.594134	0.555804	0.980217	0.912272	-0.13091	0.575205	0.466559	7.11616
0.516572	0.72503	0.710312	0.98848	0.938364	-0.16994	0.55513	0.425365	5.395745
0.377116	0.745535	0.737377	0.995167	0.958076	-0.21259	0.707042	0.441283	6.819939
0.423981	0.734077	0.724686	0.990693	0.942794	-0.08695	0.414943	0.453222	5.050616
0.427771	0.702664	0.690448	0.986913	0.92882	-0.03485	0.252306	0.49415	4.550655
0.65599	0.716193	0.696486	0.982423	0.926689	-0.16487	0.571463	0.39809	5.365191
0.976485	0.680842	0.65399	0.991974	0.948546	-0.23037	0.718949	0.396348	7.089206
0.812317	0.728962	0.715416	0.997349	0.971644	-0.16456	0.562261	0.417536	6.52451
0.280055	0.802666	0.800327	0.99309	0.955107	-0.12326	0.476223	0.37518	6.498205
0.652519	0.711005	0.695476	0.990738	0.944518	-0.13792	0.525836	0.441779	8.005439
0.349605	0.791787	0.786073	0.997519	0.972254	-0.10768	0.396842	0.368749	5.883981
0.941869	0.652442	0.626442	0.982928	0.922794	-0.11113	0.470381	0.462555	6.529335
1.028301	0.613977	0.578194	0.995342	0.955614	-0.21988	0.781607	0.454965	8.301833
0.675831	0.680064	0.659534	0.984466	0.925859	-0.15158	0.606198	0.462643	5.983644
0.287883	0.838817	0.834863	0.991905	0.957915	-0.16186	0.509964	0.289753	3.881234
0.580345	0.712456	0.697271	0.997277	0.968215	-0.23406	0.740933	0.443869	7.896871
0.776208	0.660871	0.638924	0.989999	0.9383	-0.0764	0.427282	0.486913	7.14879
0.314289	0.808484	0.805447	0.998525	0.978978	-0.20036	0.641768	0.35734	6.405948
0.720942	0.725572	0.709118	0.988601	0.941877	-0.18128	0.56299	0.404614	6.216374
0.329359	0.79134	0.787082	0.999724	0.990194	-0.13902	0.529567	0.381721	35.91201
0.555837	0.704807	0.690016	0.993924	0.952329	-0.08589	0.385465	0.464268	5.950567
0.631987	0.665669	0.643155	0.989301	0.935118	-0.13417	0.562054	0.477499	6.576825
0.568335	0.659914	0.637982	0.975428	0.903834	-0.12828	0.505419	0.494879	4.213269
0.51933	0.689061	0.673877	0.996029	0.959046	-0.04127	0.287592	0.495128	6.916058
0.582238	0.685593	0.66852	0.992284	0.945079	-0.06939	0.381713	0.483882	5.800044
0.692639	0.650925	0.624668	0.987979	0.930888	-0.10175	0.423287	0.474165	7.288077
0.470881	0.763477	0.75648	0.999531	0.987394	-0.2348	0.725587	0.413652	6.476319
0.671462	0.694221	0.675778	0.985457	0.929566	-0.10805	0.496573	0.451747	5.863064

0.680599	0.710562	0.694645	0.988545	0.939241	-0.12781	0.504899	0.438438	6.886761
0.66773	0.741461	0.728544	0.999561	0.987844	-0.14189	0.43275	0.40508	6.882026
0.728929	0.654881	0.631134	0.985889	0.926322	-0.07485	0.401377	0.485758	7.092068
0.392076	0.759012	0.75152	0.991788	0.948518	-0.15141	0.55179	0.418963	5.542764
0.536169	0.704427	0.690959	0.997257	0.966789	-0.06087	0.351893	0.477231	8.058839
0.557485	0.716663	0.701927	0.988098	0.936507	-0.07253	0.358337	0.441311	5.715504
0.44033	0.765585	0.755865	0.995657	0.963054	-0.16738	0.557401	0.386702	6.603926
0.703943	0.675622	0.654401	0.993166	0.948784	-0.11664	0.523554	0.465526	6.109838
1.258809	0.711542	0.699293	0.999471	0.987956	-0.09941	0.515753	0.464876	8.589239
0.639741	0.728181	0.713422	0.987363	0.937942	-0.10184	0.393986	0.414648	6.557301
0.7729	0.67533	0.653645	0.98606	0.930128	-0.10236	0.457438	0.45906	6.66956
0.309085	0.792841	0.790091	0.997783	0.973154	-0.10481	0.424593	0.390741	12.43018
0.430714	0.750065	0.740374	0.997015	0.967672	-0.18455	0.621063	0.418231	7.056844
0.537029	0.715387	0.702545	0.993327	0.951041	-0.17068	0.660691	0.459099	6.201823
0.724787	0.691288	0.674086	0.987518	0.934971	-0.1293	0.554536	0.465069	6.273058
0.618511	0.705237	0.688033	0.990743	0.943377	-0.12648	0.477111	0.441325	6.913079
0.478867	0.747318	0.737645	0.994432	0.957189	-0.12494	0.499931	0.42225	6.137192
0.528805	0.689173	0.672862	0.989773	0.936898	-0.06786	0.398611	0.484988	5.619192
0.350287	0.790797	0.783897	0.988899	0.944677	-0.10587	0.329964	0.361843	4.927496
0.530424	0.735199	0.722057	0.990863	0.945872	-0.1349	0.486678	0.41682	5.866323
0.339253	0.794229	0.787837	0.99331	0.956336	-0.28143	0.775686	0.358907	5.58989
0.48306	0.687531	0.670238	0.980116	0.913932	-0.16273	0.626934	0.481877	5.606046
0.287798	0.786971	0.78402	0.990339	0.945471	-0.11119	0.481032	0.401936	4.571926
0.429426	0.73947	0.729656	0.996058	0.962115	-0.20739	0.709219	0.438949	6.887644
1.032409	0.495564	0.425389	0.962314	0.869434	-0.25863	0.856193	0.378115	5.566191
0.591789	0.704735	0.689582	0.996993	0.966107	-0.16566	0.637101	0.460867	6.412013
0.42979	0.755751	0.74705	0.997093	0.968581	-0.20828	0.657174	0.414929	6.909698
0.395368	0.72587	0.715774	0.992412	0.946449	-0.1864	0.683645	0.465172	4.43351
0.481102	0.707801	0.695669	0.995365	0.957047	-0.06436	0.357801	0.482403	6.539945
0.882831	0.618359	0.581783	0.975401	0.904162	-0.16424	0.558906	0.442058	6.357691
0.81977	0.688818	0.671175	0.990584	0.943552	-0.07264	0.40651	0.464345	7.343095
0.653991	0.722063	0.705245	0.988966	0.941335	-0.19412	0.65135	0.409472	6.110678
0.98509	0.656167	0.630538	0.994428	0.95456	-0.09685	0.426243	0.459462	8.23401
0.943137	0.696303	0.67578	0.987532	0.938274	-0.17586	0.676819	0.421029	7.117517
0.403311	0.594576	0.558626	0.944449	0.84725	-0.2523	0.756981	0.517198	3.250719
0.28988	0.796903	0.794496	0.997219	0.970157	-0.11513	0.438128	0.385974	2.61355
0.422424	0.704502	0.689788	0.966791	0.894046	-0.14971	0.55418	0.470019	2.584503
0.287159	0.789679	0.786875	0.999788	0.991112	-0.09356	0.372742	0.397445	43.56511
0.309041	0.790334	0.786046	0.997389	0.970789	-0.14654	0.534829	0.384044	7.134745
0.253443	0.828457	0.826804	0.995393	0.965052	-0.11625	0.468343	0.32951	5.755334
0.290187	0.791711	0.788238	0.994059	0.957024	-0.22711	0.699526	0.388146	4.954055
0.326378	0.78593	0.781042	0.989419	0.944283	-0.07149	0.311437	0.38787	3.329819
0.433204	0.757321	0.74866	0.992915	0.952248	-0.07632	0.362573	0.41227	6.644459
0.266861	0.827917	0.825323	0.989343	0.949609	-0.08476	0.285196	0.322877	4.171005
0.430524	0.767599	0.761036	0.995271	0.96148	-0.09006	0.393029	0.407971	8.366044
0.352556	0.740312	0.733436	0.993475	0.950353	-0.08218	0.447404	0.46316	7.618547
0.405294	0.751957	0.741082	0.993839	0.954743	-0.21137	0.632977	0.405911	4.070924
0.416206	0.7689	0.760513	0.986658	0.938013	-0.0921	0.36045	0.391567	4.069415
0.290503	0.787069	0.783809	0.981437	0.927439	-0.10045	0.437644	0.399272	2.399661
0.454891	0.744074	0.734713	0.992289	0.949389	-0.10531	0.448367	0.432201	4.742403
0.363686	0.771395	0.7654	0.998736	0.978795	-0.1513	0.578843	0.407306	17.10885
0.593575	0.665404	0.642963	0.99344	0.947926	-0.19026	0.678451	0.4802	7.255254
1.231961	0.720636	0.709117	0.999339	0.986766	-0.08608	0.446593	0.455722	8.145034
1.657135	0.784029	0.7689	0.984687	0.950337	-0.24503	0.672689	0.271092	7.951196
0.514478	0.712194	0.699206	0.9922	0.946725	-0.09095	0.390318	0.465176	7.066088
0.434809	0.793972	0.784023	0.993447	0.958511	-0.11837	0.379308	0.328693	6.797394

0.252615	0.873308	0.871265	0.998573	0.983427	-0.19174	0.577747	0.235706	6.872046
0.37895	0.770334	0.76329	0.99561	0.962176	-0.22842	0.732462	0.400494	7.947584
0.258454	0.828544	0.826463	0.996182	0.968086	-0.23905	0.6995	0.325882	6.348283
4.246348	0.804092	0.79807	0.99918	0.992316	-0.12076	0.483482	0.326733	7.219274
0.261839	0.78709	0.785745	0.993031	0.952137	-0.06294	0.309054	0.414795	3.467743
0.332149	0.758213	0.75264	0.988107	0.937104	-0.07221	0.326595	0.437855	4.720823
0.372649	0.768207	0.761391	0.996241	0.964581	-0.12046	0.420439	0.406551	5.572677
0.273632	0.792909	0.790962	0.99983	0.991995	-0.08075	0.359856	0.398141	43.46181
0.56672	0.717034	0.696294	0.978855	0.919042	-0.15531	0.459282	0.392012	3.593012
0.273947	0.772001	0.770017	0.981166	0.923052	-0.04386	0.259847	0.43987	2.507841
0.347217	0.778782	0.773288	0.992952	0.95341	-0.11481	0.406536	0.396451	6.251923
0.273359	0.789161	0.786015	0.990394	0.945965	-0.11405	0.326689	0.396065	4.386565
0.572389	0.69778	0.672491	0.981512	0.921407	-0.17221	0.506375	0.393245	4.266749
0.273205	0.81114	0.808723	0.983819	0.935855	-0.05235	0.26181	0.357954	3.149081
0.285702	0.811558	0.808339	0.998011	0.975544	-0.10899	0.349847	0.350365	4.326669
0.418182	0.746164	0.735879	0.990893	0.944984	-0.11187	0.422514	0.422181	5.934887
0.384768	0.806882	0.800138	0.996051	0.967782	-0.12685	0.422109	0.329348	3.904812
0.26794	0.823717	0.821334	0.997612	0.974079	-0.2167	0.665827	0.332939	7.415299
0.462297	0.765665	0.757373	0.994863	0.960425	-0.10857	0.379018	0.396762	7.581796
0.388127	0.777769	0.772339	0.993918	0.957073	-0.07401	0.323376	0.397613	5.220259
0.377259	0.785931	0.779998	0.994187	0.958586	-0.07633	0.371977	0.378029	5.915153
0.627442	0.699537	0.682798	0.99813	0.972911	-0.22379	0.773224	0.456851	15.67412
0.28845	0.798856	0.795984	0.995394	0.962366	-0.07864	0.330303	0.378611	4.682911
0.709214	0.591154	0.55062	0.980876	0.908259	-0.19357	0.697628	0.474058	6.941448
0.404593	0.749324	0.741183	0.99642	0.964033	-0.04875	0.272741	0.433565	5.972808
0.315451	0.806634	0.802761	0.99715	0.971081	-0.08657	0.366655	0.354383	7.006164
0.437056	0.801289	0.795756	0.994939	0.964117	-0.13226	0.489678	0.348188	7.989306
1.2657	0.661789	0.634379	0.982578	0.92682	-0.28394	0.853491	0.418895	7.514049
104.9679	0.336727	0.272067	0.972079	0.914094	-0.27746	0.948055	0.220597	13.93063
0.57705	0.699679	0.68225	0.986948	0.932104	-0.1144	0.47817	0.453244	6.39139
1.043108	0.794936	0.782072	0.989534	0.95653	-0.23684	0.676481	0.283118	7.589034
0.484161	0.75262	0.74335	0.995975	0.96365	-0.11323	0.399716	0.415276	8.2595

DX50	DX51	DX52	DX53	DX54	DX55	DX56	DX57	DX58
0.176923	3.033856	0.373593	0.317451	12.75554	2.27508	0.554351	5.849832	1131.24
0.214786	2.684941	0.395995	0.344945	7.101975	2.076782	0.478423	5.713477	1317.207
0.241614	2.333422	0.395059	0.324402	9.069818	1.85991	0.325415	5.621897	1638.019
0.411405	1.96312	0.309909	0.620179	6.018397	1.567357	0.244072	4.551418	63.69401
0.141524	3.437663	0.454298	0.244772	14.29234	2.587304	0.992507	5.999261	266.4196
0.169839	3.088632	0.441069	0.261543	9.213805	2.352904	0.610587	6.043231	920.595
0.126138	3.530157	0.486729	0.249507	86.61329	2.560108	1.433854	5.993554	427.3146
0.124049	3.542675	0.58053	0.246369	10.7459	2.672623	0.868097	6.136809	876.181
0.135065	3.428862	0.549982	0.276882	7.947358	2.646666	0.800014	5.755491	73.72059
0.160182	3.269297	0.517004	0.284747	89.19306	2.413901	0.840256	5.922259	1877.121
0.142422	3.236171	0.567285	0.238138	14.7314	2.55018	0.699804	6.175447	8510.999
0.165111	3.124031	0.623031	0.29134	10.76465	2.544967	0.6767	6.224961	20848.81
0.151173	3.266159	0.567712	0.243686	10.69216	2.571728	0.73508	6.238422	1575.886
0.13246	3.491864	0.477721	0.272198	10.04419	2.624184	0.802563	6.034395	312.5037
0.163833	3.140912	0.447613	0.290566	9.44699	2.387895	0.631521	5.735026	121.7391
0.241369	2.738811	0.495851	0.443763	11.88562	2.197096	0.466824	5.859852	4289.893
0.248959	2.597837	0.401594	0.444212	8.065627	2.042461	0.399512	5.547592	169.0499
0.10706	3.602526	0.689468	0.191178	12.62328	2.824678	1.099365	6.412191	2118.74
0.132882	3.474306	0.529009	0.254801	11.93848	2.633448	0.899628	6.249651	1439.231
0.126784	3.450836	0.750473	0.19772	13.96777	2.733984	1.088418	6.275777	5498.235
0.102758	3.569935	0.742371	0.16178	11.23328	2.867949	1.128761	6.428775	8363.952
0.098182	3.960867	0.627008	0.220865	13.60737	2.973629	1.288875	6.393972	2615.201
0.174773	3.082653	0.446682	0.275292	11.03953	2.373524	0.618888	5.964176	198.393
0.137762	3.314554	0.594679	0.249401	11.51041	2.613188	0.791873	6.276393	4878.684
0.210661	2.909902	0.609145	0.396827	14.0295	2.289548	0.564415	5.990187	1859.192
0.267419	2.461813	0.326681	0.443436	8.500678	1.896811	0.3868	5.445574	189.6784
0.48838	1.771991	0.56569	0.685649	86.08792	1.462455	0.426108	5.086415	1048.105
0.103927	3.810102	0.608241	0.216174	10.5826	2.903501	1.156319	6.402151	1452.324
0.161487	3.198981	0.455744	0.28632	12.6967	2.448245	0.654282	6.054006	522.483
0.095789	3.833031	0.599073	0.182166	11.55291	2.872654	1.167439	6.417988	888.2771
0.2107	2.600347	0.301759	0.314442	6.835791	2.000052	0.381163	5.445468	172.154
0.152596	3.28441	0.577691	0.313152	9.63606	2.585824	0.734889	6.145524	3187.102
0.212465	2.895414	0.380601	0.399124	11.73782	2.195025	0.496405	5.61718	217.2844
0.119352	3.484916	0.672351	0.222463	13.30173	2.760091	1.010873	6.392193	3246.991
0.177535	2.939966	0.686578	0.284883	8.755152	2.430892	0.64049	6.077462	18882.16
0.301486	2.114883	0.383047	0.465977	9.281989	1.667125	0.290603	5.411868	385.626
0.100806	3.703118	0.77297	0.164698	9.803043	3.035021	1.299452	6.674257	8476.088
0.195007	2.773134	0.34699	0.289998	7.293485	2.088025	0.447958	5.639005	144.3922
0.224908	2.485409	0.347516	0.300232	12.85404	1.908702	0.375641	5.523969	408.3464
0.159354	3.385105	0.611474	0.296103	12.6923	2.655529	0.947143	6.230796	159.6585
0.151059	3.366123	0.50359	0.255071	14.83945	2.536241	0.80067	6.22164	1000.802
0.127914	3.70627	0.809628	0.298864	15.03678	2.978193	1.521037	6.48718	11129.07
0.071846	4.233585	0.609684	0.126672	13.94644	3.157128	1.550462	6.509484	828.5066
0.169618	3.118595	0.623264	0.303194	12.53829	2.509071	0.711831	6.24025	4988.635
0.234535	2.69497	0.57675	0.431165	13.93972	2.197756	0.474405	5.850175	2761.621
0.284201	2.419775	0.354231	0.488128	6.192969	1.917511	0.344073	5.546543	268.1487
0.257231	2.557183	0.388032	0.456905	8.01604	2.024037	0.385776	5.660293	426.6841
0.105296	3.783933	0.653061	0.218698	10.1008	2.92713	1.314782	6.401735	2470.437
0.146307	3.300882	0.661548	0.262395	8.728391	2.643301	0.804865	6.199825	1819.055
0.167296	3.164649	0.702781	0.31133	8.728422	2.613366	0.853862	6.277795	1336.596
0.234844	2.609931	0.353657	0.40399	12.42288	2.023782	0.408254	5.691604	3166.187
0.274915	2.426025	0.328464	0.444779	7.424087	1.81826	0.452393	5.390328	201.4094
0.198007	2.990321	0.406137	0.37708	88.069	2.231763	0.626648	5.70938	399.3838
0.200495	3.035012	0.655203	0.388641	11.80804	2.334888	0.604134	6.046081	777.1105
0.144387	3.397755	0.574059	0.232153	14.49497	2.63849	0.957743	6.3277	4601.874
0.205698	3.397103	0.606092	0.409487	17.64128	2.639233	1.545401	6.164956	1338.334
0.214215	2.96503	0.499091	0.413183	9.654229	2.317922	0.578443	5.845218	781.4341

0.24445	2.665969	0.298527	0.441383	7.783936	1.998762	0.40738	5.668526	238.0307
0.124384	3.608978	0.531612	0.263215	12.46926	2.730673	0.938972	6.185969	268.9537
0.167069	3.241344	0.41582	0.326034	12.08619	2.42938	0.70529	6.061148	802.6794
0.131235	3.443503	0.708428	0.22984	14.47578	2.790953	0.925403	6.424697	25651.72
0.121738	3.582711	0.434467	0.209658	8.811061	2.645612	0.863162	6.129675	311.1032
0.302987	2.509724	0.411076	0.515275	11.62188	1.977334	0.439583	5.631851	1233.847
0.248512	2.724798	0.547349	0.449444	13.33223	2.175991	0.505747	5.828513	3517.052
0.122866	3.441111	0.683721	0.195038	9.987664	2.711026	0.96615	6.260899	2980.693
0.203891	2.6874	0.316208	0.262643	8.97959	2.005927	0.426378	5.639388	330.673
0.165675	3.126416	0.322221	0.323522	7.980029	2.285431	0.578162	5.579505	139.2155
0.205991	2.810462	0.464506	0.366206	8.745599	2.223385	0.487624	5.772947	137.7758
0.10802	3.597909	0.598136	0.180215	10.40749	2.772524	0.922469	6.222543	3402.839
0.165282	3.030083	0.457957	0.25881	10.94609	2.355156	0.550455	5.884858	16762.72
0.11188	3.481198	0.752811	0.2065	7.137832	2.83901	1.112136	6.42162	7295.039
0.139625	3.325637	0.815111	0.249288	11.86152	2.699802	1.653936	6.355803	9844.863
0.28701	2.534276	0.620027	0.498426	87.66393	2.067912	0.587557	5.652691	5897.295
0.334508	2.137401	0.291137	0.524432	5.416617	1.646701	0.313905	4.941483	85.11809
0.124939	3.386794	0.597374	0.212526	9.188245	2.661436	0.811296	6.248173	908.9114
1	######	1	1	2	######	0	0.927856	5705.47
0.13367	3.480835	0.508042	0.23952	12.73545	2.614039	0.908987	6.152355	672.4796
0.134198	3.403608	0.52149	0.232694	10.86258	2.603268	0.781945	6.211134	3566.974
0.232698	2.471206	0.354781	0.347879	6.798848	1.95393	0.359156	5.71456	857.8068
0.04162	4.94782	0.801574	0.072873	14.16721	3.579706	2.637381	6.627345	5378.444
0.176389	3.412127	0.703128	0.375203	11.18937	2.730541	1.055878	6.328514	2345.055
0.129988	3.454351	0.37457	0.261946	14.08189	2.452134	0.688297	5.67083	975.617
0.192006	2.912442	0.585774	0.359149	10.15912	2.32448	0.557956	5.966883	4428.012
0.209421	2.784758	0.445205	0.387062	9.728948	2.20525	0.462139	5.846001	13408.44
0.204132	2.876822	0.31321	0.385604	9.65115	2.144056	0.461983	5.556644	1173.77
0.136537	3.375439	0.41361	0.272068	11.90943	2.448477	0.663124	5.760315	3548.125
0.184297	3.151119	0.465867	0.37002	11.57378	2.407062	0.660481	6.019637	935.7458
0.20322	2.819185	0.363948	0.370161	13.54457	2.110205	0.477288	5.535246	117.0249
0.182643	3.251443	0.480998	0.374078	13.34871	2.464933	0.767937	5.982331	1604.507
0.135754	3.270018	0.590536	0.225609	13.8566	2.564115	0.789228	6.147022	4859.516
0.205046	3.116401	0.58831	0.397391	7.797744	2.449372	1.101955	6.078581	3594.454
0.132062	3.466894	0.541828	0.227766	12.27268	2.654832	0.865207	6.215591	916.9783
0.072002	4.319653	0.650737	0.134239	12.77028	3.240012	1.868137	6.68387	4818.834
0.209427	2.897826	0.439179	0.391797	11.89259	2.204344	0.561817	5.644357	301.4719
0.262283	2.620261	0.480086	0.467434	10.08548	2.103056	0.430113	5.76576	2224.663
0.188202	2.919461	0.342747	0.304597	13.0071	2.185271	0.566185	5.628415	158.8712
0.154044	3.131726	0.387699	0.22502	6.898757	2.352863	0.586721	5.653175	248.7774
0.095007	3.892736	0.605614	0.195365	10.89524	2.919385	1.163417	6.336208	738.7575
0.16762	3.154871	0.40157	0.310349	11.49411	2.350512	0.630319	5.957053	2017.27
0.152129	3.248641	0.659953	0.247091	7.237608	2.528779	0.732128	6.081033	389.7142
0.190263	2.972396	0.573834	0.340764	12.34391	2.305084	0.5887	5.87836	3237.121
0.150816	3.278145	0.55616	0.289734	11.03794	2.534802	0.744118	6.105515	806.5065
0.196042	2.95537	0.389521	0.356974	10.28842	2.251355	0.566267	5.797716	587.7408
0.183567	3.155767	0.378373	0.362136	13.8991	2.339498	0.674971	5.861065	517.6851
0.216759	2.599799	0.354025	0.27485	12.79006	1.996603	0.525979	5.648416	1305.393
0.247347	2.472498	0.329612	0.39205	8.592896	1.911294	0.384452	5.485723	353.6944
0.260313	2.598616	0.388957	0.454053	9.553344	1.999095	0.411091	5.500178	506.5536
0.170601	3.1228	0.594381	0.335728	11.85694	2.463529	0.658725	6.066173	5006.257
0.151682	3.290153	0.615791	0.294948	9.683022	2.599051	0.988989	6.255629	11215.03
0.18344	3.127258	0.617803	0.366125	13.5629	2.464471	0.74225	6.059462	4442.531
0.282324	2.428693	0.302959	0.483472	6.196862	1.890441	0.340476	5.459699	661.2747
0.193426	2.802341	0.416089	0.270906	7.238105	2.179374	0.481753	5.82102	934.5207
0.154048	3.020966	0.737081	0.245074	85.72413	2.479465	1.48771	6.126015	6303.73
0.164814	3.130683	0.4557	0.260936	11.14449	2.377042	0.629368	6.010068	975.8015
0.260837	2.519374	0.285565	0.455336	14.23624	1.915986	0.357316	5.452486	2555.469

0.088752	3.910066	0.465023	0.155427	10.96621	2.814676	1.008877	6.014463	1471.44
0.135871	3.324994	0.40888	0.242043	6.774683	2.474591	0.690449	5.957577	650.6296
0.2116	2.986772	0.496041	0.401103	14.1846	2.275423	0.772933	5.845677	5726.266
0.247589	2.623418	0.343308	0.422051	11.37067	2.003778	0.479373	5.634379	533.4392
0.181103	2.914765	0.230217	0.322892	10.40698	2.084067	0.459602	5.177954	232.6661
0.162679	3.298185	0.670959	0.322179	14.25379	2.648896	0.986881	6.340587	6348.629
0.131375	3.320986	0.427579	0.234071	7.748999	2.486774	0.710366	5.940813	405.58
0.233495	2.573678	0.380163	0.387303	11.31522	2.03002	0.38911	5.761112	1678.349
0.233135	2.676677	0.32863	0.420693	8.354993	2.038135	0.407505	5.545249	226.2963
0.230032	2.594384	0.367781	0.36853	8.764096	2.018814	0.403934	5.720454	987.663
0.157094	3.21036	0.496347	0.2774	9.069916	2.473201	0.673434	6.125075	2063.49
0.127735	3.559032	0.356069	0.257119	11.14567	2.53826	0.867718	5.995256	686.7204
0.150385	3.354975	0.743142	0.245915	10.50154	2.663198	1.055877	6.239507	2687.899
0.302999	2.411226	0.417289	0.50226	13.52488	1.916685	0.383563	5.651083	3551.146
0.177556	3.180365	0.594627	0.322784	14.42924	2.442074	0.764795	6.03325	3475.345
0.136063	3.54129	0.619912	0.271217	17.74992	2.712166	1.095797	6.269625	1225.054
0.10708	3.706334	0.609047	0.217148	12.63615	2.80863	0.986525	6.2554	2844.949
0.158042	3.133501	0.706606	0.261683	10.29561	2.592326	0.848418	6.301072	14526.84
0.2373	2.574031	0.369293	0.394851	6.534989	2.004505	0.40009	5.554327	531.1708
0.206839	2.850574	0.40566	0.357692	10.5007	2.177945	0.50586	5.874186	758.6567
0.12523	3.419384	0.372783	0.19347	8.863164	2.497528	0.751237	5.916467	530.4402
0.237828	2.848899	0.32041	0.442663	13.85466	2.13612	0.50964	5.727135	1228.575
0.141475	3.389995	0.515923	0.272766	12.00821	2.5507	0.794958	6.284484	805.0923
0.376926	2.069097	0.211564	0.585797	8.148566	1.609805	0.25075	5.146578	280.6081
0.124687	3.445725	0.561712	0.208046	11.92559	2.661439	0.846178	6.337119	7565.354
0.142138	3.660656	0.732622	0.322771	8.901686	2.875331	1.426118	6.315888	1143.592
0.108643	3.762429	0.556747	0.249411	10.94495	2.8228	1.145232	6.168228	872.8098
0.213013	2.626029	0.468577	0.297481	87.08144	2.041678	0.447935	5.730673	1969.46
0.178435	3.030874	0.382808	0.343531	11.85564	2.269955	0.543148	5.774821	157.8804
0.094791	4.061192	0.37992	0.170641	16.82239	2.830482	1.330178	6.010559	321.6089
0.059746	4.669612	0.428249	0.123064	14.23232	3.229631	2.155221	5.976665	99.41191
0.140304	3.430829	0.480674	0.238296	10.79149	2.613875	0.892166	6.093738	378.9089
0.119613	3.520429	0.638651	0.237672	13.63988	2.763625	0.926288	6.341086	2304.755
0.166339	3.147099	0.385119	0.325023	10.10123	2.334607	0.594052	5.858246	2681.31
0.152847	3.154381	0.23507	0.228733	9.10131	2.222224	0.544918	5.551896	330.0767
0.167669	3.33564	0.4601	0.337286	10.73038	2.542539	1.051184	5.624745	90.11201
0.068877	4.490011	0.647963	0.138553	14.17841	3.3508	2.268286	6.799887	573.223
0.136176	3.541657	0.5444	0.238694	13.04902	2.697912	1.197075	6.232897	1117.864
0.231943	2.499691	0.42498	0.321033	12.99641	2.008457	0.385341	5.780688	1651.063
0.14734	3.556573	0.500741	0.297071	16.01088	2.668903	1.185259	6.034729	1951.178
0.282089	2.592504	0.389712	0.492805	11.76796	2.014493	0.440965	5.660303	609.6871
0.099812	3.987004	0.41074	0.200811	13.05867	2.855053	1.483315	6.063966	183.8965
0.042548	5.096849	0.728227	0.085916	16.60367	3.718533	3.465424	6.828758	1483.449
0.114648	3.802934	0.530744	0.253074	11.96729	2.831707	1.181099	5.853635	75.72988
0.36456	2.301074	0.503846	0.580921	7.762469	1.869491	0.376074	5.559029	654.0162
0.099512	3.953779	0.664631	0.19498	15.79374	3.071325	1.450403	6.518094	451.2183
0.106418	3.868382	0.415179	0.214119	14.29758	2.727341	1.118147	5.910109	433.1191
0.215799	2.672187	0.609986	0.367654	12.8119	2.186573	0.555898	5.929627	2609.392
0.159093	3.515178	0.49513	0.333875	12.43275	2.661707	1.040086	6.137372	415.1833
0.232648	2.782713	0.847421	0.429086	71.82403	2.205722	0.521229	5.901142	3228.674
0.147822	3.393497	0.369921	0.301634	11.90113	2.455453	0.777614	5.864081	624.4213
0.085001	4.132592	0.511584	0.18058	13.15365	2.982526	1.304006	6.276778	527.7256
0.102626	3.633567	0.391809	0.156284	8.426539	2.53726	0.889562	5.555775	65.51809
0.140519	3.375487	0.373245	0.280623	13.83212	2.362514	0.684885	5.663225	1031.706
0.131004	3.582126	0.387283	0.269902	11.60009	2.534034	0.828177	5.825484	954.2498
0.105547	3.846468	0.37721	0.226005	14.57615	2.67081	1.0915	5.788682	162.7068
0.127082	3.424207	0.672378	0.211869	12.95264	2.727651	1.094402	6.416569	5961.457
0.130074	3.645203	0.463281	0.284798	11.72613	2.665819	0.985762	6.03352	352.4061

0.122915	3.578446	0.467326	0.245393	13.77352	2.631548	0.959191	6.198638	1879.925
0.181988	3.272003	0.447825	0.33986	13.76405	2.427013	6.328541	6.247143	642.7644
0.107067	3.85578	0.359617	0.223506	14.18414	2.695676	1.060297	5.831169	428.4607
0.173981	3.105882	0.489556	0.294816	11.08553	2.427597	0.658803	6.01352	421.8117
0.151196	3.339373	0.45083	0.300112	16.11768	2.386701	0.723146	5.783904	1756.651
0.174946	3.231354	0.317738	0.354658	11.43101	2.349571	0.690735	5.734427	267.7851
0.187596	3.156335	0.554916	0.359363	13.20785	2.44933	0.729781	6.095184	1281.063

0.110524	3.957155	0.469661	0.247264	12.21968	2.869585	1.264738	6.16802	677.2622
0.137895	3.55935	0.597935	0.216226	17.17848	2.628979	1.845786	6.036497	26240.14
0.197414	3.201265	0.388357	0.380174	13.1146	2.37837	0.889471	5.770149	813.4023
0.114974	3.801067	0.412192	0.255481	13.33912	2.723022	1.134923	5.923049	310.0181

0.234139	2.427033	0.461281	0.29254	24.86036	1.925135	0.402487	5.688394	16020.97
0.133604	3.377974	0.566553	0.215594	14.11369	2.61087	0.839094	6.336624	2529.313
0.10032	3.711126	0.590758	0.165349	12.40365	2.780043	1.030999	6.338635	3900.391
0.114298	3.698159	0.47949	0.195677	12.54612	2.707901	1.052412	5.682863	81.19604
0.130678	3.619793	0.422771	0.277753	13.82616	2.663182	0.9895	6.117527	765.4686
0.174925	3.202854	0.471431	0.344926	12.27438	2.425636	0.68453	6.033266	3250.897
0.120126	3.59055	0.349076	0.232732	11.23838	2.551318	0.789008	5.962947	1576.482
0.30789	2.479278	0.299806	0.522373	9.854992	1.880255	0.405299	5.462731	244.7014
0.1733	3.251247	0.464532	0.349323	11.73265	2.466977	0.805803	5.932711	374.8601
0.183557	3.266187	0.751332	0.380415	11.17978	2.656079	0.956607	6.306534	2472.485
0.12014	3.524935	0.540688	0.246358	11.21209	2.571604	0.984042	5.542821	48.46667
0.213432	2.659357	0.415205	0.340558	9.143851	2.110238	0.415623	5.775878	4030.47
0.11173	3.648017	0.63576	0.202751	13.77529	2.838389	1.021972	6.455095	1761.765
0.042327	4.94436	0.654924	0.089758	11.13238	3.575725	3.546424	5.947529	64.22794
0.104569	3.910721	0.569661	0.19172	12.82403	2.936016	1.252288	6.382265	1121.082
0.144768	3.399838	0.627288	0.265927	13.8194	2.662111	0.908709	6.324141	3746.135
0.106553	3.60367	0.628281	0.198301	8.867019	2.787281	0.964216	6.243219	1300.519
0.153121	3.279787	0.371763	0.229305	13.07989	2.360626	0.66483	5.697811	1131.229
0.066238	4.375381	0.44197	0.113982	12.71538	3.112764	1.939866	6.039458	128.0312
0.127946	3.64027	0.408344	0.205711	14.68619	2.598831	1.027353	6.016456	675.8573
0.135609	3.76347	0.592245	0.286506	12.22136	2.867571	1.33825	6.421233	685.5651
0.097759	4.126514	0.357026	0.199964	16.46802	2.891066	1.423902	6.288879	497.7036
0.088965	4.131579	0.603119	0.188206	14.23503	3.015763	1.544956	6.521886	529.3786
0.150583	3.034996	0.664266	0.226423	6.501438	1.992664	0.694811	4.537043	38.5089
0.232394	2.476915	0.418045	0.33891	5.227101	1.97503	0.384875	5.685932	559.1929
0.149493	3.254189	0.502229	0.258117	5.169006	2.37273	0.721002	5.493296	68.51496
0.239841	2.421635	0.457648	0.34554	87.13021	1.894449	0.379565	5.668551	2063.628
0.224775	2.777497	0.512345	0.408892	14.26949	2.201061	0.481419	5.870845	6117.366
0.302848	2.269559	0.422425	0.494685	11.51067	1.850821	0.316016	5.602271	609.6065
0.158742	3.010482	0.622186	0.256623	9.90811	2.462504	0.647386	6.168598	2123.282
0.271583	2.451121	0.315558	0.451285	6.659638	1.873058	0.370054	5.468253	383.8273
0.224145	2.841002	0.342822	0.405578	13.28892	2.141579	0.538361	5.679887	279.9047
0.373815	2.037503	0.286724	0.574113	8.342009	1.610791	0.265539	5.243115	408.1112
0.21422	2.749867	0.379108	0.328929	16.73209	2.10749	0.543651	5.80342	1330.532
0.171508	3.041917	0.457095	0.305436	15.23709	2.295457	0.540009	5.744972	7439.993
0.128648	3.417787	0.574769	0.206534	8.141848	2.642311	0.85508	6.298296	2492.056
0.259729	2.726006	0.342632	0.463936	8.138829	2.085281	0.497228	5.540557	243.2315
0.223984	2.596351	0.384882	0.360518	4.799323	2.041758	0.394327	5.732839	496.9472
0.182504	3.028799	0.393338	0.318881	9.484807	2.226344	0.571291	5.765239	238.1103
0.190208	3.052471	0.641931	0.374259	34.2177	2.389602	0.639803	6.061974	4875.585
0.079204	4.147245	0.634499	0.1525	14.51051	3.088189	1.524597	6.227096	192.4592
0.174157	3.291915	0.626321	0.347632	16.29007	2.413952	1.611987	5.782108	3163.598
0.275291	2.902267	0.616582	0.494596	15.90239	2.401031	1.504566	5.407542	83.59314
0.172245	3.175288	0.383477	0.332567	14.13218	2.264364	0.65916	5.422919	333.0449
0.322022	2.489322	0.356403	0.536047	13.59479	1.923766	0.468031	5.471481	340.0153

0.427699	2.014446	0.595444	0.634375	13.74409	1.699328	0.347673	5.257546	3856.869
0.14415	3.464036	0.651932	0.303993	15.89517	2.763802	0.915442	6.47305	8711.755
0.261496	2.649606	0.683126	0.464767	12.69657	2.214792	0.518375	5.829953	7188.87
0.344305	2.579173	0.706351	0.563598	14.43855	2.013826	6.164352	5.428424	3789.825
0.248303	2.232046	0.429291	0.328437	6.935487	1.742649	0.299825	5.482505	1874.154
0.212181	2.731446	0.283059	0.364083	9.441647	2.05695	0.421304	5.606314	735.7233
0.205674	2.76476	0.464658	0.278546	11.14535	2.128809	0.516793	5.836469	601.2935
0.24826	2.321687	0.46114	0.362945	86.92362	1.829693	0.361642	5.572089	1977.826
0.167913	3.250167	0.381664	0.3331	7.186024	2.421803	0.863555	5.800998	341.1501
0.231157	2.353158	0.241207	0.275575	5.015681	1.80697	0.313874	5.396855	302.4759
0.232386	2.669864	0.415686	0.396225	12.50385	2.070024	0.460759	5.672009	648.5368
0.256944	2.379349	0.296374	0.392995	8.77313	1.844343	0.345562	5.507153	1530.842
0.157648	3.326474	0.399599	0.321009	8.533499	2.358941	0.801543	5.493957	236.0845
0.343962	2.143364	0.250704	0.545514	6.298163	1.674448	0.27367	5.29835	236.0922
0.315913	2.224224	0.397069	0.501766	8.653338	1.751546	0.342698	5.412841	1877.905
0.190812	3.041024	0.408595	0.368343	11.86977	2.278913	0.592901	5.721444	188.3662
0.308028	2.494268	0.413429	0.517101	7.809624	1.902281	0.438626	5.672411	385.8278
0.225015	2.653873	0.645897	0.326513	14.8306	2.200307	0.491821	5.983933	5023.449
0.224059	2.797972	0.379483	0.387747	15.16359	2.147481	0.586568	5.74635	2610.332
0.25709	2.562137	0.353855	0.433705	10.44052	1.967464	0.435968	5.559398	1289.576
0.290743	2.593304	0.445148	0.50186	11.83031	1.965548	0.426526	5.517753	1221.986
0.077483	4.270271	0.697281	0.163079	31.34824	3.247358	1.847229	6.686651	6895.056
0.300019	2.269527	0.352072	0.48045	9.365823	1.772817	0.330245	5.392201	1949.539
0.077262	4.15825	0.539261	0.156266	13.8829	3.010097	1.964542	5.498725	56.34172
0.242896	2.74523	0.397711	0.442867	11.94562	1.993809	0.442352	5.376675	318.3632
0.321652	2.392243	0.441233	0.535614	14.01233	1.87777	0.366477	5.466015	2936.721
0.317485	2.517732	0.681918	0.52883	15.97861	1.989564	0.733126	5.523211	3964.778
0.083828	4.639973	0.838855	0.21768	15.0281	3.552777	6.788669	6.669936	1530.156
0.011902	8.134078	0.63864	0.059682	27.86127	5.670292	160.3132	7.156631	194.1968
0.131894	3.486966	0.403707	0.237179	12.78278	2.541068	0.860734	5.592772	73.22872
0.324578	2.912698	0.720564	0.550814	15.17807	2.355304	2.521134	5.784188	999.431
0.219387	2.962773	0.40024	0.398449	16.519	2.238363	0.76122	5.847453	237.0081

DX59	DX60	DX61	DX62	DX63	DX64	DX65	DX66	DX67
0.04911	31.10712	8748.548	0.607556	41.4626	185.6012	7413.035	4.784271	0.025786
0.045419	38.04247	11199.24	0.546256	13.83966	241.5895	2799.105	22.90147	0.085266
0.049494	30.39215	15166.8	0.358156	21.11727	250.5991	5278.696	12.53816	0.050872
0.070614	23.28922	503.6275	0.32756	9.758315	162.592	1473.647	18.57398	0.11956
0.054216	25.38816	1569.648	1.235173	51.9247	151.6829	8171.068	2.943448	0.023241
0.046964	33.39994	7017.607	0.687601	21.92455	206.2469	4279.015	10.70129	0.052548
0.052971	26.87448	2491.793	4.255006	1879.445	138.5386	259361.1	0.145265	0.002391
0.054526	25.52252	4884.058	0.943574	30.13679	158.021	4528.493	5.973545	0.038004
0.060229	21.05892	352.1062	0.983444	17.10131	123.6242	2046.755	8.498658	0.082301
0.049445	29.93348	13510.62	1.579114	1987.988	176.2183	351737.6	0.103473	0.000899
0.0482	31.94401	57905.63	0.731175	54.94847	228.9836	12588.16	4.352924	0.019296
0.04661	34.03379	153744.7	0.691078	29.90995	273.3533	7894.16	10.18981	0.036917
0.045885	34.48371	11182.53	0.825817	29.3405	216.3314	6439.719	7.851674	0.039457
0.054653	25.75349	1811.9	0.875728	26.15985	148.2277	3742.939	6.331214	0.045191
0.059039	22.99917	684.0572	0.846505	23.79534	131.5752	3014.339	6.142003	0.051155
0.042754	41.8956	43400.76	0.509026	35.92776	284.01	10086.88	8.221432	0.029612
0.047366	33.85501	1564.68	0.451753	16.96918	196.9484	3175.597	12.8207	0.06723
0.051757	27.8797	10648.8	1.194367	41.84029	189.4992	7563.866	5.203122	0.026834
0.049199	30.73419	9223.681	0.966039	36.11906	188.6676	7101.927	5.445658	0.033407
0.050192	29.54435	32522.17	1.096278	50.1446	215.0718	10733.5	4.798468	0.022202
0.051641	27.82149	41025.51	1.13496	33.14993	214.3327	6964.355	7.678108	0.035157
0.053053	26.31707	12885.34	1.448836	47.82379	160.636	7577.781	3.728525	0.024502
0.047372	34.12606	1470.289	0.750493	31.23734	182.7354	5688.334	6.142036	0.036964
0.048811	31.54	30982.17	0.833736	34.58678	224.2558	7580.446	7.15638	0.032135
0.043202	39.26978	17139.2	0.648264	49.81301	251.4403	12355.17	5.25111	0.021708
0.048486	32.15317	1845.631	0.428091	18.6976	188.7612	3258.132	11.33645	0.060796
0.048206	47.68181	14429.81	1.207296	1852.815	340.5951	630191.1	0.186943	0.000802
0.053125	28.63078	7230.192	1.201445	30.23824	170.7024	5067.36	6.737134	0.040274
0.047728	32.68363	3750.363	0.771362	41.1003	188.2162	7659.894	4.797351	0.026779
0.053136	26.8974	4377.046	1.238773	34.55716	162.138	5761.973	5.239921	0.036837
0.056278	24.34881	1308.154	0.417626	12.37986	155.1778	1805.745	14.50766	0.09438
0.048823	30.86443	22282.76	0.745452	24.35122	210.2924	5035.829	9.776147	0.04771
0.046889	34.07865	1895.268	0.595843	35.41196	176.1688	6184.931	5.130375	0.030556
0.046998	34.09767	18826.01	1.051501	45.58386	218.1137	9935.872	5.194912	0.024576
0.045902	36.21364	147753.3	0.687575	20.06442	286.6931	5784.948	15.67046	0.057552
0.046913	34.15746	4247.433	0.312634	21.73309	224.6579	5223.684	10.00442	0.049775
0.048062	31.90884	41771.02	1.348363	26.12246	240.0991	5854.937	12.38322	0.048482
0.055196	25.50275	1037.129	0.527852	14.02561	153.4106	2063.579	12.52075	0.085167
0.053765	26.69656	3390.463	0.427762	41.53114	190.2434	7915.219	4.687666	0.025429
0.046807	36.9443	1022.3	1.346627	41.78892	176.1234	7268.202	4.462378	0.030054
0.047154	33.37652	7054.566	0.991853	55.53548	183.0929	10501.08	3.311808	0.020296
0.044915	35.9317	68323.13	1.529465	58.44169	234.9286	13834.22	4.439778	0.019414
0.059691	21.50152	2978.2	1.662296	50.02089	134.2927	6614.671	3.083936	0.023389
0.044514	37.10293	38807.53	0.793625	40.59707	260.129	10215.38	6.956737	0.026706
0.044187	38.44671	27295.73	0.497281	49.23532	271.2052	13151.47	5.716577	0.021267
0.044447	37.73766	2906.62	0.373569	10.01823	226.0681	2170.009	25.03476	0.119255
0.045286	36.3638	4374.311	0.404289	16.24422	238.9079	3898.156	15.38993	0.069138
0.050995	30.21464	12714.67	1.316	27.35601	184.4875	5116.12	8.6119	0.046991
0.049685	29.98176	11867.66	0.83726	20.36327	218.9849	4431.569	12.67296	0.061483
0.045379	37.69352	9506.383	0.932802	20.12389	238.4986	4811.959	13.77747	0.063611
0.044854	36.45253	31292.93	0.465997	38.67906	245.1338	9450.974	6.497495	0.027498
0.047335	34.89392	2052.323	0.539424	14.2651	196.3302	2948.14	13.71592	0.083501
0.046728	33.64444	3407.005	1.329184	1938.483	185.5997	360365.3	0.098787	0.000867
0.042946	40.14845	6910.848	0.712983	35.61597	226.3295	7943.966	6.670076	0.030738
0.043861	38.14599	33469.55	1.06384	53.19689	237.3532	12859.51	4.578066	0.020512
0.041197	44.75945	11868.23	2.126499	79.07708	232.5589	18743.01	3.122867	0.019218
0.045535	36.22971	7137.674	0.583997	23.96323	230.7981	5629.421	10.04001	0.04745

0.044047	38.91602	2399.128	0.495485	15.80292	190.6014	2980.85	12.75766	0.074682
0.052295	29.50655	1442.166	1.109949	40.79039	159.1334	6081.268	4.395496	0.027919
0.048007	33.01228	5845.392	0.808804	36.7256	188.0029	7041.715	5.254557	0.030565
0.04864	31.55981	156005	0.930987	53.57872	253.5715	13262.35	5.159785	0.020131
0.054408	26.31577	1694.367	0.969227	20.81829	144.1966	2951.796	7.985791	0.061027
0.040459	48.60977	14753.79	0.541921	33.78	272.8333	9630.845	7.879739	0.032665
0.04207	42.79444	36787.6	0.549562	44.91233	275.7665	12795.05	6.117651	0.023762
0.050835	28.88837	17662.96	0.95401	26.23672	203.924	5376.428	9.362159	0.045869
0.054226	25.69477	2506.764	0.508103	20.72417	167.4602	3388.168	8.793844	0.054905
0.058152	22.98896	903.0777	0.617685	16.70134	132.8271	2173.023	8.751049	0.072634
0.046389	35.30474	1146.807	0.580599	19.96431	195.0505	3619.411	11.0744	0.056934
0.05129	28.01377	18950.31	0.932986	28.482	183.1153	4997.87	7.588252	0.040823
0.052408	27.70408	119750.6	0.565355	30.67591	220.549	6761.768	7.612636	0.035405
0.049399	30.3103	38525.33	1.116848	14.25431	232.346	3102.978	26.65817	0.105736
0.044646	37.25681	67533.68	1.794508	37.30391	263.2508	9089.636	8.235636	0.030892
0.044228	43.01898	64183.74	0.888365	1922.722	313.8869	604035.3	0.165291	0.000639
0.063616	23.06769	698.8072	0.355518	7.772795	157.0942	1323.62	20.91991	0.175609
0.050622	29.0295	5318.801	0.852914	21.62072	198.792	4429.298	10.12866	0.055997
0.640632	0.287567	8906	0	1	1.883225	1.883225	1.883225	1
0.052329	27.16309	4048.45	1.134873	41.90942	162.9379	6922.431	4.198526	0.03114
0.049135	30.47655	22998.61	0.839516	30.64116	199.5231	5905.886	7.246505	0.036953
0.045377	35.55044	8281.089	0.39054	12.00651	244.8963	2832.956	23.08495	0.097212
0.070261	15.17458	12698.15	2.729124	53.35307	95.62668	4985.626	2.203523	0.02382
0.041308	43.53979	18614.44	1.116677	32.65172	252.9799	8496.354	8.517022	0.036856
0.057044	22.52119	5880.848	0.730301	50.02707	142.2639	7142.677	2.912831	0.021386
0.046508	34.11195	37454.82	0.583541	26.43812	249.9052	6496.625	10.21408	0.041813
0.046316	34.93266	121493.3	0.469602	24.1993	265.2663	6404.868	11.56015	0.045052
0.046762	33.68074	10856.37	0.474711	23.80829	199.4891	4779.703	8.633242	0.046213
0.055421	24.18536	22184.46	0.708033	36.39756	160.7785	5754.52	4.676749	0.029991
0.04356	38.95412	7921.136	0.760366	33.91742	221.228	7685.829	6.624807	0.033461
0.052127	27.80508	914.363	0.670067	46.58352	148.2178	6964.606	3.234214	0.025434
0.042886	40.24852	13394.29	0.912016	44.8848	209.2374	9817.458	4.625439	0.025045
0.047546	32.37708	31698.34	0.83249	49.29279	226.7485	10473.8	5.194709	0.021779
0.044276	39.65	31479.4	1.181932	16.65713	248.1958	3833.755	18.39127	0.08164
0.049685	30.35152	5692.819	0.94904	38.43016	179.1053	7131.677	4.799698	0.029788
0.057308	25.55958	17199.45	1.879339	42.71677	158.0237	6411.273	4.573178	0.028415
0.046863	33.12412	2636.509	0.619103	36.34727	192.3906	6846.869	5.531035	0.029652
0.042861	41.29087	23779.43	0.468996	26.19846	278.4404	7086.756	11.29877	0.04125
0.057479	24.00181	1073.122	0.724651	42.47757	132.225	5893.808	3.086497	0.027784
0.063206	19.6775	1434.226	0.584197	12.50915	125.7947	1584.466	11.48712	0.10109
0.056171	25.01726	3400.395	1.1955	31.07778	139.1814	4255.192	5.226196	0.039372
0.050211	29.9139	14624.8	0.69052	33.45201	194.6096	6610.073	6.011614	0.033243
0.055626	25.53338	2289.794	0.84636	14.80788	150.8673	2068.35	13.05691	0.088158
0.048113	31.72654	26649.96	0.603032	38.6584	216.4147	8341.538	5.867068	0.027969
0.046882	33.42207	5843.075	0.833348	31.48399	197.4207	6212.059	6.698813	0.036309
0.048263	31.91251	4703.146	0.656806	26.6653	190.5467	5150.771	7.369122	0.043175
0.046849	34.28378	4198.812	0.800543	49.50326	170.7104	8288.755	3.58927	0.022223
0.052226	27.56268	10753.03	0.646568	41.04977	208.5193	8763.962	5.152539	0.027455
0.051379	28.72188	3174.187	0.434058	18.74187	190.3707	3443.269	11.05712	0.062614
0.045067	36.17548	5377.236	0.430225	23.40792	218.911	5261.774	9.395089	0.047195
0.047775	32.00241	37905.84	0.689959	35.91693	215.453	7653.501	6.370747	0.03031
0.047073	33.28137	78853.69	1.142949	24.99152	231.8808	5559.838	10.60367	0.047148
0.044248	37.43794	37591.85	0.864048	47.07753	244.4391	11369.5	5.45905	0.022855
0.044684	36.86286	7238.623	0.364704	10.22988	224.2691	2128.842	24.99785	0.116194
0.049198	31.01454	7396.472	0.536287	13.86465	203.8882	2614.142	17.5754	0.085275
0.045317	36.10849	44695.93	3.545005	1840.8	268.6245	492053.2	0.257957	0.002153
0.049213	30.64714	6898.496	0.812375	32.3082	177.7527	5654.475	5.861991	0.034623
0.044692	36.37694	27904.62	0.369521	51.01525	229.706	11535.7	4.621433	0.020234

0.063421	18.65267	6412.236	1.041464	31.55493	124.3902	3773.316	4.526974	0.036809
0.055143	25.06091	3914.653	0.710065	12.37079	155.9082	1770.044	16.51863	0.105849
0.043241	39.72064	54978.16	0.857816	50.73729	237.3714	11870.91	4.831541	0.021187
0.047834	33.38276	4947.751	0.571333	32.9792	208.0956	7060.652	6.34944	0.034164
0.071678	15.13609	1329.347	0.483438	27.5228	84.02711	2248.835	3.252892	0.039474
0.045152	36.89042	46003.48	1.545342	53.4587	251.2233	12740.6	5.1943	0.020525
0.058551	25.00777	2195.967	0.747036	16.34994	148.4015	2283.992	11.18829	0.076573
0.044781	36.76974	16588.63	0.429461	32.37266	248.8012	8257.039	7.731514	0.032901
0.04568	35.29225	2184.252	0.451295	18.37081	193.979	3299.773	11.83744	0.060877
0.046969	34.23272	8950.665	0.444447	19.92382	224.3442	4209.492	12.581	0.055433
0.048969	31.42943	14438.24	0.707478	21.23684	207.0091	4477.939	10.64365	0.055669
0.051969	28.24711	4210.629	0.984978	31.23331	145.4842	4758.825	4.753356	0.039309
0.047844	32.56413	18004.91	1.066687	28.90217	217.7566	6180.064	9.28616	0.04217
0.043011	44.84781	40357.56	0.445494	46.03706	296.1123	13987.41	6.368364	0.022815
0.046995	32.92652	27567.13	0.826905	52.89685	211.7991	11065.76	4.201123	0.020365
0.049229	30.9762	7523.729	1.167719	79.4168	189.3152	15616.81	2.387027	0.013561
0.054523	24.99915	14606.21	1.082851	41.28845	165.3853	6638.936	4.418352	0.027188
0.045483	36.22796	105384.8	0.863066	27.70923	282.9061	7672.687	11.54061	0.041153
0.055801	27.38749	4128.167	0.430582	11.3201	187.7335	1970.729	19.81101	0.10502
0.047148	34.97802	6366.435	0.577456	28.37947	207.5384	5646.476	7.9669	0.039643
0.062589	21.82926	2641.378	0.782905	21.21971	137.0894	2810.054	7.497315	0.056106
0.042189	42.06349	12639.27	0.595063	48.33979	226.4499	11051.45	4.706586	0.022031
0.048573	33.35179	5071.109	0.91562	37.21961	194.1801	7122.017	5.623754	0.030474
0.042292	46.29667	3880.057	0.27079	16.83391	267.7479	4349.833	16.67162	0.064237
0.048445	31.88441	46160.6	0.881204	36.167	217.1716	8070.557	6.336454	0.031044
0.047048	34.59835	7357.231	1.400298	22.07553	201.0792	4372.573	13.99621	0.071851
0.053894	26.39246	4613.984	1.136148	31.24063	150.6582	4860.737	5.331934	0.038749
0.048174	31.92474	17619.88	0.689025	1895.038	227.2377	430992	0.122103	0.00065
0.051028	28.83832	1152.514	0.626443	35.95475	157.7569	5558.187	4.630717	0.030275
0.065011	18.5336	1341.37	1.630362	71.37497	94.6325	6897.132	1.36419	0.016708
0.084606	11.18751	236.6613	2.534033	52.73277	58.86213	3194.241	1.238903	0.028758
0.052451	30.29079	2157.061	1.103957	29.56465	160.2331	4998.559	5.551104	0.042985
0.051031	28.92035	13057.96	0.961506	48.06242	196.7044	9275.018	4.472669	0.022761
0.049339	29.84666	20200.6	0.66225	26.09524	185.9672	4816.908	7.568599	0.044008
0.057807	22.48622	2122.486	0.592797	21.43818	131.3468	2774.119	6.599911	0.05322
0.060804	23.08819	488.8003	1.260991	29.13968	115.9595	3818.096	4.029687	0.051323
0.055577	30.2783	1911.873	2.765929	53.44697	145.9461	7666.614	3.259017	0.025822
0.048518	33.07951	7068.406	1.446887	44.56515	182.1229	7702.028	4.772887	0.030496
0.04668	34.88947	15631.79	0.423449	42.85779	242.8182	10417.8	5.812335	0.024605
0.049065	30.62793	12644.39	1.503542	64.02273	170.1033	11364.99	2.663946	0.018634
0.040886	45.38219	7031.871	0.55898	35.37943	241.7654	8570.631	6.939947	0.030769
0.059687	22.89981	802.2749	1.913886	43.87115	104.321	4672.176	2.582104	0.034159
0.069078	17.87642	3241.758	3.9186	73.16037	86.07595	6239.381	1.426606	0.018666
0.060343	22.02686	341.1466	1.419044	36.65817	104.4964	4010.773	2.90418	0.038214
0.043292	54.19322	7799.551	0.459096	15.40471	324.5145	5083.083	21.61283	0.079233
0.054174	26.88947	2036.811	1.801673	63.8043	139.5643	9110.214	2.27693	0.018602
0.057466	22.96249	2126.785	1.408652	52.09088	111.0165	5878.267	2.180515	0.023291
0.043778	39.85112	23808.65	0.56296	41.43582	278.3389	12021.36	6.765041	0.025644
0.049421	35.37932	2584.401	1.301886	39.94703	172.2729	6692.398	4.673902	0.03118
0.042678	40.58918	32389.55	0.686147	1290.028	266.2583	343873.6	0.206428	0.000809
0.053034	26.94369	4074.588	0.939	35.88483	141.2269	5185.431	4.025911	0.033348
0.059023	22.55433	2117.576	1.572539	43.9717	117.6348	5347.432	2.78595	0.027417
0.076451	13.36985	231.7608	1.033659	18.62077	78.32555	1475.613	4.845174	0.071958
0.056056	24.08631	6471.864	0.80628	48.79305	121.2092	5844.789	2.575785	0.022608
0.0553	24.50853	5622.444	0.964285	34.3528	125.268	4355.046	3.780566	0.034827
0.067964	19.75106	661.1011	1.34111	56.37594	90.15957	4698.128	1.805336	0.020775
0.047692	32.96079	35939.91	1.390308	43.8665	220.3264	9241.328	5.626269	0.025447
0.05372	27.65704	2077.343	1.142279	35.10488	126.1445	4609.319	3.657155	0.035714

0.050159	29.61553	11494.32	1.113134	47.75271	162.463	7999.811	3.480409	0.02429
0.044692	38.58743	4856.675	18.74861	73.31964	177.6017	8263.797	3.998911	0.023297
0.059065	22.14479	2055.788	1.269697	50.39909	105.7863	5529.867	2.099774	0.022835
0.047395	33.76789	3134.368	0.8	31.5327	188.5047	5985.311	6.239398	0.0369
0.050957	28.09346	12384.3	0.854548	64.89122	151.5926	9918.907	2.362782	0.016589
0.048688	35.1356	2008.169	0.829947	32.81	150.2022	5210.498	4.458334	0.035442
0.045653	37.32093	10083.97	0.872459	43.9144	208.9439	9561.465	4.738962	0.02548

0.052952	28.34733	3456.768	1.618095	38.51493	130.34	4999.168	3.639258	0.034896
0.051392	28.42687	173979.2	1.84257	75.36849	180.1964	13080.32	2.540867	0.01429
0.045119	38.11393	6705.239	1.150327	42.91857	176.5169	8328.748	3.870444	0.028785
0.062948	20.31196	1449.13	1.341273	45.1267	99.58883	4701.571	2.241929	0.027369

0.049623	30.6579	144123.5	0.466953	154.2409	261.5252	40873.19	1.686045	0.006577
0.046833	34.30347	16500.44	0.948147	50.93449	204.4705	10526.91	4.195334	0.021623
0.051584	27.91537	20008.35	1.114895	39.72587	172.1373	6587.474	4.921572	0.028898
0.073018	14.49101	322.5234	1.21406	41.74011	78.49101	3306.711	2.006894	0.029404
0.051585	28.99801	4449.402	1.242419	48.67208	149.7076	7410.11	3.162519	0.0239
0.04448	36.57479	26245.36	0.818882	38.10995	212.9702	8108.831	5.80112	0.029518
0.053139	26.18539	9411.751	0.884367	32.24731	134.8847	4397.531	4.369599	0.036022
0.04631	41.9257	2430.809	0.600968	24.338	195.9129	4863.824	8.075156	0.048457
0.050898	29.84588	2657.786	0.945329	35.29464	163.0391	5874.971	4.793225	0.033864
0.042499	44.26689	19554.01	0.980075	32.6463	277.251	9029.511	9.77872	0.035962
0.076931	13.66242	178.0571	1.344986	31.42063	75.59683	2446.275	2.62903	0.0438
0.047323	33.42682	36617.95	0.425193	21.48556	236.9	5120.318	11.5875	0.050931
0.047135	33.21267	10090.36	1.16695	49.37387	186.8866	8894.968	4.180019	0.022758
0.118066	6.748054	74.60294	4.25981	34.89522	33.84926	1318.244	1.546619	0.065157
0.053046	26.75246	5551.968	1.463641	42.44327	148.1108	6187.374	3.840952	0.028549
0.047929	32.70184	24202.62	1.029113	49.24913	217.3877	10340.21	4.80149	0.022205
0.054667	25.47244	6661.539	0.994244	21.28323	158.0272	3187.359	9.374955	0.058355
0.058128	22.16189	6982.61	0.782685	43.46498	135.7304	5874.046	3.240415	0.025123
0.07992	13.23631	317.2934	2.319881	40.2784	76.7578	3558.258	2.002511	0.037147
0.05013	29.65217	4284.199	1.296079	54.33341	146.1807	8250.747	2.683033	0.022079
0.044385	39.91905	4172.644	1.75084	37.8208	183.6209	7343.625	4.951073	0.038133
0.052868	28.30555	2475.981	1.832769	69.5478	125.7347	8728.843	1.873998	0.01719
0.046113	38.01722	2610.751	1.837633	51.22988	162.5535	8486.944	3.396938	0.024288
0.137042	5.076455	91.37011	0.713314	11.69039	44.70819	515.1993	4.744723	0.116805
0.050592	30.57384	4857.294	0.460196	7.507916	211.501	1488.04	35.68952	0.173555
0.070707	19.02472	304.3457	0.822228	8.066047	104.1249	679.5459	21.30871	0.210499
0.047893	32.24876	19612.6	0.597501	1899.053	248.9795	473820.9	0.132068	0.000626
0.044207	38.06229	58290.02	0.509686	51.53232	269.9722	13711.2	5.432034	0.020287
0.042687	45.34696	7153.926	0.353674	33.36888	294.2467	9923.94	8.934938	0.031515
0.046361	34.94054	15128.35	0.689254	25.5863	251.921	6046.525	11.54726	0.04372
0.043716	38.38253	4174.376	0.423739	11.67984	231.5989	2349.516	24.02939	0.099592
0.043783	39.56644	2640.623	0.723123	43.97231	192.2182	8990.884	4.190092	0.025545
0.041858	45.39121	5387.846	0.309563	17.73569	281.6234	4671.866	17.27548	0.061257
0.045555	36.16923	12017.5	0.740438	69.94789	228.987	16083	3.310379	0.015406
0.051492	28.07601	56647.46	0.569319	58.57562	194.8038	11546.78	3.36384	0.017898
0.049325	34.11608	14726.91	0.908344	18.18552	211.0098	3474.03	15.32391	0.06919
0.044329	39.37409	2475.062	0.601268	17.04757	196.9825	3264.739	12.43104	0.07667
0.044601	36.5947	4785.948	0.419311	6.484024	227.9831	1290.365	47.83951	0.212701
0.053257	26.88903	1661.183	0.713767	23.46097	151.6276	3435.558	7.082717	0.049362
0.043264	38.89859	42423.96	0.768632	294.0715	239.0284	69316.5	0.828721	0.003495
0.069106	16.60793	602.6201	1.92735	54.16625	91.52208	4918.93	1.860543	0.023729
0.046606	33.81142	25801.49	1.671844	67.35325	181.3184	11919.5	2.802568	0.016378
0.068295	30.97984	474.4853	1.732387	67.54902	158.2418	10424.65	2.508806	0.020904
0.069894	17.70178	1736.515	0.827934	50.78153	88.25121	4448.828	1.796186	0.022566
0.042191	50.67745	3863.473	0.62155	45.94106	245.4529	11874.15	5.131905	0.023808

0.058778	53.16281	38477.66	0.411868	47.39613	418.2489	19914.38	8.890065	0.022264
0.043784	38.07926	61024.76	1.018725	64.00298	243.9006	15580.71	3.978822	0.017032
0.044383	47.42759	68256.9	0.560897	41.27518	335.7772	13211.01	8.760261	0.025599
0.042612	54.83605	47243.26	5.685796	57.35066	309.4254	15249.75	6.328742	0.021081
0.053046	26.78186	16834.58	0.318159	12.42031	235.7349	2807.138	21.50223	0.089287
0.048788	30.77726	6395.31	0.467001	22.6069	196.5617	4555.416	8.855166	0.048659
0.049045	31.13664	4875.299	0.642198	32.02504	206.684	6406.982	6.95325	0.034611
0.047466	32.572	19097.01	0.602449	1891.982	253.5867	476508.4	0.137203	0.000649
0.051009	36.17196	2114.677	1.063547	15.12829	170.4172	1904.996	17.12586	0.088223
0.057146	23.73464	2434.113	0.336148	6.59607	182.6527	1198.182	32.86506	0.193776
0.048297	32.20027	5770.436	0.555159	39.32127	207.5359	8076.671	5.464708	0.027845
0.049306	32.09829	13965.15	0.385519	20.08822	227.7666	4197.128	12.92046	0.053858
0.071282	22.34087	1063.024	0.933067	19.86292	124.0876	2176.464	7.679966	0.062973
0.043778	40.37816	3035.766	0.294504	10.06694	226.3391	2151.673	24.60612	0.117347
0.04294	41.33668	21910.29	0.425026	19.43006	287.5603	4992.942	17.05675	0.055954
0.049336	31.51835	1442.917	0.681397	36.27397	174.8528	6231.552	5.059052	0.030996
0.039875	51.37544	4636.421	0.560398	16.27315	273.7724	4271.826	18.23378	0.070689
0.04361	43.14168	47310.67	0.502297	55.47424	324.5044	18278.4	5.917142	0.018821
0.043623	38.0482	24825.1	0.733115	57.2033	231.9589	14042.85	3.90773	0.018848
0.04451	37.73716	13525.83	0.528151	27.42132	240.0957	6433.423	9.215804	0.041973
0.040931	48.40872	14640.84	0.507309	35.36218	260.4181	9320.746	7.364606	0.03042
0.050972	28.49243	29480.79	1.962922	247.3076	166.3755	41429.49	0.683053	0.004199
0.044212	37.56922	22333.83	0.384361	22.15099	257.7229	6124.65	11.18574	0.049943
0.118117	6.535272	100.5514	3.258099	48.54507	38.69392	2109.069	1.248255	0.04824
0.046777	36.52976	3100.23	0.534253	36.18249	164.1076	5901.35	4.617886	0.03027
0.041641	47.6818	36340.25	0.43288	49.12547	279.9204	13767.46	5.738658	0.021324
0.04178	47.93321	48512.25	0.930841	63.81703	297.1063	19129.67	4.839592	0.019525
0.052958	29.18703	5709.646	7.180059	61.46086	135.8323	9777.568	4.061553	0.042011
0.129984	9.197318	109.5154	152.7784	322.9639	26.16466	2063.995	0.784059	0.025815
0.064919	17.86494	353.1099	1.044021	42.49113	96.99291	4005.092	2.453441	0.02863
0.041699	55.04957	11175.2	3.134869	58.67865	284.6995	18031.48	5.913589	0.041008
0.045746	36.64008	1960.656	1.379795	69.20286	174.6402	11659.04	2.682167	0.017717

DX68	DX69	DX70	DX71	DX72	DX73	DX74	DX75	DX76
0.021716	0.899231	0.00068	4210.479	0.324234	0.790946	42.12168	7.419254	300.7202
0.013224	0.237872	0.001116	4913.958	0.344108	0.651348	15.33816	14.2499	175.3807
0.013557	0.325365	0.000825	6595.375	0.425352	0.451962	21.20904	10.17858	216.5061
0.02983	0.347608	0.005491	234.9722	0.427417	0.498107	10.18765	9.417621	87.91304
0.029685	1.37671	0.001543	824.531	0.277229	1.616162	50.93021	5.588347	297.5784
0.020329	0.474089	0.001469	3281.349	0.313405	0.887022	22.56779	8.437279	177.9049
0.038094	71.07903	9.12E-05	1364.778	0.267696	5.269778	1881.746	4.146923	7781.763
0.028351	0.810171	0.001554	2596.297	0.271108	1.107385	30.64013	5.389082	158.5173
0.03603	0.59596	0.006005	201.8718	0.251535	1.215338	17.2821	4.843849	81.51484
0.03125	61.48228	3.10E-05	6472.082	0.297203	2.145204	1983.819	5.357669	10678.25
0.019508	1.014215	0.000462	26441.6	0.299549	0.872764	54.84029	8.104288	444.8707
0.012225	0.384023	0.000492	61195.29	0.309055	0.8384	30.68446	12.17202	352.6997
0.021709	0.614879	0.001443	5114.046	0.285539	1.067348	29.10333	8.253286	244.6936
0.026803	0.640508	0.002359	974.5886	0.276979	1.069358	26.43676	5.249019	135.5615
0.030878	0.798708	0.002648	380.893	0.287016	1.088203	24.16363	5.111465	119.9787
0.018128	0.631457	0.000669	14207.54	0.321247	0.765474	36.23742	12.37753	440.9861
0.022773	0.418798	0.002141	696.3452	0.352368	0.62732	17.45325	8.227994	135.3465
0.021384	1.013177	0.000672	5563.375	0.247145	1.384587	42.82607	6.717819	275.3648
0.02299	0.723326	0.001328	4597.229	0.28273	1.17371	35.21247	6.869083	253.255
0.019577	0.943542	0.000541	15996.43	0.283768	1.13619	50.12868	7.145681	354.379
0.0168	0.57581	0.000682	20874	0.250436	1.168016	33.50417	7.563431	249.8364
0.029952	1.435542	0.001097	6827.927	0.233739	1.743452	48.01624	4.856329	229.9999
0.027803	0.792903	0.001746	715.6588	0.295998	1.02983	31.10573	7.206737	226.2695
0.01396	0.547743	0.0005	14382.18	0.284128	1.022542	35.16485	11.10148	379.7076
0.018343	0.816423	0.00064	6563.615	0.317267	0.95714	50.08035	11.27802	552.1579
0.024562	0.491769	0.00214	875.6453	0.398858	0.588584	19.44023	7.303608	130.3199
0.015889	29.01139	6.33E-05	3734.649	0.438054	3.257782	1855.447	25.63225	47424.79
0.020427	0.658915	0.000988	3832.052	0.241028	1.351329	30.61886	7.636977	229.7523
0.027294	0.99139	0.001139	1781.522	0.290289	1.030287	41.10351	6.805908	278.9305
0.027841	0.867303	0.00167	2441.465	0.247079	1.391916	34.11377	5.565236	193.8303
0.028201	0.36902	0.003863	721.3005	0.392107	0.506545	12.67126	5.199652	62.76458
0.01868	0.473788	0.001073	10160.98	0.296503	0.894346	24.57398	7.768294	188.0641
0.031989	1.22505	0.001337	867.3281	0.321371	0.833715	35.71723	5.409551	190.8427
0.017227	0.744348	0.000571	9184.798	0.255692	1.177246	45.34355	10.26698	473.0496
0.014806	0.413879	0.000946	55503.17	0.312992	0.980312	20.1154	11.97056	244.1603
0.017764	0.376777	0.001098	1911.555	0.455987	0.388306	20.75887	8.575983	196.9668
0.014203	0.462575	0.000661	19922.76	0.232616	1.432957	27.26782	10.20873	255.0416
0.029598	0.522089	0.003629	564.8247	0.355046	0.6614	14.36263	5.531416	75.7588
0.023355	0.969901	0.000886	1710.841	0.410261	0.560625	41.4115	6.311947	261.5457
0.031368	1.155807	0.002304	480.2007	0.237777	1.913862	41.71156	6.732814	278.0237
0.029014	1.458572	0.000989	3462.055	0.285437	1.31818	54.53749	6.611962	375.3471
0.019972	1.1622	0.000443	27512.93	0.223445	1.738694	58.2876	8.367249	495.8437
0.026902	1.229737	0.000858	1811.163	0.20693	1.801815	50.15482	5.047988	249.9108
0.014724	0.710947	0.000422	15760.9	0.302347	1.111523	41.70989	12.40829	493.8163
0.017534	0.893021	0.000433	9886.018	0.349166	0.721542	49.88589	10.51021	511.0037
0.020015	0.207577	0.003513	1166.658	0.374641	0.539717	10.23608	9.878345	96.24551
0.017908	0.260505	0.001817	1701.861	0.36469	0.569496	16.1598	10.10422	163.4432
0.018666	0.529974	0.001041	6627.781	0.244428	1.347677	27.28279	8.272918	223.0698
0.017824	0.419168	0.001336	5373.841	0.288133	1.008899	20.56635	7.589717	154.3405
0.01943	0.503602	0.001622	4137.537	0.284533	1.235429	20.24867	11.90982	240.1842
0.019222	0.651365	0.000822	12233.34	0.358162	0.681296	38.55583	9.228346	352.9698
0.027805	0.660403	0.003242	924.1929	0.389258	0.845891	14.04066	8.357941	123.0397
0.027994	52.89434	5.39E-05	1528.139	0.31608	2.04419	1935.201	6.121413	11875.97
0.022549	0.885094	0.000935	2792.747	0.298961	1.04943	35.96399	9.537048	337.0548
0.023008	1.342044	0.000636	14533.32	0.278265	1.531781	52.71733	9.165154	493.5468
0.025215	1.794049	0.000957	3999.506	0.24048	3.277237	77.62255	9.440305	760.9761
0.02078	0.456325	0.001635	2782.673	0.320606	0.795976	23.69942	9.353493	226.2642

0.032087	0.547773	0.003288	1054.945	0.344497	0.696432	15.94957	6.125256	96.34742
0.031475	1.298224	0.001283	747.5069	0.241386	1.398298	41.74826	6.071145	242.2326
0.026425	0.836938	0.00128	2715.292	0.288007	1.072557	36.19833	7.943554	292.6939
0.012889	0.714204	0.00028	68128.89	0.276554	1.078105	54.49936	11.39374	597.2889
0.029218	0.629171	0.002836	945.9496	0.265139	1.180685	20.95483	5.982715	124.7963
0.023366	0.714889	0.001164	4663.464	0.328414	0.873323	32.47605	12.70781	444.0948
0.020777	0.918077	0.000615	12556.27	0.330934	0.783067	43.78334	12.16477	563.5759
0.022254	0.603474	0.001208	9070.431	0.291565	0.986619	26.15484	6.747443	178.4957
0.024629	0.532772	0.001842	1326.635	0.374215	0.635143	20.92736	5.601728	114.1563
0.031777	0.490275	0.004134	494.5793	0.32223	0.775789	16.82104	4.997464	82.32656
0.026971	0.626319	0.002154	529.1263	0.320801	0.782161	20.71981	6.925021	132.0889
0.024713	0.728185	0.00125	10117.37	0.272062	1.039499	28.9845	5.66445	156.8612
0.018986	0.605882	0.000804	54165.01	0.335448	0.696251	30.73285	7.081438	217.5719
0.015089	0.235635	0.002107	18695.75	0.256923	1.115679	14.79445	9.788377	134.2475
0.019978	1.281416	0.000689	28855.3	0.283371	2.964022	39.75171	10.50132	364.5978
0.017638	34.38417	1.83E-05	18496.96	0.339733	1.746734	1922.199	15.31963	29497.3
0.027624	0.214442	0.007525	351.771	0.430963	0.465345	7.41011	9.931514	82.6419
0.02067	0.391258	0.001781	2645.444	0.274047	0.981076	21.19513	7.484599	165.156
0.828039	0.828039	0.828039	8815.846	1	0	1	1.031207	1.031207
0.027074	1.094635	0.001213	2088.115	0.274735	1.426938	41.67221	5.582606	236.1347
0.023349	0.755825	0.001156	10993.51	0.280813	1.058829	31.19954	6.984663	209.9228
0.018983	0.223397	0.00301	3575.336	0.391719	0.506009	12.26306	10.63502	123.4677
0.043194	2.478093	0.001332	8524.59	0.160601	2.918479	53.73706	2.828627	149.4643
0.019879	0.588338	0.001071	6634.899	0.242311	1.415942	31.98367	11.29909	381.7614
0.036062	1.755636	0.001022	3134.184	0.296728	0.91908	49.92133	3.906053	195.7109
0.017421	0.478176	0.000923	15181.69	0.336442	0.780679	26.72477	8.938394	232.3213
0.015819	0.379864	0.000875	45489.82	0.345181	0.63125	24.26492	10.50622	253.9581
0.030522	0.741322	0.001876	4680.976	0.342767	0.656798	23.78183	5.79048	138.7556
0.031097	1.185492	0.001241	11248.91	0.298426	0.899786	36.72031	4.493038	162.0127
0.026366	0.768459	0.001581	3159.216	0.281854	1.080487	33.2963	7.78759	267.7512
0.035505	1.539121	0.001543	476.171	0.342261	0.908711	46.42757	4.704584	221.3595
0.027915	1.094109	0.001082	5359.642	0.265389	1.255355	43.67608	7.527428	347.5019
0.019137	0.973558	0.000497	15257.51	0.297186	0.916649	51.09868	7.699279	361.3181
0.015868	0.532262	0.00145	11894.89	0.300793	2.036847	18.17939	15.4776	242.2444
0.024662	0.842014	0.00134	2866.742	0.2718	1.176132	37.70823	7.06764	277.1388
0.017943	0.778938	0.00057	9818.33	0.195784	1.984998	43.39754	8.300821	349.5131
0.030548	1.195973	0.001241	1155.797	0.32443	0.885106	36.85957	5.737956	206.8496
0.01811	0.5175	0.000848	7952.314	0.341765	0.724043	26.84816	13.63577	350.7164
0.030528	1.242721	0.001291	612.1976	0.342602	0.923456	41.74038	6.340878	276.1658
0.025111	0.316909	0.003628	863.3311	0.338551	0.672718	12.50478	5.802726	72.22186
0.028472	0.874758	0.001604	2003.028	0.242348	1.332727	31.21721	5.221188	161.6341
0.018031	0.59315	0.000745	6813.545	0.31023	0.888814	33.2702	9.073889	304.1902
0.023677	0.375387	0.003128	1278.535	0.29694	0.999467	15.23402	7.344575	105.958
0.019299	0.700027	0.000723	11749.21	0.338095	0.768394	38.63795	8.121002	310.8431
0.030646	1.068504	0.001492	2739.144	0.290594	1.108706	31.58183	6.259083	197.4677
0.021919	0.514387	0.00144	2134.059	0.314119	0.890854	26.31637	8.707455	232.9601
0.037263	2.138684	0.001004	1989.215	0.304451	1.132193	50.1099	5.461105	267.8147
0.020319	0.700002	0.00101	5073.213	0.389893	0.870931	40.30738	6.898061	286.5248
0.019953	0.38265	0.001942	1526.366	0.400794	0.583394	19.05204	7.753237	140.0512
0.023075	0.536367	0.001611	2168.483	0.369932	0.620074	23.05943	7.879523	188.8415
0.017386	0.649802	0.000608	16840.07	0.309237	0.897561	36.17705	8.636158	307.3974
0.016095	0.705854	0.000848	34219.17	0.288715	1.763533	25.94894	10.39209	255.0901
0.02208	1.218291	0.000571	14507.24	0.297187	1.289332	47.7112	9.026609	421.6377
0.019116	0.21869	0.003111	2910.358	0.380598	0.527835	10.6938	10.03308	97.91592
0.017165	0.268592	0.001951	3528.923	0.347404	0.67739	14.43942	9.486877	126.9707
0.01789	33.23755	3.68E-05	19836.42	0.315649	4.930988	1847.691	10.46469	19141.72
0.026498	0.858035	0.00121	3398.808	0.300141	1.053449	32.52956	6.183089	200.2164
0.018963	0.937062	0.000461	10935.07	0.377608	0.543923	51.41407	8.736325	439.9158

0.037652	1.350538	0.001674	3830.038	0.256788	1.197834	32.05237	3.422907	105.9939
0.023994	0.350834	0.003181	2147.798	0.302473	0.828226	12.86031	6.606294	77.73956
0.026661	1.52231	0.000782	20555.49	0.311473	1.41729	51.25958	8.268316	413.5891
0.018884	0.647623	0.00082	2128.765	0.358293	0.802234	32.4894	9.633912	327.0825
0.047855	1.353468	0.002445	880.6422	0.373948	0.56504	27.72369	3.380856	91.43758
0.014245	1.532924	0.000296	18384.06	0.27529	2.735925	56.15684	14.65417	756.6852
0.022653	0.432141	0.002051	1268.808	0.298325	0.857812	16.72991	6.985684	112.3407
0.021524	0.650373	0.001039	6751.361	0.375573	0.588231	31.75851	9.381307	310.8759
0.027629	0.536518	0.002229	982.4743	0.356742	0.611858	19.08965	6.452079	112.9792
0.016345	0.332681	0.001171	3997.451	0.371689	0.576763	20.49784	11.86464	227.9952
0.016548	0.351396	0.001231	6772.873	0.303985	0.856804	21.07176	9.652507	208.427
0.033779	0.939396	0.00205	2228.389	0.270754	1.224245	30.58563	4.666267	148.9313
0.017012	0.529023	0.001005	8404.397	0.289987	1.212801	29.27075	9.817514	276.2781
0.014458	0.629297	0.000434	12767.48	0.355499	0.733047	44.94277	20.23978	955.2106
0.024378	1.260106	0.000646	12066.49	0.31174	1.123427	53.17267	6.975136	365.4411
0.022481	1.563796	0.000426	3568.86	0.257488	1.424901	77.56579	7.760254	632.0724
0.029504	1.188136	0.001111	7692.402	0.253272	1.286005	41.71433	4.886178	199.5747
0.014427	0.534798	0.00062	42695.63	0.307098	1.174718	28.37876	13.40822	364.5369
0.01608	0.210088	0.002011	2035.601	0.382868	0.538745	11.80646	13.02741	139.7874
0.020224	0.549902	0.001302	2926.9	0.336755	0.795418	28.93742	11.51064	316.1135
0.022269	0.524756	0.00142	1544.796	0.290528	0.892034	21.59377	6.920914	142.536
0.023937	1.030613	0.000756	4728.742	0.312544	0.912221	47.88053	8.904267	433.2028
0.021268	0.808163	0.001103	2466.694	0.268512	1.179403	37.49395	9.607045	360.5054
0.027097	0.446857	0.002446	1271.046	0.407266	0.476055	17.30881	9.889566	161.9997
0.016163	0.562097	0.000699	22339.19	0.277295	1.021986	35.67388	10.16569	372.5628
0.019895	0.493366	0.001744	3271.224	0.246771	1.532086	22.35494	9.630346	208.9265
0.030063	0.802986	0.0017	2481.158	0.250408	1.274102	30.80085	5.424459	171.8068
0.020961	39.09915	1.90E-05	7929.345	0.386641	1.009581	1893.393	7.670977	14550.5
0.032194	1.24302	0.001388	593.364	0.316428	0.808599	36.32777	5.500718	195.4078
0.050203	3.501048	0.001539	838.5817	0.240788	1.947198	70.97217	3.277079	236.151
0.065989	3.282857	0.003817	169.0559	0.1856	2.847878	52.29322	2.488223	132.7836
0.027	0.735657	0.001981	1108.048	0.257207	1.384426	28.74669	7.434214	228.8891
0.019914	0.964102	0.00053	6543.631	0.267222	1.104645	48.52484	7.971707	380.2092
0.026155	0.672101	0.001644	9400.865	0.31059	0.868182	26.13487	5.853224	151.653
0.043416	1.031675	0.002992	1206.981	0.331336	0.735295	21.6311	3.763023	79.98957
0.03701	0.990556	0.002641	275.2864	0.27416	1.488298	27.85143	5.531069	172.1891
0.029266	1.691243	0.000974	1079.962	0.167861	3.219952	54.01932	6.82725	361.0181
0.025136	1.390221	0.001066	3510.665	0.267178	1.899373	45.82385	8.047096	341.5399
0.01816	0.737009	0.000626	6935.059	0.400546	0.572117	42.67531	12.07426	522.2745
0.027095	1.498856	0.000736	5774.53	0.249636	1.98282	62.44379	5.678619	368.9838
0.025648	1.042071	0.000989	2507.465	0.332556	0.92134	35.39067	11.21466	400.0633
0.05561	2.260527	0.003794	473.89	0.222828	2.326483	43.47636	3.364705	149.1568
0.052455	3.990751	0.001189	2213.909	0.143153	4.267887	73.40829	3.476953	251.3185
0.048113	1.562492	0.003969	200.9265	0.230189	1.759081	35.9972	3.771917	140.1683
0.018581	0.264932	0.002556	2026.337	0.328851	0.817547	15.13741	20.10817	314.8416
0.033951	2.084164	0.001035	1158.784	0.218384	2.259804	63.1407	6.489897	417.2657
0.053384	2.484447	0.002422	1230.302	0.24098	1.771012	51.61097	3.245945	170.2606
0.016543	0.696451	0.000513	9831.85	0.370987	0.667605	40.12404	13.69024	588.355
0.028485	1.121857	0.0014	1160.207	0.23139	1.788191	40.24962	9.085522	356.4389
0.01872	23.87011	2.65E-05	11437.8	0.327306	1.132581	1288.536	11.2841	14573.2
0.036899	1.30603	0.001835	2131.004	0.287825	1.210208	35.47507	4.606908	166.9761
0.036884	1.433809	0.001739	1241.498	0.209047	1.873431	43.4431	3.926961	174.0782
0.050647	0.910849	0.006192	158.8126	0.253898	1.161368	18.67788	3.513621	63.92532
0.046642	2.446519	0.001363	3681.875	0.303009	1.028498	49.05907	3.54639	172.1191
0.043585	1.486201	0.002399	3105.375	0.275612	1.218004	34.21386	3.779354	130.7004
0.048882	3.013279	0.001486	424.5834	0.2472	1.581236	57.36623	3.3134	182.4325
0.019427	1.424338	0.000518	16907.22	0.262633	2.085371	45.48643	10.16158	431.3566
0.047801	1.503954	0.003052	1147.43	0.265539	1.423525	34.55162	3.983744	142.4578

0.0302	1.291759	0.00114	6042.411	0.270716	1.393186	46.90856	5.783899	279.752
0.047414	16.97834	0.000978	2192.139	0.255954	32.17046	95.12914	7.193571	389.7985
0.05439	2.426926	0.001902	1202.631	0.241801	1.544986	49.62166	3.158497	161.5914
0.025256	0.787206	0.00143	1481.915	0.296574	1.083109	31.2776	8.025452	256.8321
0.037589	2.406512	0.000806	6301.884	0.299154	1.133254	64.53053	4.371401	284.2334
0.046964	1.383705	0.00287	987.0634	0.285116	1.104527	32.01167	4.582701	155.1618
0.021709	0.873069	0.000874	4347.885	0.28737	1.210552	42.76119	10.76048	491.3107
0.042959	1.58561	0.002841	1820.158	0.219581	2.06631	38.47956	3.970963	152.244
0.032436	3.700083	0.000477	84093.67	0.291573	2.854872	77.38858	5.239612	385.6717
0.035541	1.285094	0.002024	2934.955	0.276545	1.553417	40.7159	6.300959	287.0335
0.050036	2.11209	0.002614	877.1394	0.254454	1.602062	44.5183	3.624694	166.1865
0.012414	1.739136	9.93E-05	60387.72	0.411643	0.642152	152.3835	10.97066	1712.401
0.020272	0.994118	0.000583	8185.044	0.281894	1.167842	50.48141	9.238794	474.1321
0.028462	1.051755	0.001258	10919.04	0.2503	1.281345	40.14196	5.552954	218.174
0.050402	2.083538	0.002554	216.7931	0.263919	1.414504	41.67504	3.376804	141.3647
0.032847	1.484065	0.001243	2276.696	0.248023	1.613473	48.15759	5.676836	278.9678
0.025029	0.873585	0.001103	10870.99	0.282805	1.145951	37.98811	7.649914	288.6857
0.042337	1.29249	0.002368	5230.006	0.276169	1.100393	32.06581	3.955438	128.3023
0.034061	0.840612	0.002822	975.0093	0.323652	0.910987	24.02158	9.378159	232.9082
0.03083	1.070853	0.001411	1291.824	0.293144	1.223937	34.98745	7.104182	251.6074
0.015191	0.497434	0.000665	6913.585	0.261993	1.180862	32.7549	15.13649	502.3303
0.045307	1.309824	0.00402	119.312	0.256743	1.533214	31.16054	3.301497	105.5857
0.019354	0.436335	0.001153	15860.4	0.377842	0.551194	21.42668	9.27505	201.6788
0.025493	1.314343	0.000764	5178.542	0.245699	1.444409	50.12578	6.929385	337.6193
0.102584	3.334168	0.007119	60.56955	0.132312	4.41836	34.47339	2.233109	78.6857
0.03306	1.445954	0.001331	3003.36	0.231583	1.821694	42.71353	5.012229	210.3914
0.017843	0.913675	0.000469	10858.35	0.270112	1.3186	50.10265	11.06135	544.1764
0.025892	0.608047	0.001794	3778.281	0.26546	1.099385	21.78511	5.603342	117.2373
0.039704	1.828487	0.00127	3867.36	0.315578	1.006577	43.59447	4.055735	176.0912
0.042278	1.376739	0.003078	220.1371	0.186321	2.515186	38.59604	3.93852	168.5454
0.051249	2.331544	0.002111	2231.905	0.265426	1.721423	53.24726	4.036961	223.5579
0.032283	0.872728	0.003619	1850.885	0.205832	2.373129	36.19255	7.248481	282.4305
0.054702	3.509573	0.001728	1371.921	0.22027	2.420218	69.36633	4.00909	279.5586
0.045154	1.72114	0.002222	1392.13	0.197921	2.282842	50.16404	5.452082	280.3705
0.049801	0.531565	0.009859	71.30261	0.314514	0.769886	11.6335	3.357642	40.89303
0.018295	0.257663	0.003465	2319.357	0.399632	0.625188	7.905652	9.49443	68.55934
0.031734	0.315389	0.007967	192.7208	0.286368	0.931001	8.550435	4.947177	36.00509
0.015641	29.44702	2.22E-05	8433.228	0.413542	0.934896	1896.318	10.15237	19331.51
0.016002	0.796903	0.00038	21143.92	0.33704	0.722143	51.98826	11.79794	600.9169
0.022447	0.715903	0.000998	2456.486	0.394727	0.546864	32.99923	12.46596	420.6754
0.015884	0.422894	0.000882	6836.567	0.315816	0.777879	26.63575	11.22213	272.3418
0.025201	0.337953	0.003686	1687.794	0.380696	0.589406	12.67439	7.986454	84.69347
0.039553	1.518792	0.001765	1153.994	0.319374	1.024485	42.4377	5.852332	267.7385
0.017721	0.333495	0.001331	1768.461	0.403079	0.512625	18.77365	13.94394	232.6713
0.02822	1.55949	0.000867	5057.577	0.3417	1.168878	69.27323	7.972176	557.0736
0.025239	1.480877	0.000543	26519.52	0.338449	0.734349	58.297	5.733248	338.8489
0.015463	0.320008	0.001202	7396.907	0.276018	1.04129	19.10114	13.61523	236.0923
0.03162	0.529303	0.004287	1012.785	0.327373	0.906119	17.27239	7.810733	129.9158
0.021407	0.171866	0.006657	2181.454	0.384425	0.528743	6.999883	8.666962	51.21094
0.03854	1.074559	0.002409	867.1779	0.314789	0.945594	23.91551	5.425793	125.4938
0.02106	6.134833	8.45E-05	16792.07	0.301834	1.096123	296.5455	9.106327	2648.901
0.048723	2.509764	0.00252	398.9127	0.201471	2.25761	54.28428	4.519049	237.9237
0.033144	3.070336	0.000736	11405.24	0.294106	2.641033	68.34337	5.368998	355.1698
0.025122	1.746717	0.001139	225.0539	0.292972	2.589634	68.29457	10.77715	711.7427
0.050091	2.458383	0.001861	1108.328	0.320602	1.031421	50.7644	4.202744	212.9699
0.028322	1.20391	0.000886	1299.267	0.315428	1.001592	44.17403	12.81483	611.1453

0.020306	0.948886	0.000646	7224.852	0.360651	0.960425	47.10886	29.78868	1426.18
0.020354	1.241724	0.00045	25010.95	0.257877	1.339938	63.90064	9.903267	630.9442
0.01774	0.74964	0.000507	19051.66	0.304875	0.849734	43.11523	18.16352	713.4686
0.030143	5.977383	0.000719	11846.7	0.31132	13.13114	68.26831	14.09727	715.4563
0.014188	0.212409	0.001436	7763.653	0.454939	0.370327	12.80342	8.895052	107.0236
0.02351	0.535761	0.001577	2949.155	0.359104	0.621807	22.36546	6.536764	150.2494
0.019844	0.704052	0.001039	2262.57	0.348264	0.881459	32.65097	8.367871	263.7359
0.014889	28.519	1.54E-05	8119.681	0.417384	0.899928	1901.372	9.248122	17404.79
0.025242	0.506227	0.002438	1011.641	0.255225	1.231899	16.84567	8.942285	119.804
0.022248	0.153315	0.009087	1278.267	0.434668	0.395477	6.637551	5.775305	37.75923
0.017431	0.648539	0.000694	2542.481	0.355462	0.789545	39.39244	9.202487	355.9437
0.013644	0.297226	0.000831	6278.064	0.401549	0.480374	21.06044	14.33615	273.5107
0.026353	0.564335	0.002532	586.5558	0.264171	1.180557	20.74343	10.07406	185.6361
0.025758	0.230532	0.005165	1210.344	0.430359	0.455462	10.40722	8.43155	81.09117
0.014929	0.351964	0.001019	7335.876	0.38737	0.62242	21.18773	14.57789	262.8832
0.024333	0.940976	0.000898	684.4429	0.309342	0.921872	36.60136	6.535266	237.1542
0.022546	0.668187	0.001413	1506.354	0.329716	1.002431	17.29718	16.55627	260.884
0.016825	0.940932	0.000386	16359.53	0.35954	0.68742	54.76131	17.56783	994.7156
0.019688	1.022043	0.000525	9910.017	0.329252	1.045186	55.06569	9.196901	547.527
0.024145	0.590693	0.001849	5168.625	0.362154	0.820298	27.6859	8.72401	233.4615
0.028775	1.000084	0.001233	4746.377	0.331614	0.850545	35.04831	9.471213	338.0546
0.026379	6.283702	0.000129	15816.93	0.198972	2.291774	246.1066	5.367235	1329.348
0.017254	0.384699	0.001261	8342.603	0.4044	0.540066	20.97905	10.55574	246.4417
0.074519	3.020313	0.005003	77.61727	0.197507	3.450201	47.56971	2.345752	118.2713
0.040355	1.505595	0.001719	1440.348	0.347322	0.775992	36.28719	5.449073	196.809
0.021599	1.010439	0.000572	11055.06	0.34542	0.749011	49.03389	12.08906	592.4813
0.021836	1.213711	0.000663	13399.64	0.327206	1.665595	63.07233	12.91508	826.0286
0.035965	1.860077	0.002262	2834.033	0.1531	7.424629	57.80167	4.570225	302.1942
0.283169	185.351	0.003532	84.25632	0.062721	165.4779	358.884	1.813656	425.7623
0.050936	2.010359	0.00258	221.077	0.278262	1.296292	42.62653	4.058984	168.8168
0.022661	1.1072	0.001711	2759.675	0.247093	4.859868	54.3039	15.08419	936.7034
0.039565	2.585153	0.001065	861.8623	0.282002	2.016325	70.10042	5.940469	402.2201

DX77	DX78	DX79	DX80	DX81	DX82	DX83	DX84	DX85
0.191032	0.026013	3.580296	5854.988	0.416531	0.560401	2.919102	0.644401	27.45097
1.308784	0.079833	3.684985	5852.583	0.362179	0.489731	6.521539	0.579004	9.658351
0.507154	0.051616	3.5431	5250.333	0.319407	0.467716	4.238152	0.558886	12.0231
1.071823	0.124152	2.796334	249.5499	0.425896	0.584513	4.291648	0.618043	6.623302
0.117186	0.025459	3.720225	1444.029	0.451179	0.606227	2.007613	0.677062	33.84867
0.443195	0.053458	3.775472	4380.387	0.387057	0.532803	3.516152	0.622621	14.38178
0.007936	0.002825	3.577336	2571.729	0.487193	0.632034	1.3587	0.715575	1347.807
0.203491	0.038469	3.717524	4540.193	0.445889	0.595739	1.892398	0.67546	20.92187
0.360049	0.08959	3.492743	412.7306	0.481761	0.648944	1.659001	0.697957	12.13343
0.004052	0.001041	3.707863	9676.46	0.427919	0.573276	1.916677	0.667466	1321.58
0.154999	0.019584	3.919403	32857.62	0.355761	0.498574	3.30955	0.60041	32.77384
0.454696	0.036777	4.15726	65806.19	0.308995	0.443463	5.404837	0.544958	17.04709
0.308065	0.041908	3.967348	7288.961	0.380115	0.519804	3.367722	0.617725	17.88139
0.227965	0.046919	3.621687	1741.933	0.462813	0.612815	1.800995	0.684654	18.2786
0.247102	0.053778	3.449485	681.0505	0.478616	0.637693	1.74253	0.696149	17.12139
0.359498	0.030322	3.972769	15169.18	0.325367	0.439999	5.958615	0.573787	20.92054
0.540597	0.069206	3.370782	847.0971	0.398173	0.547277	3.376583	0.633034	11.3324
0.181127	0.026661	3.994611	9558.636	0.398222	0.549501	2.512237	0.633219	27.74506
0.211898	0.036273	3.845837	7076.907	0.406128	0.555109	2.620924	0.640362	21.93519
0.161306	0.02235	3.969138	21564.54	0.367104	0.514583	2.789448	0.610595	30.41644
0.26616	0.034954	4.073503	31820.99	0.361686	0.513196	2.89391	0.600295	20.3532
0.115792	0.025368	3.987368	13366.78	0.440528	0.592707	1.650226	0.676582	32.48656
0.248773	0.039676	3.664002	1081.694	0.417063	0.569594	2.826684	0.647694	20.07124
0.353114	0.03237	4.030718	20288.32	0.359366	0.50453	4.576561	0.583146	20.87427
0.239623	0.022674	3.938283	7700.542	0.347524	0.478228	5.288815	0.588655	29.35501
0.438471	0.062145	3.238338	949.6111	0.402556	0.557142	2.838244	0.636101	12.77229
0.014916	0.001232	3.705981	2443.306	0.271268	0.380204	14.26917	0.515141	956.4503
0.30258	0.040659	3.956046	7741.238	0.439155	0.580024	2.987886	0.656813	20.39442
0.176221	0.02786	3.745511	2675.48	0.408491	0.558797	2.622756	0.645386	26.51033
0.194239	0.039184	3.885586	4635.051	0.440602	0.591181	1.987485	0.671401	22.56279
0.483432	0.096095	3.111295	851.1627	0.438381	0.598536	1.74463	0.670771	8.701551
0.361257	0.048691	3.849112	13606.7	0.373715	0.523266	3.091281	0.611278	15.15783
0.160264	0.031308	3.431865	1216.284	0.435254	0.579994	2.000704	0.674494	24.3711
0.243746	0.025366	4.05617	14931.07	0.378138	0.516721	4.465591	0.607235	27.39729
0.650877	0.060082	4.091736	56228.57	0.30373	0.429748	5.498669	0.549517	11.23512
0.392082	0.053426	3.195738	1588.127	0.356423	0.507608	3.492712	0.593994	11.8902
0.513943	0.046749	4.363799	31133.73	0.337112	0.483508	4.25123	0.572139	16.23227
0.4591	0.087297	3.285727	755.4447	0.446623	0.603476	1.957605	0.674929	9.899434
0.157795	0.026107	3.307587	1673.983	0.382813	0.546594	2.250053	0.621447	25.58248
0.182172	0.033737	3.970353	955.5951	0.442363	0.584715	2.68815	0.671894	27.86
0.124584	0.021761	3.843909	5469.929	0.425237	0.567851	2.630874	0.660403	35.42414
0.156898	0.019731	4.298268	46706.14	0.363244	0.495899	3.587778	0.608861	35.56982
0.117329	0.023778	3.981502	4509.315	0.47534	0.629988	1.64605	0.689815	34.6117
0.330124	0.02678	4.141795	18704.42	0.330378	0.464075	5.646066	0.567412	24.31404
0.221825	0.021473	3.841395	9598.56	0.325942	0.451676	4.757106	0.574549	28.96524
1.113219	0.126844	3.423898	1243.843	0.3707	0.51064	4.310976	0.606449	6.323247
0.666552	0.073004	3.580187	1735.76	0.346209	0.491085	4.279893	0.583357	9.278049
0.404985	0.047202	3.989488	12428.44	0.414806	0.560744	3.406474	0.639707	17.38541
0.447683	0.063581	3.967194	7016.85	0.356836	0.509698	2.915526	0.598488	12.51544
0.715182	0.068074	4.066072	5567.02	0.350858	0.49344	5.635611	0.591775	12.09732
0.249263	0.028572	3.674613	12657.55	0.351589	0.482854	3.976252	0.594653	22.60561
0.620686	0.092121	3.306925	1023.632	0.407562	0.549779	3.725151	0.646995	9.039203
0.003959	0.001104	3.550993	2152.314	0.423773	0.564346	2.350146	0.661472	1277.173
0.283449	0.031806	3.863052	3804.794	0.379562	0.513661	4.335781	0.618855	22.51131
0.180239	0.021481	4.127186	19850.69	0.360104	0.495612	4.023081	0.605696	31.78763
0.14632	0.023438	4.288167	7086.369	0.397097	0.511385	4.358621	0.636647	48.68692
0.418115	0.050358	3.75518	3274.625	0.356475	0.50197	4.103745	0.599257	14.0341

0.427047	0.079116	3.381542	1401.329	0.437457	0.564767	2.425886	0.67355	10.86278
0.166038	0.028464	3.812773	1507.042	0.451115	0.600562	2.251127	0.677279	28.95594
0.231678	0.03235	3.766662	4203.034	0.413392	0.559634	3.364614	0.64749	23.00893
0.231715	0.020028	4.263562	85198.54	0.319816	0.465382	4.861072	0.553432	30.59249
0.341997	0.064293	3.664407	1811.336	0.467782	0.620873	2.150927	0.685627	14.49356
0.379452	0.036171	3.788002	5354.864	0.359407	0.462535	6.434757	0.61049	19.12306
0.272242	0.025151	3.884368	13211.64	0.334048	0.451407	6.008067	0.58675	25.24409
0.308208	0.046168	3.847431	12121.98	0.375986	0.529611	2.596888	0.622333	16.2374
0.301509	0.056514	3.333073	1606.083	0.425612	0.580518	1.895789	0.655127	13.78491
0.344419	0.076046	3.263162	782.4306	0.479444	0.63537	1.746969	0.700925	11.81909
0.393479	0.057624	3.530104	697.4779	0.400099	0.552344	2.708086	0.639772	13.68211
0.234081	0.040934	3.80485	15669.46	0.407775	0.559462	2.065346	0.650273	19.05502
0.245909	0.036059	3.732963	59159.81	0.354665	0.504351	2.692642	0.600187	18.51678
1.102338	0.101553	4.119965	26878.5	0.345959	0.491772	4.23614	0.585995	8.93152
0.332039	0.03087	4.193462	35089.08	0.332006	0.460266	4.907712	0.582189	24.44642
0.008418	0.000779	4.001177	16758.37	0.294679	0.405459	7.726112	0.544259	1046.737
1.433007	0.200436	2.900126	387.1261	0.430375	0.593193	4.407939	0.637079	4.537721
0.39608	0.059316	3.859106	3926.398	0.380731	0.537335	2.90956	0.619545	12.86767
1.031207	1	0.080669	8641.565	0.980176	0.989877	0.01056	0.992442	0.992442
0.159506	0.033687	3.798201	3539.11	0.438144	0.590298	1.984208	0.667903	27.68648
0.254665	0.037592	3.884141	16108.26	0.390562	0.538393	2.735597	0.632559	20.05481
1.023136	0.100752	3.564918	3354.013	0.344037	0.48197	4.779331	0.58897	7.284488
0.06634	0.024129	4.064947	28905.06	0.54086	0.69348	0.713461	0.761089	41.15322
0.380275	0.039314	4.236737	10809.56	0.366316	0.481844	5.425762	0.608758	19.34443
0.081554	0.021844	3.453318	4979.107	0.467556	0.617588	1.232924	0.706249	35.17378
0.368575	0.042753	3.848852	15851.36	0.336862	0.473465	3.747324	0.582542	15.62948
0.46074	0.046331	3.83548	44712.56	0.322891	0.454741	4.619976	0.567698	13.81139
0.255689	0.048004	3.434073	5619.392	0.405395	0.543205	2.222286	0.654994	15.61477
0.132155	0.030494	3.547183	16792.12	0.440039	0.58895	1.516812	0.682396	25.26415
0.242701	0.036197	3.866555	4535.238	0.385186	0.519197	3.248091	0.628844	20.47723
0.109216	0.027494	3.242713	677.1356	0.466011	0.616413	1.585107	0.695419	32.3304
0.174157	0.027027	3.857808	8883.733	0.417319	0.538478	3.272362	0.658468	28.08706
0.174509	0.021232	3.891923	19186.36	0.359565	0.502242	3.139664	0.60756	31.82357
1.182386	0.086458	4.094926	15913.67	0.357608	0.483034	7.426399	0.584568	11.55069
0.198078	0.031646	3.828203	4814.71	0.423157	0.569367	2.745228	0.653058	24.19499
0.235267	0.028455	4.204561	26008.14	0.455041	0.595796	3.139673	0.656051	28.83996
0.166082	0.030237	3.520802	1503.79	0.409641	0.553074	2.117207	0.654872	24.54586
0.55289	0.041981	3.878959	8145.448	0.329196	0.446167	6.778087	0.576804	15.82654
0.15909	0.030067	3.275175	981.9334	0.500163	0.638345	2.463868	0.704222	28.97975
0.560176	0.105482	3.208936	1367.035	0.486856	0.642257	1.946889	0.6886	8.600758
0.19884	0.04047	3.750274	4274.152	0.481036	0.627509	1.837906	0.698229	21.92989
0.292096	0.034572	3.746297	9701.413	0.398851	0.54487	3.683062	0.622689	20.54267
0.625292	0.090223	3.615927	2174.312	0.456035	0.607875	2.702076	0.667047	10.4256
0.224824	0.02865	3.695942	13791.59	0.373351	0.515769	3.304563	0.611642	23.48562
0.217089	0.037856	3.773936	3871.095	0.397308	0.545916	2.403719	0.64397	20.4373
0.35317	0.0463	3.627526	3063.077	0.412657	0.554038	3.696943	0.639203	16.45335
0.116925	0.022945	3.56145	3069.658	0.451922	0.589224	2.104666	0.687713	34.88215
0.180008	0.02944	3.528227	5007.15	0.364594	0.521077	2.468131	0.602515	23.67534
0.468172	0.066195	3.312519	1606.741	0.390923	0.549457	3.054122	0.620737	11.89882
0.347935	0.050104	3.456344	2337.381	0.378787	0.51854	3.248092	0.621559	14.23335
0.257411	0.030853	3.827859	21965.31	0.375073	0.517523	3.513453	0.608004	22.1186
0.47338	0.04825	4.056777	45942.56	0.356718	0.496441	4.543507	0.590758	16.01961
0.202519	0.023242	3.974356	18134.63	0.35663	0.485095	3.987138	0.606181	29.37036
1.124708	0.120623	3.40355	3060.702	0.372112	0.511937	4.465995	0.608493	6.759713
0.803763	0.085793	3.59961	4248.217	0.380145	0.532764	3.882408	0.60621	9.13244
0.026534	0.002909	3.970529	21163.16	0.326238	0.451982	4.865175	0.578217	1070.069
0.206513	0.035643	3.693997	5107.861	0.424727	0.571973	2.350793	0.659157	21.69339
0.176271	0.020412	3.444199	11362.52	0.371478	0.503681	3.724819	0.611285	31.5129

0.125059	0.037064	3.598585	7289.094	0.486008	0.642966	0.974318	0.721183	23.44365
0.697791	0.10633	3.516611	3417.698	0.444093	0.598506	2.545924	0.666483	8.874553
0.170744	0.021954	3.851551	25923.88	0.380512	0.497737	3.735415	0.630264	32.47792
0.303924	0.036596	3.565316	2514.285	0.382321	0.530081	3.940287	0.606916	19.61126
0.132661	0.039849	2.754472	1423.037	0.572818	0.719228	0.982437	0.767217	21.35368
0.30176	0.020466	4.273177	25826.25	0.344669	0.47538	6.858842	0.573057	33.96422
0.521778	0.07771	3.486336	2204.709	0.465287	0.61122	2.602829	0.673066	11.54997
0.294446	0.034477	3.649621	6483.298	0.346991	0.478412	4.202616	0.595521	18.64903
0.3929	0.061279	3.3405	1183.185	0.410704	0.553958	2.519652	0.651831	12.8284
0.659406	0.055927	3.574974	4365.903	0.36645	0.50815	5.329866	0.594948	12.48515
0.51031	0.058189	3.83019	9544.948	0.3864	0.528821	3.992209	0.611427	12.84597
0.168162	0.042383	3.585237	4137.026	0.479361	0.622015	1.612968	0.706105	21.14885
0.43107	0.042384	4.011465	11690.49	0.367691	0.515056	4.104138	0.59817	17.68924
0.438004	0.024211	3.947753	12541.97	0.318017	0.428795	10.77115	0.556184	24.53664
0.139758	0.020806	3.845427	15545.43	0.387062	0.524064	2.837352	0.633809	33.74537
0.100333	0.014162	3.948549	6091.327	0.406561	0.554489	3.158049	0.637293	48.31619
0.130905	0.02762	3.862594	13361.43	0.428119	0.582751	1.673214	0.669956	28.17013
0.549109	0.041513	4.222613	44822.66	0.304035	0.434385	6.38326	0.549816	16.04002
1.377509	0.105293	3.374451	2406.318	0.392268	0.551432	5.490248	0.59645	7.284376
0.446607	0.041378	3.663397	3788.236	0.393411	0.533222	5.315399	0.623419	18.27044
0.389228	0.056642	3.492495	2904.421	0.484385	0.625541	2.545295	0.683583	14.9376
0.18836	0.023059	3.700629	6327.771	0.393861	0.516196	3.982597	0.633598	29.99922
0.278277	0.031744	3.939289	4148.763	0.406476	0.5507	4.068108	0.630608	23.87905
0.62111	0.066494	3.326094	1090.316	0.342371	0.466106	4.54328	0.598273	10.58162
0.304796	0.032313	4.021005	32944.17	0.37084	0.514922	4.27118	0.599809	21.13881
0.69356	0.073553	4.043149	5821.145	0.400486	0.542202	4.262226	0.63148	14.34979
0.201116	0.040321	3.707026	4849.666	0.460153	0.609713	1.973853	0.687995	20.88711
0.004398	0.000741	3.576376	7620.789	0.357397	0.500945	3.067483	0.603699	1141.692
0.163578	0.030874	3.396373	888.8108	0.449407	0.60176	1.969704	0.681128	24.93116
0.051574	0.017508	3.585943	2035.353	0.561422	0.702944	0.930519	0.765775	54.08544
0.064485	0.030766	3.636886	613.4531	0.643127	0.772897	0.556157	0.814142	42.32844
0.282452	0.047462	3.775128	2223.72	0.464003	0.60003	3.072146	0.684773	19.31707
0.180113	0.022866	3.947788	10208.74	0.390173	0.540264	3.270603	0.627821	30.79467
0.247437	0.046112	3.618441	13086.93	0.416559	0.557892	2.191055	0.657687	17.12893
0.194627	0.054575	3.235879	1787.549	0.481474	0.637168	1.112772	0.713112	15.56391
0.23235	0.057601	3.412443	571.9936	0.523001	0.667809	2.040828	0.719819	19.30233
0.170261	0.027042	4.311245	3540.466	0.495232	0.624812	2.782194	0.70521	38.14262
0.257636	0.032486	3.922087	6221.036	0.435116	0.56891	3.442677	0.663749	30.89839
0.288279	0.025404	3.607542	6232.221	0.339413	0.483856	5.691479	0.584665	24.88612
0.098384	0.020195	3.910475	10974.55	0.447399	0.584232	2.081315	0.675568	40.88538
0.325676	0.032488	3.677098	3070.704	0.383538	0.499474	5.521918	0.628845	22.43288
0.102629	0.037541	3.674283	1231.004	0.555288	0.691409	0.989204	0.762867	32.85442
0.06426	0.019141	4.175025	9784.726	0.594682	0.720835	1.083865	0.78349	57.48338
0.124274	0.042601	3.548287	493.5614	0.539489	0.689182	1.188206	0.749197	26.43863
1.387671	0.093849	3.952374	2179.105	0.329582	0.406098	11.06455	0.580607	8.649674
0.112839	0.019839	4.028026	2856.804	0.493432	0.630198	2.56097	0.706158	44.13424
0.070092	0.024834	3.607176	2784.067	0.534169	0.67755	0.929957	0.753218	38.44832
0.335938	0.02678	3.809823	8791.543	0.314237	0.444498	6.888105	0.563875	22.1046
0.258441	0.033894	3.960101	2529.891	0.459482	0.588456	4.238089	0.681395	27.55608
0.008829	0.000848	3.916479	12588.77	0.342348	0.460681	5.321452	0.589723	759.0448
0.142335	0.035665	3.529846	3729.043	0.48273	0.627295	1.584457	0.709596	24.85212
0.101566	0.028886	3.822477	3227.118	0.519072	0.663426	1.244579	0.735244	31.47505
0.2464	0.074785	3.192021	400.8567	0.592652	0.72848	0.999969	0.77334	14.31716
0.077713	0.023189	3.303634	6420.121	0.517563	0.659419	1.085637	0.740382	36.50656
0.122926	0.036795	3.49052	5865.699	0.507031	0.652388	1.189375	0.732389	24.96206
0.066901	0.021196	3.398512	1052.114	0.58234	0.715732	0.961781	0.775783	45.20871
0.259369	0.025483	4.110236	25678.98	0.368679	0.511994	4.52455	0.607791	28.63976
0.129392	0.038569	3.540719	2334.454	0.519768	0.656766	1.318996	0.73888	25.16386

0.132108	0.025883	3.758868	10642.44	0.450726	0.595156	2.202683	0.683031	31.35975
0.163725	0.023509	4.181124	4163.937	0.458904	0.584983	3.157007	0.688042	79.30108
0.068158	0.023963	3.512482	2775.257	0.544474	0.686709	0.890782	0.760004	37.09772
0.273743	0.039635	3.727818	2265.322	0.422694	0.560026	3.566698	0.659243	20.55894
0.070296	0.017128	3.507081	10149.31	0.471877	0.611038	1.501926	0.707158	45.275
0.147324	0.038255	3.429445	1761.137	0.49403	0.625888	1.708259	0.72308	22.69714
0.250727	0.027514	3.909592	6549.19	0.3958	0.534596	5.019977	0.629597	26.3714

0.126223	0.03794	3.830113	4447.663	0.517694	0.650033	1.330939	0.738838	28.31713
0.074303	0.0144	3.846352	124044	0.423012	0.564635	1.924951	0.668384	53.13116
0.154684	0.03281	3.680903	5134.069	0.462081	0.585982	2.661107	0.695057	27.1202
0.091379	0.029128	3.477856	1992.502	0.551636	0.696009	1.110603	0.756121	33.19814

0.07097	0.006702	3.690755	48821.29	0.315772	0.454065	4.818968	0.550838	82.77977
0.193499	0.022449	3.947442	12724.27	0.40253	0.536738	4.054801	0.6367	31.86814
0.157681	0.029505	3.86781	19502.44	0.429826	0.576568	2.071933	0.670111	27.04705
0.09311	0.030651	3.27785	510.991	0.586217	0.730769	0.961169	0.773396	32.17833
0.127004	0.025375	3.779704	4650.849	0.476495	0.615893	2.192997	0.701257	33.38965
0.217589	0.031174	3.872592	16306.07	0.402866	0.527639	3.288744	0.645829	24.23019
0.134874	0.037768	3.514188	9589.935	0.495092	0.637466	1.280972	0.724533	23.08642
0.405169	0.053628	3.436038	1388.03	0.431238	0.561186	4.510357	0.666017	15.90104
0.226332	0.036005	3.639312	2124.93	0.447008	0.594579	2.960903	0.675453	23.36215
0.520009	0.036688	4.273696	9715.078	0.336714	0.451392	7.676174	0.574908	19.05105
0.13167	0.046572	3.191646	294.9094	0.592866	0.735775	0.926273	0.776873	24.05059
0.45477	0.052529	3.604674	15090.42	0.345503	0.489831	4.069576	0.592334	12.69595
0.154564	0.023212	4.007904	9533.175	0.427211	0.561672	2.799187	0.6629	33.72463
0.13517	0.067658	3.678847	367.8516	0.759978	0.838235	0.507122	0.873427	29.79137
0.13724	0.029846	3.921078	6399.325	0.467378	0.614759	1.774223	0.697184	29.89456
0.240986	0.022476	4.111858	16618.63	0.373343	0.515772	4.942712	0.606482	31.02617
0.323278	0.05826	3.714954	6656.416	0.443876	0.595645	2.053946	0.674831	15.06537
0.099149	0.025737	3.43862	5946.532	0.472092	0.628583	1.261645	0.704728	30.85001
0.125332	0.040701	3.668456	789.3203	0.611401	0.731922	1.245029	0.780488	29.2278
0.081342	0.024016	3.696208	4127.858	0.483128	0.623275	1.334715	0.718448	37.47013
0.225072	0.045994	4.137945	4454.894	0.461818	0.577401	3.154839	0.68846	23.78158
0.063754	0.018337	3.869897	3410.683	0.527988	0.658822	1.372126	0.745694	51.59601
0.122037	0.026537	4.030495	3561.152	0.485038	0.609247	2.216191	0.71446	34.893
0.358151	0.12071	2.586643	179.8307	0.719084	0.796332	0.963712	0.833363	9.763938
1.605502	0.177589	3.453597	2247.634	0.35689	0.521119	3.931711	0.585427	4.934766
0.996507	0.217512	3.195694	399.0382	0.545243	0.683655	1.729989	0.735481	6.543661
0.005636	0.000725	3.603608	7141.445	0.327353	0.472239	4.220706	0.563566	1068.34
0.237955	0.020527	3.904078	21914.63	0.326932	0.452747	5.400771	0.568476	29.64894
0.380226	0.032856	3.694191	2008.034	0.312258	0.431671	5.939961	0.565648	18.49759
0.512089	0.042648	3.958197	7619.551	0.327007	0.471913	4.956769	0.567581	15.58494
0.820434	0.098317	3.453585	1696.256	0.366178	0.503837	3.300015	0.612829	8.277652
0.1357	0.027963	3.523476	1601.029	0.429208	0.56326	2.28873	0.672176	27.67083
0.863549	0.061523	3.485826	1520.553	0.322959	0.447314	6.826506	0.565769	11.02855
0.117959	0.016405	3.784717	5635.558	0.367176	0.504389	3.361467	0.615926	41.9245
0.100153	0.018272	3.562575	31509.88	0.394421	0.541834	2.112096	0.642098	37.35915
0.9566	0.068243	4.00352	11997.51	0.390561	0.526918	6.059098	0.606041	12.15212
0.534621	0.085934	3.475497	1344.617	0.414502	0.555607	3.485577	0.655683	11.3755
1.818293	0.219131	3.473675	2180.291	0.366668	0.506372	3.744547	0.612997	4.55804
0.256932	0.050929	3.473061	1308.551	0.451998	0.608898	2.001813	0.682183	16.63096
0.031657	0.003532	3.905608	21724.6	0.372489	0.494357	4.1645	0.619965	184.9274
0.100177	0.025335	3.746698	1226.67	0.575417	0.705566	1.693127	0.768108	41.53403
0.085183	0.016981	3.702621	17500.43	0.441268	0.571337	2.06189	0.682781	47.19493
0.183979	0.024819	3.565339	378.4412	0.450756	0.604575	5.241376	0.658684	45.3104
0.089344	0.023615	3.074974	2160.346	0.580637	0.715409	1.427188	0.764488	38.78349
0.277178	0.026188	3.643479	1704.945	0.391438	0.501685	6.810861	0.63597	27.1545

0.631595	0.024238	4.365244	5542.206	0.272722	0.303568	17.6789	0.536171	25.24661
0.163995	0.017686	4.23241	36469.89	0.356204	0.486674	4.536224	0.603287	38.3338
0.475837	0.025314	4.19477	19832.93	0.304747	0.385351	10.08065	0.559801	24.71375
0.290094	0.02183	4.06903	13824.06	0.353558	0.424775	7.635282	0.610949	48.44328
0.808674	0.088158	3.354926	5882.738	0.329555	0.482492	3.629255	0.570695	7.529168
0.302922	0.050832	3.444104	3406.54	0.393855	0.543241	2.439792	0.631886	14.05897
0.283604	0.035581	3.682947	2653.028	0.377302	0.528868	3.338037	0.609841	20.36227
0.005292	0.000751	3.54956	6636.667	0.325602	0.466671	3.798015	0.567486	1083.793
0.835835	0.083868	3.614366	2117.516	0.474947	0.59283	4.037456	0.684169	12.59633
1.074428	0.204026	3.133954	1189.306	0.387504	0.553605	1.936381	0.624704	4.166685
0.248784	0.029244	3.583097	2956.917	0.378987	0.529176	3.713995	0.606921	23.71012
0.795852	0.052555	3.48172	6261.254	0.353371	0.500456	6.455114	0.572802	12.62839
0.618091	0.064984	3.465556	1293.789	0.512175	0.650502	4.506	0.693772	14.83592
0.941964	0.125482	3.22997	1127.204	0.382515	0.513971	3.590643	0.626607	6.626489
0.85072	0.053545	3.701866	6614.802	0.320891	0.434942	7.136337	0.560282	12.81567
0.192413	0.032278	3.520976	1035.227	0.435384	0.577991	2.522149	0.664504	24.67616
1.108507	0.073105	3.798076	1786.966	0.364386	0.464885	9.455867	0.606917	11.20841
0.319174	0.019456	4.130635	13471.87	0.283819	0.393313	9.311347	0.535205	29.19378
0.161006	0.020296	3.778996	12249.19	0.379264	0.502878	4.007273	0.61496	32.743
0.347364	0.045492	3.641697	5362.492	0.362683	0.491335	3.891781	0.612194	16.8745
0.272965	0.032274	3.697843	5620.102	0.382592	0.478441	4.611661	0.635627	22.14078
0.022297	0.004255	4.201381	36842.13	0.444313	0.586832	1.98906	0.679235	166.0593
0.477676	0.055271	3.523052	7463.324	0.345114	0.467785	4.912737	0.591843	11.86161
0.102016	0.050753	3.367103	303.5314	0.723787	0.820352	0.513598	0.847782	39.72011
0.157336	0.031545	3.207458	2008.129	0.470994	0.604037	2.174773	0.705398	25.69156
0.250973	0.022146	3.71278	11919.33	0.35666	0.452332	6.047114	0.606966	29.55798
0.220665	0.022716	3.966012	14636.69	0.343438	0.431338	6.636803	0.597003	36.90575
0.165873	0.045989	4.34673	10033.91	0.512525	0.641272	1.627753	0.72784	40.07567
0.048508	0.024811	5.210958	1120.858	0.832935	0.880033	0.426239	0.923252	348.9788
0.108827	0.030393	3.281656	455.1516	0.541829	0.696331	1.390559	0.746719	31.87861
0.39117	0.058547	4.353246	4305.801	0.365642	0.455607	8.170035	0.612705	30.82946
0.09664	0.019264	3.767929	1438.894	0.449665	0.586641	2.35104	0.684264	48.32116

DX86	DX87	DX88	DX89	DX90	DX91	DX92	DX93	DX94
0.016959	101.5714	0.284514	2.987469	40.85994	541686	21661175	13828.49	0.037086
0.043737	54.76355	0.269771	2.687762	19.5665	1550974	18795077	139501.2	0.117564
0.029112	70.80159	0.280959	3.704586	26.53175	1977706	41948453	97856.27	0.072827
0.078789	9.758621	0.336504	1.453032	11.51724	15117.38	138290	1670.394	0.191159
0.018733	14.73451	0.130394	5.874383	43.92035	65344.44	3561261	1229.016	0.067027
0.034127	63.07547	0.238021	3.660719	23.69057	505967.7	10512511	25765.53	0.090538
0.002378	44.86667	0.175948	11.15008	1853.075	73397.25	1.38E+08	39.99988	0.004462
0.026379	66.8612	0.210919	3.139627	26.25237	232203.6	6662890	8454.97	0.068349
0.067798	7.926829	0.193337	3.620464	18.09756	9970.195	163709	674.1733	0.214305
0.000804	198.5567	0.197372	6.934106	1958.975	489410.9	9.77E+08	245.4711	0.001509
0.011979	514.1487	0.212898	3.053502	50.27288	4006890	2.25E+08	73164.97	0.026667
0.020077	633.5535	0.198109	2.655542	32.06191	20743109	6.03E+08	744558.5	0.046233
0.027423	94.62782	0.177872	3.898705	27.94925	681051.4	20542099	23572.37	0.083914
0.033219	21.25225	0.191462	3.250061	22.65766	87690.29	2220815	3635.462	0.111539
0.039135	8.764706	0.171857	4.23837	25.47059	26533.53	616121.4	1206.435	0.116301
0.017813	356.7529	0.257769	2.210151	33.89957	3010245	1.08E+08	85348.4	0.040752
0.045042	15.43333	0.257222	2.280833	17.88333	89677.85	1497744	5542.108	0.121657
0.016781	79.52453	0.134559	7.030385	51.3621	672982.9	26470670	18278.97	0.035847
0.025166	76.79386	0.168408	3.54801	30.40132	557148.3	21340511	15060.49	0.076146
0.013908	298.2909	0.208595	4.028127	47.44056	2421965	1.31E+08	47134.13	0.032614
0.020885	308.3341	0.182988	3.443016	35.31691	3702635	1.26E+08	121782.1	0.047304
0.01785	175.7821	0.151406	7.06976	46.40999	505394	24793092	10761.17	0.045477
0.027467	16.69412	0.196401	3.743391	26.67059	70071.27	2199907	2317.755	0.08827
0.018985	116.8319	0.16363	5.360774	45.88095	4169272	1.44E+08	126433.4	0.04063
0.014079	111.3884	0.202157	3.150998	39.6098	1280838	64280545	25841.35	0.045037
0.040619	20.23944	0.285062	2.66336	19.57746	99358.45	1768302	5768.853	0.106817
0.0009	127.3585	0.400498	40.50046	1813.758	962616.8	1.78E+09	520.667	0.005762
0.026887	48.41017	0.164102	4.066877	34.41017	635472.7	19772388	22109.49	0.059368
0.018714	44.50427	0.190189	3.521294	34.2906	167987.8	6823890	4244.363	0.050663
0.028035	57.86726	0.1707	3.788133	29.86136	202070	7366511	5945.228	0.075508
0.065628	25.14493	0.364419	2.098719	12.63768	56823.43	678087.5	5160.279	0.176176
0.0303	185.6854	0.217941	2.624705	26.80986	1614551	39492328	69213.86	0.065552
0.021513	22.1453	0.189276	3.429323	41.61538	70768.68	2465014	2066.279	0.044516
0.015864	122.9913	0.177477	3.789551	43.69986	1750142	80419707	40405.36	0.040938
0.033973	1025.906	0.246909	8.983988	34.09723	14085735	2.87E+08	743900.8	0.065804
0.033661	23.4	0.275294	2.055917	23	402268.6	9303223	17977.93	0.076489
0.025852	235.1887	0.172047	4.760375	38.55669	5082159	1.32E+08	222361.5	0.048201
0.06048	12.63636	0.229752	2.976529	19.56364	48206.29	654856.5	3836.761	0.149283
0.016767	35.85938	0.280151	3.790771	40.73438	200062.9	8314113	4927.371	0.044796
0.02513	11.63736	0.127883	5.955561	35.18681	35286.85	1456053	881.2888	0.09325
0.015311	94.17864	0.193385	4.753024	48.57906	294776.2	16920500	5238.877	0.042302
0.0121	675.6904	0.193719	4.4963	57.5129	4561446	2.84E+08	76896.44	0.024665
0.016704	38.67227	0.162489	4.536685	40.94118	156824.3	7937369	3300.805	0.042496
0.015259	165.1413	0.16432	7.532513	55.67264	4191503	1.67E+08	108604.7	0.031934
0.012479	199.9729	0.25803	3.413005	50.76516	2135242	1.05E+08	43857.85	0.028349
0.081241	25.41379	0.292113	1.623728	11.18391	192281.3	1828347	20930.27	0.201759
0.045172	36.73684	0.322253	1.634734	13.64035	347522.8	5653889	21916.09	0.12442
0.030577	83.21008	0.174811	4.501571	30.56303	1191671	36283389	42738.05	0.076023
0.039001	100.5711	0.234431	3.195962	25.98601	980033.7	20779078	49876.55	0.085683
0.043152	59.0203	0.149798	7.15986	31.65482	649131.8	13585045	34016.4	0.105243
0.017849	256.4637	0.258532	2.525477	30.24698	2161522	83127948	57067.87	0.052928
0.064461	26.04082	0.265723	7.288213	28	88111.93	1302846	6253.83	0.13306
0.000876	34.53488	0.200784	13.69832	1848.227	162752.5	3.17E+08	83.72735	0.006353
0.02029	54.80392	0.179098	5.829478	41.08824	384586.1	13644915	11058.01	0.048569
0.01358	264.2013	0.140682	9.890376	62.74281	1707160	93816306	31782.01	0.0313
0.01749	89.70853	0.144458	7.559243	70.3285	593040.2	47416774	7489.181	0.046026
0.031871	61.97338	0.23564	2.452312	21.5057	447128.2	11007935	18622.74	0.093608

0.055774	45.71053	0.300727	1.896079	18.48026	78251.86	1226739	5105.55	0.10893
0.019585	17.26549	0.152792	4.891378	39.62832	63873.64	2468525	1730.437	0.053826
0.022152	63.78638	0.197481	3.536505	29.75851	280830.4	10643688	7559.318	0.058984
0.011057	684.4034	0.183093	3.578913	56.52809	21284724	1.15E+09	408368.1	0.024167
0.046041	20.98333	0.174861	3.799931	21.79167	76536.15	1578467	4015.5	0.130934
0.024116	141.364	0.25471	2.637202	30.10991	790660.5	27912041	22628.89	0.059572
0.015452	447.2928	0.324125	2.494068	46.20725	2147495	1.01E+08	46075.32	0.031878
0.029039	215.6262	0.237736	3.356749	28.75524	1097231	31258668	41922.67	0.061485
0.038301	30.54128	0.280195	2.8538	22.73394	137964.8	2820712	7095.304	0.09203
0.055683	13.18182	0.239669	2.609587	14.41818	37228.51	624994.1	2321.396	0.16531
0.037498	11.37931	0.196195	3.138228	25.2931	57274.41	1069950	3210.987	0.089899
0.026916	277.963	0.228776	3.312273	30.02798	981510.8	28257661	36880.47	0.058039
0.022027	1419.173	0.313907	2.648547	32.96549	8252259	2.56E+08	275811.6	0.046671
0.058273	243.5699	0.192241	2.740902	16.23362	4300679	62353078	401824.5	0.199514
0.018208	510.1084	0.153972	26.04322	79.43948	4235682	1.53E+08	123295.9	0.03685
0.000511	611.0773	0.310665	11.43304	1956.795	4195466	8.1E+09	2173.661	0.001279
0.142736	9.83871	0.317378	1.340271	8	28362	246301.6	3432.143	0.319668
0.038884	48.53712	0.211952	2.289773	16.95633	391066.2	8499893	19637.1	0.111375
0.992442	7804	1	0	1	1.503588	1.503588	1.503588	1
0.024364	57.01521	0.216788	3.954257	41.94297	188446.7	8218846	4465.732	0.050027
0.024228	213.6127	0.185912	4.49064	33.74848	1372792	40967379	48148.11	0.058736
0.062574	77.82946	0.301665	2.001923	12.05426	591835	6991085	54331.21	0.193489
0.018582	252.2367	0.107564	8.265046	60.77655	367031.8	18648697	8201.43	0.036072
0.024885	142.8029	0.187652	3.260141	28.19448	1329876	45997123	39981.85	0.066102
0.015729	117.09	0.239448	4.063658	48.78119	200269.3	10020679	4094.097	0.031488
0.025604	256.6148	0.230976	4.025057	28.41674	3087931	82249845	119381.3	0.061804
0.027013	818.7548	0.266956	2.081093	23.52038	11112624	2.69E+08	473056.8	0.064377
0.032412	171.6298	0.287487	2.256834	24.34841	434250	10394673	18583.44	0.069382
0.021124	355.256	0.225131	4.078015	38.52852	867787.1	31061504	25022.93	0.04523
0.02456	94.60899	0.212604	3.195485	28.15056	363314.6	12674012	10641.19	0.071885
0.020422	16.05556	0.222994	3.9375	43	26997.96	1270623	582.6745	0.063428
0.018961	144.2713	0.186397	3.599617	36.44703	610820.1	28896792	13112.23	0.048026
0.012743	241.156	0.195902	3.903868	49.9805	2560653	1.2E+08	57305.51	0.031273
0.052769	115.7202	0.140779	15.9508	50.54866	2951222	46949894	193771.9	0.103498
0.021849	57.15287	0.182015	4.255599	32.37261	319577.4	12899191	8218.814	0.068394
0.018687	88.0208	0.140833	4.872566	42.8576	2040303	79851839	57464.77	0.041936
0.020106	33.92994	0.216114	4.056797	41.75796	104606.6	3693307	3020.976	0.043749
0.024613	176.9617	0.260621	2.458371	29.83947	1715404	43676605	68262.19	0.050009
0.022476	17.35616	0.237756	3.53875	40.69863	39514.11	1761650	907.6142	0.052151
0.07538	15.4	0.28	2.341818	12.58182	98626.44	1223864	8713.905	0.219813
0.028866	39.82645	0.164572	3.818318	30.41322	165799.1	5259473	5650.103	0.077168
0.022461	106.2009	0.237056	3.061897	33.04241	1251325	41905684	38416.56	0.049285
0.061179	25.0531	0.221709	3.079803	17.29204	131865.6	1824548	10446.09	0.170756
0.018038	259.6936	0.268834	2.771922	35.35404	1791257	70219386	46623	0.04315
0.025335	78.38537	0.191184	5.55417	36.42439	231310.2	7472448	7444.733	0.056474
0.03172	44.19786	0.236352	2.564843	21.09626	293357.5	7938046	11127.7	0.084676
0.016209	50.72781	0.150082	5.851835	61.48225	124593.9	6006408	2616.978	0.034241
0.019311	88.2	0.245	3.182029	31.125	741567.9	31392160	17951.2	0.062533
0.043806	25.10989	0.275933	2.603309	18.86813	235221.3	4272601	13445.47	0.140783
0.032786	65.92683	0.321594	2.15881	25.05854	283038.7	6815817	11981.47	0.074851
0.019076	229.6471	0.192981	3.604322	39.13193	3072138	1.12E+08	86214.61	0.038813
0.028972	405.5536	0.159228	21.42859	59.04829	6941305	1.67E+08	304180.8	0.059044
0.014283	290.2574	0.174959	8.574109	60.66787	2179549	1.04E+08	46689.99	0.027953
0.075861	65.39048	0.311383	1.732993	11.16667	465203.8	4331848	50898.84	0.205295
0.052319	52.02439	0.253778	2.781582	17	661198	8594229	55232.25	0.144917
0.002544	375.5322	0.222077	4.173415	1866.216	3560903	6.53E+09	1973.598	0.00113
0.024072	107.5185	0.265478	3.177662	32.60494	325680.3	10139445	10836.7	0.05131
0.012631	263.3601	0.34114	2.062839	46.75518	1981933	99867739	39580.22	0.027428

0.026919	118.6576	0.179784	4.450824	38.3197	209021.2	6461221	7170.972	0.048889
0.070914	49.4492	0.264434	2.592239	16.12834	232942.9	2664360	22682.71	0.167311
0.014371	505.4098	0.176101	10.56378	60.61324	2444699	1.23E+08	49175.87	0.033422
0.023591	53.58442	0.347951	2.545286	38.7013	349127.3	11916153	10438.63	0.045854
0.030881	34.05426	0.263987	2.078	27.93798	27500.73	720537.5	1065.026	0.056231
0.011673	139.7714	0.123364	62.78654	157.6125	5450856	2.81E+08	108070.8	0.022605
0.052546	19.93478	0.216682	2.788634	21.59783	157356.6	2544594	10852.47	0.110368
0.021421	157.5904	0.261344	2.564678	29.66335	997197.3	33155566	30779.3	0.055474
0.040335	34.98165	0.320933	1.999663	18.14679	95872.79	1673051	5659.604	0.099874
0.033687	74.33624	0.324612	2.290727	19.28384	805005.5	15314950	44242.76	0.095734
0.036922	96.18571	0.229014	2.8436	21.55952	1381428	29932951	67361.04	0.094833
0.031961	77.72897	0.242146	3.13153	25.08411	162979.2	5360562	5118.028	0.080647
0.025837	102.1678	0.174945	6.021296	34.61815	1686331	51414868	58905.85	0.077606
0.014174	198.08	0.243641	3.064129	44.19434	3996792	1.9E+08	84749.94	0.034714
0.013505	273.4303	0.20497	4.920009	50.37631	1481867	78608334	28396.92	0.030536
0.009391	61.71233	0.169075	4.005825	65.27397	485849.3	40978428	5863.322	0.022396
0.018843	184.0727	0.163185	4.185299	38.8156	641325	25235199	17053.22	0.044661
0.022983	519.882	0.178838	9.73676	45.98486	11153983	3.16E+08	417283.7	0.045059
0.063314	26.4186	0.307193	2.080043	13.13953	446888.5	4799447	44500.26	0.172258
0.026988	48.60526	0.213181	3.178728	25.74123	437005.4	12246385	16060.17	0.086932
0.039148	25.13913	0.218601	2.813308	25.02609	178636.3	3733527	9149.077	0.081325
0.015247	105.352	0.203776	3.012702	40.25338	670588.1	32738399	13832.03	0.037399
0.020769	28.80374	0.134597	4.952681	38.04206	360977.4	13184847	10287.63	0.069897
0.041375	67.96386	0.409421	1.306866	14.98795	149635.6	2425606	9280.386	0.104212
0.020147	289.2313	0.196756	3.68903	35.6	4666046	1.71E+08	134218.3	0.05464
0.04685	64.38158	0.211782	3.275104	26.84868	540969.3	12736605	26549.65	0.119383
0.028719	67.27108	0.202624	2.44102	23.76205	208895.8	7034687	6657.208	0.070029
0.000504	186.1048	0.295404	5.44618	1843.678	1123020	2.13E+09	591.7069	0.002131
0.021469	18.925	0.236563	2.783594	39.5375	42239.25	1488014	1229.45	0.047511
0.013982	32.64929	0.154736	6.677298	69.09479	28819.79	2113067	400.2733	0.035252
0.026397	8.549296	0.120413	7.216425	46.67606	3230.775	179809.1	61.53565	0.072633
0.035056	19.03226	0.153486	3.737253	25.01613	117516.1	3596227	4023.153	0.100577
0.01439	107.0826	0.184307	3.23538	48.66437	945408.7	46212681	20093.3	0.029921
0.031862	225.2468	0.218899	2.880744	25.49174	1006662	26442107	39505.2	0.074196
0.039855	78.03863	0.33493	2.731824	24.13734	50131.06	1058054	2494.99	0.077316
0.04496	8.069767	0.187669	3.081666	26.25581	15053.44	503235.7	474.5289	0.086224
0.020134	21.20571	0.121176	8.139624	60.64	91347.95	5127811	1802.695	0.039835
0.023652	57.11168	0.144953	9.056069	61.08629	385642	16809687	9233.081	0.056566
0.015346	99.51236	0.223623	3.109789	39.32584	1204930	52670124	28205.26	0.04357
0.01478	133.2883	0.170011	4.709143	52.0625	611011.8	41142455	9202.144	0.033739
0.021285	71.49342	0.235176	4.575561	41.97039	320506.6	11479479	8992.159	0.043926
0.031098	24.39474	0.160492	5.826004	37.98684	15535.07	710185.1	354.5465	0.083536
0.015444	89.97974	0.107246	9.682717	77.53397	61144.46	4447675	937.0985	0.024923
0.034788	6.88	0.1376	4.6004	31.02	7828.62	294820.3	216.6792	0.099397
0.062244	59.80383	0.286143	1.952657	14.38756	548071.9	8612489	35492.69	0.161385
0.014768	23.78431	0.11659	8.399726	61.81863	74149.81	4832444	1173.995	0.03831
0.019822	54.43109	0.159622	5.975404	44.73607	43574.84	2340886	830.7596	0.054639
0.015647	119.8076	0.190473	4.4175	44.13355	2194639	96381976	51442.63	0.035178
0.024601	22.23684	0.146295	5.363573	38.88158	133073.9	5091461	3551.999	0.064033
0.000526	218.6605	0.212086	10.16746	1265.43	2252890	2.92E+09	1741.776	0.002154
0.026897	66.01734	0.190802	4.132255	34.89306	129277.6	4756453	3591.422	0.061233
0.02229	32.19283	0.144362	5.623439	37.95516	79522.94	3540249	1876.454	0.058741
0.060372	9.473684	0.249307	3.252078	18.42105	4718.132	88737.84	286.9068	0.143176
0.017562	133.9248	0.189964	4.675145	53.75461	155605.9	7509755	3287.241	0.034379
0.028288	118.6412	0.197078	4.229404	33.78904	149336.3	5119015	4482.285	0.067357
0.016678	14.37778	0.159753	4.879136	64.85556	15938.82	859719.9	303.2509	0.035201
0.015537	169.8383	0.113	28.58332	98.57219	1996055	87587324	47399.07	0.028656
0.030406	45	0.174419	3.821059	30.5155	46477.47	1712547	1288.175	0.076588

0.018814	140.3572	0.173495	3.87324	40.87392	492607.5	24591085	10122.95	0.045574
0.016205	33.81115	0.052339	154.4567	432.613	102762	4684011	2298.888	0.019098
0.019031	54.925	0.171641	4.332461	42.85	42896.3	2255242	833.9898	0.039797
0.02773	25.74684	0.162955	4.009974	29.96835	169468.6	5429970	5470.642	0.079327
0.012497	198.888	0.195371	4.882466	64.29961	395931	26144543	6069.84	0.024337
0.029397	41.97222	0.194316	3.207283	27.88426	47434.08	1635114	1402.162	0.070445
0.018478	77.97044	0.192045	4.476959	40.83251	667678.8	30687820	14805.24	0.049087
0.03013	54.32692	0.130594	6.475869	36.83173	98045.66	3778906	2629.741	0.086812
0.009701	2290.59	0.162384	25.79845	129.7427	6050600	4.4E+08	84354.55	0.014863
0.025213	94.06694	0.190805	5.098585	35.98783	236572.2	11047169	5144.003	0.071068
0.023236	34.6682	0.159761	4.567988	42.87097	28163.18	1337200	605.9645	0.059206
0.003776	398.385	0.190524	3.777636	127.2415	22028429	3.44E+09	141923.7	0.009463
0.014808	111.6908	0.168463	4.444299	49.04977	1264659	67425995	24502.63	0.034212
0.020295	248.7463	0.160171	3.995683	33.9736	891375.1	34970220	24217.87	0.055217
0.024492	7	0.159091	4.03719	41.13636	6019.5	248158.1	151.4774	0.060946
0.018766	47.83436	0.146731	5.309985	43.32515	191920.3	9767568	3857.154	0.04762
0.021362	262.0124	0.202483	3.487962	33.82303	1421280	54277994	38000.46	0.051852
0.028552	200.2166	0.207478	3.582915	29.4715	269281.1	8797480	8538.116	0.067534
0.038594	46.49701	0.278425	2.370612	25.08982	70898	1769964	2857.503	0.091845
0.025844	28.70732	0.175045	3.785247	34.43902	112196.4	4022309	3205.268	0.057703
0.021284	98.21094	0.191818	3.11412	31.87891	2131376	73826172	63927.32	0.054545
0.038064	5.454545	0.247934	3.628099	29.54545	4616.636	155363.6	146.6937	0.099586
0.032	427.0338	0.344105	1.920677	23.91459	2434605	53541712	115735	0.063567
0.015654	98.85426	0.153262	4.971206	52.93488	536865.1	26717172	11248.67	0.03577
0.060308	7.714286	0.137755	5.586416	31.125	511.1071	20462.13	18.13338	0.090146
0.021711	74.79447	0.147815	6.642706	45.34783	214325.2	9269778	5147.917	0.051043
0.013758	141.4213	0.167362	4.606589	51.07219	2164093	1.01E+08	47788.19	0.031202
0.039017	73.52857	0.175068	2.908588	24.79762	336121.2	6967270	18180.64	0.081712
0.018553	169.5212	0.248201	4.276071	46.74524	192627.5	8345573	4554.149	0.035336
0.033679	6.787234	0.144409	4.987777	27.70213	9885.149	452339.7	240.501	0.103569
0.018613	113.299	0.188204	4.969054	42.92857	92088.16	5229394	1654.06	0.048277
0.036463	49.58309	0.144557	5.04873	22.89504	175546.4	6950686	4607.764	0.164364
0.014425	54.18162	0.118559	9.007522	60.70897	46874.72	3291515	679.1966	0.037182
0.020501	67.19369	0.151337	4.277859	32.14865	58702.7	3208566	1134.53	0.059805
0.10163	2.333333	0.259259	2.17284	12.55556	2815.667	33871.89	283.0738	0.227068
0.105802	27.31884	0.197963	4.359588	18.44928	375206.3	2707137	60351.67	0.22718
0.162906	6.481481	0.240055	2.222222	11.22222	10110.04	64883.48	1935.316	0.353858
0.000475	112.2824	0.259913	11.48866	1869.657	1943922	3.7E+09	1021.858	0.003427
0.011864	414.9037	0.266476	2.336325	47.45986	5008707	2.57E+08	98994.38	0.027192
0.019377	70.77385	0.250084	2.193085	32.22968	344600.2	11875084	10163.69	0.047454
0.023943	85.51948	0.185107	2.975713	26.44156	1429105	34943501	62633.15	0.068036
0.06053	62.49727	0.341515	2.401206	12.90164	197352.9	2032090	20161.8	0.18722
0.020343	46.38865	0.202571	3.739555	37.72489	70142.5	3275757	1524.204	0.051132
0.035665	42.00763	0.320669	1.807704	17.50382	391407.5	6521533	23839.19	0.095651
0.01087	129.3385	0.182424	4.560952	48.88011	489340.8	34327900	7065.736	0.036509
0.011946	862.4156	0.305064	2.865471	59.04386	2801815	1.67E+08	47809.5	0.023309
0.040493	80.37531	0.202457	3.412914	21.87909	1688472	30538294	104228.6	0.107219
0.062508	36.77333	0.245156	2.625956	16.26667	84518.95	1393030	5206.07	0.170556
0.138931	46.75758	0.283379	1.813186	8.636364	314941.7	1874153	61887.28	0.404621
0.03573	28.18621	0.194388	3.719382	29.41379	47779.68	1099331	2157.544	0.072474
0.002216	347.1835	0.212345	5.347636	285.4544	2792404	8.13E+08	9615.285	0.004918
0.020495	13.20183	0.121118	7.165727	49.95413	13187.31	692550.9	267.605	0.067608
0.01202	260.5975	0.149855	29.10478	103.7349	965899.4	63636607	14803.76	0.025759
0.018653	2.789474	0.146814	8.759003	63.05263	30101.58	2011569	457.792	0.08713
0.01869	32.35789	0.170305	4.172964	47.17895	27809.12	1431933	546.9884	0.049814
0.0179	48.57949	0.249126	2.996345	43.30256	150673.4	7302051	3123.512	0.038282

0.01403	337.08	0.2809	3.112156	46.84833	2061672	99914413	42709.5	0.035336
0.011094	460.4075	0.161603	3.914416	59.192	4017083	2.56E+08	64114.7	0.026212
0.014228	632.4296	0.281831	1.940416	40.44786	4702690	1.81E+08	124240.3	0.03148
0.013754	371.7107	0.151657	113.05	234.8862	1661915	81704186	33887.26	0.024952
0.049897	76.47407	0.283237	2.432661	17.07037	2192054	26162755	198894.2	0.126115
0.033375	81.37956	0.297006	2.679165	24.5365	344025	7828168	15708.22	0.074398
0.022378	32.54037	0.202114	5.062845	36.24224	365839.4	11349706	12194.06	0.069577
0.000491	96.69863	0.264928	8.671841	1915.37	2169214	4.08E+09	1152.989	0.003263
0.054785	26.88333	0.224028	2.998264	20.70833	109538.9	1234032	10670.57	0.129973
0.135342	36.09091	0.410124	2.126033	7.5	145747.9	938571.2	26565.22	0.562778
0.018798	40.5625	0.253516	2.723086	33.45625	473572.6	18437299	12373.15	0.054634
0.0294	81.7619	0.353948	2.267199	21.34632	1859836	35315099	102362.4	0.078684
0.046007	10.96721	0.17979	3.557646	21.03279	50425.08	873029.8	3030.372	0.137423
0.084323	53	0.414063	1.131836	8.1875	121892.5	1129769	13326.98	0.239449
0.028996	110.0441	0.269716	4.199244	24.07598	2329915	40581394	137451.7	0.087708
0.022074	12.24138	0.211058	3.423603	40.46552	92383.76	3314443	2639.527	0.055029
0.044847	34.20468	0.200027	3.837967	35.42105	250603.3	3897569	16476.73	0.05709
0.010689	355.3199	0.241879	2.630166	57.25323	3618054	2.05E+08	65087.05	0.024493
0.013346	216.8796	0.251018	4.254287	53.51389	1693014	1.02E+08	28531.21	0.030204
0.030528	164.5606	0.28921	2.629168	21.57996	673733.5	18061731	25605.52	0.101961
0.021526	164.522	0.241235	2.997095	35.91642	618843.7	21917784	17616.15	0.047835
0.00293	299.7302	0.12981	5.836316	234.6986	1541210	3.86E+08	6240.173	0.005288
0.035721	164.413	0.344681	2.478893	26.01258	2039868	48113143	88841.56	0.087536
0.044474	4.310345	0.148633	7.06302	38.65517	1420.655	78036.97	31.5557	0.092941
0.02301	60.55365	0.259887	3.17486	37.39485	82404.23	2967565	2299.476	0.048941
0.013964	385.2981	0.315559	2.056461	44.51433	2018525	99441757	41122.96	0.029948
0.015303	440.1666	0.27666	3.125527	50.95349	2836567	1.84E+08	43968.92	0.035133
0.035304	94.81972	0.133549	7.285239	51.31127	212013.5	16679321	4345.889	0.077526
0.022174	12.89691	0.026592	227.3234	667.6309	182.5361	10262.61	4.177631	0.014247
0.023876	11.67925	0.220363	4.250623	37.58491	7033.66	292767.9	173.8446	0.066889
0.044937	95.51551	0.227961	5.589761	50.47017	618148.7	39625091	9974.306	0.083992
0.014188	30.7766	0.163705	5.632526	61.56383	51924.48	3477764	782.5972	0.036608

DX95	DX96	DX97	DX98	DX99	DX100	DX101	DX102	DX103
56.96078	0.159554	0.388526	14.91405	0.015058	5.490278	0.015498	537522.7	33.09415
23.51232	0.115824	0.299774	6.193025	0.034754	5.466748	0.007	1530565	160.0248
66.07937	0.26222	0.517021	15.00776	0.040284	4.734671	0.007614	1960458	54.3233
4.448276	0.153389	0.368741	4.211034	0.102165	3.961429	0.032151	14149.95	4.468353
24.38053	0.215757	0.469464	21.62101	0.038751	5.310273	0.022996	63453.35	6.689913
57.76226	0.217971	0.455595	10.59521	0.046112	5.121633	0.013519	500496.2	55.01552
59.91765	0.234971	0.484067	887.3756	0.000284	5.215181	0.03161	72396.46	1.487819
94.82334	0.299127	0.563837	14.2778	0.042431	4.864013	0.019727	229634	31.04182
8.658537	0.211184	0.436656	6.457778	0.101169	4.405856	0.033497	9078.954	4.110558
245.2644	0.243802	0.503577	990.4293	0.000263	5.536949	0.026499	487986.8	3.790301
605.8853	0.250884	0.50977	25.32795	0.01434	5.291186	0.013677	4001544	142.9224
580.4153	0.181493	0.419183	13.25502	0.019561	5.824584	0.00715	20723545	577.1725
127.8383	0.240298	0.499454	14.03576	0.047416	5.483265	0.01549	676883.9	54.79645
18.96396	0.170847	0.389395	7.150226	0.06833	5.120132	0.019412	85036.65	13.82439
8.254902	0.161861	0.353697	9.010595	0.047945	4.860973	0.024733	24898.83	5.472209
302.9422	0.218889	0.472676	15.79926	0.021006	5.210854	0.013793	3004989	144.393
10.6	0.176667	0.39998	7.846281	0.050765	4.649792	0.016811	86139.58	13.6189
146.2217	0.247414	0.501452	26.04177	0.019891	5.740899	0.014437	668185.2	21.41827
94.02193	0.206188	0.45871	13.94374	0.037026	5.6508	0.015588	553032.9	58.90224
417.3399	0.291846	0.554407	26.7998	0.017616	5.079586	0.013054	2416097	106.4714
405.1412	0.24044	0.494293	16.99305	0.025644	5.420057	0.010404	3693396	225.7851
279.9681	0.241144	0.49665	25.14322	0.022499	5.755049	0.023553	503591.3	20.2948
17.51765	0.20609	0.452781	12.05625	0.037402	4.839838	0.020296	67643.68	9.05495
108.6891	0.152226	0.370021	18.04206	0.016558	6.264148	0.007144	4149675	65.05803
101.2069	0.183679	0.42523	16.67329	0.021316	5.725473	0.012804	1274738	38.26802
14.74648	0.207697	0.44626	8.758374	0.04867	4.414895	0.018149	96322.6	9.845702
53.40252	0.167932	0.407283	724.7979	0.002331	4.953589	0.014626	957942.2	1.540263
58.00339	0.196622	0.439987	14.46573	0.02983	5.598014	0.010791	626884.7	32.1548
55.01709	0.235116	0.481041	15.11023	0.026654	5.092132	0.021376	165799.2	14.93251
87.15339	0.25709	0.517867	15.16383	0.041477	5.272314	0.020279	199638.2	30.5684
16.04348	0.232514	0.468871	6.384597	0.082567	4.021376	0.022556	54857.99	18.34991
160.3897	0.188251	0.428592	11.24021	0.029174	5.475841	0.013052	1608681	143.0325
28.04274	0.239681	0.49319	18.93075	0.029067	4.991677	0.025248	69199.98	6.20575
130.0303	0.187634	0.425716	17.18446	0.019942	5.784551	0.010031	1740203	82.08699
1045.304	0.251577	0.508976	20.62002	0.034276	5.194183	0.010101	14075934	139.2443
22.6	0.265882	0.518905	13.25422	0.030941	4.178312	0.010341	392916.6	22.95946
307.398	0.22487	0.47511	19.85873	0.024062	5.735193	0.007751	5065515	111.1505
15.83636	0.287934	0.55245	12.05314	0.087745	4.444818	0.021024	45943.99	11.67056
37.14063	0.290161	0.556074	25.24242	0.027328	4.606847	0.016853	196542.2	10.4696
13.37363	0.146963	0.358667	11.26912	0.047379	5.521086	0.026678	33881.83	3.725864
101.6735	0.208775	0.457536	22.37345	0.019962	5.590212	0.022946	292876.9	21.1283
799.8968	0.229328	0.485202	27.75896	0.012672	5.731113	0.014077	4556400	77.2556
46.83193	0.196773	0.448934	17.68194	0.018882	5.67855	0.017147	153423.2	16.25244
188.2537	0.187317	0.428937	25.48864	0.012637	6.129771	0.008968	4179068	47.58228
187.6529	0.242133	0.494259	25.65673	0.014017	5.017388	0.0124	2128738	32.59404
16.97701	0.195138	0.438163	4.545025	0.100197	4.607346	0.014421	187472.5	38.7011
20.77193	0.18221	0.426535	5.220987	0.06221	4.759666	0.012099	340692	35.77925
88.02101	0.184918	0.41118	12.83437	0.031179	5.717235	0.009826	1181313	63.53559
103.6434	0.241593	0.493632	13.01008	0.04674	5.038476	0.011717	972750.3	63.80506
89.6599	0.227563	0.482614	16.84116	0.0454	5.776764	0.013377	643543.3	24.23269
211.1835	0.212887	0.464505	14.54569	0.027226	5.277839	0.014053	2156459	115.3484
22.97959	0.234486	0.463089	18.54207	0.052764	4.69451	0.023032	86226.77	7.595094
41.94186	0.243848	0.501609	932.2288	0.000917	5.024356	0.020124	160283.2	1.726201
60.45098	0.197552	0.441496	19.99514	0.023934	5.662857	0.016911	381089.3	13.80635
441.4739	0.235077	0.490212	32.95693	0.016165	6.100917	0.0179	1704038	37.95135
111.9919	0.180341	0.422802	29.35553	0.020588	6.247883	0.019116	590303.6	16.87664
59.18251	0.225029	0.483041	9.430337	0.058535	5.012836	0.015325	442870.5	44.40059

30.09211	0.197974	0.445304	7.964932	0.053168	4.857138	0.028127	76987.87	18.06672
38.38053	0.339651	0.603709	22.67341	0.032291	4.786234	0.021972	61802.18	5.932539
65.82972	0.203807	0.454895	12.72508	0.031222	5.46582	0.019318	278150.8	18.63749
786.1792	0.210321	0.459821	25.25849	0.011739	5.867793	0.007088	21264818	321.9792
21.05	0.175417	0.40637	8.877756	0.062066	5.378581	0.020986	74265.63	17.19117
109.2162	0.196786	0.428317	13.55082	0.025935	5.141261	0.018199	787641.2	56.17089
323.4174	0.23436	0.488283	22.20653	0.016057	4.89884	0.016507	2143825	105.5742
280.6362	0.309411	0.571695	16.22309	0.035281	4.779347	0.015469	1093052	68.96383
26.08257	0.23929	0.495733	11.4811	0.045542	4.627651	0.017875	134834.9	20.03722
11.94545	0.21719	0.465521	6.213623	0.09821	4.574021	0.022974	35333.89	9.005021
16.13793	0.27824	0.523091	12.50562	0.06024	4.418603	0.019529	54652.27	7.261463
311.2305	0.256157	0.510176	15.3539	0.030414	5.059499	0.018313	978529.1	125.3694
1182.061	0.26146	0.521735	17.19983	0.025406	4.821207	0.014135	8247254	537.4115
319.753	0.25237	0.503171	8.04342	0.109816	5.163147	0.00858	4287094	415.1656
993.5306	0.299889	0.563077	50.17443	0.02047	5.825144	0.015024	4231252	61.54229
432.0402	0.219644	0.471943	939.9008	0.000373	5.14961	0.014752	4190871	5.28959
5.967742	0.192508	0.425328	4.198844	0.115132	4.082598	0.023169	26499.1	13.78766
62.25764	0.271867	0.532956	8.933905	0.064234	4.867458	0.012754	384918.7	54.00041
6212.863	0.796113	0.912336	0.912336	0.912336	0.609121	0.876263	0.201228	0
54.5057	0.207246	0.450546	17.3107	0.022449	5.203947	0.020465	186059.1	18.93349
332.45	0.289339	0.553693	18.00542	0.035152	5.327699	0.015827	1368800	53.60115
68.4031	0.265128	0.526202	5.797267	0.119985	4.540105	0.013648	586466.3	107.3923
748.3945	0.319145	0.583241	35.2467	0.022492	5.749405	0.030634	365966.2	36.59229
164.7267	0.216461	0.467153	12.58321	0.034783	5.508461	0.013405	1324311	70.40066
163.0245	0.333384	0.595969	28.48886	0.020228	4.563342	0.028591	199046	17.47828
285.2844	0.256782	0.514662	14.88912	0.032903	5.150192	0.011669	3080587	74.92466
716.6433	0.233663	0.488105	11.4644	0.03233	5.02307	0.010594	11103714	656.7258
183.7672	0.307818	0.573806	13.97484	0.042372	4.531334	0.023784	432482.2	57.5866
490.9316	0.31111	0.572995	21.96338	0.028586	4.855952	0.024648	866141.1	57.38675
97.86742	0.219927	0.474426	12.73699	0.040601	5.302972	0.020715	360984.2	36.19346
15.02778	0.208719	0.437172	15.97433	0.023218	4.57731	0.032071	26025.73	3.266138
181.478	0.234468	0.493283	18.00555	0.023642	5.47246	0.020688	608483.6	44.41972
396.0918	0.321764	0.586517	27.76302	0.019314	5.025616	0.012044	2553759	100.7279
129.1655	0.157136	0.378398	23.10565	0.033501	6.64188	0.010125	2941468	71.30354
71.07643	0.226358	0.483192	15.2032	0.042661	5.423126	0.017013	316122.7	29.72435
70.3024	0.112484	0.311825	12.93997	0.01331	6.655732	0.007433	2022202	111.0879
49.31847	0.31413	0.578141	22.12277	0.03046	4.679297	0.024405	102927.7	6.478473
155.8601	0.229544	0.484824	14.22726	0.0256	5.145558	0.013082	1709561	55.28663
11.24658	0.154063	0.328767	13.88473	0.012957	4.942762	0.026411	38080.5	4.72774
9.4	0.170909	0.394891	5.104023	0.085926	4.649275	0.013974	93505.08	23.27607
49.36364	0.203982	0.44448	13.32366	0.037119	5.497507	0.0184	162845.5	25.27176
71.93304	0.160565	0.386521	12.68425	0.018657	5.570111	0.011151	1243283	72.90548
16.80531	0.14872	0.343761	5.255782	0.081172	5.285331	0.016129	128021.6	23.39549
199.2422	0.206255	0.451889	15.55948	0.022129	5.242377	0.014358	1786406	111.7844
129.5756	0.316038	0.582304	22.74111	0.032277	5.09023	0.023833	229549.7	14.09732
30.89305	0.165203	0.39031	8.957484	0.032848	5.254693	0.015356	289116.5	27.31303
93.39053	0.276303	0.533251	34.31816	0.015134	5.416893	0.030588	123525.1	7.643448
110.9056	0.308071	0.568526	18.14332	0.03857	4.680848	0.014403	736747.3	52.25324
18.47253	0.202995	0.444184	9.468978	0.053088	4.600611	0.013219	229498.7	26.03489
44.86341	0.218846	0.471198	10.83384	0.04498	4.806819	0.018238	280032.5	29.80059
211.9395	0.1781	0.403777	15.68753	0.016669	5.827537	0.011356	3064384	73.26594
449.8245	0.17661	0.410132	32.77081	0.023679	6.407611	0.01069	6932555	72.00608
442.2827	0.266596	0.525671	32.85337	0.014829	5.588466	0.016524	2175886	35.36798
33.95238	0.161678	0.375593	4.413875	0.089466	5.029814	0.01419	460237.6	70.69103
30.89268	0.150696	0.371884	5.414555	0.070074	5.312703	0.010792	652612.4	75.94203
568.4004	0.336133	0.600761	1114.881	0.000326	4.754955	0.012156	3554136	14.38119
90.12099	0.222521	0.470032	14.51189	0.027283	5.073913	0.020426	323283.5	28.03561
128.3109	0.166206	0.399798	18.75664	0.012546	5.294675	0.013501	1976447	62.31712

223.6273	0.338829	0.60197	22.80721	0.031331	4.927174	0.028447	207785.5	28.0546
35.69519	0.190883	0.42885	6.661997	0.079296	5.071594	0.015849	228961.8	56.86792
755.5045	0.263242	0.521794	34.02328	0.018309	5.742205	0.021672	2442570	39.75247
23.97403	0.155675	0.382339	15.10173	0.014313	4.945038	0.013809	343883.3	23.36861
27.75969	0.215191	0.452166	13.16523	0.030875	4.685428	0.039741	26867.56	8.159485
192.1139	0.169562	0.408653	108.2708	0.007874	6.854256	0.008058	5435456	18.90086
18.86957	0.205104	0.447744	10.23129	0.051447	4.83963	0.013281	151687.5	22.16238
176.6915	0.293021	0.555522	16.56897	0.034266	4.711266	0.016089	993334.1	82.49058
25.42202	0.23323	0.478177	8.969963	0.049274	4.528143	0.022002	93807.13	15.57498
36.40611	0.158979	0.378511	7.528556	0.037283	5.132829	0.01089	796573.6	72.42202
73.74762	0.17559	0.402418	8.469764	0.044383	5.46607	0.009967	1371362	110.3635
72.84424	0.226929	0.482749	13.26252	0.035619	5.123892	0.024292	161284.6	30.75836
105.8938	0.181325	0.419742	14.34006	0.038165	5.965062	0.010395	1677077	53.6922
122.8868	0.151152	0.364324	15.9423	0.013354	5.770664	0.009847	3986478	76.00581
384.7646	0.288429	0.550714	27.87798	0.017458	5.30407	0.018039	1478794	43.42665
73.34521	0.200946	0.450029	29.1654	0.011738	5.566004	0.014667	481201	29.20344
363.8794	0.322588	0.584551	22.45785	0.027533	5.145078	0.021618	639185.2	63.29708
751.9075	0.258654	0.515753	25.6745	0.022728	5.671458	0.009102	11141911	125.6848
12.53488	0.145754	0.372448	5.249588	0.070526	4.924787	0.009035	434637.1	51.41053
39.2807	0.172284	0.398365	9.415828	0.037806	5.43536	0.014169	432024.7	37.97319
16.63478	0.14465	0.36414	9.656749	0.028736	5.326873	0.013569	173205.2	30.24232
85.10058	0.164605	0.393025	14.91113	0.017124	5.713144	0.017754	667415.4	32.7288
39.28037	0.183553	0.431369	16.32288	0.042082	5.794251	0.012911	354978.4	23.31573
33.08434	0.199303	0.431183	6.426239	0.048888	4.351193	0.025019	148038	20.53917
247.1837	0.168152	0.404353	13.73124	0.02652	6.019765	0.009413	4654760	192.1922
51.17105	0.168326	0.390869	10.82893	0.044007	5.562388	0.012507	534576.2	45.78354
84.33735	0.254028	0.511074	11.33965	0.040064	5.013022	0.0205	206516.3	35.82592
175.727	0.278932	0.543654	1002.16	0.00036	4.649732	0.01541	1118809	9.724391
18.2	0.2275	0.452549	18.78819	0.028238	4.532063	0.025856	40743.49	5.114308
45.65403	0.21637	0.461206	32.19462	0.022167	5.577788	0.042652	28270.1	3.947125
12.40845	0.174767	0.413457	20.55782	0.03788	5.352153	0.060426	2956.895	1.581493
26.45161	0.213319	0.466207	13.65616	0.047881	5.225158	0.017165	114122.1	14.73497
129.1308	0.222256	0.472256	22.00378	0.01583	5.403997	0.012864	939365.9	39.8516
276.4597	0.268668	0.526933	14.07253	0.039198	5.040323	0.018935	1003872	111.0859
62.34764	0.267586	0.525883	12.97162	0.041085	4.409151	0.040806	49530.5	18.24833
7.093023	0.164954	0.387668	11.81711	0.022766	4.580578	0.029015	13865.6	3.447802
33.13714	0.189355	0.440617	28.69634	0.018867	5.870469	0.016967	87874.37	7.541775
81.71066	0.207387	0.451905	32.876	0.021549	5.956286	0.017101	382222.4	12.63825
96.55056	0.216968	0.460714	17.62557	0.020618	5.227834	0.012581	1198612	66.51337
142.9133	0.182287	0.419134	23.51592	0.012227	5.938515	0.019715	608438.9	39.73208
65.81579	0.216499	0.465022	23.41143	0.018653	5.30373	0.020386	318100.4	12.64195
40.80263	0.268438	0.514235	21.29458	0.04436	5.005268	0.049335	15124.21	5.074259
212.2157	0.252939	0.5115	41.41594	0.013293	6.084	0.039069	60489.31	7.537075
9.12	0.1824	0.400028	13.47379	0.046505	4.973661	0.039841	7198.61	2.20775
43.03828	0.205925	0.449469	6.097835	0.078511	4.858607	0.013835	542847.2	40.70661
44.27451	0.217032	0.471042	30.16097	0.02065	5.760331	0.024493	72482.85	4.27562
96.3607	0.282583	0.54639	24.55004	0.034423	5.259754	0.045243	43086.31	8.340447
174.0684	0.276738	0.538905	25.88383	0.018891	5.169678	0.010553	2185659	37.98333
31.98684	0.21044	0.466659	18.62999	0.030461	5.478258	0.018093	130019.1	12.0351
216.0068	0.209512	0.448501	564.0558	0.000973	5.559656	0.013628	2247506	5.381357
80.14451	0.231632	0.49017	19.27108	0.025932	5.355581	0.029387	128119.6	13.45748
48.36323	0.216875	0.467045	15.95336	0.035476	5.487278	0.024941	77915.39	11.06051
8.789474	0.231302	0.447242	7.573127	0.08166	4.178738	0.044341	4209.51	3.317188
211.0965	0.299428	0.558186	30.83163	0.018041	5.045792	0.038305	154924.4	13.41534
159.1262	0.264329	0.524242	18.33225	0.0364	5.288968	0.034886	148514.6	22.68007
17.62222	0.195802	0.429585	28.35813	0.02021	5.191458	0.037594	15231.26	2.92018
422.332	0.280993	0.546601	66.59681	0.014467	6.275078	0.012024	1989138	28.81475
66.60465	0.258158	0.518957	15.51932	0.044215	5.073327	0.039329	45830.97	12.35546

180.335	0.222911	0.477915	20.51022	0.022844	5.60612	0.021585	490461.3	52.60431
208.4118	0.322619	0.586421	308.4395	0.011013	6.925401	0.044917	102266.3	2.930407
95.45	0.298281	0.563577	25.40944	0.023813	5.051448	0.044114	42382.43	10.66889
28.74684	0.181942	0.416027	14.11131	0.030332	5.457104	0.017753	166295.6	15.07831
287.0452	0.28197	0.542314	36.73556	0.012975	5.147528	0.02953	394784.3	18.30038
53.46296	0.247514	0.509386	14.66457	0.042361	5.153296	0.039273	46785.72	10.24774
61.8867	0.15243	0.384388	15.39729	0.022871	6.009752	0.014468	662901.8	25.70181
99.28846	0.238674	0.494976	18.0911	0.046341	5.778602	0.032525	97100.39	12.33765
4093.781	0.290216	0.552095	81.77873	0.008007	6.002497	0.027627	6049290	83.73947
110.9838	0.225119	0.481486	17.50583	0.036515	5.494068	0.027346	235235	29.68066
46.42857	0.213957	0.460482	19.58623	0.037169	5.441545	0.044061	27648.07	7.596601
430.6375	0.205948	0.45206	58.7519	0.004551	5.76254	0.006477	22004590	221.4116
128.3152	0.193537	0.434629	21.93536	0.016465	5.924368	0.012276	1258024	38.55629
432.7012	0.278623	0.540711	18.57299	0.031796	5.456205	0.020539	889004.6	84.5889
8.045455	0.182851	0.410847	16.25252	0.036667	4.708987	0.039568	5380.789	1.683523
74.17791	0.22754	0.486479	21.75637	0.025727	5.650208	0.021969	189848.4	17.3957
326.7589	0.252518	0.51179	16.89063	0.028821	5.409227	0.017705	1418090	81.83483
277.5886	0.287657	0.552283	16.55843	0.03916	5.126902	0.032528	268336	51.0274
29.99401	0.179605	0.420669	11.03886	0.046724	5.058448	0.031605	69896.86	11.87064
44.90244	0.273795	0.528519	18.68458	0.02786	5.005773	0.022267	110179.6	11.26246
104.543	0.204185	0.455769	14.38683	0.026318	5.532311	0.008801	2118465	73.72048
2.909091	0.132231	0.326344	8.12656	0.06378	3.845351	0.034921	3796.595	1.720898
288.1281	0.232174	0.481631	11.57655	0.030884	4.680643	0.014571	2429895	263.6022
135.7256	0.210427	0.462083	24.55977	0.018307	5.906128	0.017257	533507	27.44969
10.5	0.1875	0.419751	13.51812	0.024561	4.84353	0.102941	416.7398	1.868783
134.2688	0.265353	0.526488	23.63076	0.025594	5.563048	0.023942	212580.7	13.32057
167	0.197633	0.444122	22.475	0.013799	5.896769	0.010811	2155538	49.06544
99.59524	0.237132	0.485522	11.5641	0.042369	5.303762	0.017654	332912.8	45.32446
200.5608	0.293647	0.55678	26.50264	0.019857	4.965244	0.035096	191815.6	22.56175
7.553191	0.160706	0.392035	12.14878	0.049901	5.027889	0.029338	8723.354	3.530841
197.412	0.327927	0.594324	25.24205	0.030092	5.10456	0.044652	91586.6	15.06449
68.58017	0.199942	0.451727	9.569198	0.092018	5.773511	0.022206	173518.5	21.5775
134.4748	0.294256	0.556285	34.44307	0.022558	5.669945	0.048545	46450.38	5.358079
101.9054	0.229517	0.48332	14.47163	0.03431	5.562129	0.038676	58034.17	12.94238
1.666667	0.185185	0.367356	1.849376	0.168038	2.947703	0.032028	1840.84	3.149071
28.37681	0.205629	0.44261	10.81619	0.088251	5.264941	0.012485	368791.2	30.78068
3.148148	0.116598	0.278111	3.7098	0.03808	4.20841	0.027864	8822.025	12.90851
116.7731	0.270308	0.528416	981.4826	0.001007	4.873396	0.010026	1933973	7.420266
312.8767	0.200948	0.447673	20.97808	0.012317	5.420025	0.011252	5000808	102.49
68.32862	0.241444	0.497345	15.59608	0.02779	4.889438	0.019817	342053.7	21.56414
108.3723	0.234572	0.485084	11.93734	0.039545	5.232907	0.010088	1419278	84.39996
55.42623	0.302876	0.553808	7.165832	0.10439	4.044561	0.020843	195051	39.82326
62.86026	0.274499	0.532226	19.91028	0.028331	4.895928	0.03582	69363.14	8.621574
22.45038	0.171377	0.398267	7.087278	0.035472	4.739675	0.013436	385868	34.95813
200.3089	0.282523	0.544554	25.56539	0.022653	5.271518	0.024275	487643.8	28.16438
790.7595	0.279717	0.543484	32.00729	0.013262	4.782633	0.019566	2799203	174.287
49.73552	0.125278	0.317945	7.000353	0.03583	6.059995	0.007858	1672276	87.60905
29.01333	0.193422	0.431905	7.008786	0.072032	5.016702	0.027337	83180.86	17.71584
45.15758	0.273682	0.524974	4.470229	0.207439	4.315965	0.014809	310381.8	118.7964
43.34483	0.29893	0.560097	17.58209	0.037133	4.819412	0.032431	46828.91	8.681963
398.6771	0.243839	0.503454	142.1945	0.002199	5.51801	0.014508	2787653	19.82486
25.45872	0.233566	0.487692	22.84994	0.041609	5.410304	0.039138	12534.49	1.992652
482.678	0.277561	0.539313	69.73719	0.013811	5.925452	0.025619	964375.8	12.67718
1.631579	0.085873	0.182766	15.24091	0.008175	4.142664	0.015523	25951.51	1.114031
45.08421	0.237285	0.49772	23.89384	0.025829	5.373826	0.039874	27180.17	5.481403
39.78974	0.20405	0.419585	18.23617	0.013762	4.921333	0.024197	148965.4	9.952426

351.165	0.292638	0.55567	25.8777	0.019657	4.782715	0.018288	2058682	34.70064
692.9614	0.24323	0.500495	29.85562	0.012657	5.732559	0.014319	4012206	145.5359
563.4171	0.251077	0.512258	21.21025	0.015953	5.029238	0.013854	4697480	159.7762
686.6589	0.280155	0.543464	171.649	0.012937	6.356195	0.027559	1660598	11.3213
100.4667	0.372099	0.631602	10.80614	0.0789	4.094963	0.007642	2174931	159.4536
59.29927	0.216421	0.463949	10.76622	0.039978	4.817206	0.01817	340996	48.41358
33.64596	0.208981	0.457706	17.73308	0.036185	5.279326	0.013132	360040.7	15.74342
104.7205	0.286906	0.549827	1052.865	0.000399	4.757168	0.00876	2156182	6.776791
15.91667	0.132639	0.288336	6.055397	0.045819	5.313962	0.017943	106432.7	33.83122
28.52273	0.324122	0.58619	3.930358	0.357211	3.551323	0.016626	142130.1	64.03635
20.8125	0.130078	0.337263	13.03567	0.013781	5.512381	0.011915	466529.2	21.37983
35.57143	0.153989	0.381766	7.831648	0.03332	4.990215	0.00744	1841771	110.6837
6.245902	0.102392	0.281143	5.321279	0.050614	5.387796	0.018418	47477.13	10.59477
22.625	0.176758	0.414718	2.899882	0.107385	4.451814	0.023734	120117.3	31.64722
101.5735	0.248955	0.504029	12.81835	0.048117	4.99903	0.009329	2318426	55.29742
9.241379	0.159334	0.387117	16.3006	0.013829	4.824944	0.015191	88050.49	6.165511
37.53801	0.219521	0.476552	21.15247	0.022892	5.16241	0.017673	247401.4	14.55512
440.7876	0.30006	0.563205	32.09821	0.014912	4.91515	0.012753	3611905	85.4661
152.5116	0.176518	0.403654	21.56103	0.012857	5.646515	0.014439	1688217	60.00412
141.9139	0.249409	0.508012	11.44056	0.048227	4.965296	0.019639	671140.7	50.74357
197.9355	0.290228	0.552733	18.93219	0.029168	4.839764	0.022844	616927.4	38.12296
524.2638	0.227052	0.482143	114.0901	0.002817	6.11725	0.017069	1537777	21.55767
128.3585	0.269095	0.527961	14.18481	0.043695	4.545932	0.010818	2031322	89.11122
4.37931	0.151011	0.364457	12.30818	0.029121	4.487123	0.060797	1150.109	0.705559
49.72103	0.213395	0.440055	17.02893	0.024315	5.03198	0.034234	81550.99	7.065745
225.3702	0.184578	0.414388	18.57238	0.012647	5.249285	0.017313	2015189	52.58774
399.5141	0.251109	0.507398	26.19303	0.016787	4.992166	0.016766	2833010	82.98031
132.4676	0.186574	0.426712	23.91133	0.040394	6.250347	0.024573	210357.4	28.59424
211.5938	0.436276	0.686201	511.4204	0.006144	6.866317	0.324632	173.0471	0.471229
11.86792	0.223923	0.45738	17.14515	0.0297	4.447273	0.046986	6580.693	1.917133
59.72554	0.142543	0.35348	19.46218	0.025698	5.800073	0.017482	614876.6	21.46891
41.69149	0.221763	0.475466	29.04567	0.013581	5.454504	0.036286	51165.01	3.674302

DX104	DX105	DX106	DX107	PX1	PX2	PX3	PX4	PX5
0.000317	21.63398	0.006024	0.018783	0.884482	0.85208	28.81693	33.81952	39.45305
0.000248	6.518083	0.015024	0.003495	0.829014	0.768183	30.82133	40.12239	49.37898
0.000184	40.09831	0.001759	0.05738	0.707607	0.659473	39.70995	60.21468	70.14081
0.011525	2.811831	0.009032	0.093788	0.915664	0.614744	9.926085	16.14669	18.56419
0.001475	26.06712	0.015306	0.088376	0.881098	0.774987	22.06616	28.47295	33.67329
0.000371	12.914	0.011217	0.010631	0.847029	0.796541	34.62638	43.47091	46.38444
0.000974	66.9635	0.040137	0.92006	0.837677	0.736297	28.2192	38.32585	45.85708
0.00051	17.71712	0.012747	0.017903	0.877301	0.728593	31.42545	43.13167	47.18187
0.007671	8.885322	0.020763	0.128569	0.871913	0.687989	11.99672	17.43737	20.28529
0.000182	129.8378	0.004748	0.25634	0.802616	0.721632	44.7459	62.00656	72.46505
4.63E-05	34.76508	0.004007	0.005121	0.769122	0.727363	75.51287	103.8173	166.9516
1.95E-05	21.5785	0.004682	0.001458	0.761385	0.686829	89.11208	129.7441	153.327
0.000237	19.82709	0.007954	0.012229	0.87467	0.862653	43.53415	50.46543	63.18997
0.001419	13.58058	0.015073	0.037188	0.867895	0.689522	18.9999	27.55517	33.00095
0.003687	12.73148	0.014011	0.103489	0.924112	0.653821	13.10149	20.03834	22.7774
8.66E-05	14.0821	0.005076	0.004038	0.802141	0.758665	65.86934	86.82274	95.5027
0.002327	5.925113	0.010978	0.034006	0.881085	0.760412	19.42484	25.54514	30.87938
0.000224	105.6292	0.002901	0.108948	0.908091	0.726235	43.87002	60.40749	68.59465
0.000271	14.95931	0.01688	0.009232	0.831568	0.812008	39.32573	48.43022	58.59675
7.38E-05	30.67172	0.007431	0.006497	0.908831	0.865531	70.07632	80.96338	96.31251
5.94E-05	20.08543	0.010944	0.002678	0.789339	0.779794	74.60493	95.67256	101.4742
0.000176	197.9621	0.002854	0.166032	0.869192	0.816665	49.13489	60.16527	72.53395
0.001853	13.12831	0.01204	0.059907	0.888286	0.369456	9.898357	26.79169	27.5799
8.79E-05	77.56964	0.002286	0.035512	0.819821	0.724076	51.89909	71.67627	84.64315
0.000193	27.43195	0.003926	0.017896	0.837741	0.807866	45.65707	56.51563	70.25915
0.001717	15.17101	0.004688	0.088944	0.829915	0.676481	20.54359	30.36831	36.37543
0.000489	127.9273	0.002113	3.697284	0.868369	0.728778	31.99345	43.90016	54.06198
0.000332	36.88091	0.008886	0.031309	0.800924	0.638316	31.0698	48.67462	51.68248
0.000731	22.00567	0.007737	0.042803	0.746582	0.694259	27.55094	39.68396	44.34282
0.000491	19.79074	0.019149	0.01645	0.880083	0.809138	35.83408	44.28674	51.49574
0.002093	6.505917	0.011419	0.031907	0.778088	0.555937	15.66627	28.17992	27.18807
0.000135	11.70622	0.010855	0.003682	0.955903	0.792097	49.80801	62.88124	78.81098
0.001802	19.38044	0.005492	0.09372	0.318838	0.285941	24.46536	85.5608	32.77815
0.000138	20.66872	0.009955	0.007256	0.713198	0.670311	46.74522	69.73659	71.81052
2.24E-05	553.4871	0.000326	0.355187	0.810215	0.780219	101.7284	130.3844	139.7976
0.000747	11.0164	0.004049	0.029949	0.746246	0.561015	20.77446	37.03014	35.14325
6.31E-05	81.56327	0.003546	0.027006	0.641264	0.570633	61.33639	107.4883	104.0042
0.002492	9.786716	0.011209	0.055616	0.908207	0.900581	23.83097	26.46177	32.47321
0.000762	29.91156	0.002797	0.090452	0.735851	0.615166	22.36507	36.35616	35.07967
0.002737	23.84557	0.011093	0.163372	0.829857	0.729462	15.18857	20.8216	24.94853
0.000349	32.96705	0.007368	0.03158	0.633387	0.579719	33.96595	58.59033	50.89013
4.35E-05	127.1949	0.002564	0.028615	0.931389	0.872648	81.51216	93.40789	119.8272
0.000643	32.14815	0.017565	0.034289	0.966748	0.887353	29.43015	33.16622	40.98645
7.85E-05	144.3775	0.001098	0.114893	0.887217	0.713351	49.03077	68.73298	81.13514
0.000131	69.17235	0.001031	0.06795	0.731649	0.599306	43.85835	73.18187	81.15038
0.001372	3.601709	0.012903	0.011444	0.872665	0.834814	22.21189	26.607	32.42341
0.000888	5.770703	0.009623	0.012669	0.795593	0.645094	21.38329	33.14754	37.95232
0.000175	35.58821	0.009883	0.015176	0.852818	0.711297	37.59994	52.86113	59.71919
0.000242	21.6373	0.007369	0.020006	0.873854	0.81063	37.41902	46.1604	51.13022
0.000331	67.70751	0.002541	0.14419	0.634301	0.486937	37.63265	77.28439	64.35153
0.000102	16.32503	0.005206	0.005408	0.886248	0.756292	50.73788	67.08767	82.18983
0.001478	58.80524	0.002601	0.572644	0.932778	0.818677	19.96185	24.38305	27.3586
0.000868	58.28077	0.013951	0.957467	0.835127	0.774128	28.45703	36.76012	42.53452
0.000457	67.65348	0.002503	0.19971	0.582503	0.496646	26.71554	53.79189	56.75788
7.94E-05	188.8056	0.00151	0.105563	0.731371	0.667138	56.92053	85.32042	94.03315
0.000259	64.54642	0.009037	0.055836	0.928033	0.735708	33.62279	45.70129	54.36636
0.000495	8.685811	0.010794	0.011101	0.780294	0.711257	32.41797	45.57843	53.61984

0.001479	8.795042	0.009639	0.028557	0.901428	0.775256	24.2887	31.32989	35.98956
0.001752	27.29391	0.009962	0.097051	0.940536	0.812647	22.35294	27.50633	35.5631
0.000476	32.54499	0.005811	0.039298	0.871565	0.68868	27.93481	40.56282	47.11404
1.80E-05	43.14032	0.003498	0.002905	0.876816	0.761788	93.12975	122.2515	136.2508
0.001321	14.45412	0.018417	0.03272	0.950331	0.749429	20.75431	27.69348	30.94491
0.000257	11.84842	0.007495	0.010016	0.978597	0.729907	42.27207	57.9143	68.23976
0.000103	14.93707	0.005437	0.00603	0.875517	0.750787	58.2302	77.55891	85.69564
0.000141	39.0581	0.005353	0.019089	0.793471	0.638778	57.31409	89.72458	109.4529
0.000974	13.45863	0.008369	0.03276	0.887623	0.749307	22.4913	30.01615	32.69492
0.00301	10.44442	0.014408	0.055128	0.818526	0.688149	18.88065	27.43686	28.89286
0.002856	11.01576	0.007937	0.082196	0.916733	0.820615	20.05149	24.43471	29.88011
0.000128	16.97721	0.011975	0.004499	0.857118	0.638382	48.16824	75.45367	81.94659
2.24E-05	20.11147	0.005344	0.001296	0.949598	0.750265	87.28535	116.3393	130.9497
6.82E-05	12.08068	0.016092	0.001916	0.909894	0.850805	65.39505	76.86255	88.60535
4.30E-05	351.0342	0.001203	0.197931	0.938078	0.794721	77.59615	97.63949	120.9387
6.97E-05	308.8451	0.000546	0.530379	0.717768	0.575822	55.54391	96.46021	81.40104
0.005742	1.970111	0.019845	0.029553	0.950561	0.700424	12.98894	18.5444	21.69939
0.0005	9.278662	0.017457	0.009367	0.899586	0.72428	29.28542	40.43386	46.29535
1000000	0	0	0	0.899208	0.875367	66.8569	76.37582	86.46151
0.00058	23.61298	0.013275	0.030067	0.784592	0.621819	27.77904	44.67384	50.6478
0.000114	95.40056	0.002537	0.058526	0.824587	0.65589	49.92762	76.12195	84.96337
0.00036	6.047625	0.009818	0.005581	0.819033	0.760585	36.03527	47.37836	55.138
0.000131	140.5269	0.009493	0.037505	0.849044	0.787412	33.41131	42.43181	44.41487
0.000178	18.69638	0.009947	0.009102	0.81222	0.775903	46.18369	59.52246	70.85878
0.000437	37.22117	0.005834	0.03672	0.886281	0.82569	21.93397	26.56443	30.98832
8.38E-05	76.73512	0.001482	0.045738	0.89568	0.769948	58.22476	75.62163	91.66549
2.72E-05	11.39298	0.006398	0.000849	0.628542	0.586556	80.9679	138.0394	142.9455
0.000309	12.57492	0.007232	0.008717	0.87155	0.75092	44.62732	59.43016	68.51773
0.000118	65.44886	0.003368	0.030746	0.663385	0.602696	27.63742	45.85634	56.24683
0.000371	17.24252	0.009865	0.014355	0.92597	0.819238	36.68457	44.77887	53.04867
0.003359	18.40697	0.007943	0.197114	0.765283	0.594684	15.75649	26.49558	32.12818
0.00023	21.55225	0.009364	0.012081	0.78774	0.738991	44.18423	59.78997	64.75532
8.01E-05	29.74597	0.005422	0.00708	0.768638	0.706774	64.16891	90.79124	105.1276
0.000104	114.6677	0.002645	0.089856	0.947295	0.775104	39.85372	51.41728	62.59103
0.000451	17.59763	0.012226	0.018664	0.941248	0.684682	26.32842	38.45351	47.28493
0.000109	31.83399	0.020117	0.005347	0.781959	0.742816	39.35915	52.98637	56.85722
0.001225	37.72457	0.003388	0.176825	0.795761	0.715507	22.02371	30.78055	36.38601
0.000164	30.93525	0.002094	0.02616	0.892333	0.715548	43.33367	60.56012	72.57018
0.002453	18.33185	0.009127	0.136842	0.811329	0.664291	14.06927	21.17937	26.39611
0.00176	6.92961	0.017652	0.023563	0.943963	0.857214	17.79814	20.76277	25.86822
0.000639	18.93597	0.017504	0.020979	0.804326	0.684258	23.92618	34.96659	43.0197
0.000184	17.82979	0.009121	0.007425	0.93692	0.894739	35.5931	39.78044	46.16696
0.00112	12.71725	0.013053	0.035324	0.890323	0.672665	18.19577	27.05025	29.29638
0.000109	16.81812	0.007011	0.00552	0.849798	0.813424	45.0154	55.34061	62.83508
0.00048	76.21595	0.003088	0.224528	0.946449	0.805154	40.59186	50.41504	60.0366
0.000637	12.87173	0.01041	0.018552	0.907563	0.753995	22.45513	29.78154	38.03286
0.000703	59.32999	0.003142	0.149796	0.984156	0.750627	27.23002	36.27636	44.45965
0.000222	19.61967	0.008772	0.013496	0.833505	0.78896	36.91276	46.78659	48.75018
0.000911	9.185545	0.008494	0.022327	0.714387	0.669181	18.47195	27.60381	30.58524
0.000696	8.688051	0.007816	0.016518	0.948605	0.718276	27.07861	37.69948	47.60975
7.96E-05	64.80091	0.002066	0.027157	0.779428	0.571185	39.85775	69.7808	88.76681
3.65E-05	594.5853	0.000556	0.366817	0.828486	0.702748	61.02557	86.83851	125.491
8.53E-05	193.1139	0.001015	0.15536	0.825017	0.705115	58.5241	82.99937	99.13623
0.000556	5.642374	0.008534	0.007879	0.976751	0.802745	23.55912	29.34822	42.15972
0.000393	8.772082	0.010196	0.008599	0.838285	0.704434	26.5259	37.65562	42.40582
6.20E-05	79.06715	0.011077	0.085768	0.925311	0.788402	78.88178	100.0528	112.7508
0.000388	24.92468	0.00653	0.027512	0.67821	0.625736	30.2744	48.3821	51.69683
0.00014	19.98245	0.003082	0.010469	0.926974	0.808878	39.79051	49.19221	63.35462

0.000331	47.09996	0.007496	0.034376	0.915512	0.766754	22.91622	29.88733	31.61938
0.000615	10.72354	0.016577	0.012228	0.833471	0.732665	23.0853	31.50866	38.51491
5.83E-05	336.5206	0.000725	0.234539	0.664427	0.638277	66.09143	103.5466	114.7212
0.000634	12.83147	0.008582	0.024711	0.840241	0.762087	21.5093	28.22421	32.46947
0.002049	14.19878	0.008011	0.066115	0.95026	0.736978	34.93387	47.40149	58.70531
6.82E-05	998.5797	0.000375	1.604935	0.794925	0.748021	50.70899	67.79085	82.90591
0.00109	13.99764	0.012658	0.03067	0.934693	0.786518	20.14295	25.6103	30.64134
0.00018	12.51705	0.006046	0.007588	0.889119	0.829762	46.60603	56.16797	64.75532
0.001576	8.854845	0.008588	0.031861	0.875986	0.828229	22.77443	27.49775	31.76583
0.000333	8.779006	0.008235	0.007897	0.945352	0.844494	27.78153	32.89727	37.74404
0.000184	12.60366	0.011109	0.005146	0.843996	0.743649	34.65376	46.59961	56.50886
0.000558	16.58754	0.020513	0.015022	0.732682	0.677582	31.11955	45.92736	51.86574
0.000145	52.63943	0.004382	0.033617	0.866216	0.779183	39.19821	50.30682	57.2912
9.69E-05	23.99111	0.002347	0.010387	0.869799	0.778776	43.24706	55.5321	61.50513
9.74E-05	86.7065	0.002194	0.043698	0.676324	0.652737	57.28628	87.76314	98.36551
0.000317	22.79125	0.016323	0.020532	0.862305	0.786681	29.59963	37.62594	44.21969
0.000163	27.51111	0.00977	0.009516	0.903591	0.732583	48.89894	66.74864	69.97931
2.82E-05	241.1364	0.000732	0.092847	0.679135	0.554856	76.47137	137.822	125.6699
0.000782	5.852068	0.010598	0.011566	0.82091	0.611243	15.56261	25.46058	30.46948
0.000445	12.88756	0.008853	0.014881	0.89826	0.724448	25.62671	35.37412	42.05152
0.000873	11.04413	0.019022	0.016363	0.933454	0.821972	21.16202	25.74544	31.21098
0.000286	21.24862	0.005699	0.017263	0.833988	0.730063	32.34701	44.30717	52.15513
0.000507	21.88229	0.009765	0.023738	0.840349	0.673262	23.11435	34.33187	40.79792
0.001388	5.271499	0.005667	0.020234	0.890989	0.722945	24.51511	33.91006	42.14424
5.48E-05	25.41078	0.007196	0.003414	0.826123	0.698112	46.21742	66.20348	79.09622
0.000392	15.86552	0.017247	0.013816	0.857978	0.782212	31.49479	40.26373	48.08991
0.000511	15.15738	0.021191	0.013923	0.921334	0.86872	33.72815	38.8251	47.76765
0.000152	53.44566	0.006464	0.219741	0.777368	0.718661	49.56909	68.97427	78.75133
0.002558	17.30651	0.007744	0.106987	0.762779	0.66968	21.77285	32.51232	38.80234
0.00143	62.98638	0.012295	0.160692	0.768078	0.737894	30.03692	40.70628	47.43625
0.006643	40.52616	0.037045	0.319294	0.666665	0.602239	13.51304	22.43802	22.26733
0.001068	18.23742	0.016025	0.037585	0.771257	0.687625	26.15536	38.03725	42.95391
0.000205	29.48379	0.006311	0.015959	0.882007	0.768272	50.40098	65.60302	81.18704
0.000136	17.76754	0.008851	0.005047	0.793658	0.739724	60.53495	81.83447	91.71971
0.001081	15.38045	0.0113	0.030696	0.758738	0.718014	23.84064	33.20361	38.69231
0.005088	14.31428	0.023728	0.145885	0.585939	0.489819	15.36368	31.36606	35.23736
0.000918	70.89881	0.017975	0.094669	0.919936	0.813312	28.76885	35.37246	43.22499
0.000337	146.2842	0.0038	0.211271	0.905293	0.80887	42.44342	52.47248	61.76189
0.000195	13.52114	0.005489	0.012127	0.853991	0.607159	42.33435	69.72533	74.49476
0.000197	31.95174	0.015026	0.015238	0.932214	0.854322	38.22775	44.7463	60.76133
0.000566	39.49061	0.002334	0.107148	0.842481	0.735749	37.67296	51.20351	58.89532
0.002301	30.69111	0.030978	0.099099	0.666213	0.564558	18.7488	33.20968	35.71792
0.000497	182.2571	0.012293	0.153452	0.885832	0.834268	40.29774	48.30313	57.54788
0.006619	20.16339	0.023395	0.216577	0.751273	0.666258	15.59504	23.40691	27.30444
0.000632	6.636322	0.006816	0.013308	0.648305	0.558115	29.9474	53.65808	62.72836
0.001042	94.82458	0.006476	0.297809	0.896452	0.686731	31.75693	46.24363	61.81451
0.000961	42.6321	0.014073	0.066202	0.945067	0.89113	29.56359	33.1754	38.40412
0.00013	64.12279	0.00131	0.06297	0.748692	0.594717	52.0787	87.56892	93.94668
0.000983	23.53816	0.016019	0.043277	0.735802	0.676403	33.00213	48.79063	64.41305
0.000109	237.6605	0.000801	0.501093	0.919107	0.804009	54.43379	67.70296	78.75796
0.000607	45.55046	0.006638	0.064576	0.908173	0.702737	32.15301	45.754	52.69686
0.000956	32.45071	0.017447	0.04772	0.57446	0.555006	29.40992	52.99025	61.7023
0.009343	11.48173	0.027972	0.145777	0.796235	0.726838	13.24678	18.2252	22.05741
0.000406	67.90623	0.003574	0.080187	0.570271	0.408242	23.22287	56.88513	49.26602
0.000419	39.14205	0.008434	0.032291	0.926459	0.852938	44.24222	51.87041	65.09913
0.003033	37.27024	0.015589	0.191949	0.714693	0.552302	16.06482	29.08702	27.2151
7.48E-05	475.3882	0.000804	0.453401	0.345136	0.223083	57.46208	257.5819	206.2815
0.001133	20.14829	0.018209	0.040614	0.841241	0.690947	30.9894	44.85064	54.49819

0.000195	27.18235	0.014356	0.010234	0.853232	0.755126	54.80022	72.57092	87.6094
0.000573	881.2954	0.007989	7.192577	0.560298	0.458863	29.11506	63.45051	72.51408
0.000979	31.2579	0.018916	0.048518	0.885447	0.831791	36.12607	43.43168	51.05525
0.000933	15.39971	0.009458	0.036886	0.826069	0.695478	31.48565	45.27194	48.6146
0.000208	98.39928	0.002676	0.071729	0.729579	0.591455	45.33048	76.64225	86.93796
0.001327	19.44804	0.012346	0.048435	0.971765	0.912112	35.51629	38.93852	48.69673
0.000285	35.14252	0.0049	0.031866	0.845684	0.695407	38.92579	55.97558	61.00204
0.000634	58.65106	0.010986	0.070515	0.65572	0.607355	35.32303	58.15881	67.09515
1.32E-05	1286.478	0.000665	0.266049	0.807881	0.703932	118.1289	167.8129	198.2782
0.000399	19.73419	0.01712	0.018555	0.746039	0.643879	40.02778	62.1666	69.39756
0.001429	28.48588	0.018716	0.070099	0.638138	0.560835	26.06208	46.47017	44.23422
1.86E-05	38.36826	0.002845	0.005277	0.886252	0.795211	90.27146	113.5189	130.7583
0.000152	50.92424	0.004098	0.026446	0.732573	0.710082	54.00913	76.06042	90.88325
0.000111	35.73711	0.008952	0.007855	0.826838	0.815426	71.86381	88.1304	93.99328
0.006777	24.25312	0.014645	0.338435	0.677177	0.464028	12.67109	27.30674	28.22249
0.000522	31.13423	0.012739	0.031624	0.785516	0.693338	35.74386	51.55328	52.14303
0.000106	33.10077	0.006039	0.008696	0.754705	0.685979	61.57853	89.76737	95.33002
0.000243	25.57381	0.011085	0.01093	0.805391	0.599266	46.02993	76.81051	83.39988
0.001812	8.04112	0.010452	0.038894	0.923749	0.877985	28.24725	32.17281	41.75788
0.001023	22.51186	0.010471	0.050805	0.706039	0.674684	29.55137	43.8003	50.00104
0.000193	12.80486	0.009508	0.007123	0.92533	0.829926	45.08129	54.31965	63.28675
0.01308	12.51799	0.033419	0.321443	0.80732	0.574175	11.80268	20.5559	24.19518
8.40E-05	8.596178	0.007605	0.002089	0.971481	0.807283	63.88318	79.13359	90.20307
0.000247	42.71692	0.005905	0.028841	0.888032	0.795749	50.24638	63.14352	75.94076
0.01185	42.86174	0.108669	0.400679	0.662413	0.56825	12.62262	22.21312	26.36878
0.000403	98.66629	0.005167	0.134644	0.879444	0.717129	41.475	57.83474	68.30445
0.000114	54.50163	0.003691	0.023497	0.862952	0.809706	60.1116	74.2388	89.6802
0.000357	22.5429	0.010401	0.01828	0.87751	0.852618	43.48843	51.00576	57.14332
0.000328	43.54854	0.00534	0.040347	0.692744	0.637895	39.17643	61.41513	60.76418
0.004546	27.17714	0.050036	0.14955	0.864182	0.771327	14.90776	19.32743	21.85204
0.000502	41.66308	0.012497	0.038674	0.90611	0.8616	40.18613	46.64131	51.88541
0.00058	22.99427	0.021187	0.024388	0.755044	0.624488	30.56344	48.94161	56.69948
0.000792	110.5602	0.008533	0.163009	0.902264	0.817708	33.04784	40.4152	48.32976
0.000754	35.45036	0.022255	0.037098	0.86199	0.763863	33.40883	43.73668	47.33627
0.019883	6.249414	0.04487	0.159053	0.612332	0.576859	8.988948	15.58256	17.27668
0.000619	37.31678	0.002593	0.168149	0.650235	0.425878	19.88704	46.69656	52.75335
0.007501	4.634602	0.041229	0.060044	0.773838	0.610734	11.37506	18.62521	19.82898
0.000142	90.22606	0.003049	0.426981	0.65675	0.630128	38.5034	61.10414	64.46833
5.80E-05	35.99747	0.002111	0.009066	0.790205	0.735459	62.80701	85.39842	98.06539
0.000564	12.50569	0.003145	0.029829	0.942754	0.8144	32.88315	40.37717	46.08182
0.000192	13.94162	0.007079	0.007819	0.669321	0.595783	43.3544	72.76875	72.44632
0.000774	8.94447	0.008083	0.019266	0.878231	0.695689	28.053	40.32408	50.5435
0.001136	21.40609	0.007326	0.069522	0.963652	0.828233	27.76777	33.5265	42.34837
0.000813	5.649401	0.006815	0.01372	0.955432	0.906623	28.11809	31.01408	37.76154
0.000222	33.43896	0.005171	0.028467	0.896096	0.787872	39.71685	50.41026	61.92392
4.90E-05	24.45379	0.005562	0.004187	0.858984	0.782137	77.89383	99.59105	112.6057
0.000174	27.58212	0.00726	0.014172	0.902526	0.857195	37.39066	43.61978	56.29845
0.001436	8.787306	0.012229	0.027506	0.796989	0.664587	22.23661	33.45928	39.02105
0.000616	4.172795	0.016232	0.006278	0.989508	0.902816	31.16511	34.51991	42.48464
0.001624	22.26667	0.007554	0.08921	0.943866	0.758174	18.45938	24.34717	29.10864
7.58E-05	121.8367	0.001344	0.070254	0.807624	0.752336	75.84019	100.8063	116.0116
0.00354	49.06957	0.011671	0.332995	0.802655	0.708566	18.10636	25.55351	32.32081
0.000116	766.1536	0.000878	1.106782	0.862956	0.691677	51.85115	74.96444	83.26877
0.006581	27.39841	0.015752	0.582112	0.854424	0.719761	13.1821	18.31454	20.97428
0.001524	28.34636	0.007347	0.113216	0.701259	0.514837	23.20715	45.07674	41.91172
0.000945	20.03829	0.005848	0.060894	0.907579	0.678956	30.94649	45.57953	53.06614

0.000159	27.57909	0.000974	0.037534	0.629785	0.552776	56.0139	101.3319	113.99
4.84E-05	28.63528	0.006241	0.004474	0.921022	0.76032	78.65953	103.4557	122.1333
6.42E-05	15.02287	0.003829	0.003853	0.598646	0.540007	65.98813	122.1986	133.4154
9.29E-05	1619.29	0.001047	4.301458	0.885775	0.795389	59.92074	75.33515	84.06356
0.000152	13.2194	0.004642	0.007329	0.897124	0.804315	45.36265	56.39913	70.31252
0.000452	9.826799	0.009627	0.010646	0.706372	0.593503	27.51777	46.36502	56.01811
0.000538	37.3186	0.003648	0.079435	0.900777	0.747689	27.9333	37.35951	42.89296
0.000143	71.15046	0.002632	0.250861	0.834388	0.823023	46.91291	57.00069	64.18049
0.001069	11.34304	0.028052	0.019372	0.871908	0.773438	19.88863	25.71455	32.27863
0.001032	4.815913	0.014233	0.011531	0.729555	0.570867	22.47701	39.37348	42.58443
0.000553	16.02561	0.006161	0.029275	0.883273	0.755869	22.95348	30.36702	33.55851
0.000212	8.823737	0.006997	0.005416	0.898027	0.813215	29.70373	36.52628	46.8635
0.002187	15.59634	0.018836	0.053035	0.557647	0.356964	10.13663	28.3968	17.80562
0.001547	3.456099	0.009545	0.013218	0.763846	0.697879	25.69511	36.81885	46.29675
0.00016	48.14479	0.001461	0.061458	0.95791	0.856036	40.78064	47.63896	57.48727
0.00209	17.34112	0.008594	0.085981	0.969864	0.787054	15.93684	20.24872	25.71171
0.00089	30.56512	0.003263	0.122054	0.75761	0.676574	25.05346	37.02989	43.76758
7.36E-05	26.27579	0.002063	0.010341	0.919057	0.825267	66.40945	80.4703	90.21002
0.000113	31.7266	0.005111	0.011923	0.843011	0.680663	37.52542	55.13071	63.3585
0.000239	19.97024	0.004975	0.014457	0.885889	0.804355	42.00085	52.21682	61.74663
0.000282	18.20473	0.004464	0.015091	0.581023	0.533502	39.42266	73.89408	85.12625
7.48E-05	153.7567	0.003773	0.037279	0.739134	0.658611	58.14047	88.27741	96.69021
0.000146	19.05248	0.003261	0.011084	0.611049	0.47582	42.85526	90.06617	99.26482
0.016896	27.98792	0.046916	0.842465	0.660123	0	0	14.25528	14.95423
0.00121	34.30984	0.003529	0.144293	0.863371	0.774748	33.7983	43.62488	48.8992
0.000123	28.61668	0.001945	0.01518	0.771618	0.587582	49.44177	84.14449	93.59576
8.38E-05	24.1225	0.006473	0.008883	0.934754	0.842577	63.79272	75.71145	86.58863
0.000345	32.86654	0.079739	0.023747	0.828994	0.700265	40.06349	57.21189	60.66657
0.002878	12130.43	0.360892	17.61624	0.853232	0.782902	17.87611	22.83314	26.44971
0.006741	19.98795	0.015117	0.299354	0.775317	0.675908	14.01876	20.74063	22.33092
0.000359	24.77172	0.025876	0.030824	0.638003	0.607127	32.60103	53.69718	48.88228
0.001479	43.18207	0.007195	0.206949	0.89501	0.859145	23.74551	27.63853	35.22303

PX6	PX7	PX8	PX9	PX10	PX11	PX12	PX13	PX14
40.31303	36.42928	42.04083	20737.15	29.91275	0.755654	4830.523	0.23294	20798.38
46.78686	40.18166	50.78707	29145.79	33.26202	0.761812	6012.016	0.206274	29205.77
68.34203	52.7627	71.85506	70875.45	42.60831	0.729614	11351.54	0.160162	71003.45
18.56419	16.85449	19.17783	1806.598	14.78495	0.821399	873.3152	0.483403	1841.956
33.67329	29.45012	36.34938	11216.77	25.08746	0.800746	3026.224	0.269795	11289.98
50.45007	51.61354	51.65343	38741.67	36.82114	0.753699	7346.351	0.189624	38857.19
46.90781	36.67893	49.437	23586.27	32.10469	0.740704	5369.644	0.22766	23710.24
50.625	51.57315	51.57315	35926.84	37.83945	0.724143	7271.198	0.202389	36045.59
19.34678	19.35294	22.21785	2240.221	15.20386	0.78547	1054.1	0.470534	2285.962
69.88346	64.81427	73.47148	94029.58	49.76746	0.700529	14274.73	0.151811	94240.49
138.5188	110.7534	167.9032	421486.7	79.84816	0.68528	39670.91	0.094121	421931
144.5496	112.1091	153.6942	794377.2	98.78524	0.711488	58299.86	0.073391	794769.8
56.71169	59.81176	63.69338	67921.5	44.14062	0.743007	10835.01	0.159523	68062.43
30.77768	28.89907	34.7873	8848.551	23.91499	0.781348	2647.814	0.299237	8914.063
21.81798	22.99202	25.07255	3429.251	18.51767	0.757878	1451.056	0.423141	3482.092
97.90399	370.7447	371.318	246495	69.64405	0.681628	27891.74	0.113153	246775.6
30.4226	25.57283	31.75429	7782.981	22.50744	0.778453	2439.772	0.313475	7841.12
68.25533	73.06197	74.26545	100928.7	54.85547	0.692565	15136.78	0.149975	101138.5
55.68624	48.94222	58.98963	53862.16	40.273	0.734163	9394.655	0.17442	53993.34
92.51466	85.6644	98.0009	298665.6	73.58204	0.734866	29403.52	0.09845	298919.4
100.6406	103.8683	116.8733	387554.5	75.51803	0.752869	34144.42	0.088102	387872.3
67.58725	71.06701	72.88573	108676.7	52.29519	0.736208	14959.18	0.137648	108850.3
28.27344	28.629	28.65196	4774.921	23.79867	0.738279	1857.411	0.388993	4837.201
72.37281	82.7016	86.02603	151044.3	58.76174	0.746975	18361.75	0.121565	151196.1
61.19516	62.22193	73.35167	85704.55	47.34547	0.738638	12726.88	0.148497	85847.75
30.68192	32.5025	36.81016	11008.54	25.20311	0.749685	3192.213	0.289976	11093.57
48.63112	47.34612	54.36402	36819.81	38.12152	0.74304	7203.21	0.195634	36914.44
57.56726	50.31205	58.44007	41977.77	38.98468	0.762803	7657.463	0.182417	42099.29
40.60518	40.85469	46.11726	22021.57	29.62732	0.748675	5074.849	0.230449	22119.51
51.5876	45.49572	55.58033	44284.94	38.97602	0.775932	7801.254	0.17616	44409.81
30.99023	32.81683	34.83374	6711.145	21.92645	0.780669	2204.018	0.328412	6790.179
73.44068	74.58295	79.20945	129402	60.10834	0.694669	17810.12	0.137634	129639.2
37.7352	275.0659	276.9535	11831.74	27.28002	0.757944	3312.935	0.280004	11937.95
78.52819	70.21165	83.45348	105699.9	49.73596	0.656647	16464	0.155762	105862.1
158.6638	141.1501	158.8639	957355.6	105.6394	0.623691	75317.68	0.078673	958043.2
40.96584	45.19836	46.29138	13807.42	27.63358	0.698251	3986.094	0.288692	13910.08
103.7692	108.3258	116.767	314468.5	68.92835	0.703603	31783.97	0.101072	314760.7
30.60513	30.27801	33.20087	10799.6	24.03277	0.787081	3001.947	0.277968	10882.08
38.15836	39.7499	45.42878	14948.49	26.75273	0.728048	4030.779	0.269645	15049.99
25.3415	19.89129	26.0048	3604.402	17.27895	0.746159	1523.613	0.422709	3653.895
70.39433	56.01612	74.87899	48407	37.11036	0.638953	10052.81	0.207673	48566.25
107.9995	104.7123	120.5069	473588.7	86.99911	0.743721	39507.07	0.083421	473844.2
39.77255	38.02129	41.84717	22544.85	32.06337	0.761336	5069.198	0.224849	22629.33
76.89609	75.31503	83.58312	132779.7	60.98109	0.667732	18849.64	0.141962	132951.3
70.22325	84.42299	90.31673	114745.6	53.54345	0.607657	18792.41	0.163775	114994.4
31.5919	27.72578	33.51985	9586.157	23.21899	0.793735	2749.402	0.28681	9654.449
40.7575	30.75622	41.51651	12920.19	26.37194	0.750359	3548.633	0.274658	12981.66
55.16445	61.9364	70.73413	62441	45.0809	0.699154	10886.57	0.17435	62577.85
52.04569	54.70907	55.26305	49679.87	40.33747	0.751535	8696.044	0.175042	49812.4
84.99032	78.58119	87.93669	97979.17	49.02159	0.659901	15574.98	0.158962	98187.09
79.84112	76.67109	82.52211	130516.8	59.45631	0.652917	19057.71	0.146017	130732.8
28.81386	27.55234	30.15947	7892.483	22.74398	0.811575	2362.099	0.299285	7959.409
42.53452	37.43242	43.97927	23051.24	30.69938	0.786187	4982.199	0.216136	23152.83
54.1875	40.56323	57.53651	27401.26	31.33393	0.651077	6750.956	0.246374	27512.58
97.2007	78.76575	100.2203	207839.2	62.4009	0.741249	22891.36	0.11014	208084.2
50.12392	52.7747	55.43039	45282.62	42.41229	0.753014	8158.973	0.180179	45399.79
51.43174	39.92846	55.26331	36199.8	35.56458	0.758693	6975.177	0.192686	36311.33

37.69661	35.90685	40.84443	14548.59	28.24163	0.707376	4074.252	0.280045	14649.68
31.48593	32.04152	35.66807	11069.18	25.87069	0.755679	3178.512	0.28715	11147.45
48.78461	44.88832	49.47642	25772.24	35.35314	0.713149	5916.596	0.229572	25871.09
136.4063	134.7626	145.2069	872289.1	107.1921	0.736059	59980.76	0.068762	872583.8
32.35227	31.82026	34.59199	10737.73	26.31798	0.797925	2949.829	0.274716	10803.49
66.50016	69.33896	75.21137	91243.81	56.67474	0.677692	14462.86	0.158508	91595.9
93.86281	94.01117	101.5302	200164.5	67.90411	0.659239	25101.48	0.125404	200467.5
103.9275	87.09341	109.5949	251275.5	71.19388	0.667339	28856.11	0.114839	251668
35.94568	32.31197	39.08579	12611.15	26.64303	0.790596	3314.104	0.262792	12690.46
33.65497	29.8603	34.49604	8204.373	22.45777	0.782452	2514.143	0.306439	8281.806
30.7614	29.50027	31.55084	7265.502	22.40011	0.751643	2413.513	0.332188	7342.301
75.37666	80.22791	88.50658	162798.2	64.67268	0.709623	20318.41	0.124807	163006.8
137.1054	135.2603	137.9428	783377.9	110.4756	0.688441	59694.11	0.076201	783841.6
87.85737	88.26687	92.95272	249061	69.93678	0.736163	26004.43	0.10441	249295.8
121.6265	115.2662	122.36	469586.4	91.59349	0.677729	43109.38	0.091803	469858.9
106.6638	104.7161	108.2191	254985.1	69.23603	0.69935	27805.61	0.109048	255213.8
21.11226	20.85199	22.06215	2975.138	17.62758	0.77814	1285.576	0.432106	3022.911
48.03417	41.74962	51.34453	30458.93	36.37375	0.787547	5988.949	0.196624	30568.46
90.84913	88.60651	94.60898	242925.8	68.67776	0.658945	28572.68	0.117619	243213.5
52.25482	41.19329	52.4065	30276.98	35.05073	0.748673	6274.807	0.207247	30374.63
93.55206	76.32103	93.80148	159482.5	62.76917	0.673321	21122.11	0.132442	159723.3
57.31956	51.01826	60.80096	45742.38	38.80442	0.713612	8667.65	0.189488	45872.55
51.92594	49.58294	52.21312	34558.23	36.02647	0.714894	7177.01	0.207679	34676.03
69.67002	65.31456	71.72989	86773.04	48.34536	0.684441	13848.56	0.159595	86921.58
31.87579	29.2694	34.98865	9729.937	23.54355	0.772334	2853.769	0.293298	9808.838
91.91804	80.66512	96.01327	203491.3	67.73276	0.701679	23843.85	0.117174	203791
153.8116	109.156	158.3414	657664.2	86.76363	0.677982	53943.01	0.082022	657960.2
66.77398	66.20563	68.93381	97296.88	51.79634	0.759832	13463.75	0.138378	97520.28
57.76754	38.74189	57.90078	25512.97	30.42043	0.694436	6035.209	0.236555	25611.35
55.20322	48.50199	55.57077	48947.56	41.4639	0.772371	8378.097	0.171165	49069.56
28.1654	22.75642	33.09182	5720.901	20.27662	0.7563	2045.345	0.357521	5786.261
64.20834	66.31212	70.32516	83752.89	47.09894	0.698793	13247.54	0.158174	83955.19
105.6264	81.82712	108.051	290279.5	69.78564	0.758861	27938.26	0.096246	290537.1
59.14128	59.4402	62.70894	70028.67	48.70736	0.726509	11309.07	0.161492	70172.31
43.34938	42.28974	48.03428	25824.27	36.19427	0.737012	5732.73	0.22199	25922.58
62.76014	57.91893	66.92609	60856.14	41.43316	0.736963	10152.53	0.166828	60937.14
33.3223	32.84225	37.61761	11312.52	24.49396	0.752828	3237.136	0.286155	11395.55
68.83223	63.86199	74.92286	99192.72	54.03979	0.73976	14008.12	0.141221	99353.17
24.01248	20.55435	27.47534	3354.284	17.18343	0.740724	1462.943	0.436142	3390.268
24.48651	23.52198	26.64343	5058.744	19.59929	0.774879	1839.129	0.363555	5096.69
36.45763	37.79368	44.0355	16371.93	28.12454	0.746283	4178.114	0.2552	16443.35
47.35691	46.57588	48.87202	37659.35	37.2711	0.77159	7041.736	0.186985	37736.07
31.68123	30.55409	33.64005	7912.633	24.08346	0.732101	2622.973	0.331492	7997.154
60.1269	60.81261	64.47523	83938.96	47.02833	0.759117	12212.87	0.145497	84043.9
59.15468	59.12549	67.08261	68245.27	47.71525	0.751332	10748.98	0.157505	68454.68
36.6622	31.72674	38.19107	12871.76	27.02863	0.759271	3498.211	0.271774	12924.41
49.40089	40.75274	49.78252	23925.29	35.70161	0.725274	5536.311	0.2314	24030.92
53.13765	53.77698	56.55264	47677.18	38.99685	0.762071	8343.773	0.175006	47809.8
32.47612	24.58177	34.62924	7076.316	19.71981	0.765766	2327.695	0.328942	7123.594
44.60399	40.53381	49.26968	24728.92	35.76193	0.740559	5542.786	0.224142	24830.06
87.10846	87.72602	90.22249	103591	54.38912	0.664104	16061.86	0.155051	103739.5
91.49693	120.6237	134.2307	246573	71.94451	0.654568	29050.91	0.117819	246766.8
94.33799	88.16917	110.1237	235174.3	68.47589	0.714584	25784.41	0.10964	235438.7
37.74301	43.73044	45.00925	13830.61	28.6659	0.786995	3540.567	0.255995	13883.88
41.29872	44.1878	45.92384	21146.24	31.56613	0.735605	5027.225	0.237736	21211.28
116.5772	108.9897	122.4009	524540.8	92.57997	0.734173	42842.2	0.081676	524991.7
49.80847	47.43829	58.54033	33088.08	32.81322	0.718458	6937.407	0.209665	33170.62
63.35462	52.28984	64.85947	59662.28	45.59992	0.694864	10626.35	0.178108	59763.57

35.25999	33.11159	36.35929	13268.91	27.36219	0.78687	3444.593	0.259599	13364.74
38.51491	30.06717	39.27686	13388.8	26.26155	0.760631	3584.852	0.26775	13455.5
115.2798	85.46041	124.0187	301871.7	68.79912	0.625785	34775.53	0.1152	302210.3
34.91619	28.26286	35.87899	10025.64	23.71514	0.755893	2974.623	0.296702	10075.63
57.97978	53.4489	60.04827	50858.13	45.04373	0.682973	9719.734	0.191115	51002.51
74.41046	69.40263	85.66472	128987	53.88863	0.655147	18844.14	0.146093	129128.4
29.22504	28.82135	31.55965	8813.706	23.93777	0.783746	2632.78	0.298714	8893.349
63.35462	60.67063	68.51779	92453.44	49.93998	0.751688	13154.12	0.142278	92635.3
32.18714	29.99434	34.65363	10466.35	24.08765	0.780842	2963.36	0.283132	10540.18
38.80234	38.21881	39.49203	20450.41	31.0995	0.76868	4704.784	0.230058	20510.34
55.36654	45.26655	56.92083	45255.5	39.32987	0.754374	8141.005	0.17989	45331.26
44.35002	51.68896	52.17826	33670.67	33.65014	0.759407	6640.145	0.197209	33789.91
58.91829	54.55442	62.49182	60367.86	43.57658	0.730732	10184.28	0.168704	60459.33
66.01965	60.86502	67.28784	80436.95	48.30176	0.711228	12670.07	0.157515	80557.41
87.55628	84.46065	103.3188	204568.4	59.3563	0.658562	25494.51	0.124626	204822.6
45.56827	37.91143	47.09021	25725.31	32.44504	0.767156	5493.395	0.21354	25793.45
79.74489	76.90342	79.87575	140549.8	60.31346	0.751144	17403.95	0.123828	140803.8
153.4066	135.6955	159.5812	673967	93.5997	0.645977	57547.47	0.085386	674408
26.21866	28.53716	30.77151	5820.258	20.90084	0.767894	2037.721	0.350108	5906.105
43.07946	39.02141	43.40595	19053.02	31.77514	0.722538	4774.554	0.250593	19133.14
32.42396	28.52224	33.04573	9020.586	24.03218	0.762679	2747.676	0.304601	9066.667
47.51632	47.37544	53.4498	36440.69	36.95162	0.719781	7384.851	0.202654	36522.6
39.25424	34.66239	43.72959	15587.15	28.85075	0.729774	4134.98	0.265281	15642.1
39.28985	35.41871	43.1173	16610.69	30.21348	0.75532	4168.166	0.250933	16725.27
68.08095	67.42653	83.11654	115124.6	54.69224	0.761407	15030.71	0.13056	115234.2
44.01863	45.8628	48.94635	30950.64	34.5454	0.739403	6447.369	0.208311	31035.86
44.74632	43.2761	48.41053	33498.92	35.77088	0.784352	6407.079	0.191262	33611.13
78.18136	71.42383	82.15545	120050	53.61839	0.675851	17413.05	0.145048	120287.9
36.01757	32.07514	38.96128	11736.39	24.79971	0.750006	3329.986	0.283732	11850.4
50.45892	38.34424	51.99922	26006.94	31.2656	0.758223	5598.604	0.215273	26201.51
22.26733	24.08897	27.49611	3298.691	14.95863	0.787696	1360.462	0.412425	3378.43
48.72042	34.02369	49.05422	20378.83	29.3365	0.763827	4723.622	0.231791	20497.95
81.44818	68.95947	81.72805	135228.8	57.86229	0.725809	17553.94	0.129809	135524.3
112.0795	76.10658	112.1876	218437.1	64.9486	0.727247	24118.65	0.110415	218761.6
37.08239	34.13598	41.01213	13564.48	25.19283	0.719079	3825.099	0.281994	13654.91
32.26765	21.62452	36.67367	5639.329	18.37859	0.704045	2176.215	0.3859	5723.222
43.22499	39.22123	44.50424	23509.53	32.54041	0.777155	5106.686	0.217218	23636.8
63.61202	57.35568	66.27488	73899.97	47.50297	0.769536	11066.7	0.149752	74096.9
79.20072	77.64041	81.98302	117649.2	59.5448	0.67079	17309.72	0.14713	117944.1
54.24516	48.62342	62.29539	48756.44	41.71312	0.723455	8921.286	0.182977	48887.56
58.24254	62.25734	62.25734	54207.13	43.13797	0.688104	10066.24	0.1857	54413.22
37.96193	27.13868	39.58239	8281.02	22.12473	0.704246	2810.706	0.339415	8400.703
58.28287	57.46459	59.30031	52812.06	42.78847	0.68082	9998.634	0.189325	53077.57
26.91233	21.52298	29.38019	4384.111	17.58499	0.7585	1707.85	0.389554	4464.294
64.03283	47.96136	64.78519	37078.02	34.7868	0.677906	7932.179	0.213932	37229.31
74.3141	123.3555	124.6785	37908.72	41.45518	0.697923	7819.323	0.206267	38128.21
41.99774	37.33391	42.85082	21804.5	31.35296	0.817069	4619.441	0.211857	21903.32
96.71472	75.65132	101.575	202820.8	65.56212	0.70906	23543.77	0.116082	203122.6
60.92601	44.82569	64.57251	36874.43	35.90025	0.681384	7862.769	0.213231	37068.86
83.26878	74.28622	86.47359	153628.6	62.22628	0.691764	20052.76	0.130527	153792.7
58.87806	49.52416	58.9452	43819.18	41.55252	0.771306	7792.925	0.177843	43994.06
56.58494	37.5215	62.59853	32322.56	30.44076	0.745911	6578.612	0.20353	32478.33
21.54721	18.56303	23.866	2610.506	14.51155	0.790404	1159.983	0.444352	2680.801
59.43395	44.08092	63.39042	29041.02	32.43994	0.658123	6942.537	0.23906	29148.84
64.27524	68.85768	70.47255	71747.42	48.05582	0.696586	11987.08	0.167073	72000.16
37.80258	33.45154	38.73548	5640.224	20.78829	0.650327	2356.224	0.417754	5731.294
106.5532	176.741	238.4329	354344.7	88.90087	0.606427	39932.14	0.112693	354891.7
51.75379	42.16445	56.68289	36532.31	37.7302	0.773705	6881.663	0.188372	36718.69

82.81983	78.25779	89.42894	164086.7	61.91985	0.71	20414.62	0.124414	164400.7
72.06263	43.38274	72.90817	42560.19	35.55122	0.66513	8862.987	0.208246	42728.46
55.14202	44.49824	56.30694	42829.31	38.45647	0.778458	7604.603	0.177556	43027.53
54.8863	46.9201	57.57235	36377.29	37.39777	0.755414	7028.342	0.193207	36548.63
88.35938	70.32656	88.76131	130244.3	55.91657	0.65809	18881.55	0.14497	130547.2
46.38515	46.73965	49.08762	36455.98	37.8391	0.748043	7107.824	0.19497	36697.2
62.4094	65.20139	66.98499	68984.55	47.33766	0.701756	11591.3	0.168028	69186.65
64.68528	45.62231	67.57713	54397.06	38.13592	0.747812	9284.142	0.170674	54584.31
198.7335	160.5272	199.4398	1848005	135.5729	0.685388	106255	0.057497	1848862
60.23574	71.11302	79.25376	67963.07	46.37872	0.659776	12206.84	0.17961	68232.51
54.17734	38.21912	54.56776	23945.04	29.65436	0.705426	5695.211	0.237845	24109.14
135.9503	118.0729	141.2678	716835.8	100.6064	0.695925	55658.92	0.077645	717306.2
88.90172	70.00558	96.21144	150763.2	55.71982	0.647928	21142.41	0.140236	151066.4
100.025	100.4909	112.3596	327613.4	72.8696	0.755279	30428.79	0.09288	328041.4
23.10823	28.69922	31.53193	4588.818	18.4915	0.740547	1803.29	0.392975	4683.06
59.87798	59.44444	62.21594	50009.5	40.49593	0.692688	9476.504	0.189494	50240.2
102.4361	84.81772	104.2938	261048.1	67.74791	0.70951	27840.23	0.106648	261467.8
90.55697	79.07853	94.6448	145954.7	61.86249	0.665119	20155.64	0.138095	146365.7
39.9661	42.1105	42.13156	14894.35	29.71959	0.670103	4368.75	0.293316	15032.04
53.06614	40.76306	54.84826	25885.37	30.92471	0.703236	6017.542	0.232469	26040.48
67.54319	76.73227	81.99328	78085.18	50.2636	0.691403	12778.07	0.163643	78263.81
23.10823	20.67953	25.54688	3067.657	16.59519	0.775743	1316.147	0.42904	3144.287
96.39503	92.94221	96.92892	261234	76.87676	0.692611	28533.04	0.109224	261581
81.13032	64.35419	82.6068	115710.1	56.07349	0.68353	16799.92	0.14519	115990.1
24.75071	16.53041	26.66124	2832.476	14.71425	0.762935	1268.939	0.447997	2913.483
63.55171	65.63451	72.16571	83594.77	50.86244	0.725115	12750.58	0.152528	83853.44
82.90682	80.98281	93.95999	202882.4	64.06454	0.777371	21479.23	0.10587	203126.4
60.75203	57.28226	64.2021	68983	44.75808	0.721809	11269.12	0.163361	69194.73
59.27973	68.69067	78.29809	65859.16	42.54494	0.669939	11772.25	0.178749	66204.3
23.84736	20.55739	24.68738	3429.384	16.70241	0.803663	1368.424	0.399029	3478.612
54.92288	53.12335	55.9738	56272.22	42.26214	0.792179	8964.443	0.159305	56450.24
55.75919	44.49194	57.35876	35601.55	36.95306	0.674272	7761.78	0.218018	35725.11
48.82622	42.22725	50.49892	34319.94	36.4652	0.780419	6544.158	0.190681	34460.78
52.22374	48.29732	55.75887	38984.86	37.7006	0.751509	7398.564	0.18978	39131.64
17.27668	10.59958	17.55564	909.8167	9.541697	0.801136	566.779	0.62296	941.3206
33.94052	55.10381	57.79406	18448.92	30.36376	0.689004	4900.526	0.265627	18553.9
21.15252	18.7038	22.37921	2120.051	14.4129	0.74704	1068.327	0.503916	2163.324
68.55246	49.13986	69.07301	61864.9	40.13017	0.703911	10746.39	0.173707	61983.19
94.62077	84.73017	101.5118	250907.3	67.48229	0.709761	27104.87	0.108027	251103.9
48.67438	44.25024	49.92261	36628.73	38.06573	0.799503	6671.321	0.182134	36736.3
73.56273	73.94045	90.88642	104501.7	48.70569	0.699217	15344.57	0.146836	104704.6
45.46775	41.70208	51.90377	26650.63	35.41385	0.731667	5897.152	0.221276	26764.17
38.23905	39.92059	42.8232	20709.36	32.30788	0.754437	4833.988	0.23342	20830.61
39.76141	34.23286	40.0402	18224.11	29.63184	0.775689	4317.453	0.236909	18309.34
53.8057	53.83148	62.83049	64423.38	45.17242	0.778142	9987.443	0.155028	64564.02
115.6495	107.7754	123.7706	462407.2	85.54712	0.672273	43015.13	0.093024	462799.7
53.8145	47.14423	56.62597	44814.52	39.36797	0.732232	8332.619	0.185936	44890.31
33.36754	38.78544	39.32371	13806.56	26.66667	0.759108	3666.379	0.265553	13897.09
42.48464	39.19449	44.08968	26063.66	34.15774	0.796741	5335.692	0.204718	26184.48
32.77636	28.85748	32.80438	7032.44	22.98048	0.751049	2363.488	0.336084	7098.851
118.2108	101.9119	120.2035	417411.1	81.41355	0.687887	39265.36	0.094069	417852.8
26.16721	29.30437	34.27329	6690.571	20.51067	0.747582	2296.858	0.343298	6800.262
90.35076	99.27512	103.0877	155796.5	64.69102	0.651362	21496.46	0.137978	156030.5
21.74718	20.67564	23.62501	2717.092	15.64839	0.761824	1236.039	0.454913	2777.16
42.8302	49.44683	56.29778	23047.61	31.61046	0.708132	5530.793	0.239972	23204.13
55.01026	46.06408	56.57426	41377.84	41.36701	0.753837	7674.528	0.185474	41557.98

106.8826	77.82	115.6986	253918	63.8173	0.738034	26274.63	0.103477	254225.4
128.4167	113.4354	132.078	525353.5	95.28501	0.602897	52224.61	0.099409	525776.1
128.8837	95.42877	136.1581	391020.2	73.15376	0.632355	40893.68	0.104582	391319.1
87.26654	90.65804	91.53387	199338.4	66.73	0.65017	25381.55	0.127329	199469.5
60.50443	63.07063	71.68799	90501.4	50.597	0.743793	13105.97	0.144815	90663.15
41.99636	47.60093	56.13264	28463.43	32.75094	0.708824	6360.193	0.223451	28592.85
39.90127	41.70972	50.49715	24363.97	33.65259	0.736291	5519.927	0.226561	24459.83
60.35092	65.57655	66.92646	89781.05	47.56072	0.73123	13260.3	0.147696	89945.4
29.75527	28.94616	32.65703	7860.247	22.42074	0.760036	2515.403	0.320016	7917.696
44.92583	36.27423	45.63873	17613.07	28.72514	0.727203	4501.789	0.255594	17709.67
37.94996	33.15958	38.10357	13174.27	26.82237	0.749936	3597.031	0.273035	13254.98
43.46042	41.18743	48.78091	24955.23	32.80157	0.764761	5400.071	0.21639	25021.34
18.24881	12.87261	144.2696	1016.176	15.83538	0.743809	657.1562	0.646696	1035.45
39.03558	35.63929	46.82987	16436.6	28.12392	0.682241	4582.344	0.278789	16542.66
58.73723	57.11747	59.31487	62545.88	45.63386	0.743042	10255.02	0.16396	62671.79
25.71171	22.68436	26.99571	4264.226	19.63851	0.753026	1688.758	0.396029	4320.873
43.41946	33.03514	45.58064	17373.88	28.05421	0.73245	4428.981	0.254922	17464.54
94.991	90.13079	95.08884	278461.9	73.95679	0.718658	28694.98	0.103048	278706.5
59.9398	55.78021	65.57596	67734.25	46.47578	0.744841	10788.46	0.159276	67826.94
57.20316	57.56006	63.59406	71844.32	46.25833	0.743062	11247.45	0.156553	71985.29
70.80353	59.28607	86.7118	80513.24	42.93417	0.684198	13178.94	0.163687	80695.8
96.93773	88.77334	105.4247	229315.8	65.2488	0.68964	26271.45	0.114565	229533.5
107.3755	66.13873	108.9502	148682.1	55.03481	0.685873	19788.48	0.133093	148934.3
11.42981	14.32099	15.56363	714.5245	9.410239	0.803478	481.0477	0.673242	767.1533
51.57377	50.18828	53.42994	38908.63	37.66447	0.737023	7534.145	0.193637	39128.18
89.70904	81.70167	101.7851	179193.8	64.92739	0.676541	22719.85	0.126789	179496.8
93.89594	85.08413	94.85149	243228.4	70.77156	0.732902	25710.71	0.105706	243478.5
63.14441	66.55595	71.39793	70797.98	47.42832	0.641795	12895.42	0.182144	71027.52
28.13002	24.79515	29.31265	4999.351	19.48197	0.727966	1942.297	0.38851	5074.644
20.63225	23.16455	24.56086	3129.085	16.08057	0.754764	1370.728	0.43806	3189.045
57.68398	59.15192	61.50008	42126.58	34.25899	0.699904	8365.332	0.198576	42248.28
33.32813	32.61251	35.32901	10888.98	24.73676	0.71136	3339.794	0.306713	10979.22

PX15	PX16	PX17	PX18	PX19	PX20	PX21	PX22	PX23
9	34	10367397	1.178596	13	6.599784	62	8.213237	21.25644
9	36	19037865	1.058573	14	2.88031	60	8.203315	22.0667
28	64	92729380	1.41152	19	3.171006	105	11.297	46.82283
26	44.3	1324995	0.482809	9	3.840041	55	5.718731	35.33301
14	48	6286378	1.249227	16	5.048075	76	10.85229	33.02525
3	28	6575286	1.058503	13	6.045571	57	8.390173	15.16307
0	36	4181340	1.451879	18	6.848578	82	11.59558	17.97025
-2	31	7093283	1.407013	16	8.541025	67	11.5184	14.17686
0	38	701613	1.482238	15	6.958541	54	12.04743	22.11638
8	46	38403137	1.532746	19	6.135194	130	12.37808	27.41012
12	44	1.76E+08	1.326045	16	13.93538	85	10.71983	28.51207
32	63	1.11E+09	1.326496	15	5.603862	183	9.895162	47.4979
15	42	30486036	1.204421	14	9.65293	82	9.489661	28.45008
7	40	3294144	1.328251	17	4.365407	69	10.53395	23.18887
19	43.3	1800879	0.990838	12	8.763486	58	8.258744	31.43867
20	46	2.16E+08	1.030387	14	970.0842	101	9.410715	32.32921
-2	29	1550948	1.269451	15	4.771978	52	9.85188	14.43021
7	39	35227299	1.399812	16	7.569442	68	10.68655	23.35276
21	44	27007915	0.924166	12	14.85807	66	7.638646	32.34665
11	46	1.19E+08	1.328125	19	7.717804	78	11.21125	29.2632
15	44	1.64E+08	1.173117	16	3.537585	80	9.001086	28.97855
7	44	41205791	1.445009	19	5.720794	77	11.75808	25.8948
22	46	2885864	0.905059	12	5.721702	71	7.689236	33.58173
18	42	99781308	1.060533	13	6.415458	72	7.903703	29.89181
26	51	76349802	1.076578	13	21.56658	79	8.755603	38.4278
18	45	5376547	1.10952	12	9.655837	131	9.369282	31.79901
33	53	44570532	0.908307	10	26.04864	195	6.480604	43.49663
16	43	25645908	1.079568	14	3.116375	68	8.539296	29.97524
10	44	10804742	1.350751	17	7.685623	67	11.5965	28.32237
6	44	16263105	1.493949	19	6.238381	73	12.46293	25.68287
13	41	2152506	1.223145	13	8.751076	63	9.361009	26.96172
15	46	72875962	1.252419	16	8.158782	97	10.12897	30.561
11	39	28050926	1.186166	14	154.1049	63	16.48531	18.67286
17	40	59880981	1.119124	12	15.20684	112	7.833625	28.11735
17	42	3.59E+08	1.032063	13	5.895616	230	7.933472	29.726
20	50	8827040	1.241008	15	17.25213	72	10.11824	34.22754
18	44	1.12E+08	1.000475	13	11.3003	227	8.04041	31.30545
1	26	1078814	0.965549	12	7.216875	51	8.005638	13.08284
25	48	8222300	0.798801	12	3.627676	69	7.215079	36.53218
-16	32	969085	1.6906	21	5.342803	50	14.95864	12.44591
13	46	26859944	1.380736	16	17.99631	73	11.84222	29.32304
17	55	3.73E+08	1.444865	20	3.489252	172	11.83679	37.35568
7	38	8120982	1.244906	16	3.336254	62	9.608355	22.76108
19	45	86715413	1.093313	14	8.92145	123	8.579476	31.842
21	48	57279750	1.036927	14	5.326943	71	8.559269	34.60894
13	36	3026138	1.088554	12	3.46721	58	7.337408	24.6391
15	42	8057576	1.146559	14	9.8948	65	9.036247	28.76181
12	42	32280843	1.116687	16	3.238773	61	9.367997	27.61251
14	47	23985839	1.285069	17	3.41703	77	10.25023	30.50484
19	45	49520825	0.977959	13	4.080684	67	8.044689	32.24039
24	52	1.03E+08	1.119777	14	8.252498	77	8.977127	38.12175
8	39	2626344	1.385318	15	5.585713	66	10.07279	24.4012
5	34	4672275	1.270318	15	6.006798	60	9.316821	19.6257
20	44	15824972	0.882698	12	5.644617	62	7.563445	32.19638
21	56	1.61E+08	1.47661	17	52.4916	487	11.7719	39.23008
21	42	25167388	0.845958	11	4.50333	62	6.870734	31.50132
16	42	19680165	1.139979	13	9.315717	64	9.114382	28.81233

3	36	3598045	1.454318	16	8.310491	60	11.19875	20.19708
19	44	4874887	1.042999	12	3.605193	72	7.733913	30.393
9	45	15137408	1.54179	17	13.56278	176	12.0557	26.14876
31	86	1.91E+09	1.869992	27	3.287467	238	16.64331	56.53882
13	46	6404097	1.324977	17	15.55508	67	10.59541	29.44571
19	45	29209377	1.026222	12	18.37855	64	8.849592	31.61871
16	44	89665618	1.140685	14	6.950875	123	8.965561	30.3824
18	51	79962030	1.265921	18	4.610962	220	10.13571	34.78341
19	46	6532985	1.043446	14	5.159979	72	8.525949	32.63032
18	43	2739528	0.983653	13	3.489639	60	8.152559	31.05864
15	42	2291986	1.056774	13	3.451528	58	8.341758	28.89495
4	37	37126868	1.285906	17	3.09401	71	10.25518	20.34348
12	39	2.3E+08	1.171255	15	4.093011	188	8.751371	25.37839
13	42	1.2E+08	1.143657	16	4.60184	75	9.257844	27.60933
20	49	2.68E+08	1.166069	15	6.3738	88	9.383818	34.30755
23	51	1.54E+08	1.047678	14	17.28979	331	8.508726	37.0338
20	48	1898652	0.992868	13	5.136396	62	8.761973	35.26477
17	36	9945715	1.048029	9	7.481083	60	6.124362	26.31759
25	47	1.44E+08	0.774384	12	11.50135	75	7.008886	36.03935
7	40	10085754	1.33397	17	3.771358	69	10.37959	23.536
15	43	67423835	1.211251	14	47.43784	383	8.952359	28.76769
13	37	14276588	1.116048	12	3.574734	73	7.568622	25.15978
8	33	7786510	1.081108	13	4.685588	58	7.909081	20.50104
15	40	39595482	1.092795	13	9.798828	107	8.07298	27.789
22	48	5154534	1.041757	13	9.226625	101	8.941092	35.3213
20	52	1.24E+08	1.250092	16	34.45041	341	10.04732	36.13882
22	51	4.18E+08	1.126294	15	3.192622	82	9.058005	36.23665
8	44	19682831	1.41752	19	3.766084	80	11.32506	26.30885
20	43	13221546	0.967083	12	8.772985	64	7.992114	31.23293
6	33	10110403	1.208609	14	6.756616	61	8.889831	19.38491
4	36	1491670	1.448667	15.25	13.21916	56	11.26198	19.52334
20	47	46039780	1.06624	13	13.50781	80	8.901899	33.44746
8	34	50647267	1.108022	13	14.84294	74	8.202887	20.27145
27	50	70613842	0.971815	11	131.1426	518	8.897381	40.46642
12	38	14001393	1.194436	13	6.764003	60	8.426106	24.65641
12	33	41715341	1.078129	11	6.492338	61	6.658203	22.23859
16	44	4782399	1.181912	13	17.77097	171	9.38723	30.33673
26	47	82978593	0.692274	11	26.82916	181	6.63381	37.11767
19	44	3168736	0.956848	13	3.945647	65	7.939152	32.02787
0	36	2665348	1.359867	19	3.058105	60	10.9541	18.78199
2	38	8957142	1.461751	19	4.120113	67	11.75273	19.99012
18	47	43944252	1.221082	15	4.544711	90	9.355984	32.50431
15	37	3228111	1.150851	11	26.87383	51	8.727181	25.3871
19	50	92191113	1.261509	16	5.604419	84	10.05526	34.30831
10	46	21503256	1.379855	19	4.78661	76	11.40087	28.48544
17	45	11744079	1.098239	14	4.785503	69	9.027119	31.23441
19	43	12363487	1.084269	12	41.50747	282	8.352127	32.01663
31	55	40722011	1.101163	13	7.298412	85	8.060991	42.97077
47	80	22853215	1.299015	16	3.502255	103	10.15846	64.35193
29	61	25746420	1.353267	18	15.92877	90	10.9494	44.86652
15	47	1.03E+08	1.251487	17	4.842044	117	9.954899	31.28209
15	41	2.19E+08	1.112447	13	6.475988	75	8.321464	28.34523
10	36	65468108	1.20928	14	20.71185	236	8.613952	22.80765
24	49.8	18722339	1.015993	13.5	10.14528	71	8.469013	36.24244
0	24	4867392	0.88152	12	5.072412	51	7.557785	11.55141
11	37	94370393	1.149595	14	13.3654	111	8.350261	23.66522
6	49	27850206	1.648289	22	7.011075	94	13.85555	27.50566
16	45	62409403	1.168167	14	5.310317	79	9.13851	30.67396

7	41	3550619	1.419337	15	6.921833	84	11.32976	24.73588
9	38	7808284	1.226637	15	3.595577	67	9.203158	23.22563
14	45	1.87E+08	1.33988	15	126.985	614	10.80146	30.21698
21	46	14826327	0.975187	13	6.572707	68	7.981024	33.21744
14	52	32174264	1.52582	20	7.698439	99	12.42635	32.54567
26	47	1.9E+08	0.721446	11	14.71676	73	6.822916	36.59212
16	42	2978988	1.067812	13	3.353105	64	7.856206	28.7092
21	48	53116094	1.054963	13	8.596196	100	8.878288	34.46515
17	41	4246854	1.061688	13	4.159358	64	7.761648	28.63995
15	40	17255448	1.084239	12	4.815405	73	7.91829	27.66918
13	40	36476060	1.130719	13	5.653563	64	8.362133	26.73892
-1	51	17068807	1.814754	22	6.173989	82	16.42129	27.82536
20	58	1.17E+08	1.542984	18	25.10304	314	12.17532	41.29403
25	48	1.11E+08	0.835183	12	8.382639	77	7.457794	36.41838
14	49	89484128	1.361309	17	5.037185	117	11.25757	32.92858
6	45	21363170	1.557604	19	5.889846	88	13.10918	26.40313
8	46	46844115	1.520368	20	5.501228	87	12.29054	26.48586
23	45	4.09E+08	0.817994	11	28.35853	341	7.175671	34.23103
24	54	3724106	1.158225	15	3.479589	84	9.318549	38.44367
17	40	15105492	0.999539	12	5.282375	61	7.301608	28.79266
6	35	5027879	1.219843	14	3.733777	65	9.042606	20.97686
25	57	55651987	1.260767	16	3.750472	91	9.998089	40.99702
8	34	8172342	1.171992	14	5.691038	57	8.318669	20.48091
28	62	10236724	1.263288	18	2.841281	81	10.36486	45.0139
19	49	1.73E+08	1.151324	16	2.955139	113	9.241458	34.36346
15	44	22386791	1.149442	15	2.964833	72	9.06844	29.32514
6	34	6545738	1.062251	14	3.334505	64	8.561933	19.47199
16	49	58525392	1.348939	15	10.55674	104	11.19397	32.3092
20	47	4216263	1.07027	13	6.835212	66	9.081881	34.02722
9	43	5836308	1.530642	17	13.81208	200	12.16331	26.32998
4	43	663192	1.51039	20	3.81042	60	12.11925	24.11429
-5	34	4318897	1.557609	15	7.281083	53	12.29662	17.97626
13	45	42852742	1.292564	16	6.678246	73	10.78214	29.36166
18	49	78228610	1.268704	15	7.751262	84	10.25506	33.53904
18	56	8939773	1.464865	20	3.769295	93	12.04597	37.19207
3	38	1351230	1.355739	15	5.378645	56	10.83221	23.494
3	41	6343316	1.397753	16	6.734566	55	12.26444	24.30519
12	42	20933765	1.37449	15	15.68685	73	11.17478	25.23274
27	50	51981765	0.904412	10	22.34103	69	7.570121	38.75535
-1	46	36958980	1.74273	21	7.294402	78	15.38654	23.83215
16	38	12539245	1.124589	10	11.81102	66	7.830441	26.72879
-18	43	2192075	1.93096	23	7.539472	71	19.91519	18.55556
-28	32	7129811	1.853739	23	5.374358	64	18.24278	7.908581
-41	33	968101	2.16273	47	2.11291	61	24.48524	1.723422
26	52	18452984	1.14288	13	16.07468	72	9.51497	38.54779
-7	48	12076622	1.837877	27	4.065995	84	17.45909	24.1908
-5	49	8533628	1.923749	25	6.500665	96	17.74274	23.37649
26	50	87211634	0.925183	13	7.354457	73	7.795433	37.84841
16	41	8687762	1.080379	11	14.64733	55	9.295913	27.90406
19	43	80337078	0.917164	12	15.70269	63	7.551045	31.26585
3	35	6662073	1.35185	16	5.395173	60	10.31959	19.29347
-7	42	7126624	1.791722	21	6.433055	109	15.65251	21.15264
-5	34	376844	1.562014	22	3.547266	58	12.81547	16.10512
8	50	16965141	1.564158	22	3.845025	142	13.03438	29.00638
-1	45	16521502	1.726655	22	5.937803	77	14.86246	23.04498
19	48	2689850	1.180251	15	4.781118	71	9.217853	33.62815
13.4	38	79012145	1.090309	14	5.302486	67	7.906546	25.72727
-7	47	9568296	1.799068	22	6.688427	79	17.07816	24.35589

14	51	60495436	1.445344	16	8.411302	82	12.53627	34.18212
12	52	19553876	1.490929	20	3.417687	82	12.54716	33.38135
-19	53	10927282	2.089187	29	4.553792	86	21.36514	24.31501
11	40	8947194	1.199714	13	10.21206	56	9.731172	26.45064
14	58	58348675	1.661472	22	5.710586	104	14.0121	36.22066
-22	47	6318275	2.030285	32	4.027169	85	21.18555	20.00417
16	48	36050990	1.266895	15	8.314677	72	10.91373	32.7099
-16	34	8259776	1.816378	23	5.619211	65	16.19417	10.88229
17	56	1.25E+09	1.686683	20	216.9199	1375	14.85427	38.16661
-3	52	28010740	1.731363	20	6.866193	79	17.0919	30.09716
4	57	10794685	1.927579	25	7.213726	118	17.87946	32.29513
38	71	1.05E+09	1.289918	15	13.88663	155	11.40777	55.0278
14	47	64570133	1.325147	15	10.67787	84	11.73299	30.53163
4	39	54043206	1.473042	17	7.676326	75	11.57762	21.47932
18	47	1524681	1.15243	15	5.353974	79	9.409596	32.45685
9.7	41	12011518	1.340199	15	8.730487	81	10.79054	26.36354
-3	36	50825691	1.594037	15	8.271683	69	13.31909	17.92094
7	49	37129653	1.644146	21	7.252974	89	14.05288	28.49153
2	45	5199482	1.481257	17	7.755896	66	14.12768	26.54968
4	40	6743481	1.489931	15	8.904658	65	13.7254	22.55717
13	47	46565585	1.385699	18	5.093973	75	11.02803	29.71327
14.7	43	852078	1.144999	15.25	3.792811	67	9.189291	29.41981
19	47	1.01E+08	1.142805	15	5.334274	83	9.081755	32.93573
16	44	34406400	1.159666	14	8.946431	91	9.46196	30.29857
-51	41	655500	2.302222	60	2.004831	66	30.85994	3.410646
9	42	20301844	1.428851	16	8.622158	102	11.39056	25.99159
15	38	61713500	1.190136	12	39.97065	175	7.865502	25.29172
7	39	15971303	1.310543	16	4.240843	69	10.17186	22.65648
18	58	32098070	1.578976	20	7.107092	91	13.01919	38.51403
-22	36	1071874	1.887834	29	3.380568	56	18.46827	11.99244
-4	49	16749547	1.934729	24	7.367695	93	18.30955	23.34311
-7	36	12238563	1.556515	13	7.751748	57	13.55827	19.98704
6	39	7817634	1.501053	16	12.84974	70	12.41022	21.82115
-29	31	8923634	1.765583	20	5.682931	58	17.50323	9.308123
-46	43	452252	1.660213	58.5	2.438781	54	30.18968	14.4
25	44	12195717	0.632503	10	4.302823	65	6.062976	34.04933
23.8	44	1229863	0.662838	10	3.002977	57	6.252119	33.66278
20	43	42080368	0.892497	11	3.977865	65	7.131724	31.35685
23	45	1.65E+08	0.761909	12	4.163436	74	6.890598	33.86044
4	28	5441149	1.004285	12	5.947459	53	7.69648	15.79461
16	40	42821699	1.08545	11	6.720759	102	7.626678	27.96951
17	41	10317450	1.067647	13	5.506836	68	7.870316	28.78601
12	41	5890861	1.246038	14	5.895825	69	9.374267	27.11679
17	43	9054932	0.983116	13	7.75719	60	8.707544	31.15363
16	45	28039271	1.18596	15	6.459006	70	9.506003	30.38184
6	51	1.71E+08	1.690927	23	4.18345	109	14.26828	28.24032
25	50	96034108	0.959757	13	6.790878	75	8.024294	37.29794
-9	34	3478365	1.642429	18	6.725502	61	13.69558	16.06857
17	40	7434162	1.005383	12	4.359483	57	7.200488	28.78873
36	70	9625440	1.341395	17	4.371925	98	10.8872	53.97153
12	37	78686842	1.116456	13	3.694435	68	7.846277	24.46599
17	49	2369086	1.287055	16	5.142888	74	10.35439	33.3337
20	54	1.11E+08	1.409959	17	12.08733	89	11.4636	36.52272
30	50	2043704	0.617135	10	3.036005	60	6.052279	39.63376
22	52	11359361	1.21682	16	10.63092	84	9.809178	36.98674
-53	52	21630495	2.395572	53	3.374123	84	33.50668	15.19406

12	46	87230093	1.546063	17	62.85033	205	14.90078	27.7623
9	40	2.15E+08	1.427661	14	14.97782	144	11.93562	23.78815
25	52	3.12E+08	1.062963	14	8.796668	112	8.643516	37.92341
22	46	2.23E+08	1.036969	13	369.5031	1062	10.17925	36.17631
27	51	63062015	0.986813	12	14.82513	194	7.982171	39.44737
0	33	5909798	1.37891	16	14.28041	62	10.79392	16.80557
20	43	13231461	0.879231	11	8.103853	60	7.375983	31.61953
20	39	34934175	0.860668	9	4.017763	90	5.903067	29.61258
23	46	6560323	0.80085	12	3.01061	65	7.168961	34.29061
-13	40	5706879	1.658977	20	4.912847	61	15.91174	20.12097
11	46	6811670	1.278341	15	6.644867	68	11.39979	30.56866
31	56	49096190	1.196529	13	14.4254	78	8.356661	43.50496
17	48	1893733	1.237462	13	13.59827	59	12.35648	32.32639
18	48	8850146	1.095473	15	3.921021	76	9.065772	33.77518
29	47	53791534	0.560335	10	8.603568	68	6.000455	37.81569
22	46	2809156	1.00809	11	9.876012	107	7.954125	34.21223
26	45.9	13894020	0.620349	10	12.7945	61	6.360878	35.77211
17	43	1.11E+08	1.083864	15	6.982337	75	8.585877	30.01035
12	45	64664355	1.351069	15	8.438599	84	10.92123	28.973
21	45	38082084	0.951251	12	13.66068	68	8.490906	32.69454
16	46	33029553	1.299836	15	9.804659	81	10.48322	30.78225
13.0126	50.02008	1.28E+08	1.464244	18.91903	5.52013	105.0237	11.63536	30.55053
23	48	69156854	0.971152	12	6.249407	78	8.333928	36.16393
19.7	57.3	353375	1.353503	18.75	2.966738	77	11.25987	39.74242
11	50	10628348	1.484433	19	6.106209	81	12.57438	32.11319
27.04038	50.02008	92728204	0.94417	11.99744	10.15677	75.03012	7.815226	38.36214
16	45	1.1E+08	1.221745	14	19.71251	147	11.05955	30.23837
-73	52	69808961	2.318088	45	3.46258	83	37.57304	13.92549
2	46	1722050	1.566875	19	7.206144	76	14.43758	27.12718
-83	42	2308098	2.136057	27	4.015745	65	33.8707	7.358156
16	45	33007402	1.145034	12	14.13101	64	13.16213	28.86991
62	121	42949578	2.145096	29	10.76109	171	20.609	90.66184

PX24	PX25	PX26	PX27	PX28	PX29	PX30	PX31	PX32
22	-69	131	5.554752	23.92168	-0.95775	11901809	0.484406	120.4105
22	-23	83	6.082835	24.30784	0.166478	17256842	0.510002	103.9319
47	-31	136	8.051525	48.92667	-0.11978	1.7E+08	0.426163	201.4419
36	-1	56	4.098644	36.07723	-0.34018	2397429	0.847525	53.14549
36	-52	128	7.055691	36.06922	-1.19034	14688135	0.550846	210.3218
16	-61	118	5.415922	18.97506	-0.75984	13990643	0.602135	130.1342
19	-99	181	7.841884	23.77873	-1.02587	13406434	0.435021	242.4981
16	-83	150	6.864733	22.05541	-1.78489	17534055	0.485086	285.4576
27	-59	113	6.777517	28.1564	-1.81741	1812272	0.429329	303.6486
29	-91	221	8.074824	32.179	-1.05389	97584876	0.424315	284.1733
30	-219	304	6.952406	32.21811	-1.87082	4.38E+08	0.491323	225.0682
48	-44	227	6.748001	49.26537	0.194131	1.93E+09	0.451849	171.0254
30	-91	173	5.905196	31.51855	-1.78449	67614493	0.532947	184.0118
24	-52	121	7.315948	26.86572	-0.43831	6433875	0.447491	184.0432
33	-43	101	5.103287	33.5702	-1.68844	3924172	0.635971	138.5679
34	-1006	1107	5.610192	44.42003	-28.7515	4.87E+08	0.632511	927.9615
16	-48	100	6.547573	19.4708	-0.94968	2972664	0.512688	170.8812
25	-86	154	6.776537	27.71247	-1.45971	77672496	0.436625	222.6299
33	-89	155	5.130251	33.99145	-1.67487	62384921	0.65027	109.1129
31	-131	209	7.943137	32.63934	-1.03464	3.18E+08	0.474057	208.9922
29	-58	138	6.53559	31.11085	-0.11179	3.75E+08	0.498648	128.1287
27	-84	161	8.229489	30.08033	-0.79253	98490618	0.428044	234.2859
34	-36	107	5.279284	35.09553	-0.67996	5957962	0.66793	103.9633
30	-61	133	5.439966	31.63868	-0.67011	1.51E+08	0.559703	107.4857
40	-103	182	5.439106	40.63583	-2.91417	1.42E+08	0.618969	174.5752
33	-50	181	5.508328	34.70869	-1.09627	13364353	0.61424	193.5162
44	-36	231	4.378905	44.46399	1.655185	72981560	0.640062	85.08959
30	-34	102	6.169224	31.79865	-0.15411	42568877	0.537196	112.6394
31	-68	135	6.941656	32.8181	-1.76708	23823322	0.50755	274.8712
28	-73	146	8.02981	30.83924	-1.3547	42236340	0.435376	291.4492
29	-62	125	5.822325	30.09245	-1.69369	6148885	0.511145	178.6216
32	-90	187	6.823677	33.4458	-1.22671	1.45E+08	0.518255	184.6469
25	-1024	1087	6.085248	85.10398	-12.2672	86462819	0.475663	6894.011
29	-85	197	4.971239	30.43385	-1.93425	98051497	0.548509	135.6334
30	-51	281	5.60329	31.38634	0.056034	9.44E+08	0.555391	101.4674
35	-104	176	6.582267	37.29559	-2.39004	19348378	0.55341	219.4364
32	-93	320	5.825555	33.01625	-0.59556	3.43E+08	0.595006	110.0415
13	-60	111	5.311735	17.08932	-1.01596	3178058	0.656888	120.8843
37	-19	88	5.16715	37.66777	-0.21647	21353847	0.717767	84.2607
17.5	-81	131	9.416151	23.61327	-1.42333	2037362	0.375401	402.6855
32	-134	207	6.809006	34.71499	-2.92695	58528661	0.511394	345.2894
39	-72	244	8.455158	40.20199	-0.42753	7.66E+08	0.433713	220.7537
23	-34	96	6.83217	25.81297	-0.25314	15078142	0.461691	148.243
32	-73	196	5.752989	33.90709	-0.80589	1.53E+08	0.581317	135.7775
35	-43	114	5.993441	36.35684	-0.75103	1.52E+08	0.617599	124.041
25	-14	72	5.056304	26.36635	-0.18191	6711623	0.488849	88.09925
30	-77	142	5.845291	31.44489	-1.66467	12836014	0.545854	161.5392
29	-40	101	6.882885	29.95602	-0.48586	56155052	0.5116	134.9121
31	-40	117	7.270877	33.15888	-0.18764	54769300	0.482595	168.9663
33	-42	109	5.761913	33.8377	-0.47922	1.12E+08	0.614779	105.5472
39	-79	156	6.121324	39.98716	-1.14258	2.09E+08	0.587275	145.7052
26	-54	120	6.31319	28.08366	-1.07672	6277522	0.440036	193.2733
21	-55	115	6.339592	23.26529	-0.97179	12532018	0.473017	156.1058
33	-38	100	5.18824	33.72692	-0.96856	31295708	0.664367	100.8985
41	-84	571	7.301726	43.10992	2.085755	3.87E+08	0.466519	319.4666
32	-46	108	4.761929	32.69899	-0.38038	48542537	0.655761	76.89075
31	-78	142	5.634821	31.6205	-1.79493	36306100	0.560676	169.7059

23	-77	137	6.813033	25.80332	-1.74343	9753924	0.423368	257.8895
30	-17	89	5.396927	31.96521	0.118011	11390184	0.561368	98.04027
27	-107	283	7.490119	31.69237	-1.72244	25985083	0.422215	320.6486
54	-63	301	11.62409	60.33258	0.504725	3.18E+09	0.318812	443.3816
31	-154	221	7.214891	32.96278	-1.96649	11738476	0.488702	219.4954
33	-102	166	5.463741	34.38637	-2.76333	1.08E+08	0.639903	182.6794
31	-89	212	6.145999	32.60474	-0.80205	2.13E+08	0.543556	139.9787
35	-78	298	7.385355	37.05526	-0.24613	3.46E+08	0.507867	163.2065
33	-34	106	5.994102	34.48351	-0.75431	15090382	0.603731	124.3746
32	-13	73	5.698694	32.7256	-0.45668	8869523	0.596209	106.3257
30	-18	76	5.875664	30.78774	-0.45145	6959657	0.543925	112.9666
21	-55	126	7.381749	24.05743	-0.06755	94341793	0.463346	164.9026
25	-80	268	6.086903	27.69754	-0.02464	6.01E+08	0.476796	123.0909
28	-95	170	6.837944	29.88832	-0.27831	2.23E+08	0.491759	131.0366
35	-88	176	6.362434	36.50914	-0.90112	6.26E+08	0.569783	155.909
37	-53	384	6.242858	38.60451	0.430751	3.8E+08	0.607963	118.8055
37	-24	86	5.590185	37.21379	-1.22864	4186328	0.649895	141.2625
26	-43	103	4.227409	27.57175	-0.67577	23238184	0.513341	67.58598
36	-108	183	4.927876	37.18178	-0.89167	3.36E+08	0.730871	83.65028
24	-44	113	7.188185	27.05781	-0.48128	22238029	0.442924	178.1815
29	-76	459	6.118928	31.4055	1.84595	1.58E+08	0.502747	158.7256
25	-19	92	5.300918	26.96615	-0.0005	33357277	0.486156	94.15839
20	-59	117	5.528494	22.9055	-0.21342	18193197	0.526572	104.3693
29	-101	208	5.474111	29.9212	-1.38665	77819024	0.538241	123.0501
37	-63	164	5.599978	37.54328	-1.35445	13825538	0.644864	161.9036
36	-52	393	7.038048	38.55168	1.661543	3.03E+08	0.521945	180.2179
36	-62	144	6.552738	37.96254	-0.12516	9.48E+08	0.567519	128.0595
27	-79	159	7.905926	30.0301	-0.29219	87944436	0.429942	209.6509
32	-51	115	5.026207	33.24182	-1.63522	28301010	0.646896	129.5226
20	-83	144	5.959259	22.7562	-1.01838	25410417	0.494865	142.0701
22	-101	157	6.687242	25.87491	-2.31978	3873964	0.431517	288.35
35	-115	195	5.723289	35.79529	-1.92437	1.08E+08	0.625836	162.5707
20	-93	167	5.636841	23.35325	-1.62688	1.58E+08	0.528106	134.4424
39	-19	537	4.971301	45.44132	9.192338	1.45E+08	0.68353	427.3826
25	-57	117	5.700067	27.13405	-1.01308	19085673	0.475035	128.318
23	-62	123	4.608934	23.91995	-0.74552	34866046	0.50227	77.6093
32	-74	245	5.675353	33.64017	0.123405	12895898	0.573528	211.344
38	-55	236	4.577093	38.24281	1.1439	1.45E+08	0.771789	84.79177
33	-20	85	5.393028	33.64663	-0.56593	3838108	0.625412	106.3115
20	-27	87	7.827301	23.30369	-0.36384	2767819	0.436964	190.2988
21	-62	129	7.957528	25.14289	-0.51806	10394903	0.42307	232.5599
32	-37	127	6.289957	34.77479	0.11013	45633709	0.529116	152.7563
28	-101	152	4.528348	29.83324	-4.1445	7117646	0.531107	245.5176
35	-57	141	6.734074	36.83652	-0.77992	1.14E+08	0.53312	179.8688
30	-60	136	7.858785	32.12282	-0.85517	70636716	0.46362	220.4556
32	-33	102	6.024073	33.43896	-0.81212	14451618	0.577069	142.5759
32	-42	324	5.244117	34.67116	3.113259	28887313	0.599818	177.0248
44	-36	121	5.316896	44.3558	-1.14509	94062775	0.5526	120.9499
66	-4	107	7.204891	65.63432	-0.49961	30687467	0.49109	166.6922
46	-106	196	7.26006	47.53366	-2.2645	56102257	0.452921	246.4442
32	-83	200	6.981757	33.8256	-0.44595	1.19E+08	0.506725	165.6019
29	-75	150	5.775127	30.37196	-0.73112	2.28E+08	0.524017	119.0039
23	-80	316	5.736381	25.70785	0.605138	1.56E+08	0.475503	140.7045
37	-65	136	5.573642	38.06259	-1.37845	20114429	0.639873	135.2469
11	-61	112	5.203515	15.26035	-0.38988	4939649	0.689748	99.44338
24	-276	387	5.81976	26.05654	-0.86291	3.56E+08	0.481271	118.9006
29	-109	203	9.357293	33.15536	-1.0818	36463729	0.388918	342.7164
31	-76	155	6.30923	32.93052	-0.61971	64808761	0.537094	143.5273

27	-70	154	6.972353	29.39929	-1.44867	11551390	0.448736	252.4543
23	-46	113	6.429982	26.02109	-0.18157	9110684	0.466172	137.6671
31	-128	742	6.644521	35.49149	5.850368	3.81E+08	0.502995	346.5797
34	-45	113	5.542896	34.84648	-0.82085	12234616	0.637382	110.8788
33	-113	212	8.376041	36.56389	-0.99895	68186158	0.427254	277.6974
37	-95	168	4.696957	37.75059	-1.54078	1.84E+08	0.761495	86.12333
29	-23	87	5.713901	30.37389	0.01472	8204763	0.527174	98.35489
36	-65	165	5.825409	36.60226	-1.46347	1.24E+08	0.628686	151.8785
29	-37	101	5.355439	30.33198	-0.28143	9697273	0.53681	99.78241
28	-38	111	5.440503	29.55756	-0.66597	17918842	0.528588	108.0657
27	-59	123	5.858809	28.90659	-0.80836	37878399	0.506007	120.6214
32	-99	181	9.927558	36.02377	-1.45994	43849574	0.372499	523.4613
43	-91	405	7.878639	44.85259	0.79369	1.22E+08	0.412344	306.5572
37	-57	134	4.955549	37.84312	-1.31297	1.15E+08	0.718594	105.8029
35	-73	190	7.367853	36.14883	-0.99682	2.68E+08	0.499072	222.4471
29	-69	157	8.233318	31.9247	-1.28975	26288343	0.422979	322.0613
27	-83	170	8.328112	31.06705	-0.70214	1.36E+08	0.412062	263.661
35	-49	390	4.876983	35.58471	0.831595	8.54E+08	0.707029	94.50839
39	-17	101	6.492413	40.24774	-0.11588	9567187	0.555802	141.965
29	-43	104	5.03401	30.35093	-0.65791	17625045	0.568098	92.16179
21	-39	104	6.27864	23.99719	-0.18413	5221178	0.480941	135.8364
41	-27	118	6.996749	42.96107	-0.23443	67408062	0.50504	164.8981
21	-50	107	5.6668	23.29258	-0.85355	8486531	0.495571	123.0763
45	-10	91	7.794288	46.78394	-0.17353	36607230	0.462822	162.4862
35	-25	138	6.780145	36.23288	-0.00705	1.51E+08	0.54602	131.9745
29	-21	93	6.553529	31.43503	-0.03301	30668428	0.507926	128.1974
19	-32	96	6.092534	22.30663	0.202124	16724413	0.546931	118.4272
34	-107	211	6.686127	36.31764	-1.9107	1.59E+08	0.533703	275.0866
36	-39	105	5.655729	36.32122	-1.45069	15633421	0.62592	161.3794
28	-84	284	7.223827	32.09168	-0.52251	26984314	0.433362	336.6082
25	-43	103	8.385695	28.70263	-0.58984	2783289	0.407674	242.3422
22	-80	133	6.953933	25.23339	-1.89497	13051529	0.394748	313.5776
32	-90	163	6.834572	32.90919	-1.45049	1.47E+08	0.518582	220.9083
35	-74	158	6.681073	36.41334	-1.31355	2.9E+08	0.540349	201.0637
38	-53	146	8.370385	40.22874	-0.27844	22098438	0.435624	235.1012
27	-43	99	6.605819	27.77932	-1.402	4416556	0.457169	219.7223
28	-68	123	7.48832	29.70462	-1.6699	20856269	0.460004	291.622
27	-94	167	6.535934	31.19705	-2.91901	72115261	0.461571	336.5649
40	-102	171	4.797897	40.42504	-2.95818	1.93E+08	0.692674	132.2069
27	-119	197	9.374144	32.18185	-1.60518	50631459	0.378851	467.7005
28	-64	130	4.653555	29.2312	-2.20141	46494088	0.53753	140.0348
26	-116	187	10.93748	34.30214	-1.96862	9884579	0.352008	832.3285
14	-104	168	10.3994	26.71908	-1.54296	37892567	0.361699	651.3635
10	-76	137	19.29882	28.35614	-0.48609	3589608	0.244314	801.1004
41	-73	145	5.798109	41.32194	-2.92938	63569155	0.593424	221.5706
29	-97	181	11.86837	32.93716	-1.00941	41363636	0.347944	499.6616
27	-111	207	10.82306	34.13676	-1.49949	25524350	0.335164	618.8585
38	-73	146	5.347499	39.23484	-0.88856	3.13E+08	0.670272	106.8706
31	-77	132	5.06521	31.6899	-2.89338	37226377	0.601044	225.613
32	-101	164	5.22312	32.93965	-1.78721	1.67E+08	0.641433	107.467
21	-71	131	6.842875	23.69577	-0.93614	24702205	0.449594	189.2516
25	-105	214	9.313304	30.58391	-1.27865	30379423	0.364389	487.9409
18	-43	101	9.090779	22.8303	-0.65559	1397294	0.386654	261.8479
29	-48	190	9.273543	33.39971	0.030336	32516709	0.396647	274.1706
26	-99	176	9.468683	30.70844	-1.2467	67896732	0.370987	411.9371
34	-40	111	6.219223	35.76386	-0.46405	7330633	0.554313	148.2012
26	-76	143	5.598467	27.6144	-0.49453	2.71E+08	0.493903	100.6627
30	-104	183	9.99811	34.27876	-1.69995	43145684	0.384577	581.824

38	-85	167	7.119729	38.78483	-1.91172	2.47E+08	0.501868	335.8458
35	-41	123	8.775477	36.95905	-0.54257	58365841	0.432601	251.6567
31	-97	183	13.28243	37.59084	-1.25474	60800976	0.305671	821.8516
29	-96	152	5.560891	30.12805	-2.15882	33175173	0.535465	208.0629
38	-98	202	9.444762	40.70941	-0.90015	2.16E+08	0.387999	345.3199
27	-108	193	14.08547	33.8291	-1.13535	41996560	0.315176	744.2411
35	-71	143	6.492988	36.26306	-1.80549	90981115	0.561416	245.0719
14	-106	171	10.04197	24.67415	-1.28374	33231679	0.356153	490.3895
37	-106	1481	8.247511	49.38769	10.05394	4.51E+09	0.428524	982.4538
36	-99	178	9.171572	39.01484	-1.8772	1.04E+08	0.425101	616.3181
37	-100	218	10.58332	41.19803	-1.61389	40919910	0.345881	654.3024
58	-104	259	6.706576	57.68896	-2.55183	2.39E+09	0.547991	299.9575
34	-95	179	6.533557	35.3869	-2.28178	1.89E+08	0.551659	320.0524
23	-88	163	7.225703	27.04973	-1.57114	2.4E+08	0.42067	270.3268
33	-28	107	6.456786	34.74464	-0.62674	5653343	0.564649	153.743
29	-74	155	6.272371	30.68369	-1.86047	47300598	0.488574	246.453
22	-97	166	6.885761	27.07071	-2.09041	1.92E+08	0.399674	411.663
31	-97	186	8.769855	34.58817	-1.49803	1.75E+08	0.409535	384.5744
32	-86	152	7.867907	33.62392	-1.97799	16994739	0.487237	425.6822
28	-104	169	6.805426	30.98709	-2.28284	25004057	0.449873	451.3736
30	-68	143	7.531869	33.02393	-0.69199	85352950	0.457138	207.7019
29	-19	86	6.282539	31.69872	-0.29372	3159408	0.522792	139.2837
33	-66	149	6.211458	34.99224	-0.55924	3.2E+08	0.561065	139.6943
32	-83	174	5.898933	33.16442	-1.71254	1.28E+08	0.572785	181.8749
17	-80	146	25.03083	35.30153	-0.54919	3630775	0.237418	1234.565
28	-82	184	6.850172	30.79424	-1.63212	79517000	0.45875	272.7226
26	-178	353	5.02376	28.15624	-3.52732	1.61E+08	0.483035	153.103
23	-63	132	7.057282	26.16677	-0.4453	47377605	0.450547	171.3836
40	-82	173	8.527752	42.39938	-1.26406	1.19E+08	0.414033	314.3772
19	-77	133	12.81094	25.98863	-0.95163	2349485	0.309329	531.5904
28	-121	214	10.6377	35.3575	-1.73137	70571421	0.338776	705.252
26	-90	147	6.851529	28.37269	-2.11131	28759064	0.414973	405.5279
25	-112	182	6.793076	29.46919	-2.63369	29926880	0.427322	392.2704
16	-100	158	9.3558	26.71019	-1.69889	27917844	0.387023	626.793
32	-72	126	23.27319	38.19525	-1.02916	1373271	0.430926	1251.517
34	-22	87	4.220975	34.9346	-0.22338	22643659	0.779112	61.06882
34	9	48	4.418443	34.5717	-0.10169	2585610	0.754798	62.01942
32	-47	112	5.072653	32.65876	-0.33205	66110951	0.637503	83.34253
34	-24	98	4.888887	35.00101	-0.48005	3.08E+08	0.720806	78.54108
17	-53	106	5.204622	18.86866	-0.94238	13079086	0.615398	106.5566
29	-45	147	5.144719	29.8525	-0.96619	93309826	0.542535	108.8783
29	-41	109	5.338929	30.61206	-0.78266	25080656	0.550311	108.4636
28	-61	130	6.175825	29.89151	-0.99196	18612199	0.490326	158.182
33	-41	101	5.378543	33.50738	-1.69547	20556703	0.642621	152.1957
32	-74	144	6.224578	33.0377	-1.1953	70470938	0.550222	168.4334
29	-104	213	9.815336	33.72573	-0.48111	5.26E+08	0.369552	339.9094
38	-49	124	5.516177	38.78401	-0.72752	67523982	0.656313	113.0627
20	-89	150	7.880205	25.21933	-1.68221	8838752	0.387101	377.8159
29	-30	87	5.081454	30.24481	-0.41707	23952219	0.560839	85.9577
55	-21	119	7.379823	55.80342	-0.67804	22105972	0.475305	201.0953
24	-76	144	5.552923	26.42425	-0.09061	2.92E+08	0.484809	99.65598
35	-48	122	6.987714	35.94105	-0.79036	8784300	0.50779	180.6237
38	-115	204	7.381117	40.02234	-1.89947	2.5E+08	0.486764	267.8789
40	9	51	4.561709	40.33798	-0.09336	4518864	0.780625	56.3181
38	-90	174	6.575443	39.40937	-1.5523	36038291	0.559857	185.0795
33	-119	203	23.21633	43.93081	-1.17763	80203404	0.26019	1699.056

33	-656	861	7.10376	39.07382	-4.77652	3.88E+08	0.49469	756.0182
27	-125	269	6.264505	30.88626	-2.93341	5.02E+08	0.464018	388.0854
38	-110	222	5.983125	39.61409	-0.63917	6.14E+08	0.610462	131.0907
35	-81	1143	5.290758	51.11143	17.29667	5.21E+08	0.650546	1303.652
40	-55	249	5.266118	41.00469	-0.42138	1.52E+08	0.64643	125.2898
19	-114	176	6.802523	23.04298	-2.15836	15182205	0.468723	248.552
33	-40	100	4.957705	33.21544	-1.3936	26985681	0.670055	103.4708
30	-23	113	4.277228	30.55478	0.069596	83972538	0.6211	56.68988
34	0	65	5.190172	35.45091	-0.16465	9950697	0.701816	80.92072
26	-81	142	9.894234	29.32113	-1.43797	15225514	0.393206	454.8752
34	-51	119	6.488233	34.65137	-1.68474	15915481	0.558401	266.2745
45	-98	176	5.30341	45.13988	-2.09541	50983715	0.512214	144.9275
37	-96	155	5.855705	38.22583	-2.91949	1513014	0.612694	416.2183
35	-23	99	6.374417	35.75945	-0.61154	21153734	0.588301	137.9755
38	-45	113	4.099647	38.64052	-0.97576	93574582	0.823385	63.06276
33	-4	111	5.046286	36.07964	1.60747	5624656	0.639446	131.2636
36	-51	112	4.248579	36.83168	-1.62541	23691927	0.801268	76.92874
31	-79	154	5.923526	32.05251	-0.96943	2.86E+08	0.556376	126.7421
31	-96	180	6.782106	32.90305	-1.63469	73430196	0.502326	243.1766
34	-74	142	5.114882	35.15779	-2.41104	88978868	0.681	167.1373
32	-86	167	6.400961	34.37087	-1.83203	95330524	0.532457	233.8097
29.99359	-98.9327	203.9564	8.140837	34.12044	-0.29734	2.67E+08	0.42636	230.8695
38	-50	128	5.272696	37.92073	-1.195	2.14E+08	0.660052	130.1517
39	0	77	7.619452	42.24597	0.053647	1369156	0.459902	205.2619
35	-80	161	8.084572	36.32003	-1.21778	51615728	0.459574	287.8874
39.03781	-79.9214	154.9515	5.024485	39.87604	-1.55452	2.85E+08	0.669331	118.4453
33	-124	271	6.101448	35.43182	-3.38245	3.06E+08	0.58084	341.0553
34	-144	227	24.44003	49.1532	-1.32357	1.72E+08	0.296541	2222.117
32	-80	156	8.46953	33.95061	-1.75955	5849258	0.454709	416.7603
25	-120	185	19.43283	45.23479	-1.52818	6525380	0.320906	1992.044
34	-111	175	5.319265	37.1099	-3.19003	58181993	0.652089	543.6729
95	-102	273	12.43262	95.60415	-2.01775	1E+08	0.288092	920.583

PX33	PX34	PX35	PX36	PX37	PX38	PX39	PX40	PX41
19.19166	1.866784	-0.12754	0.719633	0.476395	0.198795	0.438981	1.059149	0.274717
5.611171	1.039304	0.277019	0.69854	0.294421	0.402117	0.29015	0.861074	0.204043
19.67763	3.337532	-0.11604	1.061893	0.545749	0.319389	0.486829	1.13299	0.301734
8.81079	0.314854	-0.09603	0.166285	0.125677	0.136736	0.123854	0.543445	0.110159
23.89683	3.569665	-0.66655	0.799654	0.443279	0.270264	0.368509	1.063328	0.300264
17.05414	1.939378	0.021377	0.585221	0.339417	0.254846	0.303677	0.940807	0.242375
27.5698	4.644224	-0.43141	0.990861	0.658347	0.196243	0.544064	1.236212	0.358077
26.23829	9.963668	-1.38936	1.136667	0.612396	0.27595	0.448753	1.189578	0.401186
20.32502	10.04325	-2.13812	1.303366	0.730917	0.272618	0.545135	1.276916	0.418106
32.16575	7.288614	-0.8871	1.185052	0.716827	0.235547	0.558502	1.278567	0.397089
114.6675	11.14469	-1.5237	1.050708	0.425465	0.409546	0.377692	1.041955	0.278129
19.81962	4.331311	0.244678	1.036094	0.446312	0.397261	0.410908	1.044629	0.270012
32.62677	4.336255	-0.92748	0.801293	0.470213	0.24773	0.390666	1.079963	0.310979
19.77468	2.707998	-0.13312	0.898865	0.535746	0.250305	0.476802	1.128902	0.302677
14.53468	2.632259	-0.75566	0.611936	0.36936	0.251062	0.310944	0.973142	0.267533
1838.469	8324.404	-101.702	1.825728	0.321438	0.391754	0.284436	0.916948	0.237903
9.674344	3.109028	-0.26072	0.806226	0.380532	0.347184	0.354463	0.990867	0.251019
30.36354	5.463038	-0.85491	1.056366	0.525555	0.323644	0.434545	1.128232	0.328257
34.27239	2.528004	-0.48383	0.500318	0.297133	0.244218	0.284812	0.885719	0.213777
59.41807	5.212594	-0.66607	1.049267	0.410121	0.435171	0.366344	1.030834	0.272559
21.96927	1.742932	-0.12254	0.801852	0.380063	0.356494	0.364428	0.980041	0.243378
31.1343	5.511742	-0.54048	1.143582	0.575338	0.327356	0.481865	1.173861	0.337638
15.21313	1.175252	-0.23953	0.473825	0.266406	0.278503	0.258923	0.840444	0.197581
22.53994	1.755901	-0.37373	0.694876	0.319153	0.369385	0.302873	0.904921	0.221961
50.08891	8.353853	-0.83781	0.671543	0.323636	0.306869	0.299312	0.922881	0.230447
14.95448	5.531685	-0.35029	0.771639	0.481952	0.23006	0.348121	1.059468	0.350204
17.87183	4.611696	0.843084	0.580965	0.321924	0.289315	0.29641	0.902989	0.230102
13.79092	1.467311	-0.20285	0.748088	0.321684	0.400528	0.315465	0.902013	0.216815
22.28618	9.162981	-1.91492	1.183694	0.504737	0.388379	0.387022	1.105862	0.346139
21.45631	8.216847	-1.41908	1.244392	0.605853	0.328781	0.468654	1.200546	0.37486
21.80511	4.160143	-0.95546	0.830985	0.494078	0.253202	0.428026	1.10797	0.305537
33.3926	4.706658	-0.70517	0.876344	0.417362	0.346639	0.371742	1.028697	0.273627
1807.457	66541.94	-804.461	10.47303	0.451103	0.40158	0.433565	1.035022	0.259812
32.68419	5.073959	-0.87463	0.747821	0.379483	0.312845	0.34549	0.982657	0.253826
22.40335	1.682359	-0.20242	0.657234	0.334809	0.325017	0.326472	0.927218	0.226097
48.62754	9.910924	-1.02946	0.845048	0.37965	0.348402	0.361859	0.98827	0.246273
33.4905	2.603086	-0.38475	0.651715	0.292851	0.378277	0.277227	0.875655	0.214454
16.31764	1.347881	-0.0732	0.432463	0.312629	0.149304	0.279803	0.911907	0.232925
9.038145	0.714041	0.001266	0.355937	0.263236	0.1486	0.251151	0.839234	0.199198
26.29726	13.67562	-2.13104	1.686358	0.987503	0.256611	0.640274	1.401572	0.535173
60.59146	31.55951	-3.76063	1.272705	0.532079	0.369253	0.409215	1.125221	0.356578
25.2987	4.59874	-0.38158	1.228971	0.50389	0.418156	0.455403	1.10427	0.291502
11.98941	1.781078	-0.03977	0.853981	0.41064	0.347795	0.384853	1.012949	0.256308
23.26897	3.083094	-0.37521	0.694702	0.36237	0.307654	0.325859	0.962138	0.24977
15.41339	2.1947	-0.35712	0.619259	0.322307	0.311871	0.307453	0.922948	0.225605
6.355704	0.838749	-0.05659	0.620683	0.454726	0.153112	0.443994	1.0146	0.252099
32.85358	3.786261	-0.83455	0.715876	0.442713	0.222546	0.381419	1.050961	0.289578
13.35015	1.519151	-0.35598	0.781545	0.325262	0.41088	0.308949	0.908817	0.222328
13.98145	2.385768	-0.11579	0.875484	0.515937	0.258946	0.461005	1.117639	0.297753
14.73175	1.202886	-0.28975	0.544744	0.314807	0.264857	0.302546	0.911972	0.221191
36.92139	3.083074	-0.21649	0.685665	0.368775	0.293214	0.336362	0.979713	0.251846
20.53709	3.132461	-0.47137	0.940134	0.59354	0.22105	0.503489	1.178374	0.331509
18.76734	2.673197	-0.20627	0.809626	0.495117	0.234542	0.441645	1.101025	0.294348
14.9357	1.153732	-0.37401	0.45228	0.274341	0.235928	0.254072	0.850623	0.207136
37.78698	87.35291	3.851843	1.468349	0.613415	0.402889	0.423843	1.139597	0.425676
14.6549	0.769361	-0.27751	0.433088	0.278382	0.214873	0.273583	0.85489	0.201517
32.91326	3.744546	-0.92968	0.750176	0.399003	0.289137	0.345323	1.013588	0.273795

29.06485	7.542716	-1.22972	1.146289	0.677853	0.243411	0.521291	1.233702	0.394915
7.579822	1.075585	-0.18644	0.602994	0.376972	0.230495	0.366103	0.970441	0.238974
44.11802	21.20477	-1.9044	1.455949	0.54054	0.44075	0.436666	1.135703	0.339095
34.1868	23.72472	1.688031	2.765422	0.49591	0.696927	0.430418	1.085714	0.294057
76.19386	8.216526	-0.97717	0.988996	0.510462	0.309379	0.4217	1.103592	0.324463
47.02526	8.391827	-1.42013	0.666754	0.353412	0.277996	0.294562	0.950234	0.263646
33.30924	2.575944	-0.42743	0.727836	0.389829	0.299296	0.361335	1.004084	0.256178
35.10323	3.01404	-0.13017	0.917984	0.417681	0.374424	0.387967	1.029727	0.26455
15.01746	1.344902	-0.26076	0.539893	0.368662	0.18398	0.335922	0.987813	0.253528
7.738331	0.896348	-0.25437	0.511102	0.372964	0.156646	0.353749	0.981398	0.244722
7.384087	0.994127	-0.2633	0.567549	0.419185	0.149728	0.400205	1.012367	0.253383
18.81881	2.222595	0.059016	0.897235	0.477136	0.305368	0.439136	1.082298	0.280919
30.66494	1.52879	0.00216	0.754399	0.432447	0.270614	0.414931	1.024486	0.257264
31.43163	1.724276	-0.13184	0.831248	0.324375	0.437748	0.314983	0.91491	0.221346
35.04466	2.872293	-0.36748	0.723104	0.423636	0.256671	0.373416	1.044625	0.280705
25.15326	4.670838	0.164804	0.661954	0.33427	0.328734	0.313175	0.925228	0.233924
8.658462	1.583991	-0.26876	0.517692	0.406249	0.129882	0.326524	0.999997	0.288864
13.07339	0.860169	-0.19162	0.609638	0.3926	0.215158	0.388101	0.96494	0.236782
48.55943	1.290279	-0.15596	0.366237	0.246395	0.19296	0.238136	0.81084	0.188864
12.24756	2.424022	-0.20424	0.907312	0.500123	0.285918	0.455252	1.093975	0.286916
32.45578	20.08622	1.039054	0.891729	0.483812	0.296255	0.409754	1.061526	0.311068
6.591274	1.047691	-0.02986	0.662872	0.440073	0.201361	0.427532	1.015113	0.252764
18.58263	1.024277	0.169674	0.594058	0.410606	0.180041	0.393436	1.011454	0.253316
44.60773	2.395484	-0.56804	0.716208	0.35529	0.329799	0.326449	0.955778	0.243213
24.7646	2.465613	-0.28744	0.526772	0.363078	0.17068	0.305325	0.975574	0.267798
24.78441	14.50022	0.641112	0.864551	0.492165	0.26973	0.432042	1.094435	0.302376
24.83681	2.033059	-0.05097	0.734706	0.358723	0.343674	0.34364	0.960107	0.236888
31.34938	2.968212	-0.15885	0.971267	0.659995	0.190398	0.556726	1.234062	0.347728
23.37451	2.522886	-0.68545	0.554642	0.335889	0.23071	0.292716	0.93414	0.246811
28.232	2.242062	-0.01979	0.792505	0.38927	0.335811	0.359178	1.002842	0.257114
40.23636	12.59182	-1.58845	1.127042	0.586094	0.277283	0.491312	1.161448	0.337603
47.61257	4.502344	-0.7128	0.617036	0.3793	0.218765	0.321646	0.991582	0.272678
28.24174	4.16735	-0.19549	0.736305	0.39266	0.298534	0.357768	0.99572	0.262271
10.10191	491.9175	26.56652	2.15108	0.75871	0.477592	0.319783	0.977537	0.650908
20.50478	2.113791	-0.32447	0.783684	0.436488	0.279028	0.402172	1.030454	0.267467
19.35983	1.080274	0.054186	0.646653	0.401701	0.230794	0.390991	0.967022	0.238428
23.32992	12.28352	0.411873	0.90128	0.531716	0.248814	0.390135	1.114494	0.372142
24.8942	4.087982	0.299875	0.390124	0.198129	0.326977	0.180035	0.693941	0.164782
8.120584	1.044219	-0.28835	0.484418	0.32011	0.196118	0.304743	0.919585	0.224681
10.98768	2.410606	-0.08145	0.940829	0.492961	0.303114	0.446187	1.056859	0.273695
18.70897	4.508394	-0.25823	1.131014	0.554085	0.334596	0.468876	1.136338	0.319189
14.9664	3.400309	0.047595	0.887938	0.360886	0.421138	0.337191	0.956616	0.240032
44.19434	17.91098	-2.50766	0.908372	0.435841	0.284684	0.390942	1.037576	0.277831
24.33305	3.766995	-0.32889	0.874197	0.421946	0.341955	0.370078	1.033295	0.277746
22.03785	4.351594	-0.66801	1.045188	0.504627	0.342447	0.434041	1.121618	0.310241
14.48402	1.881633	-0.42873	0.636553	0.393122	0.229766	0.350545	1.002781	0.262527
14.91737	20.19642	1.777829	0.865736	0.468545	0.293361	0.365005	1.057334	0.331194
18.05123	2.038796	-0.01922	0.675285	0.399525	0.252797	0.3659	1.006037	0.260651
17.20462	2.61744	-0.15398	0.852231	0.517293	0.242529	0.457973	1.110921	0.296976
54.72683	13.43627	-1.59559	1.084257	0.46616	0.381963	0.428679	1.060635	0.274614
33.6228	2.980073	-0.28975	0.872756	0.423014	0.344865	0.388384	1.032702	0.266215
21.90162	1.959897	-0.38107	0.720758	0.378054	0.30861	0.358454	0.969734	0.24262
29.53339	8.505604	0.545604	0.78108	0.472326	0.242428	0.437196	1.062365	0.277111
24.97171	2.730322	-0.33518	0.571775	0.357848	0.225927	0.315981	0.964587	0.254244
15.9643	1.191893	-0.06786	0.421578	0.243998	0.254857	0.231781	0.797587	0.187378
181.1309	1.849945	-0.07284	0.705076	0.448553	0.22074	0.426544	1.039232	0.263926
44.63567	12.49713	-1.16363	1.531033	0.668876	0.380528	0.536091	1.240823	0.369333
33.41715	2.026126	-0.31088	0.706367	0.427094	0.242212	0.390237	1.034253	0.267208

20.84391	5.884171	-1.06744	1.051679	0.712615	0.183367	0.516515	1.271696	0.431613
11.97121	1.57689	-0.00544	0.767617	0.479044	0.229078	0.448215	1.053912	0.268327
60.67498	337.2698	12.86456	1.654545	0.653266	0.428477	0.422369	1.132614	0.46944
15.10131	1.920984	-0.36026	0.559871	0.338399	0.245018	0.307647	0.935421	0.238363
47.01523	9.504277	-0.98624	1.189051	0.740418	0.227346	0.587168	1.29666	0.390306
35.9405	1.672234	-0.18711	0.354862	0.201622	0.266789	0.192985	0.716177	0.162599
7.169161	1.076796	-0.14234	0.659697	0.411617	0.233495	0.397765	1.000451	0.248534
24.40176	3.009726	-0.51829	0.595414	0.357837	0.235186	0.312227	0.969465	0.25785
13.6361	0.925056	-0.21779	0.568079	0.428201	0.138418	0.408774	1.025288	0.257834
13.60005	1.352881	-0.3782	0.666821	0.359896	0.295656	0.347124	0.952198	0.233714
21.51052	1.624526	-0.37964	0.730763	0.36432	0.328866	0.346327	0.955468	0.236781
33.0277	23.92596	-3.23474	1.93998	1.112068	0.244218	0.665992	1.469596	0.639536
38.62404	46.36565	1.876946	1.713718	0.567067	0.501952	0.435403	1.111564	0.364387
24.96543	1.657404	-0.20666	0.414764	0.236889	0.257205	0.216461	0.777861	0.187176
23.82492	5.637375	-1.00874	1.078603	0.484388	0.378098	0.419933	1.110487	0.304602
21.6512	10.72908	-1.58005	1.504013	0.524195	0.470505	0.419027	1.122352	0.3352
31.43665	5.700358	-0.43209	1.156254	0.695111	0.244959	0.56276	1.258151	0.373313
15.33812	3.154467	-0.03612	0.438868	0.2434	0.283402	0.23108	0.801107	0.188922
9.422538	1.498939	0.007567	0.637741	0.500535	0.120084	0.446821	1.117185	0.298288
13.84581	1.181589	-0.3788	0.587447	0.340844	0.262913	0.332243	0.924222	0.224398
11.1967	1.718361	0.138792	0.772428	0.423313	0.286426	0.392987	1.024142	0.261247
17.71377	2.633259	0.045227	0.87281	0.431118	0.337616	0.397023	1.034574	0.265796
11.15916	1.879741	0.015107	0.755448	0.409214	0.290495	0.362616	1.003711	0.265494
11.78731	1.718632	-0.10173	0.839031	0.45947	0.289013	0.427666	1.053571	0.269541
8.510352	2.233823	-0.10208	0.799098	0.34163	0.400636	0.328474	0.92815	0.226663
7.20937	1.570025	-0.11676	0.776236	0.399067	0.322162	0.382747	0.993416	0.247159
10.69787	1.231679	0.3292	0.644411	0.335396	0.313191	0.321527	0.939699	0.230174
47.62158	10.57215	-1.49661	1.02588	0.531838	0.294921	0.406617	1.140259	0.361154
15.41087	2.741922	-0.52938	0.649045	0.406126	0.230437	0.34305	1.024141	0.283731
31.76978	23.74449	-0.41298	1.414812	0.839849	0.248329	0.586214	1.324448	0.486078
12.60732	4.075008	-0.5607	1.041844	0.633537	0.237417	0.55124	1.194368	0.325567
28.21776	11.06062	-1.95322	1.377807	0.843016	0.219795	0.588663	1.313323	0.466381
33.02078	4.495027	-0.91794	0.883694	0.539556	0.225568	0.416406	1.140439	0.354919
24.07792	5.011189	-0.74861	0.841561	0.505453	0.239884	0.424713	1.128197	0.320843
25.45193	3.733155	-0.19305	1.024515	0.727859	0.169261	0.592107	1.276434	0.371661
12.64962	4.921779	-1.19356	1.057501	0.717294	0.190357	0.542116	1.263586	0.39756
20.85932	8.471373	-1.80663	1.218982	0.682874	0.265954	0.476333	1.225754	0.428424
30.93036	26.97671	-3.74897	1.451758	0.728444	0.301817	0.470983	1.176856	0.489914
49.83503	5.070669	-0.62578	0.515296	0.277372	0.283827	0.245841	0.845663	0.215126
43.29388	28.18287	-3.78456	1.967233	0.770547	0.411862	0.550853	1.301525	0.450238
21.63906	3.781691	-0.95856	0.763185	0.468415	0.232842	0.412998	1.074245	0.292034
40.9422	77.88568	-10.4418	3.060786	1.477366	0.312483	0.78411	1.601889	0.823621
35.37273	36.41927	-5.6475	2.499158	1.31115	0.283743	0.732726	1.537653	0.7291
22.00125	22.03697	-1.39929	2.828641	2.482208	0.058471	1.170157	1.747403	0.860193
26.11758	12.18638	-1.75741	0.96095	0.512962	0.288303	0.375173	1.06211	0.362149
31.66209	15.73587	-2.10583	1.955342	1.017316	0.293441	0.64786	1.397741	0.534903
43.01164	31.65846	-4.06113	2.170101	1.335037	0.220158	0.790651	1.585455	0.693293
25.5911	1.739962	0.044436	0.520256	0.243812	0.357628	0.2357	0.803385	0.186821
33.21833	11.977	-2.39645	0.894352	0.470935	0.2772	0.327857	1.019932	0.355346
46.46794	2.487602	-0.55263	0.512414	0.290464	0.267278	0.274852	0.878409	0.212869
18.71333	2.852969	-0.28857	0.848006	0.581769	0.178805	0.501544	1.168943	0.323809
42.0355	24.79354	-2.9148	1.914972	0.953602	0.313848	0.642562	1.413585	0.519574
10.058	3.296764	-0.44471	0.916843	1.006788	-0.05964	0.745174	1.319504	0.396138
13.7143	4.952015	-0.00377	1.176891	0.834037	0.169294	0.644116	1.346414	0.41256
30.56582	13.76363	-1.9331	1.560972	1.026672	0.193226	0.686717	1.453939	0.540532
15.2607	1.878423	-0.22646	0.653708	0.52881	0.10518	0.451097	1.152796	0.322059
30.87418	1.287379	-0.17845	0.756743	0.320147	0.403979	0.313292	0.902816	0.217936
44.03369	33.77813	-4.96859	2.067894	1.044273	0.293354	0.646215	1.465649	0.607162

35.51194	15.11438	-2.45871	1.277083	0.648819	0.305254	0.46842	1.23299	0.420216
15.40796	4.909044	-0.37897	1.222612	0.543281	0.378646	0.461316	1.1256	0.31188
32.17704	46.66494	-6.38569	3.045693	1.661165	0.273564	0.880394	1.698704	0.841748
32.21689	8.624179	-1.94879	1.005821	0.447649	0.376365	0.368685	1.064528	0.305051
36.75047	9.913079	-1.01715	1.404064	0.763531	0.28429	0.602675	1.306977	0.393575
42.11198	29.89793	-4.27875	2.575852	1.612474	0.20068	0.855801	1.679738	0.822921
23.98184	9.195612	-1.6205	0.999268	0.435899	0.372439	0.350206	1.040139	0.304619
36.43229	21.91517	-2.95994	1.908907	0.935868	0.32182	0.648405	1.414135	0.5011
50.99271	5552.124	115.5855	4.945617	1.616003	0.508157	0.643965	1.431061	1.19009
34.68097	39.52758	-5.98286	2.144574	0.916758	0.361019	0.572324	1.388187	0.572075
35.53304	52.22126	-6.15843	2.719719	1.170449	0.390726	0.729003	1.503981	0.611914
60.93571	22.03227	-3.37845	1.247618	0.408295	0.486652	0.316925	0.990725	0.302443
33.87776	18.0229	-2.95519	1.26177	0.577769	0.338665	0.397788	1.135215	0.406385
29.51676	7.833509	-1.18334	1.148385	0.63929	0.270772	0.49952	1.221216	0.383314
14.20247	2.109697	-0.43889	0.662894	0.494979	0.145836	0.415947	1.098241	0.317776
21.49125	7.042606	-1.43926	0.979073	0.600433	0.219719	0.478782	1.187937	0.362032
28.36381	20.54497	-3.33804	1.599673	0.864272	0.261445	0.58033	1.328812	0.509312
33.01549	14.84302	-2.16565	1.418766	0.892903	0.211601	0.625572	1.391777	0.492646
33.52217	14.72301	-2.52401	1.060511	0.347858	0.309088	0.320764	0.963302	0.24334
42.34942	30.87503	-5.38581	1.939707	0.883026	0.353967	0.569111	1.334703	0.525967
22.51692	4.999473	-0.30591	1.18302	0.409869	0.483148	0.360139	1.013058	0.271312
7.630174	1.086674	-0.19548	0.569264	0.475447	0.08231	0.432644	1.085585	0.284428
23.63889	2.358662	-0.30322	0.686458	0.398469	0.261124	0.369975	1.01295	0.258184
33.64016	4.476426	-0.95679	0.737307	0.45542	0.220328	0.373987	1.072108	0.310385
23.44746	45.8978	-3.45668	4.352076	3.450234	0.113244	1.39683	1.905818	1.143239
31.61633	9.070198	-1.42681	1.114865	0.686412	0.226059	0.520022	1.252069	0.406503
91.03372	10.72991	-1.23869	0.879844	0.458414	0.30655	0.395021	1.021608	0.295333
19.4703	2.266375	-0.06892	0.849698	0.508981	0.245042	0.456754	1.11007	0.295193
37.75305	9.336305	-1.13404	1.185824	0.736465	0.214624	0.586924	1.292212	0.38643
26.4567	14.75002	-2.06681	2.006235	1.222213	0.216489	0.750727	1.499508	0.600291
42.95835	46.98947	-5.93907	2.392141	1.465249	0.219471	0.81498	1.622795	0.786556
30.10378	19.0895	-3.70722	1.668738	0.761929	0.355148	0.525879	1.294257	0.467991
41.70103	24.03327	-3.63814	1.485363	0.831629	0.265844	0.55558	1.30514	0.510852
25.36324	31.74449	-4.90028	2.252896	1.053105	0.329221	0.595511	1.426154	0.670237
17.80065	50.14616	-7.86328	4.112385	3.201834	0.114109	1.087544	1.573548	1.686499
8.575002	0.497452	-0.16011	0.257113	0.17635	0.178869	0.173642	0.671036	0.145649
3.524619	0.482919	-0.19004	0.315799	0.245087	0.130908	0.244087	0.794804	0.182965
14.55179	0.954062	-0.30243	0.499303	0.285823	0.27207	0.280291	0.862049	0.204024
8.43518	0.85481	-0.2572	0.374327	0.225384	0.245957	0.218762	0.7701	0.17624
17.2432	1.28367	0.088568	0.516918	0.34881	0.190024	0.320247	0.959583	0.243779
13.68238	1.598798	-0.42741	0.653876	0.386062	0.25313	0.358019	0.993005	0.252713
13.86148	1.261983	-0.34789	0.579715	0.419877	0.156587	0.392294	1.029687	0.262766
21.73163	2.254662	-0.47525	0.754215	0.514928	0.184387	0.454988	1.123244	0.303137
14.75612	2.716448	-0.76252	0.554703	0.366906	0.177561	0.293425	0.952258	0.274539
22.96085	2.49138	-0.53988	0.688284	0.465336	0.186985	0.403438	1.092533	0.299261
44.84285	8.376599	-0.60838	1.491357	0.861896	0.264319	0.652414	1.367742	0.430696
16.28436	1.997557	-0.0812	0.538981	0.282716	0.303602	0.263296	0.849632	0.206914
27.38401	13.46938	-2.12114	1.542264	0.817105	0.29532	0.580177	1.330402	0.460774
13.74308	0.849534	-0.24049	0.556787	0.39538	0.170319	0.380865	0.994135	0.247018
14.11796	2.835974	-0.46203	0.831005	0.572748	0.177146	0.478658	1.177592	0.334666
30.24165	1.034145	0.005819	0.679099	0.421979	0.232707	0.412373	1.002903	0.248805
15.46757	2.541247	-0.22439	0.797967	0.464761	0.256787	0.417406	1.085075	0.287186
49.41467	13.1267	-1.62402	1.050566	0.61853	0.247603	0.505551	1.219721	0.359003
4.40682	0.358631	0.127522	0.258246	0.239386	0.04955	0.231937	0.780652	0.180806
36.41463	4.208981	-0.4546	0.720129	0.465951	0.197378	0.407777	1.094865	0.296775
41.68231	183.1292	-20.9888	6.555882	2.242059	0.446763	0.95205	1.759358	1.17134

821.8634	583.6809	-26.752	3.527793	0.630205	0.686472	0.362836	1.09582	0.494775
43.31565	39.75621	-5.39883	1.606361	0.532822	0.456906	0.418473	1.124051	0.350026
49.78562	2.859383	-0.02885	0.654418	0.312461	0.348449	0.296366	0.90909	0.222318
36.57063	9394.666	181.1777	4.833219	1.930894	0.364808	0.378176	1.021299	1.782799
26.01735	3.672709	0.107424	0.575385	0.320702	0.278215	0.295499	0.916364	0.231343
39.06043	9.655213	-0.99484	0.988508	0.487658	0.311858	0.438771	1.093705	0.288696
14.75392	1.535073	-0.47863	0.471515	0.295149	0.225464	0.267277	0.879419	0.220679
7.624614	0.590862	-0.24827	0.445178	0.332694	0.144975	0.330651	0.917404	0.221284
3.662669	0.779624	-0.17488	0.394531	0.254678	0.21431	0.247924	0.820621	0.191489
30.23415	14.22222	-2.85384	1.638348	0.927436	0.25465	0.601249	1.409041	0.541003
23.1288	7.617824	-1.66754	1.037818	0.562986	0.274804	0.40214	1.148659	0.390244
39.5134	3.333062	-0.24491	0.735785	0.419061	0.258805	0.390909	1.011487	0.257097
34.41979	31.11653	-4.98752	1.492968	0.770666	0.312033	0.389272	1.125146	0.600432
8.561656	1.583352	-0.22436	0.581054	0.367125	0.212952	0.337257	0.980552	0.249741
16.16787	0.605296	-0.01444	0.216452	0.153841	0.164265	0.147349	0.613462	0.131742
8.680262	3.521791	0.448988	0.613239	0.361503	0.245555	0.307857	0.965271	0.263439
24.88409	1.024538	-0.15039	0.240485	0.140937	0.22316	0.135917	0.581129	0.122009
32.9949	1.877177	-0.44384	0.680463	0.350393	0.31812	0.326323	0.959185	0.24178
33.11299	7.666452	-1.35542	1.022248	0.550779	0.281179	0.434482	1.149837	0.351581
23.91714	4.849837	-1.01925	0.570043	0.363932	0.198177	0.283517	0.942611	0.280982
33.80885	7.167078	-1.24224	0.906311	0.58313	0.199191	0.445989	1.180563	0.379371
33.16524	5.701961	0.061772	1.202632	0.511197	0.399856	0.457112	1.110397	0.297268
15.88743	2.005049	-0.33169	0.529068	0.292965	0.277293	0.266486	0.882773	0.220151
4.624425	2.887316	0.052013	0.957623	0.513323	0.30166	0.438447	1.112639	0.309443
34.61782	6.102954	-0.93215	1.022452	0.695876	0.170309	0.539141	1.28111	0.396668
36.67426	2.444351	-0.26925	0.493136	0.320019	0.203048	0.292635	0.923141	0.232557
46.06997	33.37738	-4.45439	1.252667	0.526538	0.367575	0.365857	1.078818	0.386438
54.93878	492.1978	-53.109	10.90235	1.666535	0.722511	0.791489	1.638254	0.964985
33.06401	21.93691	-3.6738	1.517592	0.596588	0.406612	0.450359	1.206374	0.386724
37.51425	433.9674	-48.6244	9.380849	1.929312	0.643933	0.845712	1.6626	1.110899
46.16749	72.40395	-9.961	1.967359	0.568758	0.501356	0.311097	0.996796	0.462911
85.9243	102.5696	-8.67268	3.467831	1.434027	0.392097	0.744412	1.545354	0.826997

PX42	PX43	PX44	PX45	PX46	PX47	PX48	PX49	PX50
0.786328	0.784238	0.987309	0.937936	-0.0633	0.254801	0.410087	4.373878	0.256347
0.855637	0.855352	0.982757	0.942112	-0.14663	0.451755	0.286413	2.347251	0.335351
0.766229	0.762474	0.989227	0.939959	-0.074	0.378029	0.436917	4.421368	0.202457
0.938377	0.938255	0.992639	0.97529	-0.04654	0.18475	0.122259	2.966586	0.756115
0.827678	0.823216	0.991328	0.954957	-0.096	0.347009	0.308039	4.87929	0.387234
0.853884	0.851729	0.99099	0.957244	-0.09331	0.317504	0.274564	4.122213	0.433053
0.745704	0.739292	0.990069	0.940784	-0.03687	0.247702	0.455723	5.24277	0.211193
0.79784	0.791612	0.988379	0.946	-0.0935	0.358769	0.350691	5.109511	0.296339
0.754344	0.745894	0.981318	0.925244	-0.08805	0.379083	0.420029	4.492412	0.230317
0.744251	0.736397	0.993034	0.950384	-0.0561	0.293925	0.445919	5.661128	0.206035
0.818422	0.815869	0.997511	0.973244	-0.11989	0.462646	0.342016	10.70098	0.295561
0.800108	0.798065	0.995606	0.962908	-0.11652	0.46667	0.382979	4.435328	0.234415
0.816096	0.81252	0.992931	0.95743	-0.07844	0.312468	0.337614	5.704725	0.332996
0.770951	0.767493	0.985795	0.932915	-0.05561	0.306732	0.429687	4.436644	0.22059
0.853574	0.85037	0.986352	0.949518	-0.08131	0.30531	0.266349	3.804471	0.455572
0.863352	0.86146	0.999848	0.993964	-0.09061	0.337202	0.257542	42.87298	0.467504
0.827053	0.825375	0.98558	0.941539	-0.10397	0.405937	0.332221	3.093202	0.34656
0.795959	0.791715	0.989791	0.946878	-0.10232	0.410225	0.372406	5.498236	0.233589
0.859538	0.858818	0.994091	0.964566	-0.06511	0.27087	0.275002	5.849918	0.464934
0.82358	0.821173	0.995972	0.96702	-0.13573	0.520731	0.332951	7.69794	0.272614
0.820311	0.819347	0.992438	0.954661	-0.0992	0.421844	0.351494	4.675876	0.279207
0.772993	0.768266	0.99133	0.947455	-0.07905	0.401321	0.414649	5.567066	0.207244
0.871786	0.871287	0.989813	0.957024	-0.07953	0.325095	0.252375	3.893748	0.493304
0.85106	0.850189	0.991457	0.957013	-0.12778	0.432244	0.290659	4.737722	0.362882
0.854235	0.852777	0.996077	0.970287	-0.09549	0.329193	0.279567	7.071178	0.44099
0.844622	0.839005	0.992886	0.962713	-0.11139	0.367126	0.263002	3.857687	0.438997
0.854978	0.854115	0.996848	0.973228	-0.08568	0.325718	0.282574	4.21983	0.435599
0.84329	0.84289	0.987679	0.94757	-0.13139	0.460878	0.310157	3.699206	0.320346
0.823535	0.81818	0.987039	0.946688	-0.13425	0.470132	0.30776	4.702789	0.33232
0.785916	0.779291	0.984392	0.935371	-0.10423	0.412972	0.372664	4.614747	0.242254
0.796054	0.792545	0.986991	0.939986	-0.06164	0.311215	0.378798	4.660559	0.301821
0.82095	0.81863	0.993666	0.959182	-0.10138	0.405539	0.338789	5.76869	0.317138
0.786141	0.784971	0.999767	0.990374	-0.05144	0.281031	0.418219	42.48462	0.24387
0.832412	0.830636	0.995411	0.96575	-0.10131	0.3628	0.320422	5.708941	0.351139
0.83797	0.837545	0.998034	0.976718	-0.08504	0.378072	0.32054	4.724695	0.340536
0.822017	0.82085	0.994184	0.959991	-0.08972	0.386583	0.346471	6.964883	0.349145
0.863468	0.862829	0.998519	0.981579	-0.11968	0.440333	0.267664	5.779341	0.400803
0.865234	0.863356	0.991712	0.960597	-0.04754	0.205867	0.254072	4.035798	0.510051
0.876439	0.875633	0.984729	0.950173	-0.03553	0.176243	0.240577	3.00248	0.542088
0.728902	0.714071	0.981473	0.92448	-0.12218	0.432522	0.416245	5.110845	0.174201
0.811742	0.807094	0.993717	0.96011	-0.10555	0.395889	0.336615	7.772083	0.327709
0.780104	0.777131	0.995043	0.958963	-0.11494	0.505232	0.415464	5.011729	0.213101
0.811823	0.810152	0.984418	0.936468	-0.10119	0.412062	0.36275	3.446525	0.249619
0.842567	0.840685	0.994493	0.964184	-0.10538	0.369092	0.299213	4.815171	0.383843
0.848733	0.847759	0.987724	0.94911	-0.08534	0.361494	0.294607	3.916518	0.423233
0.779791	0.779076	0.973441	0.911559	-0.03534	0.195082	0.434604	2.512798	0.251548
0.818388	0.81535	0.99134	0.953135	-0.07674	0.271436	0.337852	5.725823	0.353184
0.848163	0.847157	0.987631	0.948891	-0.15032	0.472544	0.295446	3.638142	0.317327
0.778426	0.774988	0.986296	0.935112	-0.05704	0.312873	0.415069	3.727126	0.243655
0.850756	0.849953	0.987992	0.949867	-0.06773	0.307073	0.291953	3.830698	0.42065
0.836865	0.835046	0.994381	0.962978	-0.0834	0.340742	0.311266	6.069756	0.388781
0.762384	0.757241	0.984392	0.929639	-0.06334	0.315746	0.432866	4.522215	0.218604
0.787703	0.784525	0.986865	0.937844	-0.06487	0.30579	0.398511	4.323029	0.254085
0.876257	0.874991	0.989621	0.958131	-0.079	0.275123	0.237137	3.858888	0.511545
0.806675	0.802187	0.998967	0.983302	-0.12756	0.489227	0.347344	6.129661	0.277013
0.864001	0.863688	0.989332	0.954517	-0.05604	0.244923	0.269453	3.823114	0.470588
0.835681	0.832702	0.992169	0.95756	-0.09328	0.3365	0.304029	5.729336	0.376219

0.761278	0.754774	0.987003	0.936868	-0.07992	0.338463	0.422023	5.380258	0.207711
0.81876	0.818035	0.978017	0.927142	-0.05588	0.27607	0.356594	2.742843	0.333677
0.796194	0.791731	0.996859	0.969282	-0.14193	0.520297	0.369827	6.624865	0.230417
0.795222	0.791303	0.997099	0.969564	-0.28259	0.779968	0.377428	5.798222	0.152351
0.801094	0.797575	0.995047	0.962289	-0.09731	0.399312	0.367805	8.722048	0.273132
0.861311	0.858535	0.994681	0.967894	-0.08163	0.291093	0.254066	6.851752	0.488515
0.823846	0.822174	0.995275	0.964121	-0.07454	0.348789	0.338573	5.764086	0.33408
0.810618	0.808922	0.997552	0.972426	-0.09938	0.449494	0.364827	5.914233	0.286349
0.837391	0.835313	0.986095	0.944785	-0.04565	0.224263	0.308261	3.86969	0.41598
0.826317	0.825047	0.978403	0.929889	-0.03688	0.204035	0.337041	2.775569	0.381252
0.803061	0.801795	0.975677	0.920592	-0.041	0.206793	0.383598	2.710533	0.32643
0.786651	0.784229	0.987268	0.937939	-0.06616	0.36302	0.406943	4.325938	0.236416
0.795354	0.794268	0.997023	0.968177	-0.05737	0.321359	0.40042	5.53032	0.24507
0.844052	0.843447	0.995023	0.965106	-0.14604	0.504336	0.306965	5.595067	0.284868
0.820955	0.818276	0.993573	0.959049	-0.06559	0.311146	0.335885	5.913522	0.359602
0.845891	0.845125	0.998855	0.982657	-0.09075	0.386663	0.30178	5.007128	0.401886
0.849375	0.84471	0.977675	0.937278	-0.107	0.336767	0.262908	2.937785	0.471369
0.806683	0.806399	0.984938	0.935422	-0.05302	0.256298	0.384316	3.608203	0.286951
0.882289	0.881758	0.997003	0.976261	-0.04475	0.219477	0.231094	6.966309	0.553747
0.779552	0.776854	0.981163	0.925171	-0.06907	0.343943	0.418772	3.485067	0.222753
0.803036	0.800921	0.998812	0.98063	-0.0743	0.355514	0.375717	5.68804	0.277893
0.788317	0.787488	0.974337	0.914911	-0.04567	0.258545	0.416628	2.556456	0.25358
0.806055	0.804994	0.988979	0.944097	-0.03589	0.218115	0.379216	4.305425	0.309504
0.841184	0.839643	0.996504	0.970536	-0.11076	0.395468	0.304861	6.672143	0.33972
0.856439	0.853113	0.994507	0.966704	-0.05571	0.232835	0.259736	4.972276	0.490429
0.792132	0.789248	0.998319	0.976161	-0.05547	0.316485	0.391958	4.969035	0.291518
0.830682	0.829688	0.99286	0.957254	-0.09535	0.400984	0.330551	4.974215	0.35287
0.738338	0.731951	0.990001	0.939276	-0.02558	0.231472	0.471094	5.592097	0.194229
0.860286	0.857952	0.99114	0.958928	-0.07682	0.261919	0.260096	4.829046	0.479314
0.825115	0.823417	0.992297	0.95551	-0.0989	0.412722	0.335758	5.303881	0.292945
0.767684	0.763594	0.991194	0.946399	-0.08177	0.360148	0.429986	6.332514	0.22003
0.847851	0.844905	0.995443	0.968342	-0.06728	0.261018	0.279764	6.895867	0.450049
0.825841	0.824509	0.992282	0.955725	-0.07627	0.353524	0.336864	5.306207	0.320255
0.86726	0.864306	0.998593	0.98672	-0.11631	0.402365	0.234638	3.123104	0.514933
0.804094	0.802339	0.988384	0.943134	-0.08555	0.348057	0.377195	4.518626	0.255109
0.806183	0.805572	0.989195	0.94433	-0.07694	0.2678	0.382603	4.393016	0.281527
0.823145	0.818188	0.994911	0.965518	-0.08516	0.332829	0.310812	4.820462	0.396683
0.912181	0.91166	0.998393	0.985103	-0.10831	0.34753	0.170999	4.984597	0.632197
0.85019	0.849165	0.981441	0.939564	-0.05469	0.232264	0.291297	2.842428	0.446311
0.784684	0.781584	0.981415	0.926748	-0.1135	0.369178	0.405434	3.297802	0.234632
0.778521	0.774031	0.98547	0.934478	-0.10601	0.403731	0.405686	4.308666	0.219022
0.835133	0.83376	0.990361	0.952242	-0.1532	0.509619	0.318445	3.851567	0.333382
0.810909	0.808937	0.993391	0.957033	-0.07504	0.290767	0.361494	6.638961	0.323061
0.822954	0.820137	0.991709	0.954439	-0.1117	0.41732	0.330769	4.921371	0.335202
0.794162	0.790033	0.990094	0.946706	-0.0937	0.414281	0.377732	4.680023	0.251391
0.831622	0.828985	0.985246	0.942575	-0.08032	0.269181	0.315191	3.797782	0.383621
0.829859	0.826634	0.997672	0.976058	-0.0777	0.339264	0.31222	3.849411	0.40206
0.822138	0.820392	0.98938	0.948304	-0.0768	0.312522	0.341213	4.240535	0.334534
0.780843	0.776945	0.986264	0.935632	-0.07077	0.298549	0.406612	4.137654	0.264449
0.791607	0.789391	0.994354	0.957467	-0.10421	0.426446	0.398594	7.387227	0.236089
0.81141	0.809268	0.994874	0.961473	-0.09534	0.401898	0.359664	5.788812	0.292435
0.823773	0.822718	0.992496	0.955456	-0.09294	0.355984	0.343716	4.670754	0.308628
0.786508	0.784746	0.997613	0.970994	-0.05143	0.283225	0.412357	5.427356	0.248337
0.848301	0.84614	0.990556	0.955573	-0.06685	0.263955	0.285441	4.991813	0.448133
0.886091	0.885331	0.993459	0.967104	-0.09387	0.284257	0.221608	3.98997	0.548373
0.790117	0.788887	0.998456	0.976366	-0.04255	0.266187	0.409635	13.4561	0.248133
0.752463	0.74516	0.99199	0.947567	-0.09933	0.444566	0.434719	6.664815	0.182665
0.810888	0.808565	0.993483	0.957046	-0.07069	0.281806	0.359261	5.774713	0.323815

0.76972	0.76107	0.986425	0.937995	-0.08613	0.370838	0.387382	4.556207	0.233948
0.780939	0.778975	0.981833	0.926025	-0.06285	0.27453	0.422108	3.449502	0.239846
0.807743	0.803789	0.999342	0.986989	-0.09578	0.416889	0.349126	7.773316	0.303976
0.850959	0.849251	0.987276	0.949433	-0.08145	0.283165	0.28397	3.878901	0.441057
0.73043	0.721625	0.991135	0.942647	-0.04114	0.283032	0.466694	6.848573	0.196608
0.904783	0.904361	0.995995	0.975991	-0.09296	0.286023	0.186905	5.99184	0.613841
0.803426	0.802503	0.976032	0.920909	-0.05428	0.284696	0.385644	2.665917	0.284673
0.850908	0.848434	0.994574	0.965801	-0.06654	0.268014	0.277695	4.933767	0.457399
0.798779	0.797555	0.983695	0.932328	-0.03093	0.186206	0.392448	3.687969	0.308806
0.82856	0.827715	0.98626	0.94245	-0.08576	0.338743	0.33601	3.677392	0.327905
0.829731	0.828636	0.990234	0.950841	-0.10733	0.379475	0.331566	4.628052	0.30476
0.723917	0.709337	0.984118	0.930486	-0.09037	0.362139	0.42442	5.728777	0.180029
0.796621	0.792978	0.998089	0.976153	-0.18311	0.609929	0.375057	6.191703	0.211202
0.89496	0.893809	0.995318	0.973219	-0.10153	0.281621	0.20059	4.992076	0.581309
0.800236	0.796461	0.992651	0.954043	-0.0968	0.445147	0.368507	4.865821	0.294837
0.806107	0.800965	0.989843	0.949018	-0.17364	0.570141	0.344749	4.626646	0.241791
0.738679	0.731685	0.989551	0.93889	-0.04656	0.301683	0.464637	5.596549	0.186364
0.886269	0.885635	0.999055	0.986446	-0.07897	0.313761	0.222214	3.910147	0.542418
0.785448	0.781961	0.981189	0.926802	-0.02413	0.187948	0.400704	3.064023	0.317001
0.835268	0.834739	0.986965	0.944828	-0.07929	0.298502	0.325133	3.712702	0.361679
0.808523	0.806539	0.983965	0.935221	-0.08627	0.347396	0.366809	3.333065	0.276247
0.807075	0.804896	0.988494	0.943887	-0.09625	0.395146	0.368088	4.195593	0.285554
0.82592	0.823352	0.984704	0.940635	-0.12388	0.390245	0.326925	3.32755	0.297586
0.791417	0.789347	0.982588	0.929476	-0.07514	0.345763	0.400318	3.4191	0.237489
0.837942	0.837078	0.990821	0.953309	-0.13513	0.46024	0.317089	2.897578	0.343577
0.811328	0.810259	0.976818	0.923993	-0.08437	0.376487	0.368639	2.667353	0.274392
0.841537	0.840624	0.987212	0.946742	-0.08169	0.36712	0.309492	3.258908	0.356347
0.814393	0.80897	0.994852	0.963936	-0.08468	0.353325	0.325272	6.891829	0.354253
0.83846	0.834783	0.984953	0.944289	-0.07172	0.29616	0.292842	3.91791	0.434621
0.738261	0.729685	0.995206	0.959232	-0.06365	0.325703	0.449491	5.623669	0.214667
0.73807	0.73261	0.976292	0.910084	-0.05823	0.35664	0.479473	3.536238	0.19547
0.740659	0.730626	0.984085	0.929691	-0.10401	0.341388	0.432643	5.299372	0.192262
0.809894	0.804022	0.989604	0.949576	-0.09705	0.355582	0.330945	5.738859	0.324212
0.800006	0.795683	0.990126	0.947995	-0.05892	0.285459	0.364158	4.898335	0.329292
0.725799	0.717491	0.985787	0.927846	-0.02946	0.225577	0.480406	5.037624	0.197158
0.756787	0.74646	0.973988	0.913722	-0.09219	0.351181	0.4016	3.544617	0.248684
0.791497	0.782246	0.982768	0.935406	-0.12887	0.381635	0.338865	4.552448	0.269569
0.792752	0.787655	0.986887	0.944142	-0.11457	0.410421	0.366042	5.545221	0.247503
0.881631	0.880184	0.995798	0.973017	-0.08948	0.311592	0.22456	7.055162	0.543731
0.755336	0.746144	0.990915	0.946799	-0.12347	0.482998	0.411096	6.556887	0.189939
0.801792	0.799029	0.987638	0.941944	-0.06922	0.286538	0.373336	4.643852	0.313705
0.692364	0.672766	0.979345	0.919728	-0.11137	0.472187	0.439949	6.367442	0.165195
0.70847	0.689833	0.981308	0.924457	-0.11107	0.419942	0.418762	5.92233	0.175946
0.591483	0.543842	0.955394	0.870491	-0.19324	0.681693	0.403787	4.681163	0.087674
0.829886	0.825846	0.987149	0.948591	-0.10445	0.342773	0.304044	5.099491	0.386951
0.728238	0.712498	0.985142	0.931882	-0.14276	0.428177	0.409801	5.605622	0.178612
0.675317	0.656109	0.984682	0.925466	-0.05211	0.296419	0.483061	6.542314	0.135727
0.883473	0.88296	0.993446	0.966472	-0.11887	0.40918	0.228865	5.051921	0.506991
0.854886	0.850013	0.99116	0.960828	-0.1098	0.313581	0.247608	5.754272	0.470609
0.865066	0.864135	0.995556	0.969632	-0.07358	0.298253	0.262232	6.812666	0.463553
0.761876	0.757245	0.984657	0.929749	-0.04737	0.237257	0.43814	4.318182	0.229248
0.722712	0.709353	0.990847	0.943826	-0.09314	0.399837	0.44121	6.464791	0.17571
0.669915	0.653574	0.963508	0.881955	-0.13279	0.553522	0.526652	3.17491	0.178909
0.708134	0.696893	0.98747	0.930505	-0.0281	0.215426	0.49145	3.691687	0.164999
0.704147	0.689726	0.984974	0.927234	-0.05396	0.278767	0.469328	5.516456	0.161694
0.787118	0.782223	0.980318	0.926647	-0.02807	0.19708	0.385801	3.902468	0.321846
0.844386	0.844025	0.993619	0.96093	-0.13301	0.465223	0.30823	5.546623	0.288692
0.730101	0.71529	0.987905	0.938734	-0.08486	0.361415	0.412105	6.61622	0.201075

0.791321	0.783525	0.990407	0.949841	-0.08856	0.346364	0.35127	5.945918	0.314706
0.782463	0.777537	0.98569	0.935536	-0.1303	0.45758	0.394689	3.903494	0.229135
0.660175	0.634381	0.976536	0.910058	-0.08271	0.393339	0.453746	5.641423	0.125118
0.827767	0.82352	0.991276	0.954975	-0.10998	0.403026	0.309285	5.663675	0.359286
0.724085	0.7147	0.990854	0.941169	-0.05678	0.336821	0.474685	6.048864	0.173892
0.671126	0.64499	0.981595	0.92076	-0.09241	0.394291	0.430081	6.470386	0.142004
0.83778	0.833433	0.988676	0.951433	-0.12585	0.404701	0.2882	4.882652	0.401687
0.717278	0.704133	0.986195	0.930985	-0.07714	0.381752	0.454719	6.015683	0.164792
0.73893	0.728587	0.999581	0.98983	-0.08037	0.429951	0.426548	7.082375	0.212063
0.75947	0.747159	0.986756	0.939927	-0.1025	0.391689	0.374275	5.862572	0.256399
0.694253	0.677712	0.986456	0.93081	-0.09522	0.453158	0.469193	5.928123	0.144952
0.853682	0.850335	0.997234	0.976093	-0.16372	0.509709	0.263729	7.792604	0.410233
0.82538	0.818711	0.991537	0.957654	-0.12905	0.411859	0.29159	5.805671	0.377225
0.769975	0.763987	0.990467	0.94596	-0.07496	0.360982	0.409235	5.421181	0.21162
0.802943	0.799756	0.987133	0.941877	-0.05092	0.291659	0.366875	3.762794	0.33497
0.778145	0.772657	0.988399	0.941747	-0.06817	0.269332	0.397379	4.625637	0.27688
0.746995	0.737484	0.983872	0.931044	-0.09549	0.348727	0.422635	5.308352	0.200431
0.724946	0.713277	0.986855	0.933283	-0.05052	0.265053	0.451408	5.734371	0.199152
0.844121	0.842327	0.993105	0.96028	-0.11478	0.338161	0.29718	5.773364	0.403612
0.757685	0.746316	0.987152	0.939999	-0.13696	0.4752	0.385963	6.487292	0.246535
0.827464	0.824887	0.99195	0.955648	-0.18882	0.594256	0.323634	4.724786	0.2576
0.790812	0.787958	0.972788	0.914898	-0.03382	0.212705	0.395192	2.757861	0.313343
0.819636	0.81786	0.992101	0.954145	-0.06384	0.307005	0.346212	4.854573	0.352479
0.825051	0.821095	0.99315	0.959313	-0.06907	0.262397	0.316778	5.793901	0.386782
0.560024	0.499795	0.939955	0.850938	-0.20027	0.72328	0.341358	4.818297	0.089933
0.763316	0.7563	0.991865	0.949425	-0.0648	0.292052	0.413729	5.613287	0.245351
0.809237	0.80783	0.998238	0.976949	-0.09194	0.367229	0.368704	9.535636	0.264523
0.779955	0.776844	0.986484	0.935664	-0.05863	0.308452	0.414561	4.402828	0.231102
0.730083	0.721448	0.988914	0.936413	-0.04575	0.269716	0.468783	6.13507	0.196142
0.690839	0.671265	0.977219	0.912282	-0.11879	0.437153	0.451429	5.124183	0.130817
0.671475	0.651952	0.98348	0.923776	-0.04531	0.268323	0.484914	6.536493	0.136047
0.769146	0.760135	0.985663	0.937288	-0.10311	0.387198	0.384138	5.465865	0.242177
0.755513	0.74783	0.988054	0.940985	-0.06851	0.310632	0.420172	6.444959	0.214849
0.760708	0.746437	0.980897	0.931282	-0.11929	0.438066	0.35225	5.006154	0.223837
0.701456	0.657424	0.931286	0.876939	-0.23028	0.615876	0.177653	4.191761	0.282726
0.91363	0.91345	0.989674	0.965362	-0.05426	0.197378	0.171273	2.924861	0.653638
0.878123	0.878056	0.975537	0.939028	-0.04479	0.193997	0.243213	1.872634	0.548769
0.860772	0.860408	0.989051	0.953416	-0.0783	0.307827	0.275494	3.807678	0.442143
0.891713	0.891281	0.986861	0.956467	-0.07095	0.271889	0.213056	2.897919	0.563594
0.844401	0.84273	0.990707	0.954749	-0.0556	0.247026	0.29746	4.147423	0.423641
0.825418	0.82379	0.992354	0.955629	-0.07501	0.310495	0.335771	3.689906	0.336269
0.808274	0.80661	0.984094	0.935262	-0.03647	0.192676	0.36981	3.717722	0.336847
0.782116	0.77848	0.986357	0.936053	-0.0445	0.236968	0.405986	4.655285	0.274606
0.864574	0.860636	0.986602	0.952777	-0.09459	0.256401	0.238195	3.83519	0.506022
0.807997	0.804459	0.987721	0.943442	-0.04359	0.223195	0.354858	4.785917	0.34826
0.706296	0.694618	0.991619	0.942241	-0.04411	0.31577	0.491018	6.684702	0.149732
0.87145	0.870294	0.992448	0.962726	-0.12141	0.336766	0.247609	4.027434	0.484727
0.743694	0.733357	0.98447	0.930572	-0.09433	0.391773	0.425009	5.215588	0.189721
0.811954	0.811019	0.984913	0.936866	-0.03617	0.212639	0.368476	3.701714	0.32859
0.775973	0.77008	0.978771	0.922437	-0.05552	0.256858	0.399915	3.748698	0.271899
0.795396	0.794774	0.991583	0.948586	-0.048	0.279991	0.404142	5.493394	0.251955
0.799106	0.796032	0.982513	0.931554	-0.05904	0.307808	0.37676	3.922165	0.305001
0.764469	0.758388	0.992589	0.950441	-0.04894	0.292518	0.420727	7.021847	0.264372
0.885273	0.884777	0.976405	0.942388	-0.08529	0.289523	0.225419	2.098111	0.591852
0.805544	0.801929	0.992918	0.955331	-0.04876	0.24337	0.359361	6.029155	0.346125
0.674739	0.643749	0.975313	0.915199	-0.18708	0.585594	0.365374	6.370521	0.128523

0.842692	0.837504	0.999527	0.990363	-0.24644	0.704836	0.268159	28.65552	0.33858
0.80609	0.801858	0.995717	0.965816	-0.12981	0.466755	0.351275	6.560934	0.276202
0.854373	0.853419	0.996917	0.973178	-0.10253	0.400966	0.283429	7.04983	0.421667
0.850427	0.848022	0.999303	0.992573	-0.07089	0.316578	0.272717	5.987073	0.469921
0.855792	0.854655	0.99739	0.975528	-0.07553	0.319424	0.278864	5.094465	0.455773
0.788559	0.785502	0.99257	0.951786	-0.08387	0.356184	0.39791	6.239746	0.258799
0.870675	0.869149	0.988915	0.956094	-0.07133	0.261153	0.246026	3.835339	0.500321
0.835015	0.834879	0.98722	0.94494	-0.02999	0.174704	0.328868	2.756171	0.400684
0.877164	0.876713	0.974844	0.938357	-0.05835	0.243903	0.242014	1.904653	0.522297
0.746115	0.731663	0.982558	0.929111	-0.08869	0.32484	0.385523	5.482156	0.227978
0.822694	0.814956	0.985673	0.945277	-0.10513	0.349101	0.289738	4.796842	0.388989
0.808997	0.807361	0.993598	0.956873	-0.08553	0.302317	0.368543	6.279639	0.301663
0.848767	0.840278	0.986618	0.955878	-0.15305	0.427352	0.223397	5.851407	0.483542
0.836251	0.834358	0.986131	0.944495	-0.0645	0.25434	0.31205	2.916779	0.410558
0.927337	0.926974	0.994144	0.975591	-0.05051	0.17575	0.142334	4.018981	0.711953
0.854156	0.851436	0.990512	0.956938	-0.08564	0.315923	0.26901	2.935527	0.467214
0.932868	0.932544	0.996212	0.980672	-0.08956	0.23663	0.131619	4.985867	0.721121
0.840528	0.839234	0.994652	0.964001	-0.08737	0.382587	0.308228	5.736931	0.353875
0.799463	0.794201	0.99177	0.952948	-0.08935	0.327239	0.356706	5.744086	0.307338
0.869356	0.86609	0.990685	0.960818	-0.06398	0.206725	0.233405	4.885221	0.531489
0.795837	0.79036	0.991348	0.951864	-0.05933	0.271494	0.361483	5.80756	0.327378
0.779814	0.776816	0.993826	0.954768	-0.1072	0.481774	0.415615	5.743899	0.210038
0.871062	0.869405	0.992198	0.962398	-0.08584	0.324552	0.244333	3.978469	0.501888
0.79289	0.788264	0.971219	0.914765	-0.10276	0.406929	0.376382	2.124451	0.257823
0.755006	0.746051	0.989553	0.941798	-0.05298	0.249861	0.416229	5.876653	0.254708
0.858018	0.856412	0.995121	0.967784	-0.05699	0.237903	0.270766	6.052341	0.48562
0.836272	0.831885	0.995841	0.970444	-0.10779	0.389566	0.288086	6.774073	0.39423
0.707355	0.685248	0.984726	0.933987	-0.23936	0.753021	0.384012	7.254009	0.156979
0.796319	0.789165	0.991117	0.951509	-0.11908	0.465649	0.347595	5.729821	0.312002
0.699999	0.676673	0.973908	0.916485	-0.23991	0.746062	0.381084	5.970223	0.169252
0.875033	0.868474	0.992046	0.96796	-0.17702	0.479617	0.190191	6.768774	0.545192
0.703867	0.686856	0.990762	0.946071	-0.14048	0.538663	0.43954	9.241996	0.114227

PX51	PX52	PX53	PX54	PX55	PX56	PX57	PX58	PX59
2.22387	0.282982	0.370155	8.747755	1.71749	0.299007	5.40017	980.8249	0.054138
1.892376	0.405635	0.497054	4.694502	1.584792	0.24824	5.365517	1483.058	0.046029
2.67152	0.322827	0.288637	8.842736	2.057488	0.401911	5.612225	2055.492	0.053063
0.856707	0.17632	0.866957	5.933172	0.73083	0.07299	3.337995	173.7642	0.170692
2.13403	0.313787	0.59991	9.758579	1.709227	0.310733	5.266406	203.964	0.042211
1.863195	0.333623	0.634681	8.244427	1.497906	0.231159	5.181204	815.4204	0.044651
2.712445	0.270135	0.360584	10.48554	1.994036	0.412302	5.480961	360.3241	0.048725
2.465228	0.382105	0.500583	10.21902	1.93118	0.437266	5.573378	595.2535	0.040821
2.622989	0.366569	0.376254	8.984824	1.977742	0.508571	5.113204	54.07797	0.061105
2.834512	0.317074	0.360443	11.32226	2.093346	0.47547	5.556731	1769.075	0.047701
2.353882	0.492002	0.486739	21.40195	1.89403	0.369043	5.566924	7572.781	0.044613
2.47565	0.419256	0.340617	8.870655	1.988284	0.370601	5.686789	22427.58	0.049089
2.159846	0.329903	0.524098	11.40945	1.695365	0.317877	5.428024	1285.648	0.041894
2.515464	0.277973	0.304791	8.873289	1.924097	0.358653	5.405153	264.8309	0.058026
1.816581	0.308439	0.652249	7.608943	1.437085	0.245324	4.852656	100.9937	0.0632
1.814884	0.435873	0.667314	85.74596	1.478024	0.536792	5.018652	5745.678	0.052552
2.179782	0.382815	0.555654	6.186403	1.767396	0.296689	5.428179	170.7868	0.041747
2.492921	0.396678	0.341214	10.99647	1.95317	0.39548	5.775222	2027.555	0.044202
1.702482	0.350567	0.658836	11.69984	1.386328	0.199363	5.017543	1068.336	0.045704
2.418067	0.526571	0.453483	15.39588	1.960993	0.364847	5.633857	5125.082	0.045743
2.207002	0.368983	0.438387	9.351752	1.796176	0.295479	5.554588	7866.831	0.046312
2.711417	0.4028	0.326095	11.13413	2.087436	0.42973	5.731819	2074.753	0.045559
1.654933	0.287115	0.685918	7.787496	1.372884	0.185058	4.341747	195.3594	0.08338
1.950753	0.399295	0.552443	9.475443	1.614455	0.253507	5.357416	4724.732	0.047399
1.872592	0.39228	0.644081	14.14236	1.519469	0.248795	5.154309	2120.918	0.045871
2.018316	0.414715	0.64359	7.715373	1.578892	0.313398	5.139115	230.1078	0.051559
1.72958	0.469522	0.625982	8.439661	1.404741	0.225722	5.131969	1021.412	0.045307
2.034352	0.4097	0.493233	7.398411	1.695925	0.267443	5.462344	1248.753	0.049235
2.3463	0.462903	0.541298	9.405579	1.882653	0.422108	5.524967	433.1633	0.043178
2.64315	0.398955	0.416834	9.229493	2.049861	0.462561	5.74009	714.0115	0.041755
2.245818	0.31514	0.480211	9.321119	1.741201	0.331266	5.172755	123.2247	0.05184
2.267202	0.413549	0.515575	11.53738	1.818803	0.323426	5.48772	2829.019	0.043424
2.227345	0.410073	0.314413	84.96924	1.740428	2.731033	5.462469	203.6532	0.052583
2.012512	0.54065	0.537337	11.41788	1.613932	0.281826	5.378473	2850.11	0.044085
1.972123	0.420003	0.525079	9.44939	1.621884	0.248011	5.344941	16957.02	0.046467
2.179929	0.430954	0.561587	13.92977	1.765654	0.306175	5.423584	286.7327	0.045183
1.855304	0.494625	0.598185	11.55868	1.551543	0.236141	5.237747	5203.733	0.050705
1.661671	0.207156	0.701682	8.071596	1.321752	0.186273	4.961696	168.5901	0.045639
1.524863	0.172974	0.725901	6.004959	1.231887	0.154793	4.844584	281.3023	0.048542
3.01635	0.368538	0.313953	10.22169	2.261432	0.668465	5.628862	100.0035	0.057539
2.380812	0.43808	0.536523	15.54417	1.878116	0.451196	5.567609	909.6662	0.040814
2.710101	0.452899	0.39308	10.02346	2.146556	0.433215	5.655581	11424.77	0.049475
2.292908	0.357597	0.338939	6.893051	1.838994	0.316155	5.650914	567.0924	0.046529
2.014968	0.399659	0.584931	9.630342	1.620055	0.264268	5.295154	3428.619	0.045457
1.929305	0.409832	0.630356	7.833035	1.576706	0.235392	5.213863	1894.796	0.043725
2.112374	0.177627	0.298659	5.025595	1.633952	0.268852	5.176184	282.4909	0.064896
2.055731	0.30534	0.543492	11.45165	1.609056	0.289647	5.258555	343.6938	0.042176
2.037616	0.424691	0.479368	7.276285	1.685038	0.276702	5.503009	1742.762	0.048446
2.494928	0.270024	0.40799	7.454252	1.913385	0.347855	5.459127	1099.756	0.050413
1.831894	0.276001	0.620003	7.661396	1.490595	0.214888	5.173159	1907.871	0.044113
2.045898	0.366912	0.5983	12.13951	1.648335	0.26361	5.266603	2825.107	0.043804
2.554289	0.280353	0.327059	9.044431	1.933432	0.383418	5.394253	194.045	0.058272
2.339837	0.308734	0.397757	8.646058	1.808456	0.326186	5.529926	409.5621	0.047447
1.581746	0.271478	0.698412	7.717775	1.290967	0.181655	5.020763	648.4165	0.046608
2.601228	0.468028	0.482004	12.25932	2.065553	0.520441	5.73321	3630.422	0.041938
1.59528	0.259716	0.659103	7.646228	1.303102	0.177867	4.960483	1088.624	0.04625
2.007283	0.342895	0.573044	11.45867	1.601314	0.287295	5.291822	862.847	0.043837

2.642086	0.346769	0.279936	10.76052	2.009647	0.456036	5.669353	275.8183	0.05104
2.000898	0.235337	0.515401	5.485686	1.59814	0.244991	5.29328	228.4433	0.047882
2.675134	0.503572	0.384239	13.24973	2.136845	0.499122	5.87156	667.4473	0.044287
3.232137	0.700772	0.254238	11.59644	2.683803	0.815333	6.341963	24563.07	0.046725
2.429925	0.408637	0.455323	17.4441	1.907724	0.374865	5.579166	279.5792	0.047435
1.757776	0.455881	0.682906	13.7035	1.417256	0.255042	5.024251	1141.319	0.046202
2.137293	0.389901	0.529715	11.52817	1.709773	0.279416	5.402321	3622.884	0.042953
2.383169	0.424982	0.491886	11.82847	1.922507	0.333916	5.554679	2616.217	0.044925
1.920074	0.205528	0.61987	7.739381	1.507221	0.227139	5.117544	235.1613	0.042803
1.877498	0.173661	0.570958	5.551137	1.468555	0.221016	5.111516	118.1267	0.046179
2.00603	0.173	0.497189	5.421065	1.550301	0.246683	5.200056	112.9272	0.046703
2.464282	0.308437	0.382152	8.651876	1.929433	0.343593	5.568441	2974.307	0.046366
2.24823	0.344771	0.315996	11.06064	1.781911	0.296711	5.487934	15492.85	0.05161
2.107873	0.495622	0.409904	11.19013	1.760421	0.288906	5.558877	6407.759	0.047723
2.182202	0.332216	0.571861	11.82704	1.716266	0.286685	5.196907	9338.796	0.04644
1.98568	0.435319	0.612321	10.01426	1.63612	0.249056	5.192121	4802.198	0.046513
1.81977	0.309131	0.670696	5.875569	1.403891	0.230985	4.170987	140.6309	0.102575
1.979062	0.253877	0.413664	7.216406	1.5764	0.25056	5.351549	670.6484	0.051261
1.49201	0.349938	0.73429	13.93262	1.223792	0.153158	4.70778	6002.02	0.05766
2.477417	0.306507	0.290726	6.970134	1.925084	0.351859	5.614005	674.8592	0.048988
2.284331	0.40454	0.44139	11.37608	1.806574	0.343885	5.521591	3158.725	0.046207
2.15101	0.257248	0.335329	5.112911	1.68214	0.275736	5.372254	1063.942	0.054192
2.03576	0.216235	0.475594	8.61085	1.592206	0.251166	5.284872	667.4227	0.044972
1.981265	0.404508	0.518386	13.34429	1.604431	0.267875	5.422643	2049.846	0.046348
1.800445	0.231981	0.688344	9.944552	1.413352	0.222463	4.965284	168.5472	0.046089
2.402189	0.399534	0.499289	9.93807	1.864784	0.339179	5.313562	3796.054	0.045535
2.135206	0.373178	0.564822	9.948429	1.743683	0.273357	5.261026	13571.08	0.046826
2.756766	0.211031	0.298621	11.18419	2.012435	0.407816	5.459041	1151.615	0.052763
1.715483	0.311237	0.6722	9.658091	1.380128	0.222633	5.022667	533.1911	0.044563
2.171164	0.390022	0.46218	10.60776	1.757527	0.295444	5.604261	830.2309	0.042524
2.552761	0.369488	0.330884	12.66503	1.975733	0.428284	5.592313	115.0036	0.051617
1.897231	0.327549	0.653516	13.79173	1.504578	0.249084	5.047149	1669.725	0.046469
2.046529	0.426968	0.494986	10.61241	1.649697	0.282241	5.376305	4296.867	0.046269
1.781707	0.649184	0.706035	6.246209	1.426988	0.727447	4.966122	1712.046	0.050064
2.209141	0.373051	0.342911	9.037251	1.753361	0.305043	5.546255	958.1911	0.050386
2.024926	0.299853	0.397176	8.786033	1.607819	0.262088	5.4122	3632.332	0.049821
2.090305	0.363033	0.599052	9.640925	1.633272	0.358249	5.184209	176.1964	0.041693
1.296654	0.470222	0.789794	9.969194	1.103621	0.147063	4.466395	4430.414	0.078087
1.753379	0.205281	0.641908	5.684856	1.403093	0.201132	4.991476	134.8557	0.04818
2.445063	0.311268	0.367111	6.595603	1.902187	0.358448	5.367237	289.1626	0.058917
2.665372	0.355236	0.358152	8.617332	2.062939	0.421275	5.73314	663.9378	0.046858
2.221579	0.492583	0.536975	7.703134	1.824222	0.312206	5.544533	1606.714	0.044215
2.008602	0.353406	0.492289	13.27792	1.587279	0.336053	5.344343	171.0695	0.047166
2.286491	0.404683	0.547934	9.842741	1.83206	0.324036	5.471791	2908.19	0.042805
2.542062	0.390007	0.429179	9.360046	1.987223	0.387454	5.655513	914.184	0.043869
2.003917	0.259137	0.583133	7.595563	1.574179	0.257419	5.221101	463.5998	0.04414
2.022469	0.473455	0.602151	7.698822	1.593326	0.33357	5.225177	427.4861	0.041564
2.053108	0.336785	0.520614	8.48107	1.639459	0.268702	5.363642	938.163	0.045326
2.44569	0.264024	0.452735	8.275307	1.87104	0.342381	5.401606	259.63	0.048941
2.421421	0.623511	0.32928	14.77445	1.921296	0.387604	5.633135	560.7118	0.049207
2.33873	0.371506	0.486594	11.57762	1.866378	0.323943	5.55935	3924.114	0.043661
2.082041	0.373476	0.474994	9.341507	1.679923	0.274703	5.467817	10761.37	0.045286
2.264785	0.608099	0.347177	10.85471	1.761154	0.313351	5.477859	5028.59	0.050763
1.883825	0.283599	0.65267	9.983626	1.497838	0.232406	5.08109	574.4011	0.044448
1.532217	0.291885	0.730776	7.97994	1.269913	0.166394	4.830091	1166.974	0.055833
2.205334	0.309209	0.323552	26.91221	1.72428	0.288407	5.446575	7184.82	0.051691
3.00651	0.42652	0.332874	13.32963	2.285107	0.549977	5.835341	1211.839	0.047833
2.177487	0.269987	0.51573	11.54943	1.70214	0.283365	5.37658	2460.009	0.042745

2.588409	0.359106	0.38481	9.112414	1.933394	0.441073	5.621611	196.7532	0.047895
2.318522	0.237466	0.312948	6.899003	1.794241	0.311665	5.428829	636.2582	0.055173
2.422989	0.546096	0.501518	15.54663	1.90989	0.576953	5.52503	6362.703	0.04294
1.847562	0.337809	0.641938	7.757801	1.484585	0.224568	5.164625	539.198	0.04416
2.915115	0.395963	0.369015	13.69715	2.10451	0.482367	5.471262	1180.426	0.04905
1.333431	0.370799	0.777001	11.98368	1.122548	0.139121	4.572301	9290.846	0.069681
2.114642	0.237623	0.43564	5.331834	1.671972	0.267829	5.367257	167.6795	0.051929
1.876884	0.294555	0.660833	9.867535	1.499951	0.238313	4.984843	1911.451	0.048212
2.026056	0.188344	0.470564	7.375939	1.562342	0.24907	5.179482	230.4133	0.049916
1.995359	0.306399	0.501262	7.354784	1.611401	0.256679	5.363535	897.4117	0.045436
2.049894	0.350936	0.460173	9.256105	1.661149	0.273771	5.516094	1940.788	0.044459
3.241888	0.347167	0.364043	11.45756	2.359015	0.763012	5.833697	600.7938	0.045677
2.762803	0.64567	0.367185	12.38341	2.231679	0.570196	5.961332	2695.375	0.046296
1.455765	0.309667	0.754786	9.984152	1.210687	0.162913	4.655797	5097.79	0.065714
2.511067	0.431156	0.508948	9.731642	1.9714	0.390748	5.501452	2935.579	0.042868
2.688578	0.527934	0.421723	9.253291	2.176	0.507052	5.892916	880.5506	0.042009
2.870244	0.29622	0.293103	11.1931	2.116664	0.462841	5.573829	2577.095	0.053098
1.529823	0.625727	0.72466	7.820293	1.269196	0.170567	4.667596	21227.16	0.065774
2.268427	0.147293	0.52307	6.128045	1.696687	0.284569	5.024793	117.0957	0.050933
1.861299	0.287878	0.540957	7.425404	1.505194	0.232073	5.259884	768.6553	0.046875
2.23644	0.298578	0.431277	6.66613	1.767203	0.298935	5.493671	414.1579	0.047435
2.352427	0.342349	0.476174	8.391186	1.873472	0.325982	5.543862	1353.888	0.044901
2.117258	0.370602	0.453976	6.655101	1.678125	0.291166	5.536161	717.0349	0.047602
2.34923	0.298409	0.316129	6.8382	1.845112	0.324625	5.477365	231.1193	0.049416
2.141216	0.403457	0.55083	5.795155	1.770562	0.285182	5.409026	6019.253	0.045627
2.218634	0.322916	0.426829	5.334705	1.785439	0.293826	5.504342	1097.201	0.048431
1.968777	0.323995	0.548203	6.517815	1.601029	0.244952	5.372703	561.5242	0.042685
2.353318	0.364801	0.571576	13.78366	1.837108	0.38943	5.34987	1895.233	0.042712
1.967594	0.285164	0.641848	7.83582	1.568299	0.263793	5.099322	149.4049	0.046747
2.824728	0.352887	0.359335	11.24734	2.08158	0.563665	5.480726	291.1059	0.051369
2.72795	0.348864	0.289147	7.072476	2.019586	0.418845	4.971367	61.19876	0.076023
2.759873	0.33188	0.263951	10.59874	2.073519	0.555206	5.535204	403.4788	0.059484
2.300917	0.362702	0.526036	11.47772	1.774454	0.355813	5.45467	1689.5	0.042699
2.334868	0.327685	0.541642	9.79667	1.798496	0.336754	5.277171	2521.397	0.042736
2.848766	0.224839	0.369247	10.07525	2.046446	0.438093	5.334465	289.765	0.052456
2.497701	0.320317	0.41226	7.089234	1.879299	0.443699	5.407361	93.42833	0.053357
2.467759	0.362909	0.447459	9.104895	1.905878	0.475464	5.507843	373.4226	0.051944
2.459924	0.45077	0.383557	11.09044	1.958976	0.545051	5.65705	1066.003	0.049561
1.562218	0.42295	0.725771	14.11032	1.280286	0.198167	4.96936	1615.54	0.050789
3.049823	0.462033	0.34843	13.11377	2.345601	0.684445	5.901259	1626.277	0.045572
2.091795	0.341255	0.485168	9.287705	1.629867	0.3079	5.264551	733.0668	0.049953
3.37043	0.437383	0.316317	12.73488	2.446148	1.134538	5.670113	111.2512	0.059716
3.22888	0.382584	0.350266	11.84466	2.403591	0.952577	5.72855	516.8124	0.051749
3.753994	0.589901	0.137384	9.362326	2.592654	1.327712	5.627822	103.1595	0.085681
2.048225	0.395409	0.590465	10.19898	1.659832	0.368478	5.30199	481.2112	0.044528
3.169252	0.364274	0.347988	11.21124	2.376986	0.743165	5.866105	543.7848	0.048849
3.520508	0.298301	0.25481	13.08463	2.450023	0.876285	5.610021	413.7115	0.056495
1.657525	0.417101	0.699989	10.10384	1.399152	0.191017	4.994784	3105.281	0.054811
1.767362	0.352918	0.663736	11.50854	1.421825	0.341322	5.092166	406.9068	0.047036
1.67589	0.388128	0.655564	13.62533	1.366389	0.20072	5.00994	3617.366	0.048856
2.49661	0.236613	0.357071	8.636363	1.865108	0.357444	5.433366	611.0659	0.051502
3.153954	0.375175	0.304944	12.92958	2.327503	0.717143	5.728832	383.132	0.050286
2.798339	0.465906	0.276383	6.349821	1.856168	0.480908	4.853953	62.3112	0.086184
3.051802	0.28597	0.285527	7.383375	2.149032	0.502732	5.425429	881.8307	0.057985
3.192346	0.280944	0.278376	11.03291	2.259652	0.646911	5.626409	941.0611	0.053714
2.292554	0.206041	0.530087	7.804935	1.68991	0.295629	5.141718	101.7532	0.048385
1.999959	0.444026	0.392498	11.09325	1.669524	0.269222	5.585554	4812.979	0.046451
3.149965	0.360961	0.392897	13.23244	2.299497	0.778042	5.716746	367.5541	0.045137

2.537932	0.37689	0.53259	11.89184	1.941987	0.481475	5.445362	1662.341	0.041335
2.70179	0.404328	0.41734	7.806988	2.107666	0.441473	5.797308	651.2068	0.045491
3.761694	0.349827	0.284799	11.28285	2.64606	1.176715	5.733002	428.003	0.055348
2.107998	0.423895	0.557072	11.32735	1.676174	0.363367	5.331025	450.3736	0.045691
3.067167	0.351093	0.33531	12.09773	2.236126	0.541899	5.624054	1789.894	0.050838
3.568953	0.344658	0.310044	12.94077	2.536101	1.047081	5.665841	301.4691	0.054604
2.162378	0.40399	0.615217	9.765304	1.743366	0.358792	5.317416	1212.398	0.044224
3.248776	0.37055	0.327117	12.03137	2.373189	0.711194	5.786768	639.9696	0.047171
3.139154	0.61602	0.409345	14.16475	2.321104	1.640405	5.641772	22539.85	0.043957
2.9067	0.400501	0.47201	11.72514	2.208289	0.765333	5.672273	796.973	0.043309
3.525925	0.433811	0.299137	11.85625	2.564405	0.972542	5.734122	372.4862	0.058567
2.13192	0.523653	0.620692	15.58521	1.761382	0.413978	5.251403	16032.42	0.050853
2.278066	0.421652	0.592729	11.61134	1.831416	0.459885	5.44551	2167.651	0.042038
2.640835	0.361363	0.276903	10.84236	2.017991	0.446919	5.735415	3403.41	0.046079
2.148646	0.282815	0.526161	7.525588	1.643567	0.289468	4.780857	83.88361	0.066416
2.391874	0.294426	0.447233	9.251274	1.81893	0.394877	5.415778	611.7282	0.047949
2.761876	0.360522	0.282799	10.6167	2.103323	0.615986	5.714296	3510.059	0.050609
3.005557	0.292287	0.375725	11.46874	2.154231	0.577917	5.521403	1418.356	0.0457
2.008585	0.345499	0.607439	11.54673	1.618599	0.352092	5.549887	203.8228	0.044319
2.626755	0.437094	0.411154	12.97458	2.052485	0.705683	5.513406	350.5649	0.049917
2.472812	0.55457	0.430223	9.449572	2.037234	0.398222	5.848383	1846.91	0.043255
2.1113	0.218138	0.49309	5.515722	1.577	0.261178	4.766112	54.07547	0.063768
2.130723	0.335536	0.557725	9.709145	1.688854	0.271232	5.292766	3493.242	0.042367
2.071409	0.299236	0.590841	11.5878	1.615812	0.298182	5.243142	1298.243	0.041501
3.946675	0.590277	0.205033	9.636593	2.847126	1.950578	5.56143	43.05323	0.08185
2.602199	0.321592	0.406013	11.22657	1.952565	0.450319	5.485531	1022.668	0.047768
2.166083	0.4151	0.364139	19.07127	1.742215	0.334564	5.500329	4076.765	0.05237
2.440112	0.30444	0.336965	8.805656	1.874531	0.33967	5.545427	1132.866	0.048567
2.885704	0.334168	0.358093	12.27014	2.082345	0.480572	5.443916	951.6487	0.053299
3.344602	0.366124	0.196744	10.24837	2.399637	0.807112	5.505722	109.8658	0.069229
3.552104	0.287584	0.253308	13.07299	2.459485	0.964347	5.663554	731.2379	0.054578
2.64128	0.37826	0.407015	10.93173	2.031044	0.607667	5.611655	639.7864	0.042083
2.683796	0.326476	0.308429	12.88992	2.031393	0.579248	5.717572	425.2002	0.047234
2.959038	0.417969	0.418714	10.01231	2.299396	0.8265	5.896295	499.3612	0.039923
2.613338	0.546166	0.483705	8.383522	2.023563	1.828555	4.538353	24.16129	0.07794
1.13815	0.199485	0.801491	5.849722	0.952942	0.108366	4.481908	713.3855	0.071389
1.36001	0.175931	0.721829	3.745268	1.11154	0.140221	4.6231	62.05734	0.060308
1.696621	0.286355	0.634188	7.615356	1.395618	0.196282	5.079293	1842.882	0.046711
1.426235	0.271896	0.739119	5.795839	1.18544	0.149928	4.59633	8838.749	0.065774
1.834693	0.24973	0.622539	8.294846	1.456	0.216432	5.119769	654.7189	0.04284
1.997467	0.310334	0.515602	7.379813	1.590373	0.259985	5.337738	2151.7	0.04478
2.005822	0.197779	0.516199	7.435443	1.553013	0.249898	5.209978	474.1526	0.043066
2.310804	0.242568	0.439117	9.310569	1.756014	0.317286	5.33135	305.1623	0.046286
1.655122	0.275717	0.694767	7.67038	1.326005	0.230402	4.885149	387.9788	0.048106
2.162372	0.227779	0.549193	9.571833	1.662076	0.288405	5.129328	1089.571	0.042414
3.232005	0.297987	0.272721	13.3694	2.313136	0.588313	5.604233	8249.921	0.054946
1.73317	0.345773	0.681239	8.054868	1.420977	0.205424	4.988387	3445.521	0.053968
2.89298	0.358312	0.316002	10.43118	2.199007	0.589842	5.699289	275.9841	0.050463
1.955854	0.191856	0.499427	7.403428	1.53231	0.238042	5.225586	385.7247	0.047462
2.467447	0.235155	0.45955	7.497395	1.831091	0.350938	5.34583	140.4196	0.045429
2.140952	0.292678	0.301782	10.98679	1.695385	0.27527	5.47067	5891.518	0.052279
2.350212	0.286095	0.514358	7.844329	1.824209	0.315682	5.266262	93.39695	0.050925
2.598741	0.462347	0.466873	14.04369	1.927857	0.417274	5.245598	3142.812	0.045199
1.235227	0.237354	0.753685	4.196221	0.975727	0.124408	4.092645	108.2389	0.086177
2.23087	0.305877	0.558333	12.05831	1.704074	0.29652	5.248302	326.3046	0.044614
3.962254	0.495913	0.309444	12.74104	2.989993	2.199485	6.164982	592.3235	0.052848

2.568791	0.776443	0.553119	57.31104	2.124007	1.0395	5.718675	2794.013	0.048903
2.422019	0.527824	0.44641	13.12187	1.932843	0.534796	5.708184	9578.427	0.042566
1.946947	0.435659	0.630039	14.09966	1.606014	0.24172	5.177222	9904.397	0.049744
1.884686	0.51171	0.669096	11.97415	1.50869	1.691028	4.936482	4340.489	0.050843
1.805664	0.368755	0.655048	10.18893	1.478438	0.224022	5.064734	1725.864	0.046016
2.429687	0.384329	0.427993	12.47949	1.900906	0.369041	5.557388	528.2207	0.047459
1.602025	0.28171	0.687485	7.670678	1.298005	0.191666	4.994784	564.4252	0.047063
1.680767	0.172364	0.582761	5.512342	1.340871	0.194468	4.98452	1704.807	0.04556
1.536942	0.219369	0.70802	3.809306	1.263103	0.162302	4.96865	252.8667	0.048442
2.819315	0.301902	0.409992	10.96431	2.109141	0.641446	5.508376	281.0542	0.04234
2.213965	0.344956	0.604095	9.593685	1.756063	0.400201	5.336667	237.4238	0.041852
2.127838	0.317725	0.467194	12.55928	1.68218	0.288712	5.442132	1128.734	0.046845
1.984295	0.404978	0.686404	11.70281	1.643095	0.565908	5.010404	78.0463	0.060221
1.961474	0.232424	0.617773	5.833558	1.5539	0.237045	5.236728	279.5956	0.040398
1.019955	0.232227	0.840441	8.037962	0.858545	0.092573	4.198752	3086.055	0.08566
1.797861	0.328288	0.66607	5.871055	1.473768	0.243685	5.073326	107.2243	0.049687
1.000054	0.310094	0.846338	9.971733	0.85638	0.095355	4.383602	822.0752	0.080265
2.002686	0.355005	0.546471	11.47386	1.626862	0.257714	5.361027	4810.836	0.044586
2.406672	0.339667	0.509242	11.48817	1.870798	0.393257	5.412616	2498.749	0.041834
1.649745	0.262518	0.717796	9.770441	1.321071	0.233494	4.779021	1699.938	0.055177
2.347865	0.32453	0.535247	11.61512	1.792958	0.37236	5.280018	1189.271	0.042536
2.688575	0.462215	0.356899	11.4878	2.123206	0.428457	5.773174	5400.451	0.049167
1.728234	0.335789	0.697036	7.956938	1.408543	0.205508	4.947916	2547.986	0.05298
2.512595	0.348462	0.45181	4.248901	1.937015	0.367736	4.032333	25.52525	0.128915
2.688766	0.252378	0.459733	11.75331	1.951028	0.429582	5.395439	361.3567	0.04485
1.728126	0.385329	0.681001	12.10468	1.382838	0.203289	4.895844	2838.565	0.048676
2.151064	0.440147	0.603012	13.54815	1.717529	0.444801	5.290471	4086.802	0.046718
3.874794	0.7441	0.355135	14.50802	3.001914	3.142221	6.255431	1311.49	0.04539
2.556841	0.545269	0.526433	11.45964	1.979263	0.528545	5.482891	66.19143	0.044305
3.566324	0.686354	0.344328	11.94045	2.786878	2.82754	5.704431	67.24468	0.059614
1.803377	0.537969	0.731314	13.53755	1.490363	0.634029	4.906222	1405.368	0.058635
3.76183	0.48203	0.206847	18.48399	2.780013	1.225464	6.238786	252.2415	0.05368

PX60	PX61	PX62	PX63	PX64	PX65	PX66	PX67	PX68
25.9702	8775.99	0.312639	19.32555	213.4698	4019.362	11.87872	0.055932	0.013809
41.53258	16432.26	0.26113	5.973557	333.932	1752.076	72.03033	0.200994	0.009337
27.17764	16508.27	0.415757	19.74923	211.8511	4218.924	11.26824	0.05561	0.019826
20.05933	862.78	0.082935	8.848723	234.9784	2111.057	26.2387	0.11912	0.02442
49.90396	2661.688	0.376259	23.68605	274.7922	6794.403	11.25041	0.046012	0.027157
49.96229	10996.19	0.262786	17.12797	337.3225	5565.826	20.76232	0.062975	0.018137
30.80581	3216.977	0.471866	27.89858	172.1986	4697.414	6.487479	0.039639	0.035916
46.12631	7073.527	0.533465	26.32972	258.0721	6711.228	10.18318	0.043669	0.02484
20.72956	379.9559	0.611582	20.26102	126.0644	2872.957	5.837954	0.061883	0.036434
32.10287	15736.56	0.542806	32.11025	194.7752	6555.262	5.957414	0.034169	0.028473
43.91089	83399.71	0.435438	114.1691	317.3462	37094.37	2.733895	0.008945	0.017402
31.48889	206438.7	0.377036	19.91919	286.9362	5655.66	15.3834	0.054509	0.011374
44.51709	16355.09	0.374997	32.55755	286.9784	9918.358	8.451078	0.032769	0.019944
23.14885	2042.349	0.378925	20.14089	167.7279	3309.583	8.953362	0.054325	0.026298
31.5297	1016.282	0.267282	14.69587	206.3054	3218.87	13.52831	0.077201	0.028208
53.7392	69154.98	1.522723	1834.975	391.1929	721916.8	0.222018	0.000887	0.018161
45.16507	2097.406	0.354227	9.891469	255.1139	2400.287	28.39391	0.125641	0.019119
38.03385	20027.98	0.461611	30.23889	261.973	8223.249	8.659236	0.035971	0.017983
47.7019	15200.05	0.228959	34.23769	348.0816	12360.32	9.869374	0.030462	0.017125
46.6273	53114.31	0.393954	59.47626	327.373	19603.21	5.552013	0.017382	0.021546
36.68842	84703.32	0.300434	22.1818	311.2994	7008.584	14.41934	0.047817	0.013574
35.93177	19493.1	0.458285	31.29589	233.0253	7389.638	7.614093	0.034476	0.023588
22.5196	1564.96	0.204361	15.25267	185.1229	2913.783	11.9604	0.070341	0.026898
40.56281	55791.75	0.264255	22.64455	347.0922	8160.652	15.2072	0.04672	0.010498
48.58383	28619.27	0.332631	49.75623	352.7372	17434.66	7.185769	0.020994	0.015555
45.67432	2741.353	0.342993	14.88125	274.2104	4315.538	17.83607	0.079623	0.024266
41.35024	14429.56	0.222583	17.85074	325.7058	5443.078	19.79887	0.058708	0.012016
35.20012	13624.91	0.263752	14.10058	302.7186	4307.872	22.70525	0.07726	0.012454
46.44906	5091.741	0.500552	22.21511	280.1134	6669.005	12.19026	0.05301	0.024153
43.28461	7444.927	0.546277	21.24292	253.4261	5783.803	11.67128	0.056189	0.024843
29.65278	1214.992	0.378418	21.79386	178.2629	4222.041	7.741374	0.051214	0.030945
43.30433	33763.27	0.364422	33.37346	300.6549	10345.81	8.917033	0.031912	0.017189
26.83088	1842.244	10.90958	1798.018	189.5004	342854.1	0.397786	0.006746	0.018943
41.08782	35461.66	0.325749	32.63356	309.1722	10641.52	9.166915	0.032503	0.012097
39.41271	202677.9	0.248666	22.58396	342.0383	8001.788	15.06977	0.046461	0.013482
38.53128	3511.941	0.41382	47.89474	215.3886	10546.15	4.443146	0.022234	0.02484
43.94921	61063.71	0.245073	33.66946	384.6361	13240.66	11.36981	0.030775	0.01577
56.11965	2426.544	0.229912	16.4843	312.1716	5021.932	19.49603	0.065574	0.030071
46.22769	4159.458	0.161629	9.040897	310.5047	2801.396	34.70896	0.120588	0.027103
24.92326	652.4465	0.730565	25.85731	136.397	3760.182	5.265889	0.046559	0.027389
48.57058	11397.95	0.619953	59.91507	273.3741	17149.48	4.411142	0.018236	0.02564
31.20596	100152.2	0.438911	25.54017	252.5539	6264.034	10.65342	0.042341	0.01689
35.13799	5627.094	0.332492	12.10199	254.2839	3074.01	22.90058	0.093752	0.016928
42.41831	43845.87	0.284229	23.38473	329.4536	7977.673	13.90503	0.045907	0.013149
47.93261	26763.02	0.249353	15.47923	325.7247	5128.335	21.1165	0.070331	0.019147
19.05686	2127.96	0.272968	6.533885	183.1475	1234.826	31.93668	0.185597	0.012938
42.79488	4448.164	0.33609	32.67616	269.0631	9395.628	7.811071	0.032438	0.01729
40.64654	18403.78	0.283744	13.47619	319.1652	4460.705	24.47637	0.0821	0.013955
30.06452	10527.81	0.348947	14.35709	219.4205	3182.809	16.11933	0.078074	0.021602
46.46016	26589.2	0.226813	14.8065	327.4539	5074.366	21.64884	0.072813	0.018765
46.52156	37875.69	0.292866	36.7423	323.8137	11834.88	8.930718	0.028293	0.017838
23.64715	1465.32	0.42716	20.71502	163.8384	3558.673	7.935187	0.053778	0.026441
32.70072	4083.084	0.357835	18.82831	224.9625	4126.022	12.80867	0.058481	0.020953
54.15959	9242.676	0.204289	14.8589	348.3541	5475.454	22.48405	0.072459	0.019589
46.67805	40385.16	0.586472	37.50227	304.7987	11428.03	8.286069	0.02867	0.019257
43.85665	15435.3	0.186767	14.72593	337.5994	5271.815	22.01505	0.072303	0.015417
44.87314	11035.78	0.347503	32.76624	313.7028	10918.36	9.149904	0.032379	0.017142

28.73164	2287.881	0.521526	28.91599	174.6477	5233.267	6.085983	0.03848	0.026593
33.99887	2678.289	0.2489	7.845735	230.8508	1914.227	30.29459	0.149082	0.018902
38.51256	6363.206	0.614839	43.75231	235.8096	10639.56	5.391767	0.025552	0.02047
34.30616	167597.5	0.802449	34.25488	286.7307	9530.709	9.329163	0.03214	0.010376
34.60876	2880.407	0.432518	76.12606	215.3091	16719.15	2.801781	0.013669	0.024654
53.50024	15807.52	0.334465	46.61665	324.2475	15763.52	6.704584	0.022524	0.023282
43.969	45846.77	0.298331	33.31074	306.2303	10586.71	9.008664	0.031377	0.019067
40.73519	29575.62	0.341293	35.2327	288.0765	10096	8.389871	0.029602	0.020768
48.39138	3316.898	0.254081	14.96341	278.1671	4376.287	17.95751	0.073025	0.0263
38.71113	1525.102	0.230841	7.921814	242.8726	2087.126	29.77184	0.147959	0.026122
35.87395	1315.211	0.259337	7.507858	203.9934	1712.398	26.13768	0.160245	0.023184
34.69026	29723.21	0.349405	19.13313	249.7214	4630.994	14.07852	0.056548	0.020663
29.09286	143128.9	0.302213	30.93346	270.2582	8351.444	9.041925	0.033666	0.014828
38.70238	66028.06	0.293515	31.81284	345.165	10895.15	11.29638	0.032787	0.013399
46.87598	114580.5	0.309845	35.05565	326.3646	11628.91	9.245299	0.029749	0.020206
44.05375	62769.11	0.252067	25.23435	351.5918	8825.426	14.18253	0.041389	0.015645
26.04302	891.0058	0.246246	8.707513	203.7236	1823.424	23.30647	0.139507	0.034795
28.95655	6716.038	0.262401	13.24123	237.9616	3335.043	18.16201	0.083095	0.012779
51.04509	76078.54	0.161174	48.64741	406.7581	19880.18	8.337117	0.020896	0.020679
30.81974	6101.717	0.379165	12.35983	219.7625	2783.867	18.95679	0.094637	0.019806
35.67347	34367.82	0.354394	32.62913	269.9166	9032.629	8.271522	0.03211	0.017756
25.48976	9544.705	0.281073	6.706871	220.2524	1563.985	36.22929	0.180851	0.014083
35.93194	7814.851	0.268171	18.90257	240.6546	4203.691	14.16514	0.05624	0.019882
40.49032	23804.79	0.295702	44.63479	324.9053	15032.06	7.139862	0.023136	0.014379
53.58944	2358.267	0.284331	24.56494	299.4796	7485.711	12.02474	0.043834	0.03314
39.83662	43512.5	0.358766	24.9983	265.5266	6611.773	10.85191	0.042462	0.021116
40.81006	164479.5	0.275757	24.98138	340.2361	8482.238	13.88471	0.041971	0.013234
27.08143	9383.904	0.422222	31.50389	181.6852	5844.053	5.832333	0.033698	0.03299
51.25525	7740.105	0.261592	23.32085	342.727	8456.37	14.02168	0.04571	0.018683
43.08269	9661.744	0.330387	28.25656	298.0217	8256.504	11.04348	0.037413	0.018592
28.77067	961.4192	0.56071	40.18627	175.1068	7048.281	4.481664	0.027639	0.029729
53.71498	22487.56	0.304964	47.42586	346.3466	16873.54	7.137985	0.021975	0.02368
41.15549	49043.6	0.31048	28.43712	326.6755	8861.853	12.33384	0.037568	0.015489
53.44882	23374.67	0.722796	10.39386	373.9559	3395.776	41.79916	0.115682	0.022151
29.64795	9033.743	0.324816	20.69212	245.4707	5184.112	12.24544	0.05221	0.013496
29.78707	36619.53	0.270757	19.55139	260.8439	4906.991	14.48731	0.054257	0.009236
48.60897	2423.728	0.400774	23.10625	258.4132	6367.042	10.61438	0.048118	0.027477
45.99944	43789	0.148121	24.98535	461.8116	11484.04	18.66317	0.04105	0.014758
39.37326	1750.529	0.220322	8.180422	235.9639	2068.563	27.81674	0.142221	0.024747
23.9513	2144.62	0.391054	11.08374	181.3341	2013.107	17.86207	0.106761	0.018337
33.53014	5994.483	0.457304	19.11356	212.7355	3965.489	12.04584	0.059071	0.020766
40.93871	19227.53	0.321787	15.02056	302.4053	4727.31	20.24463	0.073752	0.013296
34.17768	1926.326	0.516955	43.95258	215.1384	10075.26	4.702107	0.025467	0.022206
44.87425	36220.68	0.35499	24.32737	307.8633	7682.789	12.60063	0.044219	0.016414
40.94009	9661.383	0.42105	22.12827	259.3921	5927.682	11.83773	0.049951	0.023587
42.2414	6060.956	0.283418	14.56869	281.5199	4355.243	18.7176	0.076277	0.018539
47.92543	6169.129	0.351603	15.01935	281.892	4420.238	18.34613	0.073957	0.022796
37.22599	11437.71	0.284561	18.04546	280.2352	4836.832	16.70168	0.059858	0.015588
31.12302	2605.23	0.357518	17.00792	196.0409	3311.25	12.01136	0.064852	0.020688
31.13108	5161.033	0.48171	54.03405	216.772	12024.12	3.986841	0.019688	0.020653
39.41486	45542.45	0.338642	33.7038	268.6181	9238.575	7.973461	0.031062	0.017002
37.56078	124522.6	0.286149	22.0907	307.3524	7051.301	13.9047	0.04833	0.011834
29.40487	47103.3	0.33951	29.74292	248.7605	7205.124	8.862455	0.035291	0.015869
46.91695	8269.08	0.258877	25.00263	301.505	7547.082	12.12477	0.042728	0.019893
50.11133	14416.43	0.191363	16.15999	363.8305	5831.555	22.87228	0.065471	0.013761
28.69694	66894.79	0.302981	181.541	258.8902	46676.87	1.443697	0.005553	0.015588
32.21182	9853.242	0.632288	44.45214	192.012	8779.334	4.318423	0.024494	0.023984
41.3834	30910.32	0.304424	33.48948	274.4837	9536.817	8.021504	0.031133	0.016548

33.12609	1843.406	0.497286	20.95375	192.3354	4311.733	9.059823	0.055804	0.027818
25.45256	5375.893	0.324412	12.22069	199.8382	2400.176	18.07835	0.092428	0.017746
48.14119	74532.77	0.626915	60.50885	304.39	18846.01	4.966667	0.017217	0.022818
44.94829	7782.429	0.232439	15.14431	292.6665	4600.638	18.99728	0.07221	0.015396
30.26671	10282.29	0.52571	47.07376	167.7327	8039.078	3.555474	0.02254	0.031478
47.84248	101533.2	0.154616	35.90171	436.6693	15711.35	12.16853	0.028508	0.011445
29.98051	1702.246	0.260814	7.445339	200.4977	1557.772	29.19381	0.158748	0.020376
55.45659	24925.52	0.282016	24.24327	360.5904	8978.372	14.57769	0.044136	0.021899
30.2181	2477.914	0.260724	13.81607	207.5186	3092.3	14.56691	0.079113	0.020852
37.1404	10440.14	0.274883	13.61951	271.5414	3989.406	19.471	0.081206	0.013916
39.46754	22088.74	0.295651	21.55444	302.6357	6852.367	13.90628	0.049584	0.011956
36.25785	4899.476	0.917356	32.62077	177.6767	6205.824	5.333165	0.037923	0.035258
35.12439	24007.08	0.594456	38.52792	258.5905	9823.41	7.086904	0.028118	0.014734
51.07935	55744.91	0.18955	24.85449	418.8798	10483.8	16.81009	0.041833	0.013167
47.25868	34175.94	0.41984	23.78154	281.3899	6840.673	11.89857	0.046205	0.025138
43.06664	8866.068	0.5893	21.5651	271.9389	6181.838	12.67494	0.055869	0.017165
26.96103	19999.43	0.507308	31.63927	185.081	5926.581	5.989609	0.034282	0.029416
48.87183	228178.2	0.182957	15.41585	441.7842	6982.933	28.287	0.068714	0.017112
33.20886	1277.788	0.288867	9.665942	168.6442	1545.294	18.84145	0.119034	0.04238
36.443	9315.669	0.243585	13.99476	275.1434	4118.594	19.19388	0.078089	0.012966
33.7754	4199.098	0.320205	11.51598	225.2575	2416.748	22.44387	0.098582	0.016239
37.36222	15228.48	0.338276	17.60899	257.7281	4504.302	15.29316	0.06194	0.017765
37.4551	7464.78	0.310161	11.33778	270.8125	2936.82	26.84255	0.102087	0.013112
31.75564	2164.621	0.341387	11.44965	210.5326	2612.756	18.48513	0.100387	0.025582
39.49908	72033.13	0.2866	8.630143	329.7039	2857.391	41.18798	0.135514	0.010461
32.52515	11507.06	0.28971	7.558155	254.4056	1920.803	38.7156	0.159043	0.016201
46.99616	7194.881	0.25764	11.00836	308.216	3097.448	32.03214	0.099906	0.02131
54.88053	23681.49	0.500753	47.03897	309.8186	15166.45	6.374059	0.022716	0.025956
45.89141	2000.44	0.293657	15.36389	242.3999	3860.196	15.40955	0.073535	0.034448
28.7331	2455.863	0.635763	31.68978	161.7493	5450.266	4.958672	0.035838	0.033345
13.19345	328.1776	0.49079	12.64099	88.44099	1180.629	7.315861	0.099789	0.043012
21.62515	2677.577	0.640205	28.11956	159.6348	4708.018	5.732201	0.04089	0.020206
46.33292	20519.24	0.416802	32.87581	285.4701	9896.284	8.382246	0.032542	0.022346
47.86314	31880.05	0.383763	24.04339	277.4217	6880.762	11.34914	0.045804	0.025979
27.39371	2406.388	0.451972	25.52625	140.0902	3574.634	5.617956	0.042601	0.038124
32.47959	800.5031	0.468137	12.75214	150.9052	2223.859	11.1382	0.101775	0.031029
37.2313	3306.969	0.538471	20.83419	217.6438	5004.838	10.01408	0.058044	0.020508
31.75047	9927.933	0.646158	31.20661	238.0425	7656.167	7.943756	0.040643	0.017696
52.92365	22033.26	0.246582	49.53529	346.0965	17019.76	7.055063	0.020786	0.019079
35.10601	13519.67	0.831444	42.71431	208.8114	9478.438	4.781195	0.026827	0.022209
30.516	7888.258	0.340364	21.84784	212.605	5012.298	9.335705	0.050475	0.019361
24.15639	655.7912	1.414489	40.72732	100.8293	4600.641	2.407851	0.03652	0.061812
29.85906	3612.291	1.114856	35.12686	143.0885	5253.158	4.146906	0.03545	0.038751
11.75341	294.1545	1.407167	22.59468	64.15947	1667.239	3.064681	0.063043	0.053458
41.40902	6413.131	0.4186	25.81901	270.5362	6919.295	10.88698	0.044949	0.017468
34.9584	3873.317	0.863536	30.85618	172.6475	5870.624	5.334753	0.038144	0.028468
23.58619	2454.406	1.072004	42.74614	121.1352	5501.966	2.763862	0.02801	0.049701
49.87753	37973.6	0.211867	25.51248	398.9118	10158	15.80148	0.040804	0.015134
56.42017	5199.627	0.42769	32.77066	314.1134	11149.04	8.968544	0.033636	0.022868
48.39007	47493	0.229092	46.41867	377.194	18158.74	7.886702	0.022133	0.016708
27.94371	5334.434	0.401482	18.81003	186.2283	3430.419	10.57837	0.059404	0.024388
29.54793	2776.282	0.874047	41.43418	161.5279	7262.391	3.721559	0.027511	0.034523
11.02361	279.5505	0.534751	10.64592	65.84094	670.917	7.353183	0.128862	0.053235
22.22082	6032.21	0.517025	14.06792	131.2728	1868.622	9.979876	0.085144	0.037804
26.07501	6499.701	0.741876	30.40519	137.8163	4478.839	4.438009	0.038568	0.042473
35.56887	1165.721	0.306267	15.35093	153.7608	2432.102	9.883432	0.072043	0.050018
38.89137	51175.71	0.27733	31.05035	318.2336	9979.793	10.4844	0.033485	0.012423
39.08544	3131.608	1.005474	43.19563	170.4177	8045.351	3.714603	0.02745	0.04544

50.86757	20183.13	0.593162	35.15146	262.3091	9454.833	7.380486	0.031836	0.02824
36.44917	6192.684	0.473156	15.25225	217.7206	3394.284	14.96134	0.077155	0.020289
28.07629	2363.755	1.384754	31.51325	119.4588	4194.821	3.691985	0.045728	0.050689
43.62303	5278.081	0.423442	32.04738	265.0641	9202.992	7.779922	0.033877	0.02119
28.19774	13660.67	0.628847	36.27627	161.8809	6006.657	4.485668	0.030425	0.032417
30.42965	1740.085	1.260801	41.13748	123.1239	5749.069	2.749172	0.029392	0.054043
49.5468	15391.21	0.438215	23.69513	294.2002	7298.255	12.09198	0.048377	0.018519
33.54737	4831.925	0.868444	36.3081	165.9441	6238.937	4.568114	0.032004	0.037141
41.00568	219736.3	1.646876	51.28701	224.0471	10997.11	4.626965	0.02149	0.03068
46.68061	7822.717	1.049268	33.75807	202.9455	7311.203	5.824282	0.038975	0.036301
23.22235	2199.802	1.129239	34.84041	117.1843	4330.208	3.387615	0.039666	0.039322
56.02478	172766.2	0.526811	60.1745	383.0431	24127.09	6.132112	0.017577	0.015779
52.11529	28445.73	0.564726	33.57082	305.5031	10831.57	8.816983	0.034318	0.019089
34.85632	31071.11	0.533024	29.43094	230.9272	6998.315	7.925999	0.037891	0.024252
19.6182	713.152	0.305882	14.93666	104.9794	1612.443	7.058215	0.075971	0.050789
33.03288	6233.222	0.481325	21.5939	187.9942	4422.021	8.322517	0.05499	0.028553
28.79471	27719.81	0.776057	28.15175	202.5268	5934.443	7.365076	0.044421	0.020528
36.21163	12710.32	0.693893	32.73724	175.6016	6105.548	5.186316	0.035509	0.037581
48.26333	2240.802	0.734	31.99326	205.8289	7211.886	6.032327	0.03747	0.038434
31.29858	3159.456	0.821132	42.30001	182.1619	8420.463	4.109492	0.027106	0.025588
42.09427	19518.89	0.416287	22.59982	298.6056	6767.277	13.90948	0.048574	0.013822
18.47058	443.3278	0.289959	7.614387	100.5354	843.8809	13.0225	0.161147	0.047789
44.12158	46260.33	0.291123	23.75656	285.547	6993.819	11.84399	0.044672	0.020145
50.71554	17917.86	0.354313	33.41663	292.5846	10353.07	8.348882	0.031746	0.025305
17.90276	124.8821	2.088815	23.76616	63.55513	2000.945	2.699921	0.075159	0.097924
32.36004	9821.379	0.5273	31.56098	191.1902	6474.255	5.804812	0.035174	0.029678
28.63129	37601.86	0.381383	91.31523	267.375	24706.04	2.926029	0.011229	0.011553
31.11036	10509.45	0.367433	19.92433	201.7977	3876.534	11.03025	0.054677	0.02319
26.07338	7392.566	0.588703	37.2741	143.6455	5400.798	3.910387	0.029455	0.036621
15.74018	490.9055	0.938821	26.01449	102.816	2986.422	3.885699	0.04766	0.031157
24.99739	4538.914	1.2079	42.78624	127.3094	5769.698	2.94012	0.03018	0.053132
41.38724	6308.839	0.793095	29.50431	224.5308	7554.506	6.917137	0.041417	0.024453
33.1197	3846.748	0.733826	41.67563	199.9605	8740.278	4.718786	0.027672	0.028988
51.40241	4840.884	1.083699	25.00744	233.9591	6217.719	9.398914	0.059601	0.0319
31.45432	133.5871	1.996629	18.46452	149.6323	3511.194	8.913652	0.1461	0.04871
41.3672	7785.662	0.119951	8.663564	337.1363	3016.197	38.04594	0.124486	0.016824
27.75727	776.6871	0.12933	3.727891	203.0816	797.4169	54.65654	0.338543	0.02367
42.63698	25151.4	0.199874	14.68973	347.2139	5362.61	23.06317	0.072677	0.01238
48.16679	96861.96	0.156885	8.513923	440.9344	3909.313	50.77032	0.130184	0.014174
48.52468	9405.133	0.238421	17.24845	326.5301	5375.039	20.07563	0.061776	0.019242
38.9253	26069.37	0.282443	13.77821	298.9105	4445.144	21.09183	0.081177	0.014313
39.27543	6058.919	0.270813	13.91381	243.7161	3717.212	16.52932	0.079747	0.020401
34.24463	3232.722	0.353846	21.6651	206.3824	4842.501	9.070323	0.050317	0.027883
54.61226	5182.736	0.280477	14.60856	336.6315	5321.678	21.64004	0.078828	0.021401
46.96704	14134.65	0.336445	22.87796	280.868	6865.282	11.64862	0.047321	0.025721
24.40327	55486.31	0.627103	44.84533	159.5596	7287.877	3.590125	0.023779	0.031307
47.43829	41901.68	0.223055	16.28841	373.6943	6051.26	23.41804	0.066233	0.010609
30.22171	2117.054	0.711431	27.25965	166.2134	4715.018	6.131894	0.043959	0.027532
33.94916	4557.939	0.241811	13.97108	252.652	3768.597	17.69914	0.077696	0.017998
37.34775	1469.166	0.393224	13.96927	189.7836	2885.455	13.00914	0.083946	0.033215
27.86033	54634.63	0.281897	30.45576	249.637	7570.631	8.510524	0.034112	0.013912
31.83277	931.2868	0.357687	15.22683	163.494	2577.529	10.68769	0.07453	0.037724
41.52685	33846.13	0.503123	49.18813	231.2592	11413.16	4.726449	0.021706	0.026693
23.00713	980.465	0.116016	4.447452	169.4634	692.0525	42.11049	0.251371	0.023192
40.54982	4094.795	0.35732	36.25239	214.2387	7753.975	5.962476	0.029202	0.030823
35.06146	2916.205	2.763456	40.10332	154.8671	7349.272	4.003961	0.046722	0.03067

56.71424	28263.63	1.268281	820.6541	376.5151	312926.9	0.453744	0.001252	0.020581
43.19227	104416.6	0.729946	42.90681	294.7782	13509.3	6.669947	0.027471	0.017217
49.3502	121548.5	0.263289	49.82161	378.2425	18762.42	7.670771	0.02057	0.016179
55.45817	55537.73	2.124679	37.88906	366.5445	13142.36	10.26615	0.029397	0.027157
47.68939	24245.01	0.250578	25.96131	354.0094	8969.541	14.07006	0.040332	0.018515
33.17843	5216.883	0.48354	38.60889	216.4074	8329.79	5.741618	0.028733	0.020603
47.98569	8035.966	0.21521	14.7928	341.7509	5363.206	22.14516	0.07416	0.019051
37.76908	23240.95	0.195637	7.819664	304.9661	2623.178	37.27642	0.145223	0.012262
43.7	3663.479	0.164926	3.79023	291.9858	1143.454	80.10979	0.345865	0.019389
43.02666	2610.103	0.822327	29.41534	200.5258	6871.735	6.044313	0.040607	0.034873
52.61872	3167.807	0.475945	23.0171	274.394	6755.102	11.35718	0.049665	0.027079
33.94984	12341.79	0.344009	39.20095	250.2748	9638.338	6.608005	0.026769	0.01491
43.01991	794.0509	0.695864	34.3287	225.6034	8102.281	6.380968	0.037432	0.027856
51.37232	4071.632	0.270363	8.504551	274.6763	2437.353	31.89958	0.141339	0.027356
47.13912	29664.1	0.1036	16.18364	463.7457	7429.583	28.99765	0.063955	0.015832
41.09429	1379.925	0.267586	8.798424	225.3642	2010.705	25.86765	0.130392	0.025729
50.35578	8206.582	0.126762	24.63806	426.7128	10666.64	17.10448	0.041772	0.013243
45.63532	60032.94	0.278255	33.06677	346.8904	11895.62	10.29973	0.031483	0.017292
44.85517	30003.92	0.46523	32.8633	277.6922	9700.125	8.092256	0.033102	0.018312
59.47828	20980.92	0.297017	23.77305	388.3351	9661.738	15.71745	0.046688	0.026448
48.61788	14886.97	0.445751	33.65721	271.4642	9622.65	7.751015	0.032265	0.027485
31.35724	46831.42	0.461288	33.42179	242.022	8083.227	7.512346	0.031949	0.018446
54.2245	31743.88	0.233795	15.84149	388.0933	6180.523	24.65078	0.068454	0.020859
4.576574	91.06061	0.383736	4.79798	32.48485	150.1919	8.717628	0.322847	0.093911
41.1541	3702.792	0.515412	34.2136	181.6047	6464.209	5.165899	0.031748	0.041418
51.03862	39032.7	0.230975	36.57048	355.3423	12832.09	9.869653	0.028221	0.021255
52.0503	50811.3	0.593386	45.93451	354.253	16983.22	7.52206	0.025255	0.023717
41.01893	8568.259	3.624283	53.67156	181.4776	11202.1	3.742214	0.033375	0.032868
47.70542	679.3347	0.722114	32.07162	199.6185	7012.017	5.800337	0.037201	0.042088
23.78855	361.9823	3.300208	37.26596	112.3954	5013.27	5.278322	0.087006	0.045098
59.839	15629.27	0.887136	45.61649	385.0899	18726.24	8.094542	0.02734	0.020125
26.688	1353.742	1.565381	85.20281	137.3562	12098.03	1.612426	0.013913	0.04016

PX69	PX70	PX71	PX72	PX73	PX74	PX75	PX76	PX77
0.256644	0.001064	4193.262	0.452199	0.371771	19.58289	8.172828	153.4814	0.461374
0.065414	0.002484	5862.678	0.475034	0.308008	6.678791	25.05713	131.0209	5.437618
0.387418	0.001474	7720.369	0.385163	0.520312	19.61765	7.016862	138.8522	0.380107
0.189561	0.005057	355.6536	0.675907	0.202891	8.634197	25.81405	231.6058	2.902275
0.553251	0.002088	821.0792	0.360777	0.657376	22.77713	13.67422	333.889	0.577391
0.303225	0.00159	2879.07	0.403734	0.501902	17.80022	21.18506	348.6661	1.320889
0.891256	0.002543	1599.047	0.371148	0.637131	28.03059	4.980022	136.8633	0.191654
0.549945	0.001991	2454.086	0.351182	0.853803	26.39217	10.19732	263.3385	0.42115
0.574029	0.006346	217.3906	0.376638	0.743274	19.12482	4.84635	105.784	0.254437
0.846	0.001511	7084.443	0.348305	0.741174	31.32068	5.875346	194.7153	0.187452
1.929366	0.000176	25387.43	0.370904	0.694797	111.5037	14.69578	1713.746	0.127259
0.227849	0.0008	79218.87	0.408319	0.490746	20.09299	12.07466	237.13	0.653515
0.582334	0.000997	5526.745	0.406714	0.569568	30.73374	13.14841	451.4896	0.39398
0.519614	0.002078	1102.451	0.417091	0.45975	20.26356	5.452418	108.9102	0.292028
0.380916	0.003391	438.7904	0.493567	0.421663	13.94977	13.10831	203.2311	0.88199
32.80423	5.06E-05	13941.33	0.380787	3.583478	1819.347	27.68664	51073.73	0.017046
0.174435	0.005108	744.538	0.374902	0.565064	10.18964	10.79442	102.2142	1.243682
0.471644	0.001032	8163.194	0.384137	0.628487	29.2334	10.23739	318.1244	0.347328
0.567337	0.000672	3931.065	0.446549	0.439191	32.6706	20.02543	708.8985	0.572439
1.239785	0.000436	16436.47	0.370307	0.630183	58.98366	14.93797	892.1713	0.254916
0.311684	0.000746	29557.57	0.432266	0.400394	21.91726	14.06182	319.3151	0.646322
0.699768	0.00107	8454.24	0.371366	0.622092	30.96085	7.597914	239.7987	0.252878
0.353875	0.003186	725.3469	0.529336	0.339082	14.84726	15.55734	244.86	1.008929
0.225106	0.000656	16440.5	0.44756	0.394029	21.62094	24.9347	591.728	1.081317
0.727505	0.00041	6690.814	0.389264	0.690442	50.08313	22.0541	1086.448	0.453874
0.31238	0.003857	901.5076	0.424555	0.614391	14.08386	17.25714	268.0755	1.175168
0.239425	0.000795	4340.636	0.486863	0.368725	19.17943	19.60701	327.3978	1.195446
0.180514	0.001196	4961.167	0.469739	0.345702	13.96567	17.44089	251.8892	1.285714
0.482541	0.002137	1646.815	0.360249	0.782476	20.87961	13.41109	315.443	0.613692
0.455526	0.002802	2840.499	0.344697	0.775311	20.08329	9.541695	212.4417	0.472707
0.564808	0.003303	594.4516	0.432316	0.501613	20.68372	6.92943	160.5426	0.316109
0.545419	0.000711	10680.12	0.385437	0.586961	32.40322	14.1936	486.8085	0.426128
33.87926	0.000182	975.7722	0.45962	14.58992	1792.769	5.919018	10667.37	0.030624
0.34202	0.000553	11656.09	0.439421	0.473801	30.81497	17.37515	598.1871	0.518511
0.320808	0.000676	61738.45	0.45661	0.351209	21.71781	17.66481	416.369	0.771511
1.101836	0.000819	1435.478	0.418618	0.643024	47.08523	8.832923	428.7903	0.186587
0.544362	0.000535	15099.35	0.445307	0.413983	32.6566	24.0764	833.9095	0.707348
0.467882	0.003257	677.3806	0.423273	0.491616	16.89073	15.43133	249.3174	0.973487
0.243284	0.004638	1220.951	0.492593	0.35123	9.055932	17.14209	154.4999	1.943012
0.593813	0.002031	365.5468	0.331936	0.876786	25.17965	5.060055	132.5592	0.219762
1.346587	0.000657	3702.643	0.355149	1.03254	57.39573	12.12822	753.3433	0.201674
0.398583	0.000967	38974.98	0.361511	0.582247	26.00966	8.689125	216.4323	0.367575
0.19289	0.00248	2450.983	0.427107	0.40977	12.07681	10.55094	126.1623	0.971979
0.306309	0.000731	12426.2	0.42128	0.481177	22.51156	21.89608	531.0948	0.9285
0.30486	0.001607	7231.5	0.41446	0.468019	15.26724	16.90676	265.756	1.109444
0.088198	0.003352	1143.717	0.481051	0.288655	6.446487	5.973568	39.45417	1.077679
0.518532	0.000858	1607.973	0.42612	0.49211	30.8321	11.69559	402.608	0.348703
0.184358	0.001511	6473.908	0.447829	0.374033	12.95805	23.60273	330.8757	1.810503
0.327069	0.00224	4560.566	0.407805	0.466566	14.32157	7.391749	107.463	0.549713
0.281164	0.001842	7645.189	0.443358	0.392393	14.09712	17.50583	270.9495	1.164978
0.604786	0.00065	10095.02	0.389696	0.553187	36.83248	16.86114	613.0605	0.469518
0.487506	0.002438	778.4122	0.399306	0.526625	20.19242	5.557049	118.8251	0.280382
0.341253	0.002057	1816.925	0.416597	0.470263	19.04202	7.619826	139.4415	0.443027
0.280946	0.001976	2470.151	0.459091	0.383647	13.82357	24.37718	380.2834	1.600103
0.766306	0.000848	11921.48	0.32653	1.062262	37.49619	14.01536	521.2475	0.387868
0.212008	0.001495	4378.656	0.484315	0.314439	13.65487	19.75426	306.9792	1.301365
0.478378	0.000923	3321.142	0.410397	0.548225	30.6021	16.62164	574.9256	0.492209

0.701529	0.00167	1208.995	0.390559	0.645938	28.37364	5.641897	165.0895	0.208288
0.155764	0.004041	1140.8	0.473723	0.333024	7.554901	9.725232	79.90242	1.311077
0.769796	0.000818	2687.339	0.353715	0.888006	42.61401	10.10678	452.098	0.239641
0.367515	0.00036	65841.37	0.291662	0.95121	35.01829	15.20361	501.6407	0.498106
1.74642	0.000593	1265.415	0.404789	0.632512	75.07003	9.709166	751.3	0.127669
1.002168	0.000688	4289.515	0.418161	0.637302	44.29308	23.48895	1134.621	0.492574
0.630365	0.000691	14721.83	0.417847	0.462421	32.16182	14.3576	495.9833	0.424386
0.715018	0.000731	9750.877	0.384386	0.518998	35.38142	11.27903	395.574	0.329454
0.366913	0.003117	1089.65	0.428857	0.437551	14.28769	11.82741	184.1885	0.784494
0.20265	0.00689	603.6214	0.476034	0.336238	7.421309	9.127073	76.97958	1.174804
0.158745	0.006818	617.9552	0.469312	0.329271	6.999227	8.307442	67.55976	1.141554
0.383948	0.001553	12327.23	0.404168	0.461592	19.44468	8.54792	158.4844	0.485743
0.475845	0.000557	59760.66	0.450623	0.36051	30.96427	9.625123	297.6909	0.322364
0.4494	0.000471	22398.25	0.453524	0.373543	32.07565	21.43358	683.8761	0.694586
0.689341	0.000721	29451.84	0.36917	0.580632	34.46063	15.35225	545.9016	0.438195
0.399314	0.000745	15182	0.400883	0.489384	25.38689	19.04196	477.4794	0.770338
0.283882	0.010301	365.3717	0.475458	0.445159	8.565931	15.80376	139.1718	1.91342
0.138549	0.001893	3061.95	0.48362	0.296147	12.69076	10.47782	146.7062	0.804106
1.02139	0.000463	14641.88	0.441581	0.427901	48.35801	30.50591	1491.395	0.625902
0.242954	0.003066	2854.961	0.405751	0.471678	12.16081	7.165888	89.5882	0.64296
0.60055	0.000702	13222.73	0.428307	0.514077	32.01804	10.76015	359.812	0.331502
0.08979	0.004655	4670.499	0.473649	0.305027	6.483624	7.851532	54.17319	1.348403
0.369043	0.00154	3339.277	0.459541	0.345405	19.82137	8.656685	154.088	0.504592
0.577797	0.000426	7801.804	0.450311	0.411242	42.72985	18.62833	862.4961	0.410001
0.724073	0.002559	668.2254	0.407816	0.595036	24.0461	13.97876	347.51	0.572767
0.572084	0.001013	14424.74	0.374681	0.590805	25.12858	9.491081	236.6234	0.390823
0.328159	0.00063	41847.04	0.385942	0.488781	25.04347	17.55511	437.9391	0.71706
1.014567	0.001457	4680.413	0.382354	0.539901	31.20474	5.091712	162.7026	0.166313
0.412737	0.001181	1992.636	0.431628	0.479752	21.6919	19.19919	469.2409	0.80235
0.46882	0.001053	3464.721	0.417984	0.476029	28.58053	12.68728	347.3067	0.477928
1.093381	0.001467	498.7078	0.39094	0.734663	39.94569	5.411088	216.839	0.143737
1.086831	0.000652	5276.762	0.381885	0.633526	45.83723	18.89006	915.8107	0.393887
0.42784	0.000788	16096.32	0.445993	0.460563	29.73776	14.58055	393.7021	0.557618
0.562819	0.002969	5016.375	0.410498	1.931332	12.82914	26.95622	247.8319	3.048687
0.270899	0.000869	4058.658	0.450799	0.381928	20.36784	10.00545	210.7844	0.505911
0.178389	0.000589	15747.39	0.47559	0.308299	20.06627	12.84074	242.154	0.714066
0.655689	0.001869	846.0564	0.409023	0.672944	21.8946	11.06399	267.2516	0.476463
0.396677	0.000674	6784.659	0.45845	0.455482	25.41555	56.66125	1412.532	2.283759
0.202621	0.006149	687.0774	0.479329	0.350542	7.759271	11.89127	102.6001	1.467031
0.197096	0.002979	1075.541	0.390721	0.483008	11.02671	11.79504	129.5214	1.208805
0.380941	0.001866	2754.858	0.368546	0.60848	19.2684	9.433739	179.2505	0.536052
0.194667	0.001322	6269.565	0.409188	0.478522	14.44112	18.71278	290.5732	1.270466
0.725661	0.001134	867.8647	0.454361	0.717227	41.85384	8.677911	401.1637	0.198825
0.385	0.000947	10468.08	0.368634	0.596223	23.70029	16.56881	411.726	0.687417
0.518747	0.001573	3650.255	0.3715	0.604339	21.60704	9.40362	213.1731	0.440817
0.253888	0.002204	2003.334	0.423236	0.454541	13.7558	15.97016	245.3647	1.085398
0.444162	0.001929	2040.738	0.43482	0.641375	14.55752	12.90091	200.4356	0.865129
0.268984	0.001406	4068.148	0.446022	0.420187	18.69879	12.38364	212.5784	0.750189
0.296317	0.00212	1158.994	0.397305	0.490671	17.02493	7.371195	123.4059	0.465953
1.014883	0.000591	2458.728	0.414166	0.670827	52.99923	8.86291	483.0224	0.167782
0.572378	0.000623	16534.04	0.399312	0.50069	33.2165	12.40875	426.808	0.369867
0.263729	0.000689	43608.05	0.448794	0.38529	21.29836	16.65352	383.2341	0.754526
0.456633	0.000726	20559.05	0.442188	0.43585	30.20448	8.880474	257.1037	0.317898
0.460627	0.001437	2391.04	0.423584	0.524423	24.89756	16.5311	413.529	0.672387
0.220983	0.001145	3336.535	0.431764	0.447164	16.29058	35.89806	576.6885	2.261483
2.775703	9.82E-05	29139.88	0.460391	0.362401	182.23	9.145707	1648.248	0.051056
0.985669	0.000805	4601.616	0.327283	0.848451	43.72837	6.508536	294.4602	0.151136
0.541405	0.000641	10826.94	0.412806	0.465796	32.49593	13.21524	457.1554	0.389886

0.501779	0.002626	885.4385	0.388265	0.635435	20.0876	6.594806	144.8335	0.331954
0.217173	0.00244	2701.442	0.439085	0.384987	12.31458	7.87479	94.95486	0.718332
1.573504	0.000441	23105.33	0.367584	1.188294	59.496	13.33063	824.7436	0.218949
0.229621	0.001431	2491.816	0.458748	0.405565	14.61534	18.67467	292.4803	1.231363
1.467741	0.000887	4917.297	0.347072	0.712329	46.71835	4.880605	232.277	0.106048
0.401845	0.000407	17702.24	0.457058	0.455629	35.75964	58.4649	2102.936	1.631053
0.175594	0.003585	844.2624	0.483976	0.306069	7.277454	7.787551	60.59364	1.14167
0.510057	0.001395	5444.076	0.371533	0.613947	23.40627	20.4581	506.5534	0.840112
0.300541	0.002099	1152.246	0.472216	0.321979	13.19393	6.930131	100.7853	0.506867
0.168442	0.001804	4083.23	0.456392	0.35277	12.74691	14.30555	208.2063	1.045942
0.236221	0.000844	8155.679	0.448379	0.379419	20.55277	18.45426	419.6231	0.846824
1.006424	0.002098	2171.389	0.283916	1.219911	31.43566	5.630251	190.5228	0.189555
0.54842	0.000622	9996.749	0.36739	0.84707	38.86113	12.10469	456.4441	0.336964
0.31251	0.000699	9944.187	0.413316	0.514615	24.54822	50.65793	1264.3	2.044839
0.554223	0.001749	10596.25	0.342439	0.684117	23.37901	10.86019	263.3205	0.466622
0.33018	0.001601	3180.878	0.330094	0.860255	20.64511	14.26979	320.4862	0.700004
0.883741	0.001472	9714.929	0.360214	0.668092	31.41813	5.417795	172.7575	0.179278
0.28313	0.001423	37901.41	0.421835	0.475012	14.71865	41.245	653.3756	2.636736
0.406785	0.007585	597.982	0.432391	0.427095	9.936949	5.702074	53.26301	0.651921
0.171447	0.001378	3517.133	0.477651	0.323389	13.15065	15.6711	234.2367	1.10062
0.187139	0.002374	1908.707	0.428899	0.407538	11.92211	9.953769	108.8076	0.989745
0.291587	0.001493	5699.513	0.399499	0.493594	17.65706	12.07589	208.7824	0.731353
0.150855	0.001815	3002.005	0.440513	0.404239	11.6791	19.8363	218.9963	1.969616
0.237905	0.004424	1097.569	0.441597	0.399728	10.9016	8.245937	97.29105	0.772913
0.095655	0.001791	20394.6	0.399857	0.459923	8.661462	22.09901	191.5341	2.769745
0.143682	0.003609	4842.115	0.454222	0.365161	7.571527	10.94239	83.61369	1.655161
0.247068	0.002915	2448.886	0.444341	0.369335	11.88712	14.43087	145.7327	1.502257
1.044691	0.000865	6185.975	0.326786	0.916104	44.88484	13.61803	659.9914	0.286744
0.480034	0.004408	731.1482	0.439639	0.522074	14.92108	11.88156	186.8825	0.785937
1.120383	0.002058	1251.68	0.370317	0.849488	30.89977	5.130677	168.063	0.169297
0.579389	0.007858	216.5626	0.378911	0.570727	12.456	3.218429	41.64118	0.294836
0.436263	0.001489	1431.672	0.361827	0.768957	27.42185	6.389307	182.3458	0.249058
0.680152	0.000969	6560.605	0.369396	0.662493	31.27748	15.12017	520.2734	0.45258
0.598427	0.001791	9727.995	0.360284	0.659977	23.40269	10.92327	268.2937	0.461768
0.952009	0.00225	1267.219	0.362558	0.58839	25.49365	4.163654	106.0223	0.171914
0.297448	0.005673	439.2393	0.402072	0.540879	11.88684	6.694908	92.75987	0.575291
0.3807	0.002047	1445.057	0.381667	0.693414	19.46224	15.58079	349.3595	0.770028
0.527696	0.001101	4277.422	0.40835	0.873902	30.53837	10.97843	348.8245	0.418245
0.921554	0.000505	5627.579	0.447957	0.582623	49.98494	30.61087	1503.588	0.62666
0.845509	0.000842	5781.663	0.305127	1.116727	41.16644	7.30426	323.5291	0.179757
0.365053	0.001578	3547.577	0.463312	0.4247	20.68836	8.909232	206.0911	0.410278
2.386937	0.003786	392.8838	0.29895	1.66928	39.32332	3.662599	157.4559	0.111871
1.14553	0.002182	1809.298	0.2884	1.394459	34.49647	5.295161	186.4591	0.177126
1.019742	0.005289	214.2983	0.233787	1.478436	21.9866	3.529634	80.95015	0.209131
0.392283	0.00119	2164.623	0.430603	0.687475	26.03443	15.68282	395.73	0.671512
0.756803	0.001753	1850.422	0.277961	1.108929	29.40705	10.2105	334.2992	0.340483
1.752505	0.002675	1359.848	0.281504	1.365997	41.72853	3.503558	154.2757	0.089167
0.367001	0.000805	7787.488	0.421026	0.483605	25.50453	35.0204	884.8518	1.399023
0.582176	0.001225	1539.753	0.418129	0.668627	29.75537	23.07849	809.1221	0.679054
0.745438	0.000429	11393.61	0.447528	0.420686	43.98819	26.37445	1269.883	0.552423
0.402009	0.002294	2623.108	0.398033	0.520466	18.89169	6.063106	112.7874	0.350201
1.298205	0.001658	1395.89	0.307095	1.118024	39.88721	5.085842	219.3596	0.128332
0.499541	0.012909	197.9515	0.360732	0.606365	10.65721	3.894818	41.57749	0.459702
0.579261	0.004944	3407.452	0.351112	0.63994	14.07171	3.814691	53.99324	0.306547
1.192336	0.002483	3554.68	0.320536	0.929659	29.61541	4.177384	131.2237	0.148207
0.761963	0.004706	587.1349	0.440836	0.43725	15.15256	5.486595	85.80385	0.368157
0.381247	0.000476	19496.16	0.47426	0.319055	30.71457	20.28105	639.6645	0.66488
1.788364	0.00213	1407.672	0.287597	1.340937	41.34196	5.343203	242.3936	0.129456

0.87703	0.001474	6184.039	0.319003	1.005495	34.40139	10.57672	376.1561	0.310464
0.287594	0.002386	2648.314	0.351019	0.639433	15.04494	11.27006	174.0679	0.80564
1.292362	0.004287	1261.338	0.243024	1.683468	30.26945	3.583776	118.0693	0.140258
0.574099	0.001079	1915.96	0.411855	0.598765	29.68319	14.30915	490.9583	0.431261
1.088627	0.001367	6710.542	0.320714	0.823898	35.80432	4.794848	175.2826	0.139201
1.907186	0.002627	921.5731	0.248802	1.515879	39.29257	3.982	173.2046	0.103971
0.392781	0.001492	4398.462	0.356553	0.786097	22.57995	21.37165	525.0362	0.905126
1.177786	0.00192	2304.444	0.285225	1.156969	35.52214	5.039518	185.6537	0.149733
2.614487	0.00074	80091.71	0.301317	2.898763	53.23341	7.131795	354.0253	0.149328
1.015062	0.003157	2916.349	0.280775	1.546174	32.28531	7.36988	258.2048	0.238476
1.19288	0.00289	1235.655	0.289657	1.41203	33.99901	3.979522	142.2678	0.136084
0.851573	0.000395	35502.36	0.325378	1.055491	56.289	31.38836	1966.355	0.508324
0.57732	0.000971	7744.399	0.346816	0.961133	31.78048	19.38249	677.2738	0.589465
0.61507	0.001587	13647.71	0.3698	0.733479	28.7016	7.730371	232.8134	0.27389
0.802769	0.006971	436.7478	0.484091	0.415228	14.78481	6.085395	93.46953	0.419378
0.598228	0.002684	2919.052	0.404173	0.638272	20.50282	7.788157	178.6239	0.371614
0.457729	0.001947	12829.14	0.349977	1.036539	27.33517	6.928977	197.1847	0.284746
1.121153	0.002306	5754.798	0.316819	0.958424	31.77064	5.166223	175.0738	0.165366
1.122341	0.002572	918.6226	0.349696	1.058102	30.19328	10.35249	351.7842	0.328985
0.938924	0.001048	1504.805	0.37772	1.006828	40.29217	8.770082	389.3503	0.217888
0.292786	0.000911	7139.017	0.391514	0.563649	22.42049	17.55438	394.8241	0.833115
0.38093	0.009065	278.9354	0.469475	0.34625	7.369228	4.652775	37.57704	0.663316
0.497024	0.001041	14752.36	0.402667	0.483521	23.16843	13.1694	320.7308	0.554558
0.789841	0.001034	5511.5	0.395706	0.589849	31.58099	14.71443	514.1935	0.430401
1.730161	0.015029	86.29494	0.208333	2.141008	22.24824	3.079079	81.80208	0.190207
0.881435	0.001685	4563.993	0.382864	0.708656	30.31156	6.787167	224.0297	0.218068
0.987044	0.000192	15710.81	0.453096	0.531231	90.29717	13.40776	1245.716	0.146392
0.46796	0.001675	5224.841	0.416433	0.456333	20.21325	7.107543	139.2476	0.386447
1.22411	0.001618	3835.586	0.344426	0.769212	37.00151	4.342573	161.6836	0.123411
0.618305	0.002967	308.4227	0.286231	1.050518	25.05823	4.36075	117.5858	0.191288
1.870211	0.003597	2448.159	0.281275	1.560548	41.78337	3.530888	155.343	0.094283
0.438589	0.002846	2629.466	0.331413	1.049987	26.62078	8.523465	275.7403	0.290372
1.005801	0.001486	1835.892	0.375217	1.034949	40.27871	6.47289	278.7783	0.162162
0.545538	0.005132	1757.634	0.269654	1.602908	23.57641	9.121838	233.785	0.436016
0.760105	0.012089	61.6372	0.306371	2.271586	16.11924	6.869543	142.0076	0.690258
0.134613	0.003073	2227.067	0.560794	0.275182	8.216248	33.66922	300.8158	3.82425
0.103539	0.012055	355.2032	0.621198	0.217582	3.577392	13.72707	53.48594	3.847408
0.169937	0.00117	6966.68	0.474395	0.331978	13.76736	23.35504	361.5342	1.548672
0.126447	0.002594	16553.92	0.441139	0.390599	7.862858	42.20843	374.7385	4.864719
0.29152	0.001834	2590.793	0.423688	0.447935	18.04092	15.75234	259.3744	0.975966
0.173016	0.001954	9036.233	0.44782	0.383706	12.73691	14.49996	214.6235	1.040685
0.258446	0.002631	2468.995	0.456818	0.360868	12.95091	9.115079	136.2161	0.644611
0.567681	0.002329	1482.735	0.419756	0.455112	20.61023	6.772921	154.9486	0.311968
0.285596	0.002655	1353.384	0.418768	0.511498	13.16728	24.19575	376.4232	1.623967
0.532413	0.001965	4526.754	0.389	0.549537	21.51129	10.74662	258.9594	0.460091
1.35677	0.000938	28257.74	0.318257	0.802923	44.50724	4.466314	202.4823	0.102901
0.165033	0.000981	9327.932	0.413674	0.486732	16.328	40.32738	651.8813	2.545823
0.61226	0.002524	1075.439	0.332021	0.939852	26.53265	5.997185	165.806	0.240482
0.239596	0.001927	1844.288	0.480333	0.31136	13.23327	9.809228	145.215	0.697488
0.433837	0.005243	679.1544	0.386529	0.535406	13.34776	6.534559	95.91025	0.487198
0.428857	0.00053	24699.1	0.470173	0.3138	30.53602	9.16309	277.8569	0.312826
0.504836	0.004324	455.1782	0.407876	0.49669	14.94922	6.227521	96.54266	0.428872
1.180166	0.000824	12087.24	0.341364	0.814012	48.90717	7.354269	361.9453	0.153447
0.135048	0.007512	500.4926	0.646211	0.207379	4.774292	21.77544	90.30753	5.413675
1.080093	0.001183	1610.824	0.407333	0.576979	36.27965	7.766623	280.4077	0.220007
0.899875	0.003274	1275.66	0.183377	3.276443	37.02201	6.827209	297.5494	0.242864

16.20744	5.13E-05	5929.149	0.294337	2.642162	806.0124	22.15562	18417.98	0.026781
0.65465	0.000821	35480.27	0.368813	1.103636	40.19212	13.76337	624.8882	0.326766
0.787057	0.000375	26182.57	0.383615	0.547042	50.01988	25.39397	1256.492	0.516866
2.655879	0.000867	12061.66	0.388504	5.819795	41.96474	20.47308	740.6001	0.576931
0.477447	0.001057	5764.471	0.415846	0.540529	26.74267	21.19553	536.3408	0.847414
0.685099	0.001084	2241.268	0.386492	0.711227	38.5137	8.046732	306.4583	0.221682
0.246217	0.002405	2176.55	0.467658	0.397901	13.60736	22.84558	356.8964	1.503011
0.086764	0.002577	7703.586	0.500124	0.270954	7.018794	13.48453	114.8919	1.682406
0.087815	0.00997	1211.987	0.51696	0.310894	3.685654	18.75429	73.52843	5.234502
0.737331	0.00293	1122.259	0.303075	1.019101	26.57272	6.96264	225.8974	0.236533
0.580354	0.002022	972.1867	0.360449	0.787279	21.66333	13.72636	332.623	0.595731
0.511938	0.000624	4914.925	0.426308	0.512121	39.49472	12.32584	473.9683	0.32816
0.717222	0.00226	294.5458	0.406845	1.24737	32.57753	13.69875	487.6362	0.413595
0.24101	0.005951	1336.354	0.409185	0.47141	8.249818	12.7315	111.1984	1.564872
0.259629	0.001344	4737.039	0.497612	0.377702	16.62586	60.14681	963.5899	3.769154
0.215846	0.004955	556.4836	0.482308	0.452275	8.660712	12.08335	108.7275	1.411078
0.302049	0.000766	1597.576	0.510076	0.37852	23.86722	51.02956	1272.371	2.055787
0.557944	0.000687	17274.14	0.429717	0.439903	31.72277	19.34362	668.4124	0.569985
0.549539	0.000908	9711.927	0.358509	0.726734	31.153	12.414	427.354	0.374291
0.56886	0.001989	4140.311	0.388275	0.681325	22.01349	26.0107	643.0899	1.076253
0.85575	0.001357	4709.065	0.361899	0.729719	32.18712	10.11482	353.7663	0.299334
0.615644	0.000754	19537.94	0.369948	0.607885	33.45395	8.419198	282.27	0.262435
0.325743	0.002038	6283.058	0.38498	0.551162	15.80762	26.74864	424.7342	1.720228
0.516088	0.040058	33.0882	0.359445	0.55432	4.937341	9.191714	43.50351	2.500655
1.330109	0.001839	1609.603	0.33981	0.745942	33.53455	6.192502	216.3833	0.184443
0.767211	0.000699	8942.617	0.412179	0.526416	37.22268	20.38565	736.0059	0.568986
1.040246	0.000957	11657.69	0.357724	1.182164	43.45478	19.68581	940.7579	0.430723
1.429196	0.001842	3251.105	0.193546	4.334581	49.8889	6.566622	377.9736	0.175261
1.09704	0.003687	278.7233	0.323034	1.047356	30.22854	7.249922	246.4534	0.229563
1.52464	0.00515	198.8252	0.257348	3.635799	34.90964	4.483589	180.6776	0.336011
0.81248	0.001091	2868.059	0.335593	1.852538	40.95914	34.5993	1668.506	0.756262
2.765502	0.001158	762.918	0.2546	2.046981	83.54685	4.953859	430.4504	0.062947

PX78	PX79	PX80	PX81	PX82	PX83	PX84	PX85	PX86
0.056501	3.227219	3506.488	0.350801	0.511806	2.954315	0.57569	11.30572	0.033377
0.190197	3.619611	3599.826	0.262121	0.380781	12.9074	0.484046	3.499927	0.090087
0.057513	3.469006	7496.683	0.360604	0.51579	2.644129	0.603957	11.73172	0.036077
0.131456	2.299086	239.8025	0.419583	0.474611	12.31507	0.480903	4.008541	0.071533
0.050987	3.481431	805.6392	0.33974	0.461682	7.045609	0.5902	12.86328	0.033177
0.06534	3.687212	2151.813	0.284326	0.386473	11.45028	0.534717	9.692528	0.037089
0.041244	3.239419	1932.772	0.437501	0.58112	1.762992	0.677568	18.95801	0.029193
0.047594	3.632441	2675.371	0.363258	0.478525	4.797095	0.610722	15.86683	0.031983
0.070048	2.964688	288.6835	0.472184	0.64372	1.656079	0.688931	12.46164	0.053947
0.036366	3.469721	8587	0.410534	0.547911	2.234901	0.654869	20.11224	0.025176
0.009299	3.914505	21643.17	0.30416	0.402466	7.448101	0.557195	60.92315	0.005363
0.05566	3.809407	58686.44	0.287876	0.423742	5.279943	0.523202	10.55063	0.030069
0.036076	3.613483	4699.862	0.328648	0.43957	6.506495	0.577715	16.89684	0.022635
0.055366	3.116612	1114.394	0.400653	0.575996	1.742966	0.631682	12.82338	0.035932
0.08796	2.959718	346.7043	0.368629	0.529989	6.36186	0.588783	7.820549	0.058034
0.001414	3.967061	11069.55	0.290414	0.331926	16.43093	0.547648	989.2488	0.001068
0.139915	3.42206	732.7236	0.348885	0.481169	5.041691	0.591075	6.101945	0.092088
0.038741	3.701052	7385.42	0.329889	0.4629	4.501889	0.575104	16.16419	0.02415
0.033325	3.626883	2540.756	0.277638	0.371867	10.65031	0.530929	16.63411	0.019193
0.01794	3.9329	13617.9	0.299637	0.394021	7.598242	0.557443	32.57818	0.010343
0.049339	3.727686	19955.26	0.280923	0.4011	6.71049	0.525636	11.48601	0.026577
0.035991	3.582781	8516.972	0.364	0.4985	3.138724	0.613772	18.75436	0.023137
0.075833	2.679291	594.1218	0.405005	0.548015	8.175226	0.582472	8.441343	0.047263
0.050494	3.842441	9893.668	0.2507	0.365078	13.51426	0.485846	10.13174	0.026261
0.021862	3.870587	5075.951	0.280288	0.367693	12.05299	0.530649	26.42601	0.012249
0.095647	3.466896	725.603	0.323728	0.457023	8.829822	0.568642	7.550377	0.063788
0.056512	3.54486	2603.176	0.273341	0.389891	9.908847	0.511375	10.34508	0.028305
0.08012	3.630062	2997.171	0.265383	0.409385	8.179587	0.497632	6.93949	0.041193
0.06209	3.71167	1559.317	0.323948	0.450535	6.642704	0.572969	11.25344	0.041111
0.064749	3.697207	3091.969	0.358408	0.480913	4.276013	0.609182	11.56438	0.044852
0.056536	2.995641	579.3527	0.400866	0.569593	2.766728	0.637359	12.54092	0.039459
0.034287	3.760448	8858.896	0.305729	0.421456	7.029678	0.554979	17.49814	0.020377
0.008908	3.059834	850.5285	0.382339	0.547916	1.980686	0.621398	1109.724	0.00755
0.035591	3.662089	8341.515	0.292277	0.40872	8.909777	0.5304	15.52983	0.020477
0.049195	3.708907	37295.74	0.267688	0.369379	9.2442	0.516297	10.94843	0.026426
0.023513	3.282928	1389.741	0.385958	0.52697	3.83296	0.625255	28.89729	0.015505
0.032564	3.929033	8476.862	0.24501	0.32855	13.2676	0.49812	15.90884	0.017039
0.070128	3.335276	518.7401	0.318897	0.423014	7.968759	0.574468	9.892984	0.043356
0.132427	3.246948	714.3335	0.285484	0.413208	8.83178	0.532721	4.80878	0.078173
0.050105	3.230025	575.2221	0.477921	0.63238	1.544194	0.684133	16.58478	0.036971
0.020257	3.753214	3870.762	0.35463	0.46179	6.02279	0.605075	33.38538	0.013404
0.042787	3.692956	36529.3	0.32858	0.46624	3.569365	0.573621	15.04252	0.025076
0.097734	3.489771	1940.746	0.317996	0.469093	4.497117	0.558192	6.663932	0.05781
0.050203	3.795803	8625.076	0.274138	0.386842	11.86022	0.516116	11.27813	0.02805
0.076396	3.655663	5500.226	0.303369	0.397419	8.870321	0.5573	8.448114	0.045503
0.191954	2.949306	934.5335	0.371597	0.545777	1.84711	0.599641	3.807367	0.119658
0.035515	3.401238	1331.693	0.330261	0.459915	5.260787	0.570505	16.66363	0.021987
0.088631	3.730156	4162.809	0.26511	0.396582	12.22396	0.50239	6.297571	0.047872
0.081289	3.378741	4118.243	0.356019	0.509205	2.894244	0.601238	8.643002	0.050638
0.080551	3.566326	5176.153	0.289043	0.393736	9.209625	0.541645	7.35691	0.047328
0.029287	3.744233	8177.65	0.301746	0.397996	8.880872	0.552173	20.13698	0.016914
0.056981	3.140965	838.5106	0.409988	0.583068	1.904408	0.64404	12.66703	0.038979
0.060255	3.346459	1602.785	0.351562	0.50516	2.95566	0.595645	11.303	0.037726
0.082893	3.55042	1716.609	0.300431	0.378765	13.59223	0.552491	7.188991	0.050794
0.030436	4.026407	12480.7	0.327429	0.420533	7.273015	0.5797	21.51136	0.01885
0.081269	3.442025	2475.721	0.260935	0.379272	10.10854	0.505466	6.38719	0.045697
0.036077	3.680938	2523.809	0.295103	0.407845	8.487919	0.544093	15.56728	0.021699

0.040604	3.2647	1390.995	0.42361	0.573863	1.953926	0.655715	18.16797	0.028244
0.162447	3.148428	889.7468	0.350302	0.498831	4.206074	0.594625	4.37351	0.103445
0.027864	3.741371	2903.661	0.357788	0.498221	4.46517	0.597787	24.70738	0.018209
0.031993	4.333903	71278.82	0.290874	0.427389	7.109733	0.521873	18.54225	0.016726
0.014249	3.386177	1206.623	0.36589	0.521443	4.302546	0.606192	44.94284	0.009012
0.024822	3.575599	3282.311	0.3113	0.4056	14.09082	0.568125	23.9473	0.015371
0.033342	3.629949	11287.78	0.310477	0.415597	7.429451	0.564723	17.69857	0.019773
0.030138	3.682997	8240.585	0.318258	0.433574	5.314175	0.574202	20.35048	0.017649
0.081341	3.286532	866.8454	0.330142	0.455448	5.704044	0.581528	7.952414	0.052112
0.169265	3.076724	441.3337	0.335763	0.488904	3.800514	0.583641	4.069977	0.111389
0.181247	2.929686	562.2342	0.40424	0.539766	3.658202	0.645531	4.278661	0.128149
0.057324	3.495365	10713.2	0.341775	0.474464	3.638983	0.590401	11.50894	0.034779
0.033916	3.512246	41036.62	0.301556	0.441173	3.998637	0.545772	16.92295	0.018693
0.033024	3.840956	12694.63	0.248743	0.365151	11.64362	0.492194	15.92353	0.016463
0.031381	3.760206	25601.43	0.312187	0.395465	8.105038	0.5696	19.3466	0.018724
0.042849	3.822763	10688.88	0.27392	0.363971	10.13513	0.526252	13.38226	0.023358
0.164509	2.782593	322.8125	0.396105	0.525781	7.854887	0.566237	4.685462	0.11105
0.088552	3.183237	2184.151	0.326246	0.482423	4.659237	0.568219	6.969435	0.053222
0.021596	3.796852	9085.101	0.269762	0.313063	17.95596	0.530943	25.62447	0.01179
0.101204	3.406725	2654.102	0.360456	0.509783	2.652899	0.600846	7.180055	0.0654
0.033386	3.566568	10074.88	0.315427	0.449808	4.889142	0.564777	17.85942	0.019517
0.192154	3.162805	3528.72	0.343354	0.501702	3.06909	0.583583	3.673106	0.119015
0.054955	3.182833	2632.911	0.34896	0.488149	3.693747	0.596284	12.20595	0.032522
0.024558	3.697099	4827.434	0.263494	0.389823	9.517972	0.506	20.59413	0.013252
0.048527	3.405152	525.3469	0.318718	0.435351	7.067703	0.574315	13.42849	0.03099
0.043801	3.538793	13630.55	0.34537	0.460655	4.335342	0.597723	15.11042	0.026849
0.043392	3.840492	31144.74	0.275973	0.372926	9.017246	0.523402	13.13545	0.023376
0.034642	3.280707	5062.086	0.409482	0.560232	1.792124	0.658543	20.41915	0.023395
0.051813	3.615021	1432.258	0.295251	0.381825	10.03169	0.547444	11.07533	0.031612
0.03824	3.634003	2657.832	0.307495	0.424353	6.125224	0.556898	15.79416	0.022256
0.029067	3.255605	555.7199	0.414914	0.573194	1.826991	0.650695	25.63478	0.020054
0.023801	3.733751	4358.344	0.306339	0.38052	10.38034	0.565412	25.08905	0.014465
0.037746	3.672456	10551.06	0.285105	0.388564	7.282478	0.539013	16.44033	0.021018
0.120815	3.906707	3582.613	0.284407	0.349896	15.65194	0.543468	8.337473	0.069022
0.05412	3.408534	3075.442	0.31904	0.473427	4.001338	0.551544	11.01797	0.031244
0.053548	3.372055	11236.14	0.307741	0.453825	5.227438	0.526654	10.74528	0.028175
0.054534	3.359527	786.2254	0.362415	0.481361	5.211138	0.610938	12.72925	0.037024
0.042458	4.117069	3117.245	0.209634	0.254174	35.10422	0.461815	12.06455	0.020186
0.163057	3.05408	524.0531	0.345111	0.499189	5.623136	0.585767	4.319357	0.107833
0.112634	3.223365	1196.751	0.388286	0.555953	5.466075	0.602087	6.557958	0.072063
0.061603	3.511135	3084.83	0.381556	0.523586	4.104569	0.615274	11.92362	0.03956
0.080844	3.749595	4860.091	0.293203	0.416541	9.533216	0.530408	7.37679	0.046441
0.028234	3.175713	755.8318	0.373269	0.521071	3.65841	0.614392	24.55951	0.019063
0.047751	3.8101	9292.17	0.308425	0.414733	8.613493	0.554883	12.81899	0.028418
0.05361	3.641328	3486.388	0.342966	0.469995	4.145016	0.596438	12.61339	0.034197
0.086064	3.481597	1577.495	0.311757	0.443514	8.054475	0.554889	7.243296	0.053078
0.081937	3.431697	1672.079	0.342107	0.450155	6.505686	0.59518	8.500969	0.052733
0.060596	3.486323	2936.4	0.305207	0.438894	5.688669	0.549279	10.41847	0.034621
0.067467	3.242543	1219.083	0.390696	0.544537	2.776704	0.621879	10.46858	0.04404
0.020829	3.448728	2282.587	0.363346	0.514423	3.792468	0.602941	31.20218	0.013367
0.032248	3.606396	14482.89	0.330205	0.456175	5.894375	0.573009	18.87774	0.019037
0.051515	3.66594	29708.49	0.284767	0.406718	8.155728	0.519401	10.76972	0.028286
0.035326	3.442534	15678.46	0.326975	0.468821	3.74243	0.572323	17.39681	0.020499
0.046154	3.471965	1779.146	0.302757	0.424776	8.410332	0.549986	13.57878	0.027644
0.070018	3.671035	2122.498	0.25923	0.357226	21.25342	0.502645	8.265491	0.037585
0.00555	3.410883	20389.77	0.315739	0.454918	3.884394	0.564228	102.8485	0.003143
0.025788	3.590107	5985.493	0.406119	0.552261	2.523953	0.644457	27.71121	0.017454
0.032819	3.528823	9288.59	0.331881	0.451245	6.35995	0.573737	18.25256	0.019623

0.061338	3.309438	950.2558	0.395883	0.553161	2.515621	0.638436	12.28937	0.042839
0.094699	3.217427	2424.733	0.366662	0.530557	2.968218	0.595858	7.378236	0.058195
0.018098	3.850677	21878.56	0.337548	0.422778	6.973535	0.592925	35.08704	0.011133
0.079603	3.407973	1818.814	0.310332	0.433113	9.573063	0.546193	7.755272	0.047144
0.023237	3.295807	6478.197	0.447381	0.587516	1.754759	0.686628	31.91289	0.016362
0.029992	4.05702	9494.762	0.232925	0.28005	36.0592	0.479661	17.05289	0.015162
0.166898	2.994177	703.2835	0.378513	0.536222	3.076127	0.616604	4.44846	0.106386
0.049409	3.762722	4664.043	0.309012	0.366612	11.5288	0.568203	12.84381	0.030962
0.084632	3.01078	928.0623	0.36375	0.52633	2.524951	0.603698	7.645115	0.054195
0.089829	3.393651	2957.443	0.305855	0.449609	6.664385	0.540946	6.506498	0.053069
0.053343	3.629305	5726.817	0.290026	0.414521	9.501486	0.52616	10.43431	0.029853
0.042201	3.620221	3594.808	0.449521	0.581078	2.17175	0.685229	20.78462	0.031686
0.029138	3.877177	9367.811	0.320535	0.463508	5.418425	0.557528	21.66183	0.017103
0.045243	3.985065	6227.682	0.24399	0.300841	30.88515	0.491231	11.80328	0.024156
0.049862	3.705204	11305.93	0.356789	0.449948	5.365786	0.612922	14.15703	0.032454
0.064412	3.928413	3460.596	0.333893	0.456595	7.130262	0.576784	11.3784	0.042359
0.035571	3.431581	11121.07	0.404639	0.554824	1.942097	0.651116	20.30819	0.024059
0.078188	4.032315	22808.73	0.248502	0.276076	25.65778	0.508297	7.260983	0.04385
0.123843	2.835467	621.1449	0.436791	0.590859	2.199969	0.674243	6.856487	0.086648
0.085894	3.360377	2406.571	0.301313	0.444233	7.52101	0.535624	6.695998	0.049703
0.099852	3.293174	1703.905	0.352357	0.506242	4.125168	0.583101	7.097258	0.0599
0.064644	3.562871	4945.259	0.327004	0.467511	5.648448	0.569294	9.959391	0.038816
0.106324	3.497204	2309.737	0.305851	0.450922	10.56138	0.537707	6.3874	0.060645
0.109356	3.195029	977.6219	0.377058	0.524202	3.53818	0.623485	6.437095	0.07405
0.146646	3.853872	14648.4	0.265361	0.383254	11.35471	0.497495	4.346038	0.077756
0.166352	3.418248	3525.996	0.314394	0.465552	4.774401	0.553679	4.243816	0.095846
0.09738	3.456285	1732.029	0.304422	0.416835	7.373013	0.559603	6.986953	0.054555
0.025179	3.828978	6886.68	0.352985	0.424355	7.250475	0.609232	26.09899	0.016745
0.082959	3.208228	642.7353	0.375085	0.499302	5.884737	0.623209	9.022516	0.057352
0.038507	3.281516	1559.923	0.446951	0.594875	1.862651	0.684657	20.763	0.028053
0.106095	2.733115	317.7462	0.533992	0.704826	0.827103	0.740541	9.112627	0.083003
0.043484	3.308011	1841.416	0.426416	0.586625	2.266834	0.649759	17.19829	0.030368
0.035648	3.69149	6184.852	0.332268	0.44369	8.048988	0.584471	17.45815	0.022576
0.050393	3.581493	10098.17	0.363573	0.455724	5.39665	0.618211	14.11023	0.033785
0.043833	3.049077	1748.391	0.489392	0.630159	1.38879	0.719284	18.27023	0.032347
0.115439	2.957947	595.2197	0.497721	0.61991	2.685667	0.708205	7.986708	0.089127
0.066904	3.398183	1595.655	0.381155	0.521855	8.28212	0.612667	11.17502	0.046186
0.045941	3.588834	3712.236	0.331801	0.486043	4.809336	0.574009	17.03058	0.03073
0.021507	3.655888	3795.351	0.294866	0.379912	18.97691	0.551739	27.64732	0.012359
0.029281	3.768016	7908.736	0.394698	0.531549	2.905454	0.631105	25.00438	0.020117
0.055104	3.134926	2983.645	0.367337	0.51946	3.864619	0.608193	11.94143	0.036431
0.040796	3.2432	789.7455	0.576029	0.70168	1.235483	0.775303	29.66916	0.034303
0.038075	3.450715	3299.02	0.495504	0.629195	2.075265	0.717326	24.09966	0.029372
0.066456	3.172519	655.125	0.650495	0.760031	1.052474	0.801147	17.03815	0.055825
0.049015	3.530616	1778.689	0.332357	0.456271	8.035169	0.57706	14.82218	0.03153
0.04218	3.623845	3430.569	0.468054	0.58715	5.03643	0.684668	19.32946	0.031033
0.030247	3.428612	2514.955	0.511611	0.658589	1.051324	0.737611	30.1193	0.023853
0.043174	3.944961	4915.645	0.259807	0.319905	21.65996	0.516096	12.97709	0.023823
0.039379	3.447596	1296.328	0.332745	0.421045	13.86128	0.586218	15.91046	0.026113
0.023939	3.748213	6944.571	0.264318	0.34104	15.34737	0.519074	21.77273	0.013293
0.061711	3.230443	2717.191	0.397438	0.554572	2.232635	0.640321	12.04462	0.041445
0.029804	3.527261	2141.551	0.451936	0.596606	1.822938	0.687776	26.4649	0.02204
0.135363	2.598489	384.1898	0.639314	0.753697	1.271442	0.794514	8.503173	0.110878
0.08903	3.131396	4785.456	0.482791	0.636816	1.149931	0.713626	10.07719	0.065986
0.041386	3.32619	5566.143	0.487469	0.631893	1.373059	0.716444	20.73077	0.031562
0.076035	2.803448	672.7124	0.490182	0.620323	2.197402	0.716451	10.76841	0.056588
0.034144	3.624532	11724.09	0.270292	0.395633	11.0506	0.514466	15.66315	0.017917
0.030472	3.529856	2386.369	0.471462	0.597691	2.108522	0.705841	28.1106	0.023363

0.03517	3.682587	7785.724	0.387	0.479191	5.241873	0.637528	21.34551	0.024462
0.083364	3.571455	3181.496	0.388115	0.520627	5.348058	0.624236	9.240404	0.055684
0.051189	3.506984	2885.44	0.540606	0.670792	1.15857	0.756404	22.04933	0.042312
0.038185	3.430102	1649.299	0.336852	0.466119	7.539578	0.586939	16.18448	0.024841
0.031921	3.404429	9685.297	0.452052	0.593764	1.698251	0.690139	24.32191	0.023063
0.032074	3.440396	2067.125	0.538103	0.668162	1.404079	0.753769	28.45478	0.025893
0.056398	3.74147	4244.415	0.324706	0.436869	12.23065	0.570873	12.24593	0.036677
0.034455	3.547095	3866.628	0.464172	0.595909	1.887101	0.700699	24.26208	0.025819
0.022091	3.812936	111002	0.410892	0.518105	3.198342	0.660651	36.38	0.014959
0.046038	3.661178	4848.851	0.449443	0.557669	3.371024	0.686712	21.20896	0.03586
0.044252	3.41933	2317.003	0.522496	0.665639	1.301513	0.737993	24.48608	0.035645
0.019957	4.168313	35670.01	0.309978	0.342937	19.72484	0.564082	30.07014	0.012388
0.039815	3.877493	8297.677	0.348234	0.426225	10.89101	0.596923	18.02281	0.026786
0.040932	3.598043	13824.47	0.364643	0.499903	3.290665	0.61673	17.12394	0.02745
0.080763	2.534284	519.3713	0.541394	0.683111	2.532915	0.738348	10.90269	0.061946
0.061971	3.22514	3189.017	0.420229	0.559679	3.393276	0.658977	12.95508	0.044927
0.049904	3.594066	15070.69	0.391197	0.53151	2.719034	0.632424	16.55205	0.035837
0.038816	3.420928	8550.109	0.460858	0.584371	1.986456	0.699516	21.68717	0.029173
0.043236	3.42021	1256.957	0.447677	0.555673	5.141554	0.677719	19.58001	0.032336
0.029531	3.351417	1833.506	0.424139	0.566052	3.968431	0.656834	25.23318	0.021008
0.050999	3.882628	5956.714	0.303283	0.422463	9.000508	0.544953	11.93354	0.029717
0.172318	2.418075	341.4756	0.53501	0.69004	1.55456	0.733994	5.291805	0.132333
0.047601	3.523571	12601.93	0.331199	0.440851	6.673751	0.58363	13.29434	0.029201
0.034963	3.540948	5042.522	0.348524	0.440302	7.833562	0.603267	18.13162	0.022809
0.082734	3.22054	299.2782	0.681892	0.77859	0.94237	0.832098	17.69177	0.073044
0.038196	3.343702	5114.307	0.41205	0.553739	2.808081	0.655289	19.17157	0.026905
0.011545	3.561014	10840.27	0.295998	0.44418	6.440262	0.529507	47.22216	0.006333
0.055325	3.25717	5066.299	0.385425	0.535972	2.796606	0.625672	12.75265	0.035311
0.030645	3.213099	5397.35	0.471307	0.62142	1.45398	0.703835	25.71649	0.022479
0.051268	3.217168	608.8175	0.521334	0.67956	1.408126	0.725792	17.42481	0.039956
0.033378	3.490118	4478.018	0.508564	0.649425	1.080192	0.737896	30.17234	0.026864
0.049912	3.586777	3270.023	0.39153	0.51796	3.810021	0.634473	15.30277	0.036921
0.030676	3.496864	2033.114	0.401999	0.541666	2.541289	0.64911	25.28337	0.021922
0.074961	3.811707	2852.525	0.419403	0.518794	4.555449	0.665126	14.56351	0.059666
0.191034	2.951374	98.91181	0.47158	0.620347	2.597871	0.684143	9.352237	0.158745
0.143666	3.208703	1011.654	0.244444	0.377002	19.00749	0.461135	3.542416	0.077508
0.400021	2.660901	225.4716	0.363642	0.529192	6.165301	0.584334	2.030174	0.264236
0.080765	3.620936	4069.374	0.261376	0.367226	12.66574	0.499349	6.512085	0.043909
0.160791	3.942345	9295.581	0.238894	0.276217	25.53331	0.492974	3.641293	0.092862
0.062876	3.546634	1878.598	0.296194	0.397173	8.045118	0.548776	10.12031	0.035975
0.092172	3.540669	6244.164	0.293058	0.418306	7.087424	0.537573	6.362811	0.055512
0.089085	3.169888	2027.775	0.358339	0.488528	4.020847	0.604494	7.371651	0.058862
0.05451	3.176443	1415.175	0.385125	0.533841	2.647327	0.630698	12.42622	0.036937
0.09776	3.475591	1018.749	0.297422	0.39256	13.53667	0.546997	6.419993	0.064114
0.052958	3.478974	4106.84	0.344004	0.450238	5.106468	0.600084	12.2448	0.03489
0.024422	3.440875	39901.06	0.443568	0.59132	1.508409	0.685601	30.33583	0.017146
0.072383	3.9303	6306.049	0.254382	0.343748	22.91051	0.48911	7.936719	0.038818
0.048407	3.409115	1501.879	0.438054	0.589941	2.284827	0.670744	17.16427	0.035664
0.084049	3.202559	1287.149	0.32422	0.469669	4.318472	0.572494	7.215293	0.051523
0.093364	3.153701	769.5704	0.417269	0.563597	2.546878	0.65688	8.412648	0.06716
0.034199	3.32733	17268.53	0.319087	0.465686	3.875419	0.563653	17.24232	0.019372
0.079922	2.97595	508.7492	0.435448	0.59714	2.46765	0.669092	9.790133	0.057137
0.022776	3.491916	14330	0.397262	0.508679	3.208136	0.64769	31.36431	0.015457
0.258907	2.432225	368.7544	0.423529	0.573003	9.950888	0.618273	3.075743	0.164996
0.030283	3.197055	1577.403	0.388115	0.531573	3.342147	0.634535	22.94813	0.01997
0.056193	4.044809	3768.842	0.496773	0.617478	2.963408	0.707226	24.23593	0.045364

0.001333	4.56744	6359.024	0.311189	0.350655	13.11968	0.574653	456.8523	0.000812
0.032321	3.917999	32857.99	0.32545	0.428405	7.120986	0.577154	21.82954	0.021516
0.021073	3.958238	20276.54	0.286669	0.340947	15.07567	0.542915	27.09001	0.011753
0.031369	3.866874	9699.261	0.307375	0.360091	11.60538	0.569571	26.68673	0.018816
0.04145	3.776537	3981.047	0.279902	0.365308	11.83989	0.536021	14.59528	0.023155
0.030769	3.457019	2239.532	0.367545	0.517838	3.370489	0.609053	23.12754	0.020365
0.087325	3.545482	1318.748	0.271706	0.379247	12.33024	0.523258	6.538063	0.052839
0.16924	3.276182	4625.98	0.288505	0.409763	6.323373	0.534186	3.382765	0.101852
0.425774	3.165563	743.6855	0.299561	0.43242	9.532829	0.533591	1.940141	0.265565
0.047433	3.464457	1663.461	0.428718	0.555774	3.010913	0.669776	16.1796	0.035952
0.057248	3.550392	1011.474	0.35924	0.465688	7.250581	0.609003	12.4874	0.039071
0.027477	3.478364	4134.788	0.330355	0.47277	5.437227	0.561678	22.09401	0.016089
0.047139	3.324828	275.1905	0.368467	0.518756	6.504893	0.601191	18.52735	0.034084
0.164514	3.358167	1247.076	0.362677	0.463717	6.372248	0.611048	4.900239	0.113095
0.06816	3.885647	1917.354	0.201748	0.253425	36.08567	0.445874	7.620452	0.033099
0.144548	2.993444	458.2484	0.373151	0.516361	5.682091	0.60443	5.227578	0.094096
0.04564	3.748654	692.3413	0.212286	0.292311	28.53466	0.446469	10.17102	0.022704
0.033663	3.778891	11798.91	0.28641	0.370896	10.9499	0.541624	16.75348	0.019155
0.036712	3.701088	9897.469	0.344957	0.451577	6.071768	0.590314	17.52832	0.023689
0.057069	3.77807	3374.156	0.309302	0.340984	15.21457	0.570644	11.69627	0.03801
0.035582	3.569962	4933.376	0.37009	0.464306	4.958941	0.625372	19.38412	0.024139
0.032742	3.686916	18433.03	0.338428	0.480219	3.472296	0.585369	19.6235	0.019666
0.076159	3.851354	4764.331	0.284072	0.335689	15.53323	0.543794	8.570427	0.045496
0.375668	3.376082	30.41804	0.317064	0.464646	3.656764	0.562407	2.884512	0.238513
0.033629	3.244044	2235.6	0.456663	0.582647	2.607415	0.693893	22.84722	0.024479
0.028901	3.67871	6468.421	0.290898	0.367527	11.07053	0.549193	20.66191	0.016374
0.030787	4.051722	10161.73	0.302379	0.369847	10.91669	0.561867	23.27058	0.020373
0.039415	4.16195	8578.646	0.477596	0.582634	2.833732	0.700697	32.70924	0.030949
0.042991	3.398833	393.1423	0.442142	0.564411	3.2037	0.683667	19.66166	0.032736
0.098053	3.555423	439.3343	0.532097	0.676418	1.51781	0.737618	24.25499	0.080593
0.037491	4.115516	2571.322	0.292895	0.344819	21.50088	0.548054	20.08365	0.025873
0.015145	3.77464	1552.24	0.486345	0.63385	1.700888	0.706609	57.88503	0.011545

PX87	PX88	PX89	PX90	PX91	PX92	PX93	PX94	PX95
47.96875	0.374756	1.67749	15.39063	1231287	23200397	68233.02	0.118431	31.125
47.19512	0.287775	1.507585	6.597561	3182389	17451499	664441.3	0.486662	26.59756
218.1362	0.362353	2.216038	20.37043	1045032	20489017	55950.88	0.082626	142.8439
13.48276	0.464923	0.972652	8.206897	30168.07	271425.4	3354.754	0.206418	7.344828
32.19643	0.287468	2.079082	20.44643	113179.9	2785402	4644.981	0.084803	31.46429
115.5734	0.394448	1.341705	15.77133	668627.9	10915900	41257.94	0.101119	60.71331
70.17051	0.323366	2.406974	21.81567	105492.4	2934490	3891.64	0.087518	68.69585
90.45704	0.310849	1.810276	19.56014	339576.3	8824773	13233.41	0.094907	72.94502
4.73913	0.206049	2.68431	15.08696	14164.22	319860.8	657.8297	0.215507	6.304348
286.7259	0.306987	3.011579	30.65846	611710.1	20395822	18800.84	0.058949	207.2056
768.7463	0.327265	2.718852	114.5164	5845315	6.89E+08	49853.86	0.010622	524.834
1103.113	0.341944	2.465467	19.80936	28997277	5.64E+08	1563356	0.089397	689.7973
134.2248	0.287419	2.694258	31.16916	1059995	36722246	31043.47	0.05547	117.5739
31.6	0.316	2.1056	19.08	91257.22	1837191	4761.768	0.091669	29.48
14.67442	0.341266	1.455922	14.11628	37033.91	584434.7	2372.503	0.126802	10.25581
635.6031	0.35628	9.371532	1813.43	4150176	7.67E+09	2247.447	0.003772	377.1424
16.62264	0.313635	1.52225	7.264151	157584.5	1495916	17090.09	0.38804	10.73585
138.8426	0.248377	2.779836	25.30411	1618443	51074045	52952.59	0.072719	131.6512
140.2907	0.407822	1.703894	35.91279	1013403	36126413	28546.26	0.043105	71.36628
600.154	0.280839	2.165768	57.76837	2695285	1.65E+08	44636.24	0.021284	522.4853
635.911	0.385167	1.676022	25.24349	8584917	1.97E+08	387613.3	0.056824	406.0588
302.6725	0.330428	2.386171	29.99454	949513.9	30381248	30600.89	0.050534	208.3297
22.65079	0.359536	1.360544	13.77778	56642.9	902704	3565.635	0.121662	12.77778
226.8695	0.344263	1.717611	22.74659	8300475	1.98E+08	354594.1	0.070534	115.264
210.6334	0.382275	1.523809	43.97459	2359275	1.16E+08	47894.23	0.028665	109.9982
32.15686	0.315263	1.949731	13.65686	116610.6	1850103	7403.874	0.175857	22.2549
68.54651	0.398526	2.093665	19.47093	1824678	29699841	112782.5	0.082038	32.06977
87	0.384956	1.442556	16.39823	1494788	22315942	104544.8	0.103702	45.69027
70.41423	0.29462	2.154269	21.86192	206557.6	5000462	8685.987	0.091325	46.87448
90.71348	0.254813	2.704417	18.9073	348702	7984598	15797.81	0.134164	82.15169
15.09524	0.239607	2.408667	17.90476	44521.79	1050217	1938.474	0.124312	17.25397
305.0406	0.325898	2.140491	33.3141	2294164	79768784	66865.48	0.04505	161.0641
11.35897	0.291256	44.76266	1765.949	181195.7	3.28E+08	114.8728	0.026181	13.66667
116.2471	0.268469	2.150676	28.67436	5230110	1.81E+08	153777.1	0.057463	76.11778
1457.273	0.410038	1.394994	26.4682	20407387	4.85E+08	879206.6	0.048582	1033.593
53.42029	0.387104	1.779878	43	153545.2	7525296	3139.491	0.035682	22.02899
590.7662	0.426238	1.409735	36.49784	4421510	1.56E+08	126795.7	0.033771	404.0216
40.54545	0.368595	1.452893	15.34545	79532.4	1275383	4968.326	0.117864	24.70909
88.78889	0.493272	1.001821	9.061111	131623.3	1184895	14628.47	0.178395	38.97778
7.5	0.234375	2.280273	18.53125	33327.94	901156.5	1268.001	0.113867	4.0625
125.1873	0.249377	3.192108	52.9761	490995.1	30872361	7849.576	0.028984	102.8964
964.1766	0.339858	1.666632	19.95982	7928931	2.02E+08	318240.3	0.071958	756.9789
50.72671	0.315073	1.783342	11.73913	412461.9	4927286	37397.86	0.176779	33.91925
290.8098	0.384161	1.531646	25.4214	4300462	1.05E+08	178655.7	0.055498	133.0053
327.3889	0.454707	1.129931	16.93056	1564660	24833763	99209.39	0.082626	155.1167
8.25	0.34375	1.817708	7.458333	385030	2542945	68407.93	0.4864	6
31.66667	0.340502	2.425483	34.17204	384793.7	13335087	11265.16	0.053752	19.27957
122.285	0.3168	1.516182	14.16839	1675138	24125214	123289.1	0.122296	79.07772
149.9827	0.370328	1.72642	16.50617	553917.8	8188103	39313.88	0.111164	91.22716
304.1317	0.408779	1.368104	16.40457	1502124	23564141	97146.89	0.099744	163.3011
396.8942	0.403758	1.395721	32.86063	2394951	86734879	66251.15	0.038877	193.7915
17	0.242857	2.371429	18.37143	67406.14	1479469	3213.008	0.109675	14.71429
51.26357	0.397392	1.565291	13.46512	266726.2	4876632	15156.5	0.128547	38.75194
99.62651	0.400106	1.335559	15.73896	499451.2	7942639	31554.02	0.102546	55.97189
216.4053	0.178258	8.05577	42.03871	2814113	1.04E+08	76722.29	0.052761	306.0725
122.9931	0.427059	1.221451	15.49306	1235164	19416303	79603.2	0.096625	70.85417
71.32353	0.262219	2.451071	28.86765	782745.8	27426130	22584.46	0.059955	57.75735

23.625	0.246094	2.731662	24.21875	124393.4	3709519	4315.096	0.081122	30.41667
26.0625	0.407227	1.308594	9.9375	196567.4	1654528	25028.37	0.233181	15.21875
45.06867	0.193428	3.601761	35.79399	399009.3	18106671	8969.257	0.050026	42.11588
499.4944	0.186518	2.972406	36.87976	31317624	1.01E+09	1011518	0.03909	468.1867
32.70968	0.263788	3.328499	70.33065	131074.5	10310104	1677.736	0.026212	26.27419
173.2231	0.345066	2.013655	44.73108	755125.9	36865242	15488.68	0.031165	109.745
467.1613	0.352043	1.706003	34.85305	2843774	98902272	82854.75	0.037181	318.9382
301.69	0.341278	1.715848	33.37557	1886491	67051123	53589.01	0.038547	287.0611
51.85517	0.357622	1.520285	14.93103	120756.7	1910409	7699.574	0.120791	29.28966
21.41379	0.369203	1.390012	9	65369.97	565565.9	7896.536	0.279933	17.17241
14.82222	0.329383	1.493333	7.911111	69422.22	570031.9	9235.91	0.359414	12.06667
413.4759	0.415136	1.932649	18.00502	1884876	35020178	105675.1	0.086439	323.0161
1273.179	0.397124	2.127845	33.67467	13308689	4.15E+08	441215.1	0.039931	1060.362
454.9803	0.344943	1.615175	35.49128	6592759	2.15E+08	207987.1	0.036235	363.6005
1204.502	0.365333	1.663337	34.68487	6791001	2.42E+08	191320.7	0.037285	808.0713
615.9357	0.455237	1.541294	25.44494	4695737	1.18E+08	188146.1	0.049917	310.969
17.68	0.3536	1.4176	8.6	23899.82	214250.4	2684.422	0.303472	16.04
23.33766	0.303087	1.320965	8.688312	1131002	15816940	86515.65	0.23939	21.67532
1039.019	0.48394	1.101608	50.51327	3634006	1.78E+08	74206.54	0.022327	523.1127
76.27358	0.359781	2.216358	13.0566	390915.9	4932773	33621.09	0.19105	47.82075
263.2792	0.301235	3.484628	37.42792	2651489	89467114	80306.05	0.04134	241.8535
48.28788	0.365817	1.774736	6.568182	1404542	9605777	240384.2	0.557292	47.60606
73.46078	0.360102	1.515066	15.79902	559429.3	9978784	32394.86	0.101592	66.88235
130.6276	0.300293	1.750276	39.90575	2349113	1.1E+08	50565.31	0.032567	108.4483
39.04688	0.305054	1.984314	22.30469	65823.96	1642940	2640.389	0.080509	28.64063
487.9884	0.355159	2.606673	28.29549	2549468	63495816	103424.7	0.050321	335.0437
1174.424	0.434168	1.196893	24.64325	17223945	4.29E+08	697253.1	0.050466	604.6895
244.8474	0.39748	2.475352	31.26136	328332.9	10470744	10620.38	0.047075	212.7305
70.82609	0.384924	1.667031	24.59239	495657.6	12252864	20176.15	0.063623	44.34783
92.85235	0.311585	1.684125	22.78859	612141	16709253	22926.04	0.066504	75.84564
12.5102	0.25531	2.77551	33.81633	43104.29	1756584	1082.372	0.061538	18.10204
305.0049	0.37516	1.791863	47.27552	969000.2	47284557	19887.7	0.028148	179.5781
294.2702	0.320556	1.947224	24.79521	4915517	1.34E+08	184839	0.065994	344.6187
216.9141	0.300435	11.24303	30.30194	1086156	9794163	120648.7	0.143702	188.3629
43.51351	0.29401	1.826333	20.87838	1136519	24324454	55692.76	0.075993	40.58108
76.8342	0.398105	1.363419	14.51813	13790329	2.61E+08	761376	0.107739	41.72539
28.20388	0.273824	2.903195	26.94175	97173.53	2380194	4011.586	0.076895	24.04854
372.1034	0.458255	1.346579	27.6564	3037458	75874479	121664.9	0.046099	195.6453
22.8806	0.341501	1.337937	9.044776	70508.43	622910.5	8159.508	0.272492	12.70149
19.43333	0.323889	1.595556	10.2	170297.8	1867703	16707.02	0.203861	10.46667
57.44344	0.259925	2.212649	18.17647	368321.4	6916215	20524.75	0.108358	45.85068
118.8571	0.339592	1.526727	15.88	1931277	29989174	127480.1	0.113536	57.18857
15.78571	0.281888	1.534439	26.17857	122939.9	5765833	2657.557	0.066209	14.82143
303.4023	0.350754	1.763004	24.79191	2759504	67991644	113075.7	0.060918	159.2913
128.3516	0.320079	1.962873	23.43142	485528.1	11315812	21483.57	0.072103	97.47382
58.89222	0.352648	1.639571	15.24551	373382.9	5799392	24555.41	0.128358	32.36527
59.36269	0.307579	3.455985	22.98964	318459.4	5001562	20559.65	0.082179	47.47668
88.85455	0.403884	1.414442	15.34091	1053087	17862607	63310.25	0.113456	61.16364
32.89744	0.421762	1.20069	12.39744	170566.9	2850036	10394.06	0.12459	14.66667
54.97753	0.308863	3.135084	44.73034	327172.2	17681601	6151.53	0.037837	42.95506
424.3427	0.350407	1.872779	35.69694	3272083	1.13E+08	95703.87	0.038199	201.8737
713.6324	0.39039	1.743229	25.69858	15914218	3.7E+08	705125	0.060733	352.1575
351.4715	0.334098	2.844984	27.93061	4411521	1.29E+08	155587.4	0.05518	333.1578
95.43346	0.362865	1.709003	22.80608	394364.2	9855127	15790.85	0.077571	35.59316
109.9421	0.424487	1.227143	15.83784	1141928	18262482	71483.75	0.092339	30.49807
514.6448	0.367079	2.569993	173.9708	6600118	1.2E+09	36617.33	0.006826	561.8873
126.6386	0.260038	3.007079	40.29979	495197.8	22503662	11114.3	0.040483	85.79671
269.9119	0.377499	2.107342	35.84476	2434780	84727340	70883.33	0.039943	113.7776

21.13333	0.234815	2.533333	18.53333	82008.13	1868394	3741.299	0.117319	22.68889
55.92199	0.39661	1.992556	11.7305	436459.6	5273141	39157.57	0.184247	34.31915
729.3401	0.254125	8.125122	76.53589	3783683	2.34E+08	61630.89	0.019444	715.846
71.59259	0.44193	1.187776	16.40741	570872.6	9047607	36288.81	0.092829	20.53086
155.302	0.275847	2.833312	46.79751	418810	19825187	8958.253	0.031927	151.7922
634.7189	0.450475	1.358156	35.26047	9511309	3.42E+08	264283.9	0.037843	200.1327
18.48837	0.429962	1.185506	10.32558	126025.9	1007812	17516.84	0.198805	11.55814
278.4102	0.364889	1.760911	24.20839	1264760	31490636	50939.1	0.068908	179.0865
22.93443	0.375974	1.531309	16.7541	184527.7	2716448	13172.28	0.107277	21.81967
49.74854	0.290927	1.78749	12.85965	1186984	17388688	85322.27	0.165117	34.32164
85.46104	0.277471	2.090867	20.4026	3076418	69931975	140505.9	0.088612	57.76623
76.42135	0.214667	3.215945	27.6573	172574	5973175	5087.274	0.074416	95.53371
119.6418	0.212131	5.378386	36.51241	2413006	92741316	64056.76	0.058795	93.52128
401.5855	0.411039	1.343463	24.21085	4360470	1.09E+08	174462.7	0.055397	142.4391
420.4413	0.301176	1.872483	21.33095	1597171	39320568	65578.83	0.079255	359.9398
63.44928	0.229889	2.435452	18.87319	649098.1	15034771	29018.58	0.121978	42.81884
383.1326	0.313529	2.873535	29.77414	778882.2	24996512	25021.19	0.056459	357.545
2436.761	0.451922	1.446526	17.86165	13439241	2.14E+08	846562.1	0.081144	1349.593
37.57471	0.431893	1.182191	9.505747	31984.2	290782.5	3550.133	0.191593	24.88506
42.53719	0.351547	1.632539	15.58678	1252984	18838782	86709.78	0.122952	25.94215
38.85263	0.408975	1.740055	9.757895	383180.4	4207624	37311.59	0.219953	17.04211
152.2039	0.373965	1.514479	15.49877	1096488	18564526	66044.03	0.098645	78.01229
33.80702	0.296553	1.297938	10.40351	954854.5	10204309	94974.83	0.200115	19.64912
60.98077	0.586354	1.228458	7.721154	96279.78	1076110	9244.239	0.219527	28.67308
362.4901	0.421991	1.1891	10.03609	10778451	94022138	1288345	0.193362	122.0477
122.1586	0.421237	1.472117	10.36897	877803.3	7029715	121729.8	0.261806	62.68966
80.77273	0.367149	1.445124	10.55455	421295.6	4221394	43827.72	0.169179	68.72727
237.7721	0.244119	2.709115	39.60164	1049235	51083669	21627.03	0.036815	214.4251
40.1028	0.374793	1.465281	14.68224	58682.67	933011.4	3714.337	0.129587	28.83178
37.63636	0.244392	4.484061	37.28571	88696.36	2939660	2747.841	0.068488	42.1039
10.03226	0.323621	2.528616	13.35484	8354.871	108837.4	696.1709	0.242133	5.709677
19.2973	0.260774	1.521549	15.43243	238294.5	7092348	8313.061	0.109318	19.64865
236.854	0.308806	2.25242	32.35202	1032962	36119244	29883.36	0.045819	155.8996
442.8203	0.348952	1.864572	23.88574	1405430	34923109	56807.7	0.074483	304.7368
40.30719	0.263446	2.221453	25.11111	79681.62	2051225	3136.506	0.066558	44.84314
12.07692	0.309665	1.050625	8.025641	34289.97	498170.7	2513.894	0.259843	8.128205
23	0.247312	2.388484	19.17204	250328.5	5796340	11226.97	0.129818	23.43011
70.11877	0.268654	2.883457	30.75862	804580.5	26538085	25170.66	0.08511	66.1341
197.0783	0.385672	1.829979	47.72994	1346624	66090202	27450.16	0.028461	102.2603
149.6334	0.240568	3.260494	38.43569	741212.2	33727832	16615.2	0.042411	103.0643
54.46512	0.316658	1.749155	17.98256	650370.6	15500230	27929.66	0.10771	52.26744
22.8835	0.22217	3.969083	38.24272	10991.83	507919	244.3481	0.079072	26.57282
66.57823	0.226457	2.59009	26.01701	114008.9	4254781	3135.921	0.068462	78.31973
8.244444	0.18321	2.782222	17.73333	6093.556	159358.4	265.2952	0.127092	9.044444
65.48529	0.48151	1.085424	18.16176	505476.1	12869784	20007.34	0.090968	27.52941
41.18919	0.185537	2.96786	25.59459	179867.9	6049956	5499.096	0.077911	45.68468
71.39521	0.213758	3.965372	33.17066	51194.15	2306010	1161.365	0.061624	85.4491
293.9127	0.413962	1.358492	23.59437	2980267	74777774	118972	0.059113	154.1239
44.95522	0.335487	1.468089	24.35075	328753.2	11652126	9342.555	0.061438	40.68657
396.882	0.377623	1.582611	46.5785	3275538	1.59E+08	67861.24	0.026158	252.3911
84.52511	0.385959	1.727946	13.70776	284855	5313304	15918.01	0.125283	57.7032
41.6087	0.201008	4.568135	39.58937	99084	4406026	2282.136	0.054699	57.82126
6.923077	0.266272	1.556213	8	7071.192	78586.58	695.9411	0.326998	7.307692
194.0909	0.383579	2.50787	16.44466	175353.4	2426202	13606.26	0.149704	122.4743
202.1881	0.301773	3.087235	29.29552	164103.3	5261712	5279.859	0.063679	171.8418
43.61818	0.396529	1.206942	15.50909	20843.45	330673.7	1323.125	0.097788	23.52727
191.0718	0.263911	1.4512	30.85635	5186402	1.67E+08	165636.9	0.042944	217.232
79.4526	0.242974	3.83634	40.31498	73863.29	3468149	1595.205	0.052372	85.88685

264.1363	0.288044	2.420336	29.39913	842645.8	30274237	23534.35	0.061289	204.1887
54.02105	0.284321	1.901634	13.93158	444635.5	6920994	29687.87	0.149759	41.65263
54.72093	0.181797	3.645368	23.16944	55836.71	1947027	1646.009	0.106944	81.88372
38.48344	0.254857	1.992983	26.7947	325482.9	11433958	9361.097	0.06073	39.58278
186.0999	0.218684	2.7879	32.85076	534969.9	19735109	14779.19	0.049875	227.9777
53.49242	0.202623	4.040806	35.04545	32668.28	1521264	715.7259	0.055317	61.65152
116.1013	0.301562	2.252076	22.0026	1056356	26327417	42536.18	0.096718	60.83896
67.02228	0.186692	3.546062	29.95822	175346.9	6627049	4747.397	0.062798	118.3538
1582.949	0.128999	27.99361	103.4389	8886888	4.37E+08	182412.6	0.025263	3523.771
128.6968	0.232305	3.251134	26.21841	242479.8	8806352	6709.9	0.106564	110.6173
35.23	0.17615	3.823975	27.785	63140.95	2347268	1726.734	0.105778	35.02
1107.742	0.279874	3.156105	56.58489	13495452	8.51E+08	214905.3	0.026417	717.8706
224.6064	0.300678	2.247268	31.3668	1902147	67916355	53497.57	0.053978	131.2008
352.3373	0.261184	3.033406	24.58117	1672920	51025259	56700.25	0.079336	436.3595
21.03509	0.369037	1.791936	16.82456	15413.25	240351.7	1007.383	0.144742	17.5614
83.21898	0.303719	2.686624	22.60949	275998.6	6561495	11897.44	0.118086	75.29927
188.9569	0.21448	3.105106	19.52554	2151262	63848529	75001.25	0.133415	248.2168
244.7451	0.264304	3.33275	29.70086	409696.5	13989850	12228.15	0.074071	228.6479
43.17105	0.28402	2.918932	30.96711	64197.82	2267710	1835.599	0.075033	38.82895
30.2782	0.227656	2.724744	34.60902	163685.5	7579254	3604.666	0.050468	32.14286
116.4859	0.29945	1.680441	20.86889	2004681	47295799	87456.14	0.078377	68.43445
14.31429	0.40898	1.048163	8.571429	9455.257	81849	1142.175	0.215278	8.657143
569.1565	0.428259	1.44027	26.81791	2791167	68350039	115283.2	0.052351	299.7705
221.7207	0.332914	1.945037	33.63213	825332.5	29157779	23543.32	0.041788	159.6426
8.869565	0.192817	2.835539	16.17391	1151.478	36608.8	44.3491	0.168891	14.86957
147.0394	0.290019	3.234022	31.61538	405872.9	13655974	12339.42	0.064029	148.1598
80.77324	0.183159	6.193387	81.81633	6593723	6.13E+08	71605.79	0.023619	120.0839
153.8974	0.367297	2.120665	19.74463	572427.9	11395789	30187.03	0.085125	109.0621
183.8729	0.338624	2.080814	30.36832	228258.8	8641163	6107.333	0.050459	129.3867
8.142857	0.232653	2.444082	16.02857	21506.03	612743.9	806.1284	0.13236	6.485714
132.5024	0.211328	4.476108	32.21053	93163.85	4193063	2115.299	0.082262	192.3078
57.128	0.228512	2.045056	12.856	372328.5	12342077	11531.86	0.166384	47.184
36.84694	0.187995	4.073589	32.06633	174987.2	7703203	4066.75	0.06644	53.90816
60.1082	0.197076	2.606074	13.79016	190101	5058488	7332.762	0.214548	56.87541
3.352941	0.197232	2.719723	14.76471	2368.824	55304	141.3148	0.251127	3
85.23864	0.48431	0.933077	8.278409	435651.5	3919866	48435.99	0.193063	32.27273
10.09091	0.458678	0.952479	4.772727	35936.77	142760.2	9233.698	0.561869	5.727273
144.2444	0.400679	1.306667	15.49444	2717797	42442526	177102.1	0.097856	66.63889
888.5228	0.465927	1.104705	10.02622	6709760	60105028	753390.3	0.180785	369.646
107.4132	0.443856	1.091592	13.08264	581763.5	9513130	35853.03	0.112078	61.15702
137.1184	0.300698	1.756343	12.76535	2706173	40409469	189209.6	0.168257	106.7325
49.41772	0.31277	1.878064	13.64557	412786.4	6214497	28509.56	0.161387	42.27848
44.61481	0.33048	2.42941	21.65926	156297.4	3596904	7029.181	0.095076	52.88148
62.31361	0.36872	1.453941	14.10059	240696.3	3817546	15290.18	0.133131	34.21893
202.1882	0.352244	2.430817	22.85366	625938.1	15142224	26303.92	0.081032	143.1254
1060.432	0.293748	3.150013	41.95152	2228137	1E+08	50500.57	0.033727	1127.606
212.4152	0.414065	1.300191	15.27875	5100547	81888755	318277.4	0.105719	54.41131
19.5	0.1875	2.823595	19.28846	107624.1	3065643	3899.207	0.125964	19.25
33.69388	0.343815	1.579758	15.22449	372151.4	5581407	25850.11	0.115607	26.46939
29.30769	0.322063	2.096124	14.38462	48321.48	716854.7	3412.786	0.175302	20.12088
291.8814	0.329809	1.91546	31.12429	6893733	2.1E+08	234341.3	0.042869	337.6554
21.59701	0.322344	1.285364	12.86567	23614.87	372055.9	1523.142	0.128259	15.0597
378.7909	0.291154	2.326307	39.30131	1722164	85665714	34763.71	0.038302	313.3059
15.21429	0.543367	0.809949	6.535714	43526.5	174848.1	10863.73	0.37004	4.285714
87.92444	0.390775	1.550736	34.32	126067	4538401	3505.529	0.040432	36.41333
27.51579	0.14482	4.691856	25.08421	155922.8	7613842	3400.556	0.173809	42.15789

216.6203	0.239095	13.56661	786.9713	1747175	1.46E+09	2090.225	0.002892	269.1898
553.8473	0.212528	3.729936	39.12011	8849183	4.08E+08	195218	0.060389	641.4712
1110.499	0.413594	1.394923	47.32142	8783113	4.33E+08	178630.4	0.02471	603.2585
668.3162	0.285972	43.79032	107.7356	1980286	71060559	55267.22	0.031467	607.6573
195.549	0.341869	2.086859	24.9493	1561432	39355504	62112.65	0.063772	148.7867
45.83333	0.293803	2.460881	27.71795	366957.9	14044042	9727.563	0.078866	34.21795
71.38462	0.343195	1.45821	13.30769	452801.1	7176769	28775.33	0.137727	47.59615
93.80365	0.428327	1.193261	9.105023	3945650	33735993	487708.7	0.219195	72.27854
53.05455	0.482314	0.981818	4.981818	169607.3	676356.2	42937.82	0.55	17.41818
45.78947	0.240997	2.79338	19.08947	88069.34	2956831	2689.899	0.102818	47.01053
41.39416	0.302147	2.038574	21.54015	126475.9	3140225	5127.463	0.082105	29.17518
72.15179	0.322106	1.916912	27.77232	1313534	50160590	34860.05	0.058929	44.52679
8.055556	0.223765	2.841821	26.47222	28062.89	1009128	783.4498	0.088686	4.555556
65.40936	0.382511	1.394275	9.672515	156655.4	1399500	17708.48	0.252498	40.7193
305.5526	0.458788	1.165023	16.84234	1596151	25538746	99760.27	0.086841	124.964
30.62069	0.527943	1.074911	7.586207	49564.17	445976.9	5529.431	0.223989	9
63.56522	0.460618	1.149758	22.92754	604636.2	15115129	24189.37	0.059507	19.62319
517.0092	0.339245	2.022404	34.00722	4190536	1.45E+08	122657.6	0.041573	413.9514
250.9541	0.303451	2.695233	32.43531	2111837	73624976	61337.89	0.054729	137.4813
305.3873	0.358436	1.757526	22.74296	742069.5	18531393	29736.63	0.075384	185.9014
194.9491	0.309935	2.500732	32.5151	639845.4	22646336	18212.69	0.053711	163.3784
390.9287	0.262898	2.48716	33.26093	3329273	1.11E+08	102093.2	0.047941	389.23
399.7851	0.401391	1.313361	15.89257	1490261	23832176	93273.3	0.101165	213.6526
6.555556	0.364198	1.027778	5.722222	889.1111	3789.444	222.3322	0.469522	4.555556
87.78893	0.303768	2.208403	32.55363	95790.27	3397778	2719.977	0.047576	58.38408
512.3593	0.434203	1.303421	34.64915	1881285	67762312	52243.78	0.035246	240.2627
678.4696	0.352086	2.932043	46.28542	2265662	1.1E+08	46965.14	0.043168	490.7001
124.8142	0.181153	4.958753	41.25544	338262.2	21516046	5862.456	0.068156	146.5704
13.12308	0.201893	3.217515	26.55385	15028.28	523908.7	437.2288	0.093159	12.44615
7.772727	0.176653	5.830579	32.68182	8694.864	400633.1	247.8947	0.146029	8.636364
162.595	0.339447	3.041435	43.7453	769642.8	37664143	15757.92	0.053695	79.98539
20.87879	0.126538	6.002718	61.21212	35636.59	3147495	409.6315	0.034738	36.72121

PX96	PX97	PX98	PX99	PX100	PX101	PX102	PX103	PX104
0.243164	0.492577	8.398314	0.05829	4.155701	0.007065	1211253	92.54776	0.00031
0.16218	0.369043	2.676209	0.176507	4.783935	0.00509	3143791	444.4046	0.000238
0.237282	0.488309	9.966663	0.043196	4.510641	0.015541	1040891	147.2326	0.000163
0.25327	0.486117	3.593894	0.120003	3.288678	0.028487	28935.82	3.385395	0.017806
0.280931	0.540129	10.47976	0.052678	4.191945	0.023179	111318.6	11.48277	0.001732
0.207213	0.450474	7.162019	0.047423	4.561524	0.016044	664743.1	54.47035	0.000513
0.316571	0.581556	13.45717	0.056112	4.348022	0.029344	104331.1	18.3657	0.000888
0.25067	0.514154	9.766232	0.057482	4.784898	0.019956	337065.3	36.35893	0.000536
0.274102	0.521398	8.752383	0.162403	3.708132	0.025989	12683.64	4.05738	0.006867
0.221848	0.466057	13.43132	0.031746	4.900441	0.025184	610133.4	63.1643	0.000179
0.223429	0.478124	54.21764	0.00504	5.033504	0.013838	5840093	90.75716	4.66E-05
0.213824	0.460158	9.307874	0.040842	5.043184	0.007061	28977220	1013.876	1.43E-05
0.251764	0.511329	15.85735	0.030712	4.711664	0.015218	1055677	61.9861	0.000226
0.2948	0.553514	11.09974	0.054466	4.044655	0.021911	89174.21	22.13916	0.001316
0.238507	0.485093	7.276073	0.052976	3.783725	0.026909	35652.83	6.177707	0.006041
0.211403	0.463435	838.7549	0.00151	4.839805	0.016317	4146420	13.86686	8.96E-05
0.202563	0.445689	3.540012	0.204323	4.303695	0.012955	151626.4	24.37792	0.002098
0.235512	0.492947	13.07528	0.036296	5.07661	0.012187	1611710	129.7306	0.000143
0.20746	0.440085	15.87503	0.017452	4.36031	0.014717	1008786	40.00825	0.000388
0.244495	0.502604	28.71892	0.010644	4.95966	0.019073	2692536	104.5568	7.71E-05
0.245947	0.504808	12.12334	0.030777	4.465844	0.009719	8574331	506.7951	4.05E-05
0.227434	0.476832	14.10121	0.023789	4.708228	0.020114	947042.2	102.2039	0.00015
0.202822	0.446213	5.016191	0.072773	4.204591	0.026889	55259.77	8.68867	0.004198
0.174907	0.410513	9.713982	0.030756	4.989656	0.006611	8277595	303.4736	7.93E-05
0.199634	0.445331	19.57032	0.013661	4.700084	0.011917	2352234	45.643	0.000212
0.218185	0.471648	6.236647	0.090483	4.402245	0.022855	114696.1	9.543187	0.002149
0.186452	0.425078	7.758327	0.037732	4.563665	0.00763	1807499	39.96973	0.000355
0.202169	0.442998	6.773193	0.051401	4.492196	0.008911	1482193	133.6603	0.0003
0.196128	0.437153	9.050482	0.042676	4.791913	0.023824	204795.7	29.28358	0.000873
0.230763	0.486034	9.113153	0.074236	4.91724	0.020819	346394.8	65.13027	0.000424
0.273873	0.521012	9.442954	0.080937	4.051923	0.026504	43098.23	9.036681	0.002801
0.172077	0.405905	13.35246	0.017977	5.15316	0.014367	2289319	114.3225	0.000123
0.350427	0.597609	1077.681	0.000377	3.527237	0.01007	171333.6	2.832892	0.001399
0.175792	0.404228	11.02035	0.023055	5.207694	0.006697	5207816	110.7723	0.000108
0.290825	0.552918	14.91433	0.026856	4.120206	0.009739	20396843	474.3686	1.96E-05
0.15963	0.388877	17.38943	0.017215	4.637475	0.021746	151430.5	8.709935	0.001351
0.291502	0.554017	20.43329	0.018419	4.007896	0.013505	4416027	81.92527	8.48E-05
0.224628	0.475978	6.635863	0.066513	4.286776	0.029778	78404.66	8.940811	0.003112
0.216543	0.441923	4.19311	0.077466	3.960505	0.031061	130586.8	29.45354	0.002217
0.126953	0.332669	6.529602	0.034684	4.324699	0.018412	30378.09	5.291806	0.004021
0.204973	0.455835	24.05973	0.011765	5.215777	0.022523	489023.9	24.83472	0.000367
0.266824	0.524851	11.10113	0.03731	4.536347	0.012286	7922306	394.3509	3.22E-05
0.210679	0.451504	4.868224	0.092337	4.500294	0.01321	406731.1	89.9311	0.000551
0.175701	0.402095	10.3632	0.021056	4.863825	0.010036	4290535	147.6261	0.000111
0.21544	0.466406	7.582387	0.039939	4.360049	0.016615	1561037	172.6068	0.00022
0.25	0.464919	4.315614	0.189094	3.454987	0.005513	352133.2	79.59919	0.001149
0.207307	0.448248	15.04987	0.030393	4.273085	0.011412	377115.8	19.44133	0.000848
0.204865	0.442358	6.452421	0.053292	4.596431	0.01073	1666453	189.6764	0.000219
0.225252	0.463824	7.077053	0.059538	4.31138	0.018565	551016.5	103.4194	0.000313
0.219491	0.463012	7.567384	0.051255	4.313977	0.017202	1498744	188.2682	0.0002
0.197143	0.440545	14.14551	0.018665	4.734388	0.015242	2390647	93.0968	0.000141
0.210204	0.458291	8.552544	0.060177	4.607805	0.021021	65143.1	15.72186	0.001766
0.300403	0.56344	7.838828	0.076702	4.030554	0.014944	262248.6	40.98506	0.000711
0.224787	0.472934	7.734424	0.049936	4.23302	0.017898	496329.5	51.35077	0.000709
0.252119	0.510625	23.96216	0.029194	5.687035	0.014024	2809028	40.04969	9.85E-05
0.246021	0.498617	7.722743	0.050557	4.020428	0.012236	1228484	100.526	0.000362
0.212343	0.45116	12.61083	0.033448	4.808678	0.013819	777509.3	42.38298	0.000387

0.31684	0.581097	15.61489	0.049204	4.245416	0.017765	121224.7	17.61969	0.001116
0.237793	0.496383	4.639311	0.13329	3.905722	0.013414	191010.2	53.58524	0.001393
0.180755	0.414415	14.42738	0.019313	5.485218	0.01546	394825.5	14.27126	0.000493
0.174827	0.408423	14.6276	0.016591	5.990537	0.005094	31279090	461.9884	1.86E-05
0.211889	0.452941	30.8003	0.017508	4.722705	0.021038	128815.2	6.496068	0.001245
0.218616	0.46221	20.78996	0.014433	4.573398	0.020321	752704.3	28.72348	0.000396
0.240345	0.493755	17.31445	0.017786	4.568853	0.015733	2839733	132.7793	8.97E-05
0.32473	0.586865	19.72248	0.023416	4.122384	0.01518	1882151	60.83383	0.000138
0.201998	0.441345	6.125864	0.068108	4.308268	0.026392	119321.1	23.91564	0.001592
0.296076	0.552951	5.007374	0.173019	3.626844	0.022674	63424.85	26.01163	0.002755
0.268148	0.461451	3.387488	0.187737	3.59221	0.01861	66534.95	29.5064	0.002581
0.324313	0.589281	10.97183	0.051512	3.982764	0.015526	1880728	287.725	0.000102
0.330743	0.591809	19.65559	0.024218	3.858659	0.01068	13299922	472.8108	1.90E-05
0.275664	0.534386	18.68362	0.019241	4.377537	0.009824	6582397	253.1305	5.70E-05
0.245093	0.501936	17.2503	0.019252	4.625549	0.016395	6787281	303.6315	4.48E-05
0.229837	0.479208	12.56167	0.023569	4.252271	0.013105	4689914	79.72514	8.76E-05
0.3208	0.581465	4.819074	0.195979	3.693102	0.03647	23147.96	7.844841	0.008968
0.281498	0.523798	4.128831	0.146877	3.9771	0.005886	1102133	98.19279	0.000426
0.243648	0.500008	24.96315	0.011111	4.051565	0.020626	3631655	82.29367	0.000115
0.22557	0.481816	6.529501	0.094333	4.426926	0.015389	386693.4	109.876	0.000445
0.27672	0.538518	19.54419	0.024798	4.706073	0.012785	2645372	58.42825	9.31E-05
0.360652	0.607545	4.108113	0.346529	3.5135	0.006723	1382420	335.6579	0.000267
0.327855	0.583712	9.540134	0.061445	3.939857	0.013746	554136.7	68.88672	0.000405
0.249306	0.512146	21.38713	0.016858	4.69455	0.009836	2338776	54.8352	0.000164
0.223755	0.473496	10.05502	0.045602	4.493843	0.035001	65007.7	5.052051	0.003159
0.243845	0.501475	14.74802	0.025595	4.692977	0.016482	2545786	64.97083	0.000103
0.223545	0.476207	11.54566	0.024648	4.430988	0.009333	17212465	644.6563	2.99E-05
0.345342	0.608009	18.39374	0.030888	3.811779	0.028223	327077.5	57.3663	0.000266
0.241021	0.499577	12.41843	0.033815	4.194224	0.015378	491429	30.20243	0.000762
0.254516	0.512952	11.76409	0.038122	4.58989	0.015263	607848.5	50.85646	0.000366
0.369429	0.62744	23.39284	0.041409	3.932704	0.021993	41036.82	4.912593	0.00279
0.220883	0.472588	22.7695	0.013271	4.662509	0.022626	967046.8	35.74015	0.000276
0.375402	0.635176	15.29531	0.039824	4.112876	0.009885	4905284	257.3947	7.10E-05
0.26089	0.52174	17.27771	0.073448	5.010419	0.021113	1083913	27.11884	0.000312
0.274196	0.526185	11.42605	0.035948	4.190722	0.007783	1120008	85.49402	0.000316
0.216194	0.465541	6.881416	0.051816	4.566398	0.002647	13647626	364.0726	7.75E-05
0.233481	0.485213	14.27476	0.028457	4.392074	0.024373	95490.14	6.757985	0.001874
0.240943	0.495741	13.41988	0.024034	4.190166	0.014312	3032576	43.37323	0.00024
0.189575	0.404309	3.672771	0.131052	4.202732	0.023937	68763.19	21.10603	0.003318
0.174444	0.410391	4.968872	0.098699	4.499851	0.012225	163606.6	35.58651	0.001496
0.207469	0.452964	8.039599	0.05495	4.912895	0.015597	364210.9	64.46507	0.000479
0.163396	0.385979	6.411767	0.043489	5.034641	0.009632	1920498	134.4845	0.000231
0.264668	0.52483	14.21053	0.039448	4.086455	0.01544	118745.1	6.802677	0.001761
0.184152	0.418457	10.18683	0.027247	4.998824	0.012732	2753335	158.4322	0.000126
0.243077	0.504906	11.65493	0.037786	4.739272	0.019243	482827.5	59.1307	0.000358
0.193804	0.432362	6.38302	0.063637	4.510129	0.0159	369427.5	49.73309	0.000782
0.245993	0.505408	13.56796	0.037045	4.568135	0.018765	315619.6	16.51079	0.000766
0.278017	0.543332	8.219656	0.069062	4.139891	0.010629	1044235	81.48912	0.000343
0.188034	0.437805	4.769304	0.068072	4.421541	0.014703	165941.2	20.2726	0.001479
0.241321	0.493232	25.52077	0.014157	4.522268	0.015621	323074.1	17.28517	0.000574
0.1667	0.39727	13.73186	0.016421	5.185606	0.013474	3266575	136.27	8.86E-05
0.192646	0.432079	11.11802	0.026319	4.761563	0.007693	15897319	711.6433	2.83E-05
0.31669	0.57509	15.57432	0.032529	4.326729	0.01062	4402655	140.081	5.53E-05
0.135335	0.338542	7.364424	0.033383	5.101563	0.020351	391949.8	28.28256	0.000809
0.117753	0.301676	4.788216	0.027326	5.049466	0.012392	1135416	49.77796	0.000558
0.400776	0.654312	114.697	0.004706	3.709446	0.010087	6590289	88.0523	3.86E-05
0.176174	0.406118	16.58602	0.015594	5.409734	0.019222	492491.4	37.46578	0.000302
0.15913	0.384208	13.27067	0.018235	5.095986	0.012424	2428302	107.0391	0.000129

0.252099	0.509607	9.272822	0.061122	4.459986	0.021908	79924.72	15.13591	0.001442
0.243398	0.49831	6.326625	0.091817	4.108558	0.012227	429770.4	98.58589	0.000497
0.249424	0.506401	40.1514	0.009843	5.394766	0.019369	3781017	43.22481	5.16E-05
0.126734	0.331123	5.533847	0.033647	4.750825	0.013268	565191.9	49.89353	0.000746
0.269613	0.523294	24.73474	0.017218	4.761688	0.023394	416982.7	33.91767	0.000318
0.142039	0.353644	12.27457	0.014726	5.021531	0.010567	9502354	148.2603	0.0001
0.268794	0.503424	5.998224	0.072796	3.313828	0.013317	120386.9	41.38459	0.001893
0.234714	0.482475	11.73648	0.034411	4.554099	0.019245	1262060	62.92737	0.000255
0.3577	0.614716	10.66711	0.067817	3.542904	0.013215	178801.4	32.30326	0.001245
0.200711	0.436575	5.49338	0.082624	4.628816	0.008658	1173643	116.3211	0.00035
0.187553	0.422265	8.726224	0.04093	4.958199	0.007056	3056330	164.5045	0.000155
0.268353	0.534286	15.35267	0.03477	4.976994	0.027066	171208.9	27.54834	0.00058
0.165818	0.38899	13.37613	0.022298	5.860052	0.009687	2402350	41.8021	0.000132
0.145792	0.358816	8.339807	0.020793	5.041702	0.012594	4354165	113.0198	0.000161
0.257837	0.51659	10.80243	0.045123	4.697234	0.020386	1594765	131.9718	0.000129
0.155141	0.372818	7.555508	0.036632	5.368517	0.013167	643330.4	56.32553	0.000395
0.29259	0.556234	16.34823	0.033526	4.550277	0.025178	777304.7	121.8523	0.000127
0.250296	0.506544	9.237748	0.040934	4.176848	0.016708	13435659	247.8167	3.54E-05
0.286035	0.55032	4.552653	0.127952	3.685246	0.037843	31285.9	13.01265	0.003865
0.214398	0.447994	6.801563	0.058106	4.136678	0.007379	1234618	91.66956	0.000425
0.179391	0.394273	4.467681	0.084632	4.432681	0.010881	374733.8	64.15206	0.000787
0.191676	0.433764	6.849796	0.043012	4.801276	0.013498	1090999	114.0803	0.000256
0.172361	0.395536	4.466892	0.067834	4.752638	0.007568	937395.8	103.481	0.000479
0.275703	0.536006	4.921267	0.11092	3.477467	0.022236	94257.37	36.38522	0.001411
0.142081	0.360412	3.436228	0.079948	5.088317	0.006511	10754865	626.3123	6.57E-05
0.216171	0.466081	4.766865	0.133812	4.203065	0.012801	871700.4	271.5532	0.000298
0.312397	0.572638	6.160943	0.099959	3.890904	0.016724	417720.1	81.39064	0.000588
0.220149	0.468915	18.96173	0.017011	5.148064	0.021951	1047159	44.17398	0.000205
0.269456	0.513003	7.121117	0.077427	3.771103	0.033479	57790.51	12.54411	0.003037
0.273402	0.5323	20.08584	0.044767	4.768871	0.027175	87342.21	7.910418	0.001045
0.184183	0.372101	5.828006	0.107452	3.719852	0.038509	7680.547	6.771175	0.007436
0.265522	0.528931	7.910311	0.065315	4.408585	0.01091	229892.6	22.9961	0.000879
0.203259	0.442173	14.43404	0.020118	4.921405	0.019384	1030301	88.59592	0.00019
0.240139	0.496087	11.67641	0.041647	4.623028	0.021509	1403268	137.1234	0.000145
0.293092	0.546372	13.74074	0.04212	4.227357	0.027697	78378.08	14.42753	0.001428
0.208416	0.417457	3.472347	0.094458	4.169767	0.022273	32274.19	12.91036	0.003433
0.251937	0.480728	10.36818	0.057996	4.367425	0.012936	244353.1	28.80924	0.000927
0.253387	0.503259	15.63407	0.028643	4.637271	0.012134	797789.1	62.22567	0.000286
0.200118	0.443755	21.0078	0.013483	4.744713	0.016065	1342749	32.30072	0.000339
0.165698	0.388255	15.38797	0.015781	5.532714	0.01743	737920.5	55.37245	0.00021
0.30388	0.565564	11.4646	0.05989	4.084407	0.011721	643091.1	59.59478	0.000413
0.257989	0.513037	21.75442	0.033836	4.503215	0.055287	10664.68	3.950808	0.003658
0.266394	0.522686	13.68972	0.038179	4.926346	0.029438	112855	23.58153	0.000656
0.200988	0.436437	7.726358	0.064641	4.711422	0.037375	5377.696	6.260594	0.004609
0.202422	0.440371	8.191448	0.035686	4.100275	0.012584	499161.7	29.86228	0.000765
0.205787	0.452171	11.90075	0.037527	5.347266	0.019943	177353.4	25.54912	0.000652
0.255836	0.516543	16.54105	0.036301	5.073321	0.04561	50713.43	13.65707	0.000891
0.217076	0.469228	11.34529	0.027149	4.447559	0.012532	2973900	115.3652	0.000192
0.303631	0.568217	13.63391	0.03719	4.049505	0.01549	324585.2	17.6918	0.00091
0.240144	0.492838	23.88301	0.012106	4.376827	0.014195	3270575	95.45714	0.000118
0.263485	0.523426	8.152142	0.06007	4.330965	0.018458	281919.7	60.01051	0.000497
0.27933	0.540015	21.4891	0.034366	4.895732	0.027169	97729.26	12.39303	0.000842
0.281065	0.541035	4.071364	0.219494	3.792377	0.035961	6297.925	6.891188	0.008412
0.242044	0.496709	8.000311	0.082797	4.42172	0.033272	174450.1	56.28737	0.000421
0.25648	0.512578	14.88073	0.032623	4.713159	0.038242	163419.5	46.59641	0.000351
0.213884	0.44363	6.85938	0.047827	4.236201	0.052306	20477.94	9.684619	0.004125
0.300044	0.56283	18.11431	0.024397	4.518844	0.006987	5165921	257.8986	6.78E-05
0.262651	0.526693	21.63481	0.030465	4.966889	0.040157	73243.17	12.7559	0.000911

0.22267	0.470439	13.61857	0.031926	4.994194	0.022802	840722.4	63.08722	0.000224
0.219224	0.479213	6.740407	0.071025	4.834726	0.013273	438959.1	58.90759	0.000578
0.272039	0.538643	13.30014	0.057872	5.134171	0.038924	55176.69	18.09331	0.000977
0.262138	0.516069	15.26239	0.027551	4.512205	0.015319	321221.6	21.65387	0.00077
0.267894	0.52487	17.95281	0.024874	4.960388	0.024171	533258.2	59.75344	0.000211
0.233528	0.489275	17.38564	0.029982	5.079855	0.047817	32230.94	10.67195	0.001198
0.158023	0.384363	8.73634	0.036438	5.251378	0.014043	1051285	69.23117	0.000355

0.329676	0.59074	18.47366	0.038328	4.745843	0.026461	173918.8	28.05966	0.000544
0.287162	0.550208	67.04642	0.013467	6.203259	0.023931	8885142	82.82934	1.55E-05
0.19967	0.436007	12.09594	0.056153	5.316297	0.030105	241376.4	33.51562	0.000454
0.1751	0.405474	12.47404	0.036596	5.473938	0.031447	62129.71	11.8392	0.001141

0.181372	0.411982	23.31464	0.01145	5.498416	0.012554	13489107	201.4841	3.15E-05
0.175637	0.405231	12.83569	0.022724	5.249772	0.014487	1897382	84.77124	0.000166
0.323469	0.587449	15.03303	0.046538	4.618559	0.018264	1669922	194.1941	8.26E-05
0.308095	0.555013	9.332211	0.107281	3.544044	0.045131	14922.27	5.143406	0.005888
0.274815	0.527823	13.17436	0.05517	4.312105	0.021477	273830.6	42.88871	0.000489
0.281744	0.546983	11.64682	0.072999	5.107085	0.012703	2145065	251.9134	8.11E-05
0.24692	0.506289	14.81276	0.041943	5.068868	0.029836	408573.1	65.80135	0.000232
0.255454	0.517238	16.0897	0.042622	4.560164	0.033051	63282.36	7.658981	0.002305
0.241676	0.492726	18.38511	0.025395	4.58719	0.018938	160897.2	14.80673	0.000872
0.175924	0.406783	8.852686	0.033035	5.093607	0.00911	1992633	113.8683	0.000185
0.247347	0.495757	4.633514	0.086222	3.475732	0.041274	8868.233	10.00227	0.007869
0.225561	0.474614	12.72903	0.024867	4.450037	0.016119	2787318	196.2093	9.95E-05
0.239704	0.488009	16.28297	0.020296	4.554364	0.02129	823126.3	53.87018	0.000255
0.323251	0.571521	9.307182	0.125087	4.188216	0.087452	1020.724	3.099984	0.009128
0.292228	0.549803	17.6641	0.038028	4.466555	0.023682	404089.8	44.81735	0.000285
0.272299	0.532685	44.56156	0.013772	5.376258	0.005665	6562564	48.68257	7.66E-05
0.260291	0.515582	10.77646	0.045073	4.217357	0.017963	569328.7	111.3234	0.000263
0.238281	0.492646	16.13233	0.027631	4.721717	0.030412	227177.6	34.95554	0.000409
0.185306	0.350634	5.314635	0.065543	4.433154	0.022054	19450.05	5.546368	0.004178
0.306711	0.572519	18.35446	0.052147	4.969883	0.046798	92707.24	26.02363	0.000466
0.188736	0.436733	5.588335	0.093529	5.261801	0.016444	368630.4	44.52207	0.00042
0.275042	0.539339	19.68433	0.031557	4.808256	0.021773	172877.7	21.86611	0.000609
0.186477	0.432095	6.39726	0.11147	5.482752	0.024384	188419.2	38.33075	0.000564
0.176471	0.402454	8.443979	0.080607	3.807764	0.054839	2036.298	2.47977	0.012208
0.183368	0.427071	3.609628	0.080604	4.373344	0.017612	432427.7	46.03601	0.001359
0.260331	0.5153	2.916209	0.248532	3.118078	0.02138	33749.08	14.77039	0.010167
0.185108	0.415075	6.17402	0.042963	4.463364	0.009125	2705787	174.7926	0.000211
0.193836	0.43324	4.490936	0.077755	4.383161	0.014191	6704795	762.4293	8.53E-05
0.252715	0.517051	6.225752	0.065102	4.2154	0.015835	577775.3	48.7715	0.000566
0.234062	0.486298	6.587945	0.085875	4.600482	0.00949	2695069	184.6299	0.000137
0.267585	0.51899	7.216844	0.085251	4.111957	0.014351	407930.6	70.99835	0.000559
0.391715	0.649778	14.42046	0.068706	3.777016	0.020476	153912.4	28.08059	0.000919
0.202479	0.439962	6.933689	0.050877	4.31321	0.020955	238418.9	32.10994	0.00116
0.249347	0.508035	10.90505	0.047262	4.404118	0.022344	623935.1	82.20442	0.000297
0.312356	0.577228	24.70121	0.019717	4.633413	0.024043	2226407	217.5565	4.58E-05
0.106065	0.290877	4.307668	0.034885	5.35266	0.008035	5085059	165.8302	0.000169
0.185096	0.426343	9.885912	0.063668	5.076555	0.019016	104858.8	17.11417	0.001213
0.270096	0.522389	7.939834	0.066109	3.859583	0.012059	365274.2	52.94714	0.00074
0.221109	0.470481	6.452517	0.103423	4.293969	0.02944	47167.72	19.03549	0.002272
0.381531	0.638797	20.14585	0.027553	3.856448	0.007853	6877519	380.9169	4.76E-05
0.224772	0.468275	5.842949	0.061129	4.217605	0.036532	22865.58	9.17144	0.00455
0.240819	0.502266	20.52649	0.019265	5.134182	0.018711	1719307	77.95316	0.000131
0.153061	0.343766	2.352427	0.120581	3.797903	0.022293	41514.34	14.15303	0.010186
0.161837	0.390764	13.32605	0.017714	4.738154	0.030763	125010.4	10.28612	0.001307
0.221884	0.478441	12.68364	0.093919	5.415907	0.016952	152443	22.21013	0.000641

0.297119	0.559761	436.9304	0.002269	5.172679	0.015857	1743198	3.286665	0.000173
0.246152	0.504088	21.27979	0.027987	5.414094	0.011581	8841726	279.9975	3.04E-05
0.224677	0.475453	22.86333	0.011493	4.60021	0.013485	8777614	175.2507	4.86E-05
0.260016	0.516213	74.13446	0.015535	5.293272	0.027375	1978951	18.0244	0.000104
0.260117	0.519021	12.8386	0.037203	4.564548	0.015251	1557132	42.74436	0.000245
0.219346	0.47211	14.21096	0.031344	4.728566	0.014016	361867.7	21.3637	0.000631
0.228828	0.478435	6.64537	0.071961	4.357801	0.017343	449476.6	46.64837	0.000778
0.330039	0.590868	5.605223	0.139734	3.506482	0.005853	3916456	290.849	0.000173
0.158347	0.3857	2.184895	0.182001	4.238283	0.021073	167355.3	76.26336	0.002136
0.247424	0.501068	10.75417	0.055951	4.729223	0.028623	86848.75	20.46237	0.000939
0.212958	0.462027	10.91703	0.037287	4.517645	0.024149	124761.2	16.81724	0.001477
0.19878	0.447151	12.89699	0.027409	4.976254	0.009297	1301963	47.84465	0.000272
0.126543	0.319279	7.352811	0.027002	4.572431	0.027778	26766.89	2.016611	0.00812
0.238125	0.496207	4.817884	0.114267	4.237015	0.024707	155017.3	36.13643	0.001441
0.187634	0.427689	6.784573	0.038975	4.443507	0.018486	1593225	68.60392	0.000486
0.155172	0.349743	2.660681	0.083012	4.078784	0.026877	48179.82	8.776502	0.004466
0.142197	0.350947	8.200084	0.024324	4.576281	0.013474	599127.9	12.58558	0.001703
0.271622	0.527595	17.84846	0.022914	4.420604	0.014124	4185523	197.3154	6.95E-05
0.166241	0.398932	12.66364	0.025196	5.381935	0.013846	2106620	113.8818	0.000125
0.218194	0.46042	10.58136	0.036455	4.52268	0.027654	740761.9	64.83583	0.000351
0.259743	0.516974	16.36888	0.031554	4.626017	0.022497	637869.6	51.43934	0.000272
0.261755	0.517817	17.30215	0.024994	4.893017	0.013538	3323817	204.5712	6.21E-05
0.214511	0.462347	6.995196	0.052037	4.512951	0.02071	1487929	119.0962	0.000239
0.253086	0.436987	2.635023	0.21012	3.239098	0.090909	768.1111	3.237084	0.036583
0.202021	0.451646	14.32333	0.025245	4.932003	0.035869	95013.03	13.6842	0.001066
0.203612	0.441665	15.22224	0.015483	4.518939	0.020235	1878843	69.69899	0.000184
0.254645	0.512986	24.15712	0.020251	4.661774	0.022028	2263601	89.50339	9.98E-05
0.212729	0.463688	20.25841	0.033409	5.688759	0.023846	336503.6	32.22013	0.000318
0.191479	0.382403	9.041928	0.055566	4.757155	0.043507	14499.98	2.566117	0.005858
0.196281	0.441962	19.13937	0.046311	4.458543	0.039007	8037.64	2.481729	0.006446
0.166984	0.385245	18.03168	0.024682	4.961723	0.019985	767139	24.57672	0.000477
0.222553	0.465989	28.45732	0.018516	5.489089	0.035114	34825.55	3.742043	0.00166

PX105	PX106	PX107	VX1	VX2	VX3	VX4	VX5	VX6
7.326302	0.008408	0.006517	0.913168	0.823769	30.05784	36.48821	40.08333	44.75785
1.878971	0.012065	0.001364	0.893383	0.854027	33.89585	39.68945	47.79278	47.33627
9.471606	0.008255	0.003964	0.873342	0.801855	39.86013	49.70992	58.71961	64.36177
0.803405	0.001699	0.098797	0.89951	0.619313	9.574525	15.4599	18.43602	17.93847
7.664842	0.006085	0.043187	0.877675	0.79542	22.20587	27.91716	33.67329	30.67401
4.780773	0.004935	0.008275	0.803905	0.774833	34.994	45.16329	45.67346	51.98558
13.69712	0.007581	0.030132	0.949209	0.870111	28.44344	32.68943	40.5969	38.61473
9.311058	0.01009	0.013773	0.900658	0.719766	32.51765	45.1781	49.67734	54.07174
7.483548	0.01856	0.13101	0.717002	0.549938	9.835362	17.8845	20.26597	15.61
21.75834	0.005598	0.010234	0.788754	0.715375	46.19106	64.56903	75.12231	69.62119
35.6869	0.001878	0.01269	0.766249	0.7365	76.88759	104.3959	123.421	109.8951
18.50263	0.003089	0.001163	0.763875	0.680009	87.90404	129.2689	149.3126	145.4983
11.24573	0.004831	0.010119	0.876798	0.826763	39.78037	48.11583	56.61317	51.66898
7.302198	0.010385	0.026794	0.923551	0.793897	21.33448	26.87311	31.53	30.10399
2.80301	0.006989	0.069521	0.970325	0.830948	15.74944	18.95359	23.80488	23.80488
53.65345	0.007346	0.241127	0.764265	0.750809	62.83576	83.6907	84.7093	94.67668
3.366426	0.011281	0.01813	0.937683	0.881579	18.37244	20.84039	27.29472	25.29001
10.13161	0.008763	0.005041	0.767022	0.71135	42.14739	59.24983	62.59379	66.92948
7.263302	0.002846	0.017402	0.659428	0.461305	39.01222	84.56932	91.84583	82.34038
17.86233	0.002776	0.007556	0.886028	0.846547	69.92659	82.6021	99.84319	94.53739
7.977112	0.004818	0.001165	0.813317	0.786908	72.22197	91.77942	99.19402	102.5049
12.75511	0.006683	0.005866	0.860147	0.837511	49.03367	58.54686	66.31253	65.68359
2.761064	0.004804	0.054056	0.943572	0.728042	18.9883	26.08131	31.56323	30.3166
5.277981	0.005002	0.001991	0.850276	0.738858	57.95449	78.43795	94.77348	86.16044
12.70907	0.002502	0.015987	0.851075	0.739461	42.02934	56.83777	69.58655	58.4257
9.093586	0.003624	0.073496	0.90528	0.722857	19.99666	27.66338	32.56815	30.70608
13.74932	0.001346	0.033139	0.921886	0.711274	30.48305	42.85695	49.13557	48.7201
3.450999	0.007648	0.003782	0.798871	0.647618	32.74634	50.56429	57.4182	59.41015
5.394829	0.011159	0.017037	0.75113	0.705054	27.784	39.40693	46.02154	41.29438
6.654768	0.015037	0.007757	0.524811	0.413197	35.32767	85.49841	55.5482	52.68161
6.437671	0.009384	0.061979	0.599514	0.537457	19.29709	35.90442	26.97005	38.27097
10.55275	0.004293	0.005335	0.978389	0.786338	48.93043	62.22572	76.80334	75.76689
34.84433	0.436894	2.273756	0.875534	0.722842	23.0796	31.92898	36.57376	37.15072
12.7218	0.002956	0.006357	0.746987	0.551725	37.1565	67.346	62.24403	74.15747
29.05291	0.000977	0.004517	0.811141	0.761852	100.2658	131.608	136.7242	156.0904
12.59962	0.004584	0.092504	0.72986	0.424831	15.00162	35.31197	36.28976	34.39591
27.03717	0.000703	0.020318	0.667714	0.606559	64.66177	106.6043	105.8052	102.2836
4.19807	0.003908	0.043692	0.925636	0.878311	22.87149	26.0403	31.08373	34.51374
1.397858	0.00572	0.010675	0.795608	0.776023	28.02031	36.10755	38.10264	44.17931
11.49645	0.01757	0.091807	0.850915	0.722397	18.25897	25.27556	31.09198	29.44811
17.28428	0.006161	0.032911	0.502052	0.453676	34.57359	76.20762	64.78132	88.23539
18.8746	0.003814	0.001977	0.930667	0.882854	83.53896	94.62379	119.0507	110.0655
3.931813	0.01165	0.005896	0.915719	0.851785	29.28611	34.38206	40.98645	42.65138
8.860024	0.003005	0.004033	0.751666	0.689645	56.6722	82.17592	100.9673	92.43772
3.232989	0.006752	0.002611	0.830572	0.627761	46.35769	73.84607	87.27619	79.60727
2.85051	0.018986	0.006707	0.82111	0.784427	22.10907	28.18499	31.10064	37.78204
9.271102	0.005644	0.034988	0.81562	0.661852	22.51144	34.01281	38.8557	39.8844
3.26504	0.008088	0.002617	0.851577	0.740048	43.5075	58.79013	64.52175	64.77182
6.262865	0.009042	0.004705	0.898066	0.763082	39.2562	51.44425	61.26439	61.26439
3.218861	0.006248	0.002431	0.627431	0.51272	40.70429	79.38893	69.309	85.42308
10.3056	0.00311	0.006597	0.856327	0.735439	50.8571	69.152	83.47774	79.3348
7.627227	0.012563	0.035552	0.899921	0.872367	22.97331	26.33445	30.79215	32.04737
7.099396	0.009647	0.013444	0.878172	0.834458	29.28208	35.09113	41.93217	41.32551
2.723025	0.004903	0.008655	0.619089	0.515746	27.76582	53.83625	57.2905	54.87828
124.2567	0.000855	0.122847	0.748586	0.676676	56.51807	83.52312	93.0311	95.39902
3.04066	0.004813	0.004695	0.932545	0.870004	41.45557	47.64986	56.29923	56.98093
8.393898	0.005317	0.015974	0.818487	0.733113	31.36424	42.78226	51.7286	50.03173

11.23375	0.011187	0.036156	0.883995	0.818316	24.57444	30.03051	36.82353	35.5881
2.23335	0.01396	0.007796	0.890263	0.70674	20.0768	28.40759	32.40356	30.76643
31.42572	0.003036	0.066302	0.878745	0.699619	28.02209	40.05336	45.22108	48.78461
29.64685	0.003645	0.002273	0.856816	0.723771	92.4145	127.6847	145.2305	146.4107
19.23842	0.004398	0.1334	0.853191	0.72548	21.01562	28.96789	32.38489	33.31251
9.880271	0.003196	0.026356	0.918662	0.784297	47.55032	60.62796	71.84616	75.15548
12.72094	0.002685	0.004954	0.899198	0.757342	57.90293	76.45546	86.40982	95.97653
24.78569	0.001762	0.016583	0.787629	0.647	57.93225	89.53978	106.3297	102.2996
3.432274	0.007691	0.017749	0.83734	0.723282	22.86032	31.60636	35.92711	36.49258
2.121608	0.012545	0.014919	0.803438	0.642106	18.31878	28.52924	28.71822	34.34856
2.362266	0.015561	0.014211	0.869469	0.764642	19.53525	25.54824	30.7614	31.2219
6.807923	0.008961	0.001926	0.868168	0.646697	49.00828	75.78248	83.26624	75.60771
27.96324	0.001766	0.002726	0.921015	0.738554	88.86119	120.3178	134.9281	143.7228
9.569737	0.003048	0.002781	0.912713	0.809856	60.84486	75.13045	85.21743	85.38283
10.56291	0.003549	0.001862	0.965525	0.778253	78.31072	100.6238	127.1424	126.5622
42.92054	0.000591	0.038778	0.73883	0.587721	57.32802	97.54295	83.69938	106.8517
1.422711	0.010272	0.038779	0.808468	0.723705	15.82713	21.86958	25.4281	25.02933
4.376236	0.009745	0.005403	0.674088	0.667489	34.41101	51.55296	59.06613	58.34975
9.59274	0.000935	0.008844	0.857905	0.680859	64.21397	94.31321	99.18291	102.4669
4.419389	0.015314	0.004659	0.779022	0.733776	32.55795	44.37039	50.6478	50.6478
74.79171	0.000865	0.06825	0.802433	0.607428	47.80585	78.70211	81.95966	91.98801
2.728094	0.018934	0.001545	0.923469	0.834504	35.07545	42.03149	51.36949	48.21636
6.771424	0.006585	0.008456	0.866373	0.813336	34.50019	42.41811	43.38978	53.69299
15.48584	0.002034	0.014449	0.883187	0.838784	47.29448	56.3846	66.03775	66.2818
7.792993	0.002714	0.093583	0.913803	0.789562	21.83991	27.66079	31.68802	30.67763
61.4301	0.000928	0.047151	0.827625	0.748184	61.74261	82.52327	96.38792	96.0602
7.43414	0.003984	0.000831	0.632286	0.590869	82.07675	138.9086	143.9388	154.8664
14.96083	0.007105	0.010623	0.875056	0.739686	44.26248	59.83956	67.33603	65.60938
4.775079	0.004706	0.018112	0.711963	0.634934	28.9852	45.65072	54.1063	54.94574
8.847462	0.005313	0.012699	0.906977	0.8153	38.07322	46.69845	53.7967	56.13774
15.07546	0.008522	0.152422	0.936175	0.797552	18.26133	22.89671	27.26164	26.98447
12.30654	0.002421	0.018312	0.823765	0.748816	46.22507	61.73089	71.172	65.99027
9.100599	0.005049	0.002653	0.782786	0.724979	66.5375	91.77848	105.6264	107.5373
86.7919	0.00093	0.475271	0.941299	0.793154	41.56775	52.40815	62.26306	61.28472
6.769765	0.008077	0.00722	0.855978	0.736363	28.27731	38.40131	44.58305	41.70361
6.955344	0.006733	0.001757	0.765748	0.733211	40.94782	55.84725	64.68543	63.96915
16.24253	0.003	0.129115	0.849556	0.700343	20.40365	29.13379	31.76697	29.77548
10.18155	0.000454	0.025708	0.943429	0.771876	43.77607	56.7139	70.54652	66.05162
1.673394	0.009649	0.017117	0.962247	0.754554	13.0093	17.24106	21.65965	22.01449
3.98289	0.014354	0.013677	0.864321	0.799541	16.89971	21.13677	26.75622	24.22644
6.879793	0.012498	0.008357	0.840873	0.715442	24.47144	34.20466	40.5525	37.156
4.81777	0.006438	0.003686	0.847538	0.838733	35.54921	42.38441	48.3828	50.85854
13.05779	0.006522	0.126591	0.85727	0.733912	19.09093	26.01257	30.59758	28.31191
7.635845	0.005543	0.003242	0.868496	0.825695	46.07392	55.80014	63.09715	61.44306
8.43715	0.007495	0.008726	0.807843	0.607086	32.79642	54.02266	56.11778	56.10386
3.438855	0.008794	0.008872	0.829126	0.724299	22.75288	31.41363	40.10406	34.30297
28.16056	0.001694	0.164814	0.817469	0.736381	33.57581	45.59567	49.53892	58.02235
5.923894	0.006332	0.006447	0.877487	0.792172	39.67935	50.08934	54.5258	59.82545
6.250656	0.008909	0.021854	0.78858	0.717904	19.89022	27.70596	31.1332	34.2683
18.23879	0.005006	0.052865	0.935252	0.872718	31.90819	36.56185	42.68684	42.68684
13.00095	0.003103	0.004619	0.748417	0.562724	40.26826	71.55951	67.6774	71.34235
7.799183	0.004521	0.000841	0.807891	0.716797	61.1882	85.36331	102.3694	87.64219
42.99963	0.001534	0.014375	0.890541	0.736078	58.68577	79.72767	96.0121	88.0842
5.296948	0.004634	0.018501	0.953186	0.826333	24.64693	29.82686	35.96346	36.30284
3.583211	0.002765	0.008885	0.24423	0.21672	29.01492	133.882	43.59534	41.76389
44.11352	0.001569	0.013898	0.904239	0.782684	78.83432	100.723	112.8117	117.0942
17.75949	0.0075	0.016508	0.736065	0.580765	25.70306	44.25724	45.08392	43.87008
11.0818	0.003707	0.005825	0.817612	0.729617	40.27279	55.19715	66.32011	71.91661

10.17045	0.010941	0.035189	0.868994	0.801302	25.06412	31.27925	34.97232	37.21741
4.532202	0.01296	0.005483	0.847588	0.795024	25.13009	31.60924	38.05327	39.06156
222.1858	0.000492	0.170785	0.66311	0.648106	66.75401	102.9987	114.0381	115.3926
3.115485	0.006272	0.009029	0.721496	0.638553	20.23569	31.68991	36.41329	36.64881
19.69398	0.006596	0.017411	0.953546	0.846772	32.80542	38.74174	50.07157	47.39529
5.275214	0.001291	0.004834	0.724771	0.623405	46.57753	74.71471	84.3844	82.74216
2.394653	0.015482	0.010736	0.921404	0.792728	20.594	25.97864	30.76544	28.56368
8.308373	0.002822	0.008193	0.850007	0.80643	45.34558	56.23001	62.28736	61.35002
4.45919	0.009972	0.015438	0.808367	0.689974	21.99424	31.87694	37.82961	37.57553
3.682814	0.008802	0.004271	0.927224	0.849643	28.53248	33.58174	40.26787	38.80234
5.998553	0.006437	0.003742	0.860282	0.738286	34.01365	46.07113	54.12218	55.16046
15.47958	0.017778	0.016692	0.745383	0.69147	31.95008	46.20599	51.68357	44.35002
58.33803	0.001639	0.050879	0.855992	0.824489	41.22673	50.00279	58.61007	58.55231
4.838417	0.001826	0.004571	0.845146	0.786996	46.31287	58.84762	66.91402	70.68594
10.323	0.005247	0.00386	0.680586	0.663521	58.6839	88.44319	122.0795	89.44885
7.835791	0.010186	0.009063	0.910379	0.820264	30.73154	37.4654	45.25392	46.96404
14.74713	0.008585	0.004816	0.912808	0.742238	49.2099	66.29932	70.99364	79.74489
36.05735	0.000321	0.020072	0.693175	0.575139	77.04873	133.9654	122.9685	151.5317
3.669094	0.010052	0.028658	0.847509	0.769035	18.7321	24.35792	29.25754	26.85558
3.583116	0.007475	0.005455	0.908715	0.770811	26.71719	34.66112	43.48869	40.4446
3.892947	0.011135	0.008036	0.949628	0.85194	21.88786	25.69179	31.76503	32.78387
5.822331	0.007742	0.004272	0.835952	0.714558	33.01356	46.20136	55.81463	52.61191
3.702351	0.010191	0.004876	0.82111	0.705061	25.81969	36.62052	43.0495	41.76389
4.107604	0.012681	0.014115	0.820863	0.741078	26.31543	35.50965	43.74581	37.20855
5.007071	0.005589	0.001112	0.753431	0.61303	48.00592	78.30929	92.98583	87.54696
2.238555	0.016454	0.001635	0.927117	0.801818	31.50532	39.29233	47.1583	48.59751
3.382781	0.00765	0.005979	0.936529	0.916103	36.39902	39.73247	49.16912	45.72825
17.17861	0.00394	0.013364	0.760734	0.73509	49.1397	66.84853	74.1279	76.90431
3.114945	0.00839	0.033639	0.936353	0.733983	19.56543	26.65653	33.34473	32.32081
42.03532	0.004015	0.155282	0.521579	0.489554	34.28288	70.02878	79.34945	84.65326
4.835509	0.022821	0.072845	0.669431	0.567885	12.82738	22.58799	23.97361	20.66413
12.22467	0.015302	0.025424	0.801152	0.732063	27.71106	37.85338	42.64796	47.13999
9.62997	0.007477	0.006854	0.738017	0.645289	50.89798	78.87629	93.46509	95.4378
8.17103	0.006544	0.0034	0.913055	0.836355	60.50117	72.33908	86.05106	87.10461
10.7026	0.009633	0.030956	0.797591	0.754115	23.09934	30.63106	35.5022	35.5022
4.679101	0.020471	0.035563	0.65454	0.544681	14.34755	26.34118	27.90392	31.78115
6.773135	0.014789	0.018092	0.735538	0.610396	28.75212	47.10407	52.21514	53.60709
10.57614	0.01323	0.010568	0.904871	0.771667	43.62634	56.53518	67.93118	69.93025
9.88554	0.002032	0.025826	0.824455	0.682003	46.42108	68.06579	70.55622	81.14657
17.72063	0.01041	0.010815	0.959896	0.876213	38.4647	43.8988	56.04622	52.20493
6.681277	0.008456	0.009986	0.95945	0.76718	35.99812	46.92268	54.21363	57.54038
16.8962	0.028604	0.148605	0.533351	0.471426	19.5931	41.56136	45.39814	41.19764
17.63725	0.023509	0.022085	0.854046	0.790052	45.97451	58.19177	69.64201	69.21845
16.79211	0.054926	0.081863	0.77277	0.620385	14.56715	23.48083	26.16721	27.70884
6.421666	0.009117	0.019396	0.7102	0.599831	30.14398	50.25415	59.72015	56.80314
14.96023	0.015983	0.019601	0.492937	0.384188	31.66978	82.43303	79.95423	87.60729
23.79161	0.018739	0.038502	0.884284	0.811539	28.9414	35.66236	41.99774	41.57032
4.622304	0.003138	0.005214	0.90509	0.769545	58.29883	75.75755	90.48696	96.96244
8.020112	0.00656	0.036545	0.860019	0.817832	32.54098	39.78932	49.13312	47.97416
10.04263	0.002137	0.008895	0.986641	0.804973	53.9636	67.0378	80.40581	87.11886
7.710051	0.011936	0.008942	0.962639	0.701051	32.05283	45.72108	54.5369	58.42711
24.60995	0.01042	0.048338	0.525127	0.465149	28.11923	60.45213	67.84255	64.6685
5.359204	0.027788	0.068327	0.690453	0.64898	14.74194	22.71555	26.91233	25.47797
15.51563	0.009327	0.013408	0.526609	0.481196	27.50476	57.1591	50.58002	62.70736
16.34323	0.014829	0.011239	0.973609	0.864209	44.25698	51.21097	70.22143	61.39546
4.126349	0.010997	0.040769	0.729244	0.533011	16.85462	31.62153	26.74982	34.44537
8.263624	0.003951	0.003013	0.397849	0.280334	66.87923	238.57	209.2026	117.595
19.78503	0.014463	0.042295	0.784439	0.692907	31.57714	45.57195	51.22249	51.75379

12.22429	0.008265	0.007897	0.850135	0.702416	50.95227	72.53861	85.84654	78.42545
5.888507	0.011854	0.007826	0.597025	0.510207	33.13435	64.9429	74.50643	72.74696
17.59249	0.03275	0.023785	0.958177	0.905882	33.99475	37.52669	46.33692	46.53967
8.386766	0.006616	0.030559	0.819848	0.755651	32.30147	42.74657	47.30022	51.88588
18.57129	0.008325	0.008836	0.734077	0.592704	45.74115	77.17375	87.39171	88.61359
24.38271	0.023315	0.04729	0.880286	0.87088	37.77876	43.37999	50.69269	54.53595
5.239305	0.008871	0.007357	0.710705	0.548028	43.50851	79.39105	80.83854	94.65616
16.21548	0.016212	0.018569	0.62684	0.59741	35.02604	58.62982	65.538	65.83608
1285.536	0.000488	0.366953	0.81596	0.695458	116.6711	167.7616	202.7357	203.9132
13.56233	0.017492	0.014503	0.75115	0.664784	40.00179	60.17259	68.13221	59.79457
19.85405	0.017412	0.043062	0.810057	0.636745	24.92706	39.14767	41.82861	44.90547
20.06914	0.002283	0.00371	0.915752	0.803975	90.38205	112.419	143.9498	155.0611
10.35347	0.007021	0.006565	0.743713	0.71862	53.89362	74.99598	90.11278	86.76702
13.72951	0.008596	0.003142	0.87145	0.775859	69.22685	89.22603	96.08231	99.60725
5.455721	0.006991	0.092234	0.788947	0.680206	16.33928	24.02106	28.125	27.30444
9.236021	0.009536	0.012753	0.837241	0.76648	38.43395	50.14346	54.02151	61.34522
12.56746	0.019671	0.002387	0.80932	0.719985	61.02776	84.76252	96.21178	98.01304
15.09633	0.012503	0.00745	0.757338	0.593932	45.7874	77.09194	76.57467	91.0187
7.032745	0.010002	0.077284	0.873564	0.804123	27.70291	34.45109	46.59195	39.53304
15.70093	0.013886	0.047326	0.792121	0.694538	27.7225	39.91502	41.41546	48.6146
7.629066	0.006576	0.004864	0.84102	0.718127	46.49223	64.74096	77.11155	75.98372
2.333807	0.017654	0.04087	0.95997	0.813803	13.63075	16.74946	21.04813	21.04813
7.600339	0.004465	0.002638	0.963373	0.87163	69.10478	79.28226	90.18324	95.13868
10.48467	0.00425	0.010915	0.845028	0.83699	53.55686	63.98749	77.64996	84.70768
19.63081	0.100404	0.171892	0.612163	0.558479	13.10964	23.47383	25.51472	26.36878
16.97038	0.006611	0.014008	0.882598	0.739039	41.80257	56.56338	73.60631	65.11324
47.68028	0.001189	0.024952	0.867347	0.748783	58.03842	77.51031	92.91999	87.07484
7.132162	0.009808	0.005312	0.932819	0.829055	41.71452	50.31572	59.9569	59.27892
15.68673	0.00977	0.015934	0.813442	0.70588	42.19198	59.77216	60.2599	67.02019
12.82356	0.025688	0.091409	0.925074	0.746021	14.44902	19.36813	23.33635	23.84736
24.676	0.022275	0.020179	0.902631	0.825902	39.19256	47.45423	51.88541	55.55595
11.11476	0.017654	0.013077	0.705724	0.65653	32.81521	49.98283	56.33214	56.2429
17.07967	0.013325	0.03352	0.812426	0.756146	32.90697	43.51931	52.09666	52.09666
11.29882	0.027541	0.012327	0.914991	0.842986	35.20329	41.76023	48.97903	48.08991
13.52182	0.111931	0.240514	0.716214	0.601895	8.333888	13.84609	16.49271	14.16287
1.08116	0.003277	0.007565	0.760317	0.484875	22.23755	45.86245	54.78652	41.67513
0.702178	0.008742	0.023194	0.859465	0.7623	12.2732	16.10023	19.82898	19.82898
3.110058	0.005259	0.002746	0.57678	0.545092	38.89512	71.35516	73.52362	74.64087
1.319326	0.005313	0.000457	0.802477	0.746411	62.8152	84.15629	96.06614	92.82132
5.140701	0.004702	0.009409	0.928672	0.815179	33.40284	40.9761	47.51992	48.67438
8.051434	0.004601	0.003738	0.693413	0.600959	44.20467	73.55683	75.05939	73.20741
4.188463	0.00997	0.006789	0.874419	0.797869	26.61496	33.35754	36.94055	37.76887
7.225302	0.009631	0.020137	0.930215	0.890789	29.20186	32.78202	39.82614	37.56383
2.909646	0.007796	0.012924	0.861057	0.621652	26.48152	42.59863	45.95819	42.47824
6.251389	0.007987	0.00631	0.803345	0.755553	42.1872	55.83616	68.25056	62.88415
24.84883	0.007072	0.002677	0.793922	0.705712	76.10433	107.8405	118.2842	112.9754
3.930336	0.003521	0.002697	0.812714	0.725112	33.86218	46.69923	56.59941	54.31607
11.31141	0.01659	0.031572	0.717285	0.618164	22.45605	36.32699	40.1111	32.89972
4.193562	0.00867	0.009401	0.984193	0.888636	30.59145	34.42517	42.73527	42.73527
4.339655	0.016008	0.022744	0.634399	0.555289	18.28266	32.92461	29.40636	35.55332
10.9401	0.005299	0.002049	0.807624	0.752336	75.84019	100.8063	116.0116	118.2108
3.990983	0.012333	0.048871	0.94565	0.668597	14.62645	21.87632	27.04163	25.31359
17.5811	0.005481	0.007313	0.905072	0.723694	51.88482	71.69442	81.73903	84.13182
0.583838	0.006746	0.021613	0.751316	0.559743	11.93908	21.32958	26.85462	24.98352
11.119	0.004181	0.052291	0.538448	0.466383	21.80864	46.76117	34.05034	40.62099
24.9249	0.057455	0.022881	0.907579	0.678956	30.94649	45.57953	53.06614	55.01026

208.6648	0.000733	0.68451	0.629785	0.552776	56.0139	101.3319	113.99	106.8826
22.05617	0.005076	0.002805	0.92784	0.778869	78.89921	101.2997	123.0409	124.5747
14.07318	0.001562	0.004095	0.633005	0.539731	63.29756	117.2761	123.5937	124.8793
528.3824	0.000646	1.710238	0.919392	0.796555	59.21655	74.3408	85.18017	88.10589
16.05816	0.001226	0.024227	0.927138	0.819738	45.8876	55.97834	70.44606	63.63235
13.55382	0.006611	0.032059	0.810477	0.540061	22.98825	42.56604	51.23297	46.31788
2.782462	0.005387	0.009609	0.915597	0.76732	28.23889	36.80199	42.94138	39.90127
3.776077	0.006234	0.001889	0.848722	0.806199	46.83227	58.09017	67.35627	60.35092
0.668162	0.0118	0.004503	0.925076	0.780797	19.35387	24.78731	32.71272	28.94912
12.12457	0.020728	0.026446	0.762539	0.660637	23.4467	35.49106	38.82353	41.68663
5.706743	0.011113	0.029893	0.913583	0.766397	23.26754	30.35965	36.05249	36.38222
13.27377	0.004424	0.015856	0.92157	0.870315	30.64292	35.20901	40.66472	48.16387
6.742284	0.009994	0.284146	0.806153	0.680822	12.93094	18.99315	20.49431	22.54395
3.033314	0.007733	0.011555	0.74567	0.728595	25.07763	34.41915	43.22079	35.70414
1.778522	0.001429	0.006285	0.935943	0.872617	41.31763	47.3491	60.07962	54.43153
4.374799	0.004669	0.074781	0.91806	0.857755	18.71614	21.81992	29.37554	25.71171
2.640651	0.001151	0.044288	0.836525	0.755574	26.31852	34.8325	42.12264	39.43264
10.00203	0.00307	0.003375	0.914864	0.858433	69.50789	80.97063	93.74284	94.991
11.69462	0.006333	0.005015	0.843153	0.686653	37.31904	54.34921	63.40334	60.49781
4.335427	0.005117	0.007572	0.890925	0.850893	42.27943	49.68832	59.06613	56.98843
12.10293	0.006248	0.01063	0.578561	0.55594	41.1398	74.00045	84.07205	67.71837
15.70737	0.005148	0.003131	0.723895	0.6181	59.7991	96.74669	111.2784	112.8578
3.829679	0.003699	0.003526	0.581175	0.516332	45.42595	87.9781	98.42937	107.3755
2.551921	0.025709	0.18021	0.565248	0.488117	8.316553	17.03802	19.34678	16.95608
13.36168	0.007779	0.035097	0.753675	0.696939	31.92403	45.80607	48.3867	49.41949
8.857333	0.002089	0.00847	0.666995	0.521769	51.90711	99.483	103.4552	111.6754
16.76767	0.004271	0.008238	0.932224	0.841105	63.61612	75.63395	86.41929	93.4122
24.20428	0.042641	0.018765	0.828994	0.700265	40.06349	57.21189	60.66657	63.14441
11.02569	0.009785	0.202678	0.853232	0.782902	17.87611	22.83314	26.44971	28.13002
16.5215	0.074434	0.231656	0.775317	0.675908	14.01876	20.74063	22.33092	20.63225
8.288755	0.008647	0.026723	0.638003	0.607127	32.60103	53.69718	48.88228	57.68398
43.0577	0.013905	0.178417	0.768546	0.763779	23.92348	31.32251	38.24151	39.70964

VX7	VX8	VX9	VX10	VX11	VX12	VX13	VX14	VX15
40.32552	44.9452	26250.89	33.31986	0.786622	5430.177	0.206857	26315.4	23
40.82608	48.68746	34423.71	35.45788	0.781943	6544.562	0.190118	34484.23	12
51.2936	70.78768	61031.53	43.41377	0.781638	9590.648	0.157143	61145.78	64
16.20488	19.04201	1566.938	13.90633	0.822112	793.5744	0.506449	1600.429	93
29.84417	35.5105	11002.78	24.50219	0.806774	2965.29	0.269504	11075.02	54
52.37914	52.5647	40645.64	36.30699	0.76062	7516.093	0.184918	40760.48	8
36.72113	42.13605	20595.58	31.0291	0.808457	4494.45	0.218224	20697.08	23
51.64427	55.35702	41894.72	40.69	0.76299	7645.479	0.182493	42017.86	14
17.97604	20.57031	1693.263	12.82323	0.809197	849.01	0.501405	1744.944	46
63.62221	76.11486	102683.7	50.92911	0.684848	15484.28	0.150796	102893.1	66
110.8386	135.1384	446937.7	79.99324	0.702155	40260.82	0.090082	447421.6	19
112.3184	151.0225	784385.4	98.74532	0.701708	58615.73	0.074728	784685.5	47
58.09753	60.24729	56390.7	42.18788	0.716895	9919.741	0.175911	56520.64	45
29.95439	33.80204	10052.9	24.81868	0.799467	2817.588	0.280276	10121.09	63
21.77515	24.53513	3877.314	18.39115	0.818745	1457.771	0.375974	3926.615	71
89.80282	98.01987	232927.3	63.96189	0.715504	25586.99	0.10985	233142.8	38
23.40483	27.54635	5182.93	19.54168	0.786922	1840.501	0.355108	5234.441	34
58.53175	67.87487	78050.99	45.44589	0.68863	12825.78	0.164326	78236.29	17
78.88183	92.20978	97201.2	55.76739	0.578537	17671.25	0.181801	97412.07	58
86.47576	100.1643	301301.8	73.1878	0.738652	29424.72	0.097659	301515.9	19
99.56944	107.1844	357425.9	74.64574	0.759232	32079.96	0.089753	357678.9	19
68.65493	70.78195	101703.3	50.35889	0.725995	14513.55	0.142705	101873.3	21
29.48166	31.72633	8577.017	24.60961	0.781132	2594.082	0.302446	8642.136	29
290.6163	309.4156	199531.3	66.6939	0.737851	22379.78	0.112162	199959.7	27
62.26257	74.52669	81283.5	48.37323	0.748533	12122.94	0.149144	81423.64	39
30.95927	34.35108	9702.732	25.04309	0.778258	2826.767	0.291337	9778.649	59
100.4404	104.8956	35245.89	39.50921	0.75555	6880.604	0.195217	35349.05	53
49.19104	61.45096	47184.3	40.39434	0.740382	8528.904	0.180757	47293.02	33
40.13597	47.23212	22396.31	29.59971	0.757846	5070.148	0.226383	22509.77	46
329.5804	332.7882	43266.14	44.87054	0.748448	7963.21	0.184052	43420.61	22
39.1233	39.90679	10210.14	21.5252	0.735929	3092.689	0.302904	10304.63	47
74.44408	80.79685	127073	60.88093	0.702502	17399.57	0.136926	127247.3	29
35.27091	39.77912	14362.86	27.9549	0.776852	3678.24	0.256094	14459.31	46
70.66496	77.54398	84833.77	50.30659	0.680469	13721.07	0.161741	85001.13	24
143.7176	157.2249	972391.3	106.7527	0.687903	69000.3	0.070959	972807.8	22
39.27217	40.52535	9687.595	25.7728	0.720217	3051.391	0.314979	9771.533	68
112.7947	115.2431	341064.8	71.18113	0.591974	39878.58	0.116924	341190.1	19
29.52932	34.72647	9760.317	24.10383	0.764331	2889.649	0.296061	9842.188	7
41.27559	47.40082	20347.14	28.72745	0.760324	4740.468	0.23298	20451.89	68
28.24986	31.33446	6838.789	21.50734	0.747038	2332.352	0.341047	6899.932	51
54.15696	90.66412	65576.31	38.2602	0.623779	12607.19	0.192252	65776.24	34
103.718	122.5851	500362.1	88.06323	0.75749	40237.45	0.080417	500594.1	27
35.75115	42.93341	22845.64	31.48429	0.791374	4920.068	0.215361	22924.54	16
76.99228	101.2118	180279.1	61.76887	0.627449	24596.26	0.136434	180587.8	46
81.02587	92.71815	132504.9	61.33447	0.617313	20361.05	0.153663	132752.7	37
29.30814	38.43109	10073.86	23.14297	0.772813	2918.817	0.289742	10135.48	111
31.83969	40.25553	15045.58	27.74152	0.766888	3843.188	0.255436	15105.23	49
341.7049	342.1886	88260.62	50.06435	0.714194	13422.89	0.152082	88200.1	22
53.62504	63.86386	64108.58	46.20033	0.740231	10464.72	0.163234	64292.63	43
79.85645	86.69983	107980.2	49.81104	0.674434	16259.51	0.150579	108169.3	44
77.5444	84.44098	132841.4	59.2167	0.653037	19279.77	0.145134	133024	57
30.42973	32.31475	10026.45	23.69891	0.776631	2895.347	0.288771	10101.04	69.5
35.77639	43.25459	22523.96	30.81606	0.788896	4889.082	0.217061	22624.43	39
40.30132	57.61574	31115.56	33.32944	0.654042	7314.703	0.235082	31222.61	79
80.66048	97.35095	204226.4	62.5242	0.714879	23459.88	0.114872	204440.4	32
51.02428	57.61714	62513.6	44.43566	0.761563	10002.18	0.16	62658.58	64
39.92846	51.90305	33032.65	35.01674	0.779094	6390.33	0.193455	33119.81	53

32.69828	37.64623	13756.44	26.54684	0.746018	3721.676	0.270541	13852.68	54
32.42918	35.22089	10197.77	25.29022	0.792224	2870.603	0.281493	10271.12	39
43.63169	49.46948	26132.37	35.19667	0.721469	5902.724	0.225878	26217.84	41
136.1714	146.6543	926853.5	109.4023	0.73006	62969.88	0.067939	927203.1	34
32.83518	37.88364	10566.04	24.71516	0.748499	3111.007	0.294434	10634.85	47
70.25975	76.97854	102046.9	55.69658	0.670619	15747.38	0.154315	102289.4	38
90.08789	101.5154	204496.8	68.7486	0.664106	25275.78	0.1236	204757.8	30
92.45383	106.3463	251233.2	70.52412	0.685006	28108.72	0.111883	251537.5	24
31.42208	40.10694	13414.66	26.46525	0.7716	3538.44	0.263774	13492.97	78
30.54046	34.57217	8398.895	22.92148	0.765088	2611.684	0.310956	8479.549	46
30.43252	32.50229	7244.626	22.2134	0.752663	2405.622	0.332056	7324.082	74
80.94684	89.50179	170585.6	65.79194	0.72746	20447.34	0.119866	170792.6	11
136.8246	145.0472	830672.5	110.8145	0.709564	60225.33	0.072502	831090.5	26
89.65241	91.44578	218787.6	68.57252	0.697769	25164.48	0.115018	219022.4	19
115.6385	127.4306	520753.1	97.1548	0.695305	45019.08	0.08645	521021.3	30
355.6831	355.856	270768.1	72.06763	0.706109	28664.49	0.105864	270763.7	37
22.5993	27.38959	4185.345	17.68086	0.750566	1673.334	0.399808	4236.093	74
41.72163	61.29468	43812.92	34.75123	0.757304	7936.255	0.18114	43949.6	58
112.1808	113.5827	336363	80.91176	0.639111	36597.07	0.108802	336558.2	51
43.16406	51.93739	34505.32	34.56551	0.72415	7078.044	0.205129	34621.26	25
77.00373	92.93312	160082.3	63.15317	0.691754	20610.8	0.128751	160323.8	23
49.40827	52.28683	40380.4	38.81478	0.740782	7683.765	0.190285	40500.93	53
50.36838	54.09671	36921.09	36.7499	0.609022	8804.428	0.238466	36973.28	11
66.71088	69.16333	87943.89	49.79816	0.713928	13395.75	0.152322	88090.97	36
32.79582	34.48099	10873.28	25.27651	0.701701	3382.508	0.311085	10891.27	47
81.23586	99.69905	244479.4	68.2983	0.712695	26530.28	0.108517	244788.3	53
110.1642	160.6066	680353	87.82995	0.672581	55619.77	0.081751	680740.3	41
65.95491	69.1273	98636.92	52.36297	0.759366	13595.42	0.137833	98843.11	38
40.90271	55.10134	29231.37	32.50164	0.614827	7463.863	0.255337	29263.97	49
50.45758	56.24955	54018.02	42.35443	0.774597	8921.419	0.165156	54141.38	29
27.05424	28.84323	6128.42	21.43534	0.726572	2228.971	0.363711	6203.569	42.2
66.61796	74.81949	99897.15	50.85172	0.704464	14779.54	0.147948	100074.7	51
83.61226	109.4434	314240.6	71.84294	0.779581	28672.41	0.091243	314492.2	12
60.88023	65.55595	75650.47	49.33174	0.713414	12125.06	0.160277	75740.54	60
42.7799	45.80448	25189.7	32.87067	0.762716	5448.414	0.216295	25289.63	60
59.91816	68.04991	69144.02	42.76491	0.723724	11256.78	0.162802	69221.72	34
33.474	33.474	10274.62	24.75079	0.796378	2869.958	0.279325	10324.8	77
62.84467	72.20933	92551.92	53.50555	0.740171	13368.28	0.144441	92694.39	53
20.65262	22.90095	2567.52	16.59016	0.746347	1214.933	0.473193	2599.955	107
21.84175	27.00347	4479.464	18.26896	0.746435	1760.527	0.393022	4515.161	50
40.00855	42.0077	16922.24	28.76176	0.736248	4329.441	0.255843	16985.69	10
44.8494	51.63062	38650.77	35.92238	0.753619	7335.635	0.189793	38721.55	66
29.0786	31.22626	7526.103	22.29979	0.735043	2526.687	0.335723	7571.217	67
62.3077	65.78374	89560.99	48.4622	0.748865	12926.86	0.144336	89680	72
59.09765	69.56328	53079.83	43.6418	0.718808	9502.196	0.179017	53260.25	25
32.27575	40.28389	13171.28	26.04586	0.768862	3507.956	0.266334	13222.57	77
49.51088	59.16246	37087.53	37.27305	0.700509	7677.546	0.207012	37219.76	40
57.21096	60.16927	62439.01	43.95272	0.767839	9912.535	0.158755	62572.22	62
26.4259	36.28242	8556.396	21.84835	0.787853	2567.83	0.300106	8600.95	88
41.92902	45.73279	28345.02	34.19453	0.771969	5823.741	0.205459	28447.25	83
80.94294	82.44915	106278.5	53.55635	0.676587	16037.05	0.150896	106416.8	38
91.9094	104.1355	243040	68.96427	0.691897	27220.39	0.112	243193.5	31
85.45326	96.94718	236258.1	71.00078	0.746595	24754.65	0.104778	236486.8	30
33.0243	38.93696	14713.08	28.43054	0.793606	3658.883	0.248682	14767.12	90
359.0915	359.4688	22536.52	32.69803	0.695732	5545.835	0.246082	22619.42	52
107.805	124.5846	520350.2	91.07765	0.75371	41509.13	0.079772	520727.4	14
47.45051	50.76656	25516.44	32.57621	0.743007	5641.197	0.221081	25595.31	27
52.14665	72.83836	65172.49	45.12982	0.687561	11390.67	0.174777	65276.68	57

35.72061	38.10238	14895.56	27.18149	0.656007	4462.864	0.29961	14920.69	20
31.53667	39.20687	15320.55	26.79162	0.762034	3914.646	0.255516	15375.67	25
85.62131	117.5988	301127.3	68.29939	0.644695	33700.01	0.111913	301488.2	46
27.75027	37.97303	10290.91	22.86413	0.745061	3070.87	0.298406	10332.16	82
47.11401	53.73416	30447.38	36.94203	0.659628	7148.552	0.234784	30579.04	62
64.79703	85.32938	132371.6	54.15107	0.678651	18508.33	0.139821	132489	69
29.19421	31.9063	9259.558	23.93682	0.802576	2656.999	0.286947	9309.76	34
63.33857	69.33987	85911.23	47.79589	0.74376	12659.53	0.147356	86067.39	31
30.84516	37.95294	12260.79	25.76826	0.757188	3395.934	0.276975	12334.39	54
39.8131	41.09492	21446.32	31.13781	0.771734	4837.098	0.225544	21507.24	107
45.44742	55.58243	44134.49	39.63416	0.763863	7906.562	0.179147	44207.66	63
50.5024	52.08463	35684.32	34.44118	0.774868	6764.591	0.189568	35801.43	8
55.72955	61.02927	61502.97	42.80197	0.74344	10135.27	0.164793	61612.01	34
61.35442	74.69317	90616.14	49.73481	0.675553	14442.04	0.159376	90751.83	76
112.5823	122.7228	213732.2	60.19321	0.658723	26243.87	0.122789	214032	50
40.11253	47.33397	28020.23	34.10771	0.765386	5828.879	0.208024	28105.68	45
75.75943	79.97738	140842.3	60.51852	0.754732	17345.23	0.123154	141053.3	16
133.3091	156.8551	657690.2	92.86143	0.671042	54502.38	0.082869	658095.7	34
27.21858	29.39542	6649.427	20.64356	0.768139	2226.215	0.334798	6693.044	97
38.78934	44.09144	19444.01	31.49707	0.720504	4853.313	0.249605	19512.1	67
28.76875	33.48898	9530.896	24.39763	0.772778	2813.098	0.295156	9576.544	24
49.82718	56.87169	40569.99	38.62214	0.720327	7926.67	0.195383	40654.75	73
37.41423	47.2343	19818.82	30.06947	0.746867	4741.974	0.239266	19880	108
38.91469	44.01531	18963.63	29.14856	0.795313	4324.08	0.22802	19075.6	58
69.65355	93.37922	155165.8	59.00061	0.723007	19314.01	0.124473	155286	34
43.62891	50.18639	32228.18	36.42858	0.769645	6363.326	0.197446	32307.22	27
45.41368	49.77338	38267.95	37.21061	0.764309	7185.199	0.18776	38386.44	24
64.23382	78.09015	109262.5	50.85394	0.697811	15838.98	0.144963	109455.1	38
28.72504	34.9376	9116.919	24.95993	0.773208	2729.52	0.299391	9225.22	36
44.20645	85.0927	43772.45	36.52552	0.593261	10124.46	0.231298	44131.54	53
23.36974	27.76949	3202.514	15.1211	0.786686	1335.601	0.417048	3281.903	54
81.17563	87.98244	21276.1	30.32631	0.738305	5029.322	0.236384	21431.73	75
69.85432	96.12676	164596	58.21208	0.739598	19638.18	0.119311	164853.5	24
77.52571	90.75156	207547.8	66.04958	0.75476	22460.55	0.108219	207853	41
31.35811	39.95733	11840.62	24.43105	0.746804	3364.036	0.28411	11922.09	52
21.30985	31.9521	4600.613	17.24137	0.757169	1766.724	0.384019	4674.019	75.9
40.86345	56.29559	31902.34	34.64683	0.730104	6662.659	0.208845	32126.19	26
60.59185	70.66207	87146.14	51.15707	0.716266	13271.16	0.152286	87377.09	25
71.57039	81.39294	122138.5	56.11719	0.740718	16071.87	0.131587	122404.7	59
48.25008	57.38478	50288.37	42.1383	0.749809	8787.09	0.174734	50380.79	51
53.16388	58.43134	53676.28	45.01997	0.772877	8903.523	0.165874	53860.75	73
31.62987	48.49728	11463.41	22.16677	0.707794	3473.651	0.303021	11602.26	11
56.50082	69.96686	96063.7	49.69848	0.781839	12973.98	0.135056	96323.01	-2
21.66036	30.29155	4413.157	18.14528	0.767602	1695.043	0.384089	4493.958	-1
40.9791	59.96577	38068.15	35.69047	0.756923	7230.031	0.189923	38214.56	66
119.7887	127.3976	66321.92	40.6343	0.649271	12203.82	0.184009	66522.29	38
37.51772	43.51088	22814.97	31.53566	0.774369	5023.61	0.220189	22926.26	40
79.70473	97.11274	205770.8	68.56743	0.705719	23884.08	0.116071	206080.6	41
43.18992	50.17443	30498.29	34.21956	0.740917	6371.348	0.208908	30675.75	82
77.86831	89.79944	159735.7	66.14224	0.661754	21514.04	0.134685	160007.4	44
53.15923	60.5441	45809.38	44.01291	0.74173	8347.207	0.182216	46014.86	49
41.54699	68.26016	36407.04	31.74504	0.712733	7453.285	0.204721	36579.15	11
19.34552	27.85121	3656.13	15.68402	0.755767	1518.602	0.415358	3730.133	23.5
48.95914	66.42889	30430.93	30.1005	0.615806	7654.502	0.251537	30565.26	39
66.10916	70.53675	73968.48	49.85945	0.714711	11922.98	0.16119	74219.34	34
35.68273	36.77943	7431.65	23.0598	0.644228	2858.699	0.384665	7529.988	74
181.6582	231.824	424499.5	94.91492	0.610421	44747.9	0.105413	425054.9	22
44.14965	56.44373	35639.48	35.74841	0.755618	6931.109	0.194478	35834.88	28

76.5822	87.57953	155442.8	61.66764	0.673795	20749.28	0.133485	155803.7	40
47.09522	75.64352	56379.41	38.77251	0.707744	10046.66	0.178197	56551.41	46
42.24021	48.57544	32738.27	35.9572	0.790021	6264.448	0.191349	32928.61	46
45.0549	54.68778	33827.97	35.0457	0.766124	6602.404	0.195176	33993.9	57
73.53289	89.74359	132160	56.65148	0.660861	18986.32	0.143662	132453.1	39
46.37999	55.85451	44600.2	38.18678	0.779672	7800.636	0.174901	44837.17	29
69.69632	96.42071	132195.5	56.42361	0.661617	18968.01	0.143485	132432.6	57
45.53992	66.16525	50619	36.75154	0.712714	9284.912	0.183427	50818.49	5
160.5377	206.5985	1843009	136.8868	0.680529	106820.8	0.05796	1843745	23
70.3125	73.3208	64609.91	45.19867	0.669309	11633.86	0.180063	64869.46	21
39.21131	47.72111	20228.53	31.71183	0.729254	4923.207	0.243379	20382.84	20
122.3242	155.1403	725760.3	102.9479	0.70361	55506.94	0.076481	726181.7	64
73.39161	92.74671	147614.4	55.7755	0.669768	20167.2	0.136621	147922.9	32
100.039	111.3877	338089.6	77.75603	0.745627	31476.29	0.0931	338567.4	14
22.34566	28.34387	4537.989	18.95134	0.710576	1865.445	0.411073	4627.441	53
55.54496	62.06425	55154.74	41.98218	0.744523	9411.551	0.170639	55371.33	43
84.17525	102.7389	247209.9	68.59997	0.710097	26825.23	0.108512	247552.9	20
80.85562	93.3515	136683.6	58.38462	0.660862	19417.11	0.142059	137028.1	25
39.5488	46.96925	18565.88	30.09523	0.723915	4683.893	0.252285	18705.88	61
39.63156	49.89931	24063.25	31.61754	0.736172	5475.295	0.227538	24212.49	42
65.2784	79.77535	113389.7	54.44846	0.717404	15791.95	0.139271	113552	35
18.00879	22.97951	2759.903	16.07897	0.824233	1154.419	0.418283	2829.117	61.2
90.76625	97.68011	285727.7	76.37842	0.703185	29834.3	0.104415	286054.2	35
66.10871	85.54688	123845.4	54.07125	0.687667	17472.66	0.141084	124132.6	29
17.71647	27.25029	3126.27	14.36981	0.799091	1293.915	0.413884	3207.047	-41
63.10322	73.70203	81051.67	49.9227	0.740642	12228.8	0.150877	81291.89	20
79.14687	101.3171	216097.8	67.22832	0.76038	22902.72	0.105983	216335	35
57.2339	63.71666	70639.5	46.93545	0.764038	10816.02	0.153116	70829.23	17
71.39056	79.05702	82440.36	48.6212	0.670159	13668.88	0.165803	82711.81	54
20.69554	25.17097	3632.961	17.91695	0.818052	1397.045	0.384547	3682.462	6.9
54.60178	55.75508	57295.54	42.83367	0.800755	8975.626	0.156655	57469.86	22
43.82275	58.03897	35157.61	35.27407	0.651928	7960.931	0.226436	35308.23	16
42.2635	53.52467	36296.53	35.35621	0.788174	6726.234	0.185313	36439.92	55
48.70501	53.27747	40020.23	38.21021	0.779208	7261.346	0.181442	40159.96	-14
13.90813	17.12998	841.5712	9.916764	0.800318	538.6198	0.640017	877.9025	18.4
51.43356	55.87375	23128.86	34.87002	0.690208	5687.745	0.245916	23230.9	41
15.41318	20.68011	1873.9	13.83758	0.797215	922.0182	0.492032	1911.042	86
49.24731	75.23985	76012.23	41.15624	0.6944	12496.73	0.164404	76140.35	39
85.19423	101.9899	247275.3	67.53345	0.699605	27232.34	0.11013	247493.7	51
44.96156	51.42629	37823.89	38.05336	0.800062	6810.902	0.180069	37933.36	11
75.37643	92.68358	112312.1	51.00523	0.703981	15990.94	0.142379	112492.5	35
38.39359	39.95335	18674.23	29.16845	0.787442	4322.754	0.231482	18765.48	38
38.17281	42.0564	20305.87	30.49433	0.773509	4653.36	0.229163	20419.87	25
34.73516	300.0412	20550.22	36.67986	0.744493	4873.425	0.237147	20631.69	52
55.06395	68.87008	74769.87	44.85571	0.760474	11286.28	0.150947	74933.87	39
112.4599	129.3896	494701	85.61697	0.686037	44092.52	0.08913	495074.8	26
46.43109	57.00885	42420.04	37.95313	0.73139	8042.343	0.189588	42482.35	34
39.73046	40.95696	14930.88	26.05682	0.760915	3853.641	0.258099	15022.78	16.1
39.40814	45.5627	25329.74	33.88101	0.78611	5305.843	0.209471	25450.58	30
38.08544	38.7845	8250.189	20.88735	0.704813	2801.469	0.339564	8329.63	59
101.9119	120.2035	417411.1	81.41355	0.687887	39265.36	0.094069	417852.8	16
25.20485	27.7722	4957.752	20.68734	0.780606	1801.256	0.363321	5053.848	40
95.9767	97.47392	152096.6	64.88864	0.662996	20783.54	0.136647	152318.9	46
17.75268	28.0625	2651.403	16.02527	0.712457	1300.297	0.490418	2706.405	66
49.44683	55.17901	18323.01	25.17847	0.712896	4714.72	0.257311	18480.18	32
46.06408	56.57426	41377.84	41.36701	0.753837	7674.528	0.185474	41557.98	-34

77.82	115.6986	253918	63.8173	0.738034	26274.63	0.103477	254225.4	12
113.4354	129.3405	500397.6	93.98991	0.561094	54324.01	0.108562	500833.9	26
90.01272	137.277	379122.4	74.23638	0.677363	37398.03	0.098644	379389.7	27
90.19812	91.21804	201678.4	68.34831	0.681549	24402.06	0.120995	201960.2	57
61.66758	70.77696	94945.71	51.89966	0.758121	13275.85	0.139826	95109.65	74
43.54932	55.27657	22915.05	34.4988	0.715808	5450.482	0.237856	23029.13	19.3
42.13299	46.32099	24992.99	33.69578	0.772838	5349.023	0.214021	25081.88	40
64.9134	68.64299	96244.69	49.30242	0.779207	13034.14	0.135427	96406.64	42
27.25115	32.82844	7739.345	22.93014	0.758522	2494.511	0.322315	7793.318	70
34.07463	41.84632	15388.14	27.06331	0.745804	4011.591	0.260694	15481.96	35
34.00912	37.01886	13984.87	27.73607	0.761748	3685.065	0.263504	14039.78	69
37.8454	48.70194	24493.56	32.44758	0.74039	5508.814	0.224909	24553.89	67
20.69711	24.9185	2646.596	15.31138	0.755478	1224.769	0.462771	2671.385	84
33.47435	45.67824	14312.7	25.66533	0.700358	4070.473	0.284396	14405.81	63
54.5119	60.7276	60418.52	44.31608	0.743886	10009.78	0.165674	60558.2	73
24.7526	29.45722	5258.181	20.03199	0.734049	1992.122	0.378861	5326.007	45
33.73087	44.68929	18169.64	29.13826	0.741745	4506.028	0.247998	18247.6	49
88.1722	95.45756	294089.2	74.07708	0.761075	28100.2	0.09555	294369.9	67
56.04677	65.2231	65249.37	45.82472	0.727333	10776.28	0.165155	65343.04	39
53.69584	60.62778	65983.01	44.26857	0.767314	10291.19	0.155967	66148.71	70
61.53633	85.74783	84618.29	42.81381	0.686044	13586.51	0.160562	84843.29	45
90.3645	113.6292	282345.7	70.03445	0.656139	31720.67	0.112347	282676.9	15.96582
65.43577	107.4447	138479.5	51.13066	0.66528	19456.73	0.140503	138730.4	50
13.82543	20.39957	891.1377	9.630715	0.723147	619.2806	0.694933	945.3809	-2.7
47.58558	57.7906	34903.5	34.52291	0.718019	7193.291	0.206091	35116.78	48
84.41192	112.7406	227793.6	66.35466	0.661522	27266.82	0.1197	228107.7	46.97458
84.41593	95.4231	241954.6	70.50778	0.739534	25391.12	0.104942	242195.3	65
66.55595	71.39793	70797.98	47.42832	0.641795	12895.42	0.182144	71027.52	-54
24.79515	29.31265	4999.351	19.48197	0.727966	1942.297	0.38851	5074.644	36
23.16455	24.56086	3129.085	16.08057	0.754764	1370.728	0.43806	3189.045	-108
59.15192	61.50008	42126.58	34.25899	0.699904	8365.332	0.198576	42248.28	64
32.8535	40.08087	11566.95	24.07278	0.669347	3695.266	0.319468	11675.41	61

VX16	VX17	VX18	VX19	VX20	VX21	VX22	VX23	VX24
80	75894797	1.96848	30	3.838029	161	17.95294	50.00722	48
77	88919213	2.021694	40	2.597236	139	21.52131	40.60393	34
95	2.18E+08	1.24469	16	8.718972	139	9.790814	79.72154	81
126	11143315	1.282621	16	3.274616	148	10.36534	110.3514	111
111	37075886	1.898908	30	3.058336	142	17.6124	85.68101	89
56	24358251	1.810711	23	4.75638	129	15.28685	28.87399	26
77	21417825	1.932313	28	5.864922	126	17.25504	49.79877	50
78	46286877	2.160828	32	4.088623	135	20.3322	45.05756	46
87	3321165	1.602954	23.75	3.52655	111	13.54567	66.6453	67
128	4.15E+08	2.060485	30	4.532259	194	19.16579	99.31445	102
80	5.56E+08	2.024242	35	3.6604	138	19.43903	50.17494	51
96	2.53E+09	1.782118	24	3.501433	187	15.01621	72.25616	72
105	1.78E+08	2.017009	32	3.695114	165	18.71289	76.11221	78
138	60526522	2.246444	41	2.815786	173	23.42721	104.1717	108
112	16006226	1.648706	20	10.48599	130	13.8506	92.1343	95
87	4.64E+08	1.80215	24	5.12921	133	15.60466	63.76894	66
95	14569471	1.903304	33	2.537442	121	18.95729	69.31271	74
90	1.08E+08	2.193876	43	2.704019	154	23.8488	47.05972	39
170	6.42E+08	2.871042	55	3.44926	268	34.38458	115.2599	117
110	7.62E+08	2.270191	49	2.348371	160	28.13967	74.67707	86
95	6.37E+08	2.224985	51	1.901963	139	25.29595	56.7848	58
115	2.75E+08	2.476587	56	2.125689	157	29.53018	72.40269	77
77	13968355	1.818191	24	5.367909	151	15.59188	53.73746	54
98	6.55E+08	2.171438	43	323.4905	172	22.89054	62.91519	64
91	2.17E+08	1.886499	24	6.578407	150	16.48879	67.71937	71
98	26442758	1.493704	19	5.510397	193	12.35088	80.31113	82
79	1.08E+08	1.103951	13	1090.332	216	9.081125	65.74129	66
117	1.98E+08	2.363741	48	2.333363	162	25.87695	77.25028	80
96	56198830	1.8503	23	6.017969	142	15.63846	71.02909	72
102	1.69E+08	2.358672	47	175.6402	138	30.22123	61.10244	69
105	24748985	2.048789	26	7.005635	153	19.19089	77.84308	81
92	2.81E+08	2.057437	32	2.972002	158	19.16498	61.70176	62
88	22727286	1.653498	22	4.714127	138	13.73741	67.25901	68
99	2.19E+08	2.223271	48	2.244884	138	24.66425	58.20121	56
79	1.13E+09	1.899686	35	2.375616	264	18.48408	50.80079	51
107	37379453	1.578431	19	7.128606	126	13.08236	89.17069	93
115	3.29E+09	2.586252	60	2.704117	580	31.25807	66.40154	66
55	4179534	1.801473	25	3.540521	107	15.50347	29.22478	28
100	57231354	1.261755	16	4.732558	127	10.20269	84.20292	85
131	35154451	2.277196	33	5.365589	174	24.59528	97.92779	106
106	1.9E+08	2.26907	37	4.303911	175	22.85943	73.49427	78
133	2.38E+09	2.544571	60	2.319061	200	33.3605	90.41037	102
114	68697662	2.622164	61	2.28022	173	31.69966	64.44966	64
107	6.99E+08	2.047848	31	3.351753	199	19.09022	77.82346	79
84	2.09E+08	1.688359	25	2.903392	126	14.70065	62.07322	64
162	1.14E+08	1.864988	27	3.79944	202	16.28032	135.5563	135
102	61624198	1.888769	27	7.348209	138	17.22727	76.92333	80
117	3.64E+08	2.428549	50	53.16465	184	28.63995	76.95428	84
115	2.24E+08	2.13559	34	2.923758	155	21.30584	84.05333	88
120	3.99E+08	2.283219	36	4.374614	178	23.18635	86.45564	91
106	4.87E+08	1.796406	24	12.20057	151	15.72506	82.49754	85
114	39992006	1.60341	18	5.555302	137	13.64132	95.38665	99
102.6	50769920	2.062459	36	3.388094	138	20.49341	73.32235	78
133	2.04E+08	1.880428	27	9.159365	171	17.12962	108.2169	112
111	5.43E+08	2.350564	48	7.968622	548	26.01697	72.57294	73
122	3.36E+08	1.947386	24	13.08991	164	19.51165	97.26664	104
132	1.99E+08	2.186787	34	3.39	184	23.17348	101.0436	109

112	39297185	2.021578	27	4.844802	154	18.22957	84.15284		86
102	25418996	2.036353	33	2.902817	154	19.23862	70.35752		70
105	1.03E+08	2.044866	27	6.779068	228	19.40635	77.82643		83
99	2.71E+09	2.05747	32	2.84893	209	19.29676	68.82856		71
117	46172781	2.178612	36	2.783905	165	21.63688	85.04671		88
79	1.06E+08	1.626778	20	7.980831	117	13.74981	58.98086		61
81	2.95E+08	1.765606	26	3.16315	124	15.70795	57.99859		61
101	3.11E+08	2.194458	42	2.310715	185	23.78949	68.09503		74
115	56290274	1.526988	19	7.212429	156	12.24287	97.01033		99
114	21388541	2.166434	35	4.788058	147	21.46536	83.70084		88
135	29627848	1.975198	31	3.169966	205	18.53242	108.3275		111
82	2.05E+08	2.169535	42	2.372083	138	22.49788	48.17648		50
80	1.06E+09	1.87238	29	3.208423	198	16.83111	53.8624		55
145	9.74E+08	2.848118	85	1.997534	255	42.01922	77.66785		74
97	1.05E+09	2.100759	40	2.363892	154	21.2961	63.52209		65
89	6.47E+08	1.785486	25	662.8825	215	15.74104	65.63849		68
130	22289955	1.973694	21.5	8.465228	184	17.29185	102.9424		105
124	1.64E+08	2.115516	36	3.112814	175	20.692	89.82243		89
100	2.04E+09	1.814171	23	3.73517	164	15.25227	74.84083		74
99	75994706	2.256041	42	3.06439	144	23.31695	63.44427		66
81	2.19E+08	1.947763	32	4.898489	350	18.08541	51.78451		51
108	1.3E+08	1.856987	28	3.339284	148	17.10065	83.73284		88
122	4.75E+08	2.80931	66	2.531422	244	35.5654	64.68427		62
128	4.45E+08	2.303995	44	2.774889	175	27.70214	93.70586		105
107	1.94E+08	2.080782	31	5.292455	216	19.4264	77.57913		80
110	7.64E+08	1.927996	28	3.65781	226	17.58506	84.38597		87
85	1.31E+09	1.649876	22	3.426919	141	13.78005	63.01037		63
96	1.42E+08	1.95378	30	3.669471	139	18.0976	69.47026		72
101	5.95E+08	1.877725	27	3.863898	183	16.43262	75.14652		75
109	1.34E+08	2.313297	45	2.508744	148	25.16044	72.8528		77
99	13750481	2.007053	25	6.647705	167	17.76341	72.20186		74
124	4.05E+08	2.165354	35	3.359209	175	22.13288	93.07364		99
78	2.21E+08	2.033293	39	2.760953	141	21.52249	39.21479		32
123	8.22E+08	2.104603	30	19.96484	505	19.62919	94.17006		95
138	2.16E+08	2.258242	39	3.056309	177	24.0072	104.8068		112
141	7.16E+08	2.675729	60	2.404135	207	33.151	82.31031		76
135	83936236	2.00889	29	6.41524	274	18.59236	109.4044		113
91	2.79E+08	1.484726	19	4.039079	181	11.76716	71.9718		72
153	37590899	1.656564	23	3.01232	186	14.15331	130.0747		130
124	33007401	2.205868	37	2.884232	166	22.32693	82.52323		78
95	63279582	2.386169	49	2.473471	142	26.47993	56.54954		62
121	3.39E+08	1.890773	28	3.113433	171	16.80079	93.01365		93
132	74053762	2.103638	31	3.76515	190	20.14227	94.93321		91
140	9.45E+08	2.116163	29	4.33077	197	20.77013	111.326		117
85	58472863	2.046577	31	4.045898	131	19.10945	56.5764		59
141	1.57E+08	2.039032	31	3.67856	190	19.65222	113.1788		117
94	73342010	1.925922	27	8.506784	257	16.95291	65.68206		65
100	1.88E+08	1.593822	18	13.73373	152	13.71509	80.36336		83
126	83498712	1.567124	18	6.866177	183	12.26056	106.1982		106
138	1.64E+08	1.982416	26	6.289868	183	17.60068	109.5789		110
100	6.01E+08	1.972268	31	3.071338	154	18.86363	72.82429		77
93	1.07E+09	2.049324	34	2.763035	160	19.42289	63.23627		64
100	5.36E+08	2.147648	38	3.46541	363	21.74052	68.33708		72
125	1.58E+08	1.414022	17	7.467433	153	11.57585	107.4897		109
102	3.35E+08	1.910694	25	98.57517	143	27.89547	66.38445		77
74	2.97E+08	1.953349	37	2.669604	122	19.72288	40.11045		34
76	58046368	1.815688	26	5.118522	133	15.89829	51.81077		54
95	3.87E+08	1.495457	19	4.709036	157	12.07255	76.81139		78

91	1.27E+08	2.225335	38	3.156197	256	22.0295	55.82515	56
108	65299639	2.378574	45	2.813543	176	25.73686	62.63212	58
99	7.99E+08	1.881125	26	25.03393	563	16.80292	75.18106	78
117	1.21E+08	1.389196	18	3.889882	169	10.9179	99.07783	98
119	1.26E+08	1.965748	29	4.71955	176	17.74143	90.44099	91
140	1.68E+09	2.216834	36	3.193912	217	22.28235	107.1576	111
100	40413854	2.107234	38	2.52988	141	21.08169	68.69466	71
66	98319940	1.435414	18	6.16492	111	11.17041	49.49995	51
88	27665306	1.420734	17	4.307099	127	11.01463	70.9373	71
150	3.47E+08	1.7027	22	6.281738	190	14.1892	128.0445	129
136	4.68E+08	2.238561	34	3.287443	189	21.80417	100.9736	103
75	38911575	2.135761	32	4.519263	114	21.14504	44.84321	50
113	4.61E+08	2.284601	33	5.745407	374	23.48934	82.27566	89
132	1.02E+09	1.9391	27	4.622649	188	17.60785	105.7086	109
103	4.88E+08	1.871999	24	5.923802	205	17.49185	78.87656	84
142	2.84E+08	2.392366	42	3.220248	187	28.29265	104.6125	114
76	1.22E+08	2.034114	33	3.40681	138	19.05595	44.08755	42
100	1.65E+09	2.113366	40	2.436912	204	21.32981	67.62246	69
144.2	88644855	1.832476	23	5.89026	191	15.86716	119.9334	121
118	1.51E+08	1.870064	24	4.623399	178	16.11596	92.73871	93
107	46285228	2.360591	45	2.341959	156	25.33513	63.85188	62
131	3.82E+08	1.989915	26	6.312432	185	18.30365	104.591	108
162	3.56E+08	1.961279	27	5.930639	201	17.52483	134.3781	136
90	33158781	1.466811	16	19.65718	122	11.724	73.27484	75
107	8.7E+08	2.224266	42	2.686345	201	22.96008	71.041	71
128	2.39E+08	2.362305	49	2.69865	182	29.95171	91.01046	103
114	89649691	2.426298	56	1.995776	149	28.48843	69.69788	70
72	1.29E+08	1.416277	17	8.504763	124	10.96359	54.53002	55
92	11474995	1.971468	28	4.190239	164	17.54586	63.88063	65
125	86571801	2.30215	36	4.566231	226	22.80509	90.40849	93
114.9	6156499	1.970487	31	3.306811	140	18.76548	85.54604	87
141	94437474	2.091998	31	5.466716	218	21.14112	111.8316	118
94	2.17E+08	2.208741	39	3.337367	154	22.30937	61.20203	64
93	2.83E+08	1.860789	26	5.851109	131	16.65806	67.65052	70
102	30632270	1.809359	26	4.469882	140	15.73247	77.01679	78
112	12980390	1.446927	19	3.34025	142	11.54896	94.10769	94
155	1.08E+08	3.026484	74	2.53104	203	41.17542	92.3189	93
81	85307261	1.98797	31	3.885512	152	18.32429	53.15475	54
101	2.21E+08	1.587973	21	6.918575	129	13.208	80.05144	81
127	3.6E+08	2.285325	35	4.873502	175	24.20766	93.56186	100
122	1.52E+08	1.729107	22	7.447358	147	15.52923	100.1676	104
87	10022725	2.288813	31	5.299684	135	23.82571	52.61213	58
35	10137454	1.509341	18	6.519958	68	12.16491	16.60627	19
100	4821951	2.660095	46	3.059235	148	30.06028	49.78465	51
104	86759617	1.523946	19	8.054533	135	12.50345	86.73533	90
123	1.61E+08	2.498062	43	4.572921	185	27.07584	84.01395	89
106	50325678	2.190563	31	6.040009	150	21.67014	75.25636	80
99	3.38E+08	1.906159	33	3.969865	169	19.05737	74.41141	80
135	91013258	1.88058	25	4.892969	166	16.69154	110.5205	113
91	3.96E+08	1.78015	22	7.391609	138	14.99082	68.73117	71
116	96869584	2.168918	33	3.817831	166	20.89084	84.16181	87
87	27558972	2.352507	41	3.067097	155	23.97899	48.04149	48
91	4157295	2.044489	38	2.364153	119	20.61768	59.166	62
88	71274554	1.818347	25	8.227521	136	15.9054	63.26757	64
91	85269966	1.99859	29	5.35084	150	18.3796	64.2794	67
120	27611003	1.664695	24	3.118386	149	14.23204	98.3384	100
94	4.85E+08	2.166403	47	2.025715	137	23.79181	56.13017	54
97	39562289	2.185512	33	4.322337	138	21.35648	64.73726	68

102	2.21E+08	2.111713	33	3.64778	160	19.97108	71.75727	73
114	1.5E+08	2.105766	30	4.017216	159	20.54176	84.89913	90
105	39452817	2.008681	28.75	4.388624	143	18.65977	77.82815	81
106	70169902	1.7716	21	6.738994	145	15.59327	84.77225	89
94	1.84E+08	1.954849	27	6.80873	141	17.86266	67.57679	71
89	30600113	2.001325	29	5.088888	119	18.91515	61.86806	66
123	4.87E+08	2.167119	33	4.742932	168	21.1271	92.32236	96
90	50488774	2.496373	35	4.732796	156	26.44066	51.35603	56
73	1.85E+09	1.942001	25	146.6762	1256	17.79303	50.07678	49
84	72925628	2.016413	24	6.763877	128	19.9882	57.78737	64
93	25290112	2.229897	32	5.426945	145	22.97819	61.01172	67
96	2.13E+09	1.366693	15	11.73063	165	11.06458	80.04006	82
99	2.68E+08	2.117734	38	3.315491	145	21.2012	68.03426	71
90	2.38E+08	2.305413	47	2.689133	151	25.40505	46.89163	41
116	10138961	2.006218	33	2.888708	145	19.36312	86.85817	89
96	79731541	1.822282	26	5.097406	132	16.54509	72.09509	76
73	1.76E+08	1.954166	28	5.51786	149	17.34421	46.11097	47
87	1.11E+08	2.076827	33	3.616633	132	19.71861	56.46865	58
115	52063846	1.83044	23	7.737415	141	18.31167	91.49502	98
92	31865678	1.800477	25	3.876309	141	15.63047	66.89954	68
133	5.88E+08	2.564746	56	2.377082	193	30.63746	90.19209	97
115	6948074	1.845769	22	7.249887	137	17.56453	92.01311	97
80	3.37E+08	1.63933	23	3.405327	132	13.868	58.59269	59
101	1.78E+08	2.181939	43	2.240837	145	22.76648	67.64825	71
93.2	2078865	2.951791	81	2.093021	133	42.48313	32.80656	43
93	82441888	2.301385	37	3.76901	150	22.90716	55.58034	56
98	3.91E+08	2.059324	34	3.172559	155	19.76915	64.08479	62
80	73059775	2.023472	41	2.106131	118	20.78399	49.72999	52
111	1.76E+08	1.997249	26	7.120597	161	18.79847	84.80432	89
100	8096867	2.505303	55	2.839486	133	30.57479	58.91964	67
87	56008523	2.149147	32	6.156791	128	21.45743	57.10154	63
102	89388355	2.340861	32	3.949784	150	25.44212	68.28626	76
142	1.09E+08	2.52359	45	3.776858	198	27.65161	100.9028	105
116	71446924	3.007331	80	2.53409	189	43.36759	53.19764	56
90	1222673	2.35415	39	5.628435	113	25.69377	54.25424	58
71	39492099	1.29756	14	4.302708	116	9.325173	54.834	53
134	11382478	1.741947	25	2.975068	167	15.05521	110.2728	111
68	1.34E+08	1.322437	15	6.773665	148	9.775816	53.75001	54
104	9.14E+08	1.828809	24	3.893158	162	16.2054	80.28577	83
44	15463227	1.313379	17	4.809269	99	10.5032	28.10411	29
101	2.46E+08	2.036548	41	2.283527	174	21.25149	64.28397	59
65	21354902	1.21136	14	3.933572	105	8.731008	51.21834	51
76	21053307	1.836804	24	6.075137	120	15.98438	52.69457	56
81	60870472	1.202398	14	974.5118	142	10.24642	65.44066	66
85	1.38E+08	1.793938	19	11.60095	144	17.03442	62.17897	68
75	4.75E+08	1.780205	26	3.698391	130	15.49407	50.58132	51
92	2.57E+08	1.979755	31	3.448576	177	18.443	61.69116	60
69	15670913	1.923839	20.25	6.337469	124	16.64127	45.52317	49
60	17514296	1.183505	15	3.344018	92	9.351041	44.85286	44
98	22953188	1.574141	19	5.383452	133	12.62491	78.36466	79
60	2.04E+08	1.728941	22	12.64304	132	14.44597	37.32689	38
87	5675226	1.657074	23.5	2.785268	116	14.23015	62.02201	62
93	3.52E+08	1.851512	23	10.38605	191	15.92089	68.12715	69
95	8047458	1.301151	14	6.325955	149	9.326146	80.0719	79
100	32333148	2.278956	28	6.084176	163	22.02881	67.41597	71
88	45138915	2.773145	53	3.483841	156	37.35497	41.54345	56

46	87230093	1.546063	17	62.85033	205	14.90078	27.7623	33
108	1.22E+09	2.531883	37	5.561009	217	26.18488	65.98072	67
69	4.53E+08	1.532488	21	124.3016	130	13.32694	45.05109	43
89	8.3E+08	1.500683	15	246.7899	1465	15.44899	77.29825	72
104	3.3E+08	1.222563	15	5.500296	164	9.651649	89.1332	89
53	15641722	1.325985	18	3.823899	91	10.71889	36.72496	37
72	41216853	1.391326	16	6.595013	142	10.81791	55.96219	56
71	1.32E+08	1.333992	15	6.836903	148	9.747456	55.85322	54
120	45786243	1.71361	26	2.821183	164	15.4366	92.4529	90
71	18000262	1.433463	18	3.950899	129	11.21557	53.73876	54
118	1.22E+08	1.855856	27	6.923972	171	16.52222	92.46753	94
102	2.09E+08	1.429817	18	4.484321	152	11.31599	84.06822	84
161	61497846	2.227092	45	2.474541	222	24.57993	117.879	114
102	43657935	1.479738	18	5.289663	129	12.36192	83.48482	86
105	2.81E+08	1.267023	16	4.96972	154	10.09599	88.80556	89
82	11271348	1.442252	19	4.845679	146	11.7637	63.24737	62
78	43513733	1.140805	13	7.867081	152	9.575521	62.48514	60
140	1.48E+09	2.096947	34	3.611914	173	21.88407	110.5077	117
79	2.23E+08	1.658426	20	8.071195	176	13.71626	58.783	61
110	2.41E+08	1.608282	19	8.662786	149	13.35965	90.24291	93
77	1.18E+08	1.386934	16	8.863849	164	10.98083	61.24037	62
113.9756	6.96E+08	2.689051	61.00235	3.296909	191.0361	33.40102	58.79776	52.9733
76	1.81E+08	1.056087	13	8.049665	130	8.74914	62.43995	63
90.7	1022387	2.410425	50	3.067303	112	29.40224	53.70082	64
91	38326885	1.684586	20	8.317158	135	14.06041	70.0029	72
88.04272	3.75E+08	1.628187	20.02649	9.390382	129.0186	13.57339	68.53394	70.96945
113	7.87E+08	1.767631	21	7.397317	156	15.36294	91.89761	96
101	1.78E+08	2.637151	61	3.386682	144	47.16743	52.16159	78
91	8484582	2.029377	20	24.13303	381	19.96211	65.84471	63
90	7414320	2.910998	168	1.385482	115	75.69179	3.498227	38
91	1.47E+08	1.238121	14	17.19626	123	8.958784	77.12955	78
113	43812018	1.987048	22	13.74184	159	20.23675	85.10052	91

VX25	VX26	VX27	VX28	VX29	VX30	VX31	VX32	VX33
-62	223	12.3679	55.03861	0.479334	79715882	0.305815	528.5262	30.50405
-17	156	16.65265	47.9023	0.610196	79128548	0.272456	645.9508	9.597762
-127	266	6.757944	80.76227	-0.75164	3.99E+08	0.505287	167.0189	94.88873
61	87	7.065018	111.1486	-0.28371	19771707	0.503599	176.5672	8.834155
-10	152	12.56517	88.4416	-0.60033	86627885	0.308545	480.6809	24.68419
-73	202	9.879256	35.44513	0.459451	51209714	0.34605	422.6495	22.12852
-113	239	11.86574	54.65096	-0.67894	61816534	0.312664	506.81	56.74978
-85	220	13.58259	52.32801	-0.41534	1.15E+08	0.271286	708.0366	40.6757
2	109	9.620441	68.7823	-0.43716	8255340	0.376572	289.4084	10.37054
-102	296	12.91515	102.3923	-0.72966	1.08E+09	0.289302	620.8194	90.92135
-112	250	14.39704	55.58732	-0.32352	1.38E+09	0.278881	572.4253	57.38626
-29	216	10.10897	74.83499	0.168259	4.39E+09	0.342293	379.3232	29.43899
-70	235	13.20616	79.7056	-0.45483	3.59E+08	0.288558	559.9137	43.97978
-28	201	16.98092	108.0747	-0.53625	1.18E+08	0.241676	828.3939	45.2065
-43	173	8.69488	94.2469	-1.96285	34878069	0.386521	393.75	39.07726
-111	244	10.39831	67.01488	-0.88873	1.05E+09	0.346232	424.516	65.77647
-11	132	14.04356	73.04005	-0.56476	27924949	0.312482	530.5972	18.89452
-54	208	17.81785	55.05433	0.65885	2.37E+08	0.249411	816.3619	29.17259
-94	362	23.44729	123.4131	-0.33786	1.48E+09	0.162869	1945.939	87.14394
-58	218	21.37275	81.90633	-0.68644	2.02E+09	0.244429	1131.982	43.45563
-60	199	20.66192	63.7474	0.023559	1.45E+09	0.228159	839.2173	34.05824
-60	217	22.79685	80.3924	-0.29171	6.58E+08	0.195746	1220.788	41.90917
-72	223	10.17988	57.7661	0.082195	28838133	0.358077	449.2075	32.18463
-1006	1178	17.35776	70.48216	-9.60309	9.93E+08	0.243762	1009.414	1935.876
-94	244	10.72967	71.30102	-1.01082	4.14E+08	0.327156	497.922	52.56652
11	182	8.08153	81.98534	-0.44529	65728124	0.450156	271.7176	13.95117
-932	1148	5.723928	70.75603	-29.6188	1.77E+08	0.578821	684.4982	1692.634
-59	221	19.39748	83.31754	-0.25369	3.28E+08	0.213925	974.2064	43.70123
-57	199	10.01397	74.19456	-0.88763	1.24E+08	0.339276	459.7001	40.78222
-1024	1162	19.41198	96.37902	-12.0577	4.03E+08	0.223227	5555.407	1946.261
-74	227	11.22542	82.61612	-1.39869	70333476	0.326247	765.8786	44.97535
-38	196	13.41095	66.27187	-0.04667	5.59E+08	0.282204	584.8525	25.11204
-41	179	9.268528	69.6051	-0.62227	70053488	0.382257	321.0962	27.49119
-92	230	19.51277	64.92868	0.220681	3.58E+08	0.236699	828.3529	46.97246
-28	292	14.42848	55.27082	0.084079	2.97E+09	0.296914	474.143	21.08551
-28	154	7.946285	91.02906	-1.66763	80969755	0.411097	334.8771	38.0604
-291	871	24.05781	75.99465	0.185748	1.97E+09	0.182273	1366.023	231.225
-36	143	10.51365	35.36924	0.314669	12312409	0.338434	396.895	13.91608
2	125	6.976908	85.24948	-0.7627	1.49E+08	0.529377	177.3417	15.4023
-47	221	15.21625	103.4954	-1.42071	73907169	0.268606	1121.438	43.29977
-89	264	15.36765	79.23933	-0.79844	4.13E+08	0.255343	877.4641	57.23164
-87	287	25.34746	98.7477	-0.57555	4.88E+09	0.203224	1577.074	67.80119
-54	227	24.48213	74.59166	-0.02962	1.28E+08	0.178449	1410.156	39.32161
-65	264	13.25793	81.48623	-0.25758	1.2E+09	0.286132	583.5152	44.04668
-33	159	10.5198	64.69481	-0.36081	5.56E+08	0.355833	332.3331	25.11125
32	170	11.13486	137.1695	-0.1625	1.91E+08	0.329236	439.9688	24.98217
-95	233	11.36743	80.33771	-1.36532	97491407	0.332362	536.9478	59.26637
-1005	1189	21.57421	84.72515	-2.05039	6.33E+08	0.207901	1256.39	1983.564
-7	162	14.88669	88.22305	-0.59987	5E+08	0.267258	718.3434	23.99006
-91	269	15.70374	91.48972	-0.74909	9.05E+08	0.247232	895.7909	64.66391
-127	278	10.22534	85.33942	-1.7302	9.69E+08	0.362614	476.9719	97.58112
1	136	7.911118	97.27962	-1.46213	95589413	0.391084	364.7099	19.71108
-45	183	14.68286	77.58195	-0.66121	1.36E+08	0.282157	642.7925	30.23787
-110	281	11.54247	110.5904	-1.45678	3.82E+08	0.328537	519.3386	98.27029
-78	626	19.86077	79.23099	0.25285	1.28E+09	0.220158	1010.719	57.32399
-123	287	10.68742	101.6732	-2.5425	6.48E+08	0.356195	876.6487	90.61419
-52	236	15.76682	105.2627	-0.86759	3.67E+08	0.271924	870.4239	57.62864

-37	191	11.60882	87.69408	-0.90424	1.07E+08	0.308853	608.5514	35.96008
-25	179	14.04035	74.30389	-0.10307	56707524	0.279879	570.8878	18.81226
-109	337	12.36176	82.19317	-1.22448	1.77E+08	0.315365	698.7644	75.93783
-92	301	13.75235	72.95821	-0.07669	4.94E+09	0.277126	585.5288	53.28709
-3	168	15.29864	89.20807	-0.40537	84633021	0.255487	725.1376	23.69288
-72	189	8.734326	62.04659	-1.53498	3.94E+08	0.416658	371.0375	35.23767
-75	199	11.29034	61.20207	-0.5026	7.67E+08	0.343696	381.8572	34.46765
-49	234	18.03326	73.8105	-0.42841	1.37E+09	0.247541	811.056	27.98982
-16	172	7.978525	98.4472	-1.16023	1.31E+08	0.408387	280.8473	29.53063
-78	225	14.76434	88.17496	-0.97673	65927005	0.265517	768.9941	62.58724
30	175	12.94602	110.831	-0.23435	89965497	0.30155	548.6655	15.25883
-82	220	17.1715	55.17939	-0.09953	5.2E+08	0.246982	723.7914	41.73559
-103	301	12.02497	57.82118	-0.1049	2.78E+09	0.318863	442.13	58.9643
-62	317	34.14731	91.45389	0.314279	1.83E+09	0.149171	2331.518	45.3767
-56	210	16.18681	68.4643	0.082668	2.44E+09	0.260204	652.3037	37.20575
-1006	1221	10.78994	70.0237	-15.3488	1.33E+09	0.336758	594.907	1948.761
-47	231	9.975845	106.1284	-1.30564	47712139	0.339484	666.1048	44.93722
-74	249	15.13322	93.37818	-0.013	3.83E+08	0.263338	651.4146	51.2443
-60	224	9.982298	77.47207	0.147217	2.02E+09	0.337923	400.7711	42.27925
-83	227	17.31014	69.56875	-0.32944	1.68E+08	0.238158	814.6346	49.60924
-77	427	13.39799	56.437	0.32783	5.11E+08	0.299141	503.4987	43.33523
-1	149	11.71155	86.49737	-0.73796	3.03E+08	0.327294	470.6068	24.65282
-135	379	26.73015	77.51968	0.20494	2.22E+08	0.159479	1825.247	84.4541
-24	199	19.56847	99.69567	-0.84445	8.76E+08	0.254188	1158.438	28.73521
-90	306	12.9202	81.72004	-0.84465	72733707	0.287953	659.6446	59.29239
-55	281	12.12846	87.3212	-0.6391	1.87E+09	0.313696	504	48.15499
-44	185	9.44787	65.44238	0.091275	2.92E+09	0.385148	312.3995	25.5189
-70	209	12.61138	73.14689	-0.62895	5.29E+08	0.303901	524.3507	40.37659
-70	253	11.32974	78.06257	-0.12163	1.78E+08	0.325272	446.7652	42.85106
-37	185	18.78079	78.9202	-0.43433	3.37E+08	0.226058	920.8673	30.58794
-61	228	11.03434	76.28288	-0.70942	36099052	0.308091	605.969	40.956
-38	213	15.36013	97.19932	-0.77799	9.45E+08	0.269885	785.0065	40.38724
-95	236	16.19935	46.91483	0.616894	6.92E+08	0.276082	663.2011	37.2024
-19	524	12.75752	98.26549	1.728191	7.31E+08	0.28375	788.1058	28.33231
-18	195	16.78767	108.9957	-0.73604	3E+08	0.246847	895.5966	33.03942
-69	276	24.68631	91.49575	0.414587	5.79E+08	0.175104	1596.486	47.91917
-33	307	12.1276	112.2464	-0.92834	1.3E+08	0.308108	629.9358	49.04787
9	172	8.036091	73.58106	0.16946	5.02E+08	0.415977	234.2321	11.54064
66	120	10.13489	131.2845	-0.0913	44811844	0.368216	316.1884	14.113
-5	171	15.76134	87.12863	0.511933	34276386	0.25377	781.3152	23.4793
-57	199	19.69525	65.04217	-0.32544	71857704	0.218472	1032.634	34.10224
-1	172	11.92293	95.40356	0.057954	3.52E+08	0.316704	450.2999	27.78569
-6	196	13.40267	98.47831	0.664333	73425498	0.291721	685.6637	27.62121
-28	225	12.91718	114.764	-1.04311	1.18E+09	0.289213	777.2991	49.72777
-99	230	13.18414	61.64533	-0.61697	2.02E+08	0.290459	599.2585	46.47954
-17	207	13.5333	115.9325	-0.79491	1.78E+08	0.290107	630.909	37.70902
-124	381	11.20616	69.59838	-0.25863	1.8E+08	0.328358	529.802	66.18837
-78	230	7.694875	83.21	-2.4842	4.33E+08	0.454106	465.6352	60.74759
-6	189	7.703056	107.5776	-0.70016	99538221	0.426208	294.8758	33.59804
-64	247	11.37529	112.1847	-0.90529	3.58E+08	0.318625	577.853	62.91275
-38	192	13.20222	76.57693	-0.62391	6.24E+08	0.304514	560.6494	30.09071
-28	188	13.90243	67.65941	-0.00814	1.11E+09	0.277022	578.9694	25.59222
-61	424	15.86176	73.3692	-0.32766	1.27E+09	0.260088	713.0824	39.79632
-13	166	7.334645	108.6952	-1.38764	1.74E+08	0.491101	260.6023	33.95896
-1024	1167	10.47099	123.9572	-9.66684	3.48E+08	0.33134	10958.48	1982.571
-164	286	15.12322	46.4178	0.432485	1.12E+09	0.282817	545.7645	82.82228
-68	201	10.73155	55.87739	-0.83111	79915822	0.348288	437.9275	31.80464
-71	228	8.184403	78.42041	-0.59903	4.01E+08	0.421639	249.7712	43.66936

-100	356	15.76977	62.21698	0.030493	57757282	0.24697	754.506	46.16556
-26	202	18.62821	70.20432	0.512416	75781249	0.221333	1005.864	24.9865
-83	646	11.11335	78.70512	1.053712	1.87E+09	0.337895	542.3037	57.63657
40	129	7.63941	100.0637	0.241413	1.03E+08	0.435503	196.3196	11.89248
-67	243	12.34293	93.28217	-0.33857	2.66E+08	0.304822	521.9905	49.97756
-16	233	15.35638	110.8005	-0.5951	1.63E+09	0.253162	794.0174	34.05983
-21	162	15.61775	73.27959	-0.29156	49992462	0.2636	650.9421	17.39187
-73	184	7.476058	51.66359	-0.77238	2.3E+08	0.427726	218.8818	30.50921
-18	145	7.335203	72.39222	-0.29429	64640045	0.433937	208.5335	18.68991
-29	219	9.338523	129.4495	-0.88649	3.6E+08	0.371756	361.7993	58.71256
-17	206	14.72693	104.8227	-0.34244	4.86E+08	0.25475	792.1215	31.71161
-87	201	13.65087	52.84092	-1.09587	99963400	0.28115	781.2487	41.79195
-95	469	14.84046	88.12334	-0.48414	4.78E+08	0.263968	996.4385	61.62159
-31	219	11.77852	108.2025	-0.87091	1.06E+09	0.319613	533.4531	46.07597
-72	277	10.52101	82.55666	-1.44666	1.46E+09	0.35982	594.0894	45.24466
-36	223	19.0954	110.6076	-0.95686	3.44E+08	0.235113	1290.254	46.68933
-88	226	13.47323	50.1729	0.134439	3.55E+08	0.286555	573.607	39.55544
-44	248	16.00925	72.30398	0.00833	3.44E+09	0.259142	655.0688	27.80449
23	168	9.764822	121.9664	-1.03287	99564342	0.348915	491.7786	27.73472
-49	227	10.55235	95.16966	-0.33564	1.77E+08	0.334418	456.7963	38.58709
-17	173	18.82905	70.84491	0.149122	48064685	0.215324	941.939	16.41909
-101	286	11.44231	107.5598	-1.24456	4.7E+08	0.321273	629.8321	95.37763
-56	257	11.42231	136.4415	-0.96669	3.7E+08	0.317898	558.7863	80.19426
-82	204	6.954407	75.56547	-2.98972	1.09E+08	0.432639	340.9374	56.40221
-126	327	17.27972	76.26094	-0.00849	9.03E+08	0.238546	768.908	88.79727
-17	199	21.55085	98.07039	-0.84071	3.11E+08	0.241943	1334.899	26.97512
-35	184	22.67824	77.247	-0.06576	2.29E+08	0.199295	1109.304	28.7277
-88	212	7.358741	56.51474	-0.78762	3.5E+08	0.44893	220.3928	45.38984
-38	202	11.72905	67.91271	-0.31364	42547970	0.310864	531.4017	26.18018
-80	306	15.19143	95.23581	-0.5609	4E+08	0.248339	896.1643	68.77479
-3	143	13.27518	88.72858	-0.51902	25837640	0.29731	554.6366	25.24791
-41	259	13.55601	115.3952	-1.36955	2.85E+08	0.291815	809.7407	51.04284
-81	235	16.16515	67.14138	-0.47007	7.43E+08	0.249756	762.2764	49.17767
-92	223	11.37284	71.10968	-1.04059	1.05E+09	0.330166	479.9938	53.19992
-28	168	10.8287	79.69498	-0.59854	75720638	0.340827	419.703	31.60042
42	100	7.818243	95.27432	-0.15311	42426985	0.428569	220.9381	11.03215
-75	278	30.05843	105.0742	-0.26719	3.55E+08	0.136926	2517.805	54.73053
-52	204	13.08699	57.99414	-0.28922	2.94E+08	0.300703	537.8934	31.7593
-132	261	9.21302	81.86515	-0.76798	8.2E+08	0.394638	293.6706	94.59302
-73	248	15.52309	98.94753	-1.25224	4.93E+08	0.257864	1036.792	55.09928
-45	192	9.851262	102.417	-1.59985	5.65E+08	0.385434	455.6965	43.60471
-88	223	13.82954	62.41271	-1.32825	45194811	0.265801	1127.31	44.25501
-93	161	7.676228	23.65034	-1.30107	53877186	0.426649	283.5705	27.36141
-71	219	20.3095	63.07541	-0.17192	17879243	0.188925	1499.996	31.70616
-56	191	7.838954	88.43706	-1.56041	2.99E+08	0.443618	298.0967	49.54312
-117	302	18.26424	91.0497	-0.81857	5.51E+08	0.215337	1231.704	80.52915
-82	232	13.25003	81.02868	-1.37108	1.51E+08	0.281368	902.1265	58.07761
-60	229	13.87335	78.02888	-0.91031	1.25E+09	0.325571	551.4476	42.84689
-4	170	10.80192	112.7525	-0.96181	3.9E+08	0.338401	498.3516	36.662
-212	350	9.597283	71.70813	-1.0576	8.23E+08	0.354671	418.0822	151.2256
-48	214	14.30904	88.35026	-0.57305	3.59E+08	0.266445	722.5588	35.4251
-74	229	16.97817	56.67122	-0.13356	1.17E+08	0.22805	903.6426	30.94283
-8	127	15.2251	64.28452	-0.244	15414775	0.271893	631.884	15.49611
-94	230	10.66239	66.85406	-1.17101	1.37E+08	0.346822	466.6788	50.19457
-101	251	12.25503	68.71304	-0.97326	3.5E+08	0.303751	589.6405	66.41623
21	128	10.05838	99.96562	-0.35946	75248108	0.365049	322.6835	19.91618
-96	233	18.98657	62.52331	0.163968	1.66E+09	0.243483	758.5688	46.46471
-82	220	14.10989	70.55684	-0.90588	1.78E+08	0.268225	787.3544	52.1762

-73	233	13.75525	76.12545	-0.38608	9.03E+08	0.275103	645.9784	41.60071
-52	211	13.16279	89.11256	-0.94788	4.49E+08	0.287503	733.1849	49.14563
-39	182	12.15733	81.64912	-0.9206	2.2E+08	0.309055	609.3583	32.70196
-47	192	9.575914	87.48591	-1.51022	2.6E+08	0.38013	467.4505	35.58249
-113	254	11.57417	71.82656	-1.33261	6.83E+08	0.317973	592.4324	69.11206
-63	182	12.28448	66.79303	-1.17929	2E+08	0.30531	633.6518	37.58872
-77	245	14.11098	96.36435	-0.91176	1.23E+09	0.27123	762.6697	68.95231
-103	259	15.50359	63.2235	-0.94254	2.03E+08	0.232834	1359.769	59.58305
-145	1401	10.63252	60.16742	7.861865	6.67E+09	0.335844	1112.435	73.97652
-105	233	11.08294	64.56291	-1.70118	2.7E+08	0.334571	828.9898	63.77131
-86	231	14.19905	68.58124	-1.33996	95868392	0.269951	980.9575	50.14732
-164	329	6.475195	81.71132	-1.90649	4.85E+09	0.513156	270.3287	115.9585
-72	217	15.66835	72.87974	-0.53955	7.86E+08	0.267665	682.7948	39.42435
-102	253	19.41108	55.84435	0.145692	1.06E+09	0.228452	919.7662	55.27937
-2	147	13.9903	90.13416	-0.49411	37594109	0.287805	579.8252	23.26004
-63	195	11.14432	75.30208	-1.10385	3.14E+08	0.345858	472.7019	41.23909
-90	239	11.47473	51.70743	-0.74238	6.62E+08	0.320275	547.4362	41.25184
-69	201	13.91345	61.71256	-0.51399	5.22E+08	0.277812	619.7317	34.18174
-48	189	9.913274	95.37977	-2.01784	1.7E+08	0.359825	725.961	41.64114
-32	173	10.77062	69.85621	-0.46342	1.18E+08	0.343051	404.3409	27.18636
-161	354	22.98389	97.42205	-0.33001	1.08E+09	0.191162	1356.443	124.887
-23	160	10.02873	95.42671	-1.81596	25762665	0.349405	639.8452	27.75631
-39	171	9.832031	61.1635	-0.23904	1.07E+09	0.383016	307.8698	23.75632
-40	185	17.2648	72.9078	-0.21926	6.6E+08	0.245628	739.2618	27.40704
-79	212	32.64282	59.92028	-0.32611	11514709	0.138972	2514.17	37.43415
-79	229	15.63075	63.02496	-0.52197	3.23E+08	0.242835	882.9715	46.12394
-76	231	14.08878	68.67492	0.200034	1.02E+09	0.277915	609.3835	50.2915
-50	168	16.36909	55.31581	-0.12182	2.17E+08	0.266163	586.7669	20.31209
-89	250	11.2839	88.85629	-1.57728	6.53E+08	0.321696	703.6674	64.68523
-61	194	22.72722	69.42308	-0.66858	17747861	0.207157	1348.039	36.57279
-103	231	13.71839	64.07961	-1.41976	2.36E+08	0.280701	845.6109	62.15601
-63	213	15.67999	76.25126	-0.99528	2.05E+08	0.260524	1151.241	41.423
-66	264	19.15091	106.8579	-0.67198	4.16E+08	0.206384	1237.236	58.28566
-111	300	32.61474	74.0519	-0.37327	2.2E+08	0.141454	2653.696	63.34978
-81	194	16.76824	64.37899	-1.36614	3638602	0.238679	1201.132	46.37077
11	105	6.260718	56.18129	0.782177	73324564	0.460551	149.5703	7.217496
49	118	10.56676	111.9016	-0.14556	23930020	0.355803	361.8816	15.91637
-30	178	6.213953	55.43695	0.357562	2.34E+08	0.477341	184.1921	21.96783
-62	224	10.67493	83.05328	-0.65807	1.71E+09	0.35448	452.0428	45.8317
-59	158	7.163636	31.30277	-0.58947	37169517	0.478983	190.0224	22.04529
-46	220	16.6362	68.99731	0.335807	5.36E+08	0.268801	628.1993	26.44133
-5	110	5.962531	52.44861	-0.04208	51621148	0.473529	127.5382	12.8131
-88	208	10.09878	57.07463	-1.24105	66518006	0.35892	480.7955	45.09328
-1000	1142	6.143498	72.35205	-28.1962	1.08E+08	0.562363	952.3387	1859.714
-127	271	8.538949	67.92051	-2.49821	3.46E+08	0.391754	746.9707	83.24984
-101	231	10.8395	54.27053	-0.34148	1.46E+09	0.343417	386.8212	57.52015
-56	233	13.036	65.91315	0.366043	1.85E+08	0.296321	538.7443	35.27145
-85	209	9.392817	51.48491	-1.22571	39820812	0.334544	578.3364	41.0795
-2	94	6.568486	46.39996	0.267731	54793976	0.509886	141.177	10.6006
-4	137	8.143906	80.24015	-0.82141	53630171	0.412662	297.4625	22.00737
-320	452	9.243881	42.51754	-1.2873	7.55E+08	0.387904	414.4448	225.6758
14	102	10.11041	64.52732	0.206287	21043090	0.370712	317.0457	8.936353
-114	305	9.818169	71.98707	-1.30999	7.89E+08	0.357668	540.8297	68.75256
24	125	6.204096	81.08465	0.671417	17793854	0.478924	163.2122	13.67198
-88	251	12.67906	74.50339	-1.0941	1.03E+08	0.272021	1005.843	52.97717
-115	271	24.10888	63.46167	-0.92244	1.67E+08	0.198997	2301.525	54.36937

-656	861	7.10376	39.07382	-4.77652	3.88E+08	0.49469	756.0182	821.8634
-127	344	16.13634	75.39953	-0.45687	2.85E+09	0.224075	1331.634	86.29113
-741	871	9.20403	48.59599	-3.29176	8.96E+08	0.431425	331.9693	1044.03
-72	1537	6.741292	98.00664	14.46872	1.94E+09	0.439409	3630.282	47.74616
-37	201	6.447402	90.06017	-0.05794	7.71E+08	0.549798	166.1077	36.89659
-25	116	7.184622	39.2679	-0.26378	35510177	0.500321	193.2451	9.079869
-53	195	6.938447	57.89222	-0.31357	84062135	0.474236	219.742	33.28071
-39	187	6.131839	57.46159	1.107274	3.18E+08	0.469183	182.2524	22.52444
40	124	11.21405	94.39967	0.435892	69448570	0.358734	363.7581	9.945882
-21	150	7.610237	55.6946	0.06636	48023313	0.438468	214.0351	13.8078
-51	222	11.16363	95.11293	-1.11075	1.27E+08	0.339481	496.2253	52.88366
-2	154	7.360789	85.4112	-0.0725	1.79E+08	0.468888	227.6068	23.82561
44	178	18.82205	121.4838	0.467372	39425103	0.240805	862.832	16.91401
-39	168	8.003702	85.11008	-1.02879	1.04E+08	0.458408	274.0108	35.36383
-17	171	7.010821	89.76704	-0.3808	4.88E+08	0.517448	171.6946	25.94752
9	137	8.02816	65.09492	0.673524	22568150	0.461816	237.1193	9.221035
16	136	5.718723	63.98034	1.664432	74696273	0.605738	189.0915	9.041407
-51	224	14.85508	113.974	-0.99315	3.82E+09	0.280574	778.1244	64.61316
-63	239	8.29692	62.05189	-1.04689	2.52E+08	0.427293	394.995	35.20911
-60	209	8.085005	92.27866	-1.70487	5.63E+08	0.413758	371.5689	51.88102
-86	250	6.891936	63.22622	-0.39368	3.39E+08	0.513464	247.1718	48.82199
-132.895	323.9308	24.62076	71.75598	-0.20625	1.46E+09	0.1854	1691.743	80.10333
-45	175	5.649832	63.63129	-1.1962	5.62E+08	0.631145	150.1928	25.30734
-53	165	20.61888	64.73106	-0.92634	3961250	0.229542	1306.333	32.38086
-68	203	8.748206	72.80355	-1.57054	1.86E+08	0.379961	399.9509	40.87769
-97.9175	226.9361	8.603142	71.11711	-1.6936	1.15E+09	0.387681	360.7418	53.73807
-102	258	9.342969	94.34798	-1.53054	2.16E+09	0.357183	456.3693	84.93722
-129	273	31.09742	78.56434	-1.28052	4.38E+08	0.239804	3451.524	82.20627
-62	443	8.909294	75.35983	3.077321	28819433	0.379971	1343.578	38.35712
-132	247	67.98108	81.07391	-0.33747	20961525	0.161233	6560.741	53.07529
-72	195	5.813275	78.19047	-1.8939	2.58E+08	0.491268	164.7831	44.50219
-117	276	9.783816	91.76345	-2.82786	98313143	0.353884	1178.432	81.3965

VX34	VX35	VX36	VX37	VX38	VX39	VX40	VX41	VX42
23.1588	2.006122	2.445764	0.936947	0.438509	0.659765	1.369826	0.460631	0.711299
29.77981	3.930069	3.313422	0.757873	0.617574	0.543496	1.259445	0.416449	0.760635
3.876449	-0.52266	0.845863	0.40522	0.345672	0.374407	1.007479	0.258029	0.817612
2.371053	-0.02563	0.829427	0.435083	0.308816	0.402836	1.039962	0.266311	0.803957
17.21151	-2.0184	2.395943	0.953112	0.431662	0.668926	1.38485	0.465308	0.708611
16.28053	1.634154	1.959451	0.773976	0.426232	0.57469	1.299484	0.417132	0.742695
22.31886	-0.72896	2.234543	1.058677	0.354131	0.725995	1.457548	0.511624	0.685755
35.81291	-0.19735	3.116804	1.130139	0.457351	0.718445	1.473954	0.574786	0.696596
5.437175	-0.33884	1.396898	0.621157	0.383327	0.529223	1.191286	0.331341	0.75052
32.72486	-2.6656	2.877721	1.169546	0.417771	0.7459	1.506752	0.579938	0.686401
26.85064	-0.77367	3.035887	0.75158	0.599238	0.580057	1.286786	0.396767	0.736123
16.39843	0.539415	2.211952	0.522213	0.616876	0.450975	1.115464	0.304735	0.785898
26.54281	-1.18237	2.99574	0.782212	0.584558	0.593183	1.299405	0.404869	0.732675
54.82912	-4.04844	4.614587	1.217754	0.585907	0.773995	1.510669	0.570441	0.677044
15.55601	-1.97693	1.455793	0.802894	0.266365	0.575848	1.288148	0.447616	0.741967
16.75137	-1.52071	2.019505	0.708324	0.469974	0.51957	1.246645	0.418015	0.765846
21.23858	-2.66889	2.855768	0.808558	0.559583	0.593003	1.314714	0.426218	0.736765
57.39121	7.066295	4.400045	0.972551	0.634144	0.662565	1.403316	0.496111	0.713894
314.0609	-7.99681	9.676972	2.441482	0.591377	1.092522	1.859861	1.10366	0.612658
110.7541	-11.9885	6.941119	0.861102	0.780553	0.604591	1.363609	0.4721	0.735471
47.53193	0.509629	5.011605	0.753839	0.738581	0.57683	1.280946	0.398196	0.738458
102.9869	-5.33453	7.002964	1.341667	0.680216	0.806448	1.570241	0.641482	0.67145
14.748	0.170873	1.794554	0.666906	0.438062	0.500115	1.22674	0.398986	0.773114
3170.373	-36.421	4.557419	0.752986	0.697908	0.570333	1.260195	0.388803	0.743477
27.42674	-2.28002	2.387764	0.714519	0.531166	0.52652	1.254445	0.414995	0.762825
9.343625	-0.99105	1.415521	0.673919	0.362248	0.500192	1.240013	0.406007	0.774635
3957.436	-51.1307	1.403438	0.343723	0.434505	0.312117	0.938741	0.242106	0.848303
69.6443	-2.53111	5.50167	1.164388	0.651776	0.734528	1.469138	0.554734	0.695074
23.1088	-1.491	2.228455	0.822479	0.456356	0.583701	1.311636	0.458436	0.740066
56063.03	-652.462	12.56135	1.234011	0.637431	0.758868	1.511761	0.603051	0.685842
67.29425	-6.64604	3.002038	1.13545	0.427838	0.692674	1.480523	0.615939	0.712337
31.45703	-0.39274	3.268166	0.74987	0.626023	0.576125	1.277974	0.393164	0.738974
9.257411	-0.93162	1.4786	0.708839	0.346071	0.574601	1.260982	0.367547	0.73421
47.47682	2.999444	4.55243	0.944106	0.655148	0.644912	1.369169	0.473838	0.722675
18.6069	0.396866	2.826568	0.526262	0.686164	0.45983	1.123712	0.305751	0.78058
12.94308	-1.98721	1.539145	0.557114	0.449484	0.48238	1.137755	0.311891	0.770682
157.4133	4.118319	7.997228	0.993216	0.778961	0.705184	1.443344	0.49	0.690459
11.18253	0.968555	1.771235	0.812118	0.363676	0.612086	1.30661	0.406687	0.725129
2.708494	-0.33617	0.776894	0.498279	0.214533	0.427877	1.120341	0.310561	0.797133
91.07305	-9.44017	4.039055	2.138533	0.287002	0.942923	1.764261	1.124683	0.663555
58.3918	-4.54529	3.789449	1.280677	0.475535	0.722084	1.522526	0.7069	0.707861
203.3632	-16.6541	9.441342	1.029811	0.80359	0.669594	1.436796	0.547511	0.714601
114.9971	-0.8848	7.158465	1.846521	0.588999	0.950544	1.698824	0.826658	0.640836
31.30563	-1.22301	3.176153	0.735766	0.620762	0.554663	1.257712	0.395095	0.74991
10.04811	-0.654	1.929026	0.525571	0.571644	0.462066	1.123713	0.303212	0.779247
16.25735	0.225898	2.21832	0.786735	0.474614	0.592804	1.285312	0.403953	0.732596
33.08119	-3.5258	2.380163	0.867097	0.450635	0.591332	1.323056	0.478097	0.741633
370.7257	-9.89265	6.676171	1.349754	0.663219	0.791392	1.539279	0.63326	0.683308
52.12774	-4.82946	4.31986	0.76628	0.700506	0.58811	1.285988	0.39425	0.734058
82.84147	-5.63251	4.850821	0.837289	0.70205	0.59589	1.325581	0.450064	0.73652
36.4267	-2.89164	1.931894	0.76115	0.407442	0.561297	1.296279	0.424128	0.748554
9.405968	-1.6813	1.382482	0.918549	0.18259	0.597361	1.366777	0.52828	0.745357
29.26364	-2.40083	3.19482	1.043865	0.507608	0.699987	1.444704	0.518923	0.700334
34.71297	-3.41114	2.409563	0.766662	0.505485	0.558341	1.289487	0.428864	0.750482
145.7919	1.878802	5.168643	1.171554	0.62618	0.676746	1.47331	0.67255	0.720135
135.779	-12.8693	3.314749	1.097325	0.463482	0.631544	1.435539	0.658616	0.73962
71.62925	-8.33839	4.802382	1.229644	0.595932	0.741062	1.513194	0.616386	0.697361

30.00549	-2.24912	2.549011	1.242836	0.327494	0.745942	1.527594	0.645797	0.692983
22.94255	0.604876	2.796726	0.773633	0.561208	0.59039	1.298657	0.401651	0.733416
53.65426	-5.1264	3.15593	1.063013	0.486131	0.670094	1.449881	0.56834	0.71912
33.48749	-0.91611	3.453821	0.639251	0.687872	0.514988	1.196831	0.349184	0.762008
44.68862	-3.09951	4.012694	1.224612	0.538848	0.764509	1.488383	0.56547	0.685291
16.94064	-2.07776	1.557516	0.703244	0.354356	0.514962	1.251432	0.421065	0.768548
13.66147	-1.3908	2.141559	0.614817	0.553657	0.512439	1.194969	0.340015	0.759961
54.63048	-4.68718	4.8408	0.822539	0.71046	0.611805	1.337553	0.428064	0.726553
7.519106	-0.89577	1.170856	0.66047	0.267313	0.52513	1.231243	0.372489	0.757556
34.69666	-3.11314	3.037297	1.612875	0.29728	0.883992	1.651047	0.751604	0.654581
21.36019	-0.83367	2.65507	0.784005	0.538492	0.597338	1.300587	0.401094	0.731119
36.44434	-0.2885	4.022269	0.89531	0.636109	0.651467	1.376452	0.450479	0.711527
17.14877	-0.31179	2.376934	0.69375	0.546669	0.561445	1.249095	0.365298	0.740283
376.478	16.99847	13.72689	1.324001	0.822908	0.775157	1.534489	0.643318	0.687696
33.04501	0.683458	3.766259	0.646347	0.706727	0.509101	1.227242	0.372125	0.766398
2098.23	-25.8038	2.540807	0.562206	0.616867	0.478185	1.145877	0.319365	0.773964
44.43503	-3.93162	2.228523	0.992012	0.32572	0.639752	1.413646	0.542586	0.728731
27.17707	0.533459	3.147658	0.937057	0.534895	0.665801	1.378643	0.457521	0.708182
18.08684	0.409956	2.280456	0.51584	0.627589	0.446686	1.094986	0.296641	0.787752
47.72159	-1.5364	4.214312	1.056979	0.5968	0.718105	1.443137	0.503462	0.691765
27.47029	1.190704	2.653066	0.783622	0.541729	0.582232	1.291714	0.422536	0.737001
16.91708	-2.00244	2.247296	0.737569	0.494247	0.567785	1.266329	0.385925	0.743141
236.3187	7.92815	10.02482	1.713853	0.707572	0.97197	1.735154	0.754987	0.617131
130.3649	-14.8078	6.714117	1.205963	0.697565	0.715506	1.503363	0.628677	0.710326
38.74853	-3.18494	2.966454	1.066484	0.470027	0.732401	1.480263	0.523167	0.682743
27.14305	-2.75038	2.776324	0.709742	0.59184	0.558249	1.2598	0.380345	0.744337
9.823587	0.286266	1.687182	0.587775	0.482081	0.494562	1.173204	0.329875	0.767634
21.31496	-2.09135	2.587903	0.961154	0.457324	0.686698	1.418375	0.476183	0.697866
16.41672	-0.19097	2.142068	0.838502	0.436409	0.64218	1.352524	0.419815	0.709567
56.34018	-3.36855	4.845627	1.216429	0.599102	0.734723	1.51141	0.630385	0.696299
22.93765	-1.14694	2.189374	0.845349	0.413287	0.634794	1.347021	0.426362	0.715169
47.79133	-5.49382	3.818459	1.095791	0.549435	0.697306	1.465846	0.56682	0.706892
36.38973	4.727318	3.676017	0.882307	0.611214	0.623105	1.375892	0.469645	0.727201
255.4467	10.21545	4.106077	1.155818	0.556669	0.630598	1.359054	0.696478	0.736165
76.5603	-7.40678	4.986054	1.088397	0.643006	0.712627	1.438686	0.518982	0.699654
191.9919	11.6749	8.677216	1.889049	0.64343	0.93559	1.669012	0.842317	0.654009
44.09304	-3.52635	2.834838	1.021138	0.462974	0.680924	1.438054	0.542498	0.704688
6.385106	0.196293	1.321301	0.461247	0.479771	0.417148	1.058351	0.277069	0.79844
6.637644	-0.08126	1.520022	0.839192	0.289509	0.63892	1.304465	0.398007	0.712525
30.92915	1.583401	3.181361	1.647156	0.301282	0.874955	1.61192	0.751814	0.665086
70.20816	-4.27256	5.424572	1.1158	0.655315	0.719237	1.462122	0.545778	0.697372
15.92292	0.587401	2.236331	0.93192	0.411709	0.654771	1.346656	0.445554	0.715202
40.45884	3.842516	3.191701	0.940627	0.529622	0.634378	1.346598	0.470429	0.728377
82.4574	-10.0638	4.135691	0.964294	0.619851	0.644388	1.395788	0.506759	0.723668
25.41406	-1.41947	2.73785	0.905716	0.493839	0.634492	1.375651	0.481519	0.720768
30.93804	-3.34478	2.984462	1.00694	0.490013	0.666946	1.401617	0.508725	0.715269
33.06861	0.636578	2.238704	0.944677	0.389205	0.663253	1.402017	0.487701	0.708509
43.07914	-4.98114	1.771467	0.887749	0.296355	0.533791	1.303001	0.581351	0.772333
8.691675	-0.70782	1.291721	0.643589	0.323446	0.506526	1.210007	0.36977	0.766773
31.52646	-2.09601	2.500207	0.9915	0.418858	0.660727	1.427599	0.525663	0.7163
29.62665	-3.57594	3.110257	0.6772	0.64067	0.532118	1.23026	0.370856	0.756251
28.17625	-0.28755	3.196434	0.725474	0.627735	0.561788	1.247972	0.377245	0.744833
45.68583	-2.77581	3.918761	0.905122	0.624756	0.62743	1.370163	0.481356	0.725566
9.21201	-1.50108	1.245899	0.577851	0.364834	0.453229	1.173463	0.358925	0.791724
137345.4	-1607.1	21.15295	0.750633	0.51945	0.542878	1.254064	0.416365	0.758253
25.50485	3.078907	3.173214	0.612321	0.675407	0.511253	1.197181	0.341504	0.760151
16.0675	-1.57796	1.847652	0.846685	0.355154	0.610073	1.342404	0.448001	0.729898
6.45763	-0.78887	1.222013	0.589464	0.343223	0.499388	1.168359	0.32584	0.764634

47.00556	0.188783	4.003122	1.107715	0.566581	0.76223	1.494668	0.518939	0.671103
63.91416	4.072912	4.691272	1.550311	0.494192	0.855901	1.591487	0.7006	0.666801
164.7755	4.858432	2.689449	0.900544	0.49636	0.598501	1.352542	0.526649	0.7365
3.408876	0.242344	0.987142	0.514821	0.304543	0.456539	1.098255	0.292053	0.781192
21.51082	-0.46504	2.337017	1.024924	0.380748	0.722891	1.439965	0.482963	0.684818
67.30996	-5.94271	4.667351	0.788514	0.711747	0.574468	1.270899	0.409795	0.745148
27.40911	-0.95329	3.265621	1.321952	0.427369	0.821372	1.488726	0.547163	0.664425
5.430309	-0.58233	1.157522	0.509204	0.386546	0.452413	1.107098	0.298443	0.78241
3.326571	-0.0176	0.980417	0.5805	0.250428	0.502522	1.160079	0.316789	0.761547
13.97877	-1.23491	1.627755	0.687542	0.391703	0.538454	1.234832	0.374	0.753424
56.61229	-2.05206	4.079051	1.154078	0.556537	0.713552	1.450581	0.566688	0.704913
41.22907	-4.29432	3.000906	1.557304	0.293524	0.814526	1.624698	0.833979	0.682664
139.2732	-7.09131	5.4751	0.990379	0.691741	0.616877	1.376143	0.561147	0.738309
27.28969	-2.56097	2.602912	0.802628	0.51839	0.553279	1.282804	0.453646	0.757659
64.26147	-8.68104	3.146334	0.797187	0.595017	0.564573	1.332318	0.4636	0.750527
177.6689	-19.7317	7.437535	1.338595	0.697949	0.747983	1.549506	0.712941	0.702502
24.77167	1.601098	2.807711	0.855146	0.527714	0.639884	1.35653	0.433652	0.71264
35.46077	0.14258	3.848313	0.612024	0.724999	0.490438	1.188915	0.352122	0.77335
27.87795	-3.103	2.338596	1.046702	0.378328	0.662879	1.421822	0.546803	0.723514
17.12559	-0.63152	2.059157	0.798736	0.425542	0.586046	1.281201	0.409192	0.739217
58.4649	3.008072	4.7512	1.463551	0.527103	0.851982	1.561231	0.634381	0.660771
39.9251	-4.26215	2.757575	1.018649	0.450426	0.648535	1.417984	0.562433	0.723984
30.21749	-2.45472	2.335811	1.064708	0.356792	0.642675	1.417267	0.595578	0.733396
29.6409	-3.54034	1.346578	0.751892	0.270889	0.55853	1.259506	0.433142	0.744451
44.92627	0.110028	4.188222	1.026385	0.606043	0.673763	1.400306	0.507963	0.714315
164.8991	-17.8112	8.114368	1.121652	0.759319	0.689319	1.453286	0.575039	0.717609
75.64837	0.159466	6.098952	1.436386	0.620365	0.849205	1.580611	0.635828	0.65938
5.275473	-0.24283	1.027563	0.543011	0.302511	0.470024	1.142252	0.316381	0.776012
16.08044	-0.59945	1.878628	0.986794	0.278978	0.660603	1.409612	0.51056	0.716755
62.34101	-2.81396	3.756406	1.704237	0.366552	0.917967	1.686139	0.801642	0.641424
17.70127	-1.82007	2.369826	0.945641	0.424369	0.678268	1.410716	0.473462	0.70115
59.05675	-7.2226	3.287945	1.428834	0.370712	0.758727	1.563742	0.782182	0.702072
43.05144	-2.19389	3.905991	0.982914	0.592633	0.641524	1.382269	0.532276	0.723585
21.31579	-2.13838	2.128448	0.924296	0.385413	0.641535	1.387816	0.493696	0.718523
10.13014	-0.78128	1.58357	1.044803	0.193499	0.732105	1.454306	0.495351	0.681053
2.927882	0.019055	0.940854	0.627312	0.186449	0.506531	1.202973	0.352211	0.76621
351.2737	-3.97169	12.14762	2.299101	0.668297	1.068481	1.758946	0.936412	0.619961
20.26158	-0.29541	2.39481	0.931678	0.422543	0.627942	1.367352	0.49933	0.727732
9.737593	-0.65958	1.497999	0.584305	0.434492	0.471972	1.157593	0.344365	0.779945
85.57026	-8.89428	4.214982	1.740052	0.386869	0.862933	1.661641	0.913483	0.671132
22.74502	-3.0971	1.922867	0.945226	0.328921	0.59409	1.375495	0.560748	0.747665
100.0696	-9.89445	4.328094	1.951581	0.358941	0.912058	1.726808	1.053866	0.661852
7.6115	-1.01922	1.137972	0.654424	0.256631	0.522875	1.237485	0.374661	0.758196
106.291	-0.86337	5.722816	2.177853	0.427558	1.083681	1.750604	0.845539	0.600953
10.44834	-1.22771	1.314259	0.603568	0.354618	0.442887	1.174135	0.391428	0.800406
123.418	-7.6246	5.417688	1.626894	0.521977	0.843756	1.624602	0.810687	0.675437
62.97292	-6.43611	3.253779	1.819491	0.262366	0.931066	1.740533	0.920475	0.641809
29.69551	-3.72262	2.946005	0.680806	0.62276	0.506383	1.235182	0.400766	0.772082
18.97734	-1.99627	2.016796	1.068093	0.295515	0.691014	1.431851	0.531618	0.708732
19.53746	-1.73052	1.942977	0.697173	0.459996	0.519948	1.244234	0.409791	0.764159
32.76935	-2.09808	2.942187	1.591399	0.288484	0.880264	1.646767	0.752183	0.65257
53.55029	-0.18949	4.227718	1.283943	0.526883	0.803413	1.542112	0.59299	0.667093
13.89595	-0.55948	2.314694	1.344699	0.245391	0.845385	1.48386	0.542515	0.649391
27.4985	-2.52114	1.909059	1.113792	0.255855	0.740664	1.491541	0.553652	0.682118
27.46432	-2.63996	2.347659	1.262766	0.287958	0.782671	1.550909	0.627666	0.673695
6.13193	-0.41841	1.438136	0.809626	0.273835	0.63679	1.315827	0.393234	0.709545
39.50512	1.397706	4.361887	0.795775	0.691286	0.584633	1.298079	0.417242	0.740071
40.38146	-3.34197	3.195714	1.354974	0.387971	0.795649	1.58329	0.688136	0.67556

29.19811	-0.59477	2.977065	1.111813	0.44867	0.709979	1.467675	0.569993	0.699898
53.77077	-6.44639	3.469793	1.084571	0.512046	0.690245	1.440466	0.55753	0.709036
24.40328	-2.77875	2.312664	1.494109	0.205222	0.866805	1.649025	0.715674	0.651804
21.51797	-2.80801	1.907118	0.819314	0.380268	0.578099	1.321204	0.457548	0.744931
32.35535	-3.69789	2.292176	1.080271	0.338762	0.722137	1.476011	0.544666	0.689246
29.71766	-3.73456	2.5037	1.133518	0.361569	0.733811	1.495507	0.574092	0.688317
54.12405	-4.69233	3.521316	1.01773	0.536437	0.670059	1.408199	0.516277	0.71432

142.5194	-9.1544	5.241488	1.9997	0.410419	0.944719	1.758552	1.033129	0.650341
4637.147	106.3742	5.635206	1.801902	0.515968	0.715956	1.496259	1.274359	0.712602
73.9056	-9.04975	3.172814	1.239468	0.417642	0.703825	1.518966	0.71191	0.714063
84.82733	-9.41573	3.886954	1.396251	0.450661	0.812249	1.594421	0.699262	0.670584

13.75476	-2.13735	1.220559	0.473553	0.427285	0.372482	1.067085	0.327593	0.827097
34.60885	-1.95211	3.481042	0.96105	0.563536	0.634638	1.38106	0.512478	0.72793
53.85042	3.604666	4.688442	1.278845	0.567807	0.746163	1.537999	0.682674	0.694423
20.42145	-1.68151	2.416012	1.532105	0.228917	0.923016	1.536049	0.566229	0.63009
18.14616	-2.31647	2.110942	0.963018	0.369714	0.653986	1.401553	0.506139	0.716351
26.62601	-1.30568	2.368258	0.916297	0.426341	0.619769	1.37044	0.509575	0.729336
23.98051	-1.32937	2.75783	1.259934	0.369777	0.792372	1.557443	0.610115	0.669417
45.00411	-5.71355	1.868461	0.826725	0.164062	0.601353	1.300587	0.431568	0.731355
11.29063	-0.30584	1.802317	0.846694	0.353598	0.607076	1.328901	0.448233	0.730795
153.6804	-8.71841	8.109296	0.982399	0.784545	0.640377	1.397861	0.520798	0.728497
45.31543	-6.81965	2.725615	0.9586	0.477183	0.637174	1.401823	0.537187	0.725137
8.823788	-0.22113	1.664787	0.564454	0.492069	0.484979	1.158109	0.319711	0.77032
39.84341	-1.26851	4.23829	0.93803	0.6397	0.65792	1.383006	0.469454	0.713149
250.6401	-8.85507	10.30089	3.878658	0.442278	1.488267	2.036229	1.353526	0.528623
57.27484	-2.56385	3.999538	1.142076	0.540399	0.7319	1.479725	0.569031	0.690559
31.0139	2.093184	3.173363	0.761945	0.606884	0.580102	1.260927	0.387594	0.737993
22.83637	-0.18161	3.355012	0.840673	0.600778	0.63341	1.329809	0.414858	0.71595
45.09697	-5.11632	2.42109	1.143894	0.307853	0.724901	1.499313	0.599935	0.693426
105.7224	-9.84284	5.943934	1.841537	0.520062	0.949327	1.696097	0.826352	0.640466
52.84296	-5.61307	3.201378	1.614548	0.318547	0.850159	1.660951	0.863993	0.664333
104.084	-10.9469	4.785299	1.521108	0.498178	0.794007	1.609422	0.811154	0.693514
88.12217	-5.0621	5.143152	2.383096	0.363759	1.098019	1.875158	1.080877	0.604437
357.3009	-7.30298	12.47733	2.964451	0.608585	1.109504	1.965926	1.597652	0.63234
62.26987	-5.47573	2.693833	1.999838	-0.02373	1.109592	1.572552	0.590843	0.576633
2.420681	0.183145	0.869373	0.46246	0.299547	0.427356	1.050571	0.269682	0.792105
7.074216	-0.15018	1.46777	0.8696	0.24296	0.649617	1.325017	0.411627	0.710784
4.440118	-0.02071	0.954934	0.4486	0.354522	0.406574	1.050231	0.274206	0.803398
22.71944	-2.57614	2.392113	0.692308	0.549452	0.53005	1.247396	0.388549	0.759554
2.907989	-0.43915	0.901791	0.441711	0.337457	0.388072	1.063924	0.286231	0.814453
29.36664	2.849563	3.635432	0.587729	0.72025	0.479751	1.172944	0.339971	0.77705
1.383827	-0.05497	0.700572	0.500589	0.164056	0.469824	1.077019	0.275305	0.770134
20.56068	-2.53642	2.002189	0.809476	0.409217	0.579483	1.335107	0.460501	0.742501
5875.137	-69.2787	1.535763	0.356402	0.390563	0.325164	0.962969	0.245842	0.842388
83.34012	-9.29275	2.596465	0.99958	0.403493	0.581298	1.38138	0.639835	0.757803
13.03108	-0.74504	1.96633	0.74383	0.45002	0.594181	1.291361	0.383335	0.726787
21.67329	1.390331	2.595663	0.738195	0.54331	0.544071	1.23514	0.39293	0.757566
27.16256	-3.12762	2.137323	1.023883	0.333247	0.664611	1.430135	0.549932	0.716386
1.646582	0.273948	0.70352	0.403299	0.264556	0.374705	1.015468	0.258456	0.817368
5.564333	-0.55166	1.10086	0.740404	0.175602	0.551976	1.287723	0.421541	0.751271
30.75516	-1.86776	1.849702	0.678451	0.444515	0.497492	1.234797	0.418354	0.775089
5.842527	0.603679	1.351311	0.794167	0.254005	0.627148	1.269718	0.371465	0.713248
45.80819	-2.57978	2.147598	0.948478	0.370497	0.643418	1.399846	0.52333	0.718195
2.556881	0.177241	0.754989	0.552263	0.144077	0.491984	1.066715	0.280014	0.763378
82.34264	-5.39916	3.453035	1.964194	0.224236	0.894673	1.710957	1.084135	0.668388
451.7644	-35.8151	10.32182	2.824925	0.553873	1.094835	1.891226	1.393012	0.641981

583.6809	-26.752	3.527793	0.630205	0.686472	0.362836	1.09582	0.494775	0.842692
215.7779	-0.56199	6.452058	1.249871	0.66183	0.692385	1.501175	0.723839	0.719391
122.3891	-1.51583	1.601919	0.405838	0.586999	0.355066	1.008334	0.275462	0.828723
91586.12	1237.227	20.56357	5.226373	0.604836	0.655732	1.281101	4.766306	0.766637
3.249039	0.115813	0.785765	0.421071	0.297138	0.374492	1.043348	0.276316	0.82024
2.772047	-0.13209	0.820089	0.501915	0.231358	0.435279	1.115935	0.305396	0.793115
4.674202	-0.28754	0.973427	0.510762	0.30256	0.425069	1.114874	0.318813	0.800271
4.172858	0.493149	0.929771	0.48069	0.309947	0.434642	1.08066	0.284987	0.789886
7.163809	0.5647	1.448015	0.900713	0.219965	0.646559	1.299028	0.41184	0.717483
3.166118	-0.03442	0.950873	0.572175	0.244656	0.49267	1.169412	0.323169	0.766427
26.94459	-2.5841	2.25436	0.751996	0.487822	0.532747	1.255647	0.431782	0.763785
4.63965	-0.17837	1.022622	0.500873	0.325787	0.429928	1.097689	0.300666	0.796491
41.22589	4.777619	3.599348	1.575396	0.366802	0.893785	1.468712	0.565817	0.65437
6.401432	-0.91822	1.115684	0.599957	0.290244	0.483918	1.202535	0.355989	0.77622
3.20253	-0.11207	0.839762	0.448864	0.297474	0.399604	1.049227	0.27741	0.808056
4.809425	0.499213	1.017434	0.604737	0.24076	0.488547	1.185208	0.349395	0.773601
3.69771	0.572589	0.655628	0.327836	0.291063	0.286454	0.920165	0.240899	0.863268
69.11779	-9.25315	4.463981	0.733952	0.717818	0.532061	1.269013	0.429478	0.761532
18.66499	-1.75777	1.562525	0.745056	0.326726	0.527222	1.279668	0.447719	0.766179
17.13306	-2.19056	1.470766	0.726825	0.312447	0.530564	1.266541	0.430642	0.761058
6.462143	-0.17551	0.918985	0.573824	0.209931	0.444198	1.178249	0.371645	0.795989
265.2372	-2.51291	8.967333	1.563219	0.699668	0.823582	1.637486	0.827063	0.677854
2.994556	-0.32287	0.606167	0.3491	0.251906	0.3082	0.96089	0.251518	0.852493
68.08441	-6.52399	4.266042	2.511684	0.211507	1.282055	1.580209	0.597442	0.535859
18.35006	-2.50451	1.509246	0.947719	0.212606	0.661632	1.38934	0.49674	0.708153
21.91332	-2.73213	1.652152	0.597589	0.449704	0.488081	1.181471	0.34946	0.771791
38.24625	-5.30936	2.554953	0.664768	0.586311	0.524684	1.231128	0.375471	0.758243
1144.046	-98.6811	16.46916	2.129963	0.756517	0.919375	1.775642	1.187854	0.673516
566.3406	31.46337	4.847193	2.312595	0.323743	0.751782	1.604405	1.701059	0.735191
2033.955	-73.9883	37.38038	3.135657	0.843455	1.156766	1.933866	1.600158	0.621901
6.447575	-0.82586	0.846757	0.455123	0.290075	0.409028	1.050306	0.279787	0.802126
310.9199	-26.7565	4.418489	1.534856	0.447762	0.704364	1.501013	0.985504	0.728287

VX43	VX44	VX45	VX46	VX47	VX48	VX49	VX50	VX51
0.697404	0.990957	0.942045	-0.13612	0.517918	0.461579	5.488772	0.124647	3.549717
0.749594	0.985486	0.934926	-0.24007	0.686319	0.386856	2.992118	0.13727	3.383215
0.815802	0.997218	0.971365	-0.10416	0.399867	0.349944	9.735424	0.30805	2.245671
0.801807	0.974976	0.920508	-0.0879	0.387604	0.37462	2.955586	0.299815	2.328752
0.693693	0.981795	0.920181	-0.12841	0.511986	0.458941	4.931845	0.122679	3.501444
0.732445	0.990796	0.944283	-0.13175	0.501835	0.429325	4.672488	0.160033	3.205565
0.669422	0.991533	0.941549	-0.08164	0.445663	0.49223	7.513707	0.113964	3.619684
0.680269	0.989255	0.937603	-0.14395	0.585256	0.467351	6.338692	0.10164	3.844826
0.744582	0.976867	0.913971	-0.10365	0.480473	0.450582	3.19004	0.170654	2.92133
0.6682	0.993274	0.948649	-0.10695	0.492059	0.473036	9.512849	0.105635	3.821832
0.726936	0.993932	0.952733	-0.19036	0.688392	0.451663	7.537584	0.108531	3.592435
0.781622	0.994877	0.959535	-0.22331	0.701652	0.393145	5.386687	0.162876	3.140364
0.722189	0.992395	0.947473	-0.18598	0.670215	0.448062	6.589863	0.112879	3.618034
0.656638	0.985743	0.926442	-0.17516	0.682267	0.474191	6.660082	0.079063	4.12842
0.733708	0.988242	0.938324	-0.10043	0.402594	0.444767	6.238085	0.189907	2.898391
0.75832	0.994336	0.95784	-0.16421	0.585571	0.40407	8.090033	0.163757	3.174226
0.724973	0.979231	0.919005	-0.18057	0.638262	0.428939	4.287506	0.132482	3.464345
0.699275	0.990642	0.942018	-0.20905	0.712084	0.449516	5.32099	0.101616	3.814807
0.575912	0.989647	0.936271	-0.17327	0.677522	0.446309	9.237536	0.042142	5.17868
0.723121	0.991689	0.946908	-0.28523	0.833087	0.425588	6.475745	0.099538	3.908826
0.729062	0.991017	0.943874	-0.27831	0.817871	0.445202	5.743955	0.088856	3.817422
0.649161	0.987235	0.930523	-0.20958	0.75959	0.467384	6.363378	0.059215	4.448311
0.765707	0.993582	0.955719	-0.15619	0.556237	0.391532	5.648196	0.186165	3.055843
0.733068	0.999674	0.988435	-0.25858	0.767008	0.427279	43.98778	0.097446	3.752384
0.754857	0.994278	0.957263	-0.18589	0.637231	0.40681	7.221358	0.149559	3.290343
0.766638	0.990024	0.946237	-0.11314	0.459019	0.383606	3.710175	0.236533	2.82792
0.846941	0.999845	0.993511	-0.11338	0.411139	0.291902	41.13842	0.395487	2.002964
0.675184	0.988848	0.936344	-0.2207	0.728679	0.442515	6.52798	0.071633	4.209164
0.730808	0.990372	0.943636	-0.14461	0.565459	0.440692	6.358509	0.146925	3.354881
0.666461	0.999443	0.984389	-0.19547	0.705073	0.463212	44.08431	0.074836	4.181388
0.696219	0.989248	0.940149	-0.13771	0.533692	0.436015	6.671471	0.158904	3.495698
0.729249	0.991042	0.943931	-0.20968	0.711592	0.441798	4.947909	0.113046	3.656285
0.726118	0.989302	0.937626	-0.08186	0.414939	0.465259	5.224819	0.172072	3.043567
0.707169	0.990892	0.943568	-0.23657	0.734307	0.425785	6.787383	0.094103	3.874759
0.776628	0.996923	0.967466	-0.26301	0.778345	0.406461	4.528826	0.133258	3.297726
0.76627	0.991548	0.947218	-0.14029	0.517448	0.422412	6.149281	0.202895	2.771124
0.675583	0.999236	0.981143	-0.2644	0.858358	0.495595	15.1484	0.05786	4.472122
0.713951	0.984309	0.926195	-0.11376	0.445643	0.45746	3.698112	0.148302	3.232963
0.793089	0.98687	0.9401	-0.05198	0.263244	0.37244	3.915683	0.31799	2.345975
0.635178	0.976717	0.915168	-0.13966	0.51393	0.413741	6.543989	0.109815	3.962414
0.690354	0.991562	0.947264	-0.16437	0.600085	0.43083	7.523541	0.103644	3.952185
0.699804	0.994085	0.953799	-0.29906	0.862595	0.44499	8.105423	0.077201	4.316537
0.610229	0.982768	0.919852	-0.18452	0.676822	0.451722	6.163831	0.050982	4.699756
0.740518	0.994072	0.954904	-0.22206	0.705509	0.422161	6.590593	0.122201	3.591459
0.775314	0.992009	0.949358	-0.18837	0.658155	0.409342	4.975951	0.162469	3.047016
0.722679	0.988272	0.9362	-0.15137	0.564971	0.452327	4.962283	0.138	3.383278
0.730725	0.991716	0.948194	-0.15682	0.533396	0.423573	7.673796	0.151795	3.333074
0.659349	0.999439	0.984388	-0.22522	0.734362	0.430018	44.52225	0.070634	4.290615
0.723739	0.988499	0.936595	-0.24461	0.775575	0.446989	4.806246	0.103251	3.791146
0.725405	0.994344	0.95543	-0.25804	0.789507	0.43364	7.978754	0.099832	3.920531
0.738996	0.995586	0.960827	-0.12404	0.478513	0.422822	9.863473	0.174976	3.176026
0.733058	0.977237	0.919933	-0.1068	0.387084	0.403319	4.426485	0.19578	2.83469
0.683846	0.984634	0.925864	-0.14668	0.599194	0.461221	5.449757	0.104092	3.785589
0.741082	0.994813	0.958145	-0.16444	0.590788	0.419885	9.892383	0.150005	3.319125
0.704097	0.998305	0.975544	-0.22151	0.745359	0.421704	7.504903	0.082803	4.085621
0.726003	0.992801	0.95373	-0.14912	0.527581	0.400722	9.489926	0.176917	3.387125
0.676933	0.990265	0.941211	-0.18725	0.666919	0.43171	7.532243	0.104114	3.967513

0.674788	0.98566	0.929583	-0.1085	0.456533	0.456913	5.969218	0.123085	3.673464
0.723069	0.988404	0.936389	-0.1774	0.651813	0.447848	4.278451	0.119622	3.560559
0.702532	0.995388	0.959497	-0.15141	0.561234	0.421302	8.684141	0.141085	3.644991
0.75487	0.996271	0.963798	-0.26389	0.776955	0.41724	7.251435	0.117724	3.569315
0.663471	0.982056	0.91994	-0.17177	0.617786	0.446222	4.794774	0.085074	4.041743
0.760844	0.989614	0.944734	-0.11525	0.436503	0.397222	5.918102	0.224493	2.865689
0.753977	0.990707	0.944178	-0.17289	0.63952	0.430906	5.838316	0.152316	3.206077
0.715089	0.992012	0.945939	-0.24082	0.785504	0.45202	5.194646	0.091682	3.879839
0.750808	0.9901	0.943094	-0.07714	0.340683	0.428582	5.422456	0.198753	2.751596
0.627934	0.984844	0.92477	-0.10632	0.437916	0.460753	7.888661	0.090956	3.966057
0.719971	0.98823	0.935671	-0.16956	0.627522	0.446311	3.845907	0.130187	3.494005
0.698459	0.991323	0.942573	-0.19595	0.724335	0.468686	6.39944	0.086877	3.901895
0.732445	0.995952	0.960519	-0.15971	0.630454	0.455112	7.651362	0.130536	3.40745
0.665853	0.993432	0.950505	-0.32024	0.881269	0.437745	6.501599	0.053288	4.72797
0.759047	0.993707	0.954729	-0.26971	0.800097	0.406309	6.035345	0.111808	3.620028
0.769204	0.999775	0.990655	-0.20978	0.68897	0.412879	44.13917	0.153782	3.177391
0.71416	0.990538	0.944352	-0.13	0.443665	0.418598	6.680411	0.189572	3.192859
0.693916	0.992468	0.946207	-0.16344	0.622402	0.465265	7.119805	0.099443	3.765182
0.783551	0.994938	0.95991	-0.24103	0.709883	0.390099	6.468192	0.165851	3.133806
0.674494	0.989808	0.937201	-0.17838	0.680292	0.472675	6.986979	0.080052	4.048282
0.727636	0.99786	0.971347	-0.1704	0.63844	0.447899	6.547303	0.120897	3.504791
0.733076	0.985691	0.931343	-0.16179	0.571353	0.431097	4.926914	0.149052	3.281475
0.5861	0.993453	0.945154	-0.18645	0.794138	0.50204	9.07613	0.039393	5.056026
0.690379	0.986002	0.932644	-0.23644	0.754068	0.409001	5.230429	0.106996	4.056635
0.666487	0.993835	0.949223	-0.0964	0.546762	0.498674	7.669245	0.101722	3.838155
0.735953	0.995865	0.960833	-0.18479	0.669285	0.442067	6.902053	0.132255	3.467367
0.76202	0.991102	0.946069	-0.14335	0.557317	0.41872	5.024337	0.183537	3.035516
0.683742	0.988613	0.933737	-0.10566	0.536249	0.486828	6.322174	0.111731	3.653998
0.698428	0.993225	0.94772	-0.0931	0.512538	0.489083	6.521132	0.125576	3.490062
0.678448	0.982548	0.923261	-0.19249	0.706924	0.453756	5.447988	0.077609	4.139919
0.703539	0.991785	0.94385	-0.11333	0.501029	0.473583	6.373329	0.132002	3.423142
0.69014	0.989544	0.939461	-0.16665	0.626625	0.443892	6.301271	0.109983	3.846481
0.714167	0.991473	0.945253	-0.19042	0.684802	0.436887	6.041831	0.117097	3.609142
0.723409	0.99772	0.973414	-0.21715	0.669074	0.416701	5.253024	0.121744	3.702492
0.681064	0.98718	0.932029	-0.21404	0.718094	0.445338	5.662498	0.091155	4.041623
0.622734	0.987549	0.932899	-0.22529	0.709527	0.418098	6.798674	0.055071	4.734499
0.69121	0.994156	0.952877	-0.11773	0.557907	0.476074	6.97092	0.123096	3.630002
0.795814	0.992974	0.954132	-0.15923	0.561827	0.381584	3.365318	0.216959	2.68208
0.700567	0.978237	0.912236	-0.09474	0.389968	0.476847	3.733986	0.148381	3.169161
0.637538	0.976559	0.910684	-0.15686	0.516192	0.430635	4.805801	0.092163	3.939584
0.679276	0.986931	0.931503	-0.22502	0.750702	0.452886	5.746671	0.080956	4.148691
0.700252	0.986144	0.930238	-0.14301	0.47802	0.445758	5.240189	0.128734	3.485677
0.713241	0.9889	0.939244	-0.2035	0.614099	0.416749	5.201356	0.139892	3.576678
0.709321	0.990741	0.943732	-0.19627	0.67323	0.431555	6.995348	0.122822	3.728741
0.708878	0.991307	0.944259	-0.1487	0.603555	0.458105	6.783904	0.118289	3.62048
0.700073	0.988175	0.936245	-0.16011	0.561054	0.4411	6.100354	0.119728	3.649864
0.695213	0.996375	0.961868	-0.09884	0.474314	0.471929	8.115688	0.138034	3.486359
0.763846	0.993154	0.95751	-0.1047	0.374516	0.378456	7.779864	0.25741	2.805466
0.760272	0.992327	0.95054	-0.1041	0.396077	0.411913	5.782386	0.224265	2.797133
0.701801	0.992097	0.946956	-0.1209	0.503261	0.444881	7.907941	0.134798	3.574565
0.74832	0.991906	0.948071	-0.22505	0.719657	0.421932	5.429773	0.138832	3.453062
0.735437	0.991324	0.94528	-0.22162	0.709073	0.432971	4.997351	0.114081	3.604904
0.712875	0.997248	0.967681	-0.20482	0.714889	0.442406	6.248428	0.099249	3.83009
0.785661	0.991358	0.950963	-0.10926	0.423192	0.365839	5.813079	0.281532	2.621987
0.748865	0.999661	0.988777	-0.1844	0.563439	0.404395	44.46843	0.160585	3.21085
0.754402	0.995813	0.961218	-0.2394	0.757424	0.432355	9.065392	0.124132	3.380376
0.718331	0.989972	0.941057	-0.10614	0.417197	0.443484	5.617323	0.157373	3.249178
0.759291	0.994224	0.955274	-0.09592	0.400105	0.426879	6.596303	0.210033	2.758582

0.653058	0.995156	0.953602	-0.1344	0.655951	0.507962	6.741041	0.075865	4.139904
0.639332	0.985375	0.927127	-0.17549	0.586955	0.431395	4.919197	0.077189	4.225139
0.7263	0.998784	0.978971	-0.13524	0.577447	0.439932	7.562355	0.149242	3.425854
0.777558	0.986341	0.935809	-0.09607	0.356052	0.407922	3.431288	0.228603	2.528993
0.66863	0.991756	0.941649	-0.08615	0.449542	0.496208	7.046018	0.111186	3.661936
0.734019	0.99236	0.949349	-0.27623	0.783693	0.417207	5.752318	0.104631	3.82929
0.639229	0.975079	0.904	-0.14531	0.505423	0.460926	4.110854	0.087676	3.940731
0.779388	0.992272	0.950341	-0.102	0.4576	0.410141	5.50882	0.213537	2.638991
0.756537	0.988575	0.938261	-0.06804	0.305392	0.436066	4.311563	0.219107	2.63483
0.745583	0.993308	0.952147	-0.12582	0.464851	0.428055	7.647058	0.177729	3.032826
0.686178	0.986564	0.932418	-0.19772	0.641785	0.431204	5.565998	0.098508	3.995962
0.661496	0.982508	0.924585	-0.11247	0.454007	0.445997	6.436455	0.108148	3.830624
0.725109	0.99732	0.969987	-0.26269	0.776266	0.410238	7.778172	0.116453	3.881761
0.747358	0.992315	0.951476	-0.19835	0.615968	0.397123	6.754695	0.151487	3.373938
0.740468	0.994612	0.957792	-0.16605	0.637002	0.413735	6.682617	0.175362	3.355961
0.682519	0.98743	0.936134	-0.23338	0.762502	0.420576	6.72046	0.092663	4.207244
0.701308	0.991705	0.94341	-0.14336	0.622367	0.480719	6.250335	0.10993	3.651898
0.766827	0.995048	0.95989	-0.28689	0.809403	0.399275	5.195683	0.113405	3.597898
0.706664	0.984686	0.930387	-0.14278	0.484169	0.410235	5.23547	0.156495	3.337751
0.728095	0.992257	0.948287	-0.1569	0.499939	0.429187	6.18636	0.157732	3.291396
0.634197	0.978792	0.911743	-0.17296	0.61009	0.453292	3.948699	0.069635	4.260433
0.710517	0.994176	0.955324	-0.13948	0.536767	0.436071	9.743859	0.142251	3.554834
0.718734	0.992908	0.952725	-0.15239	0.491408	0.405898	8.93734	0.146888	3.449571
0.738312	0.991235	0.945717	-0.05582	0.318232	0.457507	7.500221	0.209938	2.694527
0.697767	0.995524	0.959145	-0.21305	0.68701	0.432472	9.381188	0.087414	3.962873
0.698246	0.986885	0.934815	-0.28301	0.811611	0.401157	5.022088	0.101889	4.076265
0.633309	0.979134	0.911815	-0.18859	0.697534	0.460867	5.249745	0.059516	4.40138
0.772184	0.993466	0.953639	-0.07367	0.37256	0.416213	6.728199	0.231971	2.607798
0.701913	0.988396	0.936766	-0.12236	0.467142	0.441366	5.094789	0.147943	3.339792
0.61445	0.991631	0.941861	-0.09894	0.457004	0.483214	8.261832	0.080043	4.238537
0.687265	0.981911	0.918779	-0.11233	0.55418	0.482344	4.98915	0.115675	3.556771
0.682543	0.988936	0.940674	-0.1366	0.470271	0.422529	7.111581	0.122798	3.694714
0.711151	0.99224	0.948563	-0.21448	0.718435	0.445849	6.960376	0.095287	3.862182
0.706477	0.991139	0.943697	-0.10202	0.472039	0.460928	7.273132	0.133252	3.423495
0.664852	0.984512	0.922	-0.0442	0.270859	0.503266	5.609428	0.135012	3.373602
0.758813	0.976922	0.918407	-0.08781	0.340375	0.407037	3.309625	0.224381	2.612929
0.581633	0.984927	0.924054	-0.2637	0.807887	0.422259	7.229678	0.041168	5.079019
0.715219	0.991094	0.945074	-0.15426	0.555156	0.438139	5.60292	0.126311	3.497501
0.774664	0.996034	0.96428	-0.14464	0.522884	0.396011	9.71414	0.194781	2.880022
0.647849	0.986662	0.933131	-0.13437	0.503754	0.4487	7.380841	0.09537	4.06422
0.73571	0.986468	0.937503	-0.11873	0.438561	0.400872	6.584808	0.190495	3.141334
0.637748	0.982275	0.92404	-0.12486	0.551275	0.454476	6.607613	0.099323	4.021331
0.751615	0.98732	0.936389	-0.06287	0.316455	0.428683	5.219232	0.222733	2.693799
0.562809	0.975298	0.900779	-0.17974	0.680967	0.4666	5.551536	0.061662	4.528968
0.794111	0.992889	0.957074	-0.13839	0.474015	0.345265	7.026026	0.261552	2.663811
0.650795	0.990819	0.943177	-0.18504	0.630633	0.432857	8.92084	0.076641	4.361317
0.615383	0.985986	0.92756	-0.06603	0.37982	0.483265	7.597264	0.097575	4.016814
0.763852	0.993419	0.955228	-0.23303	0.714948	0.386839	6.502348	0.160669	3.319944
0.691635	0.984352	0.92719	-0.11736	0.379117	0.438392	6.035154	0.15358	3.366569
0.756958	0.996957	0.968123	-0.14935	0.559077	0.410408	12.28471	0.169532	3.140834
0.627172	0.981846	0.9179	-0.09253	0.388511	0.475345	5.923451	0.089898	4.017268
0.645473	0.987727	0.930433	-0.1471	0.615227	0.483446	5.496046	0.075655	4.212488
0.626241	0.966638	0.887582	-0.13812	0.536781	0.506663	3.90557	0.105677	3.607787
0.665598	0.989351	0.935337	-0.04733	0.310018	0.496508	7.070763	0.132029	3.469319
0.654575	0.991556	0.94228	-0.06772	0.36525	0.48948	8.132946	0.111744	3.695512
0.698865	0.978887	0.912074	-0.05932	0.349428	0.493572	4.445097	0.149034	3.144663
0.728632	0.992282	0.94841	-0.25351	0.768093	0.425199	6.750788	0.097662	3.767534
0.655188	0.98724	0.931587	-0.10894	0.514824	0.473212	7.191287	0.096651	3.930022

0.683869	0.98941	0.938313	-0.14078	0.575226	0.463236	6.41361	0.102109	3.81331
0.693162	0.989663	0.940061	-0.16106	0.57549	0.446364	6.967662	0.120357	3.72039
0.627339	0.978521	0.910147	-0.05514	0.306083	0.486686	5.700587	0.111941	3.773794
0.734473	0.987931	0.938283	-0.12758	0.47398	0.421604	5.942263	0.195552	3.069805
0.673585	0.991393	0.942001	-0.07421	0.405252	0.4892	8.295062	0.125135	3.558417
0.671797	0.983497	0.922603	-0.08929	0.447566	0.482819	6.102813	0.122065	3.579636
0.698849	0.991889	0.946286	-0.17728	0.608513	0.440823	8.265943	0.111136	3.791937
0.623541	0.987001	0.932565	-0.13888	0.579353	0.4565	7.666223	0.085793	4.29942
0.700084	0.999465	0.987932	-0.10364	0.527705	0.460027	8.545064	0.135648	3.595369
0.698066	0.990333	0.944484	-0.11656	0.480321	0.430144	7.955206	0.160192	3.480271
0.649464	0.98685	0.930256	-0.11483	0.519686	0.476402	7.037302	0.101829	3.936291
0.823392	0.997628	0.975565	-0.14188	0.493608	0.313855	10.75972	0.337739	2.375805
0.714191	0.988775	0.939318	-0.20161	0.674481	0.427563	6.228493	0.105083	3.754988
0.676532	0.991527	0.945306	-0.19163	0.709576	0.45556	7.377436	0.08257	4.06243
0.599214	0.971197	0.892747	-0.12437	0.521274	0.482409	4.798245	0.09906	3.76094
0.702928	0.988712	0.937245	-0.10558	0.448747	0.453795	6.399377	0.142504	3.389883
0.718323	0.991267	0.945741	-0.13436	0.547395	0.446551	6.394427	0.136231	3.461315
0.649197	0.98532	0.924766	-0.0821	0.462557	0.490049	5.814394	0.091723	3.899629
0.721453	0.987764	0.935545	-0.13174	0.423247	0.453521	6.431076	0.201829	2.806387
0.719762	0.987494	0.93506	-0.12231	0.485155	0.445707	5.191081	0.149705	3.256921
0.713366	0.995719	0.96119	-0.30961	0.856086	0.41513	11.09525	0.071928	4.32256
0.712941	0.982045	0.924473	-0.15424	0.623887	0.444602	5.226253	0.161843	3.193994
0.76545	0.991437	0.946986	-0.14473	0.572826	0.419499	4.845727	0.179384	3.011324
0.698768	0.986094	0.929893	-0.21106	0.726281	0.454057	5.155565	0.087183	3.913309
0.473783	0.965165	0.880756	-0.19207	0.753992	0.435869	5.985154	0.035656	5.122071
0.673353	0.990942	0.941487	-0.16308	0.636567	0.472981	6.738656	0.088973	4.03063
0.727943	0.99385	0.952797	-0.2155	0.690106	0.441033	7.048983	0.116502	3.582827
0.703987	0.983743	0.923639	-0.18932	0.690627	0.469653	4.436371	0.096337	3.674174
0.67741	0.990957	0.9421	-0.07986	0.364797	0.475869	8.022626	0.136249	3.508208
0.609799	0.979129	0.912484	-0.17441	0.65387	0.452506	5.962095	0.070157	4.414599
0.642885	0.987619	0.933526	-0.08223	0.461783	0.48129	7.858703	0.097864	3.95402
0.671437	0.985864	0.932837	-0.16744	0.586874	0.417591	6.372238	0.120821	4.000685
0.567871	0.981809	0.915917	-0.09396	0.457221	0.466451	7.589178	0.055283	4.70468
0.598244	0.984063	0.928392	-0.20871	0.778254	0.414558	7.808208	0.04373	5.197406
0.533465	0.976717	0.896814	-0.22961	0.731084	0.485294	6.796581	0.112985	3.548107
0.789831	0.982502	0.929605	-0.09098	0.354013	0.397268	2.667414	0.244097	2.363758
0.69719	0.97747	0.911075	-0.09423	0.373556	0.46725	3.970656	0.158485	3.153047
0.800906	0.993165	0.955284	-0.11271	0.42885	0.372755	4.673456	0.271138	2.381666
0.751059	0.993273	0.953006	-0.17856	0.623745	0.410354	6.738453	0.171494	3.280002
0.81131	0.991309	0.95222	-0.09783	0.41607	0.345264	4.68297	0.283173	2.349786
0.770901	0.992946	0.952987	-0.28456	0.797551	0.39524	5.067457	0.125372	3.430546
0.768163	0.986602	0.933428	-0.03451	0.205844	0.443664	3.572546	0.239778	2.296064
0.732631	0.990464	0.944021	-0.11908	0.516846	0.431366	6.692887	0.17598	3.180447
0.840542	0.999832	0.993095	-0.10304	0.363212	0.300059	43.12099	0.405095	2.0185
0.7473	0.993424	0.957358	-0.12324	0.474575	0.389086	9.102145	0.211823	3.012228
0.717844	0.993972	0.951432	-0.10477	0.524619	0.473073	7.564025	0.137853	3.335592
0.747216	0.994053	0.955873	-0.21533	0.624707	0.399082	5.899535	0.140488	3.369525
0.702455	0.988075	0.936582	-0.10651	0.417295	0.447738	6.387562	0.156384	3.283592
0.815507	0.984722	0.938227	-0.06966	0.312133	0.35011	3.244199	0.32206	2.152793
0.742667	0.98582	0.933477	-0.07729	0.351013	0.424936	4.681555	0.214929	2.774226
0.768226	0.998147	0.975535	-0.14405	0.547409	0.391077	15.01276	0.203175	3.027574
0.703128	0.970921	0.899379	-0.09719	0.436277	0.490591	2.965875	0.171318	3.022813
0.706748	0.994562	0.955398	-0.08995	0.461768	0.465557	8.273614	0.157042	3.346742
0.760017	0.985372	0.93076	-0.1184	0.409207	0.445497	3.690634	0.265647	2.303879
0.645079	0.985252	0.931423	-0.12545	0.497553	0.448609	7.25288	0.107175	3.972147
0.604978	0.982033	0.924195	-0.20362	0.66065	0.372468	7.245303	0.082521	4.725392

0.837504	0.999527	0.990363	-0.24644	0.704836	0.268159	28.65552	0.33858	2.568791
0.703488	0.994668	0.9586	-0.24288	0.78049	0.420682	9.218974	0.088493	4.302514
0.826602	0.999689	0.990437	-0.22063	0.670138	0.324968	32.30682	0.266087	2.610011
0.761891	0.998909	0.990377	-0.10928	0.478039	0.413397	6.626596	0.222977	2.751944
0.817403	0.994911	0.962969	-0.08068	0.35425	0.336292	6.066745	0.351549	2.246209
0.789016	0.981274	0.929015	-0.06517	0.277337	0.380232	3.000053	0.301281	2.403431
0.795954	0.993875	0.958244	-0.09947	0.371574	0.363467	5.758909	0.278467	2.463142
0.787269	0.992681	0.952206	-0.08637	0.373125	0.398666	4.734121	0.256845	2.4013
0.702114	0.97681	0.912096	-0.14025	0.420733	0.439092	3.131622	0.17486	3.036654
0.761608	0.988759	0.939504	-0.05656	0.307854	0.427668	3.703115	0.228806	2.612345
0.754785	0.992782	0.953129	-0.18608	0.586813	0.395827	7.246233	0.165242	3.196481
0.792123	0.992401	0.953009	-0.11465	0.37513	0.37129	4.867735	0.279648	2.512056
0.621079	0.977045	0.907961	-0.22419	0.614901	0.41258	4.049272	0.116312	3.63804
0.769621	0.990961	0.947492	-0.07879	0.342424	0.393179	5.935839	0.263791	2.647903
0.805097	0.993173	0.956138	-0.09832	0.344499	0.359637	5.084264	0.311318	2.323248
0.767318	0.984243	0.93221	-0.08927	0.319567	0.400766	3.019521	0.260243	2.59159
0.860912	0.993555	0.964755	-0.11736	0.340066	0.254046	2.993115	0.472293	1.839937
0.753182	0.992955	0.953061	-0.26298	0.786093	0.406086	7.980002	0.12859	3.598571
0.757484	0.99405	0.957382	-0.1081	0.386795	0.393339	5.916439	0.23548	2.886933
0.753569	0.991454	0.948598	-0.09222	0.370062	0.413585	7.189872	0.215937	2.835879
0.790543	0.995385	0.963772	-0.06825	0.323559	0.361744	6.981096	0.316769	2.456437
0.655601	0.99237	0.947929	-0.23847	0.804676	0.448378	8.846022	0.064397	4.668132
0.849987	0.994691	0.966205	-0.07523	0.29062	0.274501	5.024243	0.457143	1.882496
0.480291	0.963908	0.870497	-0.23549	0.785676	0.45954	5.651408	0.078805	4.056137
0.696663	0.988894	0.93626	-0.06074	0.302458	0.485685	6.382504	0.171994	3.039995
0.766647	0.994184	0.956421	-0.12781	0.506798	0.413272	7.312567	0.191809	2.862908
0.751321	0.995478	0.960381	-0.17852	0.645066	0.425796	9.190465	0.162404	3.193299
0.646958	0.986351	0.935177	-0.24388	0.793193	0.407513	8.865763	0.115712	4.391441
0.718465	0.994402	0.965355	-0.14562	0.557638	0.369164	6.141865	0.224917	3.325626
0.588287	0.977018	0.914462	-0.32059	0.90475	0.431973	6.671223	0.054675	4.969442
0.799933	0.9931	0.955036	-0.08409	0.336375	0.377409	6.663631	0.282229	2.264986
0.714487	0.990359	0.949686	-0.14828	0.541613	0.411665	8.981904	0.181319	3.345041

VX52	VX53	VX54	VX55	VX56	VX57	VX58	VX59	VX60
0.497729	0.229757	10.97754	2.620761	0.845678	6.090031	1378.307	0.055013	25.31122
0.633111	0.252775	5.984235	2.66178	1.017824	6.225599	1867.606	0.048195	36.07538
0.38662	0.497183	19.47085	1.804873	0.312771	5.481789	1434.057	0.042989	41.79944
0.331668	0.505836	5.911171	1.842948	0.316127	4.887988	57.71619	0.063987	20.08683
0.455803	0.238009	9.863691	2.585521	0.837264	6.008024	275.2747	0.058075	23.66438
0.455877	0.247924	9.344975	2.418797	0.683357	6.054658	921.4578	0.047527	33.21114
0.42496	0.184258	15.02741	2.593997	0.823305	5.894518	428.5359	0.05976	21.0832
0.537267	0.189968	12.67738	2.826591	1.061736	6.310767	941.3792	0.05569	24.40782
0.404154	0.295395	6.38008	2.245472	0.504514	5.225465	52.66097	0.075016	13.61939
0.433196	0.216254	19.0257	2.760285	1.011817	6.156618	2180.218	0.05503	24.49434
0.633952	0.198199	15.07517	2.791289	0.946867	6.374171	9071.171	0.050395	29.10505
0.62013	0.280572	10.77337	2.552358	0.683541	6.232547	21009.36	0.046576	33.98183
0.591437	0.215131	13.17973	2.797127	0.944488	6.326922	1451.957	0.051754	27.83838
0.605692	0.160529	13.32016	3.077449	1.458085	6.290931	324.5701	0.062634	21.02758
0.351062	0.310809	12.47617	2.17045	0.564672	5.494725	108.1743	0.06003	22.58275
0.528242	0.309431	16.18007	2.468011	0.681957	6.202055	4615.525	0.044683	36.73051
0.583075	0.272459	8.575011	2.660855	0.916082	6.001305	153.4013	0.05617	25.1538
0.665983	0.203174	10.64198	2.917654	1.343149	6.41936	1859.411	0.052403	27.55984
0.594477	0.093373	18.47507	3.657856	3.029613	6.889196	2834.737	0.067218	19.32443
0.807064	0.219026	12.95149	3.114241	1.950555	6.585343	5242.73	0.046142	34.15604
0.764185	0.14503	11.48791	3.042338	1.441361	6.548993	8102.52	0.051726	27.7086
0.699866	0.10351	12.72676	3.323571	2.086158	6.601897	2479.477	0.058175	22.77636
0.486429	0.33177	11.29639	2.369268	0.615365	5.975933	197.0139	0.047065	35.50696
0.727051	0.151883	87.97556	2.975853	1.327601	6.572558	6678.55	0.05066	29.52983
0.579249	0.243891	14.44272	2.567902	0.775571	6.265361	1988.291	0.046509	34.40976
0.408633	0.43145	7.42035	2.167912	0.52236	5.667404	191.363	0.048643	34.36515
0.610416	0.601273	82.27683	1.63638	0.43679	5.363529	930.4839	0.043102	46.09991
0.655925	0.124002	13.05596	3.190453	1.666514	6.59847	1639.915	0.057557	24.59821
0.504132	0.246435	12.71702	2.557597	0.762734	6.081063	534.8609	0.052391	27.30973
0.671813	0.160803	88.16862	3.130304	3.448841	6.55393	1026.755	0.056521	23.8974
0.476201	0.328249	13.34294	2.617884	1.034372	5.895368	194.8533	0.053738	28.44604
0.628836	0.235661	9.895818	2.858752	1.004509	6.412971	3220.23	0.050359	29.13167
0.381569	0.303438	10.44964	2.272096	0.54686	5.68805	254.2066	0.05419	25.61743
0.670365	0.205073	13.57477	2.998024	1.374134	6.510692	2702.826	0.052067	29.65933
0.711581	0.222693	9.057651	2.710372	0.838207	6.353052	17506.38	0.047244	33.18835
0.510967	0.352977	12.29856	2.197652	0.524065	5.632367	272.237	0.06035	21.7139
0.797943	0.108862	30.29679	3.457862	2.247611	6.71698	33801.1	0.059263	21.41629
0.399601	0.220492	7.396225	2.389014	0.645838	5.890619	185.2227	0.055439	24.89611
0.250479	0.529415	7.831367	1.794244	0.318793	5.333416	332.1098	0.042173	44.61831
0.445226	0.257512	13.08798	2.87098	1.544397	6.102512	187.4217	0.057106	27.12315
0.516718	0.230429	15.04708	2.919506	1.267532	6.55793	1437.086	0.047608	33.83229
0.832543	0.19635	16.21085	3.393076	2.617788	6.819008	12284.6	0.050356	28.97361
0.609734	0.084393	12.32766	3.394622	2.251246	6.612742	808.9397	0.065517	18.81048
0.628181	0.242704	13.18119	2.814995	0.97798	6.44078	5181.769	0.049256	31.01056
0.58106	0.305308	9.951902	2.460536	0.613649	6.110802	2405.048	0.048076	32.05001
0.493226	0.26478	9.924566	2.572486	0.751264	6.05795	331.1573	0.054601	25.49111
0.497126	0.299055	15.34759	2.539631	0.811815	6.097598	477.9057	0.050053	30.33902
0.686468	0.141606	89.04451	3.255947	2.006481	6.614886	2842.982	0.056072	26.56888
0.711309	0.209793	9.612491	3.00014	1.271535	6.44567	1528.838	0.05305	27.21081
0.723626	0.196085	15.95751	3.084925	1.422027	6.649656	2352.262	0.049369	30.75431
0.538903	0.34981	19.72695	2.402037	0.673261	6.048995	2974.121	0.04449	36.58554
0.344775	0.309429	8.852969	2.097921	0.575258	5.739455	208.2584	0.04928	31.67671
0.529722	0.228395	10.89951	2.821094	1.059671	6.1798	468.3264	0.055522	24.78326
0.5452	0.2991	19.78477	2.560233	0.794056	6.189668	807.6148	0.048334	31.8719
0.66841	0.137214	15.00981	3.114851	1.585049	6.742733	3978.835	0.045994	35.09091
0.502205	0.355441	18.97985	2.566019	1.103019	6.137886	1427.024	0.043927	38.16478
0.616785	0.246394	15.06449	2.98574	1.508007	6.302531	965.9543	0.053805	26.73226

0.432002	0.266835	11.93844	2.645032	0.947962	6.044265	283.9483	0.055567	26.22815
0.581809	0.240984	8.556901	2.739176	0.89259	6.164024	253.298	0.055017	25.40931
0.504109	0.313119	17.36828	2.726708	1.054736	6.242291	727.157	0.047611	34.35181
0.713077	0.196664	14.50287	2.87066	1.023268	6.481977	24995.72	0.049095	30.8177
0.549292	0.165893	9.589548	2.981156	1.309326	6.277248	356.9111	0.061515	21.00382
0.431223	0.422555	11.8362	2.215071	0.56519	5.835872	1204.063	0.043646	38.74596
0.578722	0.304451	11.67663	2.525376	0.689094	6.14417	3778.626	0.047927	31.71758
0.734011	0.203435	10.38929	3.043221	1.415835	6.496969	2884.269	0.0506	29.03888
0.313424	0.264468	10.84491	2.066876	0.457832	5.666186	301.3757	0.05189	27.92948
0.356857	0.176317	15.77732	2.762257	1.162543	6.029793	173.9357	0.063226	21.09618
0.550871	0.276492	7.691814	2.683408	0.859769	6.137687	127.6277	0.052914	28.91974
0.651884	0.157855	12.79888	2.98674	1.229395	6.373244	3552.857	0.05286	26.8577
0.564274	0.249107	15.30272	2.644551	0.767671	6.150004	16438.73	0.051648	27.80243
0.834863	0.10784	13.0032	3.681402	3.762723	7.040487	6316.183	0.054232	26.90153
0.731605	0.188484	12.07069	2.913516	1.103151	6.552752	9751.653	0.043731	38.05035
0.685559	0.272419	88.27833	2.554354	0.775753	6.211696	6377.804	0.048335	31.78533
0.373633	0.365383	13.36082	2.370017	0.805134	5.762283	103.669	0.052385	30.27823
0.572166	0.179059	14.23961	2.823985	1.021179	6.315615	1017.064	0.05407	25.65694
0.633094	0.272083	12.93638	2.556327	0.699074	6.256473	15791.49	0.046522	34.40194
0.604082	0.137361	13.97396	3.041654	1.317823	6.414575	898.7279	0.057237	22.92603
0.561947	0.203567	13.09461	2.69973	0.859172	6.264265	3499.027	0.050994	28.70068
0.513503	0.297403	9.853828	2.525278	0.746216	6.100309	862.2552	0.049744	31.03769
0.723805	0.07696	18.15226	3.648329	2.934668	6.658373	5628.977	0.071144	14.97413
0.714152	0.269454	10.46086	3.131139	1.98002	6.549854	2122.208	0.047347	35.1403
0.490457	0.205138	15.33849	2.787102	1.008235	6.026738	1710.811	0.058985	21.3771
0.611338	0.270187	13.80411	2.708133	0.871517	6.271787	4944.913	0.049381	29.97702
0.48492	0.358398	10.04867	2.37693	0.568739	5.974837	14138.84	0.046336	33.98516
0.483956	0.208761	12.64435	2.683883	0.887264	6.047478	1463.455	0.055171	24.37232
0.440046	0.203339	13.04226	2.58489	0.745142	5.888304	5637.395	0.057729	22.68005
0.627364	0.160649	10.89598	3.1063	1.515514	6.560862	1134.389	0.052659	26.83305
0.442776	0.215765	12.74666	2.545937	0.758681	5.952299	139.3961	0.058991	22.73339
0.573755	0.240508	12.60254	2.879549	1.228563	6.350399	2114.01	0.049357	30.74139
0.628672	0.236112	12.08366	2.769334	1.139581	6.24375	4776.758	0.047519	33.35245
0.616121	0.215892	10.50605	2.847033	1.315474	6.453134	4273.667	0.050212	31.66185
0.650628	0.197633	11.325	3.081598	1.518613	6.47279	1023.686	0.056284	25.11101
0.649184	0.108324	13.59735	3.486809	2.641566	6.78467	5462.33	0.06387	22.40359
0.586898	0.247603	13.94184	2.694189	0.963994	6.00293	371.1807	0.055716	24.02751
0.497046	0.355481	6.730636	2.168656	0.445637	5.894709	2362.111	0.045809	35.44058
0.307272	0.26337	7.467972	2.306825	0.589803	5.505861	150.4828	0.068997	16.92506
0.427372	0.166909	9.611602	2.781843	1.207129	6.072616	295.126	0.067876	18.77441
0.700306	0.164322	11.49334	3.132704	1.635093	6.547558	819.0286	0.054755	27.41848
0.429897	0.246247	10.48038	2.567719	0.792063	6.097462	2037.759	0.054649	26.21734
0.543663	0.297846	10.40271	2.732808	1.033082	6.061785	463.2787	0.06067	26.41538
0.694287	0.236949	13.9907	2.83495	1.274996	6.397389	3461.026	0.048241	31.84793
0.529425	0.243883	13.56781	2.734908	0.910891	6.248169	768.9315	0.049973	29.54452
0.499561	0.206209	12.20071	2.731403	0.997851	6.202188	624.7016	0.053357	26.76467
0.46738	0.290322	16.23138	2.552375	0.795845	5.974609	791.1107	0.05225	27.82322
0.399806	0.464444	15.55973	2.152325	0.664804	5.685479	1125.546	0.04155	43.84954
0.369673	0.404302	11.56477	2.137157	0.483828	5.683952	346.8919	0.048079	32.81934
0.434895	0.286405	15.81588	2.630907	0.872927	6.11012	657.038	0.050328	29.50976
0.677826	0.287118	10.85955	2.727188	0.946864	6.319356	4865.396	0.047478	32.7313
0.63302	0.210843	9.994703	2.829193	0.980477	6.437946	11640.83	0.049707	30.11988
0.650362	0.204772	12.49686	2.946542	1.205971	6.539909	4799.58	0.048236	31.58956
0.417499	0.492349	11.62616	2.031013	0.455937	5.613472	582.3062	0.043563	40.0232
0.54254	0.246278	88.93686	2.49874	5.475895	6.16188	1079.367	0.049553	32.45947
0.70887	0.196837	18.13078	2.713126	0.946384	6.312991	6769.604	0.049102	30.50231
0.381167	0.291515	11.23465	2.409079	0.673584	5.922329	935.2373	0.050306	30.13993
0.381748	0.347795	13.19261	2.108836	0.452869	5.727891	3015.703	0.047975	31.79801

0.571249	0.147635	13.48208	3.038695	1.277709	6.228588	2097.544	0.06403	18.36572
0.523573	0.156318	9.838395	3.08397	1.560396	6.38382	805.018	0.060761	22.00846
0.555977	0.299498	15.12471	2.594914	0.897498	6.135632	5843.872	0.045289	35.25707
0.337376	0.339583	6.862576	1.958076	0.375491	5.628427	585.7987	0.048297	31.9788
0.416243	0.224949	14.09204	2.635543	0.840485	5.917298	883.3855	0.061223	19.98221
0.727334	0.202136	11.50464	3.026787	1.363966	6.622253	6808.992	0.049772	31.46146
0.43533	0.147664	8.221707	2.839985	1.146893	6.0788	522.6901	0.069451	18.7276
0.403532	0.321781	11.01764	2.084836	0.416681	5.787622	1723.723	0.046795	33.66262
0.264915	0.361446	8.623126	1.986666	0.390229	5.531788	259.5558	0.049168	30.30984
0.462742	0.304792	15.29412	2.298273	0.578824	5.914942	1037.866	0.050112	29.77504
0.571105	0.194695	11.132	2.991497	1.308282	6.505618	2276.092	0.053466	28.31196
0.408808	0.218526	12.87291	2.732026	1.139552	6.193234	732.9735	0.052596	27.38705
0.757883	0.258574	15.55634	3.040958	1.61637	6.5831	2868.338	0.048345	32.66227
0.554104	0.304012	13.50939	2.617699	0.851385	6.312895	4099.373	0.046908	35.38977
0.676717	0.363967	13.36523	2.585654	0.98588	6.159427	3029.971	0.042343	40.65152
0.718491	0.186161	13.44092	3.202903	2.194032	6.600037	1146.961	0.049429	32.52539
0.560355	0.221852	12.50067	2.749831	0.915714	6.171909	2680.762	0.055136	24.46722
0.734274	0.188034	10.39137	2.927863	1.115084	6.606079	14051.38	0.044619	36.76689
0.414786	0.282613	10.47094	2.492703	0.846325	5.859287	333.9247	0.056037	26.51456
0.450604	0.300843	12.37272	2.471335	0.714473	6.072887	863.4116	0.05191	31.05728
0.557422	0.121006	7.897397	3.098675	1.553688	6.317381	630.774	0.068399	17.75011
0.474232	0.302876	19.48772	2.654772	0.944056	6.180149	1579.849	0.047819	32.27412
0.502409	0.299328	17.87468	2.533247	0.85013	6.19755	928.0767	0.048479	33.47417
0.377053	0.303017	15.00044	2.014969	0.524618	5.446698	332.5671	0.05727	23.98886
0.617069	0.14388	18.76238	3.012256	1.303652	6.599343	7798.168	0.052149	28.74485
0.772498	0.256555	10.04418	3.194099	2.309005	6.492739	1291.67	0.051947	30.55949
0.627977	0.097534	10.49949	3.248785	1.883835	6.559782	897.721	0.059752	21.96591
0.348315	0.394423	13.4564	2.008934	0.392643	5.632957	1880.116	0.046565	34.07768
0.407441	0.29533	10.18958	2.404406	0.716355	5.788345	145.8915	0.058638	24.0653
0.399868	0.148035	16.52366	2.951684	1.365161	6.113826	643.0455	0.06737	17.41893
0.478323	0.219205	9.9783	2.607535	0.828867	5.484021	61.46292	0.078597	12.20948
0.433455	0.207095	14.22316	2.704617	1.179195	6.135456	385.9371	0.054419	27.32056
0.645793	0.165783	13.92075	2.953989	1.222226	6.529796	2475	0.051422	28.29641
0.435226	0.212593	14.54626	2.524846	0.763186	6.056777	2810.947	0.050144	28.98669
0.243178	0.227568	11.21886	2.336668	0.657093	5.581365	296.6774	0.061513	19.83807
0.283598	0.373385	6.619251	1.918762	0.392042	5.285909	88.03357	0.061562	21.10268
0.722966	0.087367	14.45936	3.744322	3.611681	6.837296	720.1049	0.073698	17.30376
0.501161	0.210802	11.20584	2.612173	0.831622	6.217201	1278.751	0.050416	29.79727
0.470222	0.344796	19.42828	2.257079	0.520576	5.979164	1518.731	0.046005	35.33812
0.459163	0.213285	14.76168	2.929843	1.488759	6.317269	2017.884	0.05487	25.67861
0.405316	0.369595	13.16962	2.348785	0.717023	5.936743	660.4161	0.045464	36.55618
0.522561	0.219613	13.21523	2.931649	1.569919	6.028573	164.3004	0.063856	18.8751
0.324393	0.382093	10.43846	2.034063	0.448099	5.555082	825.0852	0.045524	35.08743
0.522837	0.122746	11.10307	3.234793	1.975167	6.145449	99.83003	0.082368	14.83068
0.438312	0.467646	14.05205	2.082793	0.479457	5.873541	452.0489	0.040751	46.92616
0.565629	0.148391	17.84168	3.177988	1.761146	6.682711	1043.791	0.053743	27.72274
0.364836	0.208991	15.19453	2.767011	1.268317	5.906766	468.6003	0.061135	20.37129
0.663667	0.336083	13.0047	2.632985	0.906703	6.287396	2388.077	0.042967	40.12678
0.312235	0.304755	12.07031	2.438517	0.771222	5.904287	375.7141	0.052481	30.28638
0.505224	0.290558	24.56942	2.432071	0.660038	6.176509	3434.319	0.044582	36.48689
0.334382	0.187515	11.8469	2.777558	1.133396	6.071068	787.3425	0.063444	19.37449
0.538376	0.142126	10.99209	3.065772	1.377915	6.374919	517.32	0.060287	21.45636
0.44316	0.177572	7.81114	2.539798	0.914848	5.484145	79.93638	0.07946	11.89653
0.375049	0.262123	14.14153	2.426701	0.755713	5.666849	937.8183	0.058808	21.57739
0.355034	0.204514	16.26589	2.595443	0.902606	5.896644	1076.62	0.059614	21.0635
0.288967	0.230467	8.890194	2.277722	0.56194	5.515748	181.2439	0.065597	19.02391
0.704038	0.187183	13.50158	2.971009	1.289415	6.561628	6033.54	0.048618	32.12877
0.456048	0.182439	14.38257	2.822189	1.137672	6.149165	464.0444	0.058392	22.72937

0.503977	0.18513	12.82722	2.796685	1.022219	6.30759	2065.418	0.054192	25.64001
0.559025	0.244422	13.93532	2.778139	1.138591	6.251781	997.9087	0.052671	27.37129
0.286431	0.240523	11.40117	2.566058	0.951693	5.621237	376.8378	0.063677	18.9454
0.418945	0.380321	11.88453	2.356436	0.681608	5.834439	432.2064	0.047143	34.41254
0.405633	0.190352	16.59012	2.534007	0.843112	5.910449	2016.183	0.056441	23.22181
0.40566	0.214018	12.20563	2.600179	0.909305	5.836508	397.6855	0.05798	22.03081
0.544142	0.2069	16.53189	2.855706	1.134762	6.428334	2630.822	0.050134	31.54522

0.552346	0.202567	15.33245	3.100687	1.810297	6.366136	737.6305	0.058398	23.29975
0.578689	0.212391	17.09013	2.661571	1.859277	6.098288	25362.66	0.049599	30.18528
0.449978	0.327125	15.91041	2.602569	1.10307	6.000796	853.4292	0.048781	33.46365
0.478739	0.187348	14.0746	2.854119	1.320801	6.020227	348.3104	0.064778	19.10075

0.513409	0.549033	21.51945	1.906509	0.423528	5.55228	13709.79	0.042954	50.29082
0.602695	0.196424	12.45699	2.86324	1.110523	6.462695	2482.569	0.049169	31.58754
0.644766	0.149235	14.75487	3.014741	1.491822	6.503805	4017.455	0.052701	26.83625
0.439925	0.169289	9.59649	2.600471	0.987029	5.50608	113.7821	0.091172	11.5043
0.409988	0.261792	12.79875	2.492244	0.76849	5.953422	746.0449	0.053058	26.77021
0.507215	0.230921	12.78885	2.606319	0.821139	6.18563	3231.707	0.049215	30.17636
0.401968	0.176942	11.62879	2.751976	1.004441	6.12328	1664.134	0.057273	22.7088
0.400711	0.294127	12.86215	2.049602	0.673796	5.630918	327.6004	0.057243	23.48788
0.428979	0.282115	10.38216	2.420564	0.662253	5.927824	354.7902	0.054332	26.14501
0.807662	0.166184	22.1905	3.408314	2.272924	6.998551	2929.11	0.047282	34.75629
0.560443	0.242929	10.45251	2.471802	0.921054	5.340775	53.85452	0.070583	15.5995
0.501005	0.343472	9.691454	2.374299	0.55731	6.004417	4165.414	0.046198	34.06266
0.670989	0.162564	10.31113	3.004715	1.29408	6.471939	1759.341	0.052552	27.89968
0.570726	0.067434	11.97031	3.59367	3.544887	5.971423	66.09154	0.114148	6.624918
0.568264	0.158316	13.47731	2.997638	1.285403	6.383388	1197.945	0.057718	22.96258
0.634784	0.209607	14.09797	2.792746	0.983827	6.373398	4280.951	0.051636	28.488
0.625129	0.1656	8.872742	2.813839	1.048921	6.216869	1360.091	0.056962	23.46656
0.36633	0.273684	16.04525	2.51379	0.891246	5.87657	1219.276	0.054659	25.38412
0.56983	0.161524	11.92419	3.176178	1.946368	6.199291	118.3512	0.070447	17.27846
0.412571	0.202438	15.71741	2.778051	1.203982	6.071241	780.524	0.057223	22.98002
0.521336	0.276042	12.74448	2.931389	1.576602	6.390231	751.105	0.048856	34.95654
0.390514	0.118016	15.17836	3.186304	1.881562	6.323248	662.8983	0.069639	16.05406
0.702482	0.111039	15.61642	3.714589	3.860445	7.056059	688.7891	0.052866	32.84527
0.623016	0.173593	13.59316	2.339063	1.173418	5.119099	31.71186	0.107498	6.585533
0.331641	0.371998	5.334828	1.857356	0.332958	5.495338	688.3606	0.055016	25.96483
0.299277	0.307768	7.941312	2.267467	0.584343	5.510239	59.42244	0.065371	21.48633
0.443638	0.436025	9.346912	1.887589	0.350884	5.631409	1973.037	0.045377	35.83526
0.584262	0.353013	13.47691	2.550027	0.771105	6.145687	5880.298	0.044397	36.76523
0.371889	0.464173	9.36594	1.86315	0.335876	5.630726	652.3812	0.04134	46.35344
0.743545	0.224701	10.13491	2.790895	1.05579	6.451858	2313.418	0.044812	36.69481
0.186671	0.325689	7.145091	1.747897	0.30029	5.308469	455.0075	0.058612	22.71018
0.462048	0.351318	13.38577	2.438285	0.702916	5.906553	301.6263	0.04667	33.85272
0.450911	0.61234	86.24198	1.630242	0.473041	5.270577	483.5783	0.041587	52.53553
0.503617	0.385094	18.20429	2.334082	0.899011	5.91328	1252.724	0.042017	42.72579
0.456895	0.243467	15.12805	2.516208	0.67754	5.947807	8554.989	0.052999	26.38748
0.561437	0.241016	11.79907	2.616589	0.833464	6.270327	2868.845	0.048477	35.64983
0.368361	0.238198	12.77512	2.445688	0.790302	5.828134	318.071	0.053801	26.24417
0.272357	0.512543	6.488397	1.701669	0.276705	5.396637	331.5308	0.040754	46.53135
0.307473	0.374784	9.363109	2.034853	0.460316	5.605191	185.1571	0.051937	29.98266
0.522007	0.395351	30.02552	2.35963	0.632038	6.053506	4752.077	0.042168	41.88879
0.353249	0.314365	5.93175	2.192648	0.53637	5.210046	98.10345	0.071976	14.56405
0.490267	0.299935	16.54723	2.451798	0.774019	5.863901	3389.899	0.04994	29.22064
0.364041	0.384568	7.381267	1.710697	0.326813	5.103344	86.80556	0.07092	17.65513
0.430308	0.211569	14.50576	2.779983	1.354307	6.069261	367.2105	0.06304	20.39371
0.596784	0.235344	14.49061	3.454145	3.286685	6.66386	639.1062	0.057022	33.68366

0.776443	0.553119	57.31104	2.124007	1.0395	5.718675	2794.013	0.048903	56.71424
0.687828	0.201014	18.43795	3.287385	1.925482	6.935906	9777.887	0.045616	35.6263
0.700756	0.466978	64.61365	2.171614	0.501939	5.815426	8553.058	0.044584	47.39112
0.669569	0.34291	13.25319	2.127121	6.447486	5.712908	4234.992	0.048995	31.63983
0.331454	0.564922	12.13349	1.767984	0.301709	5.350236	1725.864	0.042398	48.82702
0.241991	0.511786	6.000106	1.837569	0.330501	5.374932	441.2979	0.043503	40.64923
0.332334	0.46731	11.51782	1.915714	0.371047	5.630042	522.4936	0.042486	41.55299
0.379025	0.415296	9.468242	1.885366	0.352615	5.551081	1905.224	0.047504	32.91913
0.34438	0.330744	6.263243	2.19838	0.587182	5.576247	307.3725	0.059823	28.22277
0.26636	0.386169	7.40623	1.970375	0.380762	5.541391	271.1124	0.046719	33.78814
0.529734	0.275732	14.49247	2.493698	0.751589	6.080159	668.2164	0.049424	32.83286
0.335261	0.481831	9.73547	1.950874	0.380874	5.615946	1222.866	0.042693	41.29786
0.516053	0.191037	8.098543	2.714415	1.293686	5.582254	417.7991	0.100264	15.70301
0.345041	0.472834	11.87168	2.013099	0.42891	5.584767	257.9902	0.042806	43.06577
0.330881	0.516681	10.16853	1.823868	0.322156	5.466696	1475.782	0.042393	41.84741
0.285127	0.462518	6.039042	1.966956	0.405543	5.457457	129.6421	0.048738	33.00898
0.35109	0.673109	5.986229	1.487202	0.245866	5.159728	476.7445	0.044849	54.03749
0.753782	0.228538	15.96	2.876541	1.299483	6.528698	4962.019	0.04354	38.29846
0.380372	0.439749	11.83288	2.199389	0.576895	5.740546	2446.838	0.042324	41.68087
0.356123	0.379537	14.37974	2.153009	0.549398	5.762931	1271.289	0.044904	36.34806
0.339035	0.532359	13.96219	1.858693	0.373202	5.438399	1209.993	0.041162	51.04179
0.726065	0.171572	17.69204	3.492395	2.632638	6.879485	7011.315	0.051832	28.81897
0.338245	0.660849	10.04849	1.507542	0.238817	5.082987	2119.363	0.047309	54.90906
0.612698	0.128389	11.30282	2.847693	1.694431	4.92099	38.09016	0.156107	3.090231
0.323141	0.225367	12.76501	2.18426	0.614241	5.49852	452.2554	0.062544	19.5049
0.498207	0.287579	14.62513	2.247585	0.562435	5.942999	3435.034	0.046351	34.05043
0.641686	0.255507	18.38093	2.513382	0.80493	6.123936	4292.269	0.048555	31.10187
0.778793	0.296088	17.73153	3.318053	4.649781	6.500541	1433.007	0.049595	37.49466
0.474242	0.440505	12.28373	2.540349	1.789947	5.812673	71.19143	0.047652	42.64733
0.861581	0.139929	13.34245	3.842409	10.12901	6.248653	82.60461	0.073231	13.92405
0.364472	0.445708	13.32726	1.787952	0.32547	5.474424	1094.348	0.045659	35.27162
0.518837	0.360471	17.96381	2.546276	1.488336	6.011164	245.9129	0.047264	35.29901

VX61	VX62	VX63	VX64	VX65	VX66	VX67	VX68	VX69
7661.88	0.928316	31.39251	154.0369	4608.501	5.513251	0.0363	0.023868	0.797947
10557.96	1.130496	10.96465	224.9781	1867.789	39.13798	0.143503	0.013337	0.197892
16855.85	0.344651	94.74819	291.9558	28319.23	3.030061	0.01075	0.016512	1.530512
454.2461	0.348244	9.012195	134.4013	1217.702	15.86758	0.136317	0.028535	0.219731
1462.505	0.865093	25.35506	139.7764	3619.355	6.027355	0.047429	0.032755	0.720455
6709.208	0.772856	22.59382	192.8447	4022.815	9.966587	0.052127	0.022225	0.523318
2242.11	0.903717	57.23219	124.3248	7167.255	2.230494	0.019226	0.039082	2.043815
4585.814	1.213674	41.20149	145.3245	5999.17	3.799256	0.028582	0.032224	1.157659
264.3533	0.551099	10.76923	99.35328	1075.943	10.72259	0.125065	0.03654	0.392091
11461.85	1.082029	91.20283	141.9447	13306.71	1.555974	0.011633	0.032468	2.76276
50198.5	1.006619	57.64562	200.0185	11410.9	3.731531	0.018877	0.021768	1.176275
154401	0.696829	29.95174	271.6515	7873.661	10.0973	0.036896	0.012307	0.38991
8095.505	0.983787	44.0797	175.8238	7873.492	4.225284	0.025402	0.022628	0.900393
1252.365	1.422835	46.10517	112.2887	5514.331	2.543462	0.025457	0.035874	1.473672
696.5105	0.723017	39.1465	137.1987	5444.639	3.569296	0.03073	0.030806	0.898938
35763.69	0.765421	65.87961	240.2917	15945.6	3.722126	0.016049	0.019843	1.197602
853.3896	0.923315	19.33687	147.268	2968.803	8.905811	0.067524	0.026201	0.525523
8849.838	1.403939	30.58245	165.7732	4546.827	6.991306	0.039421	0.024844	0.788831
6868.502	3.198074	86.55352	95.58057	8450.915	1.233319	0.014258	0.041892	3.246705
27772.46	1.897647	44.18644	209.3457	9073.108	6.245661	0.028385	0.022105	0.902925
35739.52	1.439599	34.95757	201.8612	6791.5	7.215805	0.034369	0.017694	0.643631
8342.878	2.04036	43.18003	133.0217	5590.675	4.070912	0.029244	0.032201	1.335972
1498.912	0.788435	33.00884	188.6727	6112.951	6.069311	0.03426	0.028827	0.942894
32135.16	1.701307	1940.774	210.4108	407234.6	0.113447	0.000655	0.013592	26.51601
13986.27	0.883095	53.17232	221.4532	11751.73	4.368307	0.020838	0.020073	0.960976
1770.912	0.511576	14.4362	190.215	2832.447	13.94102	0.085857	0.028293	0.395648
12495.58	1.163873	1693.193	311.2309	524863.5	0.184975	0.000748	0.014853	24.78815
6095.165	1.652005	45.30416	149.788	6789.567	3.936859	0.026271	0.022198	0.992893
3463.672	0.830654	41.22764	174.2134	7238.771	4.403674	0.02734	0.025626	0.902947
4055.135	8.821839	1941.66	141.5445	276706.9	0.308139	0.004421	0.030561	58.3577
1182.972	1.295501	45.27661	136.1682	6481.178	2.996639	0.02888	0.037477	1.479379
18045.79	1.023208	25.91932	193.1075	4857.788	8.936519	0.046635	0.019816	0.523282
1793.168	0.606199	27.81006	151.7355	4227.384	5.697243	0.040537	0.032882	0.87401
12287.26	1.399622	48.35476	178.0732	8139.162	4.317317	0.023495	0.019279	0.945982
110022.2	0.837753	21.56291	259.2266	5528.55	13.95077	0.055188	0.015261	0.358798
1854.46	0.646212	37.59676	147.4908	5691.227	3.981767	0.02964	0.022546	0.790775
103960.6	2.267275	232.5831	154.877	35115.71	0.708073	0.004474	0.027404	6.423335
1130.708	0.727324	14.3472	145.3436	1916.175	12.54097	0.088342	0.028392	0.475836
4168.842	0.350631	15.46717	239.2164	3776.141	15.50342	0.073309	0.025548	0.359657
881.5649	1.855666	43.38391	118.0634	5701.332	2.686585	0.036104	0.040516	1.339932
7707.769	1.488714	57.11681	168.2543	9976.835	3.012601	0.020521	0.030485	1.492971
49577.41	2.610422	68.79533	186.8238	12954.22	3.270665	0.017626	0.022204	1.439682
2203.314	2.338994	39.48805	107.9597	4110.791	3.621384	0.034161	0.031444	1.135973
30101.08	1.015287	44.97698	210.4615	9378.857	5.089323	0.024764	0.016662	0.744895
17800.88	0.618351	25.64554	223.5282	5714.164	9.475432	0.043617	0.019498	0.497094
1996.814	0.791436	25.18021	158.5063	4026.474	6.803409	0.046815	0.025265	0.552347
3173.388	0.931843	58.7256	186.2227	11350.08	3.16538	0.019085	0.019965	1.029337
10541.02	2.098746	1990.843	151.8841	301435.9	0.077409	0.000563	0.021742	43.21267
7702.11	1.234052	25.0543	183.0893	4665.03	9.019774	0.053296	0.020234	0.53335
11779.88	1.510062	65.18207	189.4513	12456.57	3.131387	0.017771	0.020683	1.246839
24240.39	0.84141	97.2062	210.7293	20948.83	2.154583	0.010921	0.023534	2.012744
1652.721	0.672454	19.49195	173.1775	3740.869	8.65999	0.066168	0.030851	0.494298
2379.994	1.104299	30.84849	143.7419	4623.091	4.95706	0.040033	0.029702	0.793174
5489.519	0.917752	97.79334	192.2128	19257.76	1.959496	0.010905	0.023635	2.038903
19045.19	1.696358	56.83926	195.9772	11593.59	3.634893	0.020216	0.025993	1.449995
11571.34	1.478758	90.14203	209.236	20003.37	2.2604	0.01377	0.026507	1.920479
4881.86	1.469284	58.61978	157.6781	9613.186	2.86134	0.019475	0.02526	1.362019

1578.241	1.069821	35.67691	132.3867	4936.672	3.780867	0.034051	0.035967	1.132999
1288.565	0.997294	19.78888	149.4965	2819.389	9.204041	0.066377	0.028227	0.563296
4816.567	1.200133	75.72225	172.2347	13619.02	2.254519	0.014829	0.02962	1.919832
141093.1	1.024572	53.91747	240.9094	12624.53	4.939028	0.020142	0.013199	0.74026
1482.335	1.25019	25.4433	122.2347	3000.03	6.232988	0.051585	0.029725	0.758695
11494.34	0.6683	35.25994	212.7703	7681.173	6.081155	0.032274	0.023311	0.727897
27097.69	0.700479	34.79019	215.1424	7444.725	6.644173	0.031633	0.020369	0.670058
14110.07	1.385762	28.85784	184.885	5130.464	8.820281	0.044389	0.023709	0.690117
2371.912	0.537268	29.72107	180.8912	5486.883	6.203318	0.037842	0.02806	0.72386
730.4366	1.327217	63.41658	102.8175	6883.092	1.603038	0.018007	0.04812	2.655843
727.3391	0.944734	15.72512	147.5779	2329.064	11.1836	0.089111	0.035079	0.6112
16600.37	1.235918	42.82808	168.039	6771.469	4.669188	0.02657	0.026459	1.128275
101488.9	0.790697	59.6824	205.6472	12109.9	3.634597	0.017745	0.019295	1.168685
17373.34	3.833928	47.71656	158.6332	6376.629	5.69689	0.029853	0.022133	1.204285
58023.43	1.110605	37.88806	248.6657	8883.138	7.697823	0.029962	0.019304	0.723117
44435.61	1.038717	1949.955	242.0625	472028.4	0.125626	0.000599	0.015694	30.32952
671.8398	1.130078	45.25619	140.3532	6674.755	3.049355	0.027793	0.038978	1.543516
4953.388	1.120237	51.73881	158.3634	8075.701	3.299877	0.021375	0.026675	1.293412
114703.8	0.731168	43.11398	262.1177	10926.69	6.621988	0.024937	0.011899	0.543371
3739.56	1.38274	51.17768	136.8587	6879.227	2.999799	0.022455	0.030348	1.537936
20526.15	0.891443	44.3326	191.8078	8208.126	4.756372	0.024574	0.021668	1.016814
5673.308	0.830134	24.50346	187.2026	4821.785	7.935077	0.049065	0.023086	0.507924
12618.15	3.00817	85.96244	91.99743	7534.804	1.268845	0.013562	0.045117	4.037459
11393.23	1.941458	29.65562	183.8038	5788.199	9.033535	0.052802	0.025466	0.650381
8351.797	1.136249	59.23876	139.5329	8466.327	2.395398	0.018823	0.034777	1.853422
31412.54	0.893109	48.41863	206.5263	10204.65	4.430138	0.022584	0.018949	0.880873
117524.2	0.580313	25.97737	245.0533	6212.044	10.21842	0.042453	0.01634	0.431005
8061.266	0.925195	40.60959	147.7459	6213.511	3.749556	0.027805	0.035362	1.321489
31763.81	0.800028	43.37862	166.7801	7157.345	4.058084	0.025074	0.025801	1.119641
4869.738	1.562859	31.08152	154.6127	5058.023	5.795563	0.042712	0.029939	0.756618
728.019	1.047781	42.20736	116.7241	4814.601	2.9828	0.02925	0.044571	1.814451
11559.45	1.330343	40.27219	169.37	7269.005	4.382303	0.029903	0.028098	0.953113
27752.86	1.173448	38.09016	201.5335	6769.74	6.634542	0.029736	0.021627	0.872985
24150.57	1.351174	29.29731	198.755	5662.803	7.978206	0.041978	0.01747	0.725152
4489.657	1.519224	34.13119	149.6467	5325.569	5.082195	0.038198	0.024474	0.782005
14975.2	2.630752	49.04336	132.8086	6140.689	3.561841	0.025631	0.020758	1.031617
2052.617	1.099581	48.68928	143.3557	7292.152	2.954579	0.023689	0.033919	1.420336
21449.46	0.462749	11.99552	264.0833	2954.428	26.63954	0.099754	0.015358	0.204295
803.0789	0.582404	14.47776	104.3874	1496.034	8.167111	0.084044	0.031433	0.533906
1103.394	1.328131	24.56417	106.9218	2445.944	5.341299	0.05162	0.032376	0.860218
3267.911	1.724834	35.15624	142.4204	4791.89	5.151476	0.036743	0.028934	0.999617
11809.26	0.806766	28.27052	167.8891	4663.485	6.532059	0.040219	0.018775	0.531404
2227.585	1.183021	29.35804	154.3043	4143.732	6.228528	0.040434	0.020394	0.629778
20749.29	1.323743	49.96087	187.7274	9647.304	4.071051	0.023217	0.022469	1.01275
4469.296	1.04217	47.05212	161.5861	7623.513	3.598726	0.023896	0.033575	1.425576
3396.57	1.09505	37.71677	157.2135	6224.365	4.296187	0.031232	0.02532	0.849517
4971.671	0.931051	67.33294	147.109	9686.277	2.294075	0.016355	0.033319	2.286202
12301.26	0.819548	60.7238	244.9251	15414.14	3.9711	0.018323	0.024589	1.381004
3075.09	0.558133	33.99265	194.8301	6727.617	5.795615	0.032165	0.022626	0.77525
4159.643	1.010096	63.52241	161.3628	10317.54	2.597479	0.017321	0.031695	1.741437
31205.69	0.974653	30.48604	208.1399	6495.729	7.485646	0.038747	0.018106	0.54777
64875.82	1.008463	26.51297	207.8886	5306.874	9.36274	0.044984	0.015406	0.440325
25879.06	1.225278	40.33537	194.0735	7854.599	5.441987	0.028775	0.023027	0.900905
6564.548	0.48639	34.31967	233.2617	8209.881	6.782956	0.031809	0.021099	0.658772
7217.248	17.61029	1968.648	200.2965	397363.9	0.311563	0.006089	0.018183	34.65503
38991.09	0.977713	84.09933	228.4371	18419.99	2.953133	0.012466	0.017813	1.557233
6475.021	0.778862	32.05121	167.0756	5466.322	5.367704	0.036202	0.026754	0.833852
26504.21	0.483728	43.97173	212.0442	9558.048	4.830592	0.023917	0.019302	0.827799

8090.502	1.287408	46.9137	126.5596	5806.252	3.019166	0.024101	0.034397	1.694088
2932.437	1.690046	26.92988	127.3508	2900.599	6.765562	0.048792	0.026338	0.889423
43600	0.946019	57.63069	205.4597	12146.24	3.591972	0.018677	0.026892	1.561236
5282.213	0.408062	12.49213	208.4158	2399.786	19.68621	0.092697	0.017092	0.244761
4398.276	0.917643	51.85266	115.2046	5825.968	2.378919	0.021388	0.039111	2.077275
34633.55	1.354469	35.05833	212.3738	7392.385	7.342231	0.035262	0.014297	0.518362
1983.854	1.12905	19.27013	109.9979	2023.946	7.653673	0.071933	0.027888	0.612725
15755.72	0.44996	30.81833	235.8865	7439.67	7.772097	0.035146	0.020293	0.594057
2290.752	0.42392	19.34041	186.4628	3383.967	10.75557	0.057303	0.024566	0.469748
7699.434	0.666488	59.05229	188.8764	11188.57	3.269449	0.017976	0.020083	1.09474
10844.96	1.343167	32.20122	167.5526	5495.944	5.848916	0.03898	0.019473	0.600729
3918.105	1.336914	41.25617	141.2955	6248.59	3.421334	0.030032	0.037061	1.241938
15661.51	1.690327	62.65244	200.1023	12322.84	3.637949	0.018607	0.019265	1.208422
27931.58	0.937004	46.45703	222.594	10640.87	4.904012	0.024022	0.016782	0.722743
25747.97	1.026226	45.52541	221.9463	10419.2	5.09686	0.025257	0.027729	1.124516
5455.551	2.146839	47.0849	166.2724	8538.689	3.979895	0.028437	0.025733	1.089508
13932.58	1.003347	40.50096	160.6494	6309.05	4.364729	0.027717	0.030656	1.250222
81609.39	1.129601	28.42985	256.5049	6982.544	11.03943	0.042221	0.015372	0.455823
2079.187	0.874715	29.10774	149.8367	4282.878	5.686967	0.041895	0.023828	0.711063
5562.374	0.807921	39.61588	179.9918	6889.859	4.924199	0.028184	0.021488	0.844389
1985.721	1.605869	18.19237	109.8868	1846.352	9.563162	0.088437	0.026452	0.541392
10614.21	1.08685	95.27635	178.5217	17547.89	1.856046	0.011158	0.027714	2.28082
6085.847	0.981309	80.0467	186.2285	15213.02	2.33308	0.013385	0.021813	1.572469
2512.334	0.650448	56.20389	161.5046	9445.74	2.826123	0.020122	0.033186	1.635005
35671.44	1.314747	88.94409	184.4561	16515.64	2.170934	0.011978	0.017314	1.492876
6015.904	2.22521	28.75693	177.3849	5142.343	11.52448	0.061225	0.020474	0.608867
2994.202	1.857634	30.05272	119.9764	3544.09	5.441659	0.045089	0.036836	1.034239
18125.99	0.443514	45.41014	233.4877	10844.84	5.126539	0.023193	0.022555	0.938037
773.4285	0.928033	26.68529	122.1294	3271.344	4.839108	0.045456	0.036246	0.998158
2370.394	1.518765	67.65511	90.04075	6381.656	1.334123	0.016721	0.050104	3.137072
232.4962	0.9397	25.21611	75.96419	2000.462	3.180224	0.049212	0.058169	1.263186
2069.549	1.376856	50.28017	140.6416	7711.274	2.742134	0.024028	0.032361	1.429441
12021.02	1.309579	49.84326	179.5903	8872.025	3.990727	0.022986	0.022421	1.060043
18508.12	0.852038	53.06663	171.375	9410.752	3.237494	0.020648	0.029161	1.443223
1643.811	0.750545	32.10637	108.5289	3510.005	3.511713	0.035313	0.044381	1.373815
612.8531	0.435824	11.18322	135.0098	1462.923	13.6486	0.108298	0.027996	0.320761
1337.908	4.11223	56.24562	97.51356	5937.454	2.178063	0.028731	0.032369	1.671066
7627.036	0.943328	32.85353	173.2773	5487.298	5.880776	0.035408	0.023749	0.804881
13027.79	0.555963	95.08464	225.6347	21415.85	2.411065	0.010851	0.019161	1.753928
9483.204	1.75062	54.49385	137.9432	8127.212	2.535374	0.022982	0.030467	1.380323
5598.812	0.807	43.38159	196.3734	9091.915	4.38858	0.026232	0.026119	1.000593
683.9056	1.887417	45.79285	95.5822	4463.218	2.285425	0.031481	0.057988	2.655933
7732.589	0.539387	27.36532	205.7239	5578.205	7.810484	0.041047	0.029839	0.692379
228.9769	2.437985	32.81518	66.38119	2211.678	2.391402	0.052942	0.063328	1.886531
4921.055	0.562347	49.3367	260.221	13006.16	5.296935	0.021672	0.02055	0.914233
4182.278	2.044879	80.90809	140.4179	11660.51	1.806503	0.014731	0.033179	2.376468
2156.682	1.529453	58.2334	100.0755	6116.37	1.713267	0.020635	0.056062	2.813134
18094.94	0.941639	42.93695	238.2985	10444.33	5.850446	0.026293	0.0192	0.779738
2422.612	0.883443	36.15798	147.6026	5535.326	4.09082	0.031882	0.030614	0.937908
27321.74	0.75689	151.2938	228.7509	34783.26	1.520769	0.006776	0.020455	2.902982
3306.585	1.236942	35.82385	105.0048	3903.781	3.080258	0.034672	0.042206	1.360516
1956.9	1.534464	31.1374	115.355	3576.151	4.253623	0.042193	0.039407	1.177389
273.5249	1.039734	16.17694	73.15905	1261.346	5.111739	0.088216	0.052809	0.842281
5530.777	0.824678	50.54455	114.0215	5776.587	2.324553	0.022315	0.044279	2.243821
5485.748	1.02767	66.5247	115.4631	7904.948	1.737776	0.016339	0.044845	2.736615
1008.629	0.598886	20.5067	94.73218	1985.109	4.860841	0.056315	0.050986	1.032843
30216.29	1.295429	47.03367	201.4008	9304.997	4.860501	0.0239	0.018305	0.887416
2131.583	1.348745	51.82899	113.1241	6163.836	2.186034	0.022535	0.047634	2.158448

10485.01	1.117341	41.97423	143.3574	6115.012	3.611686	0.027406	0.032951	1.286011
5447.025	1.259606	49.07421	157.7668	8018.636	3.351627	0.023524	0.02469	1.152439
1828.99	1.056355	32.79638	96.01588	3302.046	2.958248	0.037291	0.053712	1.649458
3485.031	0.833295	35.66492	178.7531	6529.747	5.049524	0.033194	0.02981	0.870882
11358.65	1.029831	68.69775	132.8548	9414.782	1.930703	0.015919	0.040526	2.587269
2094.12	1.10016	37.10016	124.1147	4974.994	3.272295	0.033334	0.042164	1.296595
14233.08	1.30001	68.68879	171.8546	12404.91	2.489254	0.016137	0.022504	1.387515

2940.922	2.260828	59.64286	112.6173	7070.267	1.956311	0.021813	0.047478	2.507057
171735.7	1.866286	74.51764	183.8875	13283.7	2.606961	0.014529	0.032632	3.642316
5853.318	1.406893	62.69534	150.4657	9975.721	2.329707	0.018603	0.040034	2.046693
1451.525	1.650134	50.17482	96.93863	5155.146	1.960481	0.026239	0.04612	2.114396

163785.7	0.501127	115.8193	336.17	39951.46	2.845376	0.008821	0.015856	1.71778
13514.66	1.16404	40.13604	183.3884	7342.025	5.071936	0.029166	0.022116	0.832405
17415.15	1.57378	56.21204	157.1613	8439.084	3.225908	0.02014	0.031394	1.709321
359.1811	0.997313	25.82933	63.1875	1621.693	2.802178	0.0481	0.055881	1.444534
4863.111	0.829285	41.83536	147.8391	6418.076	3.565157	0.026762	0.032008	1.316175
21030.84	0.961428	41.45939	183.3142	7636.747	4.626156	0.027647	0.026655	1.021509
8072.111	1.079064	34.47088	118.724	4172.539	3.66642	0.034757	0.045361	1.448584
2059.279	1.240489	39.51686	136.2602	5990.625	3.361417	0.035196	0.030735	1.013621
2240.121	0.727012	27.75268	148.6184	4096.732	5.728961	0.041384	0.02939	0.776569
11842.51	2.256351	126.15	199.2739	24611.14	1.760441	0.008591	0.018841	2.295813
266.5963	1.096585	28.19659	89.40629	2714.236	3.261248	0.056492	0.055805	1.498797
34534.61	0.572128	24.24643	222.5967	5294.298	9.96102	0.046164	0.020352	0.50413
8223.13	1.271645	28.59989	159.3368	4357.817	7.021735	0.043429	0.026055	0.77671
80.46459	4.122193	38.1399	38.54059	1666.295	1.442273	0.054458	0.086079	3.104332
5040.032	1.500625	46.98429	132.4646	6137.302	3.111377	0.025391	0.03346	1.499477
23041.09	1.050367	51.22758	195.3036	9697.989	4.18896	0.021332	0.017636	0.90307
6355.177	1.031805	21.33555	148.9296	2979.101	9.23324	0.059066	0.026775	0.612282
7176.077	1.20937	63.82189	129.0637	8555.29	2.006257	0.017751	0.041372	2.431925
348.0238	2.19994	36.80655	86.34643	3414.133	2.641502	0.042476	0.049588	1.622331
3828.767	1.444565	62.36122	123.344	7986.038	1.984861	0.018718	0.0483	2.452393
4005.292	1.941238	41.09282	157.0552	7163.998	3.792272	0.034674	0.034365	1.066233
1964.571	2.063337	59.11272	85.21515	5250.816	1.507993	0.021174	0.054338	2.641448
1843.003	4.340741	62.78594	125.1329	7947.48	2.489073	0.025075	0.058117	2.70837
70.41017	2.041023	46.41017	40.85763	2028.844	0.912624	0.035035	0.094662	2.736314
5762.413	0.360327	7.752797	199.7275	1484.055	31.39705	0.15851	0.015528	0.153669
323.4246	0.658471	16.16942	110.8086	1778.233	7.482888	0.076657	0.030947	0.524641
20755.24	0.388148	22.34611	268.58	6194.595	12.13957	0.048339	0.014839	0.340236
46950.2	0.795487	46.09387	233.3216	10911.06	5.233397	0.023564	0.017789	0.7756
7558.829	0.379541	22.03821	285.5703	6553.115	12.91888	0.049407	0.02279	0.448867
13876.87	1.092537	26.95762	241.1657	5992.162	11.1895	0.044082	0.017139	0.443573
3676.005	0.317417	13.12779	185.354	2478.183	14.99718	0.084917	0.021239	0.315155
2319.697	0.867439	44.81061	177.0702	8346.608	3.862056	0.025048	0.033876	1.293432
6539.161	1.551028	1861.55	312.608	579191.3	0.188849	0.001225	0.017514	32.8409
11680.16	1.27173	82.33393	232.0824	19669.4	2.778473	0.013679	0.030622	1.890299
55433.71	0.701116	57.61227	182.1152	10572.55	3.23381	0.018323	0.025224	1.413214
17536.25	0.94044	36.8124	221.7422	7507.76	7.000841	0.030312	0.014023	0.578818
1977.823	1.013767	41.24137	145.9526	6267.873	3.53427	0.028623	0.034586	1.199086
4147.925	0.305608	11.20738	271.9298	2693.766	28.6149	0.099976	0.022366	0.275321
1471.14	0.555963	22.16999	149.4325	3465.213	6.739892	0.051968	0.034996	0.746875
43714.06	0.744877	225.9358	249.2252	56090.76	1.113535	0.004527	0.021241	4.717087
505.2803	0.589115	9.558327	87.45781	809.5216	10.88391	0.141841	0.043509	0.514251
24278.12	0.947292	68.92699	172.7222	11802.62	2.583228	0.016008	0.032136	2.028848
586.2026	0.37197	14.3366	120.817	1748.588	8.925207	0.078473	0.02279	0.361161
1584.523	1.696166	53.81837	99.97236	5407.474	1.98207	0.023633	0.045715	2.308129
2230.355	3.73903	55.33315	134.3808	8237.621	3.10946	0.031861	0.036426	1.822262

28263.63	1.268281	820.6541	376.5151	312926.9	0.453744	0.001252	0.020581	16.20744
48030.9	2.214746	86.09656	192.6828	16231.08	2.442104	0.013525	0.025518	2.0668
82765.37	0.591633	1045.488	339.4186	352813.6	0.326864	0.000967	0.017418	17.95614
37981.18	5.911474	49.55211	231.1948	9383.235	5.852905	0.024885	0.024019	5.601685
22380.09	0.328421	37.22869	310.137	11287.31	8.594128	0.027923	0.020468	0.7614
5075.257	0.372444	9.315162	218.208	1987.067	25.11612	0.134167	0.026504	0.259163
5832.152	0.429154	33.63441	254.1464	8715.261	7.570769	0.031624	0.019971	0.644705
18817.54	0.398642	23.13147	249.1016	5758.019	11.20503	0.046436	0.016691	0.426395
1843.173	0.645721	11.01868	148.1888	1408.364	17.64329	0.11905	0.023443	0.343393
2544.431	0.433365	13.95416	194.9683	2844.311	14.22129	0.083239	0.03031	0.413121
4589.783	0.875919	52.96331	198.9148	10822.15	3.778858	0.020857	0.020249	0.96868
13430.35	0.436763	24.35321	248.4747	6072.054	10.4478	0.044605	0.016513	0.398145
1003.434	1.466534	19.34845	101.2577	1559.888	8.466054	0.071872	0.023707	0.533959
2762.825	0.502532	34.85532	207.2177	7474.303	5.830264	0.030927	0.030767	0.916254
18013.39	0.345638	26.04651	274.2689	7039.55	10.88008	0.040937	0.015982	0.402736
1228.431	0.449202	9.783835	170.7977	1554.635	20.01767	0.126566	0.026866	0.309681
6438.996	0.335461	9.646284	338.3294	3069.213	38.1449	0.119627	0.017472	0.236461
31975.29	1.324435	64.36704	241.4708	16414.06	3.846834	0.017272	0.020542	1.158699
24702.66	0.709126	35.19681	235.3139	8452.62	6.731159	0.032598	0.020695	0.692925
11713.9	0.682361	51.49235	220.2043	11436.43	4.323484	0.021035	0.026157	1.133323
15093.79	0.460794	49.03358	274.2472	13456.94	5.626641	0.021332	0.029024	1.378261
25079.26	2.765917	81.41596	153.2439	12132.7	2.166191	0.014703	0.02837	2.280968
28274.05	0.279599	25.38015	352.8695	8871.446	14.14064	0.041743	0.022342	0.572051
56.0082	2.210746	34.43033	27.19672	990.8648	0.990978	0.050539	0.082741	2.483379
2747.501	0.728477	40.60462	120.4781	5063.825	2.97505	0.027876	0.040455	1.555396
28730.65	0.672757	53.44497	228.495	12609.6	4.264741	0.020294	0.019896	0.992071
31575.36	0.828158	85.38821	222.8032	19521.55	2.615942	0.012344	0.018697	1.512225
6928.903	5.597209	79.65225	152.2872	14337.99	2.181444	0.023237	0.040379	2.601705
567.676	2.20093	39.97323	161.1861	5930.921	4.485133	0.032103	0.061249	3.44327
181.8706	10.56027	54.95745	71.24291	4269.321	5.148019	0.075037	0.072444	3.520042
11774.72	0.370553	44.28772	247.7271	11494.6	5.441287	0.024044	0.014814	0.586024
1841.256	1.937622	81.47742	167.4982	13848.91	2.167707	0.017642	0.033902	2.567922

VX70	VX71	VX72	VX73	VX74	VX75	VX76	VX77	VX78
0.001157	4099.481	0.270261	1.136205	32.03123	6.079629	185.6377	0.218568	0.036791
0.00209	5003.023	0.252767	1.200802	12.50744	13.64693	127.4944	2.210341	0.128942
0.000225	5709.167	0.395883	0.523542	92.89129	14.56904	1409.618	0.151931	0.0111
0.009384	245.2188	0.419149	0.466019	9.005711	6.722293	60.19982	0.836004	0.147092
0.002757	839.8052	0.282849	0.993996	25.00471	5.300756	138.5255	0.234318	0.049854
0.001777	3245.693	0.301096	1.003678	23.46888	7.752004	164.3477	0.406481	0.053268
0.00143	1315.953	0.282598	1.102123	57.0328	3.753096	215.3202	0.069641	0.019888
0.001597	2565.984	0.246346	1.448851	41.02864	4.840885	198.8085	0.132804	0.029969
0.0076	169.4753	0.349834	0.635126	10.71543	3.833626	41.67457	0.441675	0.133151
0.000483	6311.479	0.256372	1.295844	90.18285	4.348699	401.6399	0.049151	0.011935
0.000513	25478.67	0.263834	1.143484	57.79941	6.686666	381.5779	0.125928	0.019131
0.000495	61867.83	0.308124	0.848652	30.69873	11.96129	347.5321	0.44598	0.036798
0.000929	4334.143	0.269691	1.138747	43.71323	6.303091	279.6626	0.155123	0.026316
0.001392	797.2111	0.229107	1.532234	45.20553	3.968655	187.0302	0.096842	0.026454
0.002874	388.8759	0.340039	0.970291	38.63494	5.546293	218.1026	0.154128	0.034009
0.000408	14956.53	0.295762	1.02698	65.35384	9.470245	624.7771	0.149063	0.016541
0.002231	478.1823	0.282896	1.021813	19.06949	5.890036	117.3012	0.368597	0.070837
0.001161	4779.393	0.230295	1.508035	31.81728	5.839313	170.6454	0.235584	0.038535
0.000838	4621.052	0.156141	3.439697	85.85113	3.505242	305.5619	0.048062	0.014725
0.000817	13514.73	0.224268	1.822254	44.39895	7.110805	304.5477	0.214171	0.02798
0.00069	18628.78	0.223396	1.461322	35.62828	7.067388	243.9303	0.245558	0.033824
0.001195	5261.449	0.194155	2.019702	43.47721	4.23776	180.7129	0.126399	0.029012
0.001462	700.6686	0.291982	1.116285	33.29796	7.814991	257.9371	0.253125	0.035996
1.38E-05	16270.63	0.23656	2.033121	1944.029	9.494586	18403.05	0.00552	0.000735
0.000648	6486.728	0.294313	1.1614	52.8864	9.103446	482.1635	0.183721	0.021966
0.00438	809.7704	0.360949	0.696375	14.2679	8.011717	119.0233	0.608783	0.093653
0.000124	3792.848	0.420681	2.751772	1699.037	18.86536	31813.33	0.011648	0.001008
0.000663	3685.267	0.212978	1.658735	45.36677	6.346155	288.9964	0.165893	0.026286
0.001249	1782.129	0.303982	1.047646	40.92087	6.142164	255.4157	0.159508	0.028747
9.74E-05	2402.072	0.212599	9.609612	1937.096	4.905895	9532.079	0.027184	0.004807
0.002158	605.9557	0.258765	1.697194	44.19505	5.00288	233.7402	0.123958	0.032372
0.001153	9091.021	0.260024	1.157956	26.2869	7.070976	180.7899	0.325668	0.047151
0.00251	953.4507	0.333343	0.770317	27.7257	4.750958	132.1428	0.18458	0.042341
0.000515	6621.884	0.223005	1.487261	49.22565	8.334606	395.9538	0.195691	0.023443
0.000926	46623.24	0.272707	0.981314	21.75239	9.850107	211.7852	0.527585	0.056376
0.000995	1021.637	0.369557	0.795037	37.18481	6.250815	234.1748	0.178865	0.031057
0.000128	59912.11	0.176812	2.376569	234.8879	4.246835	971.3851	0.019281	0.004439
0.003918	632.4595	0.304989	0.878775	14.924	5.47318	74.22459	0.484046	0.089695
0.003352	1504.951	0.376945	0.566121	15.24871	8.516487	133.6601	0.57162	0.080313
0.003509	489.3415	0.220003	2.273228	41.46151	4.396151	199.7479	0.130401	0.041837
0.001024	3916.35	0.218904	1.845892	55.87757	5.906462	344.8122	0.112297	0.022034
0.000472	24817.14	0.182794	2.637878	68.42419	6.189877	430.7469	0.107229	0.017765
0.001754	1456.051	0.174673	2.442047	39.73043	4.063843	156.3638	0.139292	0.034824
0.000493	14305.09	0.259703	1.192562	45.18586	9.54212	428.3638	0.23174	0.025217
0.001081	8325.042	0.327549	0.729614	25.6823	8.17593	209.446	0.348868	0.044647
0.002113	1085.484	0.299153	0.940707	24.99135	6.324265	160.0121	0.281073	0.049314
0.000537	1513.603	0.283154	1.201268	57.43162	7.603999	455.2341	0.135625	0.020427
1.76E-05	6220.18	0.202609	2.098388	1992.823	6.612791	13082.67	0.003496	0.0006
0.001406	3994.743	0.247596	1.361995	24.956	6.686532	168.9741	0.337814	0.055316
0.000551	6037.384	0.228103	1.74666	64.67805	7.377009	482.0498	0.125544	0.018682
0.00037	10262.68	0.288387	1.17938	95.65627	7.816816	769.537	0.082007	0.011423
0.004801	848.1423	0.344663	0.836691	18.46912	6.929147	143.8743	0.393042	0.075847
0.002056	1321.084	0.251985	1.290862	30.25905	4.706397	148.3871	0.174245	0.042837
0.000383	2630.38	0.283913	1.194188	96.21122	7.383506	732.5069	0.077172	0.011428
0.000747	9869.229	0.206835	1.992143	55.55566	7.039696	407.644	0.135925	0.021287
0.000822	4679.686	0.267197	2.143656	86.62715	8.23314	771.2489	0.097157	0.01605
0.000639	2545.722	0.236483	1.597228	57.60336	6.115855	363.448	0.115549	0.020102

0.00207	873.8415	0.266825	1.288551	35.07687	4.197975	151.7459	0.133478	0.03633
0.003792	724.615	0.256286	1.169805	20.1409	5.686675	108.7105	0.363994	0.06991
0.000696	2292.554	0.254114	1.530907	73.99251	6.879829	534.3791	0.094495	0.015879
0.000284	64720.48	0.263458	1.145724	54.93549	10.59896	557.5996	0.216836	0.019954
0.001945	936.0458	0.246658	1.335078	25.70445	5.404612	135.8634	0.274467	0.052148
0.00116	4677.255	0.318719	0.943351	34.62922	8.919325	319.0249	0.267447	0.03483
0.000841	12477.27	0.304993	0.836383	34.82062	7.725349	267.2927	0.24034	0.032295
0.001266	7597.577	0.238203	1.365317	29.30511	6.075332	169.3125	0.287967	0.043288
0.002317	1197.176	0.365027	0.706669	29.30369	5.888152	177.4431	0.209478	0.040472
0.001574	458.9591	0.240422	1.570825	62.29702	3.688559	239.4046	0.062209	0.018971
0.005496	398.6158	0.265278	1.130227	15.78654	5.424902	84.79982	0.449432	0.094979
0.000864	9391.623	0.239788	1.320079	43.74166	5.194437	213.9614	0.142235	0.026313
0.00038	47807.11	0.28566	0.947682	60.15436	6.571046	389.0188	0.116184	0.017818
0.000605	10023.04	0.14411	3.722761	50.62458	6.629939	286.0002	0.222233	0.027627
0.000685	26263.55	0.244665	1.26252	39.09498	9.921026	353.0788	0.310149	0.029625
3.61E-05	19617.83	0.308697	1.436511	1949.431	9.534063	18589.16	0.005168	0.000679
0.002688	342.1327	0.269259	1.51333	44.08355	6.017208	282.8029	0.142931	0.031346
0.000797	2727.636	0.243227	1.314979	51.91406	5.696943	293.3492	0.119497	0.021815
0.000308	47244.28	0.304724	0.889706	44.1644	14.38862	602.3613	0.364537	0.024751
0.00092	2252.727	0.227949	1.52416	51.43649	4.593286	234.0246	0.101171	0.022861
0.000615	10300.12	0.273683	1.064295	45.12994	6.913134	299.5919	0.170892	0.024556
0.001628	2736.553	0.284163	1.007388	23.94732	8.059746	196.1764	0.384536	0.052562
0.000715	8551.897	0.154222	3.18248	86.99758	2.755218	232.2361	0.037589	0.013549
0.001969	5332.715	0.207879	2.039922	28.72992	7.569533	233.8797	0.377335	0.05487
0.001079	4577.352	0.254366	1.408877	58.54977	3.813273	228.94	0.06828	0.019671
0.000513	14715	0.278565	1.060291	47.87793	6.944455	339.1189	0.152353	0.023276
0.000838	46410.23	0.317072	0.76505	26.39489	9.264969	236.4752	0.387301	0.043157
0.001395	4458.155	0.274777	1.087133	40.04296	4.211428	174.351	0.111498	0.028961
0.000865	16111.1	0.286032	1.002966	43.60224	4.593813	198.0476	0.113096	0.025533
0.00254	2796.186	0.214321	1.695156	30.26633	5.16434	165.8807	0.203578	0.045727
0.002771	438.5635	0.277749	1.299861	42.48488	4.012972	168.9796	0.108365	0.031226
0.001448	5840.912	0.232308	1.562492	39.01426	5.992902	250.1943	0.165134	0.032125
0.000733	13896.89	0.256249	1.218131	40.32935	6.755589	234.9406	0.216882	0.028291
0.000803	11756.57	0.255535	1.819717	30.13451	11.04086	316.6523	0.455252	0.042914
0.001329	2518.203	0.226855	1.664984	33.48444	6.297177	220.217	0.226419	0.040162
0.00059	9262.435	0.170871	2.646379	49.85552	6.925506	330.8853	0.181493	0.025374
0.001558	1105.028	0.267873	1.374751	47.68931	4.133685	205.0341	0.091517	0.025252
0.002013	8743.232	0.37029	0.583468	12.59873	11.95859	135.6475	1.206407	0.10007
0.002908	509.523	0.342467	0.661902	14.59798	4.534927	64.74992	0.371983	0.085803
0.00218	688.8585	0.23358	1.50691	24.9993	5.589698	137.6438	0.277316	0.052558
0.001509	1913.968	0.203756	1.883343	35.5264	5.549846	191.9214	0.200453	0.037483
0.000943	6194.59	0.28555	0.955718	28.49891	7.824932	218.3126	0.309854	0.040982
0.001107	1133.162	0.242778	1.458576	30.33088	9.655524	274.8146	0.383385	0.040961
0.00069	10011.09	0.249757	1.526012	49.14749	7.441467	377.242	0.166788	0.024107
0.001278	2338.905	0.254018	1.308972	46.81423	5.247773	247.1081	0.12049	0.024928
0.00135	1818.53	0.256908	1.321336	36.75451	6.964605	268.4555	0.202754	0.033483
0.000666	2600.684	0.277829	1.183497	67.83765	4.614864	307.8053	0.073312	0.016772
0.000621	4200.248	0.313484	1.26437	59.15035	9.415741	585.4993	0.159936	0.01989
0.001097	1362.729	0.341689	0.785834	33.74177	7.719995	266.6538	0.23477	0.033783
0.001007	2089.28	0.26759	1.321789	63.11903	5.375235	343.8724	0.088869	0.018115
0.000923	14421.56	0.265039	1.146218	30.13079	8.7615	273.1969	0.319836	0.040505
0.000772	31886.24	0.258647	1.150122	27.07324	9.192675	238.7449	0.412179	0.045133
0.000894	13226.85	0.241962	1.35693	40.23258	6.870386	277.0898	0.195146	0.029395
0.001062	2467.507	0.360631	0.733375	33.66745	10.16544	356.5284	0.301757	0.034131
0.000306	3403.056	0.287365	26.11979	1957.074	11.02652	21819.93	0.036079	0.008861
0.000241	18671.16	0.272476	1.037553	86.12362	7.867637	641.237	0.100836	0.012223
0.001348	3231.518	0.294348	1.013761	31.76497	6.149972	199.3302	0.208339	0.038392
0.000558	11956.53	0.362621	0.631159	43.41188	8.168718	366.2772	0.18858	0.024624

0.001006	4885.948	0.233658	1.438552	47.30345	3.485074	161.6064	0.083566	0.02427
0.001273	1756.109	0.205463	1.86436	28.45242	5.899032	148.1962	0.298308	0.047374
0.000649	19419.95	0.279977	1.298389	56.96655	6.777854	398.4189	0.120749	0.019403
0.001825	2548.992	0.395451	0.510791	13.0344	8.905776	105.6848	0.834074	0.092699
0.000981	2658.666	0.277479	1.077012	52.23442	3.675581	189.5561	0.076708	0.021745
0.000573	16969.01	0.237285	1.483237	35.20867	11.91097	417.9471	0.414349	0.035838
0.002367	1281.44	0.25241	1.21825	19.61457	5.41535	102.3762	0.383233	0.072672
0.001029	6912.493	0.380569	0.590232	30.29955	8.50841	268.4594	0.28361	0.037004
0.001951	1132.097	0.383413	0.54322	19.88726	6.425017	119.2758	0.370186	0.057745
0.000588	3702.115	0.32111	0.87718	58.7827	7.916883	469.689	0.138451	0.018525
0.000985	5709.884	0.229847	1.552712	31.92481	8.099357	262.7528	0.301033	0.040899
0.001969	2110.534	0.241199	1.629489	39.99961	4.568278	193.9147	0.125192	0.03267
0.00049	7341.959	0.227594	1.994375	63.0818	9.719489	599.9344	0.179018	0.019171
0.00053	12156.65	0.270273	1.210774	45.58443	12.70967	599.1603	0.289744	0.025457
0.001067	10185.41	0.273122	1.335603	44.63599	7.617005	354.8295	0.179311	0.02678
0.000981	2787.332	0.201819	2.341178	45.3427	6.434793	316.4048	0.168457	0.030374
0.001211	7413.764	0.2587	1.19747	40.97173	4.754047	190.0445	0.129692	0.028196
0.000768	36636.69	0.247497	1.242073	29.16999	11.66192	316.9651	0.505167	0.042102
0.001316	1116.695	0.30289	1.088776	29.3317	7.41277	213.2251	0.299977	0.0435
0.000825	2741.448	0.288278	1.028637	40.07278	9.936007	388.8543	0.272321	0.029083
0.003199	1294.561	0.209278	1.68843	18.62317	4.981303	88.13243	0.430466	0.089912
0.000465	4998.142	0.262403	1.432209	93.51617	6.694834	649.5727	0.071555	0.01164
0.000496	2887.719	0.266724	1.305371	79.00076	8.802984	712.1527	0.113175	0.013999
0.001297	1337.836	0.39088	0.876061	55.04531	4.781657	274.9854	0.088917	0.021711
0.000243	19305.16	0.231114	1.421535	88.72414	8.677712	770.6456	0.103405	0.012097
0.001453	2955.461	0.20722	2.18435	28.7164	8.871819	251.0178	0.599917	0.059722
0.00227	1952.951	0.197354	1.875371	30.16405	4.468603	133.8949	0.201121	0.045339
0.000727	7494.685	0.373924	0.623741	44.70636	7.797254	360.6833	0.173574	0.024182
0.002902	441.649	0.267269	1.176059	26.70736	5.121911	135.0908	0.21992	0.048166
0.001228	1548.094	0.227749	1.743635	66.80059	3.172286	217.4524	0.050802	0.017325
0.005733	160.1154	0.278372	1.057582	24.81957	2.888876	74.54126	0.131794	0.051896
0.001125	1144.282	0.255473	1.633882	48.50346	5.558933	290.5147	0.122196	0.025994
0.00067	6402.562	0.234354	1.497783	49.88752	7.01483	349.0074	0.157175	0.023598
0.000838	9503.453	0.292249	1.072165	52.17875	5.406249	292.129	0.106369	0.021636
0.002415	992.9321	0.304136	0.911743	32.06731	3.380387	108.8661	0.114753	0.036469
0.004174	347.5927	0.38379	0.529291	11.3467	6.13803	67.4269	0.639817	0.112272
0.001629	901.2848	0.133914	4.277305	55.3818	5.219816	291.1454	0.143102	0.030598
0.001092	3953.978	0.2696	1.158839	33.33774	6.977669	223.6498	0.242847	0.036415
0.000254	5755.335	0.340746	0.738037	94.94653	11.10324	1060.234	0.118435	0.011036
0.001163	5139.941	0.22178	2.083272	52.77484	4.785071	268.819	0.101141	0.024922
0.001368	2487.761	0.307786	1.074778	41.80435	7.665234	347.2722	0.181498	0.028755
0.003529	422.3947	0.233896	2.231864	45.59809	3.259201	149.2896	0.093867	0.033959
0.00202	3366.617	0.34675	0.771919	27.28254	6.484039	175.5852	0.254785	0.043605
0.007599	160.7463	0.172484	2.763564	32.57836	3.084536	102.195	0.138729	0.058122
0.000628	1762.085	0.331418	0.861818	48.72075	13.13082	647.1891	0.274208	0.022934
0.000757	2379.92	0.193629	2.459274	79.57508	6.0129	498.7969	0.0812	0.015756
0.001947	1287.81	0.241638	1.857882	57.24732	3.01169	178.9663	0.057708	0.02188
0.000661	7508.586	0.269907	1.166345	42.44144	11.50264	499.1932	0.288938	0.027598
0.001588	1264.937	0.28041	1.144553	35.24717	6.773079	251.0399	0.197641	0.034362
0.000172	11825.58	0.303818	1.025109	150.3662	8.887438	1347.631	0.059495	0.006897
0.002269	2048.443	0.241609	1.437155	35.34434	3.661963	133.328	0.118383	0.03663
0.002596	1206.168	0.211103	1.761019	31.13825	3.869835	119.424	0.155067	0.044392
0.007348	189.3913	0.253157	1.164245	15.96953	4.056429	67.31818	0.323416	0.093456
0.001332	3252.491	0.304699	1.013079	50.45916	3.362491	169.9114	0.072162	0.023095
0.000986	3256.639	0.271338	1.241426	65.7691	3.547336	239.0253	0.055876	0.016866
0.00393	660.5739	0.33785	0.689762	20.43619	3.502312	72.58897	0.18896	0.057893
0.000467	15550.19	0.232067	1.361222	47.49038	8.995269	414.3893	0.21835	0.023843
0.001818	1277.694	0.238458	1.611062	50.88648	3.641101	192.0964	0.076924	0.023813

0.001343	5953.34	0.251044	1.314355	41.60848	5.208927	221.0491	0.136432	0.028571
0.000742	2819.637	0.248587	1.488488	48.31591	6.11314	303.2956	0.138013	0.024604
0.003364	1105.961	0.266377	1.265153	32.33385	2.989357	99.59871	0.104015	0.039433
0.002063	1565.675	0.29316	1.178756	34.98457	7.768133	281.3164	0.232071	0.036651
0.000935	6371.312	0.280497	1.282719	67.81861	3.917468	273.2349	0.059634	0.016561
0.003011	1195.426	0.268311	1.341199	35.98527	3.740275	142.6355	0.112909	0.036421
0.000496	7296.209	0.237921	1.591114	66.94037	7.411953	525.1438	0.112163	0.017047

0.001567	1683.637	0.198697	2.671928	58.562	3.640138	220.8998	0.074331	0.023316
0.000498	81915.86	0.285166	2.903359	76.29735	5.373699	392.6614	0.076938	0.014731
0.001414	2860.519	0.259906	1.893207	60.78171	5.429386	349.9064	0.09122	0.02031
0.002291	904.9344	0.240151	1.956295	49.24405	3.423125	175.3644	0.080995	0.028476

0.000165	42267.86	0.340395	0.87554	112.3847	20.52802	2436.998	0.174357	0.009249
0.000909	7184.528	0.250519	1.335024	39.97712	8.079392	322.771	0.229421	0.030321
0.000808	9912.159	0.21722	1.700709	56.95655	5.053698	280.4444	0.10165	0.020194
0.00402	263.3616	0.272768	1.102016	25.7695	3.899436	104.0406	0.17206	0.049585
0.001166	2645.05	0.30369	1.025966	41.22239	5.395451	230.1198	0.136576	0.028068
0.001225	10208.13	0.27624	1.252942	41.23792	6.190247	256.453	0.163136	0.029244
0.002572	4926.641	0.255922	1.259612	34.16399	3.583811	124.5325	0.117804	0.036482
0.002096	1151.011	0.31566	1.542123	37.74615	6.59519	274.0144	0.191592	0.040468
0.001611	1222.419	0.30398	0.893686	27.79243	5.946588	163.5827	0.23817	0.042945
0.000189	6330.278	0.18732	2.203002	127.1121	8.524819	1055.189	0.075015	0.008518
0.005399	171.3115	0.312739	1.337665	27.42764	3.523065	103.6371	0.163239	0.064221
0.001155	15123.03	0.327182	0.736505	24.50871	8.357436	199.785	0.377488	0.047363
0.001295	4796.182	0.238141	1.351773	29.03609	6.07344	169.8461	0.262834	0.043549
0.008301	64.12932	0.134833	4.274472	37.47971	2.243511	86.65203	0.112749	0.057381
0.001231	2931.344	0.222212	1.767889	47.12571	4.547859	212.0787	0.111718	0.026297
0.000449	11605.77	0.258755	1.203394	51.84858	9.095637	461.4961	0.193322	0.021451
0.002008	3814.666	0.261674	1.073913	21.83905	5.337783	110.649	0.319139	0.058474
0.001093	3895.93	0.270059	1.527383	62.75928	3.880495	251.2538	0.065027	0.018709
0.004411	228.5949	0.187051	2.476484	35.90289	3.776941	144.3422	0.136846	0.046823
0.00169	2197.35	0.246364	1.807471	61.24062	3.53942	224.827	0.061614	0.019948
0.002915	1913.104	0.201633	2.421448	38.89876	6.215548	271.6008	0.178235	0.040326
0.002499	1314.153	0.192086	2.338379	58.28224	2.900596	174.7166	0.056974	0.022411
0.002914	1130.659	0.12966	4.768895	61.75525	4.125858	252.0072	0.095588	0.027529
0.008905	54.8963	0.224222	2.338941	45.42006	2.783855	131.7131	0.080691	0.038926
0.002601	2805.99	0.419297	0.43774	7.967683	8.834087	68.51311	1.347433	0.159724
0.003222	193.7176	0.31331	0.795937	16.22581	4.368644	70.43599	0.314838	0.080326
0.000907	7896.796	0.400733	0.543045	21.85514	11.75868	271.1971	0.535988	0.050916
0.000528	18725.52	0.282877	1.025721	45.66655	9.534648	444.4661	0.216581	0.024366
0.001708	2694.042	0.38237	0.563492	21.23321	11.65224	266.0666	0.535749	0.053621
0.001039	6341.771	0.250728	1.19447	28.05683	10.242	256.5118	0.475254	0.043442
0.002349	1898.299	0.444995	0.373537	13.03832	5.701448	76.04603	0.46782	0.08734
0.001442	1063.747	0.284233	1.171015	43.59194	5.495964	252.6247	0.127942	0.026959
3.85E-05	1820.821	0.37081	3.02657	1870.584	17.53634	32495.87	0.012516	0.001894
0.000815	4349.771	0.285289	2.051647	79.82555	8.295129	692.7783	0.104194	0.015234
0.000553	27389.16	0.303573	0.872096	57.39262	5.285998	305.9322	0.094997	0.01866
0.000463	8055.536	0.262803	1.169614	38.57586	15.56754	541.6861	0.484429	0.029794
0.002226	1040.551	0.283249	1.345611	40.37582	5.19012	218.2616	0.135931	0.03116
0.002897	1573.623	0.417229	0.43113	12.20243	11.53515	117.7186	1.199658	0.097065
0.003096	777.6249	0.350251	0.718822	21.78673	5.242837	119.3595	0.251411	0.055362
0.000109	16296.13	0.299053	1.096766	226.305	9.830649	2211.501	0.044159	0.004591
0.008927	326.7405	0.333106	0.697167	9.76097	4.60991	42.1909	0.626434	0.146813
0.000839	11550.2	0.294407	1.305808	68.89925	5.099311	349.1613	0.078527	0.016753
0.002475	351.2221	0.431834	0.460475	14.41103	8.000826	114.9674	0.605821	0.080383
0.001829	963.2316	0.237082	2.056341	53.59239	3.711872	200.4991	0.081521	0.025028
0.00181	1111.331	0.151189	4.29106	53.06217	6.319957	376.5161	0.166192	0.035168

5.13E-05	5929.149	0.294337	2.642162	806.0124	22.15562	18417.98	0.026781	0.001333
0.000459	23250.76	0.195155	2.782064	86.6355	7.210444	610.0222	0.094389	0.01411
2.53E-05	23013.74	0.314032	1.010791	1050.547	18.51327	19224.46	0.01787	0.000978
0.000619	16341.6	0.380744	11.60093	58.42237	8.051523	346.0968	0.203254	0.024671
0.000704	6206.161	0.364374	0.593007	38.04887	14.78301	539.2764	0.41076	0.028241
0.005786	1978.269	0.366699	0.573392	9.461128	8.572893	78.19863	1.04376	0.148203
0.000942	2162.142	0.364488	0.663485	33.24981	11.42955	392.2394	0.343381	0.033181
0.000898	7435.569	0.391652	0.562152	23.2503	9.275145	216.5266	0.41661	0.047491
0.002764	982.8863	0.306914	0.795064	11.67618	9.647473	101.2703	1.118253	0.119298
0.004363	1192.203	0.37175	0.583738	13.64936	6.133363	88.36926	0.467778	0.08975
0.000759	2159.496	0.288039	1.233966	51.94052	11.13872	600.0402	0.217619	0.022442
0.000971	4843.562	0.346511	0.664228	24.24846	13.62125	336.8165	0.574092	0.046814
0.002075	649.2682	0.225513	1.541458	20.5905	13.15449	219.0154	1.061859	0.069358
0.001461	1119.913	0.337255	0.735125	34.01063	7.238109	257.0848	0.211054	0.032887
0.00084	5948.777	0.374606	0.557277	26.28418	14.2557	365.451	0.571937	0.04233
0.004778	576.5168	0.36419	0.619339	10.14411	7.212048	68.11826	0.861715	0.132277
0.002354	1531.542	0.361614	0.70675	10.38279	23.70704	218.2567	2.678558	0.128862
0.000492	13846.02	0.246946	1.494393	62.01052	9.910069	668.6615	0.160265	0.018251
0.001026	8763.554	0.3008	1.088918	34.70784	9.283295	329.9431	0.279757	0.035623
0.00089	4789.633	0.326452	1.005721	50.87379	7.947121	409.6012	0.160712	0.02224
0.000783	4554.805	0.33339	0.810833	48.96368	10.36401	507.8969	0.21508	0.022118
0.000496	13633.57	0.165313	3.078149	81.96439	4.975764	397.6462	0.071765	0.015026
0.001098	6446.265	0.381314	0.596883	25.70144	20.16709	505.3092	0.820008	0.043979
0.007661	46.37264	0.222367	2.324892	34.08172	2.210505	78.57256	0.09789	0.053459
0.001884	1622.152	0.343652	0.898569	40.09225	3.855437	158.5424	0.101944	0.029275
0.000532	12519.89	0.336981	0.904419	52.26132	8.480892	461.0501	0.164057	0.021483
0.000306	14200.66	0.317844	1.023298	84.01249	7.749402	673.4444	0.092477	0.012765
0.001435	2928.578	0.162216	6.355236	73.97611	5.183185	449.5783	0.096796	0.02668
0.002763	246.2837	0.259799	3.345207	41.64966	5.993671	228.6139	0.174286	0.034815
0.004459	123.249	0.14415	10.17471	53.65034	3.176511	185.6635	0.224535	0.07568
0.000514	4892.54	0.425656	0.503817	42.749	10.39652	478.2045	0.23326	0.025768
0.001197	848.452	0.271818	2.697559	80.72287	6.178105	505.8224	0.092467	0.020144

VX79	VX80	VX81	VX82	VX83	VX84	VX85	VX86	VX87
3.714783	7447.568	0.453293	0.602087	2.200615	0.672803	21.8477	0.025153	75.30049
4.064493	8714.302	0.383299	0.509839	6.059216	0.592068	8.203635	0.069758	45.73729
3.683106	4771.818	0.311821	0.430141	7.197296	0.559892	51.31888	0.006382	130.313
2.788177	289.9662	0.459637	0.627239	2.546071	0.663797	5.92658	0.10496	7.75
3.511031	1515.319	0.473253	0.624018	1.832087	0.692431	17.18327	0.0359	22.81651
3.763631	4715.261	0.409093	0.552515	3.328411	0.644481	15.5157	0.035463	57.82609
3.470011	2355.532	0.490773	0.647662	1.115241	0.718649	40.81986	0.014776	44.32766
3.827369	5092.292	0.462315	0.61553	1.627062	0.690117	28.06919	0.021719	68.21905
2.90023	263.6355	0.509509	0.682336	0.997119	0.707546	7.567737	0.099849	5.727273
3.702361	12145.54	0.472956	0.621636	1.406283	0.701568	62.61074	0.00857	164.4899
3.971491	39087.48	0.38648	0.535639	2.559259	0.628352	36.22978	0.012271	481.4549
4.156811	66904.15	0.310369	0.445518	5.284362	0.546391	17.10524	0.020141	657.0999
3.855226	7233.952	0.420453	0.57058	2.287778	0.650772	28.19182	0.017749	70.56854
3.656613	1950.366	0.525424	0.671036	1.202272	0.733009	32.5774	0.020054	20.19697
3.265341	574.3535	0.466561	0.626697	1.950506	0.685131	26.0388	0.0256	7.380952
4.012754	19289.2	0.356909	0.489712	4.08154	0.598914	38.63542	0.010262	289.9673
3.533081	827.6146	0.454073	0.613441	2.125201	0.673106	12.79152	0.049123	12.84906
3.932307	9875.849	0.442487	0.586292	2.10905	0.668248	21.97873	0.025214	87.28205
4.169489	17823.06	0.567943	0.702597	1.060753	0.766256	65.2646	0.011591	124.6101
4.184839	24789.06	0.393522	0.529388	2.923213	0.636335	28.01637	0.017971	294.9111
4.149885	33536.67	0.37957	0.530897	2.653414	0.616077	22.35358	0.020552	274.9095
3.98335	13690.71	0.483637	0.634624	1.363402	0.709292	30.95904	0.020529	152.4207
3.738114	1047.092	0.409599	0.562461	3.247787	0.643866	21.67683	0.024231	16.21739
4.187252	28634.61	0.374706	0.521185	3.701277	0.595987	1160.466	0.000489	135.5601
4.004157	8627.289	0.364677	0.512447	3.838768	0.603049	31.57219	0.014082	107.4448
3.412698	963.3648	0.404275	0.558308	3.380945	0.640371	9.113528	0.063942	25.58696
3.632673	2850.547	0.295407	0.412198	10.06713	0.53733	914.6067	0.000728	87.5913
4.002217	8784.577	0.459356	0.608122	2.309243	0.674492	30.71392	0.017712	53.31832
3.710782	2587.193	0.414789	0.572293	2.242243	0.648457	26.3287	0.01959	36.56923
3.958741	5679.346	0.471608	0.621287	1.64927	0.695822	1343.445	0.004196	60.57068
3.643797	1227.226	0.494973	0.639272	1.848271	0.71429	30.94323	0.025267	18.16814
3.979246	14782.33	0.395328	0.54595	2.715694	0.629215	16.76013	0.029986	139.791
3.35669	1338.779	0.449203	0.60653	1.568822	0.682773	18.84082	0.030191	30.19048
4.03625	14181.2	0.429306	0.569903	3.391186	0.646382	32.32332	0.015096	88.71537
4.155435	57714.15	0.32347	0.460209	4.261233	0.56873	12.49321	0.032548	670.5922
3.266714	1335.546	0.443252	0.607114	2.223917	0.65693	23.97133	0.021502	18.11475
4.25816	149535.3	0.438126	0.594134	1.36429	0.682159	161.236	0.003016	1544.754
3.460526	1036.601	0.46529	0.616352	1.917065	0.687053	10.54841	0.06302	12.44615
3.368634	1557.822	0.37724	0.501929	3.789815	0.627385	9.442934	0.054534	54.17919
3.722402	1268.559	0.53422	0.671682	1.49142	0.738296	29.26912	0.034692	14.34653
4.043024	8857.708	0.461996	0.592537	2.265633	0.686986	37.58232	0.016101	92.43701
4.396279	58163.84	0.407396	0.555237	2.304582	0.644576	43.90276	0.011529	491.0854
4.008936	4744.252	0.529692	0.675312	1.256258	0.734207	29.17878	0.026202	31.6
4.105087	22739.68	0.375022	0.523233	3.965356	0.604006	27.41078	0.015493	163.6307
3.777429	9603.509	0.357886	0.506248	3.288311	0.599876	15.41992	0.02743	142.9
3.620435	1729.808	0.439184	0.594876	2.29538	0.661357	16.39356	0.034267	24.88136
3.817776	2387.036	0.406963	0.558256	2.8627	0.631552	35.36893	0.013753	25.91935
4.057107	15858.03	0.46671	0.607907	2.579101	0.68148	1357.679	0.000429	73.76863
4.005709	6932.58	0.403359	0.558264	2.552366	0.637723	15.91864	0.03621	76.86921
4.195187	11630.03	0.405798	0.553942	2.859746	0.637175	40.80736	0.0125	81.48311
3.845653	15242.19	0.401689	0.53091	3.281161	0.638054	59.98678	0.007613	207.0891
3.381044	1166.564	0.438248	0.580946	2.776062	0.667329	11.73624	0.056969	23.21154
3.701216	2546.277	0.462709	0.618969	1.568413	0.691864	20.55224	0.031505	32.03911
3.837965	3997.276	0.400206	0.551439	2.847963	0.632671	59.78882	0.00758	45.2847
4.244254	20756.96	0.411022	0.550612	2.908799	0.651051	35.36937	0.014545	213.1288
4.03525	7589.225	0.404506	0.535407	3.530529	0.641204	53.49521	0.011719	97.67049
3.900455	5188.39	0.447089	0.59834	2.321835	0.674425	38.20416	0.013934	48.30556

3.567938	1731.543	0.500385	0.64218	1.333495	0.718087	24.70889	0.027686	24.48936
3.688177	1367.047	0.451094	0.610021	2.0131	0.677079	13.78514	0.049914	16.63441
3.864395	4371.527	0.449605	0.586832	2.852605	0.676689	49.0705	0.011381	52.44606
4.249173	88943.38	0.333927	0.480979	4.449406	0.566829	31.62387	0.011239	621.3085
3.668955	2111.831	0.507243	0.651486	1.881101	0.711059	18.49142	0.037263	22.69231
3.662892	6273.168	0.40037	0.531083	3.986759	0.640593	21.70969	0.023973	95.69398
3.805643	15982.14	0.372409	0.51756	3.145704	0.616353	21.39667	0.020362	229.4254
3.981471	13531.64	0.407555	0.558314	2.310264	0.649634	19.15851	0.027955	160.7965
3.415449	1392.212	0.402635	0.562778	2.028472	0.639302	18.43814	0.027765	37.30216
3.528542	1131.157	0.557259	0.69077	1.089007	0.756025	46.42245	0.014965	18.28302
3.600483	757.2926	0.471233	0.619499	1.945394	0.694787	10.99767	0.069717	11.25397
3.892417	17444.27	0.429505	0.581463	1.828961	0.667756	29.53118	0.017608	218.825
3.878403	65114.66	0.374721	0.525544	2.460566	0.618738	37.49929	0.011073	816.5107
4.516193	34675.93	0.456575	0.597021	2.630087	0.676944	35.67309	0.017655	147.8396
4.239397	39836.45	0.354186	0.480285	4.649364	0.602055	23.81905	0.01795	528.4919
3.997528	22662.87	0.334852	0.480733	3.945483	0.573703	1117.502	0.000446	292.5235
3.55199	675.3379	0.492902	0.634936	2.434233	0.708951	30.7123	0.02433	8.59322
3.851879	5288.33	0.441373	0.595391	2.042991	0.671742	34.96799	0.014955	52.96979
4.165634	55661.43	0.322363	0.458067	6.531985	0.550961	24.85204	0.013578	401.097
3.839888	4967.727	0.475398	0.627926	1.503518	0.698467	36.09465	0.016246	52.88539
3.876158	15746.07	0.39555	0.547256	2.70591	0.635551	29.10936	0.015633	168.4083
3.742839	4390.488	0.417279	0.557823	3.399448	0.649869	15.00381	0.036874	51.91935
4.09951	30696.26	0.549996	0.700912	0.687066	0.767707	67.36924	0.010416	267.6385
4.131647	12112.41	0.436131	0.569509	3.22565	0.664599	18.62275	0.037539	129.1992
3.731205	8441.717	0.465896	0.620387	1.173279	0.705119	40.81944	0.014461	130.33
3.942002	21114.68	0.379275	0.527416	2.629287	0.619706	29.31469	0.014845	205.683
3.881187	53374.83	0.34545	0.479685	3.953671	0.587863	15.71707	0.025777	707.5302
3.616837	7590.586	0.457776	0.611255	1.35219	0.695772	27.54701	0.020892	160.0138
3.695118	23926.58	0.420502	0.576763	1.511831	0.665936	29.16094	0.017318	442.5463
3.984752	6234.907	0.453069	0.603694	1.811112	0.684067	20.20109	0.033249	76.43545
3.475903	872.1766	0.521781	0.665582	1.245052	0.731427	31.29636	0.024191	13.24176
3.942616	11876.97	0.44572	0.585613	2.331669	0.678199	25.73047	0.02315	145.1474
3.914805	22927.81	0.4044	0.539674	2.765721	0.646882	26.98483	0.017825	243.7207
4.143583	21207.62	0.403568	0.54129	4.653111	0.620319	19.23359	0.027103	102.266
3.943341	5631.348	0.462095	0.609248	2.305178	0.67899	22.37639	0.028425	41.91954
4.247101	30761.92	0.498872	0.634332	2.555421	0.691349	34.82334	0.017463	89.17884
3.673057	1956.329	0.46143	0.618317	1.276187	0.696024	32.47548	0.018673	29.26519
3.807689	8052.451	0.319033	0.456887	5.384318	0.561338	7.378563	0.056707	143.7833
3.053264	862.4692	0.52993	0.678764	1.381724	0.724647	10.62845	0.063402	10.39535
3.609518	1790.829	0.541255	0.677358	1.977914	0.726375	18.56559	0.038516	15.47423
3.972868	4973.074	0.488617	0.626004	2.038708	0.700618	25.09676	0.026744	40.44876
3.725999	10543.85	0.434354	0.580669	2.950771	0.64685	18.52992	0.027028	76.09626
3.867214	2457.645	0.459365	0.603508	3.868649	0.660745	20.64928	0.027314	15.85417
4.026949	18175.91	0.418711	0.558263	3.01045	0.648558	31.38107	0.016164	196.9126
3.789438	4311.811	0.448059	0.596503	1.91181	0.683689	31.80008	0.017743	63.12469
3.797063	3489.575	0.450422	0.599176	2.720337	0.669963	23.97113	0.023915	36.03061
3.584859	4674.157	0.479283	0.618392	1.625678	0.707476	48.27531	0.012121	59.92468
3.823612	5316.996	0.37562	0.493073	4.280596	0.620871	35.82294	0.013314	117.2835
3.537036	1708.268	0.398707	0.54891	3.127269	0.631258	21.25369	0.022288	27.87603
3.753946	3714.663	0.449593	0.596818	1.923171	0.679855	42.63482	0.012847	48.78571
3.992857	22942.27	0.387733	0.52907	3.619622	0.617683	18.51997	0.025838	245.2367
4.07806	51443.17	0.379417	0.525654	3.722095	0.60648	16.76566	0.027394	367.6491
4.04267	23334.71	0.404084	0.548195	2.730683	0.643982	25.79597	0.019388	239.5391
3.580246	2678.723	0.3664	0.503807	4.515373	0.60532	20.06489	0.022029	65.70996
3.965288	5264.174	0.3941	0.540185	4.681817	0.614544	1195.055	0.00713	41.90083
4.012638	25282.6	0.356141	0.497349	3.255203	0.604197	52.94574	0.007293	265.1654
3.609022	5162.684	0.440229	0.588386	2.353206	0.669781	21.08485	0.027121	83.5097
3.562876	13353.11	0.378764	0.522093	3.287486	0.613188	26.40689	0.015465	299.2157

3.763605	10104.2	0.480114	0.638399	0.996348	0.716279	34.10355	0.017527	138.3464
3.91281	4723.269	0.498671	0.643706	2.133193	0.701193	20.96595	0.032174	28.44076
3.884218	29129.34	0.405656	0.536686	2.833647	0.650812	36.8617	0.013011	455.8715
3.40583	2667.187	0.379185	0.527579	3.530692	0.603011	8.144841	0.056245	52.60274
3.433426	5124.447	0.51509	0.663146	1.104149	0.733701	38.61799	0.016151	82.79431
4.28495	30789.45	0.379855	0.52114	5.038814	0.599243	21.22615	0.021784	167.511
3.53886	3038.85	0.536155	0.671017	1.818443	0.721821	14.37997	0.052725	31.752
3.614984	6659.458	0.352738	0.491517	3.585761	0.60057	17.97055	0.023326	139.2589
3.285194	1262.68	0.404922	0.557842	2.424794	0.642562	13.0349	0.037693	31.33333
3.663881	5079.946	0.404597	0.553325	3.101169	0.630206	36.8858	0.012031	61.14533
4.058187	12486.12	0.445743	0.584614	3.191733	0.657337	20.78619	0.028095	68.73723
3.747911	4429.294	0.482339	0.62797	1.574352	0.709445	27.44847	0.025051	70.6272
4.28262	14085.27	0.395408	0.540775	4.188852	0.623873	39.56363	0.012344	85.64834
4.107394	18913.36	0.374076	0.512221	5.786988	0.601262	26.88474	0.016154	146.7243
3.943725	15598.25	0.402592	0.520593	3.37365	0.649099	28.52729	0.018157	265.9203
4.135929	6844.035	0.45941	0.593658	2.573177	0.68247	30.0465	0.02172	55.54595
3.784586	12809.58	0.43495	0.589627	1.612275	0.675364	27.91511	0.019418	180.1149
4.327118	53361.71	0.3371	0.468325	5.469992	0.580753	17.16866	0.024665	458.3821
3.545823	1920.659	0.467918	0.611344	2.863854	0.67536	19.92506	0.030647	20.14286
3.767434	4635.203	0.432716	0.569082	4.365798	0.650762	26.37754	0.019448	41.49558
3.780873	3575.856	0.524565	0.669277	1.660082	0.719758	13.67653	0.065361	21.44286
3.871508	8789.026	0.433252	0.573335	2.684563	0.664702	61.09966	0.008037	110.4958
3.895982	5092.923	0.423048	0.563101	3.565358	0.641822	50.02518	0.009378	37.16535
3.255894	1492.877	0.42377	0.587434	1.548185	0.663979	35.813	0.015531	39.94479
4.110952	38666.97	0.414587	0.558522	3.467295	0.634021	56.02937	0.007753	198.3834
4.152844	6770.994	0.426249	0.572417	3.787326	0.648614	18.65046	0.037809	51.61111
3.864149	5424.525	0.511931	0.657691	1.49594	0.724931	21.91526	0.03306	59.29491
3.580149	7408.592	0.357377	0.495336	3.181456	0.60555	26.7363	0.015236	173.0611
3.441522	913.4788	0.512037	0.657587	1.807908	0.72169	19.13673	0.036733	12.07463
3.592965	4079.744	0.575218	0.710698	0.888551	0.774045	51.0772	0.013829	52.84367
3.101909	343.5991	0.571847	0.731359	0.708638	0.768434	18.82441	0.041768	8.3
3.719113	2369.316	0.4861	0.629029	1.988321	0.699688	32.76444	0.019515	32.45455
4.061923	12129.65	0.414601	0.565378	2.753117	0.648536	32.33439	0.015718	110.5252
3.678451	14744.8	0.43643	0.579513	1.966803	0.67449	34.6296	0.01525	277.0624
3.228977	1766.903	0.52697	0.676247	0.964006	0.745135	23.86874	0.027991	47.51366
2.999518	458.4843	0.461755	0.627972	2.144122	0.675107	7.801135	0.078183	9.5
4.316752	4324.593	0.569481	0.694	1.858018	0.747999	40.48364	0.024308	16.8882
3.81196	6920.897	0.434363	0.577757	2.697331	0.660784	22.19962	0.024858	69.608
3.727502	6699.152	0.366956	0.507892	5.131341	0.607426	57.64883	0.006812	92.50754
3.900657	12110.26	0.490794	0.630942	1.641965	0.708302	36.17053	0.019026	114.1808
3.682889	3587.257	0.415636	0.552235	3.193869	0.651727	26.41218	0.020252	62.61481
3.598903	1037.932	0.55146	0.701605	0.926109	0.759474	34.35222	0.02773	18.776
3.467275	3982.99	0.398945	0.536047	2.608779	0.647205	17.391	0.030225	139.3859
3.701478	648.4722	0.652708	0.762313	0.893777	0.812065	26.27447	0.05025	7.360656
3.785086	2119.363	0.369231	0.476746	6.61441	0.614128	29.24409	0.01494	34.47945
4.129195	6616.683	0.4947	0.630162	2.358098	0.709318	55.81881	0.011732	50.86111
3.556882	2997.528	0.552387	0.695088	0.824145	0.766392	43.16053	0.017661	57.94536
4.050104	11135.2	0.371418	0.497025	5.565414	0.612889	25.67403	0.017685	140.9281
3.553871	2428.88	0.493854	0.621875	2.812559	0.706389	24.37547	0.025659	29.58278
3.933427	15444.54	0.373125	0.505157	3.858266	0.614402	91.62219	0.004322	220.0326
3.56546	4782.277	0.536062	0.682551	1.097449	0.745206	25.99823	0.028616	71.88101
3.799909	3123.957	0.52125	0.665764	1.212878	0.737166	22.85898	0.03433	35.34426
3.155107	497.1612	0.614287	0.737422	1.45197	0.786376	12.42375	0.076675	11.63636
3.277876	5697.454	0.522092	0.668662	0.964291	0.743005	37.38691	0.017718	125.5612
3.472873	6375.385	0.515575	0.664771	1.047349	0.73735	47.95939	0.012808	141.8824
2.967908	1162.043	0.567066	0.703806	1.052386	0.766551	15.65461	0.045314	31.13793
4.100524	28940.01	0.395467	0.538059	3.850917	0.627717	29.95204	0.014994	213.1085
3.623011	2957.033	0.530886	0.672784	1.11899	0.746361	37.30243	0.01866	49.77814

3.754982	11950.41	0.475737	0.621163	1.909465	0.701721	28.95729	0.020827	144.777
3.851827	5563.41	0.451901	0.599266	2.269705	0.67466	32.0638	0.017313	45.82734
3.324012	2349.585	0.553174	0.700808	0.800839	0.765713	24.40128	0.03176	49.03861
3.623809	2502.761	0.439292	0.576378	3.514717	0.672471	23.04254	0.026817	33.54839
3.554055	11366.38	0.488461	0.635747	1.234893	0.718334	48.14381	0.012338	284.2138
3.518984	2289.374	0.498489	0.649859	1.140381	0.725587	25.28054	0.028646	43.91139
3.996828	15132.47	0.449886	0.582876	3.01617	0.669956	43.90712	0.011937	100.3888
3.916208	4799.319	0.541061	0.674969	1.142486	0.752934	43.23469	0.018688	67.55717
3.886668	124008.4	0.424515	0.561628	2.02583	0.670029	52.37327	0.010003	2129.351
3.708726	5632.419	0.491199	0.622433	2.196173	0.716875	42.24505	0.015657	80.1119
3.577729	2187.526	0.555265	0.699132	0.998753	0.760477	36.72534	0.023246	31.47236
4.053456	41945.94	0.318601	0.388155	11.75653	0.568407	62.51351	0.005452	996.369
3.978204	13179.51	0.422301	0.564587	3.367316	0.651188	25.79838	0.020594	122.8155
3.977967	21659.72	0.454283	0.599385	1.824274	0.689155	39.57986	0.014028	244.3899
3.04859	706.6447	0.672346	0.761958	1.359712	0.813672	21.10867	0.040641	11
3.510201	4331.813	0.470127	0.615264	1.966276	0.696134	28.33544	0.020362	98.10286
3.862381	16221.8	0.418305	0.562504	2.368299	0.6576	26.85496	0.020379	227.3981
3.549949	10227.78	0.517971	0.661587	1.090395	0.740414	25.04488	0.028229	192.0797
3.442364	1935.243	0.487631	0.630022	2.698474	0.703075	25.36981	0.031495	32.19697
3.481179	1996.33	0.459719	0.612817	2.191899	0.683292	18.96317	0.030402	28.82482
4.42887	15208.59	0.408946	0.545137	3.521471	0.635734	80.97185	0.005413	79.36778
3.09454	313.9465	0.548524	0.708035	1.040123	0.751411	20.12632	0.0536	7.380952
3.737119	17854.45	0.369034	0.510355	3.552968	0.614016	15.15151	0.02988	305.3965
3.858377	9844.832	0.458584	0.597515	2.305646	0.683889	20.10942	0.029733	94.41556
3.709326	362.4769	0.724302	0.821177	0.490739	0.857442	31.57048	0.051251	4.952381
3.875723	6713.19	0.482255	0.635988	1.521694	0.707187	33.3414	0.019272	78.91984
4.034121	19489.02	0.392265	0.541703	3.69059	0.619589	32.46124	0.013394	145.1366
3.650319	7034.806	0.455737	0.607653	1.884575	0.68235	15.21962	0.039501	72.45024
3.556262	7533.217	0.505241	0.646834	1.231289	0.728609	44.89912	0.014312	134.1995
3.726349	762.5944	0.58676	0.718819	1.237509	0.776085	27.37313	0.038833	10.0625
3.681755	4566.669	0.503061	0.653147	1.063032	0.732072	43.99787	0.015493	90.11424
4.037791	4997.655	0.490025	0.613468	2.580905	0.709687	26.3736	0.032028	49.89189
3.77282	4081.309	0.577097	0.718603	0.765173	0.779311	44.85991	0.018335	51.13759
4.373793	5018.047	0.551525	0.668975	1.535172	0.762353	45.70582	0.022821	82.4893
3.129162	192.1837	0.737038	0.813299	0.765759	0.855892	38.20311	0.035358	3.857143
3.283437	2659.806	0.364703	0.531465	3.484261	0.590038	4.920228	0.093404	42.59633
3.139508	355.2812	0.530678	0.67496	1.361618	0.730262	11.87555	0.060907	5.272727
3.699165	6647.498	0.315275	0.451693	5.185903	0.552888	11.94418	0.029389	121.1967
3.990884	26313.99	0.369358	0.499236	4.175284	0.606082	27.47605	0.015179	302.3521
3.673206	2407.435	0.328763	0.443591	5.502611	0.581618	11.94064	0.033642	87.00662
4.171472	9610.986	0.351972	0.488487	4.439104	0.590238	16.82057	0.025975	114.119
3.109501	1688.583	0.379662	0.547954	1.822664	0.616741	8.03047	0.055332	51.01653
3.648247	1680.181	0.431854	0.578179	2.04408	0.672149	28.47713	0.019432	36.21739
3.683192	1716.072	0.328151	0.418201	9.479831	0.575957	1080.694	0.001404	55
3.983813	6154.282	0.387381	0.509204	3.70817	0.635303	48.64813	0.010754	113.3079
3.664866	37443.79	0.406693	0.558496	1.870754	0.652427	37.31315	0.012388	668.7513
4.121337	13397.62	0.379776	0.513786	7.216134	0.593471	23.80257	0.017416	63.92183
3.61203	1802.826	0.463915	0.617649	1.827063	0.692018	27.3086	0.023342	25.3375
3.362201	1378.678	0.349122	0.461028	5.566209	0.600692	7.784041	0.057525	43.69231
3.231585	1111.6	0.472126	0.618103	1.886457	0.697525	15.01948	0.040648	25.16495
3.959783	21309.89	0.37177	0.484647	4.623289	0.619946	140.2177	0.002893	358.0564
2.890292	606.0612	0.571639	0.710085	1.652402	0.759741	7.484887	0.11513	11.26667
3.703359	17497.03	0.435707	0.577723	1.84766	0.6772	46.42807	0.011891	348.3446
2.857281	468.6211	0.507785	0.647813	2.884068	0.687648	9.975098	0.056053	6.111111
3.64166	2383.768	0.551903	0.695358	1.117738	0.75188	40.12365	0.019795	32.0991
4.284001	4358.635	0.544575	0.652508	2.859795	0.744042	38.6077	0.027675	25.61277

4.56744	6359.024	0.311189	0.350655	13.11968	0.574653	456.8523	0.000812	216.6203
4.46567	54357.04	0.426959	0.556453	3.058149	0.662233	57.36228	0.009753	432.6399
4.212395	22767.55	0.298835	0.3812	10.26072	0.553748	582.4688	0.000552	676.5038
3.749454	15640.77	0.351941	0.495139	3.316524	0.599813	41.35696	0.014743	258.9013
3.725026	5833.364	0.329432	0.416727	7.786944	0.584415	22.56925	0.016742	212.6698
3.329497	2212.007	0.389	0.525957	3.819504	0.63214	6.031778	0.102433	65.89498
3.668045	2171.913	0.343404	0.47767	5.332199	0.586632	19.42857	0.020289	50.24623
3.590386	6586.884	0.330254	0.472956	3.864349	0.573149	13.57712	0.027533	112.6667
3.340777	1810.808	0.493566	0.617196	4.253377	0.690269	8.507231	0.081106	23
3.335698	1333.09	0.401364	0.550166	2.327453	0.645881	8.681604	0.061909	48.52857
3.9015	3236.137	0.390022	0.542797	4.836573	0.613895	31.24923	0.014766	29.41885
3.674592	5592.874	0.363223	0.48486	6.577163	0.595388	14.53229	0.02879	89.55172
3.594436	2006.493	0.579306	0.682708	6.35237	0.722446	15.39765	0.049027	6.853659
3.415964	1472.755	0.421956	0.545685	3.043867	0.659291	21.85506	0.022881	40.41935
3.652671	5741.328	0.332339	0.452101	6.846601	0.570259	15.0152	0.02503	124.2368
3.219345	755.9152	0.441553	0.586987	2.886295	0.665392	7.004739	0.090529	17.82759
3.8019	1366.093	0.302438	0.389963	13.29908	0.550356	6.173951	0.074568	43.30065
4.255742	21583.6	0.362777	0.490236	4.512968	0.608844	36.73085	0.011596	260.2553
3.836434	12085.93	0.388926	0.503261	4.200389	0.627146	21.42414	0.024252	154.3652
3.711426	5903.13	0.384389	0.516948	3.400248	0.630653	31.43029	0.014914	119.5237
3.690934	5281.363	0.375946	0.463844	5.136789	0.630122	30.7495	0.01441	181.5007
4.349008	40738.49	0.471978	0.609439	1.82817	0.700657	57.44914	0.010752	280.4418
3.756825	5551.719	0.316406	0.374557	11.24733	0.574406	14.78276	0.026734	404.2609
3.025366	172.2865	0.775925	0.849622	0.486998	0.875057	29.67544	0.048266	2.25
3.211881	2410.878	0.493084	0.651979	1.131016	0.717408	28.37156	0.022041	69.89157
3.779391	14263.06	0.365145	0.502141	3.594769	0.609172	31.0487	0.013883	242.6483
3.895771	16884.23	0.359707	0.505938	3.075984	0.603634	49.95456	0.007958	213.7809
4.299137	9968.574	0.521331	0.62638	2.103382	0.735083	51.25009	0.021398	97.20436
3.729593	491.9909	0.504388	0.61971	2.678297	0.729441	31.47284	0.026864	11.75
3.989085	552.6218	0.616119	0.747272	0.933195	0.798153	42.17692	0.060251	9.886076
3.451862	4142.111	0.335738	0.477852	4.426724	0.570702	23.66426	0.015678	52.7277
3.799871	1535.743	0.462544	0.596239	2.492081	0.688457	54.96325	0.015402	22.15646

VX88	VX89	VX90	VX91	VX92	VX93	VX94	VX95	VX96
0.185469	4.110316	34.82266	435738.5	12915458	15290.25	0.058912	65.45813	0.161227
0.193802	3.678397	16.95763	1670171	14417723	266027.8	0.311104	28.20339	0.119506
0.331585	2.668259	95.91603	1408156	1.37E+08	14608.96	0.014497	84.19084	0.214226
0.322917	1.567708	8.458333	15877.58	141747.7	1817.422	0.353588	3.833333	0.159722
0.209326	3.18862	20.84404	60585.16	1620439	2410.913	0.107663	38.06422	0.349213
0.209515	4.104757	23.57971	459185.4	9675520	23107.44	0.110526	53.23188	0.192869
0.188628	4.644527	49.79149	63580.66	3640121	1133.577	0.042233	62.65106	0.2666
0.162426	4.306185	33.44048	168775.3	6997560	4224.554	0.062228	105.6381	0.251519
0.260331	1.845041	10.04545	7577.273	81435.27	769.9056	0.267374	3.727273	0.169421
0.175549	4.47742	82.58378	438781.6	40961199	4760.35	0.01679	245.3501	0.261846
0.174947	3.575391	51.89608	3033775	1.77E+08	53764.81	0.027074	677.5334	0.246197
0.200213	2.660146	32.64595	20488815	6E+08	731070.3	0.045753	584.5442	0.178106
0.158581	4.233021	38.26517	469011.7	21103942	10796.31	0.052024	84.22697	0.189274
0.153007	4.045225	39.86364	39373.68	1841168	892.6926	0.047393	27.66667	0.209596
0.175737	4.201814	24.2381	29579.62	1190215	754.0063	0.139889	10.28571	0.244898
0.197257	3.24861	57.5966	2419748	1.62E+08	36739.73	0.02317	326.4966	0.222107
0.242435	2.744749	20.13208	41864.96	883271.7	2230.038	0.116195	7.981132	0.150587
0.159857	3.46554	30.73077	532131.1	14262240	22001.99	0.058416	143.5495	0.262911
0.107981	7.31191	71.42288	186449.6	16262238	2229.844	0.02649	231.4853	0.200594
0.179605	3.893769	38.89951	1817824	87771371	42768.04	0.045177	446.9513	0.272199
0.170328	3.517511	36.48389	3195027	1.11E+08	105526.7	0.046783	385.2119	0.238669
0.161121	4.714369	40.89218	339976	15665107	8596.056	0.044829	248.6004	0.262791
0.176276	3.722117	30.04348	63618.15	2114935	1973.532	0.067075	19.34783	0.210302
0.173351	9.990444	1958.389	5100885	9.89E+09	2635.081	0.002428	123.9028	0.158443
0.179674	4.230378	44.39799	961692.4	52609090	18014.8	0.041719	114.6856	0.191782
0.278119	2.420959	15.05435	71446.57	1091844	4844.757	0.180881	22.78261	0.247637
0.380832	39.98858	1635.817	1149699	1.94E+09	682.0928	0.012207	40.18261	0.174707
0.160115	3.9078	45.16817	477107.3	22421985	11106.54	0.037029	59.63664	0.179089
0.187535	3.328179	31.71795	172233.5	7150143	4271.642	0.060891	51.42051	0.263695
0.158562	8.421562	1884.99	173375.7	3.41E+08	101.4135	0.00315	94.64921	0.247773
0.16078	5.023103	36.10619	33935.95	1609312	727.8644	0.081527	21.19469	0.187564
0.173869	3.023502	25.97761	1332384	34154393	55959.15	0.073351	166.5547	0.207158
0.239607	3.350466	26.63492	64076.79	1799199	2350.068	0.087281	35.66667	0.283069
0.16834	3.747315	48.05693	1113071	51236634	25973.06	0.034218	92.35104	0.175239
0.187578	2.391288	25.64392	10687709	2.26E+08	556428.7	0.063888	936.1748	0.261867
0.296963	2.79226	34.40984	128288.4	4863427	3444.646	0.06256	12.86885	0.210965
0.139054	5.888619	230.7239	4897072	1.12E+09	21960.78	0.004886	3522.891	0.31712
0.191479	3.196686	17.75385	55551.12	736605.6	4612.112	0.177775	13.86154	0.213254
0.313175	1.695012	13.88439	178149.9	2824925	11323.72	0.15461	24.87283	0.143774
0.142045	4.528968	27.68317	25390.73	1220566	542.8785	0.114194	12.08911	0.119694
0.143759	4.669698	44.24417	314657.7	18816353	5389.945	0.044071	124.7916	0.194077
0.139791	4.913309	61.87219	3206418	2.41E+08	47188.36	0.025337	848.8093	0.241619
0.143636	6.36	33.4	107599.6	4268755	3210.713	0.081318	37.76364	0.171653
0.163304	3.335741	44.75948	3000263	1.36E+08	68755.46	0.03513	180.8922	0.180531
0.216515	2.270549	25.38333	1275946	32980603	51655.43	0.064061	168.1303	0.254743
0.210859	2.737432	21.27119	94795.06	2341353	4023.364	0.101109	20	0.169492
0.209027	2.935679	46.34677	227284.9	13771968	3814.85	0.037949	16.96774	0.136837
0.144644	8.130657	1984.718	927382	1.88E+09	459.3109	0.002467	92.92549	0.182207
0.209453	2.979694	28.80381	575931.4	15272271	24128.42	0.078072	86.23161	0.234964
0.13764	5.031521	56.78885	848234.8	56848690	13133.75	0.037921	121.5101	0.205254
0.177454	4.057037	79.6144	1284349	1.27E+08	13104.75	0.018444	221.6272	0.189912
0.223188	2.769138	14.95192	65088.96	1383836	3227.298	0.19852	26.17308	0.251664
0.178989	3.319809	24.05028	102592.9	3335791	3364.661	0.093562	32.34078	0.180675
0.161156	4.596193	79.87189	308759.6	31065591	3103.52	0.021445	60.00356	0.213536
0.133289	9.69895	57.81864	944514.8	55861773	17088.05	0.034413	385.4478	0.241056
0.139929	7.402897	66.17335	512747.8	49502678	5353.184	0.043114	141.467	0.202675
0.167728	3.713542	50.97917	283656.5	17756687	4711.755	0.030602	64.45139	0.22379

0.173683	3.667824	30.10638	52821.56	1946990	1480.173	0.074652	32.56028	0.230924
0.178865	3.988207	19.73118	59480.54	1125680	3459.077	0.183961	19.36559	0.208232
0.152904	5.025814	60.16618	187747.8	14972545	2378.653	0.03128	65.69971	0.191544
0.173018	3.669184	58.1682	19118084	1.03E+09	368263.2	0.023765	685.479	0.190888
0.193951	3.190007	26.67521	62798.09	1600412	2722.581	0.076773	19.75214	0.168822
0.230588	2.493622	29.23373	725616.5	26186226	20388.97	0.063671	87.24578	0.210231
0.22383	2.486069	30.94439	1987352	70771563	57710.48	0.050227	295.281	0.288079
0.188066	3.265147	29.19766	867458.1	26753766	31976.96	0.063923	253.2901	0.296246
0.268361	3.118265	24.2518	97243.5	2957158	3306.424	0.100594	36.13669	0.259976
0.172481	5.075561	51.08491	17867.93	1200692	273.8823	0.040109	28.07547	0.264863
0.178634	3.475939	19.34921	26452.03	423229.7	1887.445	0.189622	18.42857	0.292517
0.174085	3.935594	41.34288	815938.6	34819755	20594.4	0.038238	330.6643	0.263058
0.200568	3.141633	60.45222	7485593	4.45E+08	128658.5	0.021508	1005.04	0.246878
0.120982	6.032283	56.95254	1493100	59318725	50372.06	0.031784	277.1047	0.226763
0.181925	2.833565	36.16282	4106059	1.52E+08	119390.3	0.040042	799.8985	0.275352
0.217813	9.326634	1919.075	4175836	8.18E+09	2133.419	0.003685	286.2107	0.213113
0.145648	5.146222	39.33898	21178.05	990542.6	462.0286	0.089151	13.91525	0.235852
0.16003	4.570166	45.39879	262179.5	13201455	5436.502	0.038093	80.38369	0.242851
0.188929	2.73999	46.78756	17542692	7.36E+08	430534.6	0.029148	328.089	0.15454
0.151534	4.621177	50.07736	151460.4	7867375	3088.169	0.037273	73.01146	0.209202
0.170626	4.246407	49.20567	1358530	59086069	32486.18	0.03121	242.5198	0.245714
0.209352	2.655242	20.71774	384357.5	9837860	15904.21	0.089837	68.66129	0.27686
0.105494	8.122921	91.31573	335528.4	27058475	4505.056	0.017719	814.1549	0.320912
0.176261	3.270501	24.5839	642099.1	21639234	22250.23	0.096015	165.1091	0.225251
0.160505	5.895671	50.99384	284970.2	17280153	4816.129	0.037724	234.2906	0.288535
0.179636	3.476117	45.84541	2640397	1.32E+08	54380.17	0.031532	283.5555	0.247647
0.221172	2.45768	26.02219	10870767	2.76E+08	441991	0.061116	710.5267	0.222109
0.220405	3.424463	36.28926	277019.9	11736796	6782.633	0.045485	229.9311	0.31671
0.217789	4.142278	43.71703	1446216	62178887	34503.08	0.03761	502.9222	0.247501
0.167255	4.176865	23.92779	200841.9	6641351	6779.088	0.110138	93.96718	0.205617
0.145514	5.587489	40.67033	17809.97	756212.5	433.6245	0.080487	24.03297	0.264099
0.172589	4.282306	31.55529	542429.7	23722410	12958.4	0.066043	194.2533	0.230979
0.19011	4.168434	40.5156	2072669	69803796	66354.11	0.041451	418.6381	0.326551
0.121456	12.21157	56.05107	2315246	67191728	84844.1	0.052422	132.1568	0.156956
0.160611	4.413734	31.60536	293847.2	10800923	8717.325	0.075438	55.14559	0.211286
0.132904	5.754971	47.80179	1640731	71866534	42639.08	0.039183	82.47541	0.122914
0.161686	5.23586	39.20994	72558.12	3691472	1464.661	0.056909	43.37569	0.239645
0.273352	2.164846	13.52662	2048023	23302870	194870.7	0.17388	117.4106	0.223214
0.241752	2.276906	18.65116	38901.88	553951.4	2959.417	0.119535	7.790698	0.181179
0.159528	3.865023	28.5567	44759.32	968309	2274.157	0.078811	15.63918	0.161229
0.142928	4.46713	34.23322	155062.3	5608312	4867.887	0.06439	53	0.187279
0.203466	3.282965	28.49733	1102796	30631994	41467.31	0.064039	58.2139	0.155652
0.165148	3.852431	30.97917	167340.7	4368586	6726.906	0.068852	11.8125	0.123047
0.183004	3.900785	44.32249	1298671	69025195	25211.25	0.035116	201.7565	0.187506
0.157418	3.894864	39.65586	155882.4	7455968	3358.263	0.045832	99.79302	0.24886
0.18383	4.342644	33.53571	193934.4	7800365	5044.618	0.069082	36.80612	0.187786
0.155649	4.979052	69.18701	183513.6	12071039	2831.415	0.026393	88.65714	0.230278
0.230873	3.387636	56.69488	634162.9	39818987	10164.12	0.027327	117.3268	0.230958
0.23038	3.337477	36.90083	176767.9	6098771	5219.792	0.05459	22.61983	0.186941
0.145196	4.796547	51.04464	151461.8	9762492	2389.331	0.038681	74.11905	0.220592
0.200193	3.036611	31.13959	2389043	77602531	77351.2	0.0612	196.151	0.160123
0.178905	3.242577	30.40925	6929642	1.82E+08	284732.6	0.05893	307.5664	0.149667
0.164293	4.523212	38.2476	1660365	70152171	41806.56	0.048559	378.0137	0.259269
0.284459	2.608122	31.56277	365191.6	12922458	10404.79	0.05747	34.43723	0.149079
0.173144	61.76212	1869.417	617911.6	1.23E+09	329.6274	0.024265	35.20661	0.145482
0.178322	3.566465	87.21453	3477959	2.79E+08	44740.77	0.014904	482.4701	0.324459
0.231329	3.425096	31.41828	317195.7	10344558	10024.34	0.062077	68.97784	0.191074
0.325943	2.595735	44.15468	1770456	79835912	40060.98	0.031181	159.5904	0.173846

0.159753	4.976873	50.36028	284722.8	13324189	6390.5	0.03279	267.7621	0.309194
0.13479	4.509962	37.34123	161073	3676465	8026.479	0.058155	31.78673	0.150648
0.173335	6.923745	59.6711	2017305	1.2E+08	34487.13	0.027187	669.2882	0.254482
0.360293	2.138488	15.61644	433755.4	5200116	39203.49	0.132475	20.46575	0.140176
0.181169	3.545222	53.99562	125303.8	6419243	2512.649	0.030548	111.919	0.244899
0.175404	3.654336	38.55707	4649159	1.7E+08	136685.1	0.050432	124.8723	0.130756
0.254016	2.740736	25.704	107801	2109868	6277.283	0.10274	19.896	0.159168
0.261274	2.828001	29.12008	1064927	33659962	34804.37	0.063384	147.4916	0.27672
0.326389	2.218641	18.30208	123979	2332144	6884.507	0.09887	22.70833	0.236545
0.211576	3.930796	52.11073	522726.1	31088666	8927.842	0.03575	46.58478	0.161193
0.167244	3.933945	31.19708	1040239	34093916	33545.14	0.067228	61.14599	0.148774
0.177902	3.780051	31.15113	123935.9	5528097	2860.78	0.065611	92.92947	0.234079
0.123948	7.81851	63.84081	1224184	79881713	19410.57	0.033328	114.1172	0.165148
0.171407	3.5776	42.75701	2716313	1.31E+08	57947.91	0.038309	132.729	0.155057
0.179676	3.572805	38.63041	1181795	56611462	25114.34	0.044966	433.0243	0.292584
0.150124	4.672673	41.94865	315249	16636000	6462.575	0.048134	61.21081	0.165435
0.163	4.272214	40.43529	583836.5	22722289	15688.95	0.047083	360.6561	0.326386
0.168585	3.262435	30.29937	8657405	2.42E+08	341308.8	0.060554	648.2306	0.238408
0.22135	3.08441	30.56044	130276.7	3792581	4642.491	0.064305	13.26374	0.145755
0.183609	3.556817	39.23894	386218.2	15226450	10052.46	0.051824	41.31858	0.182826
0.153163	4.46102	24.2	115315.4	1850799	8861.162	0.191378	18.57143	0.132653
0.15651	5.013316	75.82578	465167.3	45741835	4776.276	0.01976	132.1983	0.18725
0.14632	5.256944	67.94094	435905.2	35435984	5440.132	0.029782	43.87402	0.172732
0.24506	4.726937	47.49693	88870.88	5158259	1557.849	0.049292	54.22699	0.332681
0.153666	4.326833	83.91092	3855895	3.4E+08	45217.68	0.016704	214.6886	0.166296
0.179205	4.072483	31.85417	471017.6	15116528	19100.71	0.10878	53.89583	0.187138
0.158968	3.689971	27.02949	104830.7	3139812	4266.881	0.077108	104.0188	0.278871
0.251908	2.954906	40.68705	1042432	48116821	22962.63	0.037895	200.2897	0.291542
0.180218	4.341279	29.79104	27091.31	728722.3	1058.643	0.095728	11.80597	0.176209
0.142436	5.284733	61.79784	47835.5	3370327	693.8635	0.02819	89.79245	0.242028
0.2075	2.9275	20.15	4063.9	109748.7	158.8664	0.112689	10.25	0.25625
0.184401	4.272598	43.47727	78559.25	4290624	1480.995	0.045464	32.23864	0.183174
0.163984	4.043526	45.75519	803630.7	41348784	16457.09	0.036333	141.2433	0.20956
0.22165	3.729299	49.308	784869	43122574	14609.9	0.032732	289.6256	0.2317
0.259637	3.394846	30.62842	40396.02	1291300	1305.301	0.062744	42.6612	0.233121
0.296875	1.683594	11.0625	24531.81	252429.4	2517.342	0.201944	5.75	0.179688
0.104896	7.822306	46.47826	69813.42	4097203	1408.191	0.082252	31.78261	0.197408
0.185621	3.401913	33.75467	472290.2	15249997	15251.65	0.057825	77.53333	0.206756
0.232431	3.048963	87.52261	1024212	98031846	10797.14	0.016441	82.69849	0.207785
0.14848	5.127288	42.37061	414166.3	24455328	7203.657	0.050421	138.4512	0.180041
0.231907	3.585789	37.57037	290191.5	13442590	6369.981	0.063463	58.37037	0.216187
0.150208	5.632	46.592	12347.94	593281.4	267.753	0.072191	42.072	0.336576
0.327967	2.174671	20.71529	322814	8881277	12024.01	0.080949	132.6141	0.312033
0.120666	6.366568	29.7541	3853.148	126365.6	129.8299	0.163704	12.57377	0.206127
0.236161	2.712892	41.56164	356610.8	17782976	7206.325	0.040671	36.63014	0.250891
0.117734	6.403501	66.78935	167398.5	14241651	2030.37	0.031301	87.81944	0.203286
0.158321	5.175013	46.61202	41719.53	2563624	692.587	0.041448	99.31694	0.271358
0.21097	2.888278	39.48353	1396623	63603328	31738.92	0.040894	139.8323	0.20933
0.195912	2.993377	27.99338	105272.3	3986537	2837.605	0.064638	24.78808	0.164159
0.199305	3.863834	136.51	1824893	2.8E+08	11985.72	0.009416	232.6286	0.210714
0.181977	4.053556	30.38734	94246.65	3499605	2654.968	0.070303	91.16203	0.23079
0.144854	4.843187	30.17213	61074.96	1897675	2131.376	0.08646	63.01639	0.258264
0.264463	3.18595	16.40909	5859.318	98989.32	397.3654	0.171288	11.81818	0.268595
0.225829	3.679766	51.23022	150115.5	7650202	3005.988	0.036406	152.1691	0.273685
0.208651	4.004637	59.28824	135926	9369406	2010.304	0.024746	180.0471	0.264775
0.26843	2.107313	21.74138	22038.61	462138	1112.94	0.081545	29.84483	0.257283
0.1806	3.122909	50.23136	2164166	1.04E+08	48320.5	0.028277	270.3237	0.229088
0.160058	4.501484	42.45659	47619.44	2586358	901.7278	0.045322	80.66559	0.259375

0.161401	3.807447	37.45262	398925.7	16999163	9735.982	0.050445	219.1293	0.244291
0.164847	3.838375	47.31295	331532.8	17445664	6464.974	0.03657	46.80576	0.168366
0.189338	3.866013	29.6834	37727.72	1283282	1141.323	0.073214	72.94595	0.281645
0.180368	3.325587	25.70968	163325.2	6047574	4479.861	0.091799	42.6129	0.229102
0.223439	4.276144	63.17453	294528.4	21126529	4167.631	0.025448	308.5739	0.24259
0.18528	4.534993	29.24051	57968.49	2291917	1519.174	0.092044	67.81857	0.286154
0.151874	4.863776	59.65961	1057955	75489371	15184.62	0.027716	99.46293	0.150473
0.140452	5.608136	51.62786	70970.27	4419104	1175.908	0.03707	128.3098	0.266756
0.151093	25.45511	127.9124	5978372	4.32E+08	83947.84	0.015419	4053.436	0.287621
0.142295	5.26996	48.25044	165439.9	10993858	2510.278	0.040965	105.0213	0.186539
0.158153	5.794349	46.07538	34634.55	1894492	650.8996	0.067549	40.32663	0.202646
0.267769	3.431984	110.269	13724356	1.64E+09	115280.2	0.010917	662.7044	0.178098
0.182761	3.517538	36.78571	927379.7	38673317	23439.96	0.052186	123.5744	0.18389
0.147046	4.585257	51.15644	719325.3	38571551	14361.35	0.030389	499.0806	0.300289
0.192982	3.195445	26.98246	6537.263	179266.1	254.5996	0.082363	12.4386	0.218221
0.280294	3.613878	42.76571	184338.7	8177360	4261.287	0.042736	75.97714	0.217078
0.179619	4.098611	37.44787	1031347	43005892	25433.66	0.055567	303.9036	0.24005
0.184515	3.920229	30.8684	199398.5	7083353	5882.378	0.069433	293.3977	0.281842
0.243916	4.223313	32.26515	87404.46	3766231	2090.696	0.09921	26.60606	0.201561
0.2104	2.533859	25.88321	100800.4	2823031	3738.12	0.06626	30.48905	0.222548
0.138998	4.741379	113.9457	1192018	1.57E+08	9473.059	0.012162	104.2049	0.182495
0.175737	3.669501	24.78571	4618.262	141350.3	158.0474	0.133658	8.571429	0.204082
0.24315	2.141648	24.98487	2296058	54752763	99513.97	0.064609	283.8439	0.22599
0.179157	3.145872	29.98102	475270.4	14032486	18084.75	0.059629	128.0361	0.242953
0.117914	6.535714	36.78571	844.0238	37132.12	29.60003	0.104451	10.42857	0.248299
0.158156	4.782511	44.58116	190067.7	8920776	4230.349	0.043506	120.4028	0.241288
0.180294	3.754099	50.18012	2272214	1.11E+08	48389.01	0.030388	170.7168	0.212071
0.171683	3.137199	22.83649	316581.4	6526602	17676.33	0.096943	113.8104	0.269693
0.176115	4.588464	58.09711	193819.6	12979703	2933.782	0.029603	185.147	0.242975
0.157227	6.075928	31.54688	7965.844	330377.5	214.6131	0.116162	14.21875	0.222168
0.158373	5.360182	46.48682	86999.02	5774705	1341.442	0.041299	164.7469	0.289538
0.134843	5.320533	28.38649	143656.7	6632652	3214.738	0.114617	67.16216	0.181519
0.125645	6.497027	43	40434.89	2538165	670.2355	0.058331	117.1966	0.287952
0.12613	6.284338	38.91743	29716.17	2017999	514.8108	0.062116	162.4771	0.248436
0.137755	5.061224	27.28571	592.6071	27958.11	13.54367	0.11405	7.571429	0.270408
0.390792	1.266392	12.78899	650709.3	5029062	95632.39	0.165877	20.85321	0.191314
0.239669	2.818182	18.81818	12739.95	198646.5	874.9277	0.145619	3.818182	0.173554
0.283833	2.514488	25.09368	2086058	48113761	93572.74	0.068039	103.3279	0.241986
0.197874	3.044697	42.52552	3876831	1.85E+08	82840.05	0.034417	275.199	0.180104
0.288101	2.170442	19.77152	386366.3	8958633	17207.76	0.085678	72.23841	0.2392
0.202698	3.006527	23.36057	1186791	28988600	54323.18	0.080537	120.8686	0.214687
0.421624	1.99317	17.33884	233735.5	3129088	18850.3	0.121109	40.4876	0.334608
0.196834	3.578804	36.6413	77634.29	3644245	1684.339	0.049484	40.6413	0.220877
0.314286	11.99164	1856.571	425682.1	7.89E+08	230.0594	0.006249	23.43429	0.13391
0.146582	5.026036	54.49806	437750.9	37165804	5195.552	0.032109	176.2316	0.227984
0.226006	3.221701	55.25752	2868562	1.66E+08	50389.11	0.024128	841.0128	0.284222
0.172296	3.889917	43.06199	2687827	93815411	80873.92	0.041363	39.98113	0.107766
0.158359	5.077773	31.79375	70923.9	3047100	1693.821	0.081302	35.2375	0.220234
0.336095	1.711775	12.1	254678.4	2613491	26074.03	0.156442	42.95385	0.330414
0.259432	3.037517	22.14433	52751.76	1208607	2390.232	0.105251	23.45361	0.24179
0.219667	3.647056	217.6319	2889121	6.55E+08	12787.9	0.005729	365.108	0.223993
0.25037	2.021728	13.73333	14384.51	127984.6	1829.601	0.234741	11.13333	0.247407
0.19548	4.773722	59.66835	876124.8	60090148	12920.07	0.031494	456.9473	0.256424
0.339506	1.719136	18.16667	37657.33	558603.7	2663.05	0.125324	2.222222	0.123457
0.144591	5.477335	49.38288	36874.88	2039013	683.2619	0.046005	39.99099	0.18014
0.10899	7.76	54	91307.57	5736165	1585.205	0.066362	39.18723	0.166754

0.239095	13.56661	786.9713	1747175	1.46E+09	2090.225	0.002892	269.1898	0.297119
0.118564	5.948475	74.88079	2498903	2.13E+08	29966.69	0.022035	766.6202	0.21009
0.254708	6.061792	1018.72	5743296	5.95E+09	5547.088	0.00159	617.308	0.23242
0.152116	156.5433	307.738	1907629	77091519	48220.27	0.027415	578.4595	0.33987
0.328194	1.845336	35.65741	1369521	49901983	37759.67	0.038096	154.6173	0.238607
0.30089	1.686787	9.675799	224552.6	2045276	25363.07	0.279877	45.25571	0.206647
0.252494	3.050681	33.9397	350800	12074734	10363.03	0.052212	36.49749	0.183404
0.272142	2.348153	28.32367	1796377	41585582	80216.05	0.059252	121.058	0.292411
0.323944	2.222178	17.77465	126744.9	1301863	13469.77	0.139754	10.46479	0.147391
0.346633	2.632449	14.48571	103943.4	1473830	7820.65	0.169626	41.74286	0.298163
0.154025	5.001453	46.60733	309942	16773586	5846.574	0.046414	25.23037	0.132096
0.280726	2.370142	25.25392	1158361	28113419	48541.7	0.067844	43.06897	0.135012
0.167162	3.99881	25.02439	91581.15	1408519	7296.386	0.131262	4.707317	0.114813
0.26077	1.916587	27.69677	101944.1	3648181	2874.497	0.054195	31.95484	0.20616
0.326939	1.79831	23.78947	1582849	40492859	62350.91	0.06334	69.84737	0.183809
0.307372	1.758918	12.60345	52834.93	485985.5	5931.007	0.208343	8.034483	0.138526
0.283011	1.930625	14.71242	436405.4	3939343	48680.48	0.143059	24.52941	0.160323
0.163683	4.275369	53.65472	2133700	1.5E+08	31311.84	0.028501	409.4893	0.25754
0.189173	3.686958	33.22672	1668655	59577096	47251.04	0.062049	126.0931	0.154526
0.210059	3.166904	39.88752	565582.4	29434595	11006.79	0.040623	151.1722	0.265681
0.261152	2.583523	46.25755	609076.6	29959032	12414.76	0.029532	189.8058	0.273102
0.120413	6.270982	82.07085	1269230	99614641	17301.37	0.019694	519.9257	0.22324
0.41849	1.313461	25.24845	1272213	31853649	50858.91	0.052076	197.3934	0.204341
0.140625	5.527344	28.6875	794.625	31246.69	23.63903	0.141123	2.5	0.15625
0.280689	3.47146	38.51406	77188.94	3219909	1895.801	0.051696	68.90361	0.276721
0.238358	3.381156	48.80452	2041547	1.12E+08	37800.3	0.031549	239.4086	0.235175
0.204575	4.309328	79.65837	2612191	2.31E+08	29958.85	0.019538	310.4909	0.29712
0.111601	7.831662	61.92423	209131	20473644	2318.642	0.041154	176.3364	0.202453
0.122396	13.25684	60.09375	8327.146	301726.5	232.0697	0.054527	22.89583	0.238498
0.12514	7.393366	47.77215	2439.949	160605.5	152.0347	0.076104	21.3038	0.269668
0.247548	3.176133	37.89202	1311851	60254878	29057.45	0.049412	38.17371	0.179219
0.150724	7.128234	72.55102	61314.29	5133691	742.1504	0.045852	28.03401	0.190708

VX97	VX98	VX99	VX100	VX101	VX102	VX103	VX104	VX105
0.386006	13.2079	0.027562	5.941309	0.016205	431930.4	42.32471	0.000312	23.16309
0.316061	5.141253	0.101305	5.782823	0.00609	1643209	201.5354	0.000237	9.501994
0.466636	43.44766	0.008519	4.933648	0.011781	1400951	24.97619	0.000225	24.74928
0.377984	1.982073	0.240959	3.767894	0.026608	14465.08	8.585795	0.009362	2.09654
0.612617	11.93742	0.074303	4.266957	0.022996	58694.1	15.36992	0.00155	11.63575
0.436583	9.535735	0.051002	5.460096	0.014236	454250.8	46.45799	0.000371	17.34053
0.526915	25.22047	0.02964	5.11318	0.032771	62649.5	8.224569	0.001002	32.83242
0.507297	15.9673	0.035428	5.369721	0.024846	167155.5	25.30924	0.000473	25.29101
0.401845	4.947217	0.0937	4.095795	0.031339	6559.083	5.546435	0.010726	4.135567
0.521897	42.95417	0.00934	5.449925	0.02365	436993.8	27.60849	0.000192	51.30775
0.504078	25.496	0.014086	5.54419	0.015289	3029497	175.2163	4.85E-05	28.16585
0.414456	13.3735	0.019584	5.813592	0.007276	20469925	665.6794	1.93E-05	17.51769
0.426314	14.62056	0.028268	5.774596	0.015862	465037	36.35306	0.000302	22.01505
0.451413	17.48034	0.026238	5.278486	0.025473	37832.52	8.205205	0.001649	21.72543
0.499268	9.111143	0.084639	4.207212	0.023307	27738.8	3.749751	0.003828	15.2455
0.474829	27.33217	0.011722	5.513112	0.014231	2414811	95.40697	7.75E-05	28.34034
0.36668	8.335517	0.035901	4.808017	0.019407	39209.8	11.27068	0.003076	6.817964
0.520401	14.92948	0.032733	5.316187	0.015388	527907.7	63.15225	0.000242	23.43817
0.449024	30.26227	0.01245	6.44901	0.027364	185114.1	25.36949	0.000225	76.87761
0.531481	20.10053	0.026318	5.313325	0.014451	1813036	133.7731	8.75E-05	21.33639
0.495123	17.50662	0.025264	5.606815	0.010304	3185608	253.3396	6.56E-05	16.88429
0.523613	19.99828	0.026029	5.430881	0.022196	337946.1	59.82565	0.000225	26.00273
0.46269	15.23116	0.027764	5.113912	0.021978	61547.9	5.88291	0.002039	18.83158
0.385792	754.3488	0.000287	5.999066	0.005932	5072466	20.61353	7.62E-05	90.68214
0.439438	20.71975	0.016898	5.794706	0.013988	956581.6	44.68161	0.000189	25.51801
0.493506	6.019121	0.112294	4.213658	0.023386	69618.07	12.03237	0.001871	12.32443
0.396585	640.0411	0.010348	4.761853	0.010654	1140890	2.44003	0.000405	102.4884
0.419454	16.74894	0.017087	5.799014	0.011687	469786.5	37.38085	0.000334	24.67424
0.521034	15.10873	0.038004	4.982424	0.019101	169492.5	17.04153	0.000744	18.36861
0.506814	959.8074	0.00027	5.333632	0.021028	171114.3	3.865955	0.000489	67.79175
0.416006	15.71522	0.03222	5.304705	0.031164	32906.28	4.835018	0.002187	23.52943
0.457925	11.77003	0.034582	5.645585	0.012573	1326058	116.4769	0.000147	15.77584
0.549014	14.40378	0.06218	4.506661	0.02686	62690.7	11.43427	0.0015	13.63248
0.410834	20.01129	0.012827	5.83821	0.010152	1103368	64.811	0.000185	23.83715
0.521241	13.26958	0.034667	5.295707	0.009648	10676965	406.0034	2.70E-05	35.33829
0.464188	16.77952	0.026275	4.417321	0.013523	122819.7	9.217311	0.001536	13.81932
0.58134	133.8404	0.00289	5.51061	0.019477	4894436	77.44353	2.02E-05	221.3366
0.4597	8.243444	0.104011	4.797358	0.019455	52909.16	14.60019	0.002193	10.09422
0.36933	4.655331	0.076406	5.13481	0.021968	176077.8	28.16186	0.001107	5.606684
0.325574	10.14161	0.050129	5.70372	0.030774	24334.8	6.433601	0.002081	25.15613
0.444064	20.1723	0.020196	6.10664	0.021301	312453.8	26.9814	0.00029	37.78977
0.496325	30.23167	0.013314	5.866783	0.0144	3201596	151.867	4.58E-05	39.66475
0.413096	13.0589	0.042038	5.859087	0.017818	104449.8	22.67013	0.000704	30.00006
0.421017	18.22522	0.015311	5.980682	0.009525	2989240	105.8192	9.03E-05	23.98981
0.514834	13.10053	0.035607	5.065415	0.013193	1270201	108.342	0.000168	11.29395
0.403885	6.900331	0.056311	5.148937	0.019456	92153.27	15.36554	0.001264	13.42679
0.320357	15.12162	0.010158	5.379065	0.012987	221355.9	10.53801	0.000853	25.06344
0.41458	819.3662	0.000429	5.879846	0.010059	917498.5	10.97347	0.000179	69.8951
0.49432	13.83232	0.046035	5.211386	0.012735	569765.1	60.86011	0.000346	11.49664
0.454883	26.01583	0.016432	5.987183	0.012425	841757	35.46554	0.00021	31.42731
0.430221	32.9605	0.00916	5.864931	0.017457	1281068	40.79822	0.00012	43.46865
0.507191	8.282567	0.110218	4.69619	0.02461	63437.79	21.13647	0.001427	8.07978
0.420864	9.965722	0.042366	5.436333	0.021221	100372.3	18.76429	0.000981	14.9169
0.461558	35.87433	0.009668	5.552653	0.016817	305223.8	11.25392	0.000488	39.73717
0.499305	31.75034	0.017523	6.088601	0.018484	941588	25.95225	0.000118	207.2292
0.445367	28.82435	0.01882	5.995892	0.021486	510581.6	24.65604	0.00023	39.92326
0.479405	25.40089	0.015609	5.364085	0.016042	279770.6	18.09726	0.000471	31.52553

0.487763	14.15728	0.040171	5.03756	0.027593	51508.14	9.218275	0.001511	20.60513
0.460179	8.669235	0.108005	5.076836	0.0202	57029.76	12.41443	0.001947	12.71871
0.427821	23.56233	0.013171	5.746548	0.022458	185765.1	9.002149	0.000535	55.40068
0.433428	23.96567	0.01144	6.018726	0.007053	19097982	368.8064	1.91E-05	32.89255
0.403974	9.641346	0.03837	5.269752	0.020165	60338.94	16.40351	0.001324	15.53509
0.456208	13.12273	0.030495	5.19672	0.015043	721197.6	53.43618	0.000277	13.6379
0.551435	17.47623	0.028458	4.973145	0.013001	1981436	148.7448	0.000107	13.46244
0.559242	16.01503	0.038153	5.062888	0.015	863013.5	97.76165	0.000173	17.32059
0.521202	12.36221	0.064359	4.651044	0.023933	95497.58	14.82598	0.001092	13.61175
0.521563	25.9384	0.025231	4.820073	0.038531	17194.38	3.744186	0.002515	36.07104
0.554135	10.82975	0.128804	4.473023	0.026119	24986.24	6.810107	0.003721	12.94527
0.520997	21.42329	0.020939	5.322803	0.018702	813079.5	88.52026	0.000136	23.11462
0.505232	30.12662	0.011347	5.411929	0.01279	7479480	269.451	2.64E-05	32.19674
0.483124	24.91928	0.017033	6.10119	0.010492	1484016	88.00543	0.000112	48.15847
0.535302	18.94647	0.022746	5.262172	0.013027	4100167	263.5075	4.80E-05	18.5905
0.461934	882.8516	0.002522	5.45548	0.010178	4166183	17.27486	6.38E-05	90.61604
0.490799	18.75765	0.055786	4.921134	0.029813	20052.96	2.695686	0.003868	23.13423
0.504398	22.0109	0.020703	5.486864	0.017597	258950.1	20.27012	0.000458	29.41959
0.374543	17.34514	0.010709	6.08862	0.006254	17517129	416.4184	2.56E-05	18.24538
0.458058	23.14999	0.017677	5.589547	0.022226	149436.2	19.6685	0.000566	25.69465
0.499054	23.63791	0.017271	5.503579	0.014384	1353697	43.32633	0.000126	73.41418
0.538102	11.57602	0.0452	4.780748	0.014307	379472.2	48.92463	0.00048	9.887462
0.584373	51.94997	0.010986	5.784837	0.032065	334555.8	41.22461	0.00013	78.98204
0.481328	10.91638	0.055519	5.507574	0.016354	638359.9	81.49994	0.000204	18.23382
0.550636	28.42444	0.021837	5.322224	0.027996	283694.3	25.81307	0.000286	37.52127
0.509784	23.99949	0.015421	5.477064	0.011434	2632748	81.31868	8.76E-05	34.73314
0.472788	12.23237	0.029552	5.353467	0.010484	10861668	662.5034	2.69E-05	11.87934
0.581757	20.85993	0.027016	4.832081	0.027369	275684.9	46.31931	0.00028	20.69091
0.510041	21.77758	0.021449	5.405226	0.020808	1443906	115.3598	8.13E-05	27.00444
0.457974	9.808876	0.064506	5.630287	0.021214	198619.9	50.68958	0.000414	15.44977
0.522654	19.53498	0.045195	5.045406	0.03851	17135.68	3.404328	0.003276	22.68665
0.48855	15.13768	0.036193	5.612023	0.019635	539836	58.05982	0.000202	23.85894
0.591664	21.50598	0.026127	5.01165	0.012753	2066520	159.2455	8.08E-05	22.99842
0.387725	26.09787	0.019513	6.730552	0.009893	2305028	50.46026	0.000101	151.2845
0.454297	14.6521	0.034422	5.423579	0.01435	288991.1	32.25786	0.000479	18.87246
0.335384	15.29788	0.014673	6.742985	0.007846	1624486	108.3974	0.000103	44.64498
0.487354	19.3736	0.03413	5.248731	0.027169	71203.39	5.950441	0.001205	40.53695
0.472278	6.345783	0.08154	4.931552	0.010201	2038413	180.8486	0.000145	11.52865
0.412416	9.422108	0.036676	4.545467	0.019716	36329.27	11.49985	0.003277	7.059084
0.397935	10.89992	0.040624	5.465727	0.022309	42750.06	13.79099	0.001772	17.1629
0.430291	14.22472	0.034096	5.761456	0.01892	152268.6	27.00937	0.000627	19.36676
0.395439	10.9567	0.027885	5.911359	0.01003	1092855	93.14327	0.000201	15.045
0.28526	7.388846	0.029693	5.528442	0.012572	161013.8	14.51961	0.001166	17.14062
0.431248	17.724	0.017428	5.833922	0.014998	1294225	90.96292	0.000114	24.4526
0.512553	20.67997	0.026336	5.488592	0.026061	154410	19.53416	0.000546	23.75901
0.428163	14.72398	0.035529	5.387666	0.016741	190366.2	20.55844	0.000674	19.51908
0.470341	32.77747	0.010742	5.473732	0.025428	181967	8.935008	0.000524	61.8563
0.492006	27.51808	0.014674	5.269158	0.018753	631319.3	26.31626	0.000278	26.94618
0.423274	15.05408	0.028893	5.05861	0.016771	173212.4	12.1873	0.001046	15.91468
0.464698	21.08406	0.023488	5.625995	0.025737	149952.1	12.72537	0.000615	31.55672
0.388586	11.99695	0.027226	5.97644	0.011954	2382045	167.9747	8.89E-05	15.58916
0.369476	10.92317	0.023994	6.161019	0.008775	6916655	431.9646	3.95E-05	15.81163
0.520231	20.3653	0.026366	5.576036	0.014653	1655708	59.41721	9.16E-05	87.47191
0.369506	10.95173	0.024822	5.303826	0.017281	361843.1	23.33422	0.000613	11.79663
0.362006	676.6254	0.008488	5.917405	0.01111	609810.1	3.655577	0.000378	89.83098
0.588392	49.77476	0.009565	5.036721	0.010786	3469363	111.9439	6.41E-05	29.75703
0.434928	13.76194	0.025604	5.383712	0.019418	314543.6	33.82466	0.000411	17.75804
0.410894	17.3911	0.014485	5.286345	0.014604	1765767	89.96662	0.000107	20.61162

0.574241	28.79053	0.019712	5.263819	0.026435	283291.8	24.57085	0.000266	59.65784
0.367039	13.99612	0.022898	5.895239	0.015926	157130.3	28.89106	0.000607	28.29514
0.51249	30.84759	0.014704	5.704098	0.020382	2014898	46.05448	6.21E-05	194.6757
0.349481	5.439874	0.044422	4.92697	0.012037	426853.9	69.59248	0.000547	6.164787
0.49132	25.92361	0.013559	5.247033	0.031672	124306.9	16.24762	0.000537	30.45418
0.339413	13.10776	0.017089	6.369273	0.006981	4628639	174.0285	7.46E-05	19.3235
0.374861	9.353695	0.047577	5.243112	0.016609	104176	32.31783	0.000953	12.11458
0.537449	15.88685	0.032128	4.773416	0.01447	1060151	85.75637	0.00018	12.40003
0.482067	8.503749	0.049314	4.261579	0.018185	120955.2	19.56815	0.001233	9.540615
0.388538	18.47758	0.020921	5.664359	0.013954	517590.3	24.22624	0.000358	24.39343
0.370402	10.88285	0.025465	6.033772	0.009654	1029510	81.75894	0.000198	19.18941
0.493402	14.83175	0.035526	5.443891	0.028487	122703.7	24.6894	0.000538	23.57615
0.399145	25.23467	0.013248	6.477668	0.011647	1216812	25.8029	0.00016	86.50924
0.372322	15.77587	0.013991	6.087566	0.009795	2705890	107.3676	9.66E-05	21.57315
0.556087	20.77353	0.028566	5.287039	0.020683	1179457	67.68699	0.000118	30.38276
0.389225	16.69769	0.019119	5.893818	0.015946	311316	28.27221	0.000414	24.01251
0.589842	23.95294	0.029457	5.106752	0.022727	581900.4	68.82288	0.000171	23.09564
0.49508	15.09929	0.030612	5.66457	0.008634	8643990	373.4653	3.37E-05	22.69147
0.349158	10.5651	0.029443	5.124415	0.015271	125988.6	15.03328	0.001183	14.43812
0.431602	16.60674	0.019223	5.603347	0.013587	380801.6	22.54913	0.000493	21.38775
0.335179	7.577027	0.067956	5.679029	0.015181	110976.3	34.43423	0.000924	16.0789
0.423919	30.98702	0.010158	5.888087	0.021369	462977.4	20.99401	0.000243	46.87245
0.397203	24.34237	0.018034	5.851272	0.013268	430224.6	14.32867	0.000432	39.52229
0.593836	28.55651	0.029017	4.318354	0.02807	87601.68	10.4566	0.000936	23.12811
0.402298	31.88618	0.008222	6.280182	0.008633	3842478	78.84982	6.06E-05	56.93173
0.422918	13.25348	0.043807	5.712041	0.011583	463563.5	41.64801	0.000413	16.84922
0.540695	13.3059	0.047349	5.102402	0.024827	103208.4	41.3886	0.000605	16.23629
0.554051	21.83813	0.020774	4.733486	0.017015	1038978	64.31987	0.000167	17.64894
0.407901	12.6886	0.053279	5.148033	0.026929	25712.36	4.873385	0.003313	18.12118
0.503807	31.0281	0.016368	5.713856	0.038869	47173.58	7.635845	0.000777	61.18195
0.516637	10.39432	0.075742	4.343943	0.051151	3681.698	2.586144	0.00954	11.26085
0.419749	19.30016	0.015638	5.312292	0.024817	76935.53	9.665455	0.001061	28.35472
0.462112	20.74889	0.017196	5.757307	0.014003	798531.2	48.78273	0.000196	27.90676
0.48658	24.28287	0.016244	5.376071	0.022299	782857.9	74.23342	0.000129	26.08665
0.475499	13.66314	0.035844	4.805704	0.037943	39701.42	12.23694	0.001356	17.23943
0.389955	4.350961	0.072307	4.199699	0.022378	22534.84	11.169	0.004911	4.256715
0.450515	21.44383	0.042027	5.844918	0.016477	66130.21	9.651253	0.001189	40.65478
0.451314	15.86602	0.025695	5.505813	0.014785	467715.3	39.03295	0.000323	22.41593
0.447027	39.3625	0.007351	5.265937	0.012056	1017333	22.72603	0.000234	35.62493
0.422111	17.20473	0.02207	6.152145	0.02091	411879.2	42.59157	0.00021	36.67933
0.475702	17.5258	0.042266	5.280258	0.018587	287297	27.27401	0.000497	16.4888
0.601669	28.37859	0.048313	4.960907	0.048581	11924.24	4.03872	0.002798	29.64652
0.57123	12.52098	0.045613	4.290917	0.02345	320995.4	53.63076	0.000372	10.91273
0.459019	13.46788	0.088003	5.14755	0.05033	3458.376	3.198485	0.006343	24.17507
0.514804	21.71122	0.018649	4.865688	0.013161	350838	14.22493	0.000733	17.48264
0.454868	30.41448	0.01356	6.064622	0.022243	165377.3	14.66749	0.00043	53.31378
0.533925	25.23455	0.023552	5.310851	0.04775	41280.94	9.57788	0.000886	40.30067
0.459469	18.01133	0.019503	5.511238	0.012019	1389701	62.74268	0.00017	19.45496
0.392302	11.81494	0.028636	5.413037	0.021092	103024.5	14.49567	0.001085	15.99228
0.461469	63.15953	0.004875	5.693791	0.014331	1820024	35.50835	0.000101	54.14655
0.48601	14.50039	0.034118	5.35626	0.031829	93259.58	25.34813	0.000585	23.66973
0.522835	15.33821	0.042122	5.378676	0.028435	59838.17	14.34642	0.00104	25.05659
0.522683	8.68198	0.091542	4.03509	0.043738	5336.572	5.442236	0.008135	7.79532
0.524327	27.55978	0.018246	4.778988	0.034865	149292.8	21.46502	0.000448	27.68099
0.525983	31.13973	0.013434	5.158728	0.037652	135220.6	17.96422	0.000381	43.08611
0.519328	9.883878	0.0523	4.477768	0.041983	21471.27	12.7346	0.00247	8.001413
0.489095	24.7049	0.013935	5.6332	0.009508	2153106	147.8916	8.02E-05	21.98378
0.520852	22.50868	0.026836	5.298895	0.039134	46966.49	11.04205	0.000953	29.15634

0.501614	18.74494	0.027129	5.521704	0.023535	397120.4	54.89307	0.000211	25.06368
0.410749	20.10268	0.014054	5.877351	0.014673	326888.2	24.31889	0.000429	25.26444
0.547453	17.03139	0.045494	4.957473	0.043765	37205.63	14.30094	0.00121	18.54653
0.4917	12.81685	0.051094	5.308939	0.020288	160895.6	19.04297	0.000796	14.73912
0.501479	31.66513	0.014137	5.361337	0.035608	293739.8	39.65767	0.000189	36.80238
0.539487	15.88329	0.054448	4.825609	0.034553	57130.92	15.30386	0.00106	17.08831
0.376006	21.9579	0.011086	6.262235	0.012596	1051652	48.83717	0.000162	32.50538
0.529678	27.15807	0.020292	5.552095	0.038081	70280.69	13.1429	0.000621	45.19173
0.549766	80.16636	0.0082	6.121923	0.02756	5977055	87.50109	1.35E-05	1151.629
0.430027	19.95461	0.023395	6.015862	0.032181	164474.3	17.50012	0.000444	31.88931
0.446671	21.06676	0.030312	5.52536	0.037009	33904.46	7.961756	0.001337	28.7648
0.412229	44.89171	0.004638	5.684956	0.011658	13716998	156.1064	2.69E-05	31.67711
0.427783	15.5379	0.024463	5.827785	0.013309	921734.4	80.12231	0.000173	18.75276
0.566488	29.32099	0.017975	5.503045	0.021802	717221.5	76.25107	0.000112	39.45112
0.446636	11.31217	0.049961	4.472054	0.045673	6057.884	4.695067	0.005325	12.92268
0.459643	20.27084	0.019249	5.068307	0.024892	182724.7	23.94872	0.000507	19.7993
0.495797	18.21264	0.032124	5.611054	0.01928	1028656	94.88993	0.000117	23.11083
0.542291	17.12129	0.039692	5.195014	0.035827	198619.4	58.83989	0.000252	22.0822
0.442792	14.69616	0.037208	4.849618	0.023065	85524.71	13.08609	0.001119	15.98295
0.46299	11.52498	0.02813	4.833639	0.02098	98528.5	15.51633	0.001159	13.95857
0.427208	46.04177	0.006552	6.075774	0.009217	1180247	26.96483	0.000197	42.3371
0.433929	14.09711	0.030845	4.350069	0.055046	4288.234	2.387063	0.008925	10.88629
0.476761	12.01177	0.03144	5.193051	0.01393	2290905	205.7108	9.02E-05	11.57965
0.494507	15.20368	0.029015	5.281178	0.015742	471234.9	78.33116	0.000269	14.13558
0.506313	19.02334	0.07285	4.784942	0.072539	653.9779	1.45948	0.012775	40.30791
0.499125	20.79415	0.024471	5.584606	0.024042	188337.7	23.95626	0.000423	30.1131
0.46169	23.23641	0.014735	5.664896	0.00971	2261607	82.47622	0.000112	25.53477
0.525129	12.18029	0.052905	5.012978	0.017674	313380.1	84.62209	0.000348	10.58439
0.498494	30.43869	0.015931	5.441109	0.03416	192962.6	23.23808	0.000332	34.13639
0.482813	15.28001	0.067533	4.909774	0.038095	7276.781	3.397082	0.00486	23.46783
0.550182	25.64894	0.025016	5.168443	0.041716	86424.36	15.65838	0.000518	39.11821
0.423739	10.84807	0.064532	5.98578	0.024067	141930.2	22.10916	0.000545	25.75534
0.550115	22.44531	0.039694	5.507246	0.042757	39887.88	12.15158	0.000793	44.43011
0.501999	19.38061	0.033397	5.747563	0.050196	29319.28	12.06714	0.00075	61.3031
0.518259	7.915595	0.071556	3.99468	0.094915	481.6059	0.640762	0.021643	26.89634
0.430194	5.020913	0.092764	4.409867	0.008712	637532.8	115.6115	0.000522	4.16085
0.26623	5.517654	0.032406	3.970573	0.024202	11032.76	4.414833	0.008317	7.298845
0.498352	12.28763	0.036523	4.782471	0.00982	2075689	113.4225	0.000155	10.93326
0.42232	17.15159	0.016586	5.841176	0.011537	3869317	153.6678	6.56E-05	20.5182
0.493084	9.203636	0.04521	4.60848	0.019137	383635.7	44.1927	0.000474	7.99407
0.465545	10.90758	0.039229	5.44466	0.010906	1178383	91.89234	0.000198	14.51238
0.598017	9.925367	0.077957	3.591913	0.015587	229619.4	49.93345	0.000679	7.104814
0.470683	16.68507	0.026363	5.084062	0.02847	76400.52	9.738148	0.0012	18.80259
0.316491	591.8529	0.000259	5.093669	0.01505	421267.1	2.355694	0.000793	31.24411
0.483443	26.1325	0.016507	5.804372	0.025927	436263.2	24.25195	0.000241	37.34674
0.54841	30.4286	0.013647	5.135338	0.018331	2865586	167.7105	4.73E-05	28.22896
0.303705	12.82252	0.01601	6.326822	0.006269	2662382	69.90093	0.000153	24.39786
0.477964	15.02706	0.049955	5.374203	0.027064	69558.6	11.00065	0.00115	20.40544
0.59526	6.870917	0.097889	3.952346	0.01598	250762.6	56.66634	0.000877	3.851086
0.495919	10.59115	0.064056	4.489449	0.027209	51401.01	11.86086	0.001879	10.09919
0.476154	104.9753	0.002551	5.524296	0.014464	2884341	30.02692	7.66E-05	74.94964
0.481291	6.292085	0.148209	4.045329	0.033015	13467.09	13.0831	0.005051	4.610889
0.517397	31.43423	0.017524	5.586544	0.026253	874673.8	54.09388	0.000109	41.48975
0.304247	5.361361	0.069672	3.794653	0.014706	33033.33	6.278274	0.005214	6.144091
0.415596	19.8553	0.025044	5.810853	0.038112	36186.41	7.36115	0.001255	36.29656
0.408601	20.27073	0.03201	6.212169	0.020967	89032.88	12.75365	0.000723	48.31976

0.559761	436.9304	0.002269	5.172679	0.015857	1743198	3.286665	0.000173	208.6648
0.461505	35.58849	0.009617	6.433142	0.017023	2495452	105.8141	4.78E-05	55.08193
0.486625	492.778	0.001084	5.325668	0.013845	5738079	10.94569	5.29E-05	226.0638
0.604611	232.7855	0.015669	6.217914	0.019691	1905050	19.73732	6.63E-05	1549.309
0.496651	17.33141	0.021391	4.734665	0.015919	1365575	50.62322	0.000227	12.93173
0.44743	4.677449	0.122529	4.676123	0.021589	222407.1	62.40815	0.000866	3.639522
0.408656	11.79306	0.02894	5.082565	0.016181	346980.9	20.12571	0.000615	14.42986
0.555239	14.62319	0.036108	4.644302	0.010322	1786992	108.6473	0.000161	11.94795
0.359813	7.096987	0.043869	4.648577	0.013819	121508	32.94375	0.001409	7.615559
0.561721	7.819358	0.106068	4.035474	0.024125	102225.2	24.00831	0.001145	9.545899
0.353529	15.93145	0.023691	5.989883	0.014127	304931.5	16.00939	0.000595	21.98456
0.34588	8.115133	0.028841	5.569221	0.011137	1150299	58.62152	0.000296	10.60736
0.308253	7.386454	0.066296	4.948896	0.009839	81251.65	18.65531	0.00166	17.45589
0.456745	12.20575	0.029533	4.941115	0.025718	100432.2	10.58061	0.001375	12.92412
0.432167	10.33805	0.028704	5.094393	0.010916	1574456	67.00658	0.00024	10.40467
0.355316	4.424962	0.092706	4.540354	0.021805	50731.6	13.94981	0.003025	6.066923
0.397229	6.758503	0.051512	5.19869	0.014393	431578.3	31.62439	0.001004	6.251819
0.515837	27.56357	0.015449	5.576936	0.013952	2128563	110.1076	8.29E-05	23.92903
0.386657	13.08912	0.023857	6.092366	0.014115	1663635	69.52575	0.000142	24.50804
0.528027	20.58532	0.023232	5.12885	0.020098	563106.8	42.63063	0.000244	20.619
0.529749	24.5629	0.016606	4.824431	0.023643	607287.6	25.8834	0.000309	22.01966
0.472993	38.20286	0.009523	6.189344	0.017217	1265856	77.69568	8.03E-05	53.27778
0.44624	11.4704	0.022224	4.576765	0.021563	1270062	65.31529	0.000239	8.019206
0.361434	8.621006	0.058067	3.875	0.065574	562.0625	0.739252	0.026059	20.57524
0.531507	20.89356	0.032522	4.546435	0.034435	76345.6	15.6955	0.000775	21.96598
0.488489	25.40166	0.014471	5.188267	0.013737	2036247	95.66032	9.51E-05	21.89148
0.561016	44.90875	0.011684	5.124854	0.011821	2605035	70.44931	8.23E-05	35.16613
0.452979	29.97626	0.019739	6.268722	0.030145	208030.5	19.54822	0.000363	39.07111
0.491625	33.87884	0.022853	5.240842	0.064257	8084.954	1.216266	0.00495	89.83352
0.525152	25.73047	0.025638	4.959809	0.070035	2236.074	1.267116	0.009256	35.83811
0.387655	14.81955	0.017915	5.045148	0.008887	1299189	45.93354	0.000267	14.01936
0.433666	32.52502	0.022726	5.390813	0.028253	60061.52	4.169736	0.001447	39.57446

VX106	VX107
0.010525	0.014362
0.023622	0.004839
0.002105	0.03773
0.019269	0.047951
0.021492	0.032639
0.010226	0.014711
0.009026	0.073553
0.015289	0.021168
0.022726	0.08946
0.007919	0.024485
0.008485	0.003515
0.005802	0.00106
0.010111	0.015981
0.021817	0.06876
0.011936	0.170162
0.006185	0.007211
0.027469	0.043972
0.016169	0.01051
0.02326	0.021556
0.019287	0.004293
0.018189	0.002205
0.026204	0.009033
0.007318	0.103749
0.013618	0.127427
0.007033	0.014566
0.007406	0.063733
0.003647	1.9793
0.019566	0.014655
0.010928	0.034754
0.11246	0.384318
0.01524	0.112804
0.012447	0.004943
0.00991	0.047456
0.015178	0.009062
0.004061	0.003865
0.009273	0.074049
0.003559	0.020381
0.01657	0.042245
0.008293	0.01468
0.037835	0.079264
0.012971	0.019805
0.016843	0.003791
0.036904	0.024966
0.007842	0.005618
0.008383	0.005158
0.013398	0.034693
0.00991	0.059534
0.02675	0.13638
0.018499	0.008768
0.010381	0.017336
0.004974	0.019212
0.023007	0.023745
0.02063	0.026342
0.006282	0.076327
0.002661	0.111842
0.01161	0.031222
0.014395	0.031616

0.017067	0.052231
0.016303	0.04764
0.00594	0.076599
0.005377	0.001884
0.025377	0.03239
0.01043	0.010388
0.0104	0.00378
0.017605	0.006253
0.008602	0.040565
0.019902	0.164198
0.015111	0.098116
0.013489	0.006293
0.005276	0.002549
0.022816	0.009257
0.009912	0.002162
0.00527	0.144374
0.01291	0.210068
0.010338	0.028487
0.006084	0.001574
0.016229	0.029074
0.00298	0.039809
0.017681	0.010075
0.016842	0.014826
0.028521	0.00659
0.009218	0.023375
0.004932	0.008984
0.008308	0.000801
0.013639	0.011867
0.00692	0.005245
0.03192	0.009979
0.011466	0.174207
0.015734	0.008886
0.012744	0.003971
0.002882	0.084148
0.022358	0.016235
0.027944	0.005862
0.00725	0.114657
0.005989	0.005935
0.017931	0.042305
0.02998	0.038896
0.026422	0.019646
0.014832	0.005506
0.015875	0.038766
0.014992	0.006425
0.011605	0.028373
0.015982	0.025948
0.003909	0.093565
0.00681	0.024607
0.006522	0.046479
0.009478	0.04782
0.011514	0.003233
0.012216	0.001353
0.003912	0.02865
0.006477	0.02398
0.142104	0.808529
0.007507	0.00682
0.010451	0.015627
0.00449	0.007566

0.00666	0.036255
0.024353	0.023668
0.00138	0.082421
0.010587	0.008454
0.008678	0.035791
0.013013	0.003279
0.03235	0.016065
0.006379	0.007355
0.008539	0.028502
0.006544	0.029509
0.020547	0.006386
0.02419	0.019316
0.004704	0.049119
0.009464	0.005409
0.006995	0.009671
0.025858	0.018837
0.011101	0.008154
0.008151	0.002065
0.015784	0.036064
0.008189	0.025282
0.0348	0.020125
0.0072	0.034121
0.007695	0.0451
0.008877	0.084144
0.00643	0.00853
0.030431	0.013108
0.039799	0.013783
0.005068	0.010859
0.013077	0.108393
0.011288	0.078803
0.023127	0.200886
0.017901	0.060531
0.011836	0.011259
0.009762	0.00836
0.015284	0.041947
0.017633	0.043439
0.044629	0.056745
0.010396	0.014653
0.003377	0.041452
0.02141	0.011868
0.01409	0.022886
0.02909	0.127827
0.011619	0.010126
0.053893	0.174797
0.006232	0.045411
0.017413	0.039465
0.02015	0.054943
0.008607	0.008882
0.016608	0.034836
0.003632	0.028422
0.022951	0.018848
0.019833	0.039223
0.038891	0.087358
0.010197	0.025879
0.009497	0.033733
0.019059	0.040402
0.01299	0.003979
0.018443	0.049793

0.01366	0.009694
0.014811	0.022965
0.024708	0.031341
0.014359	0.029112
0.010735	0.017171
0.02241	0.035175
0.012237	0.013102
0.02513	0.039714
0.000753	0.228016
0.014022	0.033839
0.022987	0.071496
0.00214	0.005728
0.016071	0.006725
0.014051	0.007633
0.027871	0.114001
0.011574	0.024118
0.010845	0.005975
0.018274	0.008487
0.021733	0.053205
0.01254	0.0328
0.013611	0.021056
0.026566	0.236251
0.008047	0.002684
0.022704	0.006741
0.096717	0.408387
0.015083	0.023114
0.008397	0.007377
0.024254	0.006281
0.012342	0.026125
0.042704	0.14789
0.017782	0.035026
0.026392	0.022889
0.030673	0.040918
0.049607	0.04199
0.039367	1.007637
0.013618	0.005158
0.021193	0.103017
0.005095	0.005679
0.00746	0.003778
0.006399	0.012044
0.012032	0.00683
0.009146	0.011589
0.011478	0.059564
0.016367	1.056713
0.009748	0.030957
0.005848	0.003887
0.00743	0.009359
0.01548	0.052522
0.010554	0.008531
0.01223	0.042313
0.002256	0.03558
0.027797	0.040258
0.006212	0.012355
0.009486	0.085725
0.021112	0.072733
0.04693	0.041674

0.000733	0.68451
0.011263	0.005825
0.00045	0.251202
0.001693	2.505944
0.002909	0.011304
0.013341	0.006529
0.004484	0.029873
0.005654	0.005746
0.021788	0.016799
0.008803	0.025662
0.008658	0.040156
0.005609	0.009071
0.033417	0.040864
0.007214	0.050229
0.004211	0.007976
0.011654	0.041311
0.004234	0.026017
0.012884	0.006225
0.005664	0.009291
0.008743	0.016371
0.003178	0.022493
0.01793	0.007329
0.002662	0.007757
0.05935	0.771528
0.011241	0.040886
0.006272	0.007743
0.00541	0.012602
0.051312	0.03013
0.009448	1.718666
0.137322	0.57267
0.004935	0.018162
0.016384	0.162853
